# On the Evolution and Function of *Plasmodium vivax* Reticulocyte Binding Surface Antigen (*pvrbsa*)

**DOI:** 10.3389/fgene.2018.00372

**Published:** 2018-09-10

**Authors:** Paola Andrea Camargo-Ayala, Diego Garzón-Ospina, Darwin Andrés Moreno-Pérez, Laura Alejandra Ricaurte-Contreras, Oscar Noya, Manuel A. Patarroyo

**Affiliations:** ^1^Department of Molecular Biology and Immunology, Fundación Instituto de Inmunología de Colombia (FIDIC), Bogotá, Colombia; ^2^Microbiology Postgraduate Programme, Universidad Nacional de Colombia, Bogotá, Colombia; ^3^PhD Programme in Biomedical and Biological Sciences, Universidad del Rosario, Bogotá, Colombia; ^4^Livestock Sciences Faculty, Universidad de Ciencias Aplicadas y Ambientales, Bogotá, Colombia; ^5^Instituto de Medicina Tropical, Facultad de Medicina, Universidad Central de Venezuela, Caracas, Venezuela; ^6^School of Medicine and Health Sciences, Universidad del Rosario, Bogotá, Colombia

**Keywords:** *Plasmodium vivax*, *rbsa*, genetic diversity, evolutionary forces, protein biding, parasite–host interaction, antimalarial vaccine

## Abstract

The RBSA protein is encoded by a gene described in *Plasmodium* species having tropism for reticulocytes. Since this protein is antigenic in natural infections and can bind to target cells, it has been proposed as a potential candidate for an anti-*Plasmodium vivax* vaccine. However, genetic diversity (a challenge which must be overcome for ensuring fully effective vaccine design) has not been described at this locus. Likewise, the minimum regions mediating specific parasite-host interaction have not been determined. This is why the *rbsa* gene’s evolutionary history is being here described, as well as the *P. vivax rbsa* (*pvrbsa*) genetic diversity and the specific regions mediating parasite adhesion to reticulocytes. Unlike what has previously been reported, *rbsa* was also present in several parasite species belonging to the monkey-malaria clade; paralogs were also found in *Plasmodium* parasites invading reticulocytes. The *pvrbsa* locus had less diversity than other merozoite surface proteins where natural selection and recombination were the main evolutionary forces involved in causing the observed polymorphism. The N-terminal end (PvRBSA-A) was conserved and under functional constraint; consequently, it was expressed as recombinant protein for binding assays. This protein fragment bound to reticulocytes whilst the C-terminus, included in recombinant PvRBSA-B (which was not under functional constraint), did not. Interestingly, two PvRBSA-A-derived peptides were able to inhibit protein binding to reticulocytes. Specific conserved and functionally important peptides within PvRBSA-A could thus be considered when designing a fully-effective vaccine against *P. vivax*.

## Introduction

*Plasmodium vivax* is a parasite which emerged in Asia (Escalante et al., 2005; Carlton et al., 2013) (although an African origin is also likely, Liu et al., 2014), involving a host-switch from monkeys to humans (Mu et al., 2005) around 1.3–2.9 million years ago (Pacheco et al., 2011), *Plasmodium cynomolgi* being the most phylogenetically related species (Mu et al., 2005; Pacheco et al., 2011; Tachibana et al., 2012). *P. vivax* then reached countries on the 5 continents through human migration (Rodrigues et al., 2018), currently predominating in Asia and America (Guerra et al., 2010; Gething et al., 2012). This species is considered the second most important human-malaria parasite worldwide, due to the morbidity it causes (WHO, 2017).

*Plasmodium vivax* incidence has decreased since 2010. An estimated 6–11 million cases were attributed to this parasite in 2016 (around 7 million less than in 2010) (WHO, 2017). Despite that, the social and economic burden of malaria in endemic countries (Suh et al., 2004) could still be huge. Even though control measures against this parasite have been shown to be useful, *P. vivax* elimination (and/or malarial elimination) is not an easy task. The most relevant challenges for eliminating malaria concern the social and economic conditions of the places most affected by this illness, the social and political conflicts in several endemic areas and the anomalous weather patterns (WHO, 2017). These, together with the spread of insecticide-resistant mosquitoes and drug-resistant parasites, could bring about a recurrence of this disease (Maxmen, 2012; Price et al., 2014; Huijben and Paaijmans, 2018). Consequently, new interventions must be designed which can reduce the parasite reservoir, limiting the time that a human (or mosquito) host is infectious (Barry and Arnott, 2014). This goal could be achieved by developing a fully effective antimalarial vaccine which, together with existing control measures, could contribute towards a malaria-free world (Barry and Arnott, 2014; White et al., 2014).

Malaria-related vaccine research and development efforts were exclusively focused on *Plasmodium falciparum* for a long time (Galinski and Barnwell, 2008). During the last few decades, *P. vivax* research has been increased and more and more groups are studying approaches to an anti-*P. vivax* vaccine (Galinski and Barnwell, 2008; Chen et al., 2010; Valencia et al., 2011; The malERA Consultative Group on Vaccines, 2011; Arnott et al., 2012; Patarroyo et al., 2012; Nanda Kumar et al., 2016). Initial research was based on the knowledge acquired regarding *P. falciparum* (Patarroyo et al., 2012); however, given *P. vivax*’s unique attributes (such as hypnozoite forms in the liver, invasion preference for reticulocytes or the rapid gametocyte formation) (Galinski et al., 2013) and since *P. falciparum* and *P. vivax* have had different evolutionary paths, research regarding *P. vivax*-exclusive antigens could be relevant. For instance, the Duffy antigen, which is not present in *P. falciparum*, is essential for *P. vivax* invasion (Chitnis and Miller, 1994). PvDBP-conserved epitopes can trigger strong neutralizing antibodies; it has therefore been suggested as one of the main *P. vivax* vaccine candidates (Chen et al., 2016; Ntumngia et al., 2017).

One antigen shared just by *P. vivax* and *P. cynomolgi* (both having reticulocyte predilection) has been characterized recently (Moreno-Perez et al., 2017). This antigen (named reticulocyte binding surface antigen – RBSA) is encoded by a two-exon gene (Moreno-Perez et al., 2017) located on Chr 3. The protein product has a signal peptide and two transmembrane helices and is located on mature Mrz membrane. This protein seems to be involved in Mrz invasion, since recombinant *P. vivax* RBSA (rPvRBSA) binds to a subpopulation of immature reticulocytes having a Duffy positive phenotype; it is also recognized by the human immune system in natural infections. It has thus been suggested as a putative vaccine candidate for anti-*P. vivax* malaria vaccine development (Moreno-Perez et al., 2017).

However, since parasite’s high genetic diversity is one of the challenges to be overcome during *P. vivax* vaccine design (Neafsey et al., 2012); the antigens’ genetic diversity must therefore be assessed (Arnott et al., 2012; Barry and Arnott, 2014) for selecting those having limited polymorphism or the conserved functional regions within them (Richie and Saul, 2002). *pvrbsa* genetic diversity has not been assessed to date and, although PvRBSA binds to reticulocytes (Moreno-Perez et al., 2017), the regions involved in Mrz-host specific interactions have not been established yet.

PvRBSA is an Mrz membrane protein and is recognized by the human immune system in natural infection; it could therefore be a highly polymorphic antigen. Nevertheless, functionally important parts of the protein (i.e., regions involved in host-parasite interaction) should be functionally constrained, being maintained by negative selection as highly conserved within and between species (Graur et al., 2013; Garzon-Ospina et al., 2015). Consequently, inferring this kind of selection on *pvrbsa* could be used to predict regions under functional constraint which then might be used as putative vaccine candidates. This study evaluated *pvrbsa* genetic diversity, assessed the evolutionary forces involved in causing the observed polymorphism and established minimum regions involved in protein–cell interaction.

## Materials and Methods

### Ethics Approval and Consent to Participate

All *P. vivax*-infected patients who provided us with the blood samples were informed about the purpose of the study and all gave their written consent. Regarding newborn umbilical cord blood samples, the progenitors signed an informed consent form after having received detailed information regarding the study’s goals. All procedures carried out in this study were approved by the ethics committees of the Fundación Instituto de Inmunología de Colombia (IRB number: ACTA N° 037-CEEPA), the Universidad del Rosario (IRB number: CEI-ABN026-0001061) and the Bioethics’ committee from the Instituto de Medicina Tropical “Dr. Félix Pifano” at the Universidad Central de Venezuela (IRB number: CEC-IMT11/2018).

### Parasite DNA

One hundred and sixty-seven peripheral blood samples and/or blood drops collected on FTA cards from patients proving positive for *P. vivax* malaria by microscope examination were collected in different Colombian (Chocó, *n* = 37, Córdoba, *n* = 39, and Amazonas, *n* = 41) and Venezuelan (Bolivar, *n* = 29 and Venezuela’s coastal area, *n* = 19) endemic areas between 2010 and 2016 (**Supplementary Figure S1**). A Wizard Genomic DNA Purification kit (Promega) was used for obtaining DNA from Chocó and Córdoba samples, following the manufacturer’s instructions. A Pure Link Genomic DNA mini kit (Invitrogen) was used for extracting DNA from Amazon samples from each drop of blood collected on the FTA cards, according to the manufacturer’s specifications whilst blood were extracted from Venezuelan samples by salting-out, following Welsh and Bunce modifications (Welsh and Bunce, 1999). The recovered DNAs were stored at −20^°^C until use. All samples had a single *P. vivax* *msp3* allele, suggesting that these samples came from single *P. vivax* infection.

### Identifying the *rbsa* Gene in *Plasmodium* Monkey Lineage

A Blast search (using the 1,506 bp from Sal-I-*P. vivax rbsa* gene sequence as query) was carried out using available whole genome sequences from *P. cynomolgi* (GenBank accession number: GCA_000321355.1), *P. inui* (GenBank accession number: GCA_000524495.1), *P. knowlesi* (GenBank accession number: GCA_000006355.1), *P. coatneyi* (GenBank accession number: GCA_000725905.1) and *P. fragile* (GenBank accession number: GCA_000956335.1) to identify orthologous in these *Plasmodium* species. The MUSCLE method (Edgar, 2004) was used for aligning the recovering contigs having high identity sequences (>60). The best model for DNA substitutions was then selected using the JModelTest v.2.1.3 (Posada, 2008) with the Akaike’s information criterion and MEGA v.6 software (Tamura et al., 2013). Phylogenetic trees were inferred through ML and Bayesian (BY) methods, using the GTR and/or the TVM models (selected as the best models). MEGA v.6 software was used for ML analysis and topology reliability was evaluated by bootstrap, using 1,000 iterations. Bayesian phylogenetic analysis was conducted with MrBayes v.3.2 software (Ronquist et al., 2012) and the analysis was run until reaching a lower than 0.01 standard deviation of split frequencies value; sump and sumt commands were then used for tabulating posterior probabilities and building a consensus tree.

### PCR Amplification and Sequencing

One hundred and sixty-seven parasite DNAs from endemic Colombian (*n* = 117) and Venezuelan (*n* = 50) regions were used for amplifying the *pvrbsa* locus by nested-PCR, as follows. The first reaction involved 3.3 μL of ultrapure water, 5.4 μL of KAPA HiFi HotStart Readymix, 0.3 μM of each primer (*rbsafwd*: 5′-TTTATTTCATTTTGACGTTGTAACTTG-3′ and *rbsarev*: 5′-TTAAGAAATGATCCCAACTCG-3′) and 1 μL of DNA. The PCR was carried out as follows: one 5 min step at 95°C, a second 35 cycle step for 20 s at 98°C, 15 s at 57°C and 45 s at 72°C, followed by a final step of 10 min at 72°C. Two μL of the first PCR product were added to the second PCR reaction containing 7.5 μL of ultrapure water, 12.5 μL of KAPA HiFi HotStart Readymix and 0.3 μM of each primer (*rbsa2fwd*: 5′-GAAATACAAGATGAAAGGAATAATG-3′ and *rbsa2rev*: 5′-GATCCCAACTCGGTTTATC-3′). The thermal conditions were the same as those used in the first PCR reaction. PvRBSA fragments towards the amino and carboxyl (PvRBSA-A and PvRBSA-B, respectively) encoding regions were amplified using KAPA-HiFi HotStart Readymix (KAPA Biosystems) and the genomic DNA previously extracted from the Vivax Colombia Guaviare-I (VCG-I) strain (Moreno-Perez et al., 2014). The 25 μL PCR reaction contained 7.5 μL ultrapure water, 12.5 μL enzyme, 0.3 μM primer (designed taking into account the natural selection signatures observed for *pvrbsa*, PvRBSA-A: *rbsa-a-fwd*: 5′-GGGGTACCACAGCAAGTAGTGAGTCTCT-3′ and *rbsa-a-rev*: 5′-CCCTCGAGCTCACATTCTCCACCACTTAA-3′; PvRBSA-B: *rbsa-b-fwd*: 5′-GGGGTACCCATATAGAAGTAGGATCCGAA-3′ and *rbsa-b-*rev: 5′-CCCTCGAGCAATTGTTCTTCTCCGTATATAT-3′) and 50 ng DNA as template. Temperature cycling conditions involved 1 step of 3 min at 95°C, followed by 35 cycles of 20 sec at 98°C, 15 s at 60°C and 15 s at 72°C, and a final step of 30 s at 72°C. All amplicons were then purified by low-melt agarose gel using the Wizard SV Gel and PCR Clean-Up System (Promega) and then sequenced with a BigDye Terminator kit (Macrogen, Seoul, South Korea) in both directions using the second PCR primers and an internal primer (*rbsaintseq*: 5′-TTTATATTTACACTATTCCTTTGG-3′). Singleton SNPs were confirmed by an independent PCR amplification. The sequences obtained here were deposited in the GenBank database (accession numbers MH391806 - MH391972).

### Obtaining Recombinant Plasmids With RBSA Fragments

*pvrbsa-A* and *pvrbsa-B* PCR products were digested with KpnI (New England Biolabs) and AvaI (NEB) enzymes and then ligated into the pET32b+ vector using T4 ligase (NEB). Briefly, 0.5 μg of each purified product, 1x of CutSmart buffer and 1 U/μL of each enzyme were used in a 25 μL reaction which was incubated for 1 h at 37°C and then inactivated at 80°C for 20 min. Ligation to pET32b+ vector was performed in a 20 μL volume containing 1x T4 buffer, 30 U/μL T4 ligase and vector/product in a 1:3 ratio. The reaction was incubated at 16°C for 16 h and then inactivated at 65°C for 20 min. Each recombinant plasmid was transformed into *Escherichia coli* JM109 cells (Invitrogen) according to the manufacturer’s recommendations and recombinant colonies were then confirmed by PCR using the primers from each product. Three positive colonies were used for extracting plasmids with an UltraClean 6 Minute Mini Plasmid Prep kit (MOBIO), following the manufacturer’s recommendations and then sequenced bidirectionally using *pet32b-fwd*: 5′-CGGTGAAGTGGCGGCAA-3′ and *pet32-Rev*: 5′-CCAAGGGGTTATGCTAGT-3′ primers.

### *pvrbsa* Gene DNA Diversity and Evolutionary Analysis

Three chromatograms (forward, reverse, and internal primer) were obtained from Colombian and Venezuelan samples from sequencing; they were assembled using CLC Main workbench v.3 software (CLC bio, Cambridge, MA, United States). Colombian and Venezuelan sequences were compared and aligned [using the MUSCLE method (Edgar, 2004)] with reference strain sequences (Sal-I GenBank accession number: AAKM01000020.1, Brazil-I GenBank accession number: AFMK01000195.1/AFMK01000194.1, India-VII GenBank accession number: AFBK01001271.1 and North Korean GenBank accession number: AFNJ01000313.1) (Carlton et al., 2008; Neafsey et al., 2012) and with sequences obtained from several sequencing projects (Chan et al., 2012; Hester et al., 2013; Hupalo et al., 2016) available in PlasmoDB database (sequences were screened to rule out those having missing data or ambiguous nucleotides); 232 sequences from different regions around the world were thus used. Afterwards the intron was ruled out from all sequences and codon alignments were inferred using the TranslatorX web server (Abascal et al., 2010). This alignment was manually edited (**Supplementary Data Sheet 1A**) to ensure correct repeat alignment for further analysis.

DnaSP v.5 software (Librado and Rozas, 2009) was used for calculating the amount of singleton sites (s), the amount of parsimony-informative sites (Ps), the amount of haplotypes (H), the haplotype diversity (Hd), the nucleotide polymorphism (or Watterson estimator, θ^w^) and the nucleotide diversity per site (π) for all available sequences (worldwide samples), as well as for the Colombian and Venezuelan populations and for the subpopulations within both populations. Departure from the neutral model of molecular evolution was assessed by Tajima (1989); Fu and Li (1993), and Fay and Wu (2000) estimators. These are frequency spectrum-based tests for comparing two estimators of the population mutation parameter θ which characterizes mutation–drift equilibrium (neutral model). Under neutrality, several unbiased estimators of θ should be equal. Rejection of the neutral expectations suggests that selection or a demographic process could be taking place, the Fay and Wu test being suitable for detecting selective sweep (Fay and Wu, 2000). On the other hand, since each new polymorphic site has a high probability of delineating a new haplotype (Depaulis and Veuille, 1998), tests based on haplotype distribution have been developed [K- and Hd-test (Depaulis and Veuille, 1998) as well as Fu’s Fs (Fu, 1997)]. Similar to frequency spectrum-based tests, departures from neutral expectation could be the consequence of selection or demographic history, Fu’s Fs estimator being a more sensitive indicator of population expansion than Tajima’s test (Fu, 1997). DnaSP v.5 and/or ALLELIX software were used for these tests, coalescent simulations being used for obtaining confidence intervals (Librado and Rozas, 2009). Sites containing gaps or repeats in the alignment were not taken into account.

The aforementioned methods do not consider the classes of mutations (non-synonymous and synonymous); natural selection signatures were therefore also assessed using different methods which classified mutations as non-synonymous or synonymous. The aBSREL method (Smith et al., 2015) was used to test whether positive selection had occurred on a percentage of branches regarding *rbsa* phylogeny. The modified Nei-Gojobori method (Zhang et al., 1998) with Jukes-Cantor correction (Jukes and Cantor, 1969) was then used for calculating the non-synonymous and synonymous substitution difference rate (d_N_-d_S_) within *P. vivax*, using a *Z*-test available in MEGA software v.6 (Tamura et al., 2013) to identify significant statistical values. Two tests were performed for assessing natural selection signals comparing different species; the McDonald–Kreitman test (McDonald and Kreitman, 1991) was calculated using a web server (Egea et al., 2008) using *P. cynomolgi* orthologous sequences. Likewise, non-synonymous divergence and synonymous divergence substitution difference rates (K_N_-K_S_) were also calculated, using the Z-test for identifying statistically significant values. Both tests were carried out taking Jukes-Cantor divergence correction into account.

A sliding window for omega (ω) rates (d_N_/d_S_ and/or K_N_/K_S_) was then used for assessing how natural selection acts throughout the gene. The Datamonkey web server (Delport et al., 2010) was used for assessing codon sites under positive or negative selection by using codon-based ML and BYs [IFEL (Pond et al., 2006), FEL, SLAC, REL (Kosakovsky Pond and Frost, 2005), MEME (Murrell et al., 2012) and FUBAR (Murrell et al., 2013)]. A < 0.1 *p*-value was considered significant for IFEL, FEL, SLAC and MEME methods and a > 0.9 posterior probability for FUBAR. Since ignored recombination can bias d_N_/d_S_ estimation (Anisimova et al., 2003; Arenas and Posada, 2010, 2014), the GARD method (Kosakovsky Pond et al., 2006) was considered regarding recombination before running these tests.

Linkage disequilibrium was evaluated by calculating Z_NS_ (Kelly, 1997) to assess whether recombination was/is taking place in *pvrbsa*; this was followed by linear regression between LD and nucleotide distances. Evidence of recombination was also assessed by the GARD method (Kosakovsky Pond et al., 2006), as well as by ZZ (Rozas et al., 2001) and RM (Hudson and Kaplan, 1985) tests. The degree of genetic differentiation amongst *P. vivax* malaria-endemic regions (subpopulations) regarding the *pvrbsa* locus was evaluated by analysis of molecular variance (AMOVA) and by computing Wright’s fixating index (*F*_ST_), using Arlequin population genetics data analysis v.3.1 software (Excoffier et al., 2007). The mutational pathways giving rise to *pvrbsa* haplotypes, their distribution and frequencies were inferred by median Joining method, using Network v.5 software (Bandelt et al., 1999).

### Assessing Functional Regions in the PvRBSA Protein

Whole PvRBSA, as well as its A and B regions, were recombinantly expressed and purified, as previously described (Moreno-Perez et al., 2017) with minor modifications. For example, the protein was expressed for 4 h at 37°C (for complete PvRBSA) or 30°C (for PvRBSA-A and PvRBSA-B regions) adding 0.2 mM IPTG. The cell pellet was homogenized in B1 buffer (20 mM Tris-Cl, 500 mM NaCl and 1 mM EDTA, pH 8.5) during native protein extraction and then lysed by sonication on ice for 3 min 30 s, with 0.2 s pulses ON, 0.2 s OFF, at 40% amplitude. Protein purification and cell binding assays involved using the same protocols described for PvGAMA and PvRBSA proteins, including PvDBP-RII as positive control and PvDBP-RIII/IV as negative control (Baquero et al., 2017; Moreno-Perez et al., 2017). The competition assay was performed by pre-incubating rPvRBSA-A-derived peptides 40893 (TASSESLAESNDAPSNSYES), 40894 (FPEIRENLTASEESLTSCEE), 40895 (SLTGSNESLTGSNE), 40896 (SLTESRESLEASRESLRASR), 40897 (ESLAASRESLNDFCGSEESV) and 40898 (ACEGEPNEKTFMGDVLSGGE) [synthesized as described (Arevalo-Pinzon et al., 2017)] in a 1:20 (protein:peptide) molar ratio with 2 × 10^7^ red blood cells from umbilical cord blood for 1 hour at 4°C at 4 rpm using a tube rotator (Fisher Scientific). Furthermore, a peptide (P7) from *Mycobacterium tuberculosis* (39266 – APSNETLVKTFSPGEQVTTY) (Carabali-Isajar et al., 2018), was also used as negative control. All cysteine residues were replaced by a threonine to avoid RBSA peptide polymerisation. RBSA reticulocyte binding activity inhibition percentage was quantified by analysing 100,000 events using a FACS Canto II (Biosciences) cytometer and FACSDiva software (BD).

### Statistical Analysis

Differences between medians (*m*) were compared by Kruskal–Wallis test when comparing multiple groups. Statistical significance was assessed by comparing *m*, using a 0.05 significance level. Median values and standard deviations (SD) were calculated from the measurements of three independent experiments.

## Results

### Identifying the *rbsa* Gene in *Plasmodium* Monkey Lineage

The Sal-I-*P. vivax* *rbsa* gene (*pvrbsa*) sequence was used for a Blast search of *P. vivax* phylogenetically related species to identify orthologs in the monkey-parasite lineage. A contig was found in *P. inui* and *P. fragile* having higher than 60% identity whilst two different contigs were found in *P. cynomolgi* having 80% identity with Sal-I *pvrbsa* (**Supplementary Data Sheet 1B**). Interestingly, both *P. cynomolgi* contigs had the same identity but they did not belong to the same chr; one of the two fragments occurred at the chr3 and the other one at chr10. There was 99.57% identity between these *P. cynomolgi* DNA fragments.

**FIGURE 1:**
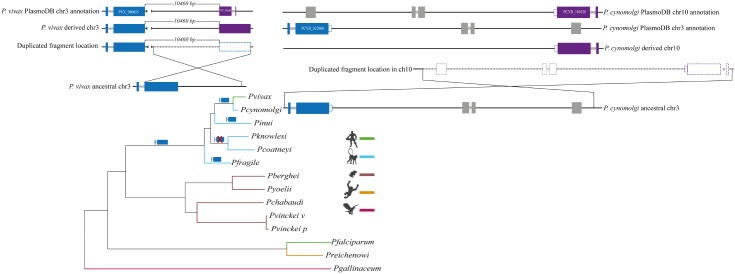
Phylogenetic tree mapping the presence/absence of *rbsa* locus and duplication events. The phylogenetic tree represents *Plasmodium* evolutionary history. The branches are colored regarding their vertebrate host. The *rbsa* locus was found in parasites from monkey-malaria clade, but it was not clear whether the *rbsa* gene became lost from the other clades or arose exclusively in the monkey-malaria clade. *rbsa* became lost in *P. knowlesi* and *P. coatneyi* whilst duplication events occurred in *P. vivax* and *P. cynomolgi*. Models of putative ancestral chromosome 3 are displayed, indicating the arrangement after the duplication event (chr derived). PlasmoDB genome fragment annotation is also shown. *P. cynomolgi rbsa* (*pcrbsa*) and its paralog (*pcrbsap*) had a premature stop codon regarding *P. vivax* *rbsa* sequences, indicated by a white rectangle in the *pcrbsa* and *pcrbsap* gene models.

A similar blast search using Sal-I *pvrbsa* sequence was then performed against other *P. vivax* reference stains’ whole genome sequences (Neafsey et al., 2012), revealing extra DNA fragments having 63% identity at the same chr, lacking the first exon and the intron sequences (**Supplementary Data Sheet 1C**), probably derived from duplication. The duplicated fragment was around 4,000 bp (**Figure 1**, **Supplementary Data Sheet 1C** and **Supplementary Figure S2**), taking upstream and downstream DNA regions into account (Sal-I Ch3 GenBank accession number: AAKM01000020.1) from *pvrbsa* and the *pvrbsa* paralog (*pvrbsap*). Counterparts to this 4,000 bp-DNA fragment were also identified in *P. cynomolgi* (at both chr), *P. inui* and *P. fragile*, which were aligned and used to infer a phylogenetic tree (**Supplementary Figure S2**). Three well-supported groups were identified in the tree; the first one placed *P. vivax* sequences together, the second group clustered *P. cynomolgi* sequences and in the third, *P. inui* and *P. fragile* sequences were found (**Supplementary Figure S2**).

Aligning *P. cynomolgi* *rbsa* (*pcrbsa*, Ch3 GenBank accession number: BAEJ01000151.1) and *pcrbsa* paralogue (*pcrbsap*, Ch10 GenBank accession number: BAEJ01000874.1) DNA fragments revealed a 10 Mb DNA region between chr3 and ch10 (**Figure 1**, **Supplementary Data Sheet 1D** and **Supplementary Figure S2**), having 98.59% identity.

### *pvrbsa* Gene PCR Amplification and Sequencing

One hundred and sixty-seven parasite DNAs from endemic Colombian and Venezuelan regions were used for amplifying the *pvrbsa* locus. The amplicons had 1,454–1,580 bp sizes. These PCR fragments were then purified and sequenced. At least three chromatograms were assembled for each sample, thereby obtaining a consensus sequence.

### *pvrbsa* Gene DNA Diversity and Evolutionary Analysis

Together with the 167 sequences obtained here, 65 additional sequences recovered from PlasmoDB (Colombia *n* = 18, Peru *n* = 16, Brazil *n* = 3, Mexico *n* = 5, Madagascar *n* = 2, China *n* = 2, Cambodia *n* = 2, Thailand *n* = 8, Papua New Guinea *n* = 6, India *n* = 1, North Korea n = 1 and the Sal-I sequences, **Supplementary Data Sheet 1A**) were used to assess genetic diversity in *pvrbsa*. The *pvrbsa* encoding region had 44 segregating sites whilst just 3 polymorphic sites were found at intron worldwide. *pvrbsa* had an intermediate diversity according to the nucleotide diversity estimator (π) (**Table 1**, π < 0.01). The Venezuelan parasite population had a higher π than the Colombian population; both subpopulations within Venezuela (Bolivar and Venezuelan’s Coastal area) had similar π values. Regarding Colombian subpopulations, parasites from Meta (sequences available in PlasmoDB) had the lowest diversity, whilst Córdoba had the highest diversity value for Colombian subpopulations (**Table 1**). The 232 sequences could be clustered into 80 different haplotypes (70 regarding CDS, **Supplementary Data Sheet 1E**). The Colombian population had 37 haplotypes whilst 25 haplotypes were observed in Venezuela (**Table 1**).

**Table 1:** Genetic diversity estimators.

	*n*		Aligned length	S	Ps	H	θw (sd)	π (sd)	Hd (sd)
Worldwide isolates	232	Gene	1,354	15	32	80	0.0067 (0.0010)	0.0085 (0.0002)	0.970 (0.005)
	232	CDS	1,113	14	30	70	0.0066 (0.0010)	0.0080 (0.0002)	0.962 (0.006)
Colombia^a^	135	Gene	1,354	13	26	37	0.0061 (0.0010)	0.0079 (0.0002)	0.951 (0.009)
	135	CDS	1,113	12	24	32	0.0059 ( 0.0010)	0.0074 (0.0002)	0.937 (0.010)
Amazonas^b^	41	Gene	1,391	6	20	13	0.0050 (0.0010)	0.0072 (0.0004)	0.890 (0.028)
	41	CDS	1,170	6	18	13	0.0049 (0.0010)	0.0067 (0.0004)	0.890 (0.028)
Chocó^b^	37	Gene	1,354	14	20	18	0.0072 (0.0012)	0.0079 (0.0005)	0.954 (0.024)
	37	CDS	1,113	13	18	16	0.0069 (0.0012)	0.0074 (0.0005)	0.936 (0.024)
Córdoba^b^	39	Gene	1,405	3	24	19	0.0053 (0.0010)	0.0080 (0.0006)	0.956 (0.023)
	39	CDS	1,170	3	22	17	0.0051 (0.0010)	0.0076 (0.0006)	0.944 (0.024)
Meta^b^	18	Gene	1,506	0	16	8	0.0036 (0.0009)	0.0055 (0.0003)	0.961 (0.033)
	18	CDS	1,290	0	14	8	0.0034 (0.0008)	0.0050 (0.0003)	0.961 (0.033)
Venezuela^a^	50	Gene	1,363	2	28	25	0.0056 (0.0010)	0.0089 (0.0003)	0.958 (0.023)
	50	CDS	1,149	2	26	25	0.0055 (0.0010)	0.0087 (0.0003)	0.958 (0.023)
Bolívar^b^	29	Gene	1,363	0	27	17	0.0059 (0.0011)	0.0088 (0.0005)	0.975 (0.027)
	29	CDS	1,149	0	25	17	0.0057 (0.0011)	0.0085 (0.0005)	0.975 (0.027)
Coastal area^b^	19	Gene	1,363	6	22	13	0.0070 (0.0013)	0.0094 (0.0005)	1.000 (0.036)
	19	CDS	1,149	5	21	13	0.0069 (0.0013)	0.0093 (0.0005)	1.000 (0.036)

*Genetic diversity estimators were calculated using all the *pvrbsa* sequences available (worldwide isolates) as well as for populations (Colombia and Venezuela) and subpopulations (Amazonas, Chocó, Córdoba, Meta, Bolivar, and Venezuela’s coastal area). n, amount of isolates analyzed; aligned length, total sites analyzed, excluding gaps; Ss, amount of segregating sites; Ps, amount of informative parsimonious sites; H, amount of haplotypes; θw, nucleotide polymorphism; π, nucleotide diversity per site; Hd, haplotype diversity. sd, standard deviation. Diversity estimates were corrected for sample size by multiplying by n/(n-1). Although nucleotide diversity was not affected by sample size, it was also corrected. ^a^Populations. ^b^Subpopulations.*

Since different evolutionary forces (drift, selection, recombination and migration) could determine the genetic diversity pattern (Casillas and Barbadilla, 2017) observed in natural populations, several evolutionary tests were performed to infer which of them were modulating the *pvrbsa* gene diversity observed here. Tests based on a neutral molecular evolutionary model were performed for population and subpopulations to assess drift/selection force. The Colombia population had negative statistically significant values just for the Fay and Wu test (**Table 2**) indicating a possible selective sweep. When subpopulations were analyzed separately, Meta had positive values for the Fu and Li test, being statistically significant (*p* < 0.05) which could have resulted from balancing selection or a decrease in population. The Chocó subpopulation had a value lower than 0 (*p* < 0.03) for Fay and Wu’s *H* test (**Table 2**). A sliding window for frequency spectrum-based tests showed regions inside *pvrbsa* having significant values (**Supplementary Figure S3**). On the other hand, the Fu and Li’s F estimator gave statistically significant values for the Venezuelan population; this pattern was also observed for the Bolivar subpopulation (**Table 2**). The sliding window analysis revealed regions inside *pvrbsa* having significant values for Venezuelan subpopulations. Tests based on haplotype distribution did not have statistically significant values (**Table 2**).

**Table 2:** Neutrality tests for Colombia, Venezuela, and subpopulations.

	n		Tajima	Fu and Li		Fay and Wu’s *H* (*p*-value)	Fu’s Fs (*p*-value)	*K*-test (*p*-value)	*H*-test (*p*-value)
			D (*p*-value)	D (*p*-value)	F (*p*-value)				
CDS	135	Colombia^a^	0.637 (*p* > 0.1)	−0.779 (*p* > 0.1)	−0.304 (*p* > 0.1)	−12.819^∗^ (*p* < 0.01)	−3.447 (*p* > 0.1)	32 (*p* > 0.05)	0.930 (*p* > 0.05)
	41	Amazonas^b^	1.192 (*p* > 0.1)	0.262 (*p* > 0.1)	0.789 (*p* > 0.1)	−4.821 (*p* > 0.1)	1.192 (*p* > 0.1)	13 (*p* > 0.05)	0.868 (*p* > 0.05)
	37	Chocó^b^	0.174 (*p* > 0.1)	−0.460 (*p* > 0.1)	−0.285 (*p* > 0.1)	−11.339^∗^ (*p* < 0.03)	−0.755 (*p* > 0.1)	16 (*p* > 0.05)	0.911 (*p* > 0.05)
	39	Córdoba^b^	1.424 (*p* > 0.1)	1.046 (*p* > 0.1)	1.447 (*p* > 0.1)	−5.036 (*p* > 0.1)	−0.758 (*p* > 0.1)	17 (*p* > 0.05)	0.920 (*p* > 0.05)
	18	Meta^b^	1.856 (*p* > 0.1)	1.543^∗^ (*p* < 0.05)	2.118^∗^ (*p* < 0.02)	−1.673 (*p* > 0.1)	1.050 (*p* > 0.1)	8 (*p* > 0.05)	0.908 (*p* > 0.05)
	50	Venezuela^a^	1.865 (*p* > 0.1)	−5.036 (*p* > 0.05)	2.168^∗^ (*p* < 0.02)	−4.140 (*p* > 0.1)	−3.851 (*p* > 0.1)	25 (*p* > 0.05)	0.939 (*p* > 0.05)
	29	Bolívar^b^	1.735 (*p* > 0.05)	1.806^∗^ (*p* < 0.02)	2.236^∗^ (*p* < 0.02)	−5.197 (*p* > 0.1)	−1.969 (*p* > 0.1)	17 (*p* > 0.05)	0.941 (*p* > 0.05)
	19	Coastal area^b^	1.426 (*p* > 0.1)	0.910 (*p* > 0.1)	1.392 (*p* > 0.1)	−4.520 (*p* > 0.1)	−1.196 (*p* > 0.1)	13 (*p* > 0.05)	0.947 (*p* > 0.05)

*The tests were carried out with just the rbsa encoding region (CDS). ^∗^Statistically significant values. ^a^Populations. ^b^Subpopulations.*

**FIGURE 2:**
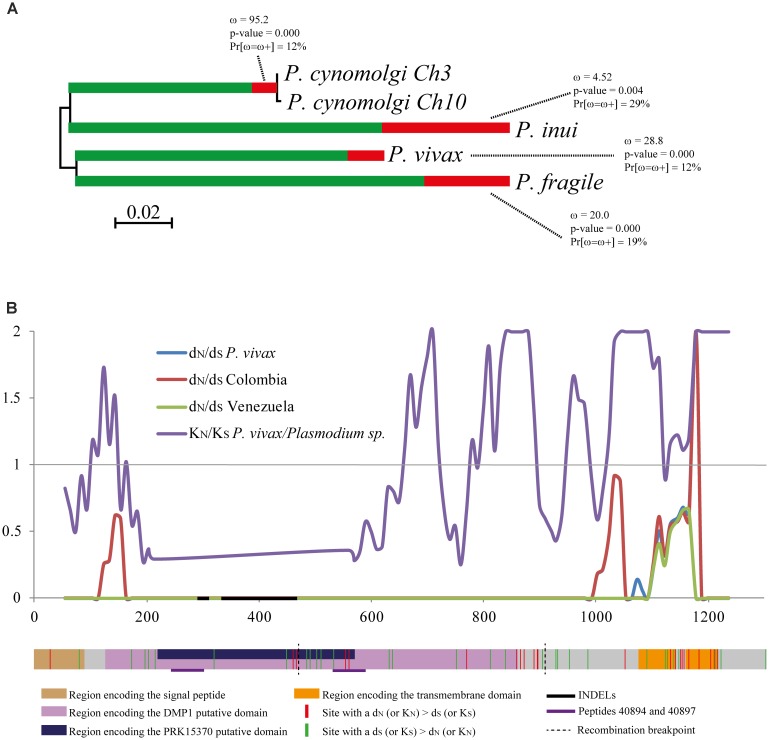
Identifying natural selection signatures in the *rbsa* gene. **(A)** A phylogeny tree inferred from *pvrbsa* CDS and its orthologous CDS was analyzed by the aBSREL method. The shade of each color on branches indicates selection strength [red indicates positive selection (ω > 1) and green negative selection (ω < 1)]. The size of each colored segment represents the percentage of selected sites in the corresponding ω class. The Figure shows the ω values, the percentage of positive selected sites (Pr [ω = ω+]) and *p*-values. Branches have been classified as undergoing episodic diversifying selection, by the *p*-value corrected for multiple testing, using the Holm–Bonferroni method at *p* < 0.05. **(B)** Representation of the *rbsa* encoding DNA sequence and the sliding window for the ω rate. The ω rates within *P. vivax* (d_N_/d_S_) and between the species (K_N_/K_S_) are shown. A gene model of the *rbsa* gene is given below the sliding window, indicating the sites under purifying selection (green) and positive selection (red). Gene model and numbering were based on the **Supplementary Data Sheet 1E** alignment.

A test based on non-synonymous and synonymous mutations was also computed to search natural selection signals in *pvrbsa*. The aBSREL method found evidence of episodic diversifying selection on *rbsa* phylogeny (**Figure 2A**). The d_N_-d_S_ difference had positive values within *P. vivax*; however, they were not statistically significant, except for the Meta subpopulation (**Table 3**). A sliding window was then inferred to assess how d_N_ and d_S_ have been accumulated throughout the *pvrbsa* gene (**Figure 2B**). The 3′-end showed omega (ω) values higher than 1 (**Figure 2B**) which is an indicator of positive selection. The region from nucleotide 200–1,100 had ω = 0 which is expected under negative selection. Although, the McDonald–Kreitman test did not have significant values, the K_N_-K_S_ differences (comparing *P. vivax* sequences to *P. cynomolgi* ones) had positive selection signals (positive significant values, **Table 4**). A similar result was obtained when *P. vivax* and all orthologous sequences from phylogenetically related species were analyzed (**Table 4**). The sliding ω (K_N_/K_S_) window for this dataset showed positive selection signatures (ω > 1) towards the 5′-end, as well as in a region between nucleotide 600 – 1,572 (hereinafter called *Pv*RBSA-B). The region between nucleotide 200 – 600 (hereinafter called *Pv*RBSA-A) had a negative selection signal (ω < 1, **Figure 2B**). Consequently, the gene was split into two regions (PvRBSA-A and PvRBSA-B) and d_N_-d_S_ (as well as K_N_-K_S_) were computed again for each one. No statistically significant values were observed for d_N_-d_S_ in either of the two regions (except for the Meta subpopulation, **Table 3**). Conversely, positive values (indicator of positive selection) having a *p*-value ≤ 0.045 were found in PvRBSA-B when K_N_-K_S_ was computed; by contrast, PvRBSA-A had a negative selection signature (negative values having *p* > 0.05, **Table 4**).

**Table 3:** d_N_-d_S_ difference within *P. vivax.*

Population	PvRBSA-A (196 - 600 bp)	PvRBSA-B (601 – 1,353 bp)	Full length gene
	d_N_ - d_S_ (se)	d_N_ - d_S_ (se)	d_N_ - d_S_ (se)
Worldwide isolates	0.0014 (0.0022)	0.0060 (0.0046)	0.0044 (0.0028)
	*p* = 0.255	*p* = 0.102	*p* = 0.062
Colombian^a^	0.0011 (0.002)	0.0050 (0.004)	0.0036 (0.003)
	*p* = 0.318	*p* = 0.145	*p* = 0.099
Amazonas^b^	0.0016 (0.001)	0.0033 (0.005)	0.0027 (0.003)
	*p* = 0.083	*p* = 0.251	*p* = 0.177
Chocó^b^	0.0023 (0.002)	0.0050 (0.005)	0.0039 (0.003)
	*p* = 0.161	*p* = 0.133	*p* = 0.078
Córdoba^b^	0.0006 (0.004)	0.0036 (0.005)	0.0027 (0.003)
	*p* = 0.441	*p* = 0.811	*p* = 0.173
Meta^b^	−0.0024 (0.004)	0.0082 (0.003)^∗^	0.0045 (0.002)^∗^
	*p* = 0.248	*p* = 0.009	*p* = 0.023
Venezuela^a^	0.0015 (0.002)	0.0064 (0.005)	0.0046 (0.003)
	*p* = 0.269	*p* = 0.120	0.070
Bolívar^b^	0.0023 (0.002)	0.0058 (0.005)	0.0045 (0.003)
	*p* = 0.166	*p* = 0.124	*p* = 0.076
Coastal area^b^	0.0002 (0.003)	0.0070 (0.006)	0.0047 (0.003)
	*p* = 0.472	*p* = 0.099	*p* = 0.081

*Non-synonymous substitution rate (d_N_) and synonymous substitution rate (d_S_) within P. vivax. se, standard error. ^∗^Statistically significant values. ^a^Populations. ^b^Subpopulations.*

**Table 4:** K_N_–K_S_ difference between *Plasmodium* species.

Population	RBSA-A (196 – 600 bp)	RBSA-B (601 – 1,353 bp)	Full length gene
	K_N_ – K_S_ (se)	K_N_ – K_S_ (se)	K_N_ – K_S_ (se)
***P. vivax/P. cynomolgi***			
Worldwide isolates	−0.0010 (0.003)	0.0080 (0.005)^∗^	0.0053 (0.003)^∗^
	*p* = 0.372	*p* = 0.045	*p* = 0.033
Colombian	−0.0030 (0.004)	0.0080 (0.005)^∗^	0.0053 (0.003)^∗^
	*p* = 0.249	*p* = 0.033	*p* = 0.032
Venezuela	−0.0092 (0.010)	0.0150 (0.006)^∗^	0.0090 (0.004)^∗^
	*p* = 0.188	*p* = 0.005	*p* = 0.012
***P. vivax/Plasmodium* sp.**			
Worldwide isolates	−0.0031 (0.004)	0.0101 (0.005)^∗^	0.0068 (0.003)^∗^
	*p* = 0.234	*p* = 0.017	*p* = 0.012
Colombian	−0.0064 (0.007)	0.0118 (0.005)^∗^	0.0077 (0.003)^∗^
	*p* = 0.184	*p* = 0.008	*p* = 0.007
Venezuela	−0.0174 (0.002)	0.0238 (0.006)^∗^	0.0152 (0.005)^∗^
	*p* = 0.167	*p* = 0.0001	*p* = 0.001

*Non-synonymous (K_N_) and synonymous (K_S_) divergence between P. vivax/P. cynomolgi and P. vivax/phylogenetically (P. cynomolgi/P. inui/P. fragile) related species. se, standard error. ^∗^Statistically significant values.*

ω rate was then computed for each codon using codon-based methods. Twenty-one positive selected codons were found between species whilst another 30 were under negative selection. Several of the negative selected sites were found in PvRBSA-A (**Figure 2B**). A protein blast (using NCBI database with Sal-I PvRBSA haplotype as query) gave two putative domains in the regions having ω < 1 (PvRBSA-A). The first belonged to the DMP1 domain and the other to a domain containing a LRR.

Recombination is an evolutionary force which can provide fresh opportunities for overcoming selective pressures to adapt to new environments and/or hosts by linking (within the same DNA region) independently arising variants (Perez-Losada et al., 2015). In fact, it has been observed that diversity levels increase with recombination rate (Hellmann et al., 2003; Kulathinal et al., 2008; Rao et al., 2011). Several tests were thus performed to assess whether this evolutionary force takes/has taken place in *pvrbsa*. A linear regression between LD and nucleotide distance showed that LD decreased regarding increased nucleotide distance, a pattern expected under recombination. Statistically significant ZZ values and several Rm were observed for all populations, thereby confirming recombination action (**Supplementary Data Sheet 2**). Two breakpoints were identified by the GARD method at nucleotides 618 and 1058 (*p* = 0.0004, nucleotide number base in the Sal-I sequence) (**Figure 2**). Recombination was thus taking place at this locus.

**FIGURE 3:**
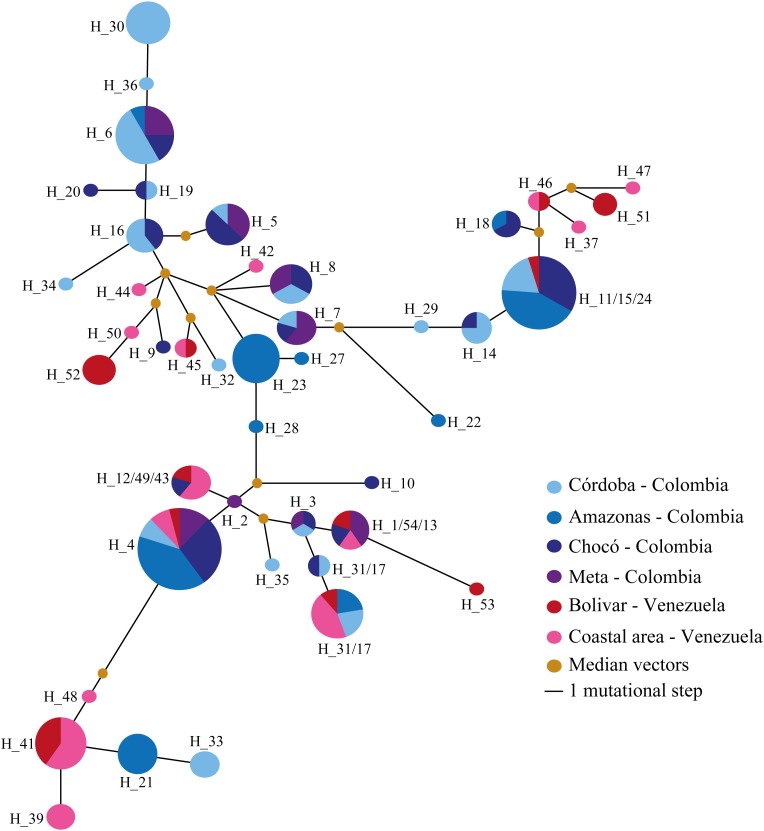
Median-joining network for Colombian and Venezuelan subpopulations. The Figure shows the *pvrbsa* haplotypes identified from Colombian and Venezuelan isolates. Some haplotypes were included within another haplotype using the contraction star algorithm (Forster et al., 2001) for simplifying network interpretation. Each node is a haplotype and its size indicates its frequency. The lines connecting the haplotypes represent the different mutational paths and the median vectors are the ancestral sequences explaining the relationship and evolutionary origin.

A haplotype network was inferred (**Figure 3**) and AMOVA used for assessing whether migration was also involved in shaping *pvrbsa* locus diversity (**Table 5**). Several haplotypes were shared between populations (Colombia and Venezuela, i.e. H_4) but also amongst subpopulations (Chocó, Córdoba, Amazonas, Meta, Bolivar, and the Venezuelan coastal area) lacking clear population structure (**Figure 3**). However, some haplotypes were restricted to particular subpopulations (i.e. H_30, H_23, H_52, H_39) or populations (i.e., H5 - H_8, H_41, H_46). AMOVA was then used to address population differentiation (**Table 5**); analysis showed that the source of variation was between populations (around 4%, *p* = 0.00), the greatest variation (93%) occurring amongst subpopulations (*p* = 0.00). The *F*_ST_ had statistically significant values (**Table 5**). However, comparing Meta/Chocó (as well as Bolivar/Venezuela’s coastal area) revealed no statistically significant *F*_ST_ values (**Table 5**).

**Table 5:** Analysis of molecular variance (AMOVA) analysis and inter-population *F*_ST_ values.

Source of variation				% of variation		*p*-value
Between populations (*F*_CT_)				2.81		0.064
Amongst subpopulations within populations (*F*_SC_)				4.39		0.000^∗^
Amongst subpopulations (*F*_ST_)				92.79		0.001^∗^

***F*_ST_**						

	**Meta**	**Chocó**	**Amazonas**	**Córdoba**	**Bolívar**	**Coastal area**

Meta		0.286	0.004	0.007	0.000	0.000
Chocó	0.007		0.000	0.006	0.000	0.001
Amazonas	0.074^∗^	0.055^∗^		0.000	0.000	0.000
Córdoba	0.041^∗^	0.027^∗^	0.086^∗^		0.000	0.000
Bolívar	0.057^∗^	0.049^∗^	0.083^∗^	0.061^∗^		0.146
Coastal area	0.076^∗^	0.056^∗^	0.094^∗^	0.065^∗^	0.014	

*F_ST_ was calculated for parasite subpopulations within Colombian and Venezuela. Values close to 0 indicate low genetic differentiation whilst values close to 1 indicate high genetic differentiation. Values below the diagonal correspond to the F_ST_ value and those above the diagonal represent the respective p-values. ^∗^Statistically significant values.*

### Assessing PvRBSA Functional Regions

**FIGURE 4:**
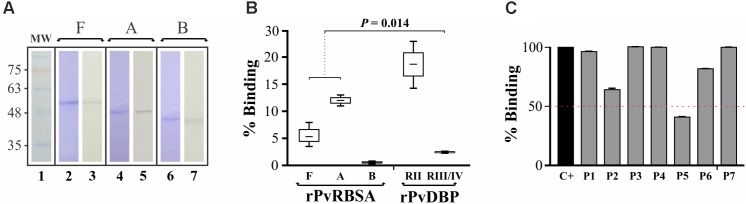
PvRBSA reticulocyte binding activity. **(A)** Purification of full rPvRBSA (F) or its fragments (A and B). The Figure shows each purified molecule analysed by Coomassie blue staining (lines 2, 4, and 6) or Western blot (lines 3, 5, and 7). MW, molecular weight marker (line 1). **(B)** rPvRBSA target cell binding assay. DBP-RII and -RIII/IV indicates positive and negative binding controls, respectively. **(C)** Binding inhibition assay. The Figure shows the rPvRBSA reticulocyte binding inhibition percentage using different PvRBSA-A-derived peptides as well as a *M. tuberculosis* peptide (negative controls). Standard deviation is shown for each assay. C+, shows the assay just with the rPvRBSA protein (positive control). P1-P6 indicate peptides 40893, 40894, 40895, 40896, 40897, and 40898, respectively. P7 is a *M. tuberculosis* peptide (39266) used as negative control. All peptides were pre-incubated with cells before incubating with the complete protein.

It has been shown that immature reticulocytes are *P. vivax* target cells, expressing the CD71 receptor abundantly on their surface (Malleret et al., 2015). Therefore, a reticulocyte-binding assay was performed by flow cytometry to evaluate whether the PvRBSA region predicted under negative selection (PvRBSA-A having a ω < 1) was able to bind to target cells (labeled with anti-CD71 antibody). First, the full PvRBSA protein and the PvRBSA-A and PvRBSA-B regions were recombinantly expressed and obtained in soluble form. Each protein was purified and recognized by WB using a monoclonal anti-polyhistidine antibody (**Figure 4A**). When proteins were incubated with umbilical cord blood (containing 6–7% reticulocytes), only the rPvRBSA (*m* ± SD = 5.5 ± 1.8) and rPvRBSA-A regions (*m* ± SD = 13 ± 1.7) bound to a CD71+CD45- cell population (reticulocytes), unlike the rPvRBSA-B region (**Figure 4B**). As can be observed, rPvRBSA and rPvRBSA-A binding activity had a statistically significant difference compared to negative control (*m* ± SD = 2.4 ± 1.3) (Kruskal–Wallis: *p* = 0.014) (**Figure 4B**). Once the PvRBSA functional region’s reticulocyte interaction activity had been determined, a binding inhibition experiment was performed with PvRBSA-A-derived peptides to search for specific regions within this region involved in interaction with target cells (**Figure 4C**). Peptide 40898 was able to inhibit protein-cell interaction by 18% whilst peptide 40894 produced a 35% reduction. Only one peptide (40897) inhibited rPvRBSA-reticulocyte interaction by more than 50%. The *Mycobacterium* peptide was unable to inhibit rPvRBSA binding activity to reticulocytes. Interestingly, synthesized peptides 40894 and 40897 were conserved in several *P. vivax* isolates and were located in the PRK15370 putative domain (**Figure 2B**).

## Discussion

Malaria remains a public health problem in several tropical and subtropical regions worldwide (WHO, 2017). Although an antimalarial vaccine appears to be a good cost-effective intervention which would help in controlling malaria, a fully effective vaccine has not been developed yet. Since *P. vivax* has a complex biology (Galinski et al., 2013) (for instance, it has tropism for reticulocytes, making it difficult to obtain a continuous *in vitro* culture), antigen identification and characterization for an antimalarial vaccine is a slow task (Patarroyo et al., 2012). Several antigens suggested as potential *P. vivax* vaccine candidates have been characterized, taking into account the candidates proposed for *P. falciparum* or other *Plasmodium* species (Arevalo-Pinzon et al., 2011, 2013, 2015; Patarroyo et al., 2012; Moreno-Perez et al., 2013). However, *P. vivax* and *P. falciparum* have different features and this could be the consequence of their different evolutionary paths. Consequently, several species-specific antigens could be found in both species. These species-specific antigens could thus be taken into account when designing an antimalarial vaccine.

The RBSA has recently been identified in species invading reticulocytes (*P. vivax* and *P. cynomolgi*) suggesting that this antigen could be specific for invading this kind of host cell and could therefore be considered a vaccine candidate when designing an anti-*P. vivax* malaria vaccine (Moreno-Perez et al., 2017). However, this antigen appears not to be exclusive for *P. vivax* and *P. cynomolgi*. A Blast search in whole monkey-malaria lineage genomes showed that *pvrbsa* orthologs were also present in species invading normocytes, such as *P. inui* and *P. fragile* (**Supplementary Data Sheet 1B**). Since the *rbsa* gene was present in *P. fragile* (the most basal species in monkey-malaria lineage, **Figure 1**) (Carlton et al., 2013; Muehlenbein et al., 2015), this gene could be present in the ancestor of all monkey-malaria parasites and, because orthologs were not found in *P. knowlesi* and *P. coatneyi*, then it has been lost in some species within this clade.

The *rbsa* gene was found as a single copy gene in *P. inui* (*pirbsa*) and *P. fragile* (*pfrbsa*), but this gene appears to be duplicated in *P. vivax* (*pvrbsa*) and *P. cynomolgi* (*pcrbsa*). The phylogenetic tree (**Supplementary Figure S2**) showed that *pvrbsa* was closer to *pvrbsap* than *pcrbsa*. This could be the consequence of concerted evolution which can homogenize duplicate gene fragments (Nei and Rooney, 2005). However, duplication was incomplete or was found at different chromosomes; the duplication event must therefore have taken place after *P. vivax* and *P. cynomolgi* divergence. A 4,000 bp fragment was duplicated at chr3 in *P. vivax* (it is present in all *P. vivax* strain genomes available in the PlasmoDB database; duplication and pseudogenisation should therefore have been taking place during early *P. vivax* evolutionary history); this duplicate (*pvrbsap*) lacked the first exon and the intron (**Figure 1**) and could, consequently, be a pseudogene. By contrast, both *pcrbsa* copies were complete in *P. cynomolgi*; in fact, they had 99.91% identity. Furthermore, *pcrbsap* was not found at chr3; instead, it was located at chr10. At least a 10 Mbp were duplicated for this species; however, the mechanism involved in this large fragment’s duplication at a different chr is not clear. Likewise, it is not yet clear whether the high identity found in *pcrbsa* and *pcrbsap* has been due to negative selection acting on both copies or has been due to a recent duplication event. According to the aforementioned results, two independent duplication events must have happened.

On the other hand, the *pvrbsa* gene was amplified in 167 samples from Colombian and Venezuelan populations. The derived sequences were analysed together with 65 sequences from different regions around the world. This gene had length polymorphism due to repeats located at the 5′-end. Worldwide, the *pvrbsa* encoding region had 44 segregating sites (47, taking encoding and non-encoding regions into account) giving 70 haplotypes (**Supplementary Data Sheet 1E**). The *pvrbsa* π value (0.0080 ± 0.0002) was lower than that observed for other Mrz surface proteins [*pvmsp1*, π > 0.05200 (Valderrama-Aguirre et al., 2011; Garzon-Ospina et al., 2015); *pvmsp3α*, π > 0.0349 (Mascorro et al., 2005; Garzon-Ospina et al., 2015); *pvmsp7C*, π = 0.0548; *pvmsp7H*, π = 0.0357; *pvmsp7I*, π = 0.0430 (Garzon-Ospina et al., 2012); *pvmsp7E*, π = 0.0573 (Garzon-Ospina et al., 2014)], but similar to that found in *pvama1* [π = 0.0067 (Gunasekera et al., 2007; Garzon-Ospina et al., 2015)] and *pvdbp* [π = 0.0101 (Nobrega de Sousa et al., 2011)] and higher than *pvmsp7* (*-A* π = 0.0002, *-K* π = 0.0025, *-F* π = 0.0008, and -*L* π = 0.0006) (Garzon-Ospina et al., 2011; Garzon-Ospina et al., 2014), *pvmsp8* (π = 0.0022) (Pacheco et al., 2012), *pvmsp10* (π = 0.0002) (Garzon-Ospina et al., 2011; Pacheco et al., 2012), *pv12* (π = 0.0004), *pv38* (π = 0.0026) and *pv41* (π = 0.0037) (Forero-Rodriguez et al., 2014a,b; Wang et al., 2014) or rhoptry proteins [*rap1*, π = 0.00088, *rap2*, π = 0.00141, and *ron4*, π = 0.0004 (Garzon-Ospina et al., 2010; Pacheco et al., 2010; Buitrago et al., 2016)].

The Venezuelan subpopulations had more genetic diversity than subpopulations within Colombia regarding this gene (**Table 1**); however, the forces causing such diversity seemed to be similar. The sliding windows for frequency spectrum-based tests (**Supplementary Figure S3**) had statistically significant values within *pvrbsa*, suggesting that natural selection seems to be determining the pattern of diversity. The negative values (*p* < 0.02) found between nucleotides 100 and 300 (**Supplementary Figure S3**, numbers based on **Supplementary Data Sheet 1E**) in populations and subpopulations, suggested directional (negative or positive) selection; this kind of selection decreased diversity and could have been a consequence of functional constraint. On the other hand, the 750–940 nucleotide region was under balancing selection, as suggested by positive values for the frequency spectrum-based tests for these positions (**Supplementary Figure S3**). Directional selection was also identified between nucleotides 1,000–1,090 in the Colombian subpopulations whilst balancing selection was also found between nucleotides 1,090–1,200 in the Venezuelan subpopulations (**Supplementary Figure S3**).

Tests based on non-synonymous and synonymous mutations confirmed natural selection. A percentage of sites under positive selection were identified in *P. vivax* and other species regarding the aBSREL method (**Figure 2A**). This pattern has also been observed in other genes, suggesting species-specific adaptation during parasite evolution (Muehlenbein et al., 2015; Buitrago et al., 2016; Garzon-Ospina et al., 2016). Evidence of positive selection was found in PvRBSA-B (**Figure 2** and **Tables 3**, **4**), agreeing with the frequency spectrum-based test results. All this data (**Figure 2**, **Tables 3**, **4**, and **Supplementary Figure S3**) suggested that non-synonymous mutations were maintained in populations by balancing (or diversifying) selection, providing the parasite with an advantage to avoid host immune responses, as has been proposed for other antigens (Garzon-Ospina et al., 2012).

Whilst PvRBSA-B could be involved in host immune evasion, PvRBSA-A might be involved in parasite-host interaction. Negative values were found in d_N_-d_S_ and K_N_-K_S_ tests; however, they were not statistically significant (**Tables 3**, **4**). Nevertheless, statistically significant negative frequency spectrum-based test values were observed. The ω rate was lower than 1 in this region, several codons being under negative selection (**Figure 2**) according codon-based methods; this suggested that negative selection is/has been operating in PvRBSA-A and this region therefore seems to be under functional constraint. This premise was also supported because two putative domains were inferred within this region (**Figure 2B**); one was the DMP1 superfamily domain which is found in dentin matrix protein 1 acting as transcriptional component in mammals (Narayanan et al., 2003). However, how this domain could act in the parasite is not clear yet.

More interesting was the finding of the PRK15370 domain. This domain belongs to the NEL superfamily where family members have an LRR. The LRR has been involved in host-pathogen interaction (Kedzierski et al., 2004). Just PvRBSA-A bound when PvRBSA-A and -B recombinant protein fragments were assessed regarding their ability to bind to human reticulocytes (**Figure 4**). The binding percentage for PvRBSA-A was higher than that for the complete protein, which has been observed for other invasion-related *Plasmodium* parasite antigens (Chitnis and Miller, 1994; Fraser et al., 2001; Kato et al., 2005; Arevalo-Pinzon et al., 2017). An inhibitory assay was then performed to identify minimum specific regions within PvRBSA-A able to inhibit rPvRBSA-reticulocyte interaction. Two peptides (FPEIRENLTASEESLTSCEE and ESLAASRESLNDFCGSEESV) were able to decrease rPvRBSA-reticulocyte interaction. Both peptides were located in the PRK15370 putative domain, the first located towards the N-terminal domain whilst the other one was at the end of this. Remarkably, this region was found in the PvRBSA repeat region; repeats are usually used by the parasite as an immune evasion mechanism. Nevertheless, repeats could also be functionally important, as has been observed in the CS protein (Aldrich et al., 2012; Ferguson et al., 2014). The PvRBSA region involved in parasite-host interaction was thus located between amino acids 76 to 176 (numbers based on Sal-I protein sequences), FPEIRENLTASEESLTSCEE and ESLAASRESLNDFCGSEESV peptides (which were fully conserved in all *P. vivax* sequences analyzed here) being critical for binding, meaning that these peptides could be considered in vaccine development.

The aforementioned results thus suggest that natural selection is an evolutionary force modulating *pvrbsa* genetic diversity whilst negative selection acts at the gene’s 5′-end, the 3′-end is under diversifying or balancing selection. Nonetheless, other forces could also be involved. Recombination might increase diversity by interchanging DNA fragments during sexual reproduction. LD decreased in *pvrbsa* as nucleotide distance increased (**Supplementary Data Sheet 2**), suggesting that recombination was/has been taking place in this gene. Recombination was confirmed by using ZZ, Rm and GARD method (**Supplementary Data Sheet 2**), suggesting that recombination can increase genetic diversity by intra-gene recombination.

A previous study has suggested that American populations are structured (Taylor et al., 2013); this pattern has also been observed in other *P. vivax* antigens in Colombia (Forero-Rodriguez et al., 2014a,b; Buitrago et al., 2016), hence migration was the last force evaluated here. The haplotype network inferred by using all available sequences did not have clear structuring; *pvrbsa* haplotypes were shared by different countries, even around the world. Several haplotypes found on the American continent were also present in Asia; consequently, most haplotypes should have arisen before *P. vivax* spread worldwide. Therefore, due to different *P. vivax* introductions to America by human migration (Rodrigues et al., 2018), *pvrbsa* haplotypes have reached different American counties. A structured population was not found when a haplotype network was inferred. In fact, several haplotypes were shared between Colombia and Venezuela, as well as amongst all subpopulations (**Figure 4**); however, AMOVA suggested that subpopulations were genetically different (**Table 5**). Statistically significant values were found for most comparisons (**Table 5**) when the fixation index (*F*_ST_ based on haplotype diversity) was computed. This could have been the consequence of limited gene flow amongst Colombian subpopulations or due to local adaptation. Synonymous and non-synonymous mutations were analysed independently to try to determine which event(s) led to this structure (**Supplementary Data Sheet 3**). Since synonymous mutations are typically silent, they are considered to follow neutral expectations and, therefore, they could represent the demographic history (migration). On the other hand, non-synonymous mutations are subject to natural selection and could consequently represent local adaptations. The synonymous data set showed that Bolívar and Venezuela’s coastal area in Venezuela as well as the Chocó, Córdoba, Amazonas and Meta departments in Colombia seem to be genetically similar populations since non statistically significant *F*_ST_ were found. However, the non-synonymous data set showed that all (except Bolívar and Venezuela’s coastal area as well as Chocó and Meta) subpopulations were genetically different. This suggested that local adaptation is responsible for the observed structure. However, limited gene flow (partly) might also have provoked such structuring in Colombia. Nevertheless, migration in Venezuela seems to be/have been an important force modulating *pvrbsa* diversity. Further analysis using neutral markers within Colombia and Venezuela populations could confirm this issue.

## Conclusion

Although the RBSA protein has previously been identified as an antigen exclusive to *Plasmodium* species invading reticulocytes (Moreno-Perez et al., 2017), it is actually present in several monkey-malaria lineage species. The encoding gene should have been present in the last monkey-malaria parasite common ancestor and it then became lost in some species (i.e. *P. knowlesi* and *P. coatneyi*). An independent duplication event took place in *P. vivax* and *P. cynomolgi*. The *pvrbsa* paralog appears to be a pseudogene whilst the *pcrbsa* paralog is a functional gene having just one mutation between *pcrbsa* and *pcrbsap*.

The *pvrbsa* locus has lower genetic diversity (π = 0.008) than other Mrz surface proteins (Mascorro et al., 2005; Valderrama-Aguirre et al., 2011; Garzon-Ospina et al., 2012, 2014); this diversity is modulated by natural selection, recombination and migration (the latter for Venezuela but not for Colombia). According to Tajima, Fu and Li, K_N_-K_S_ and codon-based tests the RBSA’s C-terminal end (PvRBSA-B) is under balancing (or diversifying) selection, likely due to this region being involved in immune response evasion whilst PvRBSA-A is under directional selection due to a functional/structural constraint (ω < 1). The latter region has the PRK15370 putative domain (characterized by an LRR) and is involved in host-parasite interaction according to binding assays. Inhibition assays showed that two PRK15370 domain-derived peptides which were conserved in *P. vivax* isolates have been particularly involved in the specific interaction between PvRBSA and reticulocytes. Thus, these minimum regions could be considered when designing a fully effective anti-*P. vivax* vaccine.

## Author Contributions

PC-A performed the molecular evolutionary, recombinant expression and binding assays, and wrote the manuscript. DG-O devised and designed the study, performed the molecular evolutionary analysis, and wrote the manuscript. DM-P devised and designed the study, performed recombinant expression and binding assays, and wrote the manuscript. LR-C performed recombinant expression and binding assays and helped in writing the manuscript. ON and MP coordinated the study and helped to write the manuscript. All the authors have read and approved the final version of the manuscript.

## Conflict of Interest Statement

The authors declare that the research was conducted in the absence of any commercial or financial relationships that could be construed as a potential conflict of interest.

## Abbreviations

ω: omega rate (d_N_/d_S_ and/or K_N_/K_S_)
BY: Bayesian method
Chr: chromosome
LD: linkage disequilibrium
LRR: leucine-rich region
ML: maximum likelihood
Mrz: merozoite
RBSA: reticulocyte binding surface antigen
rRBSA: recombinant RBSA protein
WB: Western blot.
