# Supplementary figures and images for: On the Evolution and Function of Plasmodium vivax Reticulocyte Binding Surface Antigen (pvrbsa)
Source: Front Genet. 2018 Sep 10;9:372. doi: 10.3389/fgene.2018.00372 (PMC6139305; doi:10.3389/fgene.2018.00372)

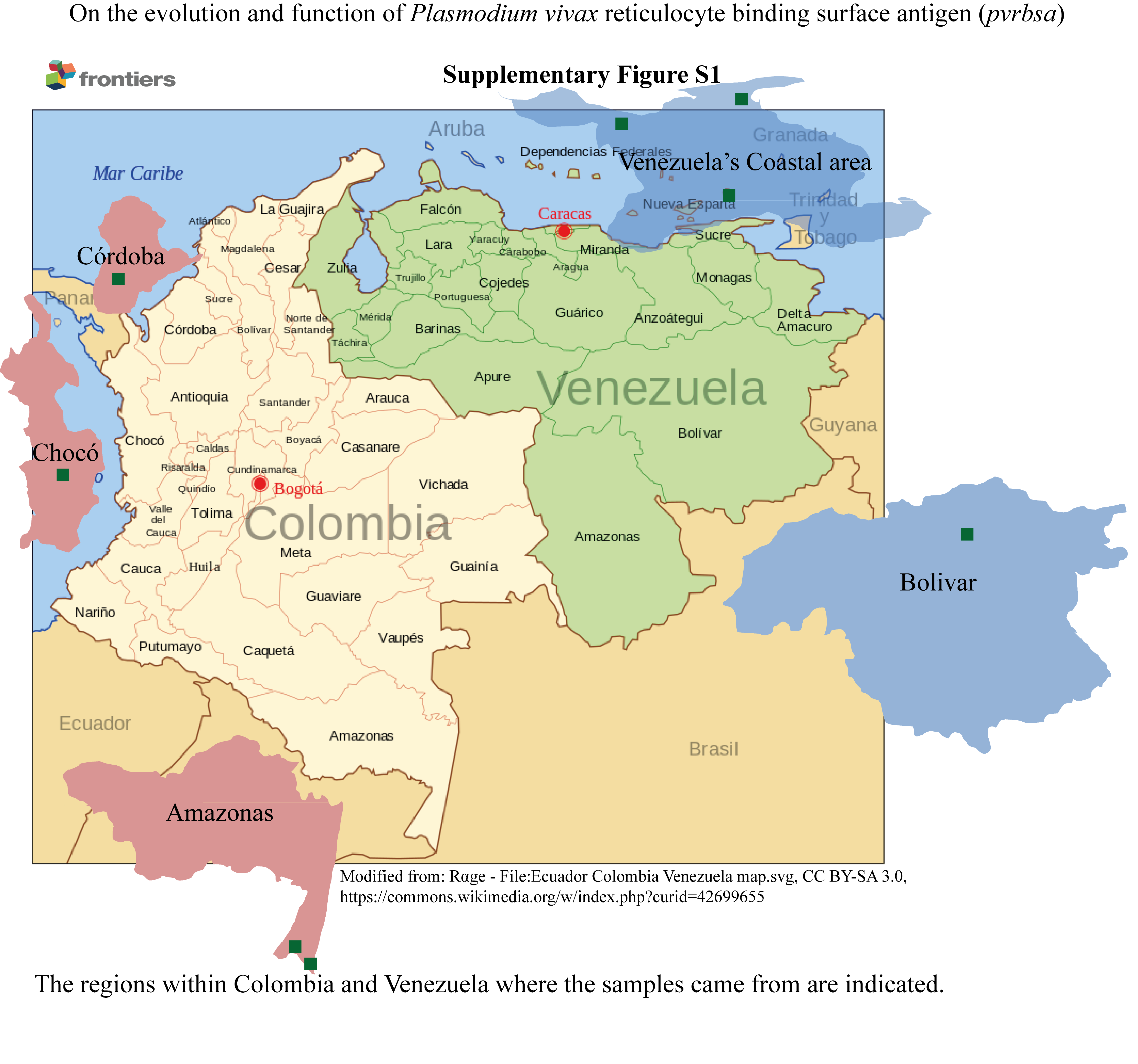

Supplement: Supplementary file 1 [file Image_1.tif]

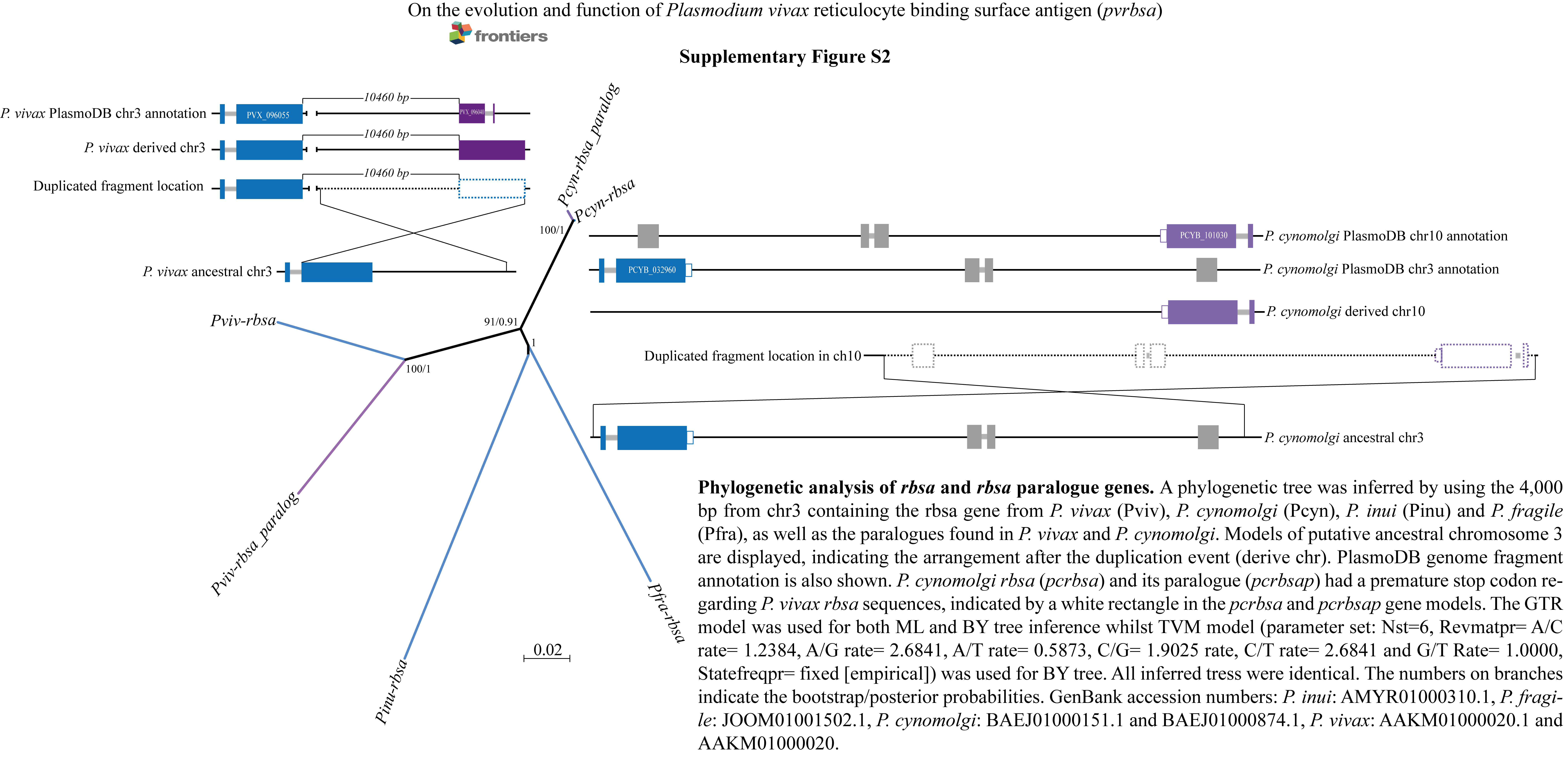

Supplement: Supplementary file 2 [file Image_2.tif]

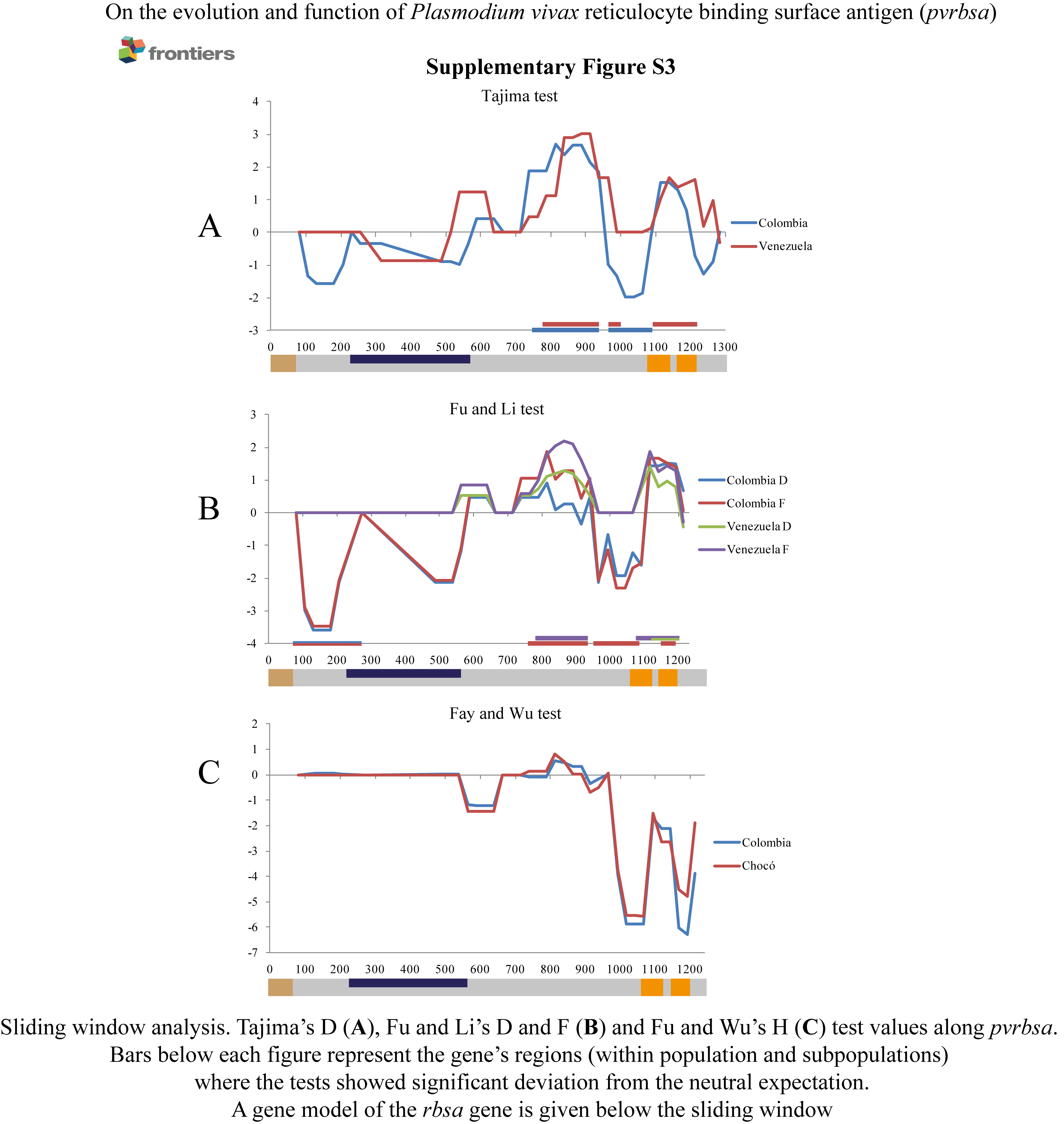

Supplement: Supplementary file 3 [file Image_3.tif]
