# Supplementary material for: On the Evolution and Function of Plasmodium vivax Reticulocyte Binding Surface Antigen (pvrbsa)
Source: Front Genet. 2018 Sep 10;9:372. doi: 10.3389/fgene.2018.00372 (PMC6139305; doi:10.3389/fgene.2018.00372)
Supplement: Supplementary file 4 [file Data_Sheet_1.pdf]

# *Supplementary Data Sheet 1*. On the evolution and function of *Plasmodium vivax* reticulocyte binding surface antigen (*pvrbsa*)

**Paola Andrea Camargo-Ayala, Diego Garzón-Ospina, Darwin Andrés Moreno-Pérez, Laura Alejandra Ricaurte-Contreras, Oscar Noya, Manuel A. Patarroyo***

*** Correspondence:** mapatarr.fidic@gmail.com

**A**. Alignment sequences edited manually in Fasta format.

```
>Pvivax_SalI .
ATGAAAGGAATAATGAATGGTTCCCTTTATTTAAGGAAAATTTCCCTGTGTTCTATTTTC
GTATTGGCATATCTCAGTTCTGCTAAGTGCGCGTACCGAGACGAATCTGACATATTGTAC
AGCGACGACTCCAGATCCGATTTAGCAAGCAACGGGGCACCCACCGACAGCTACGAATCT
TTAACAGCAAGTAGTGAGTCTCTAGCAGAAAGCAACGATGCACCCAGCAACAGCTATGAA
TCTTTTCCAGAAATTAGAGAAAATCTAACCGCAAGTGAGGAATCCCTAACATCATGTGAG
GAATCCCTAACAGGAAGTAATGAATCCCTAACAGGAAGTAATGAATCCCTAACAGGAAGT
AAT---------------------------------------------------------
------GAATCCCTAACAGGAAGTAATGAATCCCTAACAGGAAGTAATGAATCCCTAACA
GAAAGTAGAGAATCTCTAGAGGCCAGTAGAGAATCGCTAAGAGCAAGTAGAGAGTCTCTA
GCGGCCAGTAGAGAATCCCTGAACGACTTTTGTGGGAGCGAAGAATCAGTAGCATTCGAA
GGAGAGCCAAATGAAAAGACATTCATGGGAGACGTCTTAAGTGGTGGAGAATGTGAGAAT
AGTCTCTCAAGAGAAGATTTATTTCATATAGAAGTAGGATCCGAAGAATCGCTAGATGAT
GCCTCAAAATATAATTTCCAAAAGGATTTATCAACTAGCGATAATAGTTCATTCGAAGAT
GATCAGTCATTGAAAAGGGGACTCAAAAGGAACAGCTCAGTATCTAGTCTGGACAGCGAT
ATGGGAAGTTATAAAAATAAAAGTTATAGACACAGATTGGATATATATTCGGACCTACCT
AGAAGGCCAATACATGGGGATAATGCACCACAAGAAAAGGAGAAATGTCTGGATTTCAAA
GAACTTCTAGAGAGAGAAAGGAACAACCACCAAAGGAATAGTGTAAATATAAAATTAAAA
AATATATACGGAGAAGAACAATTGTATAATGTGAAGAAAGATAGCTTTATTGTAAAATTG
ATATACTATTTGCCGTTTGGCCCAATTGTTACAATGATAATGATAGGCTTTATTCTACTC
TGGTCAACGCCCTGGACTGGTTTTATTTTTATAGGGTGTAGCTCTATTATATTACCAGCT
TTGTTATACGCGATGCATTCAAACAAAAAACAATTCGAACGTGTATTTGGAAAAGGGAGA
AAAAAGCCAATGAAGGTAAAAAAAGGAGGCGGGAAAATATCTAGATTTTTTAAAGAAGTA
GACAAATTCTTGTTTGACGATTTGGATGGTTAT
>BrazilI .
ATGAAAGGAATAATGAATGGTTCCCTTTGTTTAAGGAAAATTTCCCTGTGTTCTATTTTC
GTATTGGCATATCTCAGTTCTGCTAAGTGCGCGTACCGAGACGAATCTGACATATTGTAC
AGCGACGACTCCAGATCCGATTTAGCAAGCAACGGGGCACCCACCGACAGCTACGAATCT
TTAACAGCAAGTAGTGAGTCTCTAGCAGAAAGCAACGATGCACCCAGCAACAGCTATGAA
TCTTTTCCAGAAATTAGAGAAAATCTAACCGCAAGTGAGGAATCCCTAACATCATGTGAG
GAATCCCTAACAGGAAGTAATGAATCCCTAACAGGAAGTAATGAATCCCTAACAGGAAGT
AAT---------------------------------------------------------
------GAATCCCTAACAGGAAGTAATGAATCCCTAACAGGAAGTAATGAATCCCTAACA
GAAAGTAGAGAATCTCTAGAGGCCAGTAGAGAATCGCTAAGAGCAAGTAGAGAGTCTCTA
GCGGCCAGTAGAGAATCCCTGAACGACTTTTGTGGGAGCGAAGAATCAGTAGCATTCGAA
GGAGAGCCAAATGAAAAGACATTCATGGGAGACGTCTTAAGTGGTGGAGAATGTGAGAAT
AGTCTCTCAAGAGAAGATTTATTTCATATAGAAGTAGGATCCGAAGAATCGCTAGATGAT
GCCTCAAAATATAATTTCCAAAAGGATTTATCAACTAGCGATAATAGTTCATTCGAAGAT
GATCAGTCATTGAAAAGGGGACTCAAAAGGAACAGCTCAGTATCTAGTCTGGACAGCGAT
ATGGGAAGTTATAAAAATAAAAGTTATAGACACAGATTGGATATATATTCGGACCTACCT
AGAAGGCCAATACATGGGGATAATGCACCACAAGAAAAGGAGAAATGTCTGGATTTCAAA
GAACTTCTAGAGAGAGAAAGGAACAACCACCAAAGGAATAGTGTAAATATAAAATTAAAA
AATATATACGGAGAAGAACAATTGTATAATGTGAAGAAAGATAGCTTTATTGTAAAATTG
ATATACTATCTGCCGTTTGGCCCAATTGTTACAATGATAATGATAGGCTTTATTCTACTC
```

TGCTCAACGCCCTCGACTGGTTTTATTTTTATAGGGTGTAGCTCTATTATATTACCAGCT
TTGTTATACGCGATGCATTCAAACAAAAAACAATTCGAACGTGTATTTGGAAAAGGGAGA
AAAAAGCCAATGAAGGTAAAAAAAGGAGGCGGGAAAATATCTAGATTTTTTAAAGAAGTA
GACAAATTCTTGTTTGACGATTTGGATGGTTAT
>IndiaVII .
ATGAAAGGAATAATGAATGGTTCCCTTTATTTAAGGAAAATTTCCCTGTGTTCTATTTTC
GTATTGGCATATCTCAGTTCTGCTAAGTGCGCGTACCGAGACGAATCTGACATATTGTAC
AGCGACGACTCCAGATCCGATTTAGCAAGCAACGGGGCACCCACCGACAGCTACGAATCT
TTAACAGCAAGTAGTGAGTCTCTAGCAGAAAGCAACGATGCACCCAGCAACAGCTATGAA
TCTTTTCCAGAAATTAGAGAAAATCTAACCGCAAGTGAGGAATCCCTAACATCATGTGAG
GAATCCCTAACAGGAAGTAATGAATCCCTAACAGGAAGTAATGAATCCCTAACAGGAAGT
AAT---------------------------------------------------------
------GAATCCCTAACAGGAAGTAATGAATCCCTAACAGGAAGTAATGAATCCCTAACA
GAAAGTAGAGAATCTCTAGAGGCCAGTAGAGAATCGCTAAGAGCAAGTAGAGAGTCTCTA
GCGGCCAGTAGAGAATCCCTGAACGACTTTTGTGGGAGCGAAGAATCAGTAGCATTCGAA
GGAGAGCCAAATGAAAAGACATTCATGGGAGACGTCTTAAGTGGTGGAGAATGTGAGAAT
AGTCTCTCAAGAGAAGATTTATTTCATATAGAAGTAGGATCCGAAGAATCGCTAGATGAT
GCCTCAAAATATAATTTCCAAAAGGATTTATCAACTAGCGATAATAGTTCATTCGAAGAT
GATCAGTCATTGAAAAGGGGACTCAAAAGGAACAGCTCAGTATCTAGTCTGGACAGCGAT
ATGGGAAGTTATAAAAATAAAAGTTATAGACACAGATTGGATATATATTCGGACCTACCT
AGAAGGCCAATACATGGGGATAATGCACCACAAGAAAAGGAGAAATGTCTGGATTTCAAA
GAACTTCTAGAGAGAGAAAGGAACAACCACCAAAGGAATAGTGTAAATATAAAATTAAAA
AATATATACGGAGAAGAACAATTGTATAATGTGAAGAAAGATAGCTTTATTGTAAAATTG
ATATACTATCTGCCGTTTGGCCCAATTGTTACAATGATAATGATAGGCTTTATTCTACTC
TGCTCAACGCCCTCGACTGGTTTTATTTTTATAGGGTGTAGCTCTATTATATTACCAGCT
TTGTTATACGCGATGCATTCAAACAAAAAACAATTCGAACGTGTATTTGGAAAAGGGAGA
AAAAAGCCAATGAAGGTAAAAAAAGGAGGCGGGAAAATATCTAGATTTTTTAAAGAAGTA
GACAAATTCTTGTTTGACGATTTGGATGGTTAT
>NorthKorean .
ATGAAAGGAATAATGAATGGTTCCCTTTGTTTAAGGAAAATTTCCCTGTGTTCTATTTTC
GTATTGGCATATCTCAGTTCTGCTAAGTGCGCGTACCGAGACGAATCTGACATATTGTAC
AGCGACGACTCCAGATCCGATTTAGCAAGCAACGGGGCACCCACCGACAGCTACGAATCT
TTAACAGCAAGTAGTGAGTCTCTAGCAGAAAGCAACGATGCACCCAGCAACAGCTATGAA
TCTTTTCCAGAAATTAGAGAAAATCTAACCGCAAGTGAGGAATCCCTAACATCATGTGAG
GAATCCCTAACAGGAAGTAATGAATCCCTAACAGGAAGTAATGAATCCCTAACAGGAAGT
AAT---------------------------------------------------------
------GAATCCCTAACAGGAAGTAATGAATCCCTAACAGGAAGTAATGAATCCCTAACA
GAAAGTAGAGAATCTCTAGAGGCCAGTAGAGAATCGCTAAGAGCAAGTAGAGAGTCTCTA
GCGGCCAGTAGAGAATCCCTGAACGACTTTTGTGGGAGCGAAGAATCAGTAGCATGCGAA
GGAGAGCCAAATGAAAAGACATTCATGGGAGACGTCTTAAGTGGTGGAGAATGTGAGAAT
AGTCTCTCAAGAGAAGATTTATTTCATATAGAAGTAGGATCCGAAGAATCGCTAGATGAT
GCCTCAAAATATAATTTCCAAAAGGATTTATCAACTAGCGATAATAGTTCATTCGAAGAT
GATCAGTCATTGAAAAGGGGACTCAAAAGGAACAGCTCAGTATCTAGTCTGGACAGCGAT
ATGGGAAGTTATAAAAATGGAAGATCTAGAGACAGATTGGATATATATTCGGACCTACCT
AGAAGGCTAATACATGGGGATAATGCACCACAAGAAAAGGAGAAATGTCTGGATTTCAAA
GAACTTCTAGAGAGAGAAAGGAACAACCACCAAAGGAATAGTGTAAATATAAAATTAAAA
AATATATACGGAGAAGAACAATTGTATAATGTGAAGAAAGATAGCTTTATTGTAAAATTG
ATATACTATCTGCCGTTTGGCCCAATTGTTACAATGATAATTATAGGCTTTATTCTACTC
TGGTCAACGGGCTGGACTGGTTTTATTTTTATAGGGTGTAGCTCTATTATATTACCAGCT
TTGTTATACGCGATGTATTCAAACAAAAAACAATTCGAACGTGTATTTGGAAAAGGGAGA

```
AAAAAGCCAATGAAGGTAAAAAAAGGAGGCGGGAAAATATCTAGATTTTTTAAAGAAGTA
GACAAATTCTTGTTTGACGATTTGGATGGTTAT
>Mexico_118A .
ATGAAAGGAATAATGAATGGTTCCCTTTGTTTAAGGAAAATTTCCCTGTGTTCTATTTTC
GTATTGGCATATCTCAGTTCTGCTAAGTGCGCGTACCGAGACGAATCTGACATATTGTAC
AGCGACGACTCCAGATCCGATTTAGCAAGCAACGGGGCACCCACCGACAGCTACGAATCT
TTAACAGCAAGTAGTGAGTCTCTAGCAGAAAGCAACGATGCACCCAGCAACAGCTATGAA
TCTTTTCCAGAAATTAGAGAAAATCTAACCGCAAGTGAGGAATCCCTAACATCATGTGAG
GAATCCCTAACAGGAAGTAATGAATCCCTAACAGGAAGTAATGAATCCCTAACAGGAAGT
AAT---------------------------------------------------------
------GAATCCCTAACAGGAAGTAATGAATCCCTAACAGGAAGTAATGAATCCCTAACA
GAAAGTAGAGAATCTCTAGAGGCCAGTAGAGAATCGCTAAGAGCAAGTAGAGAGTCTCTA
GCGGCCAGTAGAGAATCCCTGAACGACTTTTGTGGGAGCGAAGAATCAGTAGCATTCGAA
GGAGAGCCAAATGAAAAGACATTCATGGGAGACGTCTTAAGTGGTGGAGAATGTGAGAAT
AGTCTCTCAAGAGAAGATTTATTTCATATAGAAGTAGGATCCGAAGAATCGCTAGATGAT
GCCTCAAAATATAATTTCCAAAAGGATTTATCAACTAGCGATAATAGTTTATTCGAAGAT
GATCAGTCATTGAAAAGGGGACTCAAAAGGAACAGCTCAGTATCTAGTCTGGACAGCGAT
ATGGGAAGTTATAAAAATGGAAGATCTAGAGACAGATTGGATATATATTCGGACCTACCT
AGAAGGCTAATACATGGGGATAATGCACCACAAGAAAAGGAGAAATGTCTGGATTTCAAA
GAACTTCTAGAGAGAGAAAGGAACAACCACCAAAGGAATAGTGTAAATATAAAATTAAAA
AATATATACGGAGAAGAACAATTGTATAATGTGAAGAAAGATAGCTTTATTGTAAAATTG
ATATACTATCTGCCGTTTGGCCCAATTGTTACAATGATAATGATAGGTTTTTTTCTACTC
TGCTGGACCCCCTCGATTGGTTTTACTTTTATAGGGTGTAGCTCTATTATATTACCAGCT
TTGTTATACGCGATGCATTCAAACAAAAAACAATTCGAACGTGTATTTGGAAAAGGGAGA
AAAAAGCCAATGAAGGTAAAAAAAGGAGGCGGGAAAATATCTAGATTTTTTAAAGAAGTA
GACAAATTCTTGTTTGACGATTTGGATGGTTAT
>Mexico_566A .
ATGAAAGGAATAATGAATGGTTCCCTTTGTTTAAGGAAAATTTCCCTGTGTTCTATTTTC
GTATTGGCATATCTCAGTTCTGCTAAGTGCGCGTACCGAGACGAATCTGACATATTGTAC
AGCGACGACTCCAGATCCGATTTAGCAAGCAACGGGGCACCCACCGACAGCTACGAATCT
TTAACAGCAAGTAGTGAGTCTCTAGCAGAAAGCAACGATGCACCCAGCAACAGCTATGAA
TCTTTTCCAGAAATTAGAGAAAATCTAACCGCAAGTGAGGAATCCCTAACA---------
------------GGAAGTAATGAATCCCTAACAGGAAGTAATGAATCCCTAACAGGAAGT
AAT------------------------------------------GAATCCCTAACAGGA
AGTAATGAATCCCTAACAGGAAGTAATGAATCCCTAACAGGAAGTAATGAATCCCTAACA
GAAAGTAGAGAATCTCTAGAGGCCAGTAGAGAATCGCTAAGAGCAAGTAGAGAGTCTCTA
GCGGCCAGTAGAGAATCCCTGAACGACTTTTGTGGGAGCGAAGAATCAGTAGCATGCGAA
GGAGAGCCAAATGAAAAGACATTCATGGGAGACGTCTTAAGTGGTGGAGAATGTGAGAAT
AGTCTCTCAAGAGAAGATTTATTTCATATAGAAGTAGGATCCGAAGAATCGCTAGATGAT
GCCTCAAAATATAATTTCCAAAAGGATTTATCAACTAGCGATAATAGTTTATTCGAAGAT
GATCAGTCATTGAAAAGGGGACTCAAAAGGAACAGCTCAGTATCTAGTCTGGACAGCGAT
ATGGGAAGTTATAAAAATGGAAGATCTAGAGACAGATTGGATATATATTCGGACCTACCT
AGAAGGCCAATACATGGGGATAATGCACCACAAGAAAAGGAGAAATGTCTGGATTTCAAA
GAACTTCTAGAGAGAGAAAGGAACAACCACCAAAGGAATAGTGTAAATATAAAATTAAAA
AATATATACGGAGAAGAACAATTGTATAATGTGAAGAAAGATAGCTTTATTGTAAAATTG
ATATACTATCTGCCGTTTGGCCCAATTGTTACAATGATAATTATAGGCTTTATTCTACTC
```

TGGTCAACGACTTGGACTGGTTTTATTTTTATAGGGTGTAGCTCTATTATATTACCAGCT
TTGTTATACGCGATGCATTCAAACAAAAAACAATTCGAACGTGTATTTGGAAAAGGGAGA
AAAAAGCCAATGAAGGTAAAAAAAGGAGGCGGGAAAATATCTAGATTTTTTAAAGAAGTA
GACAAATTCTTGTTTGACGATTTGGATGGTTAT
>Mexico_5503 .
ATGAAAGGAATAATGAATGGTTCCCTTTGTTTAAGGAAAATTTCCCTGTGTTCTATTTTC
GTATTGGCATATCTCAGTTCTGCTAAGTGCGCGTACCGAGACGAATCTGACATATTGTAC
AGCGACGACTCCAGATCCGATTTAGCAAGCAACGGGGCACCCACCGACAGCTACGAATCT
TTAACAGCAAGTAGTGAGTCTCTAGCAGAAAGCAACGATGCACCCAGCAACAGCTATGAA
TCTTTTCCAGAAATTAGAGAAAATCTAACCGCAAGTGAGGAATCCCTAACATCATGTGAG
GAATCCCTAACAGGAAGTAATGAATCCCTAACAGGAAGTAATGAATCCCTAACAGGAAGT
AAT---------------------------------------------------------
------GAATCCCTAACAGGAAGTAATGAATCCCTAACAGGAAGTAATGAATCCCTAACA
GAAAGTAGAGAATCTCTAGAGGCCAGTAGAGAATCGCTAAGAGCAAGTAGAGAGTCTCTA
GCGGCCAGTAGAGAATCCCTGAACGACTTTTGTGGGAGCGAAGAATCAGTAGCATTCGAA
GGAGAGCCAAATGAAAAGACATTCATGGGAGACGTCTTAAGTGGTGGAGAATGTGAGAAT
AGTCTCTCAAGAGAAGATTTATTTCATATAGAAGTAGGATCCGAAGAATCGCTAGATGAT
GCCTCAAAATATAATTTCCAAAAGGATTTATCAACTAGCGATAATAGTTTATTCGAAGAT
GATCAGTCATTGAAAAGGGGACTCAAAAGGAACAGCTCAGTATCTAGTCTGGACAGCGAT
ATGGGAAGTTATAAAAATGGAAGATCTAGAGACAGATTGGATATATATTCGGACCTACCT
AGAAGGCTAATACATGGGGATAATGCACCACAAGAAAAGGAGAAATGTCTGGATTTCAAA
GAACTTCTAGAGAGAGAAAGGAACAACCACCAAAGGAATAGTGTAAATATAAAATTAAAA
AATATATACGGAGAAGAACAATTGTATAATGTGAAGAAAGATAGCTTTATTGTAAAATTG
ATATACTATCTGCCGTTTGGCCCAATTGTTACAATGATAATGATAGGTTTTTTTCTACTC
TGCTGGACCCCCTCGATTGGTTTTACTTTTATAGGGTGTAGCTCTATTATATTACCAGCT
TTGTTATACGCGATGCATTCAAACAAAAAACAATTCGAACGTGTATTTGGAAAAGGGAGA
AAAAAGCCAATGAAGGTAAAAAAAGGAGGCGGGAAAATATCTAGATTTTTTAAAGAAGTA
GACAAATTCTTGTTTGACGATTTGGATGGTTAT
>Mexico_20304 .
ATGAAAGGAATAATGAATGGTTCCCTTTGTTTAAGGAAAATTTCCCTGTGTTCTATTTTC
GTATTGGCATATCTCAGTTCTGCTAAGTGCGCGTACCGAGACGAATCTGACATATTGTAC
AGCGACGACTCCAGATCCGATTTAGCAAGCAACGGGGCACCCACCGACAGCTACGAATCT
TTAACAGCAAGTAGTGAGTCTCTAGCAGAAAGCAACGATGCACCCAGCAACAGCTATGAA
TCTTTTCCAGAAATTAGAGAAAATCTAACCGCAAGTGAGGAATCCCTAACATCATGTGAG
GAATCCCTAACAGGAAGTAATGAATCCCTAACAGGAAGTAATGAATCCCTAACAGGAAGT
AAT---------------------------------------------------------
------GAATCCCTAACAGGAAGTAATGAATCCCTAACAGGAAGTAATGAATCCCTAACA
GAAAGTAGAGAATCTCTAGAGGCCAGTAGAGAATCGCTAAGAGCAAGTAGAGAGTCTCTA
GCGGCCAGTAGAGAATCCCTGAACGACTTTTGTGGGAGCGAAGAATCAGTAGCATTCGAA
GGAGAGCCAAATGAAAAGACATTCATGGGAGACGTCTTAAGTGGTGGAGAATGTGAGAAT
AGTCTCTCAAGAGAAGATTTATTTCATATAGAAGTAGGATCCGAAGAATCGCTAGATGAT
GCCTCAAAATATAATTTCCAAAAGGATTTATCAACTAGCGATAATAGTTTATTCGAAGAT
GATCAGTCATTGAAAAGGGGACTCAAAAGGAACAGCTCAGTATCTAGTCTGGACAGCGAT
ATGGGAAGTTATAAAAATGGAAGATCTAGAGACAGATTGGATATATATTCGGACCTACCT
AGAAGGCTAATACATGGGGATAATGCACCACAAGAAAAGGAGAAATGTCTGGATTTCAAA
GAACTTCTAGAGAGAGAAAGGAACAACCACCAAAGGAATAGTGTAAATATAAAATTAAAA
AATATATACGGAGAAGAACAATTGTATAATGTGAAGAAAGATAGCTTTATTGTAAAATTG
ATATACTATCTGCCGTTTGGCCCAATTGTTACAATGATAATGATAGGTTTTTTTCTACTC
TGCTGGACCCCCTCGATTGGTTTTACTTTTATAGGGTGTAGCTCTATTATATTACCAGCT
TTGTTATACGCGATGCATTCAAACAAAAAACAATTCGAACGTGTATTTGGAAAAGGGAGA

AAAAAGCCAATGAAGGTAAAAAAAGGAGGCGGGAAAATATCTAGATTTTTTAAAGAAGTA
GACAAATTCTTGTTTGACGATTTGGATGGTTAT
>Mexico_16104 .
ATGAAAGGAATAATGAATGGTTCCCTTTGTTTAAGGAAAATTTCCCTGTGTTCTATTTTC
GTATTGGCATATCTCAGTTCTGCTAAGTGCGCGTACCGAGACGAATCTGACATATTGTAC
AGCGACGACTCCAGATCCGATTTAGCAAGCAACGGGGCACCCACCGACAGCTACGAATCT
TTAACAGCAAGTAGTGAGTCTCTAGCAGAAAGCAACGATGCACCCAGCAACAGCTATGAA
TCTTTTCCAGAAATTAGAGAAAATCTAACCGCAAGTGAGGAATCCCTAACATCATGTGAG
GAATCCCTAACAGGAAGTAATGAATCCCTAACAGGAAGTAATGAATCCCTAACAGGAAGT
AAT---------------------------------------------------------
------GAATCCCTAACAGGAAGTAATGAATCCCTAACAGGAAGTAATGAATCCCTAACA
GAAAGTAGAGAATCTCTAGAGGCCAGTAGAGAATCGCTAAGAGCAAGTAGAGAGTCTCTA
GCGGCCAGTAGAGAATCCCTGAACGACTTTTGTGGGAGCGAAGAATCAGTAGCATTCGAA
GGAGAGCCAAATGAAAAGACATTCATGGGAGACGTCTTAAGTGGTGGAGAATGTGAGAAT
AGTCTCTCAAGAGAAGATTTATTTCATATAGAAGTAGGATCCGAAGAATCGCTAGATGAT
GCCTCAAAATATAATTTCCAAAAGGATTTATCAACTAGCGATAATAGTTTATTCGAAGAT
GATCAGTCATTGAAAAGGGGACTCAAAAGGAACAGCTCAGTATCTAGTCTGGACAGCGAT
ATGGGAAGTTATAAAAATGGAAGATCTAGAGACAGATTGGATATATATTCGGACCTACCT
AGAAGGCTAATACATGGGGATAATGCACCACAAGAAAAGGAGAAATGTCTGGATTTCAAA
GAACTTCTAGAGAGAGAAAGGAACAACCACCAAAGGAATAGTGTAAATATAAAATTAAAA
AATATATACGGAGAAGAACAATTGTATAATGTGAAGAAAGATAGCTTTATTGTAAAATTG
ATATACTATCTGCCGTTTGGCCCAATTGTTACAATGATAATGATAGGTTTTTTTCTACTC
TGCTGGACCCCCTCGATTGGTTTTACTTTTATAGGGTGTAGCTCTATTATATTACCAGCT
TTGTTATACGCGATGCATTCAAACAAAAAACAATTCGAACGTGTATTTGGAAAAGGGAGA
AAAAAGCCAATGAAGGTAAAAAAAGGAGGCGGGAAAATATCTAGATTTTTTAAAGAAGTA
GACAAATTCTTGTTTGACGATTTGGATGGTTAT
>Peru08 .
ATGAAAGGAATAATGAATGGTTCCCTTTGTTTAAGGAAAATTTCCCTGTGTTCTATTTTC
GTATTGGCATATCTCAGTTCTGCTAAGTGCGCGTACCGAGACGAATCTGACATATTGTAC
AGCGACGACTCCAGATCCGATTTAGCAAGCAACGGGGCACCCACCGACAGCTACGAATCT
TTAACAGCAAGTAGTGAGTCTCTAGCAGAAAGCAACGATGCACCCAGCAACAGCTATGAA
TCTTTTCCAGAAATTAGAGAAAATCTAACCGCAAGTGAGGAATCCCTAACATCATGTGAG
GAATCCCTAACAGGAAGTAATGAATCCCTAACAGGAAGTAATGAATCCCTAACAGGAAGT
AAT---------------------------------------------------------
------GAATCCCTAACAGGAAGTAATGAATCCCTAACAGGAAGTAATGAATCCCTAACA
GAAAGTAGAGAATCTCTAGAGGCCAGTAGAGAATCGCTAAGAGCAAGTAGAGAGTCTCTA
GCGGCCAGTAGAGAATCCCTGAACGACTTTTGTGGGAGCGAAGAATCAGTAGCATTCGAA
GGAGAGCCAAATGAAAAGACATTCATGGGAGACGTCTTAAGTGGTGGAGAATGTGAGAAT
AGTCTCTCAAGAGAAGATTTATTTCATATAGAAGTAGGATCCGAAGAATCGCTAGATGAT
GCCTCAAAATATAATTTCCAAAAGGATTTATCAACTAGCGATAATAGTTCATTCGAAGAT
GATCAGTCATTGAAAAGGGGACTCAAAAGGAACAGCTCAGTATCTAGTCTGGACAGCGAT
ATGGGAAGTTATAAAAATAAAAGTTATAGACACAGATTGGATATATATTCGGACCTACCT
AGAAGGCCAATACATGGGGATAATGCACCACAAGAAAAGGAGAAATGTCTGGATTTCAAA
GAACTTCTAGAGAGAGAAAGGAACAACCACCAAAGGAATAGTGTAAATATAAAATTAAAA
AATATATACGGAGAAGAACAATTGTATAATGTGAAGAAAGATAGCTTTATTGTAAAATTG
ATATACTATCTGCCGTTTGGCCCAATTGTTACAATGATAATGATAGGCTTTATTCTACTC

```
TGGTCAACGGGCTGGACTGGTTTTATTTTTATAGGGTGTAGCTCTATTATATTACCAGCT
TTGTTATACGCGATGTATTCAAACAAAAAACAATTCGAACGTGTATTTGGAAAAGGGAGA
AAAAAGCCAATGAAGGTAAAAAAAGGAGGCGGGAAAATATCTAGATTTTTTAAAGAAGTA
GACAAATTCTTGTTTGACGATTTGGATGGTTAT
>Peru06 .
ATGAAAGGAATAATGAATGGTTCCCTTTATTTAAGGAAAATTTCCCTGTGTTCTATTTTC
GTATTGGCATATCTCAGTTCTGCTAAGTGCGCGTACCGAGACGAATCTGACATATTGTAC
AGCGACGACTCCAGATCCGATTTAGCAAGCAACGGGGCACCCACCGACAGCTACGAATCT
TTAACAGCAAGTAGTGAGTCTCTAGCAGAAAGCAACGATGCACCCAGCAACAGCTATGAA
TCTTTTCCAGAAATTAGAGAAAATCTAACCGCAAGTGAGGAATCCCTAACATCATGTGAG
GAATCCCTAACAGGAAGTAATGAATCCCTAACAGGAAGTAATGAATCCCTAACAGGAAGT
AAT---------------------------------------------------------
------GAATCCCTAACAGGAAGTAATGAATCCCTAACAGGAAGTAATGAATCCCTAACA
GAAAGTAGAGAATCTCTAGAGGCCAGTAGAGAATCGCTAAGAGCAAGTAGAGAGTCTCTA
GCGGCCAGTAGAGAATCCCTGAACGACTTTTGTGGGAGCGAAGAATCAGTAGCATTCGAA
GGAGAGCCAAATGAAAAGACATTCATGGGAGACGTCTTAAGTGGTGGAGAATGTGAGAAT
AGTCTCTCAAGAGAAGATTTATTTCATATAGAAGTAGGATCCGAAGAATCGCTAGATGAT
GCCTCAAAATATAATTTCCAAAAGGATTTATCAACTAGCGATAATAGTTCATTCGAAGAT
GATCAGTCATTGAAAAGGGGACTCAAAAGGAACAGCTCAGTATCTAGTCTGGACAGCGAT
ATGGGAAGTTATAAAAATAAAAGTTATAGACACAGATTGGATATATATTCGGACCTACCT
AGAAGGCCAATACATGGGGATAATGCACCACAAGAAAAGGAGAAATGTCTGGATTTCAAA
GAACTTCTAGAGAGAGAAAGGAACAACCACCAAAGGAATAGTGTAAATATAAAATTAAAA
AATATATACGGAGAAGAACAATTGTATAATGTGAAGAAAGATAGCTTTATTGTAAAATTG
ATATACTATCTGCCGTTTGGCCCAATTGTTACAATGATAATGATAGGCTTTATTCTACTC
TGCTCAACGCCCTCGACTGGTTTTATTTTTATAGGGTGTAGCTCTATTATATTACCAGCT
TTGTTATACGCGATGCATTCAAACAAAAAACAATTCGAACGTGTATTTGGAAAAGGGAGA
AAAAAGCCAATGAAGGTAAAAAAAGGAGGCGGGAAAATATCTAGATTTTTTAAAGAAGTA
GACAAATTCTTGTTTGACGATTTGGATGGTTAT
>Peru07 .
ATGAAAGGAATAATGAATGGTTCCCTTTATTTAAGGAAAATTTCCCTGTGTTCTATTTTC
GTATTGGCATATCTCAGTTCTGCTAAGTGCGCGTACCGAGACGAATCTGACATATTGTAC
AGCGACGACTCCAGATCCGATTTAGCAAGCAACGGGGCACCCACCGACAGCTACGAATCT
TTAACAGCAAGTAGTGAGTCTCTAGCAGAAAGCAACGATGCACCCAGCAACAGCTATGAA
TCTTTTCCAGAAATTAGAGAAAATCTAACCGCAAGTGAGGAATCCCTAACATCATGTGAG
GAATCCCTAACAGGAAGTAATGAATCCCTAACAGGAAGTAATGAATCCCTAACAGGAAGT
AAT---------------------------------------------------------
------GAATCCCTAACAGGAAGTAATGAATCCCTAACAGGAAGTAATGAATCCCTAACA
GAAAGTAGAGAATCTCTAGAGGCCAGTAGAGAATCGCTAAGAGCAAGTAGAGAGTCTCTA
GCGGCCAGTAGAGAATCCCTGAACGACTTTTGTGGGAGCGAAGAATCAGTAGCATTCGAA
GGAGAGCCAAATGAAAAGACATTCATGGGAGACGTCTTAAGTGGTGGAGAATGTGAGAAT
AGTCTCTCAAGAGAAGATTTATTTCATATAGAAGTAGGATCCGAAGAATCGCTAGATGAT
GCCTCAAAATATAATTTCCAAAAGGATTTATCAACTAGCGATAATAGTTCATTCGAAGAT
GATCAGTCATTGAAAAGGGGACTCAAAAGGAACAGCTCAGTATCTAGTCTGGACAGCGAT
ATGGGAAGTTATAAAAATAAAAGTTATAGACACAGATTGGATATATATTCGGACCTACCT
AGAAGGCCAATACATGGGGATAATGCACCACAAGAAAAGGAGAAATGTCTGGATTTCAAA
GAACTTCTAGAGAGAGAAAGGAACAACCACCAAAGGAATAGTGTAAATATAAAATTAAAA
AATATATACGGAGAAGAACAATTGTATAATGTGAAGAAAGATAGCTTTATTGTAAAATTG
ATATACTATCTGCCGTTTGGCCCAATTGTTACAATGATAATGATAGGCTTTATTCTACTC
TGCTCAACGCCCTCGACTGGTTTTATTTTTATAGGGTGTAGCTCTATTATATTACCAGCT
TTGTTATACGCGATGCATTCAAACAAAAAACAATTCGAACGTGTATTTGGAAAAGGGAGA
```

AAAAAGCCAATGAAGGTAAAAAAAGGAGGCGGGAAAATATCTAGATTTTTTAAAGAAGTA
GACAAATTCTTGTTTGACGATTTGGATGGTTAT
>Peru257 .
ATGAAAGGAATAATGAATGGTTCCCTTTGTTTAAGGAAAATTTCCCTGTGTTCTATTTTC
GTATTGGCATATCTCAGTTCTGCTAAGTGCGCGTACCGAGACGAATCTGACATATTGTAC
AGCGACGACTCCAGATCCGATTTAGCAAGCAACGGGGCACCCACCGACAGCTACGAATCT
TTAACAGCAAGTAGTGAGTCTCTAGCAGAAAGCAACGATGCACCCAGCAACAGCTATGAA
TCTTTTCCAGAAATTAGAGAAAATCTAACCGCAAGTGAGGAATCCCTAACATCATGTGAG
GAATCCCTAACAGGAAGTAATGAATCCCTAACAGGAAGTAATGAATCCCTAACAGGAAGT
AAT---------------------------------------------------------
------GAATCCCTAACAGGAAGTAATGAATCCCTAACAGGAAGTAATGAATCCCTAACA
GAAAGTAGAGAATCTCTAGAGGCCAGTAGAGAATCGCTAAGAGCAAGTAGAGAGTCTCTA
GCGGCCAGTAGAGAATCCCTGAACGACTTTTGTGGGAGCGAAGAATCAGTAGCATGCGAA
GGAGAGCCAAATGAAAAGACATTCATGGGAGACGTCTTAAGTGGTGGAGAATGTGAGAAT
AGTCTCTCAAGAGAAGATTTATTTCATATAGAAGTAGGATCCGAAGAATCGCTAGATGAT
GCCTCAAAATATAATTTCCAAAAGGATTTATCAACTAGCGATAATAGTTCATTCGAAGAT
GATCAGTCATTGAAAAGGGGACTCAAAAGGAACAGCTCAGTATCTAGTCTGGACAGCGAT
ATGGGAAGTTATAAAAATGGAAGATCTAGAGACAGATTGGATATATATTCGGACCTACCT
AGAAGGCTAATACATGGGGATAATGCACCACAAGAAAAGGAGAAATGTCTGGATTTCAAA
GAACTTCTAGAGAGAGAAAGGAACAACCACCAAAGGAATAGTGTAAATATAAAATTAAAA
AATATATACGGAGAAGAACAATTGTATAATGTGAAGAAAGATAGCTTTATTGTAAAATTG
ATATACTATCTGCCGTTTGGCCCAATTGTTACAATGATAATGATAGGCTTTATTCTACTC
TGCTCAACGCCCTCGACTGGTTTTATTTTTATAGGGTGTAGCTCTATTATATTACCAGCT
TTGTTATACGCGATGCATTCAAACAAAAAACAATTCGAACGTGTATTTGGAAAAGGGAGA
AAAAAGCCAATGAAGGTAAAAAAAGGAGGCGGGAAAATATCTAGATTTTTTAAAGAAGTA
GACAAATTCTTGTTTGACGATTTGGATGGTTAT
>Peru858 .
ATGAAAGGAATAATGAATGGTTCCCTTTGTTTAAGGAAAATTTCCCTGTGTTCTATTTTC
GTATTGGCATATCTCAGTTCTGCTAAGTGCGCGTACCGAGACGAATCTGACATATTGTAC
AGCGACGACTCCAGATCCGATTTAGCAAGCAACGGGGCACCCACCGACAGCTACGAATCT
TTAACAGCAAGTAGTGAGTCTCTAGCAGAAAGCAACGATGCACCCAGCAACAGCTATGAA
TCTTTTCCAGAAATTAGAGAAAATCTAACCGCAAGTGAGGAATCCCTAACATCATGTGAG
GAATCCCTAACAGGAAGTAATGAATCCCTAACAGGAAGTAATGAATCCCTAACAGGAAGT
AAT---------------------------------------------------------
------GAATCCCTAACAGGAAGTAATGAATCCCTAACAGGAAGTAATGAATCCCTAACA
GAAAGTAGAGAATCTCTAGAGGCCAGTAGAGAATCGCTAAGAGCAAGTAGAGAGTCTCTA
GCGGCCAGTAGAGAATCCCTGAACGACTTTTGTGGGAGCGAAGAATCAGTAGCATTCGAA
GGAGAGCCAAATGAAAAGACATTCATGGGAGACGTCTTAAGTGGTGGAGAATGTGAGAAT
AGTCTCTCAAGAGAAGATTTATTTCATATAGAAGTAGGATCCGAAGAATCGCTAGATGAT
GCCTCAAAATATAATTTCCAAAAGGATTTATCAACTAGCGATAATAGTTCATTCGAAGAT
GATCAGTCATTGAAAAGGGGACTCAAAAGGAACAGCTCAGTATCTAGTCTGGACAGCGAT
ATGGGAAGTTATAAAAATGGAAGATCTAGAGACAGATTGGATATATATTCGGACCTACCT
AGAAGGCCAATACATGGGGATAATGCACCACAAGAAAAGGAGAAATGTCTGGATTTCAAA
GAACTTCTAGAGAGAGAAAGGAACAACCACCAAAGGAATAGTGTAAATATAAAATTAAAA
AATATATACGGAGAAGAACAATTGTATAATGTGAAGAAAGATAGCTTTATTGTAAAATTG
ATATACTATCTGCCGTTTGGCCCAATTGTTACAATGATAATGATAGGCTTTATTCTACTC

TGCTCAACGCCCTCGACTGGTTTTATTTTTATAGGGTGTAGCTCTATTATATTACCAGCT
TTGTTATACGCGATGCATTCAAACAAAAAACAATTCGAACGTGTATTTGGAAAAGGGAGA
AAAAAGCCAATGAAGGTAAAAAAAGGAGGCGGGAAAATATCTAGATTTTTTAAAGAAGTA
GACAAATTCTTGTTTGACGATTTGGATGGTTAT
>Peru259 .
ATGAAAGGAATAATGAATGGTTCCCTTTGTTTAAGGAAAATTTCCCTGTGTTCTATTTTC
GTATTGGCATATCTCAGTTCTGCTAAGTGCGCGTACCGAGACGAATCTGACATATTGTAC
AGCGACGACTCCAGATCCGATTTAGCAAGCAACGGGGCACCCACCGACAGCTACGAATCT
TTAACAGCAAGTAGTGAGTCTCTAGCAGAAAGCAACGATGCACCCAGCAACAGCTATGAA
TCTTTTCCAGAAATTAGAGAAAATCTAACCGCAAGTGAGGAATCCCTAACATCATGTGAG
GAATCCCTAACAGGAAGTAATGAATCCCTAACAGGAAGTAATGAATCCCTAACAGGAAGT
AAT---------------------------------------------------------
------GAATCCCTAACAGGAAGTAATGAATCCCTAACAGGAAGTAATGAATCCCTAACA
GAAAGTAGAGAATCTCTAGAGGCCAGTAGAGAATCGCTAAGAGCAAGTAGAGAGTCTCTA
GCGGCCAGTAGAGAATCCCTGAACGACTTTTGTGGGAGCGAAGAATCAGTAGCATGCGAA
GGAGAGCCAAATGAAAAGACATTCATGGGAGACGTCTTAAGTGGTGGAGAATGTGAGAAT
AGTCTCTCAAGAGAAGATTTATTTCATATAGAAGTAGGATCCGAAGAATCGCTAGATGAT
GCCTCAAAATATAATTTCCAAAAGGATTTATCAACTAGCGATAATAGTTCATTCGAAGAT
GATCAGTCATTGAAAAGGGGACTCAAAAGGAACAGCTCAGTATCTAGTCTGGACAGCGAT
ATGGGAAGTTATAAAAATGGAAGATCTAGAGACAGATTGGATATATATTCGGACCTACCT
AGAAGGCTAATACATGGGGATAATGCACCACAAGAAAAGGAGAAATGTCTGGATTTCAAA
GAACTTCTAGAGAGAGAAAGGAACAACCACCAAAGGAATAGTGTAAATATAAAATTAAAA
AATATATACGGAGAAGAACAATTGTATAATGTGAAGAAAGATAGCTTTATTGTAAAATTG
ATATACTATCTGCCGTTTGGCCCAATTGTTACAATGATAATGATAGGCTTTATTCTACTC
TGCTCAACGCCCTCGACTGGTTTTATTTTTATAGGGTGTAGCTCTATTATATTACCAGCT
TTGTTATACGCGATGCATTCAAACAAAAAACAATTCGAACGTGTATTTGGAAAAGGGAGA
AAAAAGCCAATGAAGGTAAAAAAAGGAGGCGGGAAAATATCTAGATTTTTTAAAGAAGTA
GACAAATTCTTGTTTGACGATTTGGATGGTTAT
>Peru260 .
ATGAAAGGAATAATGAATGGTTCCCTTTATTTAAGGAAAATTTCCCTGTGTTCTATTTTC
GTATTGGCATATCTCAGTTCTGCTAAGTGCGCGTACCGAGACGAATCTGACATATTGTAC
AGCGACGACTCCAGATCCGATTTAGCAAGCAACGGGGCACCCACCGACAGCTACGAATCT
TTAACAGCAAGTAGTGAGTCTCTAGCAGAAAGCAACGATGCACCCAGCAACAGCTATGAA
TCTTTTCCAGAAATTAGAGAAAATCTAACCGCAAGTGAGGAATCCCTAACATCATGTGAG
GAATCCCTAACAGGAAGTAATGAATCCCTAACAGGAAGTAATGAATCCCTAACAGGAAGT
AAT---------------------------------------------------------
------GAATCCCTAACAGGAAGTAATGAATCCCTAACAGGAAGTAATGAATCCCTAACA
GAAAGTAGAGAATCTCTAGAGGCCAGTAGAGAATCGCTAAGAGCAAGTAGAGAGTCTCTA
GCGGCCAGTAGAGAATCCCTGAACGACTTTTGTGGGAGCGAAGAATCAGTAGCATTCGAA
GGAGAGCCAAATGAAAAGACATTCATGGGAGACGTCTTAAGTGGTGGAGAATGTGAGAAT
AGTCTCTCAAGAGAAGATTTATTTCATATAGAAGTAGGATCCGAAGAATCGCTAGATGAT
GCCTCAAAATATAATTTCCAAAAGGATTTATCAACTAGCGATAATAGTTCATTCGAAGAT
GATCAGTCATTGAAAAGGGGACTCAAAAGGAACAGCTCAGTATCTAGTCTGGACAGCGAT
ATGGGAAGTTATAAAAATAAAAGTTATAGACACAGATTGGATATATATTCGGACCTACCT
AGAAGGCCAATACATGGGGATAATGCACCACAAGAAAAGGAGAAATGTCTGGATTTCAAA
GAACTTCTAGAGAGAGAAAGGAACAACCACCAAAGGAATAGTGTAAATATAAAATTAAAA
AATATATACGGAGAAGAACAATTGTATAATGTGAAGAAAGATAGCTTTATTGTAAAATTG
ATATACTATCTGCCGTTTGGCCCAATTGTTACAATGATAATTATAGGCTTTATTCTACTC
TGGTCAACGACTTGGACTGGTTTTATTTTTATAGGGTGTAGCTCTATTATATTACCAGCT
TTGTTATACGCGATGCATTCAAACAAAAAACAATTCGAACGTGTATTTGGAAAAGGGAGA

AAAAAGCCAATGAAGGTAAAAAAAGGAGGCGGGAAAATATCTAGATTTTTTAAAGAAGTA
GACAAATTCTTGTTTGACGATTTGGATGGTTAT
>Peru262 .
ATGAAAGGAATAATGAATGGTTCCCTTTGTTTAAGGAAAATTTCCCTGTGTTCTATTTTC
GTATTGGCATATCTCAGTTCTGCTAAGTGCGCGTACCGAGACGAATCTGACATATTGTAC
AGCGACGACTCCAGATCCGATTTAGCAAGCAACGGGGCACCCACCGACAGCTACGAATCT
TTAACAGCAAGTAGTGAGTCTCTAGCAGAAAGCAACGATGCACCCAGCAACAGCTATGAA
TCTTTTCCAGAAATTAGAGAAAATCTAACCGCAAGTGAGGAATCCCTAACATCATGTGAG
GAATCCCTAACAGGAAGTAATGAATCCCTAACAGGAAGTAATGAATCCCTAACAGGAAGT
AAT---------------------------------------------------------
------GAATCCCTAACAGGAAGTAATGAATCCCTAACAGGAAGTAATGAATCCCTAACA
GAAAGTAGAGAATCTCTAGAGGCCAGTAGAGAATCGCTAAGAGCAAGTAGAGAGTCTCTA
GCGGCCAGTAGAGAATCCCTGAACGACTTTTGTGGGAGCGAAGAATCAGTAGCATGCGAA
GGAGAGCCAAATGAAAAGACATTCATGGGAGACGTCTTAAGTGGTGGAGAATGTGAGAAT
AGTCTCTCAAGAGAAGATTTATTTCATATAGAAGTAGGATCCGAAGAATCGCTAGATGAT
GCCTCAAAATATAATTTCCAAAAGGATTTATCAACTAGCGATAATAGTTCATTCGAAGAT
GATCAGTCATTGAAAAGGGGACTCAAAAGGAACAGCTCAGTATCTAGTCTGGACAGCGAT
ATGGGAAGTTATAAAAATGGAAGATCTAGAGACAGATTGGATATATATTCGGACCTACCT
AGAAGGCTAATACATGGGGATAATGCACCACAAGAAAAGGAGAAATGTCTGGATTTCAAA
GAACTTCTAGAGAGAGAAAGGAACAACCACCAAAGGAATAGTGTAAATATAAAATTAAAA
AATATATACGGAGAAGAACAATTGTATAATGTGAAGAAAGATAGCTTTATTGTAAAATTG
ATATACTATCTGCCGTTTGGCCCAATTGTTACAATGATAATGATAGGCTTTATTCTACTC
TGCTCAACGCCCTCGACTGGTTTTATTTTTATAGGGTGTAGCTCTATTATATTACCAGCT
TTGTTATACGCGATGCATTCAAACAAAAAACAATTCGAACGTGTATTTGGAAAAGGGAGA
AAAAAGCCAATGAAGGTAAAAAAAGGAGGCGGGAAAATATCTAGATTTTTTAAAGAAGTA
GACAAATTCTTGTTTGACGATTTGGATGGTTAT
>Peru2025 .
ATGAAAGGAATAATGAATGGTTCCCTTTGTTTAAGGAAAATTTCCCTGTGTTCTATTTTC
GTATTGGCATATCTCAGTTCTGCTAAGTGCGCGTACCGAGACGAATCTGACATATTGTAC
AGCGACGACTCCAGATCCGATTTAGCAAGCAACGGGGCACCCACCGACAGCTACGAATCT
TTAACAGCAAGTAGTGAGTCTCTAGCAGAAAGCAACGATGCACCCAGCAACAGCTATGAA
TCTTTTCCAGAAATTAGAGAAAATCTAACCGCAAGTGAGGAATCCCTAACATCATGTGAG
GAATCCCTAACAGGAAGTAATGAATCCCTAACAGGAAGTAATGAATCCCTAACAGGAAGT
AAT---------------------------------------------------------
------GAATCCCTAACAGGAAGTAATGAATCCCTAACAGGAAGTAATGAATCCCTAACA
GAAAGTAGAGAATCTCTAGAGGCCAGTAGAGAATCGCTAAGAGCAAGTAGAGAGTCTCTA
GCGGCCAGTAGAGAATCCCTGAACGACTTTTGTGGGAGCGAAGAATCAGTAGCATTCGAA
GGAGAGCCAAATGAAAAGACATTCATGGGAGACGTCTTAAGTGGTGGAGAATGTGAGAAT
AGTCTCTCAAGAGAAGATTTATTTCATATAGAAGTAGGATCCGAAGAATCGCTAGATGAT
GCCTCAAAATATAATTTCCAAAAGGATTTATCAACTAGCGATAATAGTTCATTCGAAGAT
GATCAGTCATTGAAAAGGGGACTCAAAAGGAACAGCTCAGTATCTAGTCTGGACAGCGAT
ATGGGAAGTTATAAAAATAAAAGTTATAGACACAGATTGGATATATATTCGGACCTACCT
AGAAGGCCAATACATGGGGATAATGCACCACAAGAAAAGGAGAAATGTCTGGATTTCAAA
GAACTTCTAGAGAGAGAAAGGAACAACCACCAAAGGAATAGTGTAAATATAAAATTAAAA
AATATATACGGAGAAGAACAATTGTATAATGTGAAGAAAGATAGCTTTATTGTAAAATTG
ATATACTATCTGCCGTTTGGCCCAATTGTTACAATGATAATGATAGGCTTTATTCTACTC

TGCTCAACGCCCTCGACTGGTTTTATTTTTATAGGGTGTAGCTCTATTATATTACCAGCT
TTGTTATACGCGATGCATTCAAACAAAAAACAATTCGAACGTGTATTTGGAAAAGGGAGA
AAAAAGCCAATGAAGGTAAAAAAAGGAGGCGGGAAAATATCTAGATTTTTTAAAGAAGTA
GACAAATTCTTGTTTGACGATTTGGATGGTTAT
>Peru3133 .
ATGAAAGGAATAATGAATGGTTCCCTTTGTTTAAGGAAAATTTCCCTGTGTTCTATTTTC
GTATTGGCATATCTCAGTTCTGCTAAGTGCGCGTACCGAGACGAATCTGACATATTGTAC
AGCGACGACTCCAGATCCGATTTAGCAAGCAACGGGGCACCCACCGACAGCTACGAATCT
TTAACAGCAAGTAGTGAGTCTCTAGCAGAAAGCAACGATGCACCCAGCAACAGCTATGAA
TCTTTTCCAGAAATTAGAGAAAATCTAACCGCAAGTGAGGAATCCCTAACATCATGTGAG
GAATCCCTAACAGGAAGTAATGAATCCCTAACAGGAAGTAATGAATCCCTAACAGAAAGT
AAT---------------------------------------------------------
------GAATCCCTAACAGGAAGTAATGAATCCCTAACAGGAAGTAATGAATCCCTAACA
GAAAGTAGAGAATCTCTAGAGGCCAGTAGAGAATCGCTAAGAGCAAGTAGAGAGTCTCTA
GCGGCCAGTAGAGAATCCCTGAACGACTTTTGTGGGAGCGAAGAATCAGTAGCATTCGAA
GGAGAGCCAAATGAAAAGACATTCATGGGAGACGTCTTAAGTGGTGGAGAATGTGAGAAT
AGTCTCTCAAGAGAAGATTTATTTCATATAGAAGTAGGATCCGAAGAATCGCTAGATGAT
GCCTCAAAATATAATTTCCAAAAGGATTTATCAACTAGCGATAATAGTTTATTCGAAGAT
GATCAGTCATTGAAAAGGGGACTCAAAAGGAACAGCTCAGTATCTAGTCTGGACAGCGAT
ATGGGAAGTTATAAAAATGGAAGATCTAGAGACAGATTGGATATATATTCGGACCTACCT
AGAAGGCTAATACATGGGGATAATGCACCACAAGAAAAGGAGAAATGTCTGGATTTCAAA
GAACTTCTAGAGAGAGAAAGGAACAACCACCAAAGGAATAGTGTAAATATAAAATTAAAA
AATATATACGGAGAAGAACAATTGTATAATGTGAAGAAAGATAGCTTTATTGTAAAATTG
ATATACTATCTGCCGTTTGGCCCAATTGTTACAATGATAATGATAGGTTTTTTTCTACTC
TGCTGGACCCCCTCGATTGGTTTTATTTTTATAGGGTGTAGCTCTATTATATTACCAGCT
TTGTTATACGCGATGCATTCAAACAAAAAACAATTCGAACGTGTATTTGGAAAAGGGAGA
AAAAAGCCAATGAAGGTAAAAAAAGGAGGCGGGAAAATATCTAGATTTTTTAAAGAAGTA
GACAAATTCTTGTTTGACGATTTGGATGGTTAT
>Peru3136 .
ATGAAAGGAATAATGAATGGTTCCCTTTATTTAAGGAAAATTTCCCTGTGTTCTATTTTC
GTATTGGCATATCTCAGTTCTGCTAAGTGCGCGTACCGAGACGAATCTGACATATTGTAC
AGCGACGACTCCAGATCCGATTTAGCAAGCAACGGGGCACCCACCGACAGCTACGAATCT
TTAACAGCAAGTAGTGAGTCTCTAGCAGAAAGCAACGATGCACCCAGCAACAGCTATGAA
TCTTTTCCAGAAATTAGAGAAAATCTAACCGCAAGTGAGGAATCCCTAACATCATGTGAG
GAATCCCTAACAGGAAGTAATGAATCCCTAACAGGAAGTAATGAATCCCTAACAGGAAGT
AAT---------------------------------------------------------
------GAATCCCTAACAGGAAGTAATGAATCCCTAACAGGAAGTAATGAATCCCTAACA
GAAAGTAGAGAATCTCTAGAGGCCAGTAGAGAATCGCTAAGAGCAAGTAGAGAGTCTCTA
GCGGCCAGTAGAGAATCCCTGAACGACTTTTGTGGGAGCGAAGAATCAGTAGCATTCGAA
GGAGAGCCAAATGAAAAGACATTCATGGGAGACGTCTTAAGTGGTGGAGAATGTGAGAAT
AGTCTCTCAAGAGAAGATTTATTTCATATAGAAGTAGGATCCGAAGAATCGCTAGATGAT
GCCTCAAAATATAATTTCCAAAAGGATTTATCAACTAGCGATAATAGTTCATTCGAAGAT
GATCAGTCATTGAAAAGGGGACTCAAAAGGAACAGCTCAGTATCTAGTCTGGACAGCGAT
ATGGGAAGTTATAAAAATAAAAGTTATAGACACAGATTGGATATATATTCGGACCTACCT
AGAAGGCCAATACATGGGGATAATGCACCACAAGAAAAGGAGAAATGTCTGGATTTCAAA
GAACTTCTAGAGAGAGAAAGGAACAACCACCAAAGGAATAGTGTAAATATAAAATTAAAA
AATATATACGGAGAAGAACAATTGTATAATGTGAAGAAAGATAGCTTTATTGTAAAATTG
ATATACTATCTGCCGTTTGGCCCAATTGTTACAATGATAATGATAGGCTTTATTCTACTC
TGCTCAACGCCCTCGACTGGTTTTATTTTTATAGGGTGTAGCTCTATTATATTACCAGCT
TTGTTATACGCGATGCATTCAAACAAAAAACAATTCGAACGTGTATTTGGAAAAGGGAGA

AAAAAGCCAATGAAGGTAAAAAAAGGAGGCGGGAAAATATCTAGATTTTTTAAAGAAGTA
GACAAATTCTTGTTTGACGATTTGGATGGTTAT
>Peru3232 .
ATGAAAGGAATAATGAATGGTTCCCTTTGTTTAAGGAAAATTTCCCTGTGTTCTATTTTC
GTATTGGCATATCTCAGTTCTGCTAAGTGCGCGTACCGAGACGAATCTGACATATTGTAC
AGCGACGACTCCAGATCCGATTTAGCAAGCAACGGGGCACCCACCGACAGCTACGAATCT
TTAACAGCAAGTAGTGAGTCTCTAGCAGAAAGCAACGATGCACCCAGCAACAGCTATGAA
TCTTTTCCAGAAATTAGAGAAAATCTAACCGCAAGTGAGGAATCCCTAACATCATGTGAG
GAATCCCTAACAGGAAGTAATGAATCCCTAACAGGAAGTAATGAATCCCTAACAGGAAGT
AAT---------------------------------------------------------
------GAATCCCTAACAGGAAGTAATGAATCCCTAACAGGAAGTAATGAATCCCTAACA
GAAAGTAGAGAATCTCTAGAGGCCAGTAGAGAATCGCTAAGAGCAAGTAGAGAGTCTCTA
GCGGCCAGTAGAGAATCCCTGAACGACTTTTGTGGGAGCGAAGAATCAGTAGCATTCGAA
GGAGAGCCAAATGAAAAGACATTCATGGGAGACGTCTTAAGTGGTGGAGAATGTGAGAAT
AGTCTCTCAAGAGAAGATTTATTTCATATAGAAGTAGGATCCGAAGAATCGCTAGATGAT
GCCTCAAAATATAATTTCCAAAAGGATTTATCAACTAGCGATAATAGTTCATTCGAAGAT
GATCAGTCATTGAAAAGGGGACTCAAAAGGAACAGCTCAGTATCTAGTCTGGACAGCGAT
ATGGGAAGTTATAAAAATAAAAGTTATAGACACAGATTGGATATATATTCGGACCTACCT
AGAAGGCCAATACATGGGGATAATGCACCACAAGAAAAGGAGAAATGTCTGGATTTCAAA
GAACTTCTAGAGAGAGAAAGGAACAACCACCAAAGGAATAGTGTAAATATAAAATTAAAA
AATATATACGGAGAAGAACAATTGTATAATGTGAAGAAAGATAGCTTTATTGTAAAATTG
ATATACTATCTGCCGTTTGGCCCAATTGTTACAATGATAATGATAGGCTTTATTCTACTC
TGGTCAACGCCCTGGACTGGTTTTATTTTTATAGGGTGTAGCTCTATTATATTACCAGCT
TTGTTATACGCGATGCATTCAAACAAAAAACAATTCGAACGTGTATTTGGAAAAGGGAGA
AAAAAGCCAATGAAGGTAAAAAAAGGAGGCGGGAAAATATCTAGATTTTTTAAAGAAGTA
GACAAATTCTTGTTTGACGATTTGGATGGTTAT
>Peru4023 .
ATGAAAGGAATAATGAATGGTTCCCTTTGTTTAAGGAAAATTTCCCTGTGTTCTATTTTC
GTATTGGCATATCTCAGTTCTGCTAAGTGCGCGTACCGAGACGAATCTGACATATTGTAC
AGCGACGACTCCAGATCCGATTTAGCAAGCAACGGGGCACCCACCGACAGCTACGAATCT
TTAACAGCAAGTAGTGAGTCTCTAGCAGAAAGCAACGATGCACCCAGCAACAGCTATGAA
TCTTTTCCAGAAATTAGAGAAAATCTAACCGCAAGTGAGGAATCCCTAACATCATGTGAG
GAATCCCTAACAGGAAGTAATGAATCCCTAACAGGAAGTAATGAATCCCTAACAGGAAGT
AAT---------------------------------------------------------
------GAATCCCTAACAGGAAGTAATGAATCCCTAACAGGAAGTAATGAATCCCTAACA
GAAAGTAGAGAATCTCTAGAGGCCAGTAGAGAATCGCTAAGAGCAAGTAGAGAGTCTCTA
GCGGCCAGTAGAGAATCCCTGAACGACTTTTGTGGGAGCGAAGAATCAGTAGCATTCGAA
GGAGAGCCAAATGAAAAGACATTCATGGGAGACGTCTTAAGTGGTGGAGAATGTGAGAAT
AGTCTCTCAAGAGAAGATTTATTTCATATAGAAGTAGGATCCGAAGAATCGCTAGATGAT
GCCTCAAAATATAATTTCCAAAAGGATTTATCAACTAGCGATAATAGTTCATTCGAAGAT
GATCAGTCATTGAAAAGGGGACTCAAAAGGAACAGCTCAGTATCTAGTCTGGACAGCGAT
ATGGGAAGTTATAAAAATAAAAGTTATAGACACAGATTGGATATATATTCGGACCTACCT
AGAAGGCCAATACATGGGGATAATGCACCACAAGAAAAGGAGAAATGTCTGGATTTCAAA
GAACTTCTAGAGAGAGAAAGGAACAACCACCAAAGGAATAGTGTAAATATAAAATTAAAA
AATATATACGGAGAAGAACAATTGTATAATGTGAAGAAAGATAGCTTTATTGTAAAATTG
ATATACTATCTGCCGTTTGGCCCAATTGTTACAATGATAATGATAGGCTTTATTCTACTC

TGGTCAACGGGCTGGACTGGTTTTATTTTTATAGGGTGTAGCTCTATTATATTACCAGCT
TTGTTATACGCGATGTATTCAAACAAAAAACAATTCGAACGTGTATTTGGAAAAGGGAGA
AAAAAGCCAATGAAGGTAAAAAAAGGAGGCGGGAAAATATCTAGATTTTTTAAAGAAGTA
GACAAATTCTTGTTTGACGATTTGGATGGTTAT
>Thailand_VKTS37 .
ATGAAAGGAATAATGAATGGTTCCCTTTGTTTAAGGAAAATTTCCCTGTGTTCTATTTTC
GTATTGGCATATCTCAGTTCTGCTAAGTGCGCGTACCGAGACGAATCTGACATATTGTAC
AGCGACGACTCCAGATCCGATTTAGCAAGCAACGGGGCACCCACCGACAGCTACGAATCT
TTAACAGCAAGTAGTGAGTCTCTAGCAGAAAGCAACGATGCACCCAGCAACAGCTATGAA
TCTTTTCCAGAAATTAGAGAAAATCTAACCGCAAGTGAGGAATCCCTAACATCATGTGAG
GAATCCCTAACAGGAAGTAATGAATCCCTAACAGGAAGTAATGAATCCCTAACAGGAAGT
AAT---------------------------------------------------------
------GAATCCCTAACAGGAAGTAATGAATCCCTAACAGAAAGTAGAGAATCCCTAACA
GAAAGTAGAGAATCTCTAGAGGCCAGTAGAGAATCGCTAAGAGCAAGTAGAGAGTCTCTA
GCGGCCAGTAGAGAATCCCTGAACGACTTTTGTGGGAGCGAAGAATCAGTAGCATTCGAA
GGAGAGCCAAATGAAAAGACATTCATGGGAGACGTCTTAAGTGGTGGAGAATGTGAGAAT
AGTCTCTCAAGAGAAGATTTATTTCATATAGAAGTAGGATCCGAAGAATCGCTAGATGAT
GCCTCAAAATATAATTTCCAAAAGGATTTATCAACTAGCGATAATAGTTCATTCGAAGAT
GATCAGTCATTGAAAAGGGGACTCAAAAGGAACAGCTCAGTATCTAGTCTGGACAGCGAT
ATGGGAAGTTATAAAAATAAAAGTTATAGACACAGATTGGATATATATTCGGACCTACCT
AGAAGGCTAATACATGGGGATAATGCACCACAAGAAAAGGAGAAATGTCTGGATTTCAAA
GAACTTCTAGAGAGAGAAAGGAACAACCACCAAAGGAATAGTGTAAATATAAAATTAAAA
AATATATACGGAGAAGAACAATTGTATAATGTGAAGAAAGATAGCTTTATTGTAAAATTG
ATATACTATCTGCCGTTTGGCCCAATTGTTACAATGATAATGATAGGCTTTATTCTACTC
TGGTCAACGGCCTGGACTGGTTTTATTTTTATAGGGTGTAGCTCTATTATATTACCAGCT
TTGTTATACGCGATGCATTCAAACAAAAAACAATTCGAACGTGTATTTGGAAAAGGGAGA
AAAAAGCCAATGAAGGTAAAAAAAGGAGGCGGGAAAATATCTAGATTTTTTAAAGAAGTA
GACAAATTCTTGTTTGACGATTTGGATGGTTAT
>Thailand_VKTS52 .
ATGAAAGGAATAATGAATGGTTCCCTTTGTTTAAGGAAAATTTCCCTGTGTTCTATTTTC
GTATTGGCATATCTCAGTTCTGCTAAGTGCGCGTACCGAGACGAATCTGACATATTGTAC
AGCGACGACTCCAGATCCGATTTAGCAAGCAACGGGGCACCCACCGACAGCTACGAATCT
TTAACAGCAAGTAGTGAGTCTCTAGCAGAAAGCAACGATGCACCCAGCAACAGCTATGAA
TCTTTTCCAGAAATTAGAGAAAATCTAACCGCAAGTGAGGAATCCCTAACATCATGTGAG
GAATCCCTAACAGGAAGTAATGAATCCCTAACAGGAAGTAATGAATCCCTAACAGGAAGT
AAT---------------------------------------------------------
------GAATCCCTAACAGGAAGTAATGAATCCCTAACAGGAAGTAATGAATCCCTAACA
GAAAGTAGAGAATCTCTAGAGGCCAGTAGAGAATCGCTAAGAGCAAGTAGAGAGTCTCTA
GCGGCCAGTAGAGAATCCCTGAACGACTTTTGTGGGAGCGAAGAATCAGTAGCATTCGAA
GGAGAGCCAAATGAAAAGACATTCATGGGAGACGTCTTAAGTGGTGGAGAATGTGAGAAT
AGTCTCTCAAGAGAAGATTTATTTCATATAGAAGTAGGATCCGAAGAATCGCTAGATGAT
GCCTCAAAATATAATTTCCAAAAGGATTTATCAACTAGCGATAATAGTTCATTCGAAGAT
GATCAGTCATTGAAAAGGGGACTCAAAAGGAACAGCTCAGTATCTAGTCTGGACAGCGAT
ATGGGAAGTTATAAAAATAAAAGTTATAGACACAGATTGGATATATATTCGGACCTACCT
AGAAGGCCAATACATGGGGATAATGCACCACAAGAAAAGGAGAAATGTCTGGATTTCAAA
GAACTTCTAGAGAGAGAAAGGAACAACCACCAAAGGAATAGTGTAAATATAAAATTAAAA
AATATATACGGAGAAGAACAATTGTATAATGTGAAGAAAGATAGCTTTATTGTAAAATTG
ATATACTATCTGCCGTTTGGCCCAATTGTTACAATGATAATGATAGGTTTTTTTCTACTC
TGCTGGACCCCCTCGATTGGTTTTACTTTTATAGGGTGTAGCTCTATTATATTACCAGCT
TTGTTATACGCGATGTATTCAAACAAAAAACAATTCGAACGTGTATTTGGAAAAGGGAGA

AAAAAGCCAATGAAGGTAAAAAAAGGAGGCGGGAAAATATCTAGATTTTTTAAAGAAGTA
GACAAATTCTTGTTTGACGATTTGGATGGTTAT
>Thailand_VKTS45 .
ATGAAAGGAATAATGAATGGTTCCCTTTGTTTAAGGAAAATTTCCCTGTGTTCTATTTTC
GTATTGGCATATCTCAGTTCTGCTAAGTGCGCGTACCGAGACGAATCTGACATATTGTAC
AGCGACGACTCCAGATCCGATTTAGCAAGCAACGGGGCACCCACCGACAGCTACGAATCT
TTAACAGCAAGTAGTGAGTCTCTAGCAGAAAGCAACGATGCACCCAGCAACAGCTATGAA
TCTTTTCCAGAAATTAGAGAAAATCTAACCGCAAGTGAGGAATCCCTAACATCATGTGAG
GAATCCCTAACAGGAAGTAATGAATCCCTAACAGGAAGTAATGAATCCCTAACAGGAAGT
AAT---------------------------------------------------------
------GAATCCCTAACAGGAAGTAATGAATCCCTAACAGGAAGTAATGAATCCCTAACA
GAAAGTAGAGAATCTCTAGAGGCCAGTAGAGAATCGCTAAGAGCAAGTAGAGAGTCTCTA
GCGGCCAGTAGAGAATCCCTGAACGACTTTTGTGGGAGCGAAGAATCAGTAGCATTCGAA
GGAGAGCCAAATGAAAAGACATTCATGGGAGACGTCTTAAGTGGTGGAGAATGTGAGAAT
AGTCTCTCAAGAGAAGATTTATTTCATATAGAAGTAGGATCCGAAGAATCGCTAGATGAT
GCCTCAAAATATAATTTCCAAAAGGATTTATCAACTAGCGATAATAGTTCATTCGAAGAT
GATCAGTCATTGAAAAGGGGACTCAAAAGGAACAGCTCAGTATCTAGTCTGGACAGCGAT
ATGGGAAGTTATAAAAATAAAAGTTATAGACACAGATTGGATATATATTCGGACCTACCT
AGAAGGCCAATACATGGGGATAATGCACCACAAGAAAAGGAGAAATGTCTGGATTTCAAA
GAACTTCTAGAGAGAGAAAGGAACAACCACCAAAGGAATAGTGTAAATATAAAATTAAAA
AATATATACGGAGAAGAACAATTGTATAATGTGAAGAAAGATAGCTTTATTGTAAAATTG
ATATACTATTTGCCGTTTGGCCCAATTGTTACAATGATAATTATAGGCTTTATTCTACTC
TGGTCAACGGGCTGGACTGGTTTTATTTTTATAGGGTGTAGCTCTATTATATTACCAGCT
TTGTTATACGCGATGTATTCAAACAAAAAACAATTCGAACGTGTATTTGGAAAAGGGAGA
AAAAAGCCAATGAAGGTAAAAAAAGGAGGCGGGAAAATATCTAGATTTTTTAAAGAAGTA
GACAAATTCTTGTTTGACGATTTGGATGGTTAT
>Thailand_VKBT72 .
ATGAAAGGAATAATGAATGGTTCCCTTTATTTAAGGAAAATTTCCCTGTGTTCTATTTTC
GTATTGGCATATCTCAGTTCTGCTAAGTGCGCGTACCGAGACGAATCTGACATATTGTAC
AGCGACGACTCCAGATCCGATTTAGCAAGCAACGGGGCACCCACCGACAGCTACGAATCT
TTAACAGCAAGTAGTGAGTCTCTAGCAGAAAGCAACGATGCACCCAGCAACAGCTATGAA
TCTTTTCCAGAAATTAGAGAAAATCTAACCGCAAGTGAGGAATCCCTAACATCATGTGAG
GAATCCCTAACAGGAAGTAATGAATCCCTAACAGGAAGTAATGAATCCCTAACAGGAAGT
AAT---------------------------------------------------------
------GAATCCCTAACAGGAAGTAATGAATCCCTAACAGGAAGTAATGAATCCCTAACA
GAAAGTAGAGAATCTCTAGAGGCCAGTAGAGAATCGCTAAGAGCAAGTAGAGAGTCTCTA
GCGGCCAGTAGAGAATCCCTGAACGACTTTTGTGGGAGCGAAGAATCAGTAGCATGCGAA
GGAGAGCCAAATGAAAAGACATTCATGGGAGACGTCTTAAGTGGTGGAGAATGTGAGAAT
AGTCTCTCAAGAGAAGATTTATTTCATATAGAAGTAGGATCCGAAGAATCGCTAGATGAT
GCCTCAAAATATAATTTCCAAAAGGATTTATCAACTAGCGATAATAGTTCATTCGAAGAT
GATCAGTCATTGAAAAGGGGACTCAAAAGGAACAGCTCAGTATCTAGTCTGGACAGCGAT
ATGGGAAGTTATAAAAATGGAAGATCTAGAGACAGATTGGATATATATTCGGACCTACCT
AGAAGGCTAATACATGGGGATAATGCACCACAAGAAAAGGAGAAATGTCTGGATTTCAAA
GAACTTCTAGAGAGAGAAAGGAACAACCACCAAAGGAATAGTGTAAATATAAAATTAAAA
AATATATACGGAGAAGAACAATTGTATAATGTGAAGAAAGATAGCTTTATTGTAAAATTG
ATATACTATCTGCCGTTTGGCCCAATTGTTACAATGATAATGATAGGCTTTATTCTACTC

TGGTCAACGGCTTGGACTGGTTTTATTTTTATAGGGTGTAGCTCTATTATATTACCAGCT
TTGTTATACGCGATGCATTCAAACAAAAAACAATTCGAACGTGTATTTGGAAAAGGGAGA
AAAAAGCCAATGAAGGTAAAAAAAGGAGGCGGGAAAATATCTAGATTTTTTAAAGAAGTA
GACAAATTCTTGTTTGACGATTTGGATGGTTAT
>Thailand_VKBT95 .
ATGAAAGGAATAATGAATGGTTCCCTTTGTTTAAGGAAAATTTCCCTGTGTTCTATTTTC
GTATTGGCATATCTCAGTTCTGCTAAGTGCGCGTACCGAGACGAATCTGACATATTGTAC
AGCGACGACTCCAGATCCGATTTAGCAAGCAACGGGGCACCCACCGACAGCTACGAATCT
TTAACAGCAAGTAGTGAGTCTCTAGCAGAAAGCAACGATGCACCCAGCAACAGCTATGAA
TCTTTTCCAGAAATTAGAGAAAATCTAACCGCAAGTGAGGAATCCCTAACATCATGTGAG
GAATCCCTAACAGGAAGTAATGAATCCCTAACAGGAAGTAATGAATCCCTAACAGGAAGT
AAT---------------------------------------------------------
------GAATCCCTAACAGGAAGTAATGAATCCCTAACAGGAAGTAATGAATCCCTAACA
GAAAGTAGAGAATCTCTAGAGGCCAGTAGAGAATCGCTAAGAGCAAGTAGAGAGTCTCTA
GCGGCCAGTAGAGAATCCCTGAACGACTTTTGTGGGAGCGAAGAATCAGTAGCATTCGAA
GGAGAGCCAAATGAAAAGACATTCATGGGAGACGTCTTAAGTGGTGGAGAATGTGAGAAT
AGTCTCTCAAGAGAAGATTTATTTCATATAGAAGTAGGATCCGAAGAATCGCTAGATGAT
GCCTCAAAATATAATTTCCAAAAGGATTTATCAACTAGCGATAATAGTTCATTCGAAGAT
GATCAGTCATTGAAAAGGGGACTCAAAAGGAACAGCTCAGTATCTAGTCTGGACAGCGAT
ATGGGAAGTTATAAAAATAAAAGTTATAGACACAGATTGGATATATATTCGGACCTACCT
AGAAGGCCAATACATGGGGATAATGCACCACAAGAAAAGGAGAAATGTCTGGATTTCAAA
GAACTTCTAGAGAGAGAAAGGAACAACCACCAAAGGAATAGTGTAAATATAAAATTAAAA
AATATATACGGAGAAGAACAATTGTATAATGTGAAGAAAGATAGCTTTATTGTAAAATTG
ATATACTATTTGCCGTTTGGCCCAATTGTTACAATGATAATGATAGGCTTTATTCTACTC
TGGTCAACGCCCTGGACTGGTTTTATTTTTATAGGGTGTAGCTCTATTATATTACCAGCT
TTGTTATACGCGATGCATTCAAACAAAAAACAATTCGAACGTGTATTTGGAAAAGGGAGA
AAAAAGCCAATGAAGGTAAAAAAAGGAGGCGGGAAAATATCTAGATTTTTTAAAGAAGTA
GACAAATTCTTGTTTGACGATTTGGATGGTTAT
>Thailand_VKBT98 .
ATGAAAGGAATAATGAATGGTTCCCTTTGTTTAAGGAAAATTTCCCTGTGTTCTATTTTC
GTATTGGCATATCTCAGTTCTGCTAAGTGCGCGTACCGAGACGAATCTGACATATTGTAC
AGCGACGACTCCAGATCCGATTTAGCAAGCAACGGGGCACCCACCGACAGCTACGAATCT
TTAACAGCAAGTAGTGAGTCTCTAGCAGAAAGCAACGATGCACCCAGCAACAGCTATGAA
TCTTTTCCAGAAATTAGAGAAAATCTAACCGCAAGTGAGGAATCCCTAACATCATGTGAG
GAATCCCTAACAGGAAGTAATGAATCCCTAACAGGAAGTAATGAATCCCTAACAGGAAGT
AAT---------------------------------------------------------
------GAATCCCTAACAGGAAGTAATGAATCCCTAACAGGAAGTAATGAATCCCTAACA
GAAAGTAGAGAATCTCTAGAGGCCAGTAGAGAATCGCTAAGAGCAAGTAGAGAGTCTCTA
GCGGCCAGTAGAGAATCCCTGAACGACTTTTGTGGGAGCGAAGAATCAGTAGCATTCGAA
GGAGAGCCAAATGAAAAGACATTCATGGGAGACGTCTTAAGTGGTGGAGAATGTGAGAAT
AGTCTCTCAAGAGAAGATTTATTTCATATAGAAGTAGGATCCGAAGAATCGCTAGATGAT
GCCTCAAAATATAATTTCCAAAAGGATTTATCAACTAGCGATAATAGTTCATTCGAAGAT
GATCAGTCATTGAAAAGGGGACTCAAAAGGAACAGCTCAGTATCTAGTCTGGACAGCGAT
ATGGGAAGTTATAAAAATAAAAGTTATAGACACAGATTGGATATATATTCGGACCTACCT
AGAAGGCCAATACATGGGGATAATGCACCACAAGAAAAGGAGAAATGTCTGGATTTCAAA
GAACTTCTAGAGAGAGAAAGGAACAACCACCAAAGGAATAGTGTAAATATAAAATTAAAA
AATATATACGGAGAAGAACAATTGTATAATGTGAAGAAAGATAGCTTTATTGTAAAATTG
ATATACTATTTGCCGTTTGGCCCAATTGTTACAATGATAATTATAGGCTTTATTCTACTC
TGGTCAACGGGCTGGACTGGTTTTATTTTTATAGGGTGTAGCTCTATTATATTACCAGCT
TTGTTATACGCGATGTATTCAAACAAAAAACAATTCGAACGTGTATTTGGAAAAGGGAGA

AAAAAGCCAATGAAGGTAAAAAAAGGAGGCGGGAAAATATCTAGATTTTTTAAAGAAGTA
GACAAATTCTTGTTTGACGATTTGGATGGTTAT
>Thailand_VKBT99 .
ATGAAAGGAATAATGAATGGTTCCCTTTGTTTAAGGAAAATTTCCCTGTGTTCTATTTTC
GTATTGGCATATCTCAGTTCTGCTAAGTGCGCGTACCGAGACGAATCTGACATATTGTAC
AGCGACGACTCCAGATCCGATTTAGCAAGCAACGGGGCACCCACCGACAGCTACGAATCT
TTAACAGCAAGTAGTGAGTCTCTAGCAGAAAGCAACGATGCACCCAGCAACAGCTATGAA
TCTTTTCCAGAAATTAGAGAAAATCTAACCGCAAGTGAGGAATCCCTAACATCATGTGAG
GAATCCCTAACAGGAAGTAATGAATCCCTAACA---------------------------
------------------------------------GAAAGTAGAGAATCCCTAACAGGA
AGTAATGAATCCCTAACAGAAAGTAGAGAATCCCTAACAGAAAGTAGAGAATCCCTAACA
GAAAGTAGAGAATCTCTAGAGGCCAGTAGAGAATCGCTAAGAGCAAGTAGAGAGTCTCTA
GCGGCCAGTAGAGAATCCCTGAACGACTTTTGTGGGAGCGAAGAATCAGTAGCATGCGAA
GGAGAGCCAAATGAAAAGACATTCATGGGAGACGTCTTAAGTGGTGGAGAATGTGAGAAT
AGTCTCTCAAGAGAAGATTTATTTCATATAGAAGTAGGATCCGAAGAATCGCTAGATGAT
GCCTCAAAATATAATTTCCAAAAGGATTTATCAACTAGCGATAATAGTTCATTCGAAGAT
GATCAGTCATTGAAAAGGGGACTCAAAAGGAACAGCTCAGTATCTAGTCTGGACAGCGAT
ATGGGAAGTTATAAAAATAAAAGTTATAGACACAGATTGGATATATATTCGGACCTACCT
AGAAGGCCAATACATGGGGATAATGCACCACAAGAAAAGGAGAAATGTCTGGATTTCAAA
GAACTTCTAGAGAGAGAAAGGAACAACCACCAAAGGAATAGTGTAAATATAAAATTAAAA
AATATATACGGAGAAGAACAATTGTATAATGTGAAGAAAGATAGCTTTATTGTAAAATTG
ATATACTATCTGCCGTTTGGCCCAATTGTTACAATGATAATGATAGGCTTTATTCTACTC
TGCTCAACGCCCTCGACTGGTTTTATTTTTATAGGGTGTAGCTCTATTATATTACCAGCT
TTGTTATACGCGATGCATTCAAACAAAAAACAATTCGAACGTGTATTTGGAAAAGGGAGA
AAAAAGCCAATGAAGGTAAAAAAAGGAGGCGGGAAAATATCTAGATTTTTTAAAGAAGTA
GACAAATTCTTGTTTGACGATTTGGATGGTTAT
>Thailand_VKBT100 .
ATGAAAGGAATAATGAATGGTTCCCTTTGTTTAAGGAAAATTTCCCTGTGTTCTATTTTC
GTATTGGCATATCTCAGTTCTGCTAAGTGCGCGTACCGAGACGAATCTGACATATTGTAC
AGCGACGACTCCAGATCCGATTTAGCAAGCAACGGGGCACCCACCGACAGCTACGAATCT
TTAACAGCAAGTAGTGAGTCTCTAGCAGAAAGCAACGATGCACCCAGCAACAGCTATGAA
TCTTTTCCAGAAATTAGAGAAAATCTAACCGCAAGTGAGGAATCCCTAACATCATGTGAG
GAATCCCTAACAGGAAGTAATGAATCCCTAACAGGAAGTAATGAATCCCTAACAGGAAGT
AAT---------------------------------------------------------
------GAATCCCTAACAGGAAGTAATGAATCCCTAACAGGAAGTAATGAATCCCTAACA
GAAAGTAGAGAATCTCTAGAGGCCAGTAGAGAATCGCTAAGAGCAAGTAGAGAGTCTCTA
GCGGCCAGTAGAGAATCCCTGAACGACTTTTGTGGGAGCGAAGAATCAGTAGCATTCGAA
GGAGAGCCAAATGAAAAGACATTCATGGGAGACGTCTTAAGTGGTGGAGAATGTGAGAAT
AGTCTCTCAAGAGAAGATTTATTTCATATAGAAGTAGGATCCGAAGAATCGCTAGATGAT
GCCTCAAAATATAATTTCCAAAAGGATTTATCAACTAGCGATAATAGTTCATTCGAAGAT
GATCAGTCATTGAAAAGGGGACTCAAAAGGAACAGCTCAGTATCTAGTCTGGACAGCGAT
ATGGGAAGTTATAAAAATAAAAGTTATAGACACAGATTGGATATATATTCGGACCTACCT
AGAAGGCCAATACATGGGGATAATGCACCACAAGAAAAGGAGAAATGTCTGGATTTCAAA
GAACTTCTAGAGAGAGAAAGGAACAACCACCAAAGGAATAGTGTAAATATAAAATTAAAA
AATATATACGGAGAAGAACAATTGTATAATGTGAAGAAAGATAGCTTTATTGTAAAATTG
ATATACTATTTGCCGTTTGGCCCAATTGTTACAATGATAATGATAGGCTTTATTCTACTC

```
TGGTCAACGCCCTGGACTGGTTTTATTTTTATAGGGTGTAGCTCTATTATATTACCAGCT
TTGTTATACGCGATGCATTCAAACAAAAAACAATTCGAACGTGTATTTGGAAAAGGGAGA
AAAAAGCCAATGAAGGTAAAAAAAGGAGGCGGGAAAATATCTAGATTTTTTAAAGAAGTA
GACAAATTCTTGTTTGACGATTTGGATGGTTAT
>Belem .
ATGAAAGGAATAATGAATGGTTCCCTTTGTTTAAGGAAAATTTCCCTGTGTTCTATTTTC
GTATTGGCATATCTCAGTTCTGCTAAGTGCGCGTACCGAGACGAATCTGACATATTGTAC
AGCGACGACTCCAGATCCGATTTAGCAAGCAACGGGGCACCCACCGACAGCTACGAATCT
TTAACAGCAAGTAGTGAGTCTCTAGCAGAAAGCAACGATGCACCCAGCAACAGCTATGAA
TCTTTTCCAGAAATTAGAGAAAATCTAACCGCAAGTGAGGAATCCCTAACATCATGTGAG
GAATCCCTAACAGGAAGTAATGAATCCCTAACAGGAAGTAATGAATCCCTAACAGGAAGT
AAT---------------------------------------------------------
------GAATCCCTAACAGGAAGTAATGAATCCCTAACAGGAAGTAATGAATCCCTAACA
GAAAGTAGAGAATCTCTAGAGGCCAGTAGAGAATCGCTAAGAGCAAGTAGAGAGTCTCTA
GCGGCCAGTAGAGAATCCCTGAACGACTTTTGTGGGAGCGAAGAATCAGTAGCATTCGAA
GGAGAGCCAAATGAAAAGACATTCATGGGAGACGTCTTAAGTGGTGGAGAATGTGAGAAT
AGTCTCTCAAGAGAAGATTTATTTCATATAGAAGTAGGATCCGAAGAATCGCTAGATGAT
GCCTCAAAATATAATTTCCAAAAGGATTTATCAACTAGCGATAATAGTTCATTCGAAGAT
GATCAGTCATTGAAAAGGGGACTCAAAAGGAACAGCTCAGTATCTAGTCTGGACAGCGAT
ATGGGAAGTTATAAAAATAAAAGTTATAGACACAGATTGGATATATATTCGGACCTACCT
AGAAGGCCAATACATGGGGATAATGCACCACAAGAAAAGGAGAAATGTCTGGATTTCAAA
GAACTTCTAGAGAGAGAAAGGAACAACCACCAAAGGAATAGTGTAAATATAAAATTAAAA
AATATATACGGAGAAGAACAATTGTATAATGTGAAGAAAGATAGCTTTATTGTAAAATTG
ATATACTATCTGCCGTTTGGCCCAATTGTTACAATGATAATGATAGGTTTTTTTCTACTC
TGCTGGACCCCCTCGATTGGTTTTACTTTTATAGGGTGTAGCTCTATTATATTACCAGCT
TTGTTATACGCGATGCATTCAAACAAAAAACAATTCGAACGTGTATTTGGAAAAGGGAGA
AAAAAGCCAATGAAGGTAAAAAAAGGAGGCGGGAAAATATCTAGATTTTTTAAAGAAGTA
GACAAATTCTTGTTTGACGATTTGGATGGTTAT
>Brazil30 .
ATGAAAGGAATAATGAATGGTTCCCTTTATTTAAGGAAAATTTCCCTGTGTTCTATTTTC
GTATTGGCATATCTCAGTTCTGCTAAGTGCGCGTACCGAGACGAATCTGACATATTGTAC
AGCGACGACTCCAGATCCGATTTAGCAAGCAACGGGGCACCCACCGACAGCTACGAATCT
TTAACAGCAAGTAGTGAGTCTCTAGCAGAAAGCAACGATGCACCCAGCAACAGCTATGAA
TCTTTTCCAGAAATTAGAGAAAATCTAACCGCAAGTGAGGAATCCCTAACATCATGTGAG
GAATCCCTAACAGGAAGTAATGAATCCCTAACAGGAAGTAATGAATCCCTAACAGGAAGT
AAT---------------------------------------------------------
------GAATCCCTAACAGGAAGTAATGAATCCCTAACAGGAAGTAATGAATCCCTAACA
GAAAGTAGAGAATCTCTAGAGGCCAGTAGAGAATCGCTAAGAGCAAGTAGAGAGTCTCTA
GCGGCCAGTAGAGAATCCCTGAACGACTTTTGTGGGAGCGAAGAATCAGTAGCATTCGAA
GGAGAGCCAAATGAAAAGACATTCATGGGAGACGTCTTAAGTGGTGGAGAATGTGAGAAT
AGTCTCTCAAGAGAAGATTTATTTCATATAGAAGTAGGATCCGAAGAATCGCTAGATGAT
GCCTCAAAATATAATTTCCAAAAGGATTTATCAACTAGCGATAATAGTTCATTCGAAGAT
GATCAGTCATTGAAAAGGGGACTCAAAAGGAACAGCTCAGTATCTAGTCTGGACAGCGAT
ATGGGAAGTTATAAAAATAAAAGTTATAGACACAGATTGGATATATATTCGGACCTACCT
AGAAGGCCAATACATGGGGATAATGCACCACAAGAAAAGGAGAAATGTCTGGATTTCAAA
GAACTTCTAGAGAGAGAAAGGAACAACCACCAAAGGAATAGTGTAAATATAAAATTAAAA
AATATATACGGAGAAGAACAATTGTATAATGTGAAGAAAGATAGCTTTATTGTAAAATTG
ATATACTATCTGCCGTTTGGCCCAATTGTTACAATGATAATGATAGGCTTTATTCTACTC
TGGTCAACGCCCTGGACTGGTTTTATTTTTATAGGGTGTAGCTCTATTATATTACCAGCT
TTGTTATACGCGATGCATTCAAACAAAAAACAATTCGAACGTGTATTTGGAAAAGGGAGA
```

AAAAAGCCAATGAAGGTAAAAAAAGGAGGCGGGAAAATATCTAGATTTTTTAAAGAAGTA
GACAAATTCTTGTTTGACGATTTGGATGGTTAT
>China_NB17 .
ATGAAAGGAATAATGAATGGTTCCCTTTGTTTAAGGAAAATTTCCCTGTGTTCTATTTTC
GTATTGGCATATCTCAGTTCTGCTAAGTGCGCGTACCGAGACGAATCTGACATATTGTAC
AGCGACGACTCCAGATCCGATTTAGCAAGCAACGGGGCACCCACCGACAGCTACGAATCT
TTAACAGCAAGTAGTGAGTCTCTAGCAGAAAGCAACGATGCACCCAGCAACAGCTATGAA
TCTTTTCCAGAAATTAGAGAAAATCTAACCGCAAGTGAGGAATCCCTAACATCATGTGAG
GAATCCCTAACAGGAAGTAATGAATCCCTAACAGGAAGTAATGAATCCCTAACAGGAAGT
AAT---------------------------------------------------------
------GAATCCCTAACAGGAAGTAATGAATCCCTAACAGGAAGTAATGAATCCCTAACA
GAAAGTAGAGAATCTCTAGAGGCCAGTAGAGAATCGCTAAGAGCAAGTAGAGAGTCTCTA
GCGGCCAGTAGAGAATCCCTGAACGACTTTTGTGGGAGCGAAGAATCAGTAGCATTCGAA
GGAGAGCCAAATGAAAAGACATTCATGGGAGACGTCTTAAGTGGTGGAGAATGTGAGAAT
AGTCTCTCAAGAGAAGATTTATTTCATATAGAAGTAGGATCCGAAGAATCGCTAGATGAT
GCCTCAAAATATAATTTCCAAAAGGATTTATCAACTAGCGATAATAGTTCATTCGAAGAT
GATCAGTCATTGAAAAGGGGACTCAAAAGGAACAGCTCAGTATCTAGTCTGGACAGCGAT
ATGGGAAGTTATAAAAATAAAAGTTATAGACACAGATTGGATATATATTCGGACCTACCT
AGAAGGCCAATACATGGGGATAATGCACCACAAGAAAAGGAGAAATGTCTGGATTTCAAA
GAACTTCTAGAGAGAGAAAGGAACAACCACCAAAGGAATAGTGTAAATATAAAATTAAAA
AATATATACGGAGAAGAACAATTGTATAATGTGAAGAAAGATAGCTTTATTGTAAAATTG
ATATACTATCTGCCGTTTGGCCCAATTGTTACAATGATAATGATAGGCTTTATTCTACTC
TGGTCAACGCCCTCGATTGGTTTTATTTTTATAGGGTGTAGCTCTATTATATTACCAGCT
TTGTTATACGCGATGCATTCAAACAAAAAACAATTCGAACGTGTATTTGGAAAAGGGAGA
AAAAAGCCAATGAAGGTAAAAAAAGGAGGCGGGAAAATATCTAGATTTTTTAAAGAAGTA
GACAAATTCTTGTTTGACGATTTGGATGGTTAT
>China_LZCH20 .
ATGAAAGGAATAATGAATGGTTCCCTTTGTTTAAGGAAAATTTCCCTGTGTTCTATTTTC
GTATTGGCATATCTCAGTTCTGCTAAGTGCGCGTACCGAGACGAATCTGACATATTGTAC
AGCGACGACTCCAGATCCGATTTAGCAAGCAACGGGGCACCCACCGACAGCTACGAATCT
TTAACAGCAAGTAGTGAGTCTCTAGCAGAAAGCAACGATGCACCCAGCAACAGCTATGAA
TCTTTTCCAGAAATTAGAGAAAATCTAACCGCAAGTGAGGAATCCCTAACATCATGTGAG
GAATCCCTAACAGGAAGTAATGAATCCCTAACAGGAAGTAATGAATCCCTAACAGGAAGT
AAT---------------------------------------------------------
------GAATCCCTAACAGGAAGTAATGAATCCCTAACAGGAAGTAATGAATCCCTAACA
GAAAGTAGAGAATCTCTAGAGGCCAGTAGAGAATCGCTAAGAGCAAGTAGAGAGTCTCTA
GCGGCCAGTAGAGAATCCCTGAACGACTTTTGTGGGAGCGAAGAATCAGTAGCATGCGAA
GGAGAGCCAAATGAAAAGACATTCATGGGAGACGTCTTAAGTGGTGGAGAATGTGAGAAT
AGTCTCTCAAGAGAAGATTTATTTCATATAGAAGTAGGATCCGAAGAATCGCTAGATGAT
GCCTCAAAATATAATTTCCAAAAGGATTTATCAACTAGCGATAATAGTTTATTCGAAGAT
GATCAGTCATTGAAAAGGGGACTCAAAAGGAACAGCTCAGTATCTAGTCTGGACAGCGAT
ATGGGAAGTTATAAAAATAAAAGTTATAGACACAGATTGGATATATATTCGGACCTACCT
AGAAGGCCAATACATGGGGATAATGCACCACAAGAAAAGGAGAAATGTCTGGATTTCAAA
GAACTTCTAGAGAGAGAAAGGAACAACCACCAAAGGAATAGTGTAAATATAAAATTAAAA
AATATATACGGAGAAGAACAATTGTATAATGTGAAGAAAGATAGCTTTATTGTAAAATTG
ATATACTATCTGCCGTTTGGCCCAATTGTTACAATGATAATGATAGGCTTTATTCTACTC

TGCTCAACGCCCTCGACTGGTTTTATTTTTATAGGGTGTAGCTCTATTATATTACCAGCT
TTGTTATACGCGATGTATTCAAACAAAAAACAATTCGAACGTGTATTTGGAAAAGGGAGA
AAAAAGCCAATGAAGGTAAAAAAAGGAGGCGGGAAAATATCTAGATTTTTTAAAGAAGTA
GACAAATTCTTGTTTGACGATTTGGATGGTTAT
>C08 .
ATGAAAGGAATAATGAATGGTTCCCTTTGTTTAAGGAAAATTTCCCTGTGTTCTATTTTC
GTATTGGCATATCTCAGTTCTGCTAAGTGCGCGTACCGAGACGAATCTGACATATTGTAC
AGCGACGACTCCAGATCCGATTTAGCAAGCAACGGGGCACCCACCGACAGCTACGAATCT
TTAACAGCAAGTAGTGAGTCTCTAGCAGAAAGCAACGATGCACCCAGCAACAGCTATGAA
TCTTTTCCAGAAATTAGAGAAAATCTAACCGCAAGTGAGGAATCCCTAACATCATGTGAG
GAATCCCTAACAGGAAGTAATGAATCCCTAACAGGAAGTAATGAATCCCTAACAGGAAGT
AAT---------------------------------------------------------
------GAATCCCTAACAGGAAGTAATGAATCCCTAACAGGAAGTAATGAATCCCTAACA
GAAAGTAGAGAATCTCTAGAGGCCAGTAGAGAATCGCTAAGAGCAAGTAGAGAGTCTCTA
GCGGCCAGTAGAGAATCCCTGAACGACTTTTGTGGGAGCGAAGAATCAGTAGCATGCGAA
GGAGAGCCAAATGAAAAGACATTCATGGGAGACGTCTTAAGTGGTGGAGAATGTGAGAAT
AGTCTCTCAAGAGAAGATTTATTTCATATAGAAGTAGGATCCGAAGAATCGCTAGATGAT
GCCTCAAAATATAATTTCCAAAAGGATTTATCAACTAGCGATAATAGTTCATTCGAAGAT
GATCAGTCATTGAAAAGGGGACTCAAAAGGAACAGCTCAGTATCTAGTCTGGACAGCGAT
ATGGGAAGTTATAAAAATAAAAGTTATAGACACAGATTGGATATATATTCGGACCTACCT
AGAAGGCCAATACATGGGGATAATGCACCACAAGAAAAGGAGAAATGTCTGGATTTCAAA
GAACTTCTAGAGAGAGAAAGGAACAACCACCAAAGGAATAGTGTAAATATAAAATTAAAA
AATATATACGGAGAAGAACAATTGTATAATGTGAAGAAAGATAGCTTTATTGTAAAATTG
ATATACTATCTGCCGTTTGGCCCAATTGTTACAATGATAATGATAGGTTTTTTTCTACTC
TGCTGGACCCCCTCGATTGGTTTTACTTTTATAGGGTGTAGCTCTATTATATTACCAGCT
TTGTTATACGCGATGTATTCAAACAAAAAACAATTCGAACGTGTATTTGGAAAAGGGAGA
AAAAAGCCAATGAAGGTAAAAAAAGGAGGCGGGAAAATATCTAGATTTTTTAAAGAAGTA
GACAAATTCTTGTTTGACGATTTGGATGGTTAT
>M08 .
ATGAAAGGAATAATGAATGGTTCCCTTTGTTTAAGGAAAATTTCCCTGTGTTCTATTTTC
GTATTGGCATATCTCAGTTCTGCTAAGTGCGCGTACCGAGACGAATCTGACATATTGTAC
AGCGACGACTCCAGATCCGATTTAGCAAGCAACGGGGCACCCACCGACAGCTACGAATCT
TTAACAGCAAGTAGTGAGTCTCTAGCAGAAAGCAACGATGCACCCAGCAACAGCTATGAA
TCTTTTCCAGAAATTAGAGAAAATCTAACCGCAAGTGAGGAATCCCTAACATCATGTGAG
GAATCCCTAACAGGAAGTAATGAATCCCTAACAGGAAGTAATGAATCCCTAACAGGAAGT
AAT---------------------------------------------------------
------GAATCCCTAACAGGAAGTAATGAATCCCTAACAGGAAGTAATGAATCCCTAACA
GAAAGTAGAGAATCTCTAGAGGCCAGTAGAGAATCGCTAAGAGCAAGTAGAGAGTCTCTA
GCGGCCAGTAGAGAATCCCTGAACGACTTTTGTGGGAGCGAAGAATCAGTAGCATGCGAA
GGAGAGCCAAATGAAAAGACATTCATGGGAGACGTCTTAAGTGGTGGAGAATGTGAGAAT
AGTCTCTCAAGAGAAGATTTATTTCATATAGAAGTAGGATCCGAAGAATCGCTAGATGAT
GCCTCAAAATATAATTTCCAAAAGGATTTATCAACTAGCGATAATAGTTCATTCGAAGAT
GATCAGTCATTGAAAAGGGGACTCAAAAGGAACAGCTCAGTATCTAGTCTGGACAGCGAT
ATGGGAAGTTATAAAAATAAAAGTTATAGACACAGATTGGATATATATTCGGACCTACCT
AGAAGGCCAATACATGGGGATAATGCACCACAAGAAAAGGAGAAATGTCTGGATTTCAAA
GAACTTCTAGAGAGAGAAAGGAACAACCACCAAAGGAATAGTGTAAATATAAAATTAAAA
AATATATACGGAGAAGAACAATTGTATAATGTGAAGAAAGATAGCTTTATTGTAAAATTG
ATATACTATCTGCCGTTTGGCCCAATTGTTACAATGATAATGATAGGCTTTATTCTACTC
TGCTGGACCCCCTCGATTGGTTTTATTTTTATAGGGTGTAGCTCTATTATATTACCAGCT
TTGATATACGCGATGCATTCAAACAAAAAACAATTCGAACGTGTATTTGGAAAAGGGAGA

```
AAAAAGCCAATGAAGGTAAAAAAAGGAGGCGGGAAAATATCTAGATTTTTTAAAGAAGTA
GACAAATTCTTGTTTGACGATTTGGATGGTTAT
>M15 .
ATGAAAGGAATAATGAATGGTTCCCTTTATTTAAGGAAAATTTCCCTGTGTTCTATTTTC
GTATTGGCATATCTCAGTTCTGCTAAGTGCGCGTACCGAGACGAATCTGACATATTGTAC
AGCGACGACTCCAGATCCGATTTAGCAAGCAACGGGGCACCCACCGACAGCTACGAATCT
TTAACAGCAAGTAGTGAGTCTCTAGCAGAAAGCAACGATGCACCCAGCAACAGCTATGAA
TCTTTTCCAGAAATTAGAGAAAATCTAACCGCAAGTGAGGAATCCCTAACATCATGTGAG
GAATTCCTAACAGGAAGTAATGAATCCCTAACAGGAAGTAATGAATCCCTAACAGGAAGT
AAT---------------------------------------------------------
------GAATCCCTAACAGGAAGTAATGAATCCCTAACAGGAAGTAATGAATCCCTAACA
GAAAGTAGAGAATCTCTAGAGGCCAGTAGAGAATCGCTAAGAGCAAGTAGAGAGTCTCTA
GCGGCCAGTAGAGAATCCCTGAACGACTTTTGTGGGAGCGAAGAATCAGTAGCATGCGAA
GGAGAGCCAAATGAAAAGACATTCATGGGAGACGTCTTAAGTGGTGGAGAATGTGAGAAT
AGTCTCTCAAGAGAAGATTTATTTCATATAGAAGTAGGATCCGAAGAATCGCTAGATGAT
GCCTCAAAATATAATTTCCAAAAGGATTTATCAACTAGCGATAATAGTTCATTCGAAGAT
GATCAGTCATTGAAAAGGGGACTCAAAAGGAACAGCTCAGTATCTAGTCTGGACAGCGAT
ATGGGAAGTTATAAAAATAAAAGTTATAGACACAGATTGGATATATATTCGGACCTACCT
AGAAGGCCAATACATGGGGATAATGCACCACAAGAAAAGGAGAAATGTCTGGATTTCAAA
GAACTTCTAGAGAGAGAAAGGAACAACCACCAAAGGAATAGTGTAAATATAAAATTAAAA
AATATATACGGAGAAGAACAATTGTATAATGTGAAGAAAGATAGCTTTATTGTAAAATTG
ATATACTATCTGCCGTTTGGCCCAATTGTTACAATGATAATGATAGGCTTTATTCTACTT
TGGTCAACGCCCTGGACTGGTTTTATTTTTATAGGGTGTAGCTCTATTATATTACCAGCT
TTGTTATACGCGATGCATTCAAACAAAAAACAATTCGAACGTGTATTTGGAAAAGGGAGA
AAAAAGCCAATGAAGGTAAAAAAAGGAGGCGGGAAAATATCTAGATTTTTTAAAGAAGTA
GACAAATTCTTGTTTGACGATTTGGATGGTTAT
>C127 .
ATGAAAGGAATAATGAATGGTTCCCTTTGTTTAAGGAAAATTTCCCTGTGTTCTATTTTC
GTATTGGCATATCTCAGTTCTGCTAAGTGCGCGTACCGAGACGAATCTGACATATTGTAC
AGCGACGACTCCAGATCCGATTTAGCAAGCAACGGGGCACCCACCGACAGCTACGAATCT
TTAACAGCAAGTAGTGAGTCTCTAGCAGAAAGCAACGATGCACCCAGCAACAGCTATGAA
TCTTTTCCAGAAATTAGAGAAAATCTAACCGCAAGTGAGGAATCCCTAACATCATGTGAG
GAATCCCTAACAGGAAGTAATGAATCCCTAACAGGAAGTAATGAATCCCTAACAGGAAGT
AAT---------------------------------------------------------
------GAATCCCTAACAGGAAGTAATGAATCCCTAACAGGAAGTAATGAATCCCTAACA
GAAAGTAGAGAATCTCTAGAGGCCAGTAGAGAATCGCTAAGAGCAAGTAGAGAGTCTCTA
GCGGCCAGTAGAGAATCCCTGAACGACTTTTGTGGGAGCGAAGAATCAGTAGCATTCGAA
GGAGAGCCAAATGAAAAGACATTCATGGGAGACGTCTTAAGTGGTGGAGAATGTGAGAAT
AGTCTCTCAAGAGAAGATTTATTTCATATAGAAGTAGGATCCGAAGAATCGCTAGATGAT
GCCTCAAAATATAATTTCCAAAAGGATTTATCAACTAGCGATAATAGTTCATTCGAAGAT
GATCAGTCATTGAAAAGGGGACTCAAAAGGAACAGCTCAGTATCTAGTCTGGACAGCGAT
ATGGGAAGTTATAAAAATAAAAGTTATAGACACAGATTGGATATATATTCGGACCTACCT
AGAAGGCTAATACATGGGGATAATGCACCACAAGAAAAGGAGAAATGTCTGGATTTCAAA
GAACTTCTAGAGAGAGAAAGGAACAACCACCAAAGGAATAGTGTAAATATAAAATTAAAA
AATATATACGGAGAAGAACAATTGTATAATGTGAAGAAAGATAGCTTTATTGTAAAATTG
ATATACTATTTGCCGTTTGGCCCAATTGTTACAATGATAATGATAGGCTTTATTCTACTC
```

TGGTCAACGCCCTGGACTGGTTTTATTTTTATAGGGTGTAGCTCTATTATATTACCAGCT
TTGTTATACGCGATGCATTCAAACAAAAAACAATTCGAACGTGTATTTGGAAAAGGGAGA
AAAAAGCCAATGAAGGTAAAAAAAGGAGGCGGGAAAATATCTAGATTTTTTAAAGAAGTA
GACAAATTCTTGTTTGACGATTTGGATGGTTAT
>Chesson .
ATGAAAGGAATAATGAATGGTTCCCTTTGTTTAAGGAAAATTTCCCTGTGTTCTATTTTC
GTATTGGCATATCTCAGTTCTGCTAAGTGCGCGTACCGAGACGAATCTGACATATTGTAC
AGCGACGACTCCAGATCCGATTTAGCAAGCAACGGGGCACCCACCGACAGCTACGAATCT
TTAACAGCAAGTAGTGAGTCTCTAGCAGAAAGCAACGATGCACCCAGCAACAGCTATGAA
TCTTTTCCAGAAATTAGAGAAAATCTAACCGCAAGTGAGGAATCCCTAACATCATGTGAG
GAATCCCTAACAGGAAGTAATGAATCCCTAACAGGAAGTAATGAATCCCTAACAGGAAGT
AAT---------------------------------------------------------
------GAATCCCTAACAGGAAGTAATGAATCCCTAACAGGAAGTAATGAATCCCTAACA
GAAAGTAGAGAATCTCTAGAGGCCAGTAGAGAATCGCTAAGAGCAAGTAGAGAGTCTCTA
GCGGCCAGTAGAGAATCCCTGAACGACTTTTGTGGGAGCGAAGAATCAGTAGCATTCGAA
GGAGAGCCAAATGAAAAGACATTCATGGGAGACGTCTTAAGTGGTGGAGAATGTGAGAAT
AGTCTCTCAAGAGAAGATTTATTTCATATAGAAGTAGGATCCGAAGAATCGCTAGATGAT
GCCTCAAAATATAATTTCCAAAAGGATTTATCAACTAGCGATAATAGTTCATTCGAAGAT
GATCAGTCATTGAAAAGGGGACTCAAAAGGAACAGCTCAGTATCTAGTCTGGACAGCGAT
ATGGGAAGTTATAAAAATAAAAGTTATAGACACAGATTGGATATATATTCGGACCTACCT
AGAAGGCCAATACATGGGGATAATGCACCACAAGAAAAGGAGAAATGTCTGGATTTCAAA
GAACTTCTAGAGAGAGAAAGGAACAACCACCAAAGGAATAGTGTAAATATAAAATTAAAA
AATATATACGGAGAAGAACAATTGTATAATGTGAAGAAAGATAGCTTTATTGTAAAATTG
ATATACTATCTGCCGTTTGGCCCAATTGTTACAATGATAATGATAGGCTTTATTCTACTC
TGGTCAACGCCCTCGATTGGTTTTATTTTTATAGGGTGTAGCTCTATTATATTACCAGCT
TTGTTATACGCGATGCATTCAAACAAAAAACAATTCGAACGTGTATTTGGAAAAGGGAGA
AAAAAGCCAATGAAGGTAAAAAAAGGAGGCGGGAAAATATCTAGATTTTTTAAAGAAGTA
GACAAATTCTTGTTTGACGATTTGGATGGTTAT
>PNG58 .
ATGAAAGGAATAATGAATGGTTCCCTTTGTTTAAGGAAAATTTCCCTGTGTTCTATTTTC
GTATTGGCATATCTCAGTTCTGCTAAGTGCGCGTACCGAGACGAATCTGACATATTGTAC
AGCGACGACTCCAGATCCGATTTAGCAAGCAACGGGGCACCCACCGACAGCTACGAATCT
TTAACAGCAAGTAGTGAGTCTCTAGCAGAAAGCAACGATGCACCCAGCAACAGCTATGAA
TCTTTTCCAGAAATTAGAGAAAATCTAACCGCAAGTGAGGAATCCCTAACATCATGTGAG
GAATCCCTAACAGGAAGTAATGAATCCCTAACAGGAAGTAATGAATCCCTAACAGGAAGT
AAT---------------------------------------------------------
------GAATCCCTAACAGGAAGTAATGAATCCCTAACAGGAAGTAATGAATCCCTAACA
GAAAGTAGAGAATCTCTAGAGGCCAGTAGAGAATCGCTAAGAGCAAGTAGAGAGTCTCTA
GCGGCCAGTAGAGAATCCCTGAACGACTTTTGTGGGAGCGAAGAATCAGTAGCATTCGAA
GGAGAGCCAAATGAAAAGACATTCATGGGAGACGTCTTAAGTGGTGGAGAATGTGAGAAT
AGTCTCTCAAGAGAAGATTTATTTCATATAGAAGTAGGATCCGAAGAATCGCTAGATGAT
GCCTCAAAATATAATTTCCAAAAGGATTTATCAACTAGCGATAATAGTTCATTCGAAGAT
GATCAGTCATTGAAAAGGGGACTCAAAAGGAACAGCTCAGTATCTAGTCTGGACAGCGAT
ATGGGAAGTTATAAAAATAAAAGTTATAGACACAGATTGGATATATATTCGGACCTACCT
AGAAGGCCAATACATGGGGATAATGCACCACAAGAAAAGGAGAAATGTCTGGATTTCAAA
GAACTTCTAGAGAGAGAAAGGAACAACCACCAAAGGAATAGTGTAAATATAAAATTAAAA
AATATATACGGAGAAGAACAATTGTATAATGTGAAGAAAGATAGCTTTATTGTAAAATTG
ATATACTATCTGCCGTTTGGCCCAATTGTTACAATGATAATGATAGGCTTTATTCTACTC
TGGTCAACGCCCTGGACTGGTTTTATTTTTATAGGGTGTAGCTCTATTATATTACCAGCT
TTGTTATACGCGATGCATTCAAACAAAAAACAATTCGAACGTGTATTTGGAAAAGGGGGA

AAAAAGCCAATGAAGGTAAAAAAAGGAGGCGGGAAAATATCTAGATTTTTTAAAGAAGTA
GACAAATTCTTGTTTGACGATTTGGATGGTTAT
>PNG72 .
ATGAAAGGAATAATGAATGGTTCCCTTTGTTTAAGGAAAATTTCCCTGTGTTCTATTTTC
GTATTGGCATATCTCAGTTCTGCTAAGTGCGCGTACCGAGACGAATCTGACATATTGTAC
AGCGACGACTCCAGATCCGATTTAGCAAGCAACGGGGCACCCACCGACAGCTACGAATCT
TTAACAGCAAGTAGTGAGTCTCTAGCAGAAAGCAACGATGCACCCAGCAACAGCTATGAA
TCTTTTCCAGAAATTAGAGAAAATCTAACCGCAAGTGAGGAATCCCTAACATCATGTGAG
GAATCCCTAACAGGAAGTAATGAATCCCTAACAGGAAGTAATGAATCCCTAACAGGAAGT
AAT---------------------------------------------------------
------GAATCCCTAACAGGAAGTAATGAATCCCTAACAGGAAGTAATGAATCCCTAACA
GAAAGTAGAGAATCTCTAGAGGCCAGTAGAGAATCGCTAAGAGCAAGTAGAGAGTCTCTA
GCGGCCAGTAGAGAATCCCTGAACGACTTTTGTGGGAGCGAAGAATCAGTAGCATTCGAA
GGAGAGCCAAATGAAAAGACATTCATGGGAGACGTCTTAAGTGGTGGAGAATGTGAGAAT
AGTCTCTCAAGAGAAGATTTATTTCATATAGAAGTAGGATCCGAAGAATCGCTAGATGAT
GCCTCAAAATATAATTTCCAAAGGGATTTATCAACTAGCGATAATAGTTCATTCGAAGAT
GATCAGTCATTGAAAAGGGGACTCAAAAGGAACAGCTCAGTATCTAGTCTGGACAGCGAT
ATGGGAAGTTATAAAAATAAAAGTTATAGACACAGATTGGATATATATTCGGACCTACCT
AGAAGGCCAATACATGGGGATAATGCACCACAAGAAAAGGAGAAATGTCTGGATTTCAAA
GAACTTCTAGAGAGAGAAAGGAACAACCACCAAAGGAATAGTGTAAATATAAAATTAAAA
AATATATACGGAGAAGAACAATTGTATAATGTGAAGAAAGATAGCTTTATTGTAAAATTG
ATATACTATCTGCCGTTTGGCCCAATTGTTACAATGATAATGATAGGCTTTATTCTACTC
TGGTCAACGCCCTGGACTGGTTTTATTTTTATAGGGTGTAGCTCTATTATATTACCAGCT
TTGTTATACGCGATGCATTCAAACAAAAAACAATTCGAACGTGTATTTGGAAAAGGGAGA
AAAAAGCCAATGAAGGTAAAAAAAGGAGGCGGGAAAATATCTAGATTTTTTAAAGAAGTA
GACAAATTCTTGTTTGACGATTTGGATGGTTAT
>XUC014 .
ATGAAAGGAATAATGAATGGTTCCCTTTGTTTAAGGAAAATTTCCCTGTGTTCTATTTTC
GTATTGGCATATCTCAGTTCTGCTAAGTGCGCGTACCGAGACGAATCTGACATATTGTAC
AGCGACGACTCCAGATCCGATTTAGCAAGCAACGGGGCACCCACCGACAGCTACGAATCT
TTAACAGCAAGTAGTGAGTCTCTAGCAGAAAGCAACGATGCACCCAGCAACAGCTATGAA
TCTTTTCCAGAAATTAGAGAAAATCTAACCGCAAGTGAGGAATCCCTAACATCATGTGAG
GAATCCCTAACAGGAAGTAATGAATCCCTAACAGGAAGTAATGAATCCCTAACAGGAAGT
AAT---------------------------------------------------------
------GAATCCCTAACAGGAAGTAATGAATCCCTAACAGGAAGTAATGAATCCCTAACA
GAAAGTAGAGAATCTCTAGAGGCCAGTAGAGAATCGCTAAGAGCAAGTAGAGAGTCTCTA
GCGGCCAGTAGAGAATCCCTGAACGACTTTTGTGGGAGCGAAGAATCAGTAGCATTCGAA
GGAGAGCCAAATGAAAAGACATTCATGGGAGACGTCTTAAGTGGTGGAGAATGTGAGAAT
AGTCTCTCAAGAGAAGATTTATTTCATATAGAAGTAGGATCCGAAGAATCGCTAGATGAT
GCCTCAAAATATAATTTCCAAAAGGATTTATCAACTAGCGATAATAGTTCATTCGAAGAT
GATCAGTCATTGAAAAGGGGACTCAAAAGGAACAGCTCAGTATCTAGTCTGGACAGCGAT
ATGGGAAGTTATAAAAATAAAAGTTATAGACACAGATTGGATATATATTCGGACCTACCT
AGAAGGCCAATACATGGGGATAATGCACCACAAGAAAAGGAGAAATGTCTGGATTTCAAA
GAACTTCTAGAGAGAGAAAGGAACAACCACCAAAGGAATAGTGTAAATATAAAATTAAAA
AATATATACGGAGAAGAACAATTGTATAATGTGAAGAAAGATAGCTTTATTGTAAAATTG
ATATACTATCTGCCGTTTGGCCCAATTGTTACAATGATAATGATAGGCTTTATTCTACTC

TGGTCAACGCCCTGGACTGGTTTTATTTTTATAGGGTGTAGCTCTATTGTATTACCAGCT
TTGTTATACGCGATGCATTCAAACAAAAAACAATTCGAACGTGTATTTGGAAAAGGGGGA
AAAAAGCCAATGAAGGTAAAAAAAGGAGGCGGGAAAATATCTAGATTTTTTAAAGAAGTA
GACAAATTCTTGTTTGACGATTTGGATGGTTAT
>XUA070 .
ATGAAAGGAATAATGAATGGTTCCCTTTGTTTAAGGAAAATTTCCCTGTGTTCTATTTTC
GTATTGGCATATCTCAGTTCTGCTAAGTGCGCGTACCGAGACGAATCTGACATATTGTAC
AGCGACGACTCCAGATCCGATTTAGCAAGCAACGGGGCACCCACCGACAGCTACGAATCT
TTAACAGCAAGTAGTGAGTCTCTAGCAGAAAGCAACGATGCACCCAGCAACAGCTATGAA
TCTTTTCCAGAAATTAGAGAAAATCTAACCGCAAGTGAGGAATCCCTAACATCATGTGAG
GAATCCCTAACAGGAAGTAATGAATCCCTAACAGGAAGTAATGAATCCCTAACAGGAAGT
AAT---------------------------------------------------------
------GAATCCCTAACAGGAAGTAATGAATCCCTAACAGGAAGTAATGAATCCCTAACA
GAAAGTAGAGAATCTCTAGAGGCCAGTAGAGAATCGCTAAGAGCAAGTAGAGAGTCTCTA
GCGGCCAGTAGAGAATCCCTGAACGACTTTTGTGGGAGCGAAGAATCAGTAGCATTCGAA
GGAGAGCCAAATGAAAAGACATTCATGGGAGACGTCTTAAGTGGTGGAGAATGTGAGAAT
AGTCTCTCAAGAGAAGATTTATTTCATATAGAAGTAGGATCCGAAGAATCGCTAGATGAT
GCCTCAAAATATAATTTCCAAAAGGATTTATCAACTAGCGATAATAGTTCATTCGAAGAT
GATCAGTCATTGAAAAGGGGACTCAAAAGGAACAGCTCAGTATCTAGTCTGGACAGCGAT
ATGGGAAGTTATAAAAATAAAAGTTATAGACACAGATTGGATATATATTCGGACCTACCT
AGAAGGCCAATACATGGGGATAATGCACCACAAGAAAAGGAGAAATGTCTGGATTTCAAA
GAACTTCTAGAGAGAGAAAGGAACAACCACCAAAGGAATAGTGTAAATATAAAATTAAAA
AATATATACGGAGAAGAACAATTGTATAATGTGAAGAAAGATAGCTTTATTGTAAAATTG
ATATACTATCTGCCGTTTGGCCCAATTGTTACAATGATAATTATAGGCTTTATTCTACTC
TGCTCAACGCCCTCGACTGGTTTTATTTTTATAGGGTGTAGCTCTATTATATTACCAGCT
TTGTTATACGCGATGCATTCAAACAAAAAACAATTCGAACGTGTATTTGGAAAAGGGAGA
AAAAAGCCAATGAAGGTAAAAAAAGGAGGCGGGAAAATATCTAGATTTTTTAAAGAAGTA
GACAAATTCTTGTTTGACGATTTGGATGGTTAT
>DTS0830 .
ATGAAAGGAATAATGAATGGTTCCCTTTATTTAAGGAAAATTTCCCTGTGTTCTATTTTC
GTATTGGCATATCTCAGTTCTGCTAAGTGCGCGTACCGAGACGAATCTGACATATTGTAC
AGCGACGACTCCAGATCCGATTTAGCAAGCAACGGGGCACCCACCGACAGCTACGAATCT
TTAACAGCAAGTAGTGAGTCTCTAGCAGAAAGCAACGATGCACCCAGCAACAGCTATGAA
TCTTTTCCAGAAATTAGAGAAAATCTAACCGCAAGTGAGGAATCCCTAACATCATGTGAG
GAATCCCTAACAGGAAGTAATGAATCCCTAACAGGAAGTAATGAATCCCTAACAGGAAGT
AAT---------------------------------------------------------
------GAATCCCTAACAGGAAGTAATGAATCCCTAACAGGAAGTAATGAATCCCTAACA
GAAAGTAGAGAATCTCTAGAGGCCAGTAGAGAATCGCTAAGAGCAAGTAGAGAGTCTCTA
GCGGCCAGTAGAGAATCCCTGAACGACTTTTGTGGGAGCGAAGAATCAGTAGCATTCGAA
GGAGAGCCAAATGAAAAGACATTCATGGGAGACGTCTTAAGTGGTGGAGAATGTGAGAAT
AGTCTCTCAAGAGAAGATTTATTTCATATAGAAGTAGGATCCGAAGAATCGCTAGATGAT
GCCTCAAAATATAATTTCCAAAAGGATTTATCAACTAGCGATAATAGTTCATTCGAAGAT
GATCAGTCATTGAAAAGGGGACTCAAAAGGAACAGCTCAGTATCTAGTCTGGACAGCGAT
ATGGGAAGTTATAAAAATAAAAGTTATAGACACAGATTGGATATATATTCGGACCTACCT
AGAAGGCCAATACATGGGGATAATGCACCACAAGAAAAGGAGAAATGTCTGGATTTCAAA
GAACTTCTAGAGAGAGAAAGGAACAACCACCAAAGGAATAGTGTAAATATAAAATTAAAA
AATATATACGGAGAAGAACAATTGTATAATGTGAAGAAAGATAGCTTTATTGTAAAATTG
ATATACTATCTGCCGTTTGGCCCAATTGTTACAATGATAATGATAGGCTTTATTCTACTC
TGCTCAACGCCCTCGACTGGTTTTATTTTTATAGGGTGTAGCTCTATTATATTACCAGCT
TTGTTATACGCGATGCATTCAAACAAAAAACAATTCGAACGTGTATTTGGAAAAGGGAGA

```
AAAAAGCCAATGAAGGTAAAAAAAGGAGGCGGGAAAATATCTAGATTTTTTAAAGAAGTA
GACAAATTCTTGTTTGACGATTTGGATGGTTAT
>DTS0839 .
ATGAAAGGAATAATGAATGGTTCCCTTTGTTTAAGGAAAATTTCCCTGTGTTCTATTTTC
GTATTGGCATATCTCAGTTCTGCTAAGTGCGCGTACCGAGACGAATCTGACATATTGTAC
AGCGACGACTCCAGATCCGATTTAGCAAGCAACGGGGCACCCACCGACAGCTACGAATCT
TTAACAGCAAGTAGTGAGTCTCTAGCAGAAAGCAACGATGCACCCAGCAACAGCTATGAA
TCTTTTCCAGAAATTAGAGAAAATCTAACCGCAAGTGAGGAATCCCTAACATCATGTGAG
GAATCCCTAACAGGAAGTAATGAATCCCTAACAGGAAGTAATGAATCCCTAACAGGAAGT
AAT---------------------------------------------------------
------GAATCCCTAACAGGAAGTAATGAATCCCTAACAGGAAGTAATGAATCCCTAACA
GAAAGTAGAGAATCTCTAGAGGCCAGTAGAGAATCGCTAAGAGCAAGTAGAGAGTCTCTA
GCGGCCAGTAGAGAATCCCTGAACGACTTTTGTGGGAGCGAAGAATCAGTAGCATTCGAA
GGAGAGCCAAATGAAAAGACATTCATGGGAGACGTCTTAAGTGGTGGAGAATGTGAGAAT
AGTCTCTCAAGAGAAGATTTATTTCATATAGAAGTAGGATCCGAAGAATCGCTAGATGAT
GCCTCAAAATATAATTTCCAAAAGGATTTATCAACTAGCGATAATAGTTCATTCGAAGAT
GATCAGTCATTGAAAAGGGGACTCAAAAGGAACAGCTCAGTATCTAGTCTGGACAGCGAT
ATGGGAAGTTATAAAAATGGAAGATCTAGAGACAGATTGGATATATATTCGGACCTACCT
AGAAGGCCAATACATGGGGATAATGCACCACAAGAAAAGGAGAAATGTCTGGATTTCAAA
GAACTTCTAGAGAGAGAAAGGAACAACCACCAAAGGAATAGTGTAAATATAAAATTAAAA
AATATATACGGAGAAGAACAATTGTATAATGTGAAGAAAGATAGCTTTATTGTAAAATTG
ATATACTATCTGCCGTTTGGCCCAATTGTTACAATGATAATGATAGGCTTTATTCTACTC
TGCTCAACGCCCTCGACTGGTTTTATTTTTATAGGGTGTAGCTCTATTATATTACCAGCT
TTGTTATACGCGATGCATTCAAACAAAAAACAATTCGAACGTGTATTTGGAAAAGGGAGA
AAAAAGCCAATGAAGGTAAAAAAAGGAGGCGGGAAAATATCTAGATTTTTTAAAGAAGTA
GACAAATTCTTGTTTGACGATTTGGATGGTTAT
>DTS0791 .
ATGAAAGGAATAATGAATGGTTCCCTTTGTTTAAGGAAAATTTCCCTGTGTTCTATTTTC
GTATTGGCATATCTCAGTTCTGCTAAGTGCGCGTACCGAGACGAATCTGACATATTGTAC
AGCGACGACTCCAGATCCGATTTAGCAAGCAACGGGGCACCCACCGACAGCTACGAATCT
TTAACAGCAAGTAGTGAGTCTCTAGCAGAAAGCAACGATGCACCCAGCAACAGCTATGAA
TCTTTTCCAGAAATTAGAGAAAATCTAACCGCAAGTGAGGAATCCCTAACATCATGTGAG
GAATCCCTAACAGGAAGTAATGAATCCCTAACAGGAAGTAATGAATCCCTAACAGGAAGT
AAT---------------------------------------------------------
------GAATCCCTAACAGGAAGTAATGAATCCCTAACAGGAAGTAATGAATCCCTAACA
GAAAGTAGAGAATCTCTAGAGGCCAGTAGAGAATCGCTAAGAGCAAGTAGAGAGTCTCTA
GCGGCCAGTAGAGAATCCCTGAACGACTTTTGTGGGAGCGAAGAATCAGTAGCATTCGAA
GGAGAGCCAAATGAAAAGACATTCATGGGAGACGTCTTAAGTGGTGGAGAATGTGAGAAT
AGTCTCTCAAGAGAAGATTTATTTCATATAGAAGTAGGATCCGAAGAATCGCTAGATGAT
GCCTCAAAATATAATTTCCAAAAGGATTTATCAACTAGCGATAATAGTTCATTCGAAGAT
GATCAGTCATTGAAAAGGGGACTCAAAAGGAACAGCTCAGTATCTAGTCTGGACAGCGAT
ATGGGAAGTTATAAAAATGGAAGATCTAGAGACAGATTGGATATATATTCGGACCTACCT
AGAAGGCCAATACATGGGGATAATGCACCACAAGAAAAGGAGAAATGTCTGGATTTCAAA
GAACTTCTAGAGAGAGAAAGGAACAACCACCAAAGGAATAGTGTAAATATAAAATTAAAA
AATATATACGGAGAAGAACAATTGTATAATGTGAAGAAAGATAGCTTTATTGTAAAATTG
ATATACTATCTGCCGTTTGGCCCAATTGTTACAATGATAATGATAGGCTTTATTCTACTC
```

TGCTCAACGCCCTCGACTGGTTTTATTTTTATAGGGTGTAGCTCTATTATATTACCAGCT
TTGTTATACGCGATGCATTCAAACAAAAAACAATTCGAACGTGTATTTGGAAAAGGGAGA
AAAAAGCCAATGAAGGTAAAAAAAGGAGGCGGGAAAATATCTAGATTTTTTAAAGAAGTA
GACAAATTCTTGTTTGACGATTTGGATGGTTAT
>PVRVL1997 .
ATGAAAGGAATAATGAATGGTTCCCTTTGTTTAAGGAAAATTTCCCTGTGTTCTATTTTC
GTATTGGCATATCTCAGTTCTGCTAAGTGCGCGTACCGAGACGAATCTGACATATTGTAC
AGCGACGACTCCAGATCCGATTTAGCAAGCAACGGGGCACCCACCGACAGCTACGAATCT
TTAACAGCAAGTAGTGAGTCTCTAGCAGAAAGCAACGATGCACCCAGCAACAGCTATGAA
TCTTTTCCAGAAATTAGAGAAAATCTAACCGCAAGTGAGGAATCCCTAACATCATGTGAG
GAATCCCTAACAGGAAGTAATGAATCCCTAACAGGAAGTAATGAATCCCTAACAGGAAGT
AAT---------------------------------------------------------
------GAATCCCTAACAGGAAGTAATGAATCCCTAACAGGAAGTAATGAATCCCTAACA
GAAAGTAGAGAATCTCTAGAGGCCAGTAGAGAATCGCTAAGAGCAAGTAGAGAGTCTCTA
GCGGCCAGTAGAGAATCCCTGAACGACTTTTGTGGGAGCGAAGAATCAGTAGCATTCGAA
GGAGAGCCAAATGAAAAGACATTCATGGGAGACGTCTTAAGTGGTGGAGAATGTGAGAAT
AGTCTCTCAAGAGAAGATTTATTTCATATAGAAGTAGGATCCGAAGAATCGCTAGATGAT
GCCTCAAAATATAATTTCCAAAAGGATTTATCAACTAGCGATAATAGTTCATTCGAAGAT
GATCAGTCATTGAAAAGGGGACTCAAAAGGAACAGCTCAGTATCTAGTCTGGACAGCGAT
ATGGGAAGTTATAAAAATAAAAGTTATAGACACAGATTGGATATATATTCGGACCTACCT
AGAAGGCCAATACATGGGGATAATGCACCACAAGAAAAGGAGAAATGTCTGGATTTCAAA
GAACTTCTAGAGAGAGAAAGGAACAACCACCAAAGGAATAGTGTAAATATAAAATTAAAA
AATATATACGGAGAAGAACAATTGTATAATGTGAAGAAAGATAGCTTTATTGTAAAATTG
ATATACTATCTGCCGTTTGGCCCAATTGTTACAATGATAATGATAGGCTTTATTCTACTC
TGGTCAACGCCCTGGACTGGTTTTATTTTTATAGGGTGTAGCTCTATTATATTACCAGCT
TTGTTATACGCGATGCATTCAAACAAAAAACAATTCGAACGTGTATTTGGAAAAGGGAGA
AAAAAGCCAATGAAGGTAAAAAAAGGAGGCGGGAAAATATCTAGATTTTTTAAAGAAGTA
GACAAATTCTTGTTTGACGATTTGGATGGTTAT
>Columbia_30101099040 .
ATGAAAGGAATAATGAATGGTTCCCTTTATTTAAGGAAAATTTCCCTGTGTTCTATTTTC
GTATTGGCATATCTCAGTTCTGCTAAGTGCGCGTACCGAGACGAATCTGACATATTGTAC
AGCGACGACTCCAGATCCGATTTAGCAAGCAACGGGGCACCCACCGACAGCTACGAATCT
TTAACAGCAAGTAGTGAGTCTCTAGCAGAAAGCAACGATGCACCCAGCAACAGCTATGAA
TCTTTTCCAGAAATTAGAGAAAATCTAACCGCAAGTGAGGAATCCCTAACATCATGTGAG
GAATCCCTAACAGGAAGTAATGAATCCCTAACAGGAAGTAATGAATCCCTAACAGGAAGT
AAT---------------------------------------------------------
------GAATCCCTAACAGGAAGTAATGAATCCCTAACAGGAAGTAATGAATCCCTAACA
GAAAGTAGAGAATCTCTAGAGGCCAGTAGAGAATCGCTAAGAGCAAGTAGAGAGTCTCTA
GCGGCCAGTAGAGAATCCCTGAACGACTTTTGTGGGAGCGAAGAATCAGTAGCATTCGAA
GGAGAGCCAAATGAAAAGACATTCATGGGAGACGTCTTAAGTGGTGGAGAATGTGAGAAT
AGTCTCTCAAGAGAAGATTTATTTCATATAGAAGTAGGATCCGAAGAATCGCTAGATGAT
GCCTCAAAATATAATTTCCAAAAGGATTTATCAACTAGCGATAATAGTTCATTCGAAGAT
GATCAGTCATTGAAAAGGGGACTCAAAAGGAACAGCTCAGTATCTAGTCTGGACAGCGAT
ATGGGAAGTTATAAAAATAAAAGTTATAGACACAGATTGGATATATATTCGGACCTACCT
AGAAGGCCAATACATGGGGATAATGCACCACAAGAAAAGGAGAAATGTCTGGATTTCAAA
GAACTTCTAGAGAGAGAAAGGAACAACCACCAAAGGAATAGTGTAAATATAAAATTAAAA
AATATATACGGAGAAGAACAATTGTATAATGTGAAGAAAGATAGCTTTATTGTAAAATTG
ATATACTATCTGCCGTTTGGCCCAATTGTTACAATGATAATGATAGGTTTTATTCTACTC
TGGTCAACGCCCTGGACTGGTTTTATTTTTATAGGGTGTAGCTCTATTATATTACCAGCT
TTGTTATACGCGATGCATTCAAACAAAAAACAATTCGAACGTGTATTTGGAAAAGGGAGA

AAAAAGCCAATGAAGGTAAAAAAAGGAGGCGGGAAAATATCTAGATTTTTTAAAGAAGTA
GACAAATTCTTGTTTGACGATTTGGATGGTTAT
>Columbia_30101099036 .
ATGAAAGGAATAATGAATGGTTCCCTTTGTTTAAGGAAAATTTCCCTGTGTTCTATTTTC
GTATTGGCATATCTCAGTTCTGCTAAGTGCGCGTACCGAGACGAATCTGACATATTGTAC
AGCGACGACTCCAGATCCGATTTAGCAAGCAACGGGGCACCCACCGACAGCTACGAATCT
TTAACAGCAAGTAGTGAGTCTCTAGCAGAAAGCAACGATGCACCCAGCAACAGCTATGAA
TCTTTTCCAGAAATTAGAGAAAATCTAACCGCAAGTGAGGAATCCCTAACATCATGTGAG
GAATCCCTAACAGGAAGTAATGAATCCCTAACAGGAAGTAATGAATCCCTAACAGGAAGT
AAT---------------------------------------------------------
------GAATCCCTAACAGGAAGTAATGAATCCCTAACAGGAAGTAATGAATCCCTAACA
GAAAGTAGAGAATCTCTAGAGGCCAGTAGAGAATCGCTAAGAGCAAGTAGAGAGTCTCTA
GCGGCCAGTAGAGAATCCCTGAACGACTTTTGTGGGAGCGAAGAATCAGTAGCATTCGAA
GGAGAGCCAAATGAAAAGACATTCATGGGAGACGTCTTAAGTGGTGGAGAATGTGAGAAT
AGTCTCTCAAGAGAAGATTTATTTCATATAGAAGTAGGATCCGAAGAATCGCTAGATGAT
GCCTCAAAATATAATTTCCAAAAGGATTTATCAACTAGCGATAATAGTTCATTCGAAGAT
GATCAGTCATTGAAAAGGGGACTCAAAAGGAACAGCTCAGTATCTAGTCTGGACAGCGAT
ATGGGAAGTTATAAAAATAAAAGTTATAGACACAGATTGGATATATATTCGGACCTACCT
AGAAGGCCAATACATGGGGATAATGCACCACAAGAAAAGGAGAAATGTCTGGATTTCAAA
GAACTTCTAGAGAGAGAAAGGAACAACCACCAAAGGAATAGTGTAAATATAAAATTAAAA
AATATATACGGAGAAGAACAATTGTATAATGTGAAGAAAGATAGCTTTATTGTAAAATTG
ATATACTATCTGCCGTTTGGCCCAATTGTTACAATGATAATGATAGGCTTTATTCTACTC
TGCTCAACGCCCTCGACTGGTTTTATTTTTATAGGGTGTAGCTCTATTATATTACCAGCT
TTGTTATACGCGATGCATTCAAACAAAAAACAATTCGAACGTGTATTTGGAAAAGGGAGA
AAAAAGCCAATGAAGGTAAAAAAAGGAGGCGGGAAAATATCTAGATTTTTTAAAGAAGTA
GACAAATTCTTGTTTGACGATTTGGATGGTTAT
>Columbia_30102100490 .
ATGAAAGGAATAATGAATGGTTCCCTTTGTTTAAGGAAAATTTCCCTGTGTTCTATTTTC
GTATTGGCATATCTCAGTTCTGCTAAGTGCGCGTACCGAGACGAATCTGACATATTGTAC
AGCGACGACTCCAGATCCGATTTAGCAAGCAACGGGGCACCCACCGACAGCTACGAATCT
TTAACAGCAAGTAGTGAGTCTCTAGCAGAAAGCAACGATGCACCCAGCAACAGCTATGAA
TCTTTTCCAGAAATTAGAGAAAATCTAACCGCAAGTGAGGAATCCCTAACATCATGTGAG
GAATCCCTAACAGGAAGTAATGAATCCCTAACAGGAAGTAATGAATCCCTAACAGGAAGT
AAT---------------------------------------------------------
------GAATCCCTAACAGGAAGTAATGAATCCCTAACAGGAAGTAATGAATCCCTAACA
GAAAGTAGAGAATCTCTAGAGGCCAGTAGAGAATCGCTAAGAGCAAGTAGAGAGTCTCTA
GCGGCCAGTAGAGAATCCCTGAACGACTTTTGTGGGAGCGAAGAATCAGTAGCATTCGAA
GGAGAGCCAAATGAAAAGACATTCATGGGAGACGTCTTAAGTGGTGGAGAATGTGAGAAT
AGTCTCTCAAGAGAAGATTTATTTCATATAGAAGTAGGATCCGAAGAATCGCTAGATGAT
GCCTCAAAATATAATTTCCAAAAGGATTTATCAACTAGCGATAATAGTTCATTCGAAGAT
GATCAGTCATTGAAAAGGGGACTCAAAAGGAACAGCTCAGTATCTAGTCTGGACAGCGAT
ATGGGAAGTTATAAAAATAAAAGTTATAGACACAGATTGGATATATATTCGGACCTACCT
AGAAGGCCAATACATGGGGATAATGCACCACAAGAAAAGGAGAAATGTCTGGATTTCAAA
GAACTTCTAGAGAGAGAAAGGAACAACCACCAAAGGAATAGTGTAAATATAAAATTAAAA
AATATATACGGAGAAGAACAATTGTATAATGTGAAGAAAGATAGCTTTATTGTAAAATTG
ATATACTATCTGCCGTTTGGCCCAATTGTTACAATGATAATGATAGGCTTTATTCTACTC

TGGTCAACGCCCTGGACTGGTTTTATTTTTATAGGGTGTAGCTCTATTATATTACCAGCT
TTGTTATACGCGATGCATTCAAACAAAAAACAATTCGAACGTGTATTTGGAAAAGGGAGA
AAAAAGCCAATGAAGGTAAAAAAAGGAGGCGGGAAAATATCTAGATTTTTTAAAGAAGTA
GACAAATTCTTGTTTGACGATTTGGATGGTTAT
>Columbia_30102100441B .
ATGAAAGGAATAATGAATGGTTCCCTTTATTTAAGGAAAATTTCCCTGTGTTCTATTTTC
GTATTGGCATATCTCAGTTCTGCTAAGTGCGCGTACCGAGACGAATCTGACATATTGTAC
AGCGACGACTCCAGATCCGATTTAGCAAGCAACGGGGCACCCACCGACAGCTACGAATCT
TTAACAGCAAGTAGTGAGTCTCTAGCAGAAAGCAACGATGCACCCAGCAACAGCTATGAA
TCTTTTCCAGAAATTAGAGAAAATCTAACCGCAAGTGAGGAATCCCTAACATCATGTGAG
GAATCCCTAACAGGAAGTAATGAATCCCTAACAGGAAGTAATGAATCCCTAACAGGAAGT
AAT---------------------------------------------------------
------GAATCCCTAACAGGAAGTAATGAATCCCTAACAGGAAGTAATGAATCCCTAACA
GAAAGTAGAGAATCTCTAGAGGCCAGTAGAGAATCGCTAAGAGCAAGTAGAGAGTCTCTA
GCGGCCAGTAGAGAATCCCTGAACGACTTTTGTGGGAGCGAAGAATCAGTAGCATTCGAA
GGAGAGCCAAATGAAAAGACATTCATGGGAGACGTCTTAAGTGGTGGAGAATGTGAGAAT
AGTCTCTCAAGAGAAGATTTATTTCATATAGAAGTAGGATCCGAAGAATCGCTAGATGAT
GCCTCAAAATATAATTTCCAAAAGGATTTATCAACTAGCGATAATAGTTCATTCGAAGAT
GATCAGTCATTGAAAAGGGGACTCAAAAGGAACAGCTCAGTATCTAGTCTGGACAGCGAT
ATGGGAAGTTATAAAAATAAAAGTTATAGACACAGATTGGATATATATTCGGACCTACCT
AGAAGGCCAATACATGGGGATAATGCACCACAAGAAAAGGAGAAATGTCTGGATTTCAAA
GAACTTCTAGAGAGAGAAAGGAACAACCACCAAAGGAATAGTGTAAATATAAAATTAAAA
AATATATACGGAGAAGAACAATTGTATAATGTGAAGAAAGATAGCTTTATTGTAAAATTG
ATATACTATCTGCCGTTTGGCCCAATTGTTACAATGATAATGATAGGCTTTATTCTACTC
TGCTCAACGCCCTCGACTGGTTTTATTTTTATAGGGTGTAGCTCTATTATATTACCAGCT
TTGTTATACGCGATGCATTCAAACAAAAAACAATTCGAACGTGTATTTGGAAAAGGGAGA
AAAAAGCCAATGAAGGTAAAAAAAGGAGGCGGGAAAATATCTAGATTTTTTAAAGAAGTA
GACAAATTCTTGTTTGACGATTTGGATGGTTAT
>Columbia_30102100504 .
ATGAAAGGAATAATGAATGGTTCCCTTTATTTAAGGAAAATTTCCCTGTGTTCTATTTTC
GTATTGGCATATCTCAGTTCTGCTAAGTGCGCGTACCGAGACGAATCTGACATATTGTAC
AGCGACGACTCCAGATCCGATTTAGCAAGCAACGGGGCACCCACCGACAGCTACGAATCT
TTAACAGCAAGTAGTGAGTCTCTAGCAGAAAGCAACGATGCACCCAGCAACAGCTATGAA
TCTTTTCCAGAAATTAGAGAAAATCTAACCGCAAGTGAGGAATCCCTAACATCATGTGAG
GAATCCCTAACAGGAAGTAATGAATCCCTAACAGGAAGTAATGAATCCCTAACAGGAAGT
AAT---------------------------------------------------------
------GAATCCCTAACAGGAAGTAATGAATCCCTAACAGGAAGTAATGAATCCCTAACA
GAAAGTAGAGAATCTCTAGAGGCCAGTAGAGAATCGCTAAGAGCAAGTAGAGAGTCTCTA
GCGGCCAGTAGAGAATCCCTGAACGACTTTTGTGGGAGCGAAGAATCAGTAGCATTCGAA
GGAGAGCCAAATGAAAAGACATTCATGGGAGACGTCTTAAGTGGTGGAGAATGTGAGAAT
AGTCTCTCAAGAGAAGATTTATTTCATATAGAAGTAGGATCCGAAGAATCGCTAGATGAT
GCCTCAAAATATAATTTCCAAAAGGATTTATCAACTAGCGATAATAGTTCATTCGAAGAT
GATCAGTCATTGAAAAGGGGACTCAAAAGGAACAGCTCAGTATCTAGTCTGGACAGCGAT
ATGGGAAGTTATAAAAATAAAAGTTATAGACACAGATTGGATATATATTCGGACCTACCT
AGAAGGCCAATACATGGGGATAATGCACCACAAGAAAAGGAGAAATGTCTGGATTTCAAA
GAACTTCTAGAGAGAGAAAGGAACAACCACCAAAGGAATAGTGTAAATATAAAATTAAAA
AATATATACGGAGAAGAACAATTGTATAATGTGAAGAAAGATAGCTTTATTGTAAAATTG
ATATACTATCTGCCGTTTGGCCCAATTGTTACAATGATAATGATAGGCTTTATTCTACTC
TGCTCAACGCCCTCGACTGGTTTTATTTTTATAGGGTGTAGCTCTATTATATTACCAGCT
TTGTTATACGCGATGCATTCAAACAAAAAACAATTCGAACGTGTATTTGGAAAAGGGAGA

AAAAAGCCAATGAAGGTAAAAAAAGGAGGCGGGAAAATATCTAGATTTTTTAAAGAAGTA
GACAAATTCTTGTTTGACGATTTGGATGGTTAT
>Columbia_30102100489 .
ATGAAAGGAATAATGAATGGTTCCCTTTATTTAAGGAAAATTTCCCTGTGTTCTATTTTC
GTATTGGCATATCTCAGTTCTGCTAAGTGCGCGTACCGAGACGAATCTGACATATTGTAC
AGCGACGACTCCAGATCCGATTTAGCAAGCAACGGGGCACCCACCGACAGCTACGAATCT
TTAACAGCAAGTAGTGAGTCTCTAGCAGAAAGCAACGATGCACCCAGCAACAGCTATGAA
TCTTTTCCAGAAATTAGAGAAAATCTAACCGCAAGTGAGGAATCCCTAACATCATGTGAG
GAATCCCTAACAGGAAGTAATGAATCCCTAACAGGAAGTAATGAATCCCTAACAGGAAGT
AAT---------------------------------------------------------
------GAATCCCTAACAGGAAGTAATGAATCCCTAACAGGAAGTAATGAATCCCTAACA
GAAAGTAGAGAATCTCTAGAGGCCAGTAGAGAATCGCTAAGAGCAAGTAGAGAGTCTCTA
GCGGCCAGTAGAGAATCCCTGAACGACTTTTGTGGGAGCGAAGAATCAGTAGCATTCGAA
GGAGAGCCAAATGAAAAGACATTCATGGGAGACGTCTTAAGTGGTGGAGAATGTGAGAAT
AGTCTCTCAAGAGAAGATTTATTTCATATAGAAGTAGGATCCGAAGAATCGCTAGATGAT
GCCTCAAAATATAATTTCCAAAAGGATTTATCAACTAGCGATAATAGTTCATTCGAAGAT
GATCAGTCATTGAAAAGGGGACTCAAAAGGAACAGCTCAGTATCTAGTCTGGACAGCGAT
ATGGGAAGTTATAAAAATAAAAGTTATAGACACAGATTGGATATATATTCGGACCTACCT
AGAAGGCCAATACATGGGGATAATGCACCACAAGAAAAGGAGAAATGTCTGGATTTCAAA
GAACTTCTAGAGAGAGAAAGGAACAACCACCAAAGGAATAGTGTAAATATAAAATTAAAA
AATATATACGGAGAAGAACAATTGTATAATGTGAAGAAAGATAGCTTTATTGTAAAATTG
ATATACTATCTGCCGTTTGGCCCAATTGTTACAATGATAATGATAGGCTTTATTCTACTC
TGCTCAACGCCCTCGACTGGTTTTATTTTTATAGGGTGTAGCTCTATTATATTACCAGCT
TTGTTATACGCGATGCATTCAAACAAAAAACAATTCGAACGTGTATTTGGAAAAGGGAGA
AAAAAGCCAATGAAGGTAAAAAAAGGAGGCGGGAAAATATCTAGATTTTTTAAAGAAGTA
GACAAATTCTTGTTTGACGATTTGGATGGTTAT
>Columbia_30102100488 .
ATGAAAGGAATAATGAATGGTTCCCTTTGTTTAAGGAAAATTTCCCTGTGTTCTATTTTC
GTATTGGCATATCTCAGTTCTGCTAAGTGCGCGTACCGAGACGAATCTGACATATTGTAC
AGCGACGACTCCAGATCCGATTTAGCAAGCAACGGGGCACCCACCGACAGCTACGAATCT
TTAACAGCAAGTAGTGAGTCTCTAGCAGAAAGCAACGATGCACCCAGCAACAGCTATGAA
TCTTTTCCAGAAATTAGAGAAAATCTAACCGCAAGTGAGGAATCCCTAACATCATGTGAG
GAATCCCTAACAGGAAGTAATGAATCCCTAACAGGAAGTAATGAATCCCTAACAGGAAGT
AAT---------------------------------------------------------
------GAATCCCTAACAGGAAGTAATGAATCCCTAACAGGAAGTAATGAATCCCTAACA
GAAAGTAGAGAATCTCTAGAGGCCAGTAGAGAATCGCTAAGAGCAAGTAGAGAGTCTCTA
GCGGCCAGTAGAGAATCCCTGAACGACTTTTGTGGGAGCGAAGAATCAGTAGCATTCGAA
GGAGAGCCAAATGAAAAGACATTCATGGGAGACGTCTTAAGTGGTGGAGAATGTGAGAAT
AGTCTCTCAAGAGAAGATTTATTTCATATAGAAGTAGGATCCGAAGAATCGCTAGATGAT
GCCTCAAAATATAATTTCCAAAAGGATTTATCAACTAGCGATAATAGTTTATTCGAAGAT
GATCAGTCATTGAAAAGGGGACTCAAAAGGAACAGCTCAGTATCTAGTCTGGACAGCGAT
ATGGGAAGTTATAAAAATGGAAGATCTAGAGACAGATTGGATATATATTCGGACCTACCT
AGAAGGCTAATACATGGGGATAATGCACCACAAGAAAAGGAGAAATGTCTGGATTTCAAA
GAACTTCTAGAGAGAGAAAGGAACAACCACCAAAGGAATAGTGTAAATATAAAATTAAAA
AATATATACGGAGAAGAACAATTGTATAATGTGAAGAAAGATAGCTTTATTGTAAAATTG
ATATACTATCTGCCGTTTGGCCCAATTGTTACAATGATAATGATAGGCTTTATTCTACTC

```
TGGTCAACGCCCTGGACTGGTTTTATTTTTATAGGGTGTAGCTCTATTATATTACCAGCT
TTGTTATACGCGATGCATTCAAACAAAAAACAATTCGAACGTGTATTTGGAAAAGGGAGA
AAAAAGCCAATGAAGGTAAAAAAAGGAGGCGGGAAAATATCTAGATTTTTTAAAGAAGTA
GACAAATTCTTGTTTGACGATTTGGATGGTTAT
>Columbia_30102100440 .
ATGAAAGGAATAATGAATGGTTCCCTTTGTTTAAGGAAAATTTCCCTGTGTTCTATTTTC
GTATTGGCATATCTCAGTTCTGCTAAGTGCGCGTACCGAGACGAATCTGACATATTGTAC
AGCGACGACTCCAGATCCGATTTAGCAAGCAACGGGGCACCCACCGACAGCTACGAATCT
TTAACAGCAAGTAGTGAGTCTCTAGCAGAAAGCAACGATGCACCCAGCAACAGCTATGAA
TCTTTTCCAGAAATTAGAGAAAATCTAACCGCAAGTGAGGAATCCCTAACATCATGTGAG
GAATCCCTAACAGGAAGTAATGAATCCCTAACAGGAAGTAATGAATCCCTAACAGGAAGT
AAT---------------------------------------------------------
------GAATCCCTAACAGGAAGTAATGAATCCCTAACAGGAAGTAATGAATCCCTAACA
GAAAGTAGAGAATCTCTAGAGGCCAGTAGAGAATCGCTAAGAGCAAGTAGAGAGTCTCTA
GCGGCCAGTAGAGAATCCCTGAACGACTTTTGTGGGAGCGAAGAATCAGTAGCATTCGAA
GGAGAGCCAAATGAAAAGACATTCATGGGAGACGTCTTAAGTGGTGGAGAATGTGAGAAT
AGTCTCTCAAGAGAAGATTTATTTCATATAGAAGTAGGATCCGAAGAATCGCTAGATGAT
GCCTCAAAATATAATTTCCAAAAGGATTTATCAACTAGCGATAATAGTTTATTCGAAGAT
GATCAGTCATTGAAAAGGGGACTCAAAAGGAACAGCTCAGTATCTAGTCTGGACAGCGAT
ATGGGAAGTTATAAAAATGGAAGATCTAGAGACAGATTGGATATATATTCGGACCTACCT
AGAAGGCTAATACATGGGGATAATGCACCACAAGAAAAGGAGAAATGTCTGGATTTCAAA
GAACTTCTAGAGAGAGAAAGGAACAACCACCAAAGGAATAGTGTAAATATAAAATTAAAA
AATATATACGGAGAAGAACAATTGTATAATGTGAAGAAAGATAGCTTTATTGTAAAATTG
ATATACTATCTGCCGTTTGGCCCAATTGTTACAATGATAATGATAGGCTTTATTCTACTC
TGGTCAACGCCCTGGACTGGTTTTATTTTTATAGGGTGTAGCTCTATTATATTACCAGCT
TTGTTATACGCGATGCATTCAAACAAAAAACAATTCGAACGTGTATTTGGAAAAGGGAGA
AAAAAGCCAATGAAGGTAAAAAAAGGAGGCGGGAAAATATCTAGATTTTTTAAAGAAGTA
GACAAATTCTTGTTTGACGATTTGGATGGTTAT
>Columbia_30102100448 .
ATGAAAGGAATAATGAATGGTTCCCTTTGTTTAAGGAAAATTTCCCTGTGTTCTATTTTC
GTATTGGCATATCTCAGTTCTGCTAAGTGCGCGTACCGAGACGAATCTGACATATTGTAC
AGCGACGACTCCAGATCCGATTTAGCAAGCAACGGGGCACCCACCGACAGCTACGAATCT
TTAACAGCAAGTAGTGAGTCTCTAGCAGAAAGCAACGATGCACCCAGCAACAGCTATGAA
TCTTTTCCAGAAATTAGAGAAAATCTAACCGCAAGTGAGGAATCCCTAACATCATGTGAG
GAATCCCTAACAGGAAGTAATGAATCCCTAACAGGAAGTAATGAATCCCTAACAGGAAGT
AAT---------------------------------------------------------
------GAATCCCTAACAGGAAGTAATGAATCCCTAACAGGAAGTAATGAATCCCTAACA
GAAAGTAGAGAATCTCTAGAGGCCAGTAGAGAATCGCTAAGAGCAAGTAGAGAGTCTCTA
GCGGCCAGTAGAGAATCCCTGAACGACTTTTGTGGGAGCGAAGAATCAGTAGCATTCGAA
GGAGAGCCAAATGAAAAGACATTCATGGGAGACGTCTTAAGTGGTGGAGAATGTGAGAAT
AGTCTCTCAAGAGAAGATTTATTTCATATAGAAGTAGGATCCGAAGAATCGCTAGATGAT
GCCTCAAAATATAATTTCCAAAAGGATTTATCAACTAGCGATAATAGTTTATTCGAAGAT
GATCAGTCATTGAAAAGGGGACTCAAAAGGAACAGCTCAGTATCTAGTCTGGACAGCGAT
ATGGGAAGTTATAAAAATGGAAGATCTAGAGACAGATTGGATATATATTCGGACCTACCT
AGAAGGCTAATACATGGGGATAATGCACCACAAGAAAAGGAGAAATGTCTGGATTTCAAA
GAACTTCTAGAGAGAGAAAGGAACAACCACCAAAGGAATAGTGTAAATATAAAATTAAAA
AATATATACGGAGAAGAACAATTGTATAATGTGAAGAAAGATAGCTTTATTGTAAAATTG
ATATACTATCTGCCGTTTGGCCCAATTGTTACAATGATAATTATAGGCTTTATTCTACTC
TGGTCAACGCCCTGGACTGGTTTTATTTTTATAGGGTGTAGCTCTATTATATTACCAGCT
TTGTTATACGCGATGCATTCAAACAAAAAACAATTCGAACGTGTATTTGGAAAAGGGAGA
```

```
AAAAAGCCAATGAAGGTAAAAAAAGGAGGCGGGAAAATATCTAGATTTTTTAAAGAAGTA
GACAAATTCTTGTTTGACGATTTGGATGGTTAT
>Columbia_30102100485 .
ATGAAAGGAATAATGAATGGTTCCCTTTGTTTAAGGAAAATTTCCCTGTGTTCTATTTTC
GTATTGGCATATCTCAGTTCTGCTAAGTGCGCGTACCGAGACGAATCTGACATATTGTAC
AGCGACGACTCCAGATCCGATTTAGCAAGCAACGGGGCACCCACCGACAGCTACGAATCT
TTAACAGCAAGTAGTGAGTCTCTAGCAGAAAGCAACGATGCACCCAGCAACAGCTATGAA
TCTTTTCCAGAAATTAGAGAAAATCTAACCGCAAGTGAGGAATCCCTAACATCATGTGAG
GAATCCCTAACAGGAAGTAATGAATCCCTAACAGGAAGTAATGAATCCCTAACAGGAAGT
AAT---------------------------------------------------------
------GAATCCCTAACAGGAAGTAATGAATCCCTAACAGGAAGTAATGAATCCCTAACA
GAAAGTAGAGAATCTCTAGAGGCCAGTAGAGAATCGCTAAGAGCAAGTAGAGAGTCTCTA
GCGGCCAGTAGAGAATCCCTGAACGACTTTTGTGGGAGCGAAGAATCAGTAGCATTCGAA
GGAGAGCCAAATGAAAAGACATTCATGGGAGACGTCTTAAGTGGTGGAGAATGTGAGAAT
AGTCTCTCAAGAGAAGATTTATTTCATATAGAAGTAGGATCCGAAGAATCGCTAGATGAT
GCCTCAAAATATAATTTCCAAAAGGATTTATCAACTAGCGATAATAGTTTATTCGAAGAT
GATCAGTCATTGAAAAGGGGACTCAAAAGGAACAGCTCAGTATCTAGTCTGGACAGCGAT
ATGGGAAGTTATAAAAATGGAAGATCTAGAGACAGATTGGATATATATTCGGACCTACCT
AGAAGGCTAATACATGGGGATAATGCACCACAAGAAAAGGAGAAATGTCTGGATTTCAAA
GAACTTCTAGAGAGAGAAAGGAACAACCACCAAAGGAATAGTGTAAATATAAAATTAAAA
AATATATACGGAGAAGAACAATTGTATAATGTGAAGAAAGATAGCTTTATTGTAAAATTG
ATATACTATCTGCCGTTTGGCCCAATTGTTACAATGATAATTATAGGCTTTATTCTACTC
TGGTCAACGCCCTGGACTGGTTTTATTTTTATAGGGTGTAGCTCTATTATATTACCAGCT
TTGTTATACGCGATGCATTCAAACAAAAAACAATTCGAACGTGTATTTGGAAAAGGGAGA
AAAAAGCCAATGAAGGTAAAAAAAGGAGGCGGGAAAATATCTAGATTTTTTAAAGAAGTA
GACAAATTCTTGTTTGACGATTTGGATGGTTAT
>Columbia_30102100445 .
ATGAAAGGAATAATGAATGGTTCCCTTTGTTTAAGGAAAATTTCCCTGTGTTCTATTTTC
GTATTGGCATATCTCAGTTCTGCTAAGTGCGCGTACCGAGACGAATCTGACATATTGTAC
AGCGACGACTCCAGATCCGATTTAGCAAGCAACGGGGCACCCACCGACAGCTACGAATCT
TTAACAGCAAGTAGTGAGTCTCTAGCAGAAAGCAACGATGCACCCAGCAACAGCTATGAA
TCTTTTCCAGAAATTAGAGAAAATCTAACCGCAAGTGAGGAATCCCTAACATCATGTGAG
GAATCCCTAACAGGAAGTAATGAATCCCTAACAGGAAGTAATGAATCCCTAACAGGAAGT
AAT---------------------------------------------------------
------GAATCCCTAACAGGAAGTAATGAATCCCTAACAGGAAGTAATGAATCCCTAACA
GAAAGTAGAGAATCTCTAGAGGCCAGTAGAGAATCGCTAAGAGCAAGTAGAGAGTCTCTA
GCGGCCAGTAGAGAATCCCTGAACGACTTTTGTGGGAGCGAAGAATCAGTAGCATTCGAA
GGAGAGCCAAATGAAAAGACATTCATGGGAGACGTCTTAAGTGGTGGAGAATGTGAGAAT
AGTCTCTCAAGAGAAGATTTATTTCATATAGAAGTAGGATCCGAAGAATCGCTAGATGAT
GCCTCAAAATATAATTTCCAAAAGGATTTATCAACTAGCGATAATAGTTTATTCGAAGAT
GATCAGTCATTGAAAAGGGGACTCAAAAGGAACAGCTCAGTATCTAGTCTGGACAGCGAT
ATGGGAAGTTATAAAAATGGAAGATCTAGAGACAGATTGGATATATATTCGGACCTACCT
AGAAGGCTAATACATGGGGATAATGCACCACAAGAAAAGGAGAAATGTCTGGATTTCAAA
GAACTTCTAGAGAGAGAAAGGAACAACCACCAAAGGAATAGTGTAAATATAAAATTAAAA
AATATATACGGAGAAGAACAATTGTATAATGTGAAGAAAGATAGCTTTATTGTAAAATTG
ATATACTATCTGCCGTTTGGCCCAATTGTTACAATGATAATTATAGGCTTTATTCTACTC
```

TGGTCAACGCCCTGGACTGGTTTTATTTTTATAGGGTGTAGCTCTATTATATTACCAGCT
TTGTTATACGCGATGCATTCAAACAAAAAACAATTCGAACGTGTATTTGGAAAAGGGAGA
AAAAAGCCAATGAAGGTAAAAAAAGGAGGCGGGAAAATATCTAGATTTTTTAAAGAAGTA
GACAAATTCTTGTTTGACGATTTGGATGGTTAT
>Columbia_30102100438B .
ATGAAAGGAATAATGAATGGTTCCCTTTATTTAAGGAAAATTTCCCTGTGTTCTATTTTC
GTATTGGCATATCTCAGTTCTGCTAAGTGCGCGTACCGAGACGAATCTGACATATTGTAC
AGCGACGACTCCAGATCCGATTTAGCAAGCAACGGGGCACCCACCGACAGCTACGAATCT
TTAACAGCAAGTAGTGAGTCTCTAGCAGAAAGCAACGATGCACCCAGCAACAGCTATGAA
TCTTTTCCAGAAATTAGAGAAAATCTAACCGCAAGTGAGGAATCCCTAACATCATGTGAG
GAATCCCTAACAGGAAGTAACGAATCCCTAACAGGAAGTAATGAATCCCTAACAGGAAGT
AAT---------------------------------------------------------
------GAATCCCTAACAGGAAGTAATGAATCCCTAACAGGAAGTAATGAATCCCTAACA
GAAAGTAGAGAATCTCTAGAGGCCAGTAGAGAATCGCTAAGAGCAAGTAGAGAGTCTCTA
GCGGCCAGTAGAGAATCCCTGAACGACTTTTGTGGGAGCGAAGAATCAGTAGCATTCGAA
GGAGAGCCAAATGAAAAGACATTCATGGGAGACGTCTTAAGTGGTGGAGAATGTGAGAAT
AGTCTCTCAAGAGAAGATTTATTTCATATAGAAGTAGGATCCGAAGAATCGCTAGATGAT
GCCTCAAAATATAATTTCCAAAAGGATTTATCAACTAGCGATAATAGTTCATTCGAAGAT
GATCAGTCATTGAAAAGGGGACTCAAAAGGAACAGCTCAGTATCTAGTCTGGACAGCGAT
ATGGGAAGTTATAAAAATGGAAGATCTAGAGACAGATTGGATATATATTCGGACCTACCT
AGAAGGCTAATACATGGGGATAATGCACCACAAGAAAAGGAGAAATGTCTGGATTTCAAA
GAACTTCTAGAGAGAGAAAGGAACAACCACCAAAGGAATAGTGTAAATATAAAATTAAAA
AATATATACGGAGAAGAACAATTGTATAATGTGAAGAAAGATAGCTTTATTGTAAAATTG
ATATACTATCTGCCGTTTGGCCCAATTGTTACAATGATAATGATAGGCTTTATTCTACTC
TGCTCAACGCCCTCGACTGGTTTTATTTTTATAGGGTGTAGCTCTATTATATTACCAGCT
TTGTTATACGCGATGCATTCAAACAAAAAACAATTCGAACGTGTATTTGGAAAAGGGAGA
AAAAAGCCAATGAAGGTAAAAAAAGGAGGCGGGAAAATATCTAGATTTTTTAAAGAAGTA
GACAAATTCTTGTTTGACGATTTGGATGGTTAT
>Columbia_30102100438A .
ATGAAAGGAATAATGAATGGTTCCCTTTATTTAAGGAAAATTTCCCTGTGTTCTATTTTC
GTATTGGCATATCTCAGTTCTGCTAAGTGCGCGTACCGAGACGAATCTGACATATTGTAC
AGCGACGACTCCAGATCCGATTTAGCAAGCAACGGGGCACCCACCGACAGCTACGAATCT
TTAACAGCAAGTAGTGAGTCTCTAGCAGAAAGCAACGATGCACCCAGCAACAGCTATGAA
TCTTTTCCAGAAATTAGAGAAAATCTAACCGCAAGTGAGGAATCCCTAACATCATGTGAG
GAATCCCTAACAGGAAGTAACGAATCCCTAACAGGAAGTAATGAATCCCTAACAGGAAGT
AAT---------------------------------------------------------
------GAATCCCTAACAGGAAGTAATGAATCCCTAACAGGAAGTAATGAATCCCTAACA
GAAAGTAGAGAATCTCTAGAGGCCAGTAGAGAATCGCTAAGAGCAAGTAGAGAGTCTCTA
GCGGCCAGTAGAGAATCCCTGAACGACTTTTGTGGGAGCGAAGAATCAGTAGCATTCGAA
GGAGAGCCAAATGAAAAGACATTCATGGGAGACGTCTTAAGTGGTGGAGAATGTGAGAAT
AGTCTCTCAAGAGAAGATTTATTTCATATAGAAGTAGGATCCGAAGAATCGCTAGATGAT
GCCTCAAAATATAATTTCCAAAAGGATTTATCAACTAGCGATAATAGTTCATTCGAAGAT
GATCAGTCATTGAAAAGGGGACTCAAAAGGAACAGCTCAGTATCTAGTCTGGACAGCGAT
ATGGGAAGTTATAAAAATGGAAGATCTAGAGACAGATTGGATATATATTCGGACCTACCT
AGAAGGCTAATACATGGGGATAATGCACCACAAGAAAAGGAGAAATGTCTGGATTTCAAA
GAACTTCTAGAGAGAGAAAGGAACAACCACCAAAGGAATAGTGTAAATATAAAATTAAAA
AATATATACGGAGAAGAACAATTGTATAATGTGAAGAAAGATAGCTTTATTGTAAAATTG
ATATACTATCTGCCGTTTGGCCCAATTGTTACAATGATAATGATAGGCTTTATTCTACTC
TGCTCAACGCCCTCGACTGGTTTTATTTTTATAGGGTGTAGCTCTATTATATTACCAGCT
TTGTTATACGCGATGCATTCAAACAAAAAACAATTCGAACGTGTATTTGGAAAAGGGAGA

```
AAAAAGCCAATGAAGGTAAAAAAAGGAGGCGGGAAAATATCTAGATTTTTTAAAGAAGTA
GACAAATTCTTGTTTGACGATTTGGATGGTTAT
>Columbia_30102100446 .
ATGAAAGGAATAATGAATGGTTCCCTTTGTTTAAGGAAAATTTCCCTGTGTTCTATTTTC
GTATTGGCATATCTCAGTTCTGCTAAGTGCGCGTACCGAGACGAATCTGACATATTGTAC
AGCGACGACTCCAGATCCGATTTAGCAAGCAACGGGGCACCCACCGACAGCTACGAATCT
TTAACAGCAAGTAGTGAGTCTCTAGCAGAAAGCAACGATGCACCCAGCAACAGCTATGAA
TCTTTTCCAGAAATTAGAGAAAATCTAACCGCAAGTGAGGAATCCCTAACATCATGTGAG
GAATCCCTAACAGGAAGTAATGAATCCCTAACAGGAAGTAATGAATCCCTAACAGGAAGT
AAT---------------------------------------------------------
------GAATCCCTAACAGGAAGTAATGAATCCCTAACAGGAAGTAATGAATCCCTAACA
GAAAGTAGAGAATCTCTAGAGGCCAGTAGAGAATCGCTAAGAGCAAGTAGAGAGTCTCTA
GCGGCCAGTAGAGAATCCCTGAACGACTTTTGTGGGAGCGAAGAATCAGTAGCATGCGAA
GGAGAGCCAAATGAAAAGACATTCATGGGAGACGTCTTAAGTGGTGGAGAATGTGAGAAT
AGTCTCTCAAGAGAAGATTTATTTCATATAGAAGTAGGATCCGAAGAATCGCTAGATGAT
GCCTCAAAATATAATTTCCAAAAGGATTTATCAACTAGCGATAATAGTTCATTCGAAGAT
GATCAGTCATTGAAAAGGGGACTCAAAAGGAACAGCTCAGTATCTAGTCTGGACAGCGAT
ATGGGAAGTTATAAAAATGGAAGATCTAGAGACAGATTGGATATATATTCGGACCTACCT
AGAAGGCTAATACATGGGGATAATGCACCACAAGAAAAGGAGAAATGTCTGGATTTCAAA
GAACTTCTAGAGAGAGAAAGGAACAACCACCAAAGGAATAGTGTAAATATAAAATTAAAA
AATATATACGGAGAAGAACAATTGTATAATGTGAAGAAAGATAGCTTTATTGTAAAATTG
ATATACTATCTGCCGTTTGGCCCAATTGTTACAATGATAATGATAGGCTTTATTCTACTC
TGCTCAACGCCCTCGACTGGTTTTATTTTTATAGGGTGTAGCTCTATTATATTACCAGCT
TTGTTATACGCGATGCATTCAAACAAAAAACAATTCGAACGTGTATTTGGAAAAGGGAGA
AAAAAGCCAATGAAGGTAAAAAAAGGAGGCGGGAAAATATCTAGATTTTTTAAAGAAGTA
GACAAATTCTTGTTTGACGATTTGGATGGTTAT
>Columbia_30103103280 .
ATGAAAGGAATAATGAATGGTTCCCTTTGTTTAAGGAAAATTTCCCTGTGTTCTATTTTC
GTATTGGCATATCTCAGTTCTGCTAAGTGCGCGTACCGAGACGAATCTGACATATTGTAC
AGCGACGACTCCAGATCCGATTTAGCAAGCAACGGGGCACCCACCGACAGCTACGAATCT
TTAACAGCAAGTAGTGAGTCTCTAGCAGAAAGCAACGATGCACCCAGCAACAGCTATGAA
TCTTTTCCAGAAATTAGAGAAAATCTAACCGCAAGTGAGGAATCCCTAACATCATGTGAG
GAATCCCTAACAGGAAGTAATGAATCCCTAACAGGAAGTAATGAATCCCTAACAGGAAGT
AAT---------------------------------------------------------
------GAATCCCTAACAGGAAGTAATGAATCCCTAACAGGAAGTAATGAATCCCTAACA
GAAAGTAGAGAATCTCTAGAGGCCAGTAGAGAATCGCTAAGAGCAAGTAGAGAGTCTCTA
GCGGCCAGTAGAGAATCCCTGAACGACTTTTGTGGGAGCGAAGAATCAGTAGCATGCGAA
GGAGAGCCAAATGAAAAGACATTCATGGGAGACGTCTTAAGTGGTGGAGAATGTGAGAAT
AGTCTCTCAAGAGAAGATTTATTTCATATAGAAGTAGGATCCGAAGAATCGCTAGATGAT
GCCTCAAAATATAATTTCCAAAAGGATTTATCAACTAGCGATAATAGTTCATTCGAAGAT
GATCAGTCATTGAAAAGGGGACTCAAAAGGAACAGCTCAGTATCTAGTCTGGACAGCGAT
ATGGGAAGTTATAAAAATGGAAGATCTAGAGACAGATTGGATATATATTCGGACCTACCT
AGAAGGCTAATACATGGGGATAATGCACCACAAGAAAAGGAGAAATGTCTGGATTTCAAA
GAACTTCTAGAGAGAGAAAGGAACAACCACCAAAGGAATAGTGTAAATATAAAATTAAAA
AATATATACGGAGAAGAACAATTGTATAATGTGAAGAAAGATAGCTTTATTGTAAAATTG
ATATACTATCTGCCGTTTGGCCCAATTGTTACAATGATAATGATAGGCTTTATTCTACTC
```

TGCTCAACGCCCTCGACTGGTTTTATTTTTATAGGGTGTAGCTCTATTATATTACCAGCT
TTGTTATACGCGATGCATTCAAACAAAAAACAATTCGAACGTGTATTTGGAAAAGGGAGA
AAAAAGCCAATGAAGGTAAAAAAAGGAGGCGGGAAAATATCTAGATTTTTTAAAGAAGTA
GACAAATTCTTGTTTGACGATTTGGATGGTTAT
>Columbia_30111110026 .
ATGAAAGGAATAATGAATGGTTCCCTTTATTTAAGGAAAATTTCCCTGTGTTCTATTTTC
GTATTGGCATATCTCAGTTCTGCTAAGTGCGCGTACCGAGACGAATCTGACATATTGTAC
AGCGACGACTCCAGATCCGATTTAGCAAGCAACGGGGCACCCACCGACAGCTACGAATCT
TTAACAGCAAGTAGTGAGTCTCTAGCAGAAAGCAACGATGCACCCAGCAACAGCTATGAA
TCTTTTCCAGAAATTAGAGAAAATCTAACCGCAAGTGAGGAATCCCTAACATCATGTGAG
GAATCCCTAACAGGAAGTAACGAATCCCTAACAGGAAGTAATGAATCCCTAACAGGAAGT
AAT---------------------------------------------------------
------GAATCCCTAACAGGAAGTAATGAATCCCTAACAGGAAGTAATGAATCCCTAACA
GAAAGTAGAGAATCTCTAGAGGCCAGTAGAGAATCGCTAAGAGCAAGTAGAGAGTCTCTA
GCGGCCAGTAGAGAATCCCTGAACGACTTTTGTGGGAGCGAAGAATCAGTAGCATTCGAA
GGAGAGCCAAATGAAAAGACATTCATGGGAGACGTCTTAAGTGGTGGAGAATGTGAGAAT
AGTCTCTCAAGAGAAGATTTATTTCATATAGAAGTAGGATCCGAAGAATCGCTAGATGAT
GCCTCAAAATATAATTTCCAAAAGGATTTATCAACTAGCGATAATAGTTCATTCGAAGAT
GATCAGTCATTGAAAAGGGGACTCAAAAGGAACAGCTCAGTATCTAGTCTGGACAGCGAT
ATGGGAAGTTATAAAAATGGAAGATCTAGAGACAGATTGGATATATATTCGGACCTACCT
AGAAGGCTAATACATGGGGATAATGCACCACAAGAAAAGGAGAAATGTCTGGATTTCAAA
GAACTTCTAGAGAGAGAAAGGAACAACCACCAAAGGAATAGTGTAAATATAAAATTAAAA
AATATATACGGAGAAGAACAATTGTATAATGTGAAGAAAGATAGCTTTATTGTAAAATTG
ATATACTATCTGCCGTTTGGCCCAATTGTTACAATGATAATGATAGGCTTTATTCTACTC
TGCTCAACGCCCTCGACTGGTTTTATTTTTATAGGGTGTAGCTCTATTATATTACCAGCT
TTGTTATACGCGATGCATTCAAACAAAAAACAATTCGAACGTGTATTTGGAAAAGGGAGA
AAAAAGCCAATGAAGGTAAAAAAAGGAGGCGGGAAAATATCTAGATTTTTTAAAGAAGTA
GACAAATTCTTGTTTGACGATTTGGATGGTTAT
>Columbia_30111110020 .
ATGAAAGGAATAATGAATGGTTCCCTTTATTTAAGGAAAATTTCCCTGTGTTCTATTTTC
GTATTGGCATATCTCAGTTCTGCTAAGTGCGCGTACCGAGACGAATCTGACATATTGTAC
AGCGACGACTCCAGATCCGATTTAGCAAGCAACGGGGCACCCACCGACAGCTACGAATCT
TTAACAGCAAGTAGTGAGTCTCTAGCAGAAAGCAACGATGCACCCAGCAACAGCTATGAA
TCTTTTCCAGAAATTAGAGAAAATCTAACCGCAAGTGAGGAATCCCTAACATCATGTGAG
GAATCCCTAACAGGAAGTAATGAATCCCTAACAGGAAGTAATGAATCCCTAACAGGAAGT
AAT---------------------------------------------------------
------GAATCCCTAACAGGAAGTAATGAATCCCTAACAGGAAGTAATGAATCCCTAACA
GAAAGTAGAGAATCTCTAGAGGCCAGTAGAGAATCGCTAAGAGCAAGTAGAGAGTCTCTA
GCGGCCAGTAGAGAATCCCTGAACGACTTTTGTGGGAGCGAAGAATCAGTAGCATTCGAA
GGAGAGCCAAATGAAAAGACATTCATGGGAGACGTCTTAAGTGGTGGAGAATGTGAGAAT
AGTCTCTCAAGAGAAGATTTATTTCATATAGAAGTAGGATCCGAAGAATCGCTAGATGAT
GCCTCAAAATATAATTTCCAAAAGGATTTATCAACTAGCGATAATAGTTCATTCGAAGAT
GATCAGTCATTGAAAAGGGGACTCAAAAGGAACAGCTCAGTATCTAGTCTGGACAGCGAT
ATGGGAAGTTATAAAAATAAAAGTTATAGACACAGATTGGATATATATTCGGACCTACCT
AGAAGGCCAATACATGGGGATAATGCACCACAAGAAAAGGAGAAATGTCTGGATTTCAAA
GAACTTCTAGAGAGAGAAAGGAACAACCACCAAAGGAATAGTGTAAATATAAAATTAAAA
AATATATACGGAGAAGAACAATTGTATAATGTGAAGAAAGATAGCTTTATTGTAAAATTG
ATATACTATCTGCCGTTTGGCCCAATTGTTACAATGATAATGATAGGTTTTATTCTACTC
TGGTCAACGCCCTGGACTGGTTTTATTTTTATAGGGTGTAGCTCTATTATATTACCAGCT
TTGTTATACGCGATGCATTCAAACAAAAAACAATTCGAACGTGTATTTGGAAAAGGGAGA

```
AAAAAGCCAATGAAGGTAAAAAAAGGAGGCGGGAAAATATCTAGATTTTTTAAAGAAGTA
GACAAATTCTTGTTTGACGATTTGGATGGTTAT
>Columbia_30111110015 .
ATGAAAGGAATAATGAATGGTTCCCTTTGTTTAAGGAAAATTTCCCTGTGTTCTATTTTC
GTATTGGCATATCTCAGTTCTGCTAAGTGCGCGTACCGAGACGAATCTGACATATTGTAC
AGCGACGACTCCAGATCCGATTTAGCAAGCAACGGGGCACCCACCGACAGCTACGAATCT
TTAACAGCAAGTAGTGAGTCTCTAGCAGAAAGCAACGATGCACCCAGCAACAGCTATGAA
TCTTTTCCAGAAATTAGAGAAAATCTAACCGCAAGTGAGGAATCCCTAACATCATGTGAG
GAATCCCTAACAGGAAGTAATGAATCCCTAACAGGAAGTAATGAATCCCTAACAGGAAGT
AAT---------------------------------------------------------
------GAATCCCTAACAGGAAGTAATGAATCCCTAACAGGAAGTAATGAATCCCTAACA
GAAAGTAGAGAATCTCTAGAGGCCAGTAGAGAATCGCTAAGAGCAAGTAGAGAGTCTCTA
GCGGCCAGTAGAGAATCCCTGAACGACTTTTGTGGGAGCGAAGAATCAGTAGCATTCGAA
GGAGAGCCAAATGAAAAGACATTCATGGGAGACGTCTTAAGTGGTGGAGAATGTGAGAAT
AGTCTCTCAAGAGAAGATTTATTTCATATAGAAGTAGGATCCGAAGAATCGCTAGATGAT
GCCTCAAAATATAATTTCCAAAAGGATTTATCAACTAGCGATAATAGTTTATTCGAAGAT
GATCAGTCATTGAAAAGGGGACTCAAAAGGAACAGCTCAGTATCTAGTCTGGACAGCGAT
ATGGGAAGTTATAAAAATGGAAGATCTAGAGACAGATTGGATATATATTCGGACCTACCT
AGAAGGCTAATACATGGGGATAATGCACCACAAGAAAAGGAGAAATGTCTGGATTTCAAA
GAACTTCTAGAGAGAGAAAGGAACAACCACCAAAGGAATAGTGTAAATATAAAATTAAAA
AATATATACGGAGAAGAACAATTGTATAATGTGAAGAAAGATAGCTTTATTGTAAAATTG
ATATACTATCTGCCGTTTGGCCCAATTGTTACAATGATAATGATAGGCTTTATTCTACTC
TGGTCAACGCCCTGGACTGGTTTTATTTTTATAGGGTGTAGCTCTATTATATTACCAGCT
TTGTTATACGCGATGCATTCAAACAAAAAACAATTCGAACGTGTATTTGGAAAAGGGAGA
AAAAAGCCAATGAAGGTAAAAAAAGGAGGCGGGAAAATATCTAGATTTTTTAAAGAAGTA
GACAAATTCTTGTTTGACGATTTGGATGGTTAT
>CH144 .
------------------------------TTAAGGAAAATTTCCCTGTGTTCTATTTTC
GTATTGGCATATCTCAGTTCTGCTAAGTGCGCGTACCGAGACGAATCTGACATATTGTAC
AGCGACGACTCCAGATCCGATTTAGCAAGCAACGGGGCACCCACCGACAGCTACGAATCT
TTAACAGCAAGTAGTGAGTCTCTAGCAGAAAGCAACGATGCACCCAGCAACAGCTATGAA
TCTTTTCCAGAAATTAGAGAAAATCTAACCGCAAGTGAGGAATCCCTAACATCATGTGAG
GAATCCCTAACAGGAAGTAATGAATCCCTAACAGGAAGTAATGAATCCCTAACAGGAAGT
AAT---------------------------------------------------------
---------------------------GAATCCCTAACAGGAAGTAATGAATCCCTAACA
GAAAGTAGAGAATCTCTAGAGGCCAGTAGAGAATCGCTAAGAGCAAGTAGAGAGTCTCTA
GCGGCCAGTAGAGAATCCCTGAACGACTTTTGTGGGAGCGAAGAATCAGTAGCATGCGAA
GGAGAGCCAAATGAAAAGACATTCATGGGAGACGTCTTAAGTGGTGGAGAATGTGAGAAT
AGTCTCTCAAGAGAAGATTTATTTCATATAGAAGTAGGATCCGAAGAATCGCTAGATGAT
GCCTCAAAATATAATTTCCAAAAGGATTTATCAACTAGCGATAATAGTTCATTCGAAGAT
GATCAGTCATTGAAAAGGGGACTCAAAAGGAACAGCTCAGTATCTAGTCTGGACAGCGAT
ATGGGAAGTTATAAAAATGGAAGATCTAGAGACAGATTGGATATATATTCGGACCTACCT
AGAAGGCTAATACATGGGGATAATGCACCACAAGAAAAGGAGAAATGTCTGGATTTCAAA
GAACTTCTAGAGAGAGAAAGGAACAACCACCAAAGGAATAGTGTAAATATAAAATTAAAA
AATATATACGGAGAAGAACAATTGTATAATGTGAAGAAAGATAGCTTTATTGTAAAATTG
ATATACTATCTGCCGTTTGGCCCAATTGTTACAATGATAATGATAGGCTTTATTCTACTC
```

TGCTCAACGCCCTCGACTGGTTTTATTTTTATAGGGTGTAGCTCTATTATATTACCAGCT
TTGTTATACGCGATGCATTCAAACAAAAAACAATTCGAACGTGTATTTGGAAAAGGGAGA
AAAAAGCCAATGAAGGTAAAAAAAGGAGGCGGGAAAATATCTAGATTTTTTAAAGAAGTA
GACAAA---------------------------
>CH147 .
------------------------------TTAAGGAAAATTTCCCTGTGTTCTATTTTC
GTATTGGCATATCTCAGTTCTGCTAAGTGCGCGTACCGAGACGAATCTGACATATTGTAC
AGCGACGACTCCAGATCCGATTTAGCAAGCAACGGGGCACCCACCGACAGCTACGAATCT
TTAACAGCAAGTAGTGAGTCTCTAGCAGAAAGCAACGATGCACCCAGCAACAGCTATGAA
TCTTTTCCAGAAATTAGAGAAAATCTAACCGCAAGTGAGGAATCCCTAACA---------
------------GGAAGTAATGAATCCCTAACAGGAAGTAATGAATCCCTAACAGAAAGT
AAT------------------------------------------GAATCCCTAACAGGA
AGTAATGAATCCCTAACAGGAAGTAATGAATCCCTAACAGGAAGTAATGAATCCCTAACA
GAAAGTAGAGAATCTCTAGAGGCCAGTAGAGAATCGCTAAGAGCAAGTAGAGAGTCTCTA
GCGGCCAGTAGAGAATCCCTGAACGACTTTTGTGGGAGCGAAGAATCAGTAGCATTCGAA
GGAGAGCCAAATGAAAAGACATTCATGGGAGACGTCTTAAGTGGTGGAGAATGTGAGAAT
AGTCTCTCAAGAGAAGATTTATTTCATATAGAAGTAGGATCCGAAGAATCGCTAGATGAT
GCCTCAAAATATAATTTCCAAAAGGATTTATCAACTAGCGATAATAGTTCATTCGAAGAT
GATCAGTCATTGAAAAGGGGACTCAAAAGGAACAGCTCAGTATCTAGTCTGGACAGCGAT
ATGGGAAGTTATAAAAATGGAAGATCTAGAGACAGATTGGTTATATATTCGGACCTACCT
AGAAGGCCAATACATGGGGATAATGCACCACAAGAAAAGGAGAAATGTCTGGATTTCAAA
GAACTTCTAGAGAGAGAAAGGAACAACCACCAAAGGAATAGTGTAAATATAAAATTAAAA
AATATATACGGAGAAGAACAATTGTATAATGTGAAGAAAGATAGCTTTATTGTAAAATTG
ATATACTATCTGCCGTTTGGCCCAATTGTTACAATGATAATGATAGGTTTTATTCTACTC
TGGTCAACGCCCTGGACTGGTTTTATTTTTATAGGGTGTAGCTCTATTATATTACCAGCT
TTGTTATACGCGATGCATTCAAACAAAAAACAATTCGAACGTGTATTTGGAAAAGGGAGA
AAAAAGCCAATGAAGGTAAAAAAAGGAGGCGGGAAAATATCTAGATTTTTTAAAGAAGTA
GACAAA---------------------------
>CH148_R .
------------------------------TTAAGGAAAATTTCCCTGTGTTCTATTTTC
GTATTGGCATATCTCAGTTCTGCTAAGTGCGCGTACCGAGACGAATCTGACATATTGTAC
AGCGACGACTCCAGATCCGATTTAGCAAGCAACGGGGCACCCACCGACAGCTACGAATCT
TTAACAGCAAGTAGTGAGTCTCTAGCAGAAAGCAACGATGCACCCAGCAACAGCTATGAA
TCTTTTCCAGAAATTAGAGAAAATCTAACCGCAAGTGAGGAATCCCTAACATCATGTGAG
GAATCCCTAACAGGAAGTAATGAATCCCTAACAGGAAGTAATGAATCCCTAACAGGAAGT
AAT---------------------------------------------------------
------------------------------------------------GAATCCCTAACA
GAAAGTAGAGAATCTCTAGAGGCCAGTAGAGAATCGCTAAGAGCAAGTAGAGAGTCTCTA
GCGGCCAGTAGAGAATCCCTGAACGACTTTTGTGGGAGCGAAGAATCAGTAGCATTCGAA
GGAGAGCCAAATGAAAAGACATTCATGGGAGACGTCTTAAGTGGTGGAGAATGTGAGAAT
AGTCTCTCAAGAGAAGATTTATTTCATATAGAAGTAGGATCCGAAGAATCGCTAGATGAT
GCCTCAAAATATAATTTCCAAAAGGATTTATCAACTAGCGATAATAGTTCATTCGAAGAT
GATCAGTCATTGAAAAGGGGACTCAAAAGGAACAGCTCAGTATCTAGTCTGGACAGCGAT
ATGGGAAGTTATAAAAATAAAAGTTATAGACACAGATTGGATATATATTCGGACCTACCT
AGAAGGCCAATACATGGGGATAATGCACCACAAGAAAAGGAGAAATGTCTGGATTTCAAA
GAACTTCTAGAGAGAGAAAGGAACAACCACCAAAGGAATAGTGTAAATGTAAAATTAAAA
AATATTTACGGAGAAGAACAAATGTATAAAGTGAAGAGAAATAGCTTTATTGTAAAATTG
ATATACTATCTGCCGTTTGGCCCAATTGTTACAATGATAATGATAGGCTTTATTCTACTC
TGCTCAACGCCCTCGACTGGTTTTATTTTTATAGGGTGTAGCTCTATTATATTACCAGCT
TTGTTATACGCGATGCATTCAAACAAAAAACAATTCGAACGTGTATTTGGAAAAGGGAGA

```
AAAAAGCCAATGAAGGTAAAAAAAGGAGGCGGGAAAATATCTAGATTTTTTAAAGAAGTA
GACAAA---------------------------
>CH151 .
------------------------------TTAAGGAAAATTTCCCTGTGTTCTATTTTC
GTATTGGCATATCTCAGTTCTGCTAAGTGCGCGTACCGAGACGAATCTGACATATTGTAC
AGCGACGACTCCAGATCCGATTTAGCAAGCAACGGGGCACCCACCGACAGCTACGAATCT
TTAACAGCAAGTAGTGAGTCTCTAGCAGAAAGCAACGATGCACCCAGCAACAGCTATGAA
TCTTTTCCAGAAATTAGAGAAAATCTAACCGCAAGTGAGGAATCCCTAACATCATGTGAG
GAATCCCTAACAGGAAGTAATGAATCCCTAACAGGAAGTAATGAATCCCTAACAGGAAGT
AAT---------------------GAATCCCTAACAGGAAGTAATGAATCCCTAACAGGA
AGTAATGAATCCCTAACAGGAAGTAATGAATCCCTAACAGGAAGTAATGAATCCCTAACA
GAAAGTAGAGAATCTCTAGAGGCCAGTAGAGAATCGCTAAGAGCAAGTAGAGAGTCTCTA
GCGGCCAGTAGAGAATCCCTGAACGACTTTTGTGGGAGCGAAGAATCAGTAGCATTCGAA
GGAGAGCCAAATGAAAAGACATTCATGGGAGACGTCTTAAGTGGTGGAGAATGTGAGAAT
AGTCTCTCAAGAGAAGATTTATTTCATATAGAAGTAGGATCCGAAGAATCGCTAGATGAT
GCCTCAAAATATAATTTCCAAAAGGATTTATCAACTAGCGATAATAGTTTATTCGAAGAT
GATCAGTCATTGAAAAGGGGACTCAAAAGGAACAGCTCAGTATCTAGTCTGGACAGCGAT
ATGGGAAGTTATAAAAATGGAAGATCTAGAGACAGATTGGATATATATTCGGACCTACCT
AGAAGGCTAATACATGGGGATAATGCACCACAAGAAAAGGAGAAATGTCTGGATTTCAAA
GAACTTCTAGAGAGAGAAAGGAACAACCACCAAAGGAATAGTGTAAATATAAAATTAAAA
AATATATACGGAGAAGAACAATTGTATAATGTGAAGAAAGATAGCTTTATTGTAAAATTG
ATATACTATCTGCCGTTTGGCCCAATTGTTACAATGATAATGATAGGTTTTTTTCTACTC
TGCTGGACCCCCTCGATTGGTTTTACTTTTATAGGGTGTAGCTCTATTATATTACCAGCT
TTGTTATACGCGATGCATTCAAACAAAAAACAATTCGAACGTGTATTTGGAAAAGGGAGA
AAAAAGCCAATGAAGGTAAAAAAAGGAGGCGGGAAAATATCTAGATTTTTTAAAGAAGTA
GACAAA---------------------------
>CH182 .
------------------------------TTAAGGAAAATTTCCCTGTGTTCTATTTTC
GTATTGGCATATCTCAGTTCTGCTAAGTGCGCGTACCGAGACGAATCTGACATATTGTAC
AGCGACGACTCCAGATCCGATTTAGCAAGCAACGGGGCACCCACCGACAGCTACGAATCT
TTAACAGCAAGTAGTGAGTCTCTAGCAGAAAGCAACGATGCACCCAGCAACAGCTATGAA
TCTTTTCCAGAAATTAGAGAAAATCTAACCGCAAGTGAGGAATCCCTAACATCATGTGAG
GAATCCCTAACAGGAAGTAATGAATCCCTAACAGGAAGTAATGAATCCCTAACAGGAAGT
AAT---------------------------------------------------------
------GAATCCCTAACAGGAAGTAATGAATCCCTAACAGGAAGTAATGAATCCCTAACA
GAAAGTAGAGAATCTCTAGAGGCCAGTAGAGAATCGCTAAGAGCAAGTAGAGAGTCTCTA
GCGGCCAGTAGAGAATCCCTGAACGACTTTTGTGGGAGCGAAGAATCAGTAGCATTCGAA
GGAGAGCCAAATGAAAAGACATTCATGGGAGACGTCTTAAGTGGTGGAGAATGTGAGAAT
AGTCTCTCAAGAGAAGATTTATTTCATATAGAAGTAGGATCCGAAGAATCGCTAGATGAT
GCCTCAAAATATAATTTCCAAAAGGATTTATCAACTAGCGATAATAGTTTATTCGAAGAT
GATCAGTCATTGAAAAGGGGACTCAAAAGGAACAGCTCAGTATCTAGTCTGGACAGCGAT
ATGGGAAGTTATAAAAATAAAAGTTATAGACACAGATTGGATATATATTCGGACCTACCT
AGAAGGCTAATACATGGGGATAATGCACCACAAGAAAAGGAGAAATGTCTGGATTTCAAA
GAACTTCTAGAGAGAGAAAGGAACAACCACCAAAGGAATAGTGTAAATATAAAATTAAAA
AATATATACGGAGAAGAACAATTGTATAATGTGAAGAAAGATAGCTTTATTGTAAAATTG
ATATACTATCTGCCGTTTGGCCCAATTGTTACAATGATAATGATAGGCTTTATTCTACTC
```

```
TGCTCAACGCCCTCGACTGGTTTTATTTTTATAGGGTGTAGCTCTATTATATTACCAGCT
TTGTTATACGCGATGCATTCAAACAAAAAACAATTCGAACGTGTATTTGGAAAAGGGAGA
AAAAAGCCAATGAAGGTAAAAAAAGGAGGCGGGAAAATATCTAGATTTTTTAAAGAAGTA
GACAAA---------------------------
>CHH2 .
------------------------------TTAAGGAAAATTTCCCTGTGTTCTATTTTC
GTATTGGCATATCTCAGTTCTGCTAAGTGCGCGTACCGAGACGAATCTGACATATTGTAC
AGCGACGACTCCAGATCCGATTTAGCAAGCAACGGGGCACCCACCGACAGCTACGAATCT
TTAACAGCAAGTAGTGAGTCTCTAGCAGAAAGCAACGATGCACCCAGCAACAGCTATGAA
TCTTTTCCAGAAATTAGAGAAAATCTAACCGCAAGTGAGGAATCCCTAACATCATGTGAG
GAATCCCTAACAGGAAGTAATGAATCCCTAACAGGAAGTAATGAATCCCTAACAGGAAGT
AAT---------------------------------------------------------
---------------------------GAATCCCTAACAGGAAGTAATGAATCCCTAACA
GAAAGTAGAGAATCTCTAGAGGCCAGTAGAGAATCGCTAAGAGCAAGTAGAGAGTCTCTA
GCGGCCAGTAGAGAATCCCTGAACGACTTTTGTGGGAGCGAAGAATCAGTAGCATTCGAA
GGAGAGCCAAATGAAAAGACATTCATGGGAGACGTCTTAAGTGGTGGAGAATGTGAGAAT
AGTCTCTCAAGAGAAGATTTATTTCATATAGAAGTAGGATCCGAAGAATCGCTAGATGAT
GCCTCAAAATATAATTTCCAAAAGGATTTATCAACTAGCGATAATAGTTCATTCGAAGAT
GATCAGTCATTGAAAAGGGGACTCAAAAGGAACAGCTCAGTATCTAGTCTGGACAGCGAT
ATGGGAAGTTATAAAAATAAAAGTTATAGACACAGATTGGATATATATTCGGACCTACCT
AGAAGGCCAATACATGGGGATAATGCACCACAAGAAAAGGAGAAATGTCTGGATTTCAAA
GAACTTCTAGAGAGAGAAAGGAACAACCACCAAAGGAATAGTGTAAATATAAAATTAAAA
AATATATACGGAGAAGAACAATTGTATAATGTGAAGAAAGATAGCTTTATTGTAAAATTG
ATATACTATCTGCCGTTTGGCCCAATTGTTACAATGATAATGATAGGCTTTATTCTACTC
TGGTCAACGGGCTGGACTGGTTTTATTTTTATAGGGTGTAGCTCTATTATATTACCAGCT
TTGTTATACGCGATGTATTCAAACAAAAAACAATTCGAACGTGTATTTGGAAAAGGGAGA
AAAAAGCCAATGAAGGTAAAAAAAGGAGGCGGGAAAATATCTAGATTTTTTAAAGAAGTA
GACAAA---------------------------
>CHH7 .
------------------------------TTAAGGAAAATTTCCCTGTGTTCTATTTTC
GTATTGGCATATCTCAGTTCTGCTAAGTGCGCGTACCGAGACGAATCTGACATATTGTAC
AGCGACGACTCCAGATCCGATTTAGCAAGCAACGGGGCACCCACCGACAGCTACGAATCT
TTAACAGCAAGTAGTGAGTCTCTAGCAGAAAGCAACGATGCACCCAGCAACAGCTATGAA
TCTTTTCCAGAAATTAGAGAAAATCTAACCGCAAGTGAGGAATCCCTAACATCATGTGAG
GAATCCCTAACAGGAAGTAATGAATCCCTAACAGGAAGTAATGAATCCCTAACAGGAAGT
AAT---------------------------------------------------------
---------------------------GAATCCCTAACAGGAAGTAATGAATCCCTAACA
GAAAGTAGAGAATCTCTAGAGGCCAGTAGAGAATCGCTAAGAGCAAGTAGAGAGTCTCTA
GCGGCCAGTAGAGAATCCCTGAACGACTTTTGTGGGAGCGAAGAATCAGTAGCATGCGAA
GGAGAGCCAAATGAAAAGACATTCATGGGAGACGTCTTAAGTGGTGGAGAATGTGAGAAT
AGTCTCTCAAGAGAAGATTTATTTCATATAGAAGTAGGATCCGAAGAATCGCTAGATGAT
GCCTCAAAATATAATTTCCAAAAGGATTTATCAACTAGCGATAATAGTTCATTCGAAGAT
GATCAGTCATTGAAAAGGGGACTCAAAAGGAACAGCTCAGTATCTAGTCTGGACAGCGAT
ATGGGAAGTTATAAAAATGGAAGATCTAGAGACAGATTGGATATATATTCGGACCTACCT
AGAAGGCTAATACATGGGGATAATGCACCACAAGAAAAGGAGAAATGTCTGGATTTCAAA
GAACTTCTAGAGAGAGAAAGGAACAACCACCAAAGGAATAGTGTAAATATAAAATTAAAA
AATATATACGGAGAAGAACAATTGTATAATGTGAAGAAAGATAGCTTTATTGTAAAATTG
ATATACTATCTGCCGTTTGGCCCAATTGTTACAATGATAATGATAGGCTTTATTCTACTC
TGCTCAACGCCCTCGACTGGTTTTATTTTTATAGGGTGTAGCTCTATTATATTACCAGCT
TTGTTATACGCGATGCATTCAAACAAAAAACAATTCGAACGTGTATTTGGAAAAGGGAGA
```

AAAAAGCCAATGAAGGTAAAAAAAGGAGGCGGGAAAATATCTAGATTTTTTAAAGAAGTA
GACAAA---------------------------
>CHINS2 .
------------------------------TTAAGGAAAATTTCCCTGTGTTCTATTTTC
GTATTGGCATATCTCAGTTCTGCTAAGTGCGCGTACCGAGACGAATCTGACATATTGTAC
AGCGACGACTCCAGATCCGATTTAGCAAGCAACGGGGCACCCACCGACAGCTACGAATCT
TTAACAGCAAGTAGTGAGTCTCTAGCAGAAAGCAACGATGCACCCAGCAACAGCTATGAA
TCTTTTCCAGAAATTAGAGAAAATCTAACCGCAAGTGAGGAATCCCTAACATCATGTGAG
GAATCCCTAACAGGAAGTAATGAATCCCTAACAGGAAGTAATGAATCCCTAACAGGAAGT
AAT---------------------------------------------------------
------------------------------------------------GAATCCCTAACA
GAAAGTAGAGAATCTCTAGAGGCCAGTAGAGAATCGCTAAGAGCAAGTAGAGAGTCTCTA
GCGGCCAGTAGAGAATCCCTGAACGACTTTTGTGGGAGCGAAGAATCAGTAGCATTCGAA
GGAGAGCCAAATGAAAAGACATTCATGGGAGACGTCTTAAGTGGTGGAGAATGTGAGAAT
AGTCTCTCAAGAGAAGATTTATTTCATATAGAAGTAGGATCCGAAGAATCGCTAGATGAT
GCCTCAAAATATAATTTCCAAAAGGATTTATCAACTAGCGATAATAGTTTATTCGAAGAT
GATCAGTCATTGAAAAGGGGACTCAAAAGGAACAGCTCAGTATCTAGTCTGGACAGCGAT
ATGGGAAGTTATAAAAATGGAAGATCTAGAGACAGATTGGATATATATTCGGACCTACCT
AGAAGGCTAATACATGGGGATAATGCACCACAAGAAAAGGAGAAATGTCTGGATTTCAAA
GAACTTCTAGAGAGAGAAAGGAACAACCACCAAAGGAATAGTGTAAATATAAAATTAAAA
AATATATACGGAGAAGAACAATTGTATAATGTGAAGAAAGATAGCTTTATTGTAAAATTG
ATATACTATCTGCCGTTTGGCCCAATTGTTACAATGATAATTATAGGCTTTATTCTACTC
TGGTCAACGCCCTGGACTGGTTTTATTTTTATAGGGTGTAGCTCTATTATATTACCAGCT
TTGTTATACGCGATGCATTCAAACAAAAAACAATTCGAACGTGTATTTGGAAAAGGGAGA
AAAAAGCCAATGAAGGTAAAAAAAGGAGGCGGGAAAATATCTAGATTTTTTAAAGAAGTA
GACAAA---------------------------
>CHINS4 .
------------------------------TTAAGGAAAATTTCCCTGTGTTCTATTTTC
GTATTGGCATATCTCAGTTCTGCTAAGTGCGCGTACCGAGACGAATCTGACATATTGTAC
AGCGACGACTCCAGATCCGATTTAGCAAGCAACGGGGCACCCACCGACAGCTACGAATCT
TTAACAGCAAGTAGTGAGTCTCTAGCAGAAAGCAACGATGCACCCAGCAACAGCTATGAA
TCTTTTCCAGAAATTAGAGAAAATCTAACCGCAAGTGAGGAATCCCTAACATCATGTGAG
GAATCCCTAACAGGAAGTAATGAATCCCTAACAGGAAGTAATGAATCCCTAACAGGAAGT
AAT---------------------------------------------------------
------------------------------------------------GAATCCCTAACA
GAAAGTAGAGAATCTCTAGAGGCCAGTAGAGAATCGCTAAGAGCAAGTAGAGAGTCTCTA
GCGGCCAGTAGAGAATCCCTGAACGACTTTTGTGGGAGCGAAGAATCAGTAGCATTCGAA
GGAGAGCCAAATGAAAAGACATTCATGGGAGACGTCTTAAGTGGTGGAGAATGTGAGAAT
AGTCTCTCAAGAGAAGATTTATTTCATATAGAAGTAGGATCCGAAGAATCGCTAGATGAT
GCCTCAAAATATAATTTCCAAAAGGATTTATCAACTAGCGATAATAGTTTATTCGAAGAT
GATCAGTCATTGAAAAGGGGACTCAAAAGGAACAGCTCAGTATCTAGTCTGGACAGCGAT
ATGGGAAGTTATAAAAATGGAAGATCTAGAGACAGATTGGATATATATTCGGACCTACCT
AGAAGGCTAATACATGGGGATAATGCACCACAAGAAAAGGAGAAATGTCTGGATTTCAAA
GAACTTCTAGAGAGAGAAAGGAACAACCACCAAAGGAATAGTGTAAATATAAAATTAAAA
AATATATACGGAGAAGAACAATTGTATAATGTGAAGAAAGATAGCTTTATTGTAAAATTG
ATATACTATCTGCCGTTTGGCCCAATTGTTACAATGATAATTATAGGCTTTATTCTACTC

TGGTCAACGCCCTGGACTGGTTTTATTTTTATAGGGTGTAGCTCTATTATATTACCAGCT
TTGTTATACGCGATGCATTCAAACAAAAAACAATTCGAACGTGTATTTGGAAAAGGGAGA
AAAAAGCCAATGAAGGTAAAAAAAGGAGGCGGGAAAATATCTAGATTTTTTAAAGAAGTA
GACAAA---------------------------
>CHINS5 .
------------------------------TTAAGGAAAATTTCCCTGTGTTCTATTTTC
GTATTGGCATATCTCAGTTCTGCTAAGTGCGCGTACCGAGACGAATCTGACATATTGTAC
AGCGACGACTCCAGATCCGATTTAGCAAGCAACGGGGCACCCACCGACAGCTACGAATCT
TTAACAGCAAGTAGTGAGTCTCTAGCAGAAAGCAACGATGCACCCAGCAACAGCTATGAA
TCTTTTCCAGAAATTAGAGAAAATCTAACCGCAAGTGAGGAATCCCTAACATCATGTGAG
GAATCCCTAACAGGAAGTAATGAATCCCTAACAGGAAGTAATGAATCCCTAACAGGAAGT
AAT---------------------------------------------------------
---------------------------GAATCCCTAACAGGAAGTAATGAATCCCTAACA
GAAAGTAGAGAATCTCTAGAGGCCAGTAGAGAATCGCTAAGAGCAAGTAGAGAGTCTCTA
GCGGCCAGTAGAGAATCCCTGAACGACTTTTGTGGGAGCGAAGAATCAGTAGCATTCGAA
GGAGAGCCAAATGAAAAGACATTCATGGGAGACGTCTTAAGTGGTGGAGAATGTGAGAAT
AGTCTCTCAAGAGAAGATTTATTTCATATAGAAGTAGGATCCGAAGAATCGCTAGATGAT
GCCTCAAAATATAATTTCCAAAAGGATTTATCAACTAGCGATAATAGTTCATTCGAAGAT
GATCAGTCATTGAAAAGGGGACTCAAAAGGAACAGCTCAGTATCTAGTCTGGACAGCGAT
ATGGGAAGTTATAAAAATGGAAGATCTAGAGACAGATTGGATATATATTCGGACCTACCT
AGAAGGCTAATACATGGGGATAATGCACCACAAGAAAAGGAGAAATGTCTGGATTTCAAA
GAACTTCTAGAGAGAGAAAGGAACAACCACCAAAGGAATAGTGTAAATATAAAATTAAAA
AATATATACGGAGAAGAACAATTGTATAATGTGAAGAAAGATAGCTTTATTGTAAAATTG
ATATACTATCTGCCGTTTGGCCCAATTGTTACAATGATAATGATAGGCTTTATTCTACTC
TGCTCAACGCCCTCGACTGGTTTTATTTTTATAGGGTGTAGCTCTATTATATTACCAGCT
TTGTTATACGCGATGCATTCAAACAAAAAACAATTCGAACGTGTATTTGGAAAAGGGAGA
AAAAAGCCAATGAAGGTAAAAAAAGGAGGCGGGAAAATATCTAGATTTTTTAAAGAAGTA
GACAAA---------------------------
>CHA1 .
------------------------------TTAAGGAAAATTTCCCTGTGTTCTATTTTC
GTATTGGCATATCTCAGTTCTGCTAAGTGCGCGTACCGAGACGAATCTGACATATTGTAC
AGCGACGACTCCAGATCCGATTTAGCAAGCAACGGGGCACCCACCGACAGCTACGAATCT
TTAACAGCAAGTAGTGAGTCTCTAGCAGAAAGCAACGATGCACCCAGCAACAGCTATGAA
TCTTTTCCAGAAATTAGAGAAAATCTAACCGCAAGTGAGGAATCCCTAACATCATGTGAG
GAATCCCTAACAGGAAGTAATGAATCCCTAACAGGAAGTAATGAATCCCTAACAGGAAGT
AAT---------------------------------------------------------
------------------------------------------------GAATCCCTAACA
GAAAGTAGAGAATCTCTAGAGGCCAGTAGAGAATCGCTAAGAGCAAGTAGAGAGTCTCTA
GCGGCCAGTAGAGAATCCCTGAACGACTTTTGTGGGAGCGAAGAATCAGTAGCATTCGAA
GGAGAGCCAAATGAAAAGACATTCATGGGAGACGTCTTAAGTGGTGGAGAATGTGAGAAT
AGTCTCTCAAGAGAAGATTTATTTCATATAGAAGTAGGATCCGAAGAATCGCTAGATGAT
GCCTCAAAATATAATTTCCAAAAGGATTTATCAACTAGCGATAATAGTTTATTCGAAGAT
GATCAGTCATTGAAAAGGGGACTCAAAAGGAACAGCTCAGTATCTAGTCTGGACAGCGAT
ATGGGAAGTTATAAAAATGGAAGATCTAGAGACAGATTGGATATATATTCGGACCTACCT
AGAAGGCTAATACATGGGGATAATGCACCACAAGAAAAGGAGAAATGTCTGGATTTCAAA
GAACTTCTAGAGAGAGAAAGGAACAACCACCAAAGGAATAGTGTAAATATAAAATTAAAA
AATATATACGGAGAAGAACAATTGTATAATGTGAAGAAAGATAGCTTTATTGTAAAATTG
ATATACTATCTGCCGTTTGGCCCAATTGTTACAATGATAATTATAGGCTTTATTCTACTC
TGGTCAACGCCCTGGACTGGTTTTATTTTTATAGGGTGTAGCTCTATTATATTACCAGCT
TTGTTATACGCGATGCATTCAAACAAAAAACAATTCGAACGTGTATTTGGAAAAGGGAGA

```
AAAAAGCCAATGAAGGTAAAAAAAGGAGGCGGGAAAATATCTAGATTTTTTAAAGAAGTA
GACAAA---------------------------
>CH4 .
------------------------------TTAAGGAAAATTTCCCTGTGTTCTATTTTC
GTATTGGCATATCTCAGTTCTGCTAAGTGCGCGTACCGAGACGAATCTGACATATTGTAC
AGCGACGACTCCAGATCCGATTTAGCAAGCAACGGGGCACCCACCGACAGCTACGAATCT
TTAACAGCAAGTAGTGAGTCTCTAGCAGAAAGCAACGATGCACCCAGCAACAGCTATGAA
TCTTTTCCAGAAATTAGAGAAAATCTAACCGCAAGTGAGGAATCCCTAACATCATGTGAG
GAATCCCTAACAGGAAGTAACGAATCCCTAACAGGAAGTAATGAATCCCTAACAGGAAGT
AAT---------------------------------------------------------
---------------------------GAATCCCTAACAGGAAGTAATGAATCCCTAACA
GAAAGTAGAGAATCTCTAGAGGCCAGTAGAGAATCGCTAAGAGCAAGTAGAGAGTCTCTA
GCGGCCAGTAGAGAATCCCTGAACGACTTTTGTGGGAGCGAAGAATCAGTAGCATTCGAA
GGAGAGCCAAATGAAAAGACATTCATGGGAGACGTCTTAAGTGGTGGAGAATGTGAGAAT
AGTCTCTCAAGAGAAGATTTATTTCATATAGAAGTAGGATCCGAAGAATCGCTAGATGAT
GCCTCAAAATATAATTTCCAAAAGGATTTATCAACTAGCGATAATAGTTCATTCGAAGAT
GATCAGTCATTGAAAAGGGGACTCAAAAGGAACAGCTCAGTATCTAGTCTGGACAGCGAT
ATGGGAAGTTATAAAAATGGAAGATCTAGAGACAGATTGGATATATATTCGGACCTACCT
AGAAGGCTAATACATGGGGATAATGCACCACAAGAAAAGGAGAAATGTCTGGATTTCAAA
GAACTTCTAGAGAGAGAAAGGAACAACCACCAAAGGAATAGTGTAAATATAAAATTAAAA
AATATATACGGAGAAGAACAATTGTATAATGTGAAGAAAGATAGCTTTATTGTAAAATTG
ATATACTATCTGCCGTTTGGCCCAATTGTTACAATGATAATGATAGGTTTTTTTCTACTC
TGCTGGACCCCCTCGATTGGTTTTACTTTTATAGGGTGTAGCTCTATTATATTACCAGCT
TTGTTATACGCGATGCATTCAAACAAAAAACAATTCGAACGTGTATTTGGAAAAGGGAGA
AAAAAGCCAATGAAGGTAAAAAAAGGAGGCGGGAAAATATCTAGATTTTTTAAAGAAGTA
GACAAA---------------------------
>CH5 .
------------------------------TTAAGGAAAATTTCCCTGTGTTCTATTTTC
GTATTGGCATATCTCAGTTCTGCTAAGTGCGCGTACCGAGACGAATCTGACATATTGTAC
AGCGACGACTCCAGATCCGATTTAGCAAGCAACGGGGCACCCACCGACAGCTACGAATCT
TTAACAGCAAGTAGTGAGTCTCTAGCAGAAAGCAACGATGCACCCAGCAACAGCTATGAA
TCTTTTCCAGAAATTAGAGAAAATCTAACCGCAAGTGAGGAATCCCTAACATCATGTGAG
GAATCCCTAACAGGAAGTAATGAATCCCTAACAGGAAGTAATGAATCCCTAACAGGAAGT
AAT---------------------------------------------------------
------------------------------------------------GAATCCCTAACA
GAAAGTAGAGAATCTCTAGAGGCCAGTAGAGAATCGCTAAGAGCAAGTAGAGAGTCTCTA
GCGGCCAGTAGAGAATCCCTGAACGACTTTTGTGGGAGCGAAGAATCAGTAGCATTCGAA
GGAGAGCCAAATGAAAAGACATTCATGGGAGACGTCTTAAGTGGTGGAGAATGTGAGAAT
AGTCTCTCAAGAGAAGATTTATTTCATATAGAAGTAGGATCCGAAGAATCGCTAGATGAT
GCCTCAAAATATAATTTCCAAAAGGATTTATCAACTAGCGATAATAGTTTATTCGAAGAT
GATCAGTCATTGAAAAGGGGACTCAAAAGGAACAGCTCAGTATCTAGTCTGGACAGCGAT
ATGGGAAGTTATAAAAATGGAAGATCTAGAGACAGATTGGATATATATTCGGACCTACCT
AGAAGGCTAATACATGGGGATAATGCACCACAAGAAAAGGAGAAATGTCTGGATTTCAAA
GAACTTCTAGAGAGAGAAAGGAACAACCACCAAAGGAATAGTGTAAATATAAAATTAAAA
AATATATACGGAGAAGAACAATTGTATAATGTGAAGAAAGATAGCTTTATTGTAAAATTG
ATATACTATCTGCCGTTTGGCCCAATTGTTACAATGATAATGATAGGCTTTATTCTACTC
```

TGGTCAACGCCCTGGACTGGTTTTATTTTTATAGGGTGTAGCTCTATTATATTACCAGCT
TTGTTATACGCGATGCATTCAAACAAAAAACAATTCGAACGTGTATTTGGAAAAGGGAGA
AAAAAGCCAATGAAGGTAAAAAAAGGAGGCGGGAAAATATCTAGATTTTTTAAAGAAGTA
GACAAA---------------------------
>CH38 .
------------------------------TTAAGGAAAATTTCCCTGTGTTCTATTTTC
GTATTGGCATATCTCAGTTCTGCTAAGTGCGCGTACCGAGACGAATCTGACATATTGTAC
AGCGACGACTCCAGATCCGATTTAGCAAGCAACGGGGCACCCACCGACAGCTACGAATCT
TTAACAGCAAGTAGTGAGTCTCTAGCAGAAAGCAACGATGCACCCAGCAACAGCTATGAA
TCTTTTCCAGAAATTAGAGAAAATCTAACCGCAAGT---------------------GAG
GAATCCCTAACAGGAAGTAATGAATCCCTAACAGGAAGTAATGAATCCCTAACAGAAAGT
AAT------------------------------------------GAATCCCTAACAGGA
AGTAATGAATCCCTAACAGGAAGTAATGAATCCCTAACAGGAAGTAATGAATCCCTAACA
GAAAGTAGAGAATCTCTAGAGGCCAGTAGAGAATCGCTAAGAGCAAGTAGAGAGTCTCTA
GCGGCCAGTAGAGAATCCCTGAACGACTTTTGTGGGAGCGAAGAATCAGTAGCATTCGAA
GGAGAGCCAAATGAAAAGACATTCATGGGAGACGTCTTAAGTGGTGGAGAATGTGAGAAT
AGTCTCTCAAGAGAAGATTTATTTCATATAGAAGTAGGATCCGAAGAATCGCTAGATGAT
GCCTCAAAATATAATTTCCAAAAGGATTTATCAACTAGCGATAATAGTTTATTCGAAGAT
GATCAGTCATTGAAAAGGGGACTCAAAAGGAACAGCTCAGTATCTAGTCTGGACAGCGAT
ATGGGAAGTTATAAAAATGGAAGATCTAGAGACAGATTGGATATATATTCGGACCTACCT
AGAAGGCTAATACATGGGGATAATGCACCACAAGAAAAGGAGAAATGTCTGGATTTCAAA
GAACTTCTAGAGAGAGAAAGGAACAACCACCAAAGGAATAGTGTAAATATAAAATTAAAA
AATATATACGGAGAAGAACAATTGTATAATGTGAAGAAAGATAGCTTTATTGTAAAATTG
ATATACTATCTGCCGTTTGGCCCAATTGTTACAATGATAATGATAGGTTTTTTTCTACTC
TGCTGGACCCCCTCGATTGGTTTTATTTTTATAGGGTGTAGCTCTATTATATTACCAGCT
TTGTTATACGCGATGCATTCAAACAAAAAACAATTCGAACGTGTATTTGGAAAAGGGAGA
AAAAAGCCAATGAAGGTAAAAAAAGGAGGCGGGAAAATATCTAGATTTTTTAAAGAAGTA
GACAAA---------------------------
>CH41_R .
------------------------------TTAAGGAAAATTTCCCTGTGTTCTATTTTC
GTATTGGCATATCTCAGTTCTGCTAAGTGCGCGTACCGAGACGAATCTGACATATTGTAC
AGCGACGACTCCAGATCCGATTTAGCAAGCAACGGGGCACCCACCGACAGCTACGAATCT
TTAACAGCAAGTAGTGAGTCTCTAGCAGAAAGCAACGATGCACCCAGCAACAGCTATGAA
TCTTTTCCAGAAATTAGAGAAAATCTAACCGCAAGTGAGGAATCCCTAACATCATGTGAG
GAATCCCTAACAGGAAGTAATGAATCCCTAACAGGAAGTAATGAATCCCTAACAGGAAGT
AAT---------------------------------------------------------
------------------------------------------------GAATCCCTAACA
GAAAGTAGAGAATCTCTAGAGGCCAGTAGAGAATCGCTAAGAGCAAGTAGAGAGTCTCTA
GCGGCCAGTAGAGAATCCCTGAACGACTTTTGTGGGAGCGAAGAATCAGTAGCATTCGAA
GGAGAGCCAAATGAAAAGACATTCATGGGAGACGTCTTAAGTGGTGGAGAATGTGAGAAT
AGTCTCTCAAGAGAAGATTTATTTCATATAGAAGTAGGATCCGAAGAATCGCTAGATGAT
GCCTCAAAATATAATTTCCAAAAGGATTTATCAACTAGCGATAATAGTTTATTCGAAGAT
GATCAGTCATTGAAAAGGGGACTCAAAAGGAACAGCTCAGTATCTAGTCTGGACAGCGAT
ATGGGAAGTTATAAAAATGGAAGATCTAGAGACAGATTGGATATATATTCGGACCTACCT
AGAAGGCTAATACATGGGGATAATGCACCACAAGAAAAGGAGAAATGTCTGGATTTCAAA
GAACTTCTAGAGAGAGAAAGGAACAACCACCAAAGGAATAGTGTAAATATAAAATTAAAA
AATATATACGGAGAAGAACAATTGTATAATGTGAAGAAAGATAGCTTTATTGTAAAATTG
ATATACTATCTGCCGTTTGGCCCAATTGTTACAATGATAATGATAGGCTTTATTCTACTC
TGGTCAACGCCCTGGACTGGTTTTATTTTTATAGGGTGTAGCTCTATTATATTACCAGCT
TTGTTATACGCGATGCATTCAAACAAAAAACAATTCGAACGTGTATTTGGAAAAGGGAGA

```
AAAAAGCCAATGAAGGTAAAAAAAGGAGGCGGGAAAATATCTAGATTTTTTAAAGAAGTA
GACAAA---------------------------
>CH48 .
------------------------------TTAAGGAAAATTTCCCTGTGTTCTATTTTC
GTATTGGCATATCTCAGTTCTGCTAAGTGCGCGTACCGAGACGAATCTGACATATTGTAC
AGCGACGACTCCAGATCCGATTTAGCAAGCAACGGGGCACCCACCGACAGCTACGAATCT
TTAACAGCAAGTAGTGAGTCTCTAGCAGAAAGCAACGATGCACCCAGCAACAGCTATGAA
TCTTTTCCAGAAATTAGAGAAAATCTAACCGCAAGTGAGGAATCCCTAACATCATGTGAG
GAATCCCTAACAGGAAGTAATGAATCCCTAACAGGAAGTAATGAATCCCTAACAGGAAGT
AAT---------------------------------------------------------
---------------------------GAATCCCTAACAGGAAGTAATGAATCCCTAACA
GAAAGTAGAGAATCTCTAGAGGCCAGTAGAGAATCGCTAAGAGCAAGTAGAGAGTCTCTA
GCGGCCAGTAGAGAATCCCTGAACGACTTTTGTGGGAGCGAAGAATCAGTAGCATTCGAA
GGAGAGCCAAATGAAAAGACATTCATGGGAGACGTCTTAAGTGGTGGAGAATGTGAGAAT
AGTCTCTCAAGAGAAGATTTATTTCATATAGAAGTAGGATCCGAAGAATCGCTAGATGAT
GCCTCAAAATATAATTTCCAAAAGGATTTATCAACTAGCGATAATAGTTTATTCGAAGAT
GATCAGTCATTGAAAAGGGGACTCAAAAGGAACAGCTCAGTATCTAGTCTGGACAGCGAT
ATGGGAAGTTATAAAAATGGAAGATCTAGAGACAGATTGGATATATATTCGGACCTACCT
AGAAGGCTAATACATGGGGATAATGCACCACAAGAAAAGGAGAAATGTCTGGATTTCAAA
GAACTTCTAGAGAGAGAAAGGAACAACCACCAAAGGAATAGTGTAAATATAAAATTAAAA
AATATATACGGAGAAGAACAATTGTATAATGTGAAGAAAGATAGCTTTATTGTAAAATTG
ATATACTATCTGCCGTTTGGCCCAATTGTTACAATGATAATGATAGGCTTTATTCTACTC
TGGTCAACGCCCTGGACTGGTTTTATTTTTATAGGGTGTAGCTCTATTATATTACCAGCT
TTGTTATACGCGATGCATTCAAACAAAAAACAATTCGAACGTGTATTTGGAAAAGGGAGA
AAAAAGCCAATGAAGGTAAAAAAAGGAGGCGGGAAAATATCTAGATTTTTTAAAGAAGTA
GACAAA---------------------------
>CH53 .
------------------------------TTAAGGAAAATTTCCCTGTGTTCTATTTTC
GTATTGGCATATCTCAGTTCTGCTAAGTGCGCGTACCGAGACGAATCTGACATATTGTAC
AGCGACGACTCCAGATCCGATTTAGCAAGCAACGGGGCACCCACCGACAGCTACGAATCT
TTAACAGCAAGTAGTGAGTCTCTAGCAGAAAGCAACGATGCACCCAGCAACAGCTATGAA
TCTTTTCCAGAAATTAGAGAAAATCTAACCGCAAGTGAGGAATCCCTAACATCATGTGAG
GAATCCCTAACAGGAAGTAATGAATCCCTAACAGGAAGTAATGAATCCCTAACAGGAAGT
AAT---------------------------------------------------------
---------------------------GAATCCCTAACAGGAAGTAATGAATCCCTAACA
GAAAGTAGAGAATCTCTAGAGGCCAGTAGAGAATCGCTAAGAGCAAGTAGAGAGTCTCTA
GCGGCCAGTAGAGAATCCCTGAACGACTTTTGTGGGAGCGAAGAATCAGTAGCATTCGAA
GGAGAGCCAAATGAAAAGACATTCATGGGAGACGTCTTAAGTGGTGGAGAATGTGAGAAT
AGTCTCTCAAGAGAAGATTTATTTCATATAGAAGTAGGATCCGAAGAATCGCTAGATGAT
GCCTCAAAATATAATTTCCAAAAGGATTTATCAACTAGCGATAATAGTTCATTCGAAGAT
GATCAGTCATTGAAAAGGGGACTCAAAAGGAACAGCTCAGTATCTAGTCTGGACAGCGAT
ATGGGAAGTTATAAAAATAAAAGTTATAGACACAGATTGGATATATATTCGGACCTACCT
AGAAGGCCAATACATGGGGATAATGCACCACAAGAAAAGGAGAAATGTCTGGATTTCAAA
GAACTTCTAGAGAGAGAAAGGAACAACCACCAAAGGAATAGTGTAAATATAAAATTAAAA
AATATATACGGAGAAGAACAATTGTATAATGTGAAGAAAGATAGCTTTATTGTAAAATTG
ATATACTATCTGCCGTTTGGCCCAATTGTTACAATGATAATGATAGGCTTTATTCTACTC
```

```
TGCTCAACGCCCTCGACTGGTTTTATTTTTATAGGGTGTAGCTCTATTATATTACCAGCT
TTGTTATACGCGATGCATTCAAACAAAAAACAATTCGAACGTGTATTTGGAAAAGGGAGA
AAAAAGCCAATGAAGGTAAAAAAAGGAGGCGGGAAAATATCTAGATTTTTTAAAGAAGTA
GACAAA---------------------------
>CH54 .
------------------------------TTAAGGAAAATTTCCCTGTGTTCTATTTTC
GTATTGGCATATCTCAGTTCTGCTAAGTGCGCGTACCGAGACGAATCTGACATATTGTAC
AGCGACGACTCCAGATCCGATTTAGCAAGCAACGGGGCACCCACCGACAGCTACGAATCT
TTAACAGCAAGTAGTGAGTCTCTAGCAGAAAGCAACGATGCACCCAGCAACAGCTATGAA
TCTTTTCCAGAAATTAGAGAAAATCTAACCGCAAGTGAGGAATCCCTAACATCATGTGAG
GAATCCCTAACAGGAAGTAATGAATCCCTAACAGGAAGTAATGAATCCCTAACAGGAAGT
AAT---------------------------------------------------------
---------------------------GAATCCCTAACAGGAAGTAATGAATCCCTAACA
GAAAGTAGAGAATCTCTAGAGGCCAGTAGAGAATCGCTAAGAGCAAGTAGAGAGTCTCTA
GCGGCCAGTAGAGAATCCCTGAACGACTTTTGTGGGAGCGAAGAATCAGTAGCATTCGAA
GGAGAGCCAAATGAAAAGACATTCATGGGAGACGTCTTAAGTGGTGGAGAATGTGAGAAT
AGTCTCTCAAGAGAAGATTTATTTCATATAGAAGTAGGATCCGAAGAATCGCTAGATGAT
GCCTCAAAATATAATTTCCAAAAGGATTTATCAACTAGCGATAATAGTTCATTCGAAGAT
GATCAGTCATTGAAAAGGGGACTCAAAAGGAACAGCTCAGTATCTAGTCTGGACAGCGAT
ATGGGAAGTTATAAAAATAAAAGTTATAGACACAGATTGGATATATATTCGGACCTACCT
AGAAGGCCAATACATGGGGATAATGCACCACAAGAAAAGGAGAAATGTCTGGATTTCAAA
GAACTTCTAGAGAGAGAAAGGAACAACCACCAAAGGAATAGTGTAAATATAAAATTAAAA
AATATATACGGAGAAGAACAATTGTATAATGTGAAGAAAGATAGCTTTATTGTAAAATTG
ATATACTATCTGCCGTTTGGCCCAATTGTTACAATGATAATGATAGGCTTTATTCTACTT
TGGTCAACGCCCTGGACTGGTTTTATTTTTATAGGGTGTAGCTCTATTATATTACCAGCT
TTGTTATACGCGATGCATTCAAACAAAAAACAATTCGAACGTGTATTTGGAAAAGGGAGA
AAAAAGCCAATGAAGGTAAAAAAAGGAGGCGGGAAAATATCTAGATTTTTTAAAGAAGTA
GACAAA---------------------------
>CH59 .
------------------------------TTAAGGAAAATTTCCCTGTGTTCTATTTTC
GTATTGGCATATCTCAGTTCTGCTAAGTGCGCGTACCGAGACGAATCTGACATATTGTAC
AGCGACGACTCCAGATCCGATTTAGCAAGCAACGGGGCACCCACCGACAGCTACGAATCT
TTAACAGCAAGTAGTGAGTCTCTAGCAGAAAGCAACGATGCACCCAGCAACAGCTATGAA
TCTTTTCCAGAAATTAGAGAAAATCTAACCGCAAGTGAGGAATCCCTAACA---------
------------GGAAGTAATGAATCCCTAACAGGAAGTAATGAATCCCTAACAGAAAGT
AAT------------------------------------------GAATCCCTAACAGGA
AGTAATGAATCCCTAACAGGAAGTAATGAATCCCTAACAGGAAGTAATGAATCCCTAACA
GAAAGTAGAGAATCTCTAGAGGCCAGTAGAGAATCGCTAAGAGCAAGTAGAGAGTCTCTA
GCGGCCAGTAGAGAATCCCTGAACGACTTTTGTGGGAGCGAAGAATCAGTAGCATTCGAA
GGAGAGCCAAATGAAAAGACATTCATGGGAGACGTCTTAAGTGGTGGAGAATGTGAGAAT
AGTCTCTCAAGAGAAGATTTATTTCATATAGAAGTAGGATCCGAAGAATCGCTAGATGAT
GCCTCAAAATATAATTTCCAAAAGGATTTATCAACTAGCGATAATAGTTTATTCGAAGAT
GATCAGTCATTGAAAAGGGGACTCAAAAGGAACAGCTCAGTATCTAGTCTGGACAGCGAT
ATGGGAAGTTATAAAAATGGAAGATCTAGAGACAGATTGGATATATATTCGGACCTACCT
AGAAGGCTAATACATGGGGATAATGCACCACAAGAAAAGGAGAAATGTCTGGATTTCAAA
GAACTTCTAGAGAGAGAAAGGAACAACCACCAAAGGAATAGTGTAAATATAAAATTAAAA
AATATATACGGAGAAGAACAATTGTATAATGTGAAGAAAGATAGCTTTATTGTAAAATTG
ATATACTATCTGCCGTTTGGCCCAATTGTTACAATGATAATGATAGGTTTTTTTCTACTC
TGCTGGACCCCCTCGATTGGTTTTATTTTTATAGGGTGTAGCTCTATTATATTACCAGCT
TTGTTATACGCGATGCATTCAAACAAAAAACAATTCGAACGTGTATTTGGAAAAGGGAGA
```

AAAAAGCCAATGAAGGTAAAAAAAGGAGGCGGGAAAATATCTAGATTTTTTAAAGAAGTA
GACAAA---------------------------
>CH62 .
------------------------------TTAAGGAAAATTTCCCTGTGTTCTATTTTC
GTATTGGCATATCTCAGTTCTGCTAAGTGCGCGTACCGAGACGAATCTGACATATTGTAC
AGCGACGACTCCAGATCCGATTTAGCAAGCAACGGGGCACCCACCGACAGCTACGAATCT
TTAACAGCAAGTAGTGAGTCTCTAGCAGAAAGCAACGATGCACCCAGCAACAGCTATGAA
TCTTTTCCAGAAATTAGAGAAAATCTAACCGCAAGTGAGGAATCCCTAACATCATGTGAG
GAATCCCTAACAGGAAGTAATGAATCCCTAACAGGAAGTAATGAATCCCTAACAGGAAGT
AAT---------------------------------------------------------
---------------------------GAATCCCTAACAGGAAGTAATGAATCCCTAACA
GAAAGTAGAGAATCTCTAGAGGCCAGTAGAGAATCGCTAAGAGCAAGTAGAGAGTCTCTA
GCGGCCAGTAGAGAATCCCTGAACGACTTTTGTGGGAGCGAAGAATCAGTAGCATTCGAA
GGAGAGCCAAATGAAAAGACATTCATGGGAGACGTCTTAAGTGGTGGAGAATGTGAGAAT
AGTCTCTCAAGAGAAGATTTATTTCATATAGAAGTAGGATCCGAAGAATCGCTAGATGAT
GCCTCAAAATATAATTTCCAAAAGGATTTATCAACTAGCGATAATAGTTCATTCGAAGAT
GATCAGTCATTGAAAAGGGGACTCAAAAGGAACAGCTCAGTATCTAGTCTGGACAGCGAT
ATGGGAAGTTATAAAAATAAAAGTTATAGACACAGATTGGATATATATTCGGACCTACCT
AGAAGGCCAATACATGGGGATAATGCACCACAAGAAAAGGAGAAATGTCTGGATTTCAAA
GAACTTCTAGAGAGAGAAAGGAACAACCACCAAAGGAATAGTGTAAATATAAAATTAAAA
AATATATACGGAGAAGAACAATTGTATAATGTGAAGAAAGATAGCTTTATTGTAAAATTG
ATATACTATCTGCCGTTTGGCCCAATTGTTACAATGATAATGATAGGCTTTATTCTACTC
TGCTCAACGCCCTCGACTGGTTTTATTTTTATAGGGTGTAGCTCTATTATATTACCAGCT
TTGTTATACGCGATGCATTCAAACAAAAAACAATTCGAACGTGTATTTGGAAAAGGGAGA
AAAAAGCCAATGAAGGTAAAAAAAGGAGGCGGGAAAATATCTAGATTTTTTAAAGAAGTA
GACAAA---------------------------
>CH63_R .
------------------------------TTAAGGAAAATTTCCCTGTGTTCTATTTTC
GTATTGGCATATCTCAGTTCTGCTAAGTGCGCGTACCGAGACGAATCTGACATATTGTAC
AGCGACGACTCCAGATCCGATTTAGCAAGCAACGGGGCACCCACCGACAGCTACGAATCT
TTAACAGCAAGTAGTGAGTCTCTAGCAGAAAGCAACGATGCACCCAGCAACAGCTATGAA
TCTTTTCCAGAAATTAGAGAAAATCTAACCGCAAGTGAGGAATCCCTAACATCATGTGAG
GAATCCCTAACAGGAAGTAATGAATCCCTAACAGGAAGTAATGAATCCCTAACAGGAAGT
AAT---------------------------------------------------------
------------------------------------------------GAATCCCTAACA
GAAAGTAGAGAATCTCTAGAGGCCAGTAGAGAATCGCTAAGAGCAAGTAGAGAGTCTCTA
GCGGCCAGTAGAGAATCCCTGAACGACTTTTGTGGGAGCGAAGAATCAGTAGCATTCGAA
GGAGAGCCAAATGAAAAGACATTCATGGGAGACGTCTTAAGTGGTGGAGAATGTGAGAAT
AGTCTCTCAAGAGAAGATTTATTTCATATAGAAGTAGGATCCGAAGAATCGCTAGATGAT
GCCTCAAAATATAATTTCCAAAAGGATTTATCAACTAGCGATAATAGTTTATTCGAAGAT
GATCAGTCATTGAAAAGGGGACTCAAAAGGAACAGCTCAGTATCTAGTCTGGACAGCGAT
ATGGGAAGTTATAAAAATGGAAGATCTAGAGACAGATTGGATATATATTCGGACCTACCT
AGAAGGCTAATACATGGGGATAATGCACCACAAGAAAAGGAGAAATGTCTGGATTTCAAA
GAACTTCTAGAGAGAGAAAGGAACAACCACCAAAGGAATAGTGTAAATATAAAATTAAAA
AATATATACGGAGAAGAACAATTGTATAATGTGAAGAAAGATAGCTTTATTGTAAAATTG
ATATACTATCTGCCGTTTGGCCCAATTGTTACAATGATAATGATAGGCTTTATTCTACTC

TGGTCAACGCCCTGGACTGGTTTTATTTTTATAGGGTGTAGCTCTATTATATTACCAGCT
TTGTTATACGCGATGCATTCAAACAAAAAACAATTCGAACGTGTATTTGGAAAAGGGAGA
AAAAAGCCAATGAAGGTAAAAAAAGGAGGCGGGAAAATATCTAGATTTTTTAAAGAAGTA
GACAAA---------------------------
>CH64 .
------------------------------TTAAGGAAAATTTCCCTGTGTTCTATTTTC
GTATTGGCATATCTCAGTTCTGCTAAGTGCGCGTACCGAGACGAATCTGACATATTGTAC
AGCGACGACTCCAGATCCGATTTAGCAAGCAACGGGGCACCCACCGACAGCTACGAATCT
TTAACAGCAAGTAGTGAGTCTCTAGCAGAAAGCAACGATGCACCCAGCAACAGCTATGAA
TCTTTTCCAGAAATTAGAGAAAATCTAACCGCAAGTGAGGAATCCCTAACATCATGTGAG
GAATCCCTAACAGGAAGTAATGAATCCCTAACAGGAAGTAATGAATCCCTAACAGGAAGT
AAT---------------------------------------------------------
------------------------------------------------GAATCCCTAACA
GAAAGTAGAGAATCTCTAGAGGCCAGTAGAGAATCGCTAAGAGCAAGTAGAGAGTCTCTA
GCGGCCAGTAGAGAATCCCTGAACGACTTTTGTGGGAGCGAAGAATCAGTAGCATTCGAA
GGAGAGCCAAATGAAAAGACATTCATGGGAGACGTCTTAAGTGGTGGAGAATGTGAGAAT
AGTCTCTCAAGAGAAGATTTATTTCATATAGAAGTAGGATCCGAAGAATCGCTAGATGAT
GCCTCAAAATATAATTTCCAAAAGGATTTATCAACTAGCGATAATAGTTTATTCGAAGAT
GATCAGTCATTGAAAAGGGGACTCAAAAGGAACAGCTCAGTATCTAGTCTGGACAGCGAT
ATGGGAAGTTATAAAAATGGAAGATCTAGAGACAGATTGGATATATATTCGGACCTACCT
AGAAGGCTAATACATGGGGATAATGCACCACAAGAAAAGGAGAAATGTCTGGATTTCAAA
GAACTTCTAGAGAGAGAAAGGAACAACCACCAAAGGAATAGTGTAAATATAAAATTAAAA
AATATATACGGAGAAGAACAATTGTATAATGTGAAGAAAGATAGCTTTATTGTAAAATTG
ATATACTATCTGCCGTTTGGCCCAATTGTTACAATGATAATGATAGGCTTTATTCTACTC
TGGTCAACGCCCTGGACTGGTTTTATTTTTATAGGGTGTAGCTCTATTATATTACCAGCT
TTGTTATACGCGATGCATTCAAACAAAAAACAATTCGAACGTGTATTTGGAAAAGGGAGA
AAAAAGCCAATGAAGGTAAAAAAAGGAGGCGGGAAAATATCTAGATTTTTTAAAGAAGTA
GACAAA---------------------------
>CH68 .
------------------------------TTAAGGAAAATTTCCCTGTGTTCTATTTTC
GTATTGGCATATCTCAGTTCTGCTAAGTGCGCGTACCGAGACGAATCTGACATATTGTAC
AGCGACGACTCCAGATCCGATTTAGCAAGCAACGGGGCACCCACCGACAGCTACGAATCT
TTAACAGCAAGTAGTGAGTCTCTAGCAGAAAGCAACGATGCACCCAGCAACAGCTATGAA
TCTTTTCCAGAAATTAGAGAAAATCTAACCGCAAGTGAGGAATCCCTAACA---------
------------GGAAGTAATGAATCCCTAACAGGAAGTAATGAATCCCTAACAGAAAGT
AAT------------------------------------------GAATCCCTAACAGGA
AGTAATGAATCCCTAACAGGAAGTAATGAATCCCTAACAGGAAGTAATGAATCCCTAACA
GAAAGTAGAGAATCTCTAGAGGCCAGTAGAGAATCGCTAAGAGCAAGTAGAGAGTCTCTA
GCGGCCAGTAGAGAATCCCTGAACGACTTTTGTGGGAGCGAAGAATCAGTAGCATGCGAA
GGAGAGCCAAATGAAAAGACATTCATGGGAGACGTCTTAAGTGGTGGAGAATGTGAGAAT
AGTCTCTCAAGAGAAGATTTATTTCATATAGAAGTAGGATCCGAAGAATCGCTAGATGAT
GCCTCAAAATATAATTTCCAAAAGGATTTATCAACTAGCGATAATAGTTTATTCGAAGAT
GATCAGTCATTGAAAAGGGGACTCAAAAGGAACAGCTCAGTATCTAGTCTGGACAGCGAT
ATGGGAAGTTATAAAAATGGAAGATCTAGAGACAGATTGGATATATATTCGGACCTACCT
AGAAGGCTAATACATGGGGATAATGCACCACAAGAAAAGGAGAAATGTCTGGATTTCAAA
GAACTTCTAGAGAGAGAAAGGAACAACCACCAAAGGAATAGTGTAAATATAAAATTAAAA
AATATATACGGAGAAGAACAATTGTATAATGTGAAGAAAGATAGCTTTATTGTAAAATTG
ATATACTATCTGCCGTTTGGCCCAATTGTTACAATGATAATGATAGGTTTTTTTCTACTC
TGCTGGACCCCCTCGATTGGTTTTACTTTTATAGGGTGTAGCTCTATTATATTACCAGCT
TTGTTATACGCGATGCATTCAAACAAAAAACAATTCGAACGTGTATTTGGAAAAGGGAGA

```
AAAAAGCCAATGAAGGTAAAAAAAGGAGGCGGGAAAATATCTAGATTTTTTAAAGAAGTA
GACAAA---------------------------
>CH67 .
------------------------------TTAAGGAAAATTTCCCTGTGTTCTATTTTC
GTATTGGCATATCTCAGTTCTGCTAAGTGCGCGTACCGAGACGAATCTGACATATTGTAC
AGCGACGACTCCAGATCCGATTTAGCAAGCAACGGGGCACCCACCGACAGCTACGAATCT
TTAACAGCAAGTAGTGAGTCTCTAGCAGAAAGCAACGATGCACCCAGCAACAGCTATGAA
TCTTTTCCAGAAATTAGAGAAAATCTAACCGCAAGTGAGGAATCCCTAACATCATGTGAG
GAATCCCTAACAGGAAGTAATGAATCCCTAACAGGAAGTAATGAATCCCTAACAGGAAGT
AAT---------------------------------------------------------
---------------------------GAATCCCTAACAGGAAGTAATGAATCCCTAACA
GAAAGTAGAGAATCTCTAGAGGCCAGTAGAGAATCGCTAAGAGCAAGTAGAGAGTCTCTA
GCGGCCAGTAGAGAATCCCTGAACGACTTTTGTGGGAGCGAAGAATCAGTAGCATTCGAA
GGAGAGCCAAATGAAAAGACATTCATGGGAGACGTCTTAAGTGGTGGAGAATGTGAGAAT
AGTCTCTCAAGAGAAGATTTATTTCATATAGAAGTAGGATCCGAAGAATCGCTAGATGAT
GCCTCAAAATATAATTTCCAAAAGGATTTATCAACTAGCGATAATAGTTCATTCGAAGAT
GATCAGTCATTGAAAAGGGGACTCAAAAGGAACAGCTCAGTATCTAGTCTGGACAGCGAT
ATGGGAAGTTATAAAAATAAAAGTTATAGACACAGATTGGATATATATTCGGACCTACCT
AGAAGGCCAATACATGGGGATAATGCACCACAAGAAAAGGAGAAATGTCTGGATTTCAAA
GAACTTCTAGAGAGAGAAAGGAACAACCACCAAAGGAATAGTGTAAATATAAAATTAAAA
AATATATACGGAGAAGAACAATTGTATAATGTGAAGAAAGATAGCTTTATTGTAAAATTG
ATATACTATCTGCCGTTTGGCCCAATTGTTACAATGATAATGATAGGCTTTATTCTACTC
TGCTCAACGCCCTCGACTGGTTTTATTTTTATAGGGTGTAGCTCTATTATATTACCAGCT
TTGTTATACGCGATGCATTCAAACAAAAAACAATTCGAACGTGTATTTGGAAAAGGGAGA
AAAAAGCCAATGAAGGTAAAAAAAGGAGGCGGGAAAATATCTAGATTTTTTAAAGAAGTA
GACAAA---------------------------
>CH70 .
------------------------------TTAAGGAAAATTTCCCTGTGTTCTATTTTC
GTATTGGCATATCTCAGTTCTGCTAAGTGCGCGTACCGAGACGAATCTGACATATTGTAC
AGCGACGACTCCAGATCCGATTTAGCAAGCAACGGGGCACCCACCGACAGCTACGAATCT
TTAACAGCAAGTAGTGAGTCTCTAGCAGAAAGCAACGATGCACCCAGCAACAGCTATGAA
TCTTTTCCAGAAATTAGAGAAAATCTAACCGCAAGTGAGGAATCCCTAACA---------
------------GGAAGTAATGAATCCCTAACAGGAAGTAATGAATCCCTAACAGAAAGT
AAT------------------------------------------GAATCCCTAACAGGA
AGTAATGAATCCCTAACAGGAAGTAATGAATCCCTAACAGGAAGTAATGAATCCCTAACA
GAAAGTAGAGAATCTCTAGAGGCCAGTAGAGAATCGCTAAGAGCAAGTAGAGAGTCTCTA
GCGGCCAGTAGAGAATCCCTGAACGACTTTTGTGGGAGCGAAGAATCAGTAGCATGCGAA
GGAGAGCCAAATGAAAAGACATTCATGGGAGACGTCTTAAGTGGTGGAGAATGTGAGAAT
AGTCTCTCAAGAGAAGATTTATTTCATATAGAAGTAGGATCCGAAGAATCGCTAGATGAT
GCCTCAAAATATAATTTCCAAAAGGATTTATCAACTAGCGATAATAGTTTATTCGAAGAT
GATCAGTCATTGAAAAGGGGACTCAAAAGGAACAGCTCAGTATCTAGTCTGGACAGCGAT
ATGGGAAGTTATAAAAATGGAAGATCTAGAGACAGATTGGATATATATTCGGACCTACCT
AGAAGGCTAATACATGGGGATAATGCACCACAAGAAAAGGAGAAATGTCTGGATTTCAAA
GAACTTCTAGAGAGAGAAAGGAACAACCACCAAAGGAATAGTGTAAATATAAAATTAAAA
AATATATACGGAGAAGAACAATTGTATAATGTGAAGAAAGATAGCTTTATTGTAAAATTG
ATATACTATCTGCCGTTTGGCCCAATTGTTACAATGATAATGATAGGTTTTTTTCTACTC
```

TGCTGGACCCCCTCGATTGGTTTTACTTTTATAGGGTGTAGCTCTATTATATTACCAGCT
TTGTTATACGCGATGCATTCAAACAAAAAACAATTCGAACGTGTATTTGGAAAAGGGAGA
AAAAAGCCAATGAAGGTAAAAAAAGGAGGCGGGAAAATATCTAGATTTTTTAAAGAAGTA
GACAAA---------------------------
>CH76 .
------------------------------TTAAGGAAAATTTCCCTGTGTTCTATTTTC
GTATTGGCATATCTCAGTTCTGCTAAGTGCGCGTACCGAGACGAATCTGACATATTGTAC
AGCGACGACTCCAGATCCGATTTAGCAAGCAACGGGGCACCCACCGACAGCTACGAATCT
TTAACAGCAAGTAGTGAGTCTCTAGCAGAAAGCAACGATGCACCCAGCAACAGCTATGAA
TCTTTTCCAGAAATTAGAGAAAATCTAACCGCAAGTGAGGAATCCCTAACATCATGTGAG
GAATCCCTAACAGGAAGTAATGAATCCCTAACAGGAAGTAATGAATCCCTAACAGGAAGT
AAT---------------------------------------------------------
---------------------------GAATCCCTAACAGGAAGTAATGAATCCCTAACA
GAAAGTAGAGAATCTCTAGAGGCCAGTAGAGAATCGCTAAGAGCAAGTAGAGAGTCTCTA
GCGGCCAGTAGAGAATCCCTGAACGACTTTTGTGGGAGCGAAGAATCAGTAGCATTCGAA
GGAGAGCCAAATGAAAAGACATTCATGGGAGACGTCTTAAGTGGTGGAGAATGTGAGAAT
AGTCTCTCAAGAGAAGATTTATTTCATATAGAAGTAGGATCCGAAGAATCGCTAGATGAT
GCCTCAAAATATAATTTCCAAAAGGATTTATCAACTAGCGATAATAGTTCATTCGAAGAT
GATCAGTCATTGAAAAGGGGACTCAAAAGGAACAGCTCAGTATCTAGTCTGGACAGCGAT
ATGGGAAGTTATAAAAATAAAAGTTATAGACACAGATTGGATATATATTCGGACCTACCT
AGAAGGCCAATACATGGGGATAATGCACCACAAGAAAAGGAGAAATGTCTGGATTTCAAA
GAACTTCTAGAGAGAGAAAGGAACAACCACCAAAGGAATAGTGTAAATATAAAATTAAAA
AATATATACGGAGAAGAACAATTGTATAATGTGAAGAAAGATAGCTTTATTGTAAAATTG
ATATACTATCTGCCGTTTGGCCCAATTGTTACAATGATAATGATAGGCTTTATTCTACTC
TGCTCAACGCCCTCGACTGGTTTTATTTTTATAGGGTGTAGCTCTATTATATTACCAGCT
TTGTTATACGCGATGCATTCAAACAAAAAACAATTCGAACGTGTATTTGGAAAAGGGAGA
AAAAAGCCAATGAAGGTAAAAAAAGGAGGCGGGAAAATATCTAGATTTTTTAAAGAAGTA
GACAAA---------------------------
>CH79 .
------------------------------TTAAGGAAAATTTCCCTGTGTTCTATTTTC
GTATTGGCATATCTCAGTTCTGCTAAGTGCGCGTACCGAGACGAATCTGACATATTGTAC
AGCGACGACTCCAGATCCGATTTAGCAAGCAACGGGGCACCCACCGACAGCTACGAATCT
TTAACAGCAAGTAGTGAGTCTCTAGCAGAAAGCAACGATGCACCCAGCAACAGCTATGAA
TCTTTTCCAGAAATTAGAGAAAATCTAACCGCAAGTGAGGAATCCCTAACATCATGTGAG
GAATCCCTAACAGGAAGTAATGAATCCCTAACAGGAAGTAATGAATCCCTAACAGGAAGT
AAT---------------------------------------------------------
------------------------------------------------GAATCCCTAACA
GAAAGTAGAGAATCTCTAGAGGCCAGTAGAGAATCGCTAAGAGCAAGTAGAGAGTCTCTA
GCGGCCAGTAGAGAATCCCTGAACGACTTTTGTGGGAGCGAAGAATCAGTAGCATTCGAA
GGAGAGCCAAATGAAAAGACATTCATGGGAGACGTCTTAAGTGGTGGAGAATGTGAGAAT
AGTCTCTCAAGAGAAGATTTATTTCATATAGAAGTAGGATCCGAAGAATCGCTAGATGAT
GCCTCAAAATATAATTTCCAAAAGGATTTATCAACTAGCGATAATAGTTTATTCGAAGAT
GATCAGTCATTGAAAAGGGGACTCAAAAGGAACAGCTCAGTATCTAGTCTGGACAGCGAT
ATGGGAAGTTATAAAAATGGAAGATCTAGAGACAGATTGGATATATATTCGGACCTACCT
AGAAGGCTAATACATGGGGATAATGCACCACAAGAAAAGGAGAAATGTCTGGATTTCAAA
GAACTTCTAGAGAGAGAAAGGAACAACCACCAAAGGAATAGTGTAAATATAAAATTAAAA
AATATATACGGAGAAGAACAATTGTATAATGTGAAGAAAGATAGCTTTATTGTAAAATTG
ATATACTATCTGCCGTTTGGCCCAATTGTTACAATGATAATGATAGGCTTTATTCTACTC
TGGTCAACGCCCTGGACTGGTTTTATTTTTATAGGGTGTAGCTCTATTATATTACCAGCT
TTGTTATACGCGATGCATTCAAACAAAAAACAATTCGAACGTGTATTTGGAAAAGGGAGA

AAAAAGCCAATGAAGGTAAAAAAAGGAGGCGGGAAAATATCTAGATTTTTTAAAGAAGTA
GACAAA---------------------------
>CH80 .
------------------------------TTAAGGAAAATTTCCCTGTGTTCTATTTTC
GTATTGGCATATCTCAGTTCTGCTAAGTGCGCGTACCGAGACGAATCTGACATATTGTAC
AGCGACGACTCCAGATCCGATTTAGCAAGCAACGGGGCACCCACCGACAGCTACGAATCT
TTAACAGCAAGTAGTGAGTCTCTAGCAGAAAGCAACGATGCACCCAGCAACAGCTATGAA
TCTTTTCCAGAAATTAGAGAAAATCTAACCGCAAGTGAGGAATCCCTAACA---------
------------GGAAGTAATGAATCCCTAACAGGAAGTAATGAATCCCTAACAGAAAGT
AAT------------------------------------------GAATCCCTAACAGGA
AGTAATGAATCCCTAACAGGAAGTAATGAATCCCTAACAGGAAGTAATGAATCCCTAACA
GAAAGTAGAGAATCTCTAGAGGCCAGTAGAGAATCGCTAAGAGCAAGTAGAGAGTCTCTA
GCGGCCAGTAGAGAATCCCTGAACGACTTTTGTGGGAGCGAAGAATCAGTAGCATTCGAA
GGAGAGCCAAATGAAAAGACATTCATGGGAGACGTCTTAAGTGGTGGAGAATGTGAGAAT
AGTCTCTCAAGAGAAGATTTATTTCATATAGAAGTAGGATCCGAAGAATCGCTAGATGAT
GCCTCAAAATATAATTTCCAAAAGGATTTATCAACTAGCGATAATAGTTTATTCGAAGAT
GATCAGTCATTGAAAAGGGGACTCAAAAGGAACAGCTCAGTATCTAGTCTGGACAGCGAT
ATGGGAAGTTATAAAAATGGAAGATCTAGAGACAGATTGGATATATATTCGGACCTACCT
AGAAGGCTAATACATGGGGATAATGCACCACAAGAAAAGGAGAAATGTCTGGATTTCAAA
GAACTTCTAGAGAGAGAAAGGAACAACCACCAAAGGAATAGTGTAAATATAAAATTAAAA
AATATATACGGAGAAGAACAATTGTATAATGTGAAGAAAGATAGCTTTATTGTAAAATTG
ATATACTATCTGCCGTTTGGCCCAATTGTTACAATGATAATGATAGGTTTTTTTCTACTC
TGCTGGACCCCCTCGATTGGTTTTATTTTTATAGGGTGTAGCTCTATTATATTACCAGCT
TTGTTATACGCGATGCATTCAAACAAAAAACAATTCGAACGTGTATTTGGAAAAGGGAGA
AAAAAGCCAATGAAGGTAAAAAAAGGAGGCGGGAAAATATCTAGATTTTTTAAAGAAGTA
GACAAA---------------------------
>CH84 .
------------------------------TTAAGGAAAATTTCCCTGTGTTCTATTTTC
GTATTGGCATATCTCAGTTCTGCTAAGTGCGCGTACCGAGACGAATCTGACATATTGTAC
AGCGACGACTCCAGATCCGATTTAGCAAGCAACGGGGCACCCACCGACAGCTACGAATCT
TTAACAGCAAGTAGTGAGTCTCTAGCAGAAAGCAACGATGCACCCAGCAACAGCTATGAA
TCTTTTCCAGAAATTAGAGAAAATCTAACCGCAAGTGAGGAATCCCTAACA---------
------------GGAAGTAATGAATCCCTAACAGGAAGTAATGAATCCCTAACAGAAAGT
AAT------------------------------------------GAATCCCTAACAGGA
AGTAATGAATCCCTAACAGGAAGTAATGAATCCCTAACAGGAAGTAATGAATCCCTAACA
GAAAGTAGAGAATCTCTAGAGGCCAGTAGAGAATCGCTAAGAGCAAGTAGAGAGTCTCTA
GCGGCCAGTAGAGAATCCCTGAACGACTTTTGTGGGAGCGAAGAATCAGTAGCATTCGAA
GGAGAGCCAAATGAAAAGACATTCATGGGAGACGTCTTAAGTGGTGGAGAATGTGAGAAT
AGTCTCTCAAGAGAAGATTTATTTCATATAGAAGTAGGATCCGAAGAATCGCTAGATGAT
GCCTCAAAATATAATTTCCAAAAGGATTTATCAACTAGCGATAATAGTTTATTCGAAGAT
GATCAGTCATTGAAAAGGGGACTCAAAAGGAACAGCTCAGTATCTAGTCTGGACAGCGAT
ATGGGAAGTTATAAAAATGGAAGATCTAGAGACAGATTGGATATATATTCGGACCTACCT
AGAAGGCTAATACATGGGGATAATGCACCACAAGAAAAGGAGAAATGTCTGGATTTCAAA
GAACTTCTAGAGAGAGAAAGGAACAACCACCAAAGGAATAGTGTAAATATAAAATTAAAA
AATATATACGGAGAAGAACAATTGTATAATGTGAAGAAAGATAGCTTTATTGTAAAATTG
ATATACTATCTGCCGTTTGGCCCAATTGTTACAATGATAATGATAGGTTTTTTTCTACTC

TGCTGGACCCCCTCGATTGGTTTTATTTTTATAGGGTGTAGCTCTATTATATTACCAGCT
TTGTTATACGCGATGCATTCAAACAAAAAACAATTCGAACGTGTATTTGGAAAAGGGAGA
AAAAAGCCAATGAAGGTAAAAAAAGGAGGCGGGAAAATATCTAGATTTTTTAAAGAAGTA
GACAAA---------------------------
>CH84_1 .
------------------------------TTAAGGAAAATTTCCCTGTGTTCTATTTTC
GTATTGGCATATCTCAGTTCTGCTAAGTGCGCGTACCGAGACGAATCTGACATATTGTAC
AGCGACGACTCCAGATCCGATTTAGCAAGCAACGGGGCACCCACCGACAGCTACGAATCT
TTAACAGCAAGTAGTGAGTCTCTAGCAGAAAGCAACGATGCACCCAGCAACAGCTATGAA
TCTTTTCCAGAAATTAGAGAAAATCTAACCGCAAGTGAGGAATCCCTAACA---------
------------GGAAGTAATGAATCCCTAACAGGAAGTAATGAATCCCTAACAGAAAGT
AAT------------------------------------------GAATCCCTAACAGGA
AGTAATGAATCCCTAACAGGAAGTAATGAATCCCTAACAGGAAGTAATGAATCCCTAACA
GAAAGTAGAGAATCTCTAGAGGCCAGTAGAGAATCGCTAAGAGCAAGTAGAGAGTCTCTA
GCGGCCAGTAGAGAATCCCTGAACGACTTTTGTGGGAGCGAAGAATCAGTAGCATTCGAA
GGAGAGCCAAATGAAAAGACATTCATGGGAGACGTCTTAAGTGGTGGAGAATGTGAGAAT
AGTCTCTCAAGAGAAGATTTATTTCATATAGAAGTAGGATCCGAAGAATCGCTAGATGAT
GCCTCAAAATATAATTTCCAAAAGGATTTATCAACTAGCGATAATAGTTTATTCGAAGAT
GATCAGTCATTGAAAAGGGGACTCAAAAGGAACAGCTCAGTATCTAGTCTGGACAGCGAT
ATGGGAAGTTATAAAAATGGAAGATCTAGAGACAGATTGGATATATATTCGGACCTACCT
AGAAGGCTAATACATGGGGATAATGCACCACAAGAAAAGGAGAAATGTCTGGATTTCAAA
GAACTTCTAGAGAGAGAAAGGAACAACCACCAAAGGAATAGTGTAAATATAAAATTAAAA
AATATATACGGAGAAGAACAATTGTATAATGTGAAGAAAGATAGCTTTATTGTAAAATTG
ATATACTATCTGCCGTTTGGCCCAATTGTTACAATGATAATGATAGGTTTTTTTCTACTC
TGCTGGACCCCCTCGATTGGTTTTATTTTTATAGGGTGTAGCTCTATTATATTACCAGCT
TTGTTATACGCGATGCATTCAAACAAAAAACAATTCGAACGTGTATTTGGAAAAGGGAGA
AAAAAGCCAATGAAGGTAAAAAAAGGAGGCGGGAAAATATCTAGATTTTTTAAAGAAGTA
GACAAA---------------------------
>CH85_R .
------------------------------TTAAGGAAAATTTCCCTGTGTTCTATTTTC
GTATTGGCATATCTCAGTTCTGCTAAGTGCGCGTACCGAGACGAATCTGACATATTGTAC
AGCGACGACTCCAGATCCGATTTAGCAAGCAACGGGGCACCCACCGACAGCTACGAATCT
TTAACAGCAAGTAGTGAGTCTCTAGCAGAAAGCAACGATGCACCCAGCAACAGCTATGAA
TCTTTTCCAGAAATTAGAGAAAATCTAACCGCAAGTGAGGAATCCCTAACATCATGTGAG
GAATCCCTAACAGGAAGTAATGAATCCCTAACAGGAAGTAATGAATCCCTAACAGGAAGT
AAT---------------------------------------------------------
------------------------------------------------GAATCCCTAACA
GAAAGTAGAGAATCTCTAGAGGCCAGTAGAGAATCGCTAAGAGCAAGTAGAGAGTCTCTA
GCGGCCAGTAGAGAATCCCTGAACGACTTTTGTGGGAGCGAAGAATCAGTAGCATTCGAA
GGAGAGCCAAATGAAAAGACATTCATGGGAGACGTCTTAAGTGGTGGAGAATGTGAGAAT
AGTCTCTCAAGAGAAGATTTATTTCATATAGAAGTAGGATCCGAAGAATCGCTAGATGAT
GCCTCAAAATATAATTTCCAAAAGGATTTATCAACTAGCGATAATAGTTCATTCGAAGAT
GATCAGTCATTGAAAAGGGGACTCAAAAGGAACAGCTCAGTATCTAGTCTGGACAGCGAT
ATGGGAAGTTATAAAAATAAAAGTTATAGACACAGATTGGATATATATTCGGACCTACCT
AGAAGGCCAATACATGGGGATAATGCACCACAAGAAAAGGAGAAATGTCTGGATTTCAAA
GAACTTCTAGAGAGAGAAAGGAACAACCACCAAAGGAATAGTGTAAATATAAAATTAAAA
AATATATACGGAGAAGAACAATTGTATAATGTGAAGAAAGATAGCTTTATTGTAAAATTG
ATATACTATCTGCCGTTTGGCCCAATTGTTACAATGATAATGATAGGCTTTATTCTACTC
TGGTCAACGCCCTGGACTGGTTTTATTTTTATAGGGTGTAGCTCTATTATATTACCAGCT
TTGTTATACGCGATGCATTCAAACAAAAAACAATTCGAACGTGTATTTGGAAAAGGGAGA

AAAAAGCCAATGAAGGTAAAAAAAGGAGGCGGGAAAATATCTAGATTTTTTAAAGAAGTA
GACAAA---------------------------
>CH87 .
------------------------------TTAAGGAAAATTTCCCTGTGTTCTATTTTC
GTATTGGCATATCTCAGTTCTGCTAAGTGCGCGTACCGAGACGAATCTGACATATTGTAC
AGCGACGACTCCAGATCCGATTTAGCAAGCAACGGGGCACCCACCGACAGCTACGAATCT
TTAACAGCAAGTAGTGAGTCTCTAGCAGAAAGCAACGATGCACCCAGCAACAGCTATGAA
TCTTTTCCAGAAATTAGAGAAAATCTAACCGCAAGTGAGGAATCCCTAACATCATGTGAG
GAATCCCTAACAGGAAGTAATGAATCCCTAACAGGAAGTAATGAATCCCTAACAGGAAGT
AAT---------------------------------------------------------
---------------------------GAATCCCTAACAGGAAGTAATGAATCCCTAACA
GAAAGTAGAGAATCTCTAGAGGCCAGTAGAGAATCGCTAAGAGCAAGTAGAGAGTCTCTA
GCGGCCAGTAGAGAATCCCTGAACGACTTTTGTGGGAGCGAAGAATCAGTAGCATTCGAA
GGAGAGCCAAATGAAAAGACATTCATGGGAGACGTCTTAAGTGGTGGAGAATGTGAGAAT
AGTCTCTCAAGAGAAGATTTATTTCATATAGAAGTAGGATCCGAAGAATCGCTAGATGAT
GCCTCAAAATATAATTTCCAAAAGGATTTATCAACTAGCGATAATAGTTCATTCGAAGAT
GATCAGTCATTGAAAAGGGGACTCAAAAGGAACAGCTCAGTATCTAGTCTGGACAGCGAT
ATGGGAAGTTATAAAAATAAAAGTTATAGACACAGATTGGATATATATTCGGACCTACCT
AGAAGGCCAATACATGGGGATAATGCACCACAAGAAAAGGAGAAATGTCTGGATTTCAAA
GAACTTCTAGAGAGAGAAAGGAACAACCACCAAAGGAATAGTGTAAATATAAAATTAAAA
AATATATACGGAGAAGAACAATTGTATAATGTGAAGAAAGATAGCTTTATTGTAAAATTG
ATATACTATCTGCCGTTTGGCCCAATTGTTACAATGATAATGATAGGCTTTATTCTACTC
TGCTCAACGCCCTCGACTGGTTTTATTTTTATAGGGTGTAGCTCTATTATATTACCAGCT
TTGTTATACGCGATGCATTCAAACAAAAAACAATTCGAACGTGTATTTGGAAAAGGGAGA
AAAAAGCCAATGAAGGTAAAAAAAGGAGGCGGGAAAATATCTAGATTTTTTAAAGAAGTA
GACAAA---------------------------
>CH88 .
------------------------------TTAAGGAAAATTTCCCTGTGTTCTATTTTC
GTATTGGCATATCTCAGTTCTGCTAAGTGCGCGTACCGAGACGAATCTGACATATTGTAC
AGCGACGACTCCAGATCCGATTTAGCAAGCAACGGGGCACCCACCGACAGCTACGAATCT
TTAACAGCAAGTAGTGAGTCTCTAGCAGAAAGCAACGATGCACCCAGCAACAGCTATGAA
TCTTTTCCAGAAATTAGAGAAAATCTAACCGCAAGTGAGGAATCCCTAACATCATGTGAG
GAATCCCTAACAGGAAGTAATGAATCCCTAACAGGAAGTAATGAATCCCTAACAGGAAGT
AAT---------------------------------------------------------
------------------------------------------------GAATCCCTAACA
GAAAGTAGAGAATCTCTAGAGGCCAGTAGAGAATCGCTAAGAGCAAGTAGAGAGTCTCTA
GCGGCCAGTAGAGAATCCCTGAACGACTTTTGTGGGAGCGAAGAATCAGTAGCATTCGAA
GGAGAGCCAAATGAAAAGACATTCATGGGAGACGTCTTAAGTGGTGGAGAATGTGAGAAT
AGTCTCTCAAGAGAAGATTTATTTCATATAGAAGTAGGATCCGAAGAATCGCTAGATGAT
GCCTCAAAATATAATTTCCAAAAGGATTTATCAACTAGCGATAATAGTTCATTCGAAGAT
GATCAGTCATTGAAAAGGGGACTCAAAAGGAACAGCTCAGTATCTAGTCTGGACAGCGAT
ATGGGAAGTTATAAAAATAAAAGTTATAGACACAGATTGGATATATATTCGGACCTACCT
AGAAGGCCAATACATGGGGATAATGCACCACAAGAAAAGGAGAAATGTCTGGATTTCAAA
GAACTTCTAGAGAGAGAAAGGAACAACCACCAAAGGAATAGTGTAAATATAAAATTAAAA
AATATATACGGAGAAGAACAATTGTATAATGTGAAGAAAGATAGCTTTATTGTAAAATTG
ATATACTATCTGCCGTTTGGCCCAATTGTTACAATGATAATGATAGGCTTTATTCTACTC

TGCTCAACGCCCTCGACTGGTTTTATTTTTATAGGGTGTAGCTCTATTATATTACCAGCT
TTGTTATACGCGATGCATTCAAACAAAAAACAATTCGAACGTGTATTTGGAAAAGGGAGA
AAAAAGCCAATGAAGGTAAAAAAAGGAGGCGGGAAAATATCTAGATTTTTTAAAGAAGTA
GACAAA---------------------------
>CH88_1 .
------------------------------TTAAGGAAAATTTCCCTGTGTTCTATTTTC
GTATTGGCATATCTCAGTTCTGCTAAGTGCGCGTACCGAGACGAATCTGACATATTGTAC
AGCGACGACTCCAGATCCGATTTAGCAAGCAACGGGGCACCCACCGACAGCTACGAATCT
TTAACAGCAAGTAGTGAGTCTCTAGCAGAAAGCAACGATGCACCCAGCAACAGCTATGAA
TCTTTTCCAGAAATTAGAGAAAATCTAACCGCAAGTGAGGAATCCCTAACATCATGTGAG
GAATCCCTAACAGGAAGTAATGAATCCCTAACAGGAAGTAATGAATCCCTAACAGGAAGT
AAT---------------------------------------------------------
------------------------------------------------GAATCCCTAACA
GAAAGTAGAGAATCTCTAGAGGCCAGTAGAGAATCGCTAAGAGCAAGTAGAGAGTCTCTA
GCGGCCAGTAGAGAATCCCTGAACGACTTTTGTGGGAGCGAAGAATCAGTAGCATTCGAA
GGAGAGCCAAATGAAAAGACATTCATGGGAGACGTCTTAAGTGGTGGAGAATGTGAGAAT
AGTCTCTCAAGAGAAGATTTATTTCATATAGAAGTAGGATCCGAAGAATCGCTAGATGAT
GCCTCAAAATATAATTTCCAAAAGGATTTATCAACTAGCGATAATAGTTCATTCGAAGAT
GATCAGTCATTGAAAAGGGGACTCAAAAGGAACAGCTCAGTATCTAGTCTGGACAGCGAT
ATGGGAAGTTATAAAAATAAAAGTTATAGACACAGATTGGATATATATTCGGACCTACCT
AGAAGGCCAATACATGGGGATAATGCACCACAAGAAAAGGAGAAATGTCTGGATTTCAAA
GAACTTCTAGAGAGAGAAAGGAACAACCACCAAAGGAATAGTGTAAATATAAAATTAAAA
AATATATACGGAGAAGAACAATTGTATAATGTGAAGAAAGATAGCTTTATTGTAAAATTG
ATATACTATCTGCCGTTTGGCCCAATTGTTACAATGATAATGATAGGCTTTATTCTACTC
TGCTCAACGCCCTCGACTGGTTTTATTTTTATAGGGTGTAGCTCTATTATATTACCAGCT
TTGTTATACGCGATGCATTCAAACAAAAAACAATTCGAACGTGTATTTGGAAAAGGGAGA
AAAAAGCCAATGAAGGTAAAAAAAGGAGGCGGGAAAATATCTAGATTTTTTAAAGAAGTA
GACAAA---------------------------
>CH89 .
------------------------------TTAAGGAAAATTTCCCTGTGTTCTATTTTC
GTATTGGCATATCTCAGTTCTGCTAAGTGCGCGTACCGAGACGAATCTGACATATTGTAC
AGCGACGACTCCAGATCCGATTTAGCAAGCAACGGGGCACCCACCGACAGCTACGAATCT
TTAACAGCAAGTAGTGAGTCTCTAGCAGAAAGCAACGATGCACCCAGCAACAGCTATGAA
TCTTTTCCAGAAATTAGAGAAAATCTAACCGCAAGTGAGGAATCCCTAACATCATGTGAG
GAATCCCTAACAGGAAGTAATGAATCCCTAACAGGAAGTAATGAATCCCTAACAGGAAGT
AAT---------------------GAATCCCTAACAGGAAGTAATGAATCCCTAACAGGA
AGTAATGAATCCCTAACAGGAAGTAATGAATCCCTAACAGGAAGTAATGAATCCCTAACA
GAAAGTAGAGAATCTCTAGAGGCCAGTAGAGAATCGCTAAGAGCAAGTAGAGAGTCTCTA
GCGGCCAGTAGAGAATCCCTGAACGACTTTTGTGGGAGCGAAGAATCAGTAGCATTCGAA
GGAGAGCCAAATGAAAAGACATTCATGGGAGACGTCTTAAGTGGTGGAGAATGTGAGAAT
AGTCTCTCAAGAGAAGATTTATTTCATATAGAAGTAGGATCCGAAGAATCGCTAGATGAT
GCCTCAAAATATAATTTCCAAAAGGATTTATCAACTAGCGATAATAGTTTATTCGAAGAT
GATCAGTCATTGAAAAGGGGACTCAAAAGGAACAGCTCAGTATCTAGTCTGGACAGCGAT
ATGGGAAGTTATAAAAATGGAAGATCTAGAGACAGATTGGATATATATTCGGACCTACCT
AGAAGGCTAATACATGGGGATAATGCACCACAAGAAAAGGAGAAATGTCTGGATTTCAAA
GAACTTCTAGAGAGAGAAAGGAACAACCACCAAAGGAATAGTGTAAATATAAAATTAAAA
AATATATACGGAGAAGAACAATTGTATAATGTGAAGAAAGATAGCTTTATTGTAAAATTG
ATATACTATCTGCCGTTTGGCCCAATTGTTACAATGATAATTATAGGCTTTATTCTACTC
TGGTCAACGCCCTGGACTGGTTTTATTTTTATAGGGTGTAGCTCTATTATATTACCAGCT
TTGTTATACGCGATGCATTCAAACAAAAAACAATTCGAACGTGTATTTGGAAAAGGGAGA

```
AAAAAGCCAATGAAGGTAAAAAAAGGAGGCGGGAAAATATCTAGATTTTTTAAAGAAGTA
GACAAA---------------------------
>CH91 .
------------------------------TTAAGGAAAATTTCCCTGTGTTCTATTTTC
GTATTGGCATATCTCAGTTCTGCTAAGTGCGCGTACCGAGACGAATCTGACATATTGTAC
AGCGACGACTCCAGATCCGATTTAGCAAGCAACGGGGCACCCACCGACAGCTACGAATCT
TTAACAGCAAGTAGTGAGTCTCTAGCAGAAAGCAACGATGCACCCAGCAACAGCTATGAA
TCTTTTCCAGAAATTAGAGAAAATCTAACCGCAAGTGAGGAATCCCTAACATCATGTGAG
GAATCCCTAACAGGAAGTAAT---------------------------------------
------------------------------------------------------------
------------------------------------------------GAATCCCTAACA
GAAAGTAGAGAATCTCTAGAGGCCAGTAGAGAATCGCTAAGAGCAAGTAGAGAGTCTCTA
GCGGCCAGTAGAGAATCCCTGAACGACTTTTGTGGGAGCGAAGAATCAGTAGCATTCGAA
GGAGAGCCAAATGAAAAGACATTCATGGGAGACGTCTTAAGTGGTGGAGAATGTGAGAAT
AGTCTCTCAAGAGAAGATTTATTTCATATAGAAGTAGGATCCGAAGAATCGCTAGATGAT
GCCTCAAAATATAATTTCCAAAAGGATTTATCAACTAGCGATAATAGTTTATTCGAAGAT
GATCAGTCATTGAAAAGGGGACTCAAAAGGAACAGCTCAGTATCTAGTCTGGACAGCGAT
ATGGGAAGTTATAAAAATGGAAGATCTAGAGACAGATTGGATATATATTCGGACCTACCT
AGAAGGCTAATACATGGGGATAATGCACCACAAGAAAAGGAGAAATGTCTGGATTTCAAA
GAACTTCTAGAGAGAGAAAGGAACAACCACCAAAGGAATAGTGTAAATATAAAATTAAAA
AATATATACGGAGAAGAACAATTGTATAATGTGAAGAAAGATAGCTTTATTGTAAAATTG
ATATACTATCTGCCGTTTGGCCCAATTGTTACAATGATAATTATAGGCTTTATTCTACTC
TGGTCAACGACTTGGACTGGTTTTATTTTTATAGGGTGTAGCTCTATTATATTACCAGCT
TTGTTATACGCGATGCATTCAAACAAAAAACAATTCGAACGTGTATTTGGAAAAGGGAGA
AAAAAGCCAATGAAGGTAAAAAAAGGAGGCGGGAAAATATCTAGATTTTTTAAAGAAGTA
GACAAA---------------------------
>CH92 .
------------------------------TTAAGGAAAATTTCCCTGTGTTCTATTTTC
GTATTGGCATATCTCAGTTCTGCTAAGTGCGCGTACCGAGACGAATCTGACATATTGTAC
AGCGACGACTCCAGATCCGATTTAGCAAGCAACGGGGCACCCACCGACAGCTACGAATCT
TTAACAGCAAGTAGTGAGTCTCTAGCAGAAAGCAACGATGCACCCAGCAACAGCTATGAA
TCTTTTCCAGAAATTAGAGAAAATCTAACCGCAAGTGAGGAATCCCTAACA---------
------------GGAAGTAATGAATCCCTAACAGGAAGTAATGAATCCCTAACAGAAAGT
AAT------------------------------------------GAATCCCTAACAGGA
AGTAATGAATCCCTAACAGGAAGTAATGAATCCCTAACAGGAAGTAATGAATCCCTAACA
GAAAGTAGAGAATCTCTAGAGGCCAGTAGAGAATCGCTAAGAGCAAGTAGAGAGTCTCTA
GCGGCCAGTAGAGAATCCCTGAACGACTTTTGTGGGAGCGAAGAATCAGTAGCATTCGAA
GGAGAGCCAAATGAAAAGACATTCATGGGAGACGTCTTAAGTGGTGGAGAATGTGAGAAT
AGTCTCTCAAGAGAAGATTTATTTCATATAGAAGTAGGATCCGAAGAATCGCTAGATGAT
GCCTCAAAATATAATTTCCAAAAGGATTTATCAACTAGCGATAATAGTTTATTCGAAGAT
GATCAGTCATTGAAAAGGGGACTCAAAAGGAACAGCTCAGTATCTAGTCTGGACAGCGAT
ATGGGAAGTTATAAAAATGGAAGATCTAGAGACAGATTGGATATATATTCGGACCTACCT
AGAAGGCTAATACATGGGGATAATGCACCACAAGAAAAGGAGAAATGTCTGGATTTCAAA
GAACTTCTAGAGAGAGAAAGGAACAACCACCAAAGGAATAGTGTAAATATAAAATTAAAA
AATATATACGGAGAAGAACAATTGTATAATGTGAAGAAAGATAGCTTTATTGTAAAATTG
ATATACTATCTGCCGTTTGGCCCAATTGTTACAATGATAATGATAGGTTTTTTTCTACTC
```

```
TGCTGGACCCCCTCGATTGGTTTTATTTTTATAGGGTGTAGCTCTATTATATTACCAGCT
TTGTTATACGCGATGCATTCAAACAAAAAACAATTCGAACGTGTATTTGGAAAAGGGAGA
AAAAAGCCAATGAAGGTAAAAAAAGGAGGCGGGAAAATATCTAGATTTTTTAAAGAAGTA
GACAAA---------------------------
>AMZ18 .
------------------------------TTAAGGAAAATTTCCCTGTGTTCTATTTTC
GTATTGGCATATCTCAGTTCTGCTAAGTGCGCGTACCGAGACGAATCTGACATATTGTAC
AGCGACGACTCCAGATCCGATTTAGCAAGCAACGGGGCACCCACCGACAGCTACGAATCT
TTAACAGCAAGTAGTGAGTCTCTAGCAGAAAGCAACGATGCACCCAGCAACAGCTATGAA
TCTTTTCCAGAAATTAGAGAAAATCTAACCGCAAGTGAGGAATCCCTAACATCATGTGAG
GAATCCCTAACAGGAAGTAATGAATCCCTAACAGGAAGTAATGAATCCCTAACAGGAAGT
AAT---------------------------------------------------------
------------------------------------------------GAATCCCTAACA
GAAAGTAGAGAATCTCTAGAGGCCAGTAGAGAATCGCTAAGAGCAAGTAGAGAGTCTCTA
GCGGCCAGTAGAGAATCCCTGAACGACTTTTGTGGGAGCGAAGAATCAGTAGCATTCGAA
GGAGAGCCAAATGAAAAGACATTCATGGGAGACGTCTTAAGTGGTGGAGAATGTGAGAAT
AGTCTCTCAAGAGAAGATTTATTTCATATAGAAGTAGGATCCGAAGAATCGCTAGATGAT
GCCTCAAAATATAATTTCCAAAAGGATTTATCAACTAGCGATAATAGTTTATTCGAAGAT
GATCAGTCATTGAAAAGGGGACTCAAAAGGAACAGCTCAGTATCTAGTCTGGACAGCGAT
ATGGGAAGTTATAAAAATGGAAGATCTAGAGACAGATTGGATATATATTCGGACCTACCT
AGAAGGCTAATACATGGGGATAATGCACCACAAGAAAAGGAGAAATGTCTGGATTTCAAA
GAACTTCTAGAGAGAGAAAGGAACAACCACCAAAGGAATAGTGTAAATATAAAATTAAAA
AATATATACGGAGAAGAACAATTGTATAATGTGAAGAAAGATAGCTTTATTGTAAAATTG
ATATACTATCTGCCGTTTGGCCCAATTGTTACAATGATAATTATAGGCTTTATTCTACTC
TGGTCAACGCCCTGGACTGGTTTTATTTTTATAGGGTGTAGCTCTATTATATTACCAGCT
TTGTTATACGCGATGCATTCAAACAAAAAACAATTCGAACGTGTATTTGGAAAAGGGAGA
AAAAAGCCAATGAAGGTAAAAAAAGGAGGCGGGAAAATATCTAGATTTTTTAAAGAAGTA
GACAAA---------------------------
>AMZ20 .
------------------------------TTAAGGAAAATTTCCCTGTGTTCTATTTTC
GTATTGGCATATCTCAGTTCTGCTAAGTGCGCGTACCGAGACGAATCTGACATATTGTAC
AGCGACGACTCCAGATCCGATTTAGCAAGCAACGGGGCACCCACCGACAGCTACGAATCT
TTAACAGCAAGTAGTGAGTCTCTAGCAGAAAGCAACGATGCACCCAGCAACAGCTATGAA
TCTTTTCCAGAAATTAGAGAAAATCTAACCGCAAGTGAGGAATCCCTAACATCATGTGAG
GAATCCCTAACAGGAAGTAATGAATCCCTAACAGGAAGTAATGAATCCCTAACAGGAAGT
AAT------------------------------------------GAATCCCTAACAGGA
AGTAATGAATCCCTAACAGGAAGTAATGAATCCCTAACAGGAAGTAATGAATCCCTAACA
GAAAGTAGAGAATCTCTAGAGGCCAGTAGAGAATCGCTAAGAGCAAGTAGAGAGTCTCTA
GCGGCCAGTAGAGAATCCCTGAACGACTTTTGTGGGAGCGAAGAATCAGTAGCATTCGAA
GGAGAGCCAAATGAAAAGACATTCATGGGAGACGTCTTAAGTGGTGGAGAATGTGAGAAT
AGTCTCTCAAGAGAAGATTTATTTCATATAGAAGTAGGATCCGAAGAATCGCTAGATGAT
GCCTCAAAATATAATTTCCAAAAGGATTTATCAACTAGCGATAATAGTTCATTCGAAGAT
GATCAGTCATTGAAAAGGGGACTCAAAAGGAACAGCTCAGTATCTAGTCTGGACAGCGAT
ATGGGAAGTTATAAAAATAAAAGTTATAGACACAGATTGGATATATATTCGGACCTACCT
AGAAGGCCAATACATGGGGATAATGCACCACAAGAAAAGGAGAAATGTCTGGATTTCAAA
GAACTTCTAGAGAGAGAAAGGAACAACCACCAAAGGAATAGTGTAAATATAAAATTAAAA
AATATATACGGAGAAGAACAATTGTATAATGTGAAGAAAGATAGCTTTATTGTAAAATTG
ATATACTATCTGCCGTTTGGCCCAATTGTTACAATGATAATGATAGGTTTTTTTCTACTC
TGCTGGACCCCCTCGATTGGTTTTACTTTTATAGGGTGTAGCTCTATTATATTACCAGCT
TTGTTATACGCGATGCATTCAAACAAAAAACAATTCGAACGTGTATTTGGAAAAGGGAGA
```

```
AAAAAGCCAATGAAGGTAAAAAAAGGAGGCGGGAAAATATCTAGATTTTTTAAAGAAGTA
GACAAA---------------------------
>AMZ53 .
------------------------------TTAAGGAAAATTTCCCTGTGTTCTATTTTC
GTATTGGCATATCTCAGTTCTGCTAAGTGCGCGTACCGAGACGAATCTGACATATTGTAC
AGCGACGACTCCCGATCCGATTTATCAAGCAACGGGGCACCCCCCGACAGCTACGAATCT
TTAACAGCAAGTAGTGAGTCTCTAGCAGAAAGCAACGATGCACCCAGCAACAGCTATGAA
TCTTTTCCAGAAATTAGAGAAAATCTAACCGCAAGTGAGGAATCCCTAACATCATGTGAG
GAATCCCTAACAGGAAGTAATGAATCCCTAACAGGAAGTAATGAATCCCTAACAGGAAGT
AAT---------------------------------------------------------
---------------------------GAATCCCTAACAGGAAGTAATGAATCCCTAACA
GAAAGTAGAGAATCTCTAGAGGCCAGTAGAGAATCGCTAAGAGCAAGTAGAGAGTCTCTA
GCGGCCAGTAGAGAATCCCTGAACGACTTTTGTGGGAGCGAAGAATCAGTAGCATTCGAA
GGAGAGCCAAATGAAAAGACATTCATGGGAGACGTCTTAAGTGGTGGAGAATGTGAGAAT
AGTCTCTCAAGAGAAGATTTATTTCATATAGAAGTAGGATCCGAAGAATCGCTAGATGAT
GCCTCAAAATATAATTTCCAAAAGGATTTATCAACTAGCGATAATAGTTCATTCGAAGAT
GATCAGTCATTGAAAAGGGGACTCAAAAGGAACAGCTCAGTATCTAGTCTGGACAGCGAT
ATGGGAAGTTATAAAAATGGAAGATATAGAGACAGATTGGATATATATTCGGACCTACCT
AGAAGGCCAATACATGGGGATAATGCACCACAAGAAAAGGAGAAATGTCTGGATTTCAAA
GAACTTCTAGAGAGAGAAAGGAACAACCACCAAAGGAATAGTGTAAATATAAAATTAAAA
AATATATACGGAGAAGAACAATTGTATAATGTGAAGAAAGATAGCTTTATTGTAAAATTG
ATATACTATCTGCCGTTTGGCCCAATTGTTACAATGATAATGATAGGCTTTATTCTACTC
TGCTCAACGCCCTCGACTGGTTTTATTTTTATAGGGTGTAGCTCTATTATATTACCAGCT
TTGTTATACGCGATGCATTCAAACAAAAAACAATTCGAACGTGTATTTGGAAAAGGGAGA
AAAAAGCCAATGAAGGTAAAAAAAGGAGGCGGGAAAATATCTAGATTTTTTAAAGAAGTA
GACAAA---------------------------
>AMZ54 .
------------------------------TTAAGGAAAATTTCCCTGTGTTCTATTTTC
GTATTGGCATATCTCAGTTCTGCTAAGTGCGCGTACCGAGACGAATCTGACATATTGTAC
AGCGACGACTCCAGATCCGATTTAGCAAGCAACGGGGCACCCACCGACAGCTACGAATCT
TTAACAGCAAGTAGTGAGTCTCTAGCAGAAAGCAACGATGCACCCAGCAACAGCTATGAA
TCTTTTCCAGAAATTAGAGAAAATCTAACCGCAAGTGAGGAATCCCTAACATCATGTGAG
GAATCCCTAACAGGAAGTAATGAATCCCTAACAGGAAGTAATGAATCCCTAACAGGAAGT
AAT---------------------------------------------------------
---------------------------GAATCCCTAACAGGAAGTAATGAATCCCTAACA
GAAAGTAGAGAATCTCTAGAGGCCAGTAGAGAATCGCTAAGAGCAAGTAGAGAGTCTCTA
GCGGCCAGTAGAGAATCCCTGAACGACTTTTGTGGGAGCGAAGAATCAGTAGCATTCGAA
GGAGAGCCAAATGAAAAGACATTCATGGGAGACGTCTTAAGTGGTGGAGAATGTGAGAAT
AGTCTCTCAAGAGAAGATTTATTTCATATAGAAGTAGGATCCGAAGAATCGCTAGATGAT
GCCTCAAAATATAATTTCCAAAAGGATTTATCAACTAGCGATAATAGTTCATTCGAAGAT
GATCAGTCATTGAAAAGGGGACTCAAAAGGAACAGCTCAGTATCTAGTCTGGACAGCGAT
ATGGGAAGTTATAAAAATAAAAGTTATAGACACAGATTGGATATATATTCGGACCTACCT
AGAAGGCCAATACATGGGGATAATGCACCACAAGAAAAGGAGAAATGTCTGGATTTCAAA
GAACTTCTAGAGAGAGAAAGGAACAACCACCAAAGGAATAGTGTAAATATAAAATTAAAA
AATATATACGGAGAAGAACAATTGTATAATGTGAAGAAAGATAGCTTTATTGTAAAATTG
ATATACTATCTGCCGTTTGGCCCAATTGTTACAATGATAATGATAGGCTTTATTCTACTC
```

TGCTCAACGCCCTCGACTGGTTTTATTTTTATAGGGTGTAGCTCTATTATATTACCAGCT
TTGTTATACGCGATGCATTCAAACAAAAAACAATTCGAACGTGTATTTGGAAAAGGGAGA
AAAAAGCCAATGAAGGTAAAAAAAGGAGGCGGGAAAATATCTAGATTTTTTAAAGAAGTA
GACAAA---------------------------
>AMZ59 .
------------------------------TTAAGGAAAATTTCCCTGTGTTCTATTTTC
GTATTGGCATATCTCAGTTCTGCTAAGTGCGCGTACCGAGACGAATCTGACATATTGTAC
AGCGACGACTCCAGATCCGATTTAGCAAGCAACGGGGCACCCACCGACAGCTACGAATCT
TTAACAGCAAGTAGTGAGTCTCTAGCAGAAAGCAACGATGCACCCAGCAACAGCTATGAA
TCTTTTCCAGAAATTAGAGAAAATCTAACCGCAAGTGAGGAATCCCTAACATCATGTGAG
GAATCCCTAACAGGAAGTAATGAATCCCTAACAGGAAGTAATGAATCCCTAACAGGAAGT
AAT--------------------------------------------GAATCCCTAACAGGA
AGTAATGAATCCCTAACAGGAAGTAATGAATCCCTAACAGGAAGTAATGAATCCCTAACA
GAAAGTAGAGAATCTCTAGAGGCCAGTAGAGAATCGCTAAGAGCAAGTAGAGAGTCTCTA
GCGGCCAGTAGAGAATCCCTGAACGACTTTTGTGGGAGCGAAGAATCAGTAGCATTCGAA
GGAGAGCCAAATGAAAAGACATTCATGGGAGACGTCTTAAGTGGTGGAGAATGTGAGAAT
AGTCTCTCAAGAGAAGATTTATTTCATATAGAAGTAGGATCCGAAGAATCGCTAGATGAT
GCCTCAAAATATAATTTCCAAAAGGATTTATCAACTAGCGATAATAGTTCATTCGAAGAT
GATCAGTCATTGAAAAGGGGACTCAAAAGGAACAGCTCAGTATCTAGTCTGGACAGCGAT
ATGGGAAGTTATAAAAATAAAAGTTATAGACACAGATTGGATATATATTCGGACCTACCT
AGAAGGCCAATACATGGGGATAATGCACCACAAGAAAAGGAGAAATGTCTGGATTTCAAA
GAACTTCTAGAGAGAGAAAGGAACAACCACCAAAGGAATAGTGTAAATATAAAATTAAAA
AATATATACGGAGAAGAACAATTGTATAATGTGAAGAAAGATAGCTTTATTGTAAAATTG
ATATACTATCTGCCGTTTGGCCCAATTGTTACAATGATAATGATAGGTTTTTTTCTACTC
TGCTGGACCCCCTCGATTGGTTTTACTTTTATAGGGTGTAGCTCTATTATATTACCAGCT
TTGTTATACGCGATGCATTCAAACAAAAAACAATTCGAACGTGTATTTGGAAAAGGGAGA
AAAAAGCCAATGAAGGTAAAAAAAGGAGGCGGGAAAATATCTAGATTTTTTAAAGAAGTA
GACAAA---------------------------
>AMZ60 .
------------------------------TTAAGGAAAATTTCCCTGTGTTCTATTTTC
GTATTGGCATATCTCAGTTCTGCTAAGTGCGCGTACCGAGACGAATCTGACATATTGTAC
AGCGACGACTCCAGATCCGATTTAGCAAGCAACGGGGCACCCACCGACAGCTACGAATCT
TTAACAGCAAGTAGTGAGTCTCTAGCAGAAAGCAACGATGCACCCAGCAACAGCTATGAA
TCTTTTCCAGAAATTAGAGAAAATCTAACCGCAAGTGAGGAATCCCTAACATCATGTGAG
GAATCCCTAACAGGAAGTAATGAATCCCTAACAGGAAGTAATGAATCCCTAACAGGAAGT
AAT---------------------GAATCCCTAACAGGAAGTAATGAATCCCTAACAGGA
AGTAATGAATCCCTAACAGGAAGTAATGAATCCCTAACAGGAAGTAATGAATCCCTAACA
GAAAGTAGAGAATCTCTAGAGGCCAGTAGAGAATCGCTAAGAGCAAGTAGAGAGTCTCTA
GCGGCCAGTAGAGAATCCCTGAACGACTTTTGTGGGAGCGAAGAATCAGTAGCATTCGAA
GGAGAGCCAAATGAAAAGACATTCATGGGAGACGTCTTAAGTGGTGGAGAATGTGAGAAT
AGTCTCTCAAGAGAAGATTTATTTCATATAGAAGTAGGATCCGAAGAATCGCTAGATGAT
GCCTCAAAATATAATTTCCAAAAGGATTTATCAACTAGCGATAATAGTTTATTCGAAGAT
GATCAGTCATTGAAAAGGGGACTCAAAAGGAACAGCTCAGTATCTAGTCTGGACAGCGAT
ATGGGAAGTTATAAAAATGGAAGATCTAGAGACAGATTGGATATATATTCGGACCTACCT
AGAAGGCTAATACATGGGGATAATGCACCACAAGAAAAGGAGAAATGTCTGGATTTCAAA
GAACTTCTAGAGAGAGAAAGGAACAACCACCAAAGGAATAGTGTAAATATAAAATTAAAA
AATATATACGGAGAAGAACAATTGTATAATGTGAAGAAAGATAGCTTTATTGTAAAATTG
ATATACTATCTGCCGTTTGGCCCAATTGTTACAATGATAATGATAGGTTTTTTTCTACTC
TGCTGGACCCCCTCGATTGGTTTTACTTTTATAGGGTGTAGCTCTATTATATTACCAGCT
TTGTTATACGCGATGCATTCAAACAAAAAACAATTCGAACGTGTATTTGGAAAAGGGAGA

AAAAAGCCAATGAAGGTAAAAAAAGGAGGCGGGAAAATATCTAGATTTTTTAAAGAAGTA
GACAAA---------------------------
>AMZ71 .
------------------------------TTAAGGAAAATTTCCCTGTGTTCTATTTTC
GTATTGGCATATCTCAGTTCTGCTAAGTGCGCGTACCGAGACGAATCTGACATATTGTAC
AGCGACGACTCCAGATCCGATTTAGCAAGCAACGGGGCACCCACCGACAGCTACGAATCT
TTAACAGCAAGTAGTGAGTCTCTAGCAGAAAGCAACGATGCACCCAGCAACAGCTATGAA
TCTTTTCCAGAAATTAGAGAAAATCTAACCGCAAGTGAGGAATCCCTAACATCATGTGAG
GAATCCCTAACAGGAAGTAATGAATCCCTAACAGGAAGTAATGAATCCCTAACAGGAAGT
AAT---------------------GAATCCCTAACAGGAAGTAATGAATCCCTAACAGGA
AGTAATGAATCCCTAACAGGAAGTAATGAATCCCTAACAGGAAGTAATGAATCCCTAACA
GAAAGTAGAGAATCTCTAGAGGCCAGTAGAGAATCGCTAAGAGCAAGTAGAGAGTCTCTA
GCGGCCAGTAGAGAATCCCTGAACGACTTTTGTGGGAGCGAAGAATCAGTAGCATTCGAA
GGAGAGCCAAATGAAAAGACATTCATGGGAGACGTCTTAAGTGGTGGAGAATGTGAGAAT
AGTCTCTCAAGAGAAGATTTATTTCATATAGAAGTAGGATCCGAAGAATCGCTAGATGAT
GCCTCAAAATATAATTTCCAAAAGGATTTATCAACTAGCGATAATAGTTTATTCGAAGAT
GATCAGTCATTGAAAAGGGGACTCAAAAGGAACAGCTCAGTATCTAGTCTGGACAGCGAT
ATGGGAAGTTATAAAAATGGAAGATCTAGAGACAGATTGGATATATATTCGGACCTACCT
AGAAGGCTAATACATGGGGATAATGCACCACAAGAAAAGGAGAAATGTCTGGATTTCAAA
GAACTTCTAGAGAGAGAAAGGAACAACCACCAAAGGAATAGTGTAAATATAAAATTAAAA
AATATATACGGAGAAGAACAATTGTATAATGTGAAGAAAGATAGCTTTATTGTAAAATTG
ATATACTATCTGCCGTTTGGCCCAATTGTTACAATGATAATGATAGGTTTTTTTCTACTC
TGCTGGACCCCCTCGATTGGTTTTACTTTTATAGGGTGTAGCTCTATTATATTACCAGCT
TTGTTATACGCGATGCATTCAAACAAAAAACAATTCGAACGTGTATTTGGAAAAGGGAGA
AAAAAGCCAATGAAGGTAAAAAAAGGAGGCGGGAAAATATCTAGATTTTTTAAAGAAGTA
GACAAA---------------------------
>AMZ98 .
------------------------------TTAAGGAAAATTTCCCTGTGTTCTATTTTC
GTATTGGCATATCTCAGTTCTGCTAAGTGCGCGTACCGAGACGAATCTGACATATTGTAC
AGCGACGACTCCAGATCCGATTTAGCAAGCAACGGGGCACCCACCGACAGCTACGAATCT
TTAACAGCAAGTAGTGAGTCTCTAGCAGAAAGCAACGATGCACCCAGCAACAGCTATGAA
TCTTTTCCAGAAATTAGAGAAAATCTAACCGCAAGTGAGGAATCCCTAACATCATGTGAG
GAATCCCTAACAGGAAGTAATGAATCCCTAACAGGAAGTAATGAATCCCTAACAGGAAGT
AAT---------------------------------------------------------
---------------------------GAATCCCTAACAGGAAGTAATGAATCCCTAACA
GAAAGTAGAGAATCTCTAGAGGCCAGTAGAGAATCGCTAAGAGCAAGTAGAGAGTCTCTA
GCGGCCAGTAGAGAATCCCTGAACGACTTTTGTGGGAGCGAAGAATCAGTAGCATTCGAA
GGAGAGCCAAATGAAAAGACATTCATGGGAGACGTCTTAAGTGGTGGAGAATGTGAGAAT
AGTCTCTCAAGAGAAGATTTATTTCATATAGAAGTAGGATCCGAAGAATCGCTAGATGAT
GCCTCAAAATATAATTTCCAAAAGGATTTATCAACTAGCGATAATAGTTCATTCGAAGAT
GATCAGTCATTGAAAAGGGGACTCAAAAGGAACAGCTCAGTATCTAGTCTGGACAGCGAT
ATGGGAAGTTATAAAAATAAAAGTTATAGACACAGATTGGATATATATTCGGACCTACCT
AGAAGGCCAATACATGGGGATAATGCACCACAAGAAAAGGAGAAATGTCTGGATTTCAAA
GAACTTCTAGAGAGAGAAAGGAACAACCACCAAAGGAATAGTGTAAATATAAAATTAAAA
AATATATACGGAGAAGAACAATTGTATAATGTGAAGAAAGATAGCTTTATTGTAAAATTG
ATATACTATCTGCCGTTTGGCCCAATTGTTACAATGATAATGATAGGCTTTATTCTACTC

```
TGCTCAACGCCCTCGACTGGTTTTATTTTTATAGGGTGTAGCTCTATTATATTACCAGCT
TTGTTATACGCGATGCATTCAAACAAAAAACAATTCGAACGTGTATTTGGAAAAGGGAGA
AAAAAGCCAATGAAGGTAAAAAAAGGAGGCGGGAAAATATCTAGATTTTTTAAAGAAGTA
GACAAA---------------------------
>AMZ110 .
------------------------------TTAAGGAAAATTTCCCTGTGTTCTATTTTC
GTATTGGCATATCTCAGTTCTGCTAAGTGCGCGTACCGAGACGAATCTGACATATTGTAC
AGCGACGACTCCAGATCCGATTTAGCAAGCAACGGGGCACCCACCGACAGCTACGAATCT
TTAACAGCAAGTAGTGAGTCTCTAGCAGAAAGCAACGATGCACCCAGCAACAGCTATGAA
TCTTTTCCAGAAATTAGAGAAAATCTAACCGCAAGTGAGGAATCCCTAACATCATGTGAG
GAATCCCTAACAGGAAGTAATGAATCCCTAACAGGAAGTAATGAATCCCTAACAGGAAGT
AAT---------------------------------------------------------
---------------------------GAATCCCTAACAGGAAGTAATGAATCCCTAACA
GAAAGTAGAGAATCTCTAGAGGCCAGTAGAGAATCGCTAAGAGCAAGTAGAGAGTCTCTA
GCGGCCAGTAGAGAATCCCTGAACGACTTTTGTGGGAGCGAAGAATCAGTAGCATTCGAA
GGAGAGCCAAATGAAAAGACATTCATGGGAGACGTCTTAAGTGGTGGAGAATGTGAGAAT
AGTCTCTCAAGAGAAGATTTATTTCATATAGAAGTAGGATCCGAAGAATCGCTAGATGAT
GCCTCAAAATATAATTTCCAAAAGGATTTATCAACTAGCGATAATAGTTCATTCGAAGAT
GATCAGTCATTGAAAAGGGGACTCAAAAGGAACAGCTCAGTATCTAGTCTGGACAGCGAT
ATGGGAAGTTATAAAAATGGAAGATCTAGAGACAGATTGGATATATATTCGGACCTACCT
AGAAGGCCAATACATGGGGATAATGCACCACAAGAAAAGGAGAAATGTCTGGATTTCAAA
GAACTTCTAGAGAGAGAAAGGAACAACCACCAAAGGAATAGTGTAAATATAAAATTAAAA
AATATATACGGAGAAGAACAATTGTATAATGTGAAGAAAGATAGCTTTATTGTAAAATTG
ATATACTATCTGCCGTTTGGCCCAATTGTTACAATGATAATGATAGGCTTTATTCTACTC
TGCTCAACGCCCTCGACTGGTTTTATTTTTATAGGGTGTAGCTCTATTATATTACCAGCT
TTGTTATACGCGATGCATTCAAACAAAAAACAATTCGAACGTGTATTTGGAAAAGGGAGA
AAAAAGCCAATGAAGGTAAAAAAAGGAGGCGGGAAAATATCTAGATTTTTTAAAGAAGTA
GACAAA---------------------------
>AMZ114 .
------------------------------TTAAGGAAAATTTCCCTGTGTTCTATTTTC
GTATTGGCATATCTCAGTTCTGCTAAGTGCGCGTACCGAGACGAATCTGACATATTGTAC
AGCGACGACTCCAGATCCGATTTAGCAAGCAACGGGGCACCCACCGACAGCTACGAATCT
TTAACAGCAAGTAGTGAGTCTCTAGCAGAAAGCAACGATGCACCCAGCAACAGCTATGAA
TCTTTTCCAGAAATTAGAGAAAATCTAACCGCAAGTGAGGAATCCCTAACATCATGTGAG
GAATCCCTAACAGGAAGTAATGAATCCCTAACAGGAAGTAATGAATCCCTAACAGAAAGT
AAT---------------------------------------------------------
------GAATCCCTAACAGGAAGTAATGAATCCCTAACAGGAAGTAATGAATCCCTAACA
GAAAGTAGAGAATCTCTAGAGGCCAGTAGAGAATCGCTAAGAGCAAGTAGAGAGTCTCTA
GCGGCCAGTAGAGAATCCCTGAACGACTTTTGTGGGAGCGAAGAATCAGTAGCATTCGAA
GGAGAGCCAAATGAAAAGACATTCATGGGAGACGTCTTAAGTGGTGGAGAATGTGAGAAT
AGTCTCTCAAGAGAAGATTTATTTCATATAGAAGTAGGATCCGAAGAATCGCTAGATGAT
GCCTCAAAATATAATTTCCAAAAGGATTTATCAACTAGCGATAATAGTTTATTCGAAGAT
GATCAGTCATTGAAAAGGGGACTCAAAAGGAACAGCTCAGTATCTAGTCTGGACAGCGAT
ATGGGAAGTTATAAAAATGGAAGATCTAGAGACAGATTGGATATATATTCGGACCTACCT
AGAAGGCTAATACATGGGGATAATGCACCACAAGAAAAGGAGAAATGTCTGGATTTCAAA
GAACTTCTAGAGAGAGAAAGGAACAACCACCAAAGGAATAGTGTAAATATAAAATTAAAA
AATATATACGGAGAAGAACAATTGTATAATGTGAAGAAAGATAGCTTTATTGTAAAATTG
ATATACTATCTGCCGTTTGGCCCAATTGTTACAATGATAATGATAGGTTTTTTTCTACTC
TGCTGGACCCCCTCGATTGGTTTTATTTTTATAGGGTGTAGCTCTATTATATTACCAGCT
TTGTTATACGCGATGCATTCAAACAAAAAACAATTCGAACGTGTATTTGGAAAAGGGAGA
```

AAAAAGCCAATGAAGGTAAAAAAAGGAGGCGGGAAAATATCTAGATTTTTTAAAGAAGTA
GACAAA---------------------------
>AMZ115 .
------------------------------TTAAGGAAAATTTCCCTGTGTTCTATTTTC
GTATTGGCATATCTCAGTTCTGCTAAGTGCGCGTACCGAGACGAATCTGACATATTGTAC
AGCGACGACTCCAGATCCGATTTAGCAAGCAACGGGGCACCCACCGACAGCTACGAATCT
TTAACAGCAAGTAGTGAGTCTCTAGCAGAAAGCAACGATGCACCCAGCAACAGCTATGAA
TCTTTTCCAGAAATTAGAGAAAATCTAACCGCAAGTGAGGAATCCCTAACATCATGTGAG
GAATCCCTAACAGGAAGTAATGAATCCCTAACAGGAAGTAATGAATCCCTAACAGGAAGT
AAT------------------------------------------GAATCCCTAACAGGA
AGTAATGAATCCCTAACAGGAAGTAATGAATCCCTAACAGGAAGTAATGAATCCCTAACA
GAAAGTAGAGAATCTCTAGAGGCCAGTAGAGAATCGCTAAGAGCAAGTAGAGAGTCTCTA
GCGGCCAGTAGAGAATCCCTGAACGACTTTTGTGGGAGCGAAGAATCAGTAGCATTCGAA
GGAGAGCCAAATGAAAAGACATTCATGGGAGACGTCTTAAGTGGTGGAGAATGTGAGAAT
AGTCTCTCAAGAGAAGATTTATTTCATATAGAAGTAGGATCCGAAGAATCGCTAGATGAT
GCCTCAAAATATAATTTCCAAAAGGATTTATCAACTAGCGATAATAGTTCATTCGAAGAT
GATCAGTCATTGAAAAGGGGACTCAAAAGGAACAGCTCAGTATCTAGTCTGGACAGCGAT
ATGGGAAGTTATAAAAATAAAAGTTATAGACACAGATTGGATATATATTCGGACCTACCT
AGAAGGCCAATACATGGGGATAATGCACCACAAGAAAAGGAGAAATGTCTGGATTTCAAA
GAACTTCTAGAGAGAGAAAGGAACAACCACCAAAGGAATAGTGTAAATATAAAATTAAAA
AATATATACGGAGAAGAACAATTGTATAATGTGAAGAAAGATAGCTTTATTGTAAAATTG
ATATACTATCTGCCGTTTGGCCCAATTGTTACAATGATAATGATAGGTTTTTTTCTACTC
TGCTGGACCCCCTCGATTGGTTTTACTTTTATAGGGTGTAGCTCTATTATATTACCAGCT
TTGTTATACGCGATGCATTCAAACAAAAAACAATTCGAACGTGTATTTGGAAAAGGGAGA
AAAAAGCCAATGAAGGTAAAAAAAGGAGGCGGGAAAATATCTAGATTTTTTAAAGAAGTA
GACAAA---------------------------
>AMZ122 .
------------------------------TTAAGGAAAATTTCCCTGTGTTCTATTTTC
GTATTGGCATATCTCAGTTCTGCTAAGTGCGCGTACCGAGACGAATCTGACATATTGTAC
AGCGACGACTCCAGATCCGATTTAGCAAGCAACGGGGCACCCACCGACAGCTACGAATCT
TTAACAGCAAGTAGTGAGTCTCTAGCAGAAAGCAACGATGCACCCAGCAACAGCTATGAA
TCTTTTCCAGAAATTAGAGAAAATCTAACCGCAAGTGAGGAATCCCTAACATCATGTGAG
GAATCCCTAACAGGAAGTAATGAATCCCTAACAGGAAGTAATGAATCCCTAACAGGAAGT
AAT------------------------------------------GAATCCCTAACAGGA
AGTAATGAATCCCTAACAGGAAGTAATGAATCCCTAACAGGAAGTAATGAATCCCTAACA
GAAAGTAGAGAATCTCTAGAGGCCAGTAGAGAATCGCTAAGAGCAAGTAGAGAGTCTCTA
GCGGCCAGTAGAGAATCCCTGAACGACTTTTGTGGGAGCGAAGAATCAGTAGCATTCGAA
GGAGAGCCAAATGAAAAGACATTCATGGGAGACGTCTTAAGTGGTGGAGAATGTGAGAAT
AGTCTCTCAAGAGAAGATTTATTTCATATAGAAGTAGGATCCGAAGAATCGCTAGATGAT
GCCTCAAAATATAATTTCCAAAAGGATTTATCAACTAGCGATAATAGTTCATTCGAAGAT
GATCAGTCATTGAAAAGGGGACTCAAAAGGAACAGCTCAGTATCTAGTCTGGACAGCGAT
ATGGGAAGTTATAAAAATAAAAGTTATAGACACAGATTGGATATATATTCGGACCTACCT
AGAAGGCCAATACATGGGGATAATGCACCACAAGAAAAGGAGAAATGTCTGGATTTCAAA
GAACTTCTAGAGAGAGAAAGGAACAACCACCAAAGGAATAGTGTAAATATAAAATTAAAA
AATATATACGGAGAAGAACAATTGTATAATGTGAAGAAAGATAGCTTTATTGTAAAATTG
ATATACTATCTGCCGTTTGGCCCAATTGTTACAATGATAATTATAGGCTTTATTCTACTC

TGGTCAACGCCCTGGACTGGTTTTATTTTTATAGGGTGTAGCTCTATTATATTACCAGCT
TTGTTATACGCGATGCATTCAAACAAAAAACAATTCGAACGTGTATTTGGAAAAGGGAGA
AAAAAGCCAATGAAGGTAAAAAAAGGAGGCGGGAAAATATCTAGATTTTTTAAAGAAGTA
GACAAA---------------------------
>AMZ123 .
------------------------------TTAAGGAAAATTTCCCTGTGTTCTATTTTC
GTATTGGCATATCTCAGTTCTGCTAAGTGCGCGTACCGAGACGAATCTGACATATTGTAC
AGCGACGACTCCAGATCCGATTTAGCAAGCAACGGGGCACCCACCGACAGCTACGAATCT
TTAACAGCAAGTAGTGAGTCTCTAGCAGAAAGCAACGATGCACCCAGCAACAGCTATGAA
TCTTTTCCAGAAATTAGAGAAAATCTAACCGCAAGTGAGGAATCCCTAACATCATGTGAG
GAATCCCTAACAGGAAGTAATGAATCCCTAACAGGAAGTAATGAATCCCTAACAGGAAGT
AAT---------------------------------------------------------
---------------------------GAATCCCTAACAGGAAGTAATGAATCCCTAACA
GAAAGTAGAGAATCTCTAGAGGCCAGTAGAGAATCGCTAAGAGCAAGTAGAGAGTCTCTA
GCGGCCAGTAGAGAATCCCTGAACGACTTTTGTGGGAGCGAAGAATCAGTAGCATTCGAA
GGAGAGCCAAATGAAAAGACATTCATGGGAGACGTCTTAAGTGGTGGAGAATGTGAGAAT
AGTCTCTCAAGAGAAGATTTATTTCATATAGAAGTAGGATCCGAAGAATCGCTAGATGAT
GCCTCAAAATATAATTTCCAAAAGGATTTATCAACTAGCGATAATAGTTCATTCGAAGAT
GATCAGTCATTGAAAAGGGGACTCAAAAGGAACAGCTCAGTATCTAGTCTGGACAGCGAT
ATGGGAAGTTATAAAAATGGAAGATCTAGAGACAGATTGGATATATATTCGGACCTACCT
AGAAGGCCAATACATGGGGATAATGCACCACAAGAAAAGGAGAAATGTCTGGATTTCAAA
GAACTTCTAGAGAGAGAAAGGAACAACCACCAAAGGAATAGTGTAAATATAAAATTAAAA
AATATATACGGAGAAGAACAATTGTATAATGTGAAGAAAGATAGCTTTATTGTAAAATTG
ATATACTATCTGCCGTTTGGCCCAATTGTTACAATGATAATGATAGGCTTTATTCTACTC
TGCTCAACGCCCTCGACTGGTTTTATTTTTATAGGGTGTAGCTCTATTATATTACCAGCT
TTGTTATACGCGATGCATTCAAACAAAAAACAATTCGAACGTGTATTTGGAAAAGGGAGA
AAAAAGCCAATGAAGGTAAAAAAAGGAGGCGGGAAAATATCTAGATTTTTTAAAGAAGTA
GACAAA---------------------------
>AMZ124 .
------------------------------TTAAGGAAAATTTCCCTGTGTTCTATTTTC
GTATTGGCATATCTCAGTTCTGCTAAGTGCGCGTACCGAGACGAATCTGACATATTGTAC
AGCGACGACTCCAGATCCGATTTAGCAAGCAACGGGGCACCCACCGACAGCTACGAATCT
TTAACAGCAAGTAGTGAGTCTCTAGCAGAAAGCAACGATGCACCCAGCAACAGCTATGAA
TCTTTTCCAGAAATTAGAGAAAATCTAACCGCAAGTGAGGAATCCCTAACATCATGTGAG
GAATCCCTAACAGGAAGTAATGAATCCCTAACAGGAAGTAATGAATCCCTAACAGGAAGT
AAT------------------------------------------GAATCCCTAACAGGA
AGTAATGAATCCCTAACAGGAAGTAATGAATCCCTAACAGGAAGTAATGAATCCCTAACA
GAAAGTAGAGAATCTCTAGAGGCCAGTAGAGAATCGCTAAGAGCAAGTAGAGAGTCTCTA
GCGGCCAGTAGAGAATCCCTGAACGACTTTTGTGGGAGCGAAGAATCAGTAGCATTCGAA
GGAGAGCCAAATGAAAAGACATTCATGGGAGACGTCTTAAGTGGTGGAGAATGTGAGAAT
AGTCTCTCAAGAGAAGATTTATTTCATATAGAAGTAGGATCCGAAGAATCGCTAGATGAT
GCCTCAAAATATAATTTCCAAAAGGATTTATCAACTAGCGATAATAGTTCATTCGAAGAT
GATCAGTCATTGAAAAGGGGACTCAAAAGGAACAGCTCAGTATCTAGTCTGGACAGCGAT
ATGGGAAGTTATAAAAATAAAAGTTATAGACACAGATTGGATATATATTCGGACCTACCT
AGAAGGCCAATACATGGGGATAATGCACCACAAGAAAAGGAGAAATGTCTGGATTTCAAA
GAACTTCTAGAGAGAGAAAGGAACAACCACCAAAGGAATAGTGTAAATATAAAATTAAAA
AATATATACGGAGAAGAACAATTGTATAATGTGAAGAAAGATAGCTTTATTGTAAAATTG
ATATACTATCTGCCGTTTGGCCCAATTGTTACAATGATAATGATAGGTTTTTTTCTACTC
TGCTGGACCCCCTCGATTGGTTTTACTTTTATAGGGTGTAGCTCTATTATATTACCAGCT
TTGTTATACGCGATGCATTCAAACAAAAAACAATTCGAACGTGTATTTGGAAAAGGGAGA

AAAAAGCCAATGAAGGTAAAAAAAGGAGGCGGGAAAATATCTAGATTTTTTAAAGAAGTA
GACAAA---------------------------
>AMZ133 .
------------------------------TTAAGGAAAATTTCCCTGTGTTCTATTTTC
GTATTGGCATATCTCAGTTCTGCTAAGTGCGCGTACCGAGACGAATCTGACATATTGTAC
AGCGACGACTCCAGATCCGATTTAGCAAGCAACGGGGCACCCACCGACAGCTACGAATCT
TTAACAGCAAGTAGTGAGTCTCTAGCAGAAAGCAACGATGCACCCAGCAACAGCTATGAA
TCTTTTCCAGAAATTAGAGAAAATCTAACCGCAAGTGAGGAATCCCTAACATCATGTGAG
GAATCCCTAACAGGAAGTAATGAATCCCTAACAGGAAGTAATGAATCCCTAACAGAAAGT
AAT---------------------------------------------------------
------GAATCCCTAACAGGAAGTAATGAATCCCTAACAGGAAGTAATGAATCCCTAACA
GAAAGTAGAGAATCTCTAGAGGCCAGTAGAGAATCGCTAAGAGCAAGTAGAGAGTCTCTA
GCGGCCAGTAGAGAATCCCTGAACGACTTTTGTGGGAGCGAAGAATCAGTAGCATTCGAA
GGAGAGCCAAATGAAAAGACATTCATGGGAGACGTCTTAAGTGGTGGAGAATGTGAGAAT
AGTCTCTCAAGAGAAGATTTATTTCATATAGAAGTAGGATCCGAAGAATCGCTAGATGAT
GCCTCAAAATATAATTTCCAAAAGGATTTATCAACTAGCGATAATAGTTTATTCGAAGAT
GATCAGTCATTGAAAAGGGGACTCAAAAGGAACAGCTCAGTATCTAGTCTGGACAGCGAT
ATGGGAAGTTATAAAAATGGAAGATCTAGAGACAGATTGGATATATATTCGGACCTACCT
AGAAGGCTAATACATGGGGATAATGCACCACAAGAAAAGGAGAAATGTCTGGATTTCAAA
GAACTTCTAGAGAGAGAAAGGAACAACCACCAAAGGAATAGTGTAAATATAAAATTAAAA
AATATATACGGAGAAGAACAATTGTATAATGTGAAGAAAGATAGCTTTATTGTAAAATTG
ATATACTATCTGCCGTTTGGCCCAATTGTTACAATGATAATGATAGGTTTTTTTCTACTC
TGCTGGACCCCCTCGATTGGTTTTATTTTTATAGGGTGTAGCTCTATTATATTACCAGCT
TTGTTATACGCGATGCATTCAAACAAAAAACAATTCGAACGTGTATTTGGAAAAGGGAGA
AAAAAGCCAATGAAGGTAAAAAAAGGAGGCGGGAAAATATCTAGATTTTTTAAAGAAGTA
GACAAA---------------------------
>AMZ134 .
------------------------------TTAAGGAAAATTTCCCTGTGTTCTATTTTC
GTATTGGCATATCTCAGTTCTGCTAAGTGCGCGTACCGAGACGAATCTGACATATTGTAC
AGCGACGACTCCAGATCCGATTTAGCAAGCAACGGGGCACCCACCGACAGCTACGAATCT
TTAACAGCAAGTAGTGAGTCTCTAGCAGAAAGCAACGATGCACCCAGCAACAGCTATGAA
TCTTTTCCAGAAATTAGAGAAAATCTAACCGCAAGTGAGGAATCCCTAACATCATGTGAG
GAATCCCTAACAGGAAGTAATGAATCCCTAACAGGAAGTAATGAATCCCTAACAGAAAGT
AAT---------------------------------------------------------
------GAATCCCTAACAGGAAGTAATGAATCCCTAACAGGAAGTAATGAATCCCTAACA
GAAAGTAGAGAATCTCTAGAGGCCAGTAGAGAATCGCTAAGAGCAAGTAGAGAGTCTCTA
GCGGCCAGTAGAGAATCCCTGAACGACTTTTGTGGGAGCGAAGAATCAGTAGCATTCGAA
GGAGAGCCAAATGAAAAGACATTCATGGGAGACGTCTTAAGTGGTGGAGAATGTGAGAAT
AGTCTCTCAAGAGAAGATTTATTTCATATAGAAGTAGGATCCGAAGAATCGCTAGATGAT
GCCTCAAAATATAATTTCCAAAAGGATTTATCAACTAGCGATAATAGTTTATTCGAAGAT
GATCAGTCATTGAAAAGGGGACTCAAAAGGAACAGCTCAGTATCTAGTCTGGACAGCGAT
ATGGGAAGTTATAAAAATGGAAGATCTAGAGACAGATTGGATATATATTCGGACCTACCT
AGAAGGCTAATACATGGGGATAATGCACCACAAGAAAAGGAGAAATGTCTGGATTTCAAA
GAACTTCTAGAGAGAGAAAGGAACAACCACCAAAGGAATAGTGTAAATATAAAATTAAAA
AATATATACGGAGAAGAACAATTGTATAATGTGAAGAAAGATAGCTTTATTGTAAAATTG
ATATACTATCTGCCGTTTGGCCCAATTGTTACAATGATAATGATAGGTTTTTTTCTACTC

TGCTGGACCCCCTCGATTGGTTTTATTTTTATAGGGTGTAGCTCTATTATATTACCAGCT
TTGTTATACGCGATGCATTCAAACAAAAAACAATTCGAACGTGTATTTGGAAAAGGGAGA
AAAAAGCCAATGAAGGTAAAAAAAGGAGGCGGGAAAATATCTAGATTTTTTAAAGAAGTA
GACAAA---------------------------
>AMZ138 .
------------------------------TTAAGGAAAATTTCCCTGTGTTCTATTTTC
GTATTGGCATATCTCAGTTCTGCTAAGTGCGCGTACCGAGACGAATCTGACATATTGTAC
AGCGACGACTCCAGATCCGATTTAGCAAGCAACGGGGCACCCACCGACAGCTACGAATCT
TTAACAGCAAGTAGTGAGTCTCTAGCAGAAAGCAACGATGCACCCAGCAACAGCTATGAA
TCTTTTCCAGAAATTAGAGAAAATCTAACCGCAAGTGAGGAATCCCTAACATCATGTGAG
GAATCCCTAACAGGAAGTAATGAATCCCTAACAGGAAGTAATGAATCCCTAACAGGAAGT
AAT---------------------------------------------------------
---------------------------GAATCCCTAACAGGAAGTAATGAATCCCTAACA
GAAAGTAGAGAATCTCTAGAGGCCAGTAGAGAATCGCTAAGAGCAAGTAGAGAGTCTCTA
GCGGCCAGTAGAGAATCCCTGAACGACTTTTGTGGGAGCGAAGAATCAGTAGCATTCGAA
GGAGAGCCAAATGAAAAGACATTCATGGGAGACGTCTTAAGTGGTGGAGAATGTGAGAAT
AGTCTCTCAAGAGAAGATTTATTTCATATAGAAGTAGGATCCGAAGAATCGCTAGATGAT
GCCTCAAAATATAATTTCCAAAAGGATTTATCAACTAGCGATAATAGTTCATTCGAAGAT
GATCAGTCATTGAAAAGGGGACTCAAAAGGAACAGCTCAGTATCTAGTCTGGACAGCGAT
ATGGGAAGTTATAAAAATAAAAGTTATAGACACAGATTGGATATATATTCGGACCTACCT
AGAAGGCCAATACATGGGGATAATGCACCACAAGAAAAGGAGAAATGTCTGGATTTCAAA
GAACTTCTAGAGAGAGAAAGGAACAACCACCAAAGGAATAGTGTAAATATAAAATTAAAA
AATATATACGGAGAAGAACAATTGTATAATGTGAAGAAAGATAGCTTTATTGTAAAATTG
ATATACTATCTGCCGTTTGGCCCAATTGTTACAATGATAATGATAGGCTTTATTCTACTC
TGCTCAACGCCCTCGACTGGTTTTATTTTTATAGGGTGTAGCTCTATTATATTACCAGCT
TTGTTATACGCGATGCATTCAAACAAAAAACAATTCGAACGTGTATTTGGAAAAGGGAGA
AAAAAGCCAATGAAGGTAAAAAAAGGAGGCGGGAAAATATCTAGATTTTTTAAAGAAGTA
GACAAA---------------------------
>AMZ160 .
------------------------------TTAAGGAAAATTTCCCTGTGTTCTATTTTC
GTATTGGCATATCTCAGTTCTGCTAAGTGCGCGTACCGAGACGAATCTGACATATTGTAC
AGCGACGACTCCAGATCCGATTTAGCAAGCAACGGGGCACCCACCGACAGCTACGAATCT
TTAACAGCAAGTAGTGAGTCTCTAGCAGAAAGCAACGATGCACCCAGCAACAGCTATGAA
TCTTTTCCAGAAATTAGAGAAAATCTAACCGCAAGTGAGGAATCCCTAACATCATGTGAG
GAATCCCTAACAGGAAGTAATGAATCCCTAACAGGAAGTAATGAATCCCTAACAGGAAGT
AAT---------------------GAATCCCTAACAGGAAGTAATGAATCCCTAACAGGA
AGTAATGAATCCCTAACAGGAAGTAATGAATCCCTAACAGGAAGTAATGAATCCCTAACA
GAAAGTAGAGAATCTCTAGAGGCCAGTAGAGAATCGCTAAGAGCAAGTAGAGAGTCTCTA
GCGGCCAGTAGAGAATCCCTGAACGACTTTTGTGGGAGCGAAGAATCAGTAGCATTCGAA
GGAGAGCCAAATGAAAAGACATTCATGGGAGACGTCTTAAGTGGTGGAGAATGTGAGAAT
AGTCTCTCAAGAGAAGATTTATTTCATATAGAAGTAGGATCCGAAGAATCGCTAGATGAT
GCCTCAAAATATAATTTCCAAAAGGATTTATCAACTAGCGATAATAGTTTATTCGAAGAT
GATCAGTCATTGAAAAGGGGACTCAAAAGGAACAGCTCAGTATCTAGTCTGGACAGCGAT
ATGGGAAGTTATAAAAATGGAAGATCTAGAGACAGATTGGATATATATTCGGACCTACCT
AGAAGGCTAATACATGGGGATAATGCACCACAAGAAAAGGAGAAATGTCTGGATTTCAAA
GAACTTCTAGAGAGAGAAAGGAACAACCACCAAAGGAATAGTGTAAATATAAAATTAAAA
AATATATACGGAGAAGAACAATTGTATAATGTGAAGAAAGATAGCTTTATTGTAAAATTG
ATATACTATCTGCCGTTTGGCCCAATTGTTACAATGATAATGATAGGTTTTTTTCTACTC
TGCTGGACCCCCTCGATTGGTTTTACTTTTATAGGGTGTAGCTCTATTATATTACCAGCT
TTGTTATACGCGATGCATTCAAACAAAAAACAATTCGAACGTGTATTTGGAAAAGGGAGA

AAAAAGCCAATGAAGGTAAAAAAAGGAGGCGGGAAAATATCTAGATTTTTTAAAGAAGTA
GACAAA---------------------------
>AMZ161 .
------------------------------TTAAGGAAAATTTCCCTGTGTTCTATTTTC
GTATTGGCATATCTCAGTTCTGCTAAGTGCGCGTACCGAGACGAATCTGACATATTGTAC
AGCGACGACTCCAGATCCGATTTAGCAAGCAACGGGGCACCCACCGACAGCTACGAATCT
TTAACAGCAAGTAGTGAGTCTCTAGCAGAAAGCAACGATGCACCCAGCAACAGCTATGAA
TCTTTTCCAGAAATTAGAGAAAATCTAACCGCAAGTGAGGAATCCCTAACATCATGTGAG
GAATCCCTAACAGGAAGTAATGAATCCCTAACAGGAAGTAATGAATCCCTAACAGGAAGT
AAT---------------------------------------------------------
------------------------------------------------GAATCCCTAACA
GAAAGTAGAGAATCTCTAGAGGCCAGTAGAGAATCGCTAAGAGCAAGTAGAGAGTCTCTA
GCGGCCAGTAGAGAATCCCTGAACGACTTTTGTGGGAGCGAAGAATCAGTAGCATTCGAA
GGAGAGCCAAATGAAAAGACATTCATGGGAGACGTCTTAAGTGGTGGAGAATGTGAGAAT
AGTCTCTCAAGAGAAGATTTATTTCATATAGAAGTAGGATCCGAAGAATCGCTAGATGAT
GCCTCAAAATATAATTTCCAAAAGGATTTATCAACTAGCGATAATAGTTCATTCGAAGAT
GATCAGTCATTGAAAAGGGGACTCAAAAGGAACAGCTCAGTATCTAGTCTGGACAGCGAT
ATGGGAAGTTATAAAAATAAAAGTTATAGACACAGATTGGATATATATTCGGACCTACCT
AGAAGGCCAATACATGGGGATAATGCACCACAAGAAAAGGAGAAATGTCTGGATTTCAAA
GAACTTCTAGAGAGAGAAAGGAACAACCACCAAAGGAATAGTGTAAATATAAAATTAAAA
AATATATACGGAGAAGAACAATTGTATAATGTGAAGAAAGATAGCTTTATTGTAAAATTG
ATATACTATCTGCCGTTTGGCCCAATTGTTACAATGATAATTATAGGCTTTATTCTACTC
TGGTCAACGCCCTGGACTGGTTTTATTTTTATAGGGTGTAGCTCTATTATATTACCAGCT
TTGATATACGCGATGCATTCAAACAAAAAACAATTCGAACGTGTATTTGGAAAAGGGAGA
AAAAAGCCAATGAAGGTAAAAAAAGGAGGCGGGAAAATATCTAGATTTTTTAAAGAAGTA
GACAAA---------------------------
>AMZ170 .
------------------------------TTAAGGAAAATTTCCCTGTGTTCTATTTTC
GTATTGGCATATCTCAGTTCTGCTAAGTGCGCGTACCGAGACGAATCTGACATATTGTAC
AGCGACGACTCCAGATCCGATTTAGCAAGCAACGGGGCACCCACCGACAGCTACGAATCT
TTAACAGCAAGTAGTGAGTCTCTAGCAGAAAGCAACGATGCACCCAGCAACAGCTATGAA
TCTTTTCCAGAAATTAGAGAAAATCTAACCGCAAGTGAGGAATCCCTAACATCATGTGAG
GAATCCCTAACAGGAAGTAATGAATCCCTAACAGGAAGTAATGAATCCCTAACAGGAAGT
AAT---------------------------------------------------------
---------------------------GAATCCCTAACAGGAAGTAATGAATCCCTAACA
GAAAGTAGAGAATCTCTAGAGGCCAGTAGAGAATCGCTAAGAGCAAGTAGAGAGTCTCTA
GCGGCCAGTAGAGAATCCCTGAACGACTTTTGTGGGAGCGAAGAATCAGTAGCATTCGAA
GGAGAGCCAAATGAAAAGACATTCATGGGAGACGTCTTAAGTGGTGGAGAATGTGAGAAT
AGTCTCTCAAGAGAAGATTTATTTCATATAGAAGTAGGATCCGAAGAATCGCTAGATGAT
GCCTCAAAATATAATTTCCAAAAGGATTTATCAACTAGCGATAATAGTTCATTCGAAGAT
GATCAGTCATTGAAAAGGGGACTCAAAAGGAACAGCTCAGTATCTAGTCTGGACAGCGAT
ATGGGAAGTTATAAAAATGGAAGATCTAGAGACAGATTGGATATATATTCGGACCTACCT
AGAAGGCCAATACATGGGGATAATGCACCACAAGAAAAGGAGAAATGTCTGGATTTCAAA
GAACTTCTAGAGAGAGAAAGGAACAACCACCAAAGGAATAGTGTAAATATAAAATTAAAA
AATATATACGGAGAAGAACAATTGTATAATGTGAAGAAAGATAGCTTTATTGTAAAATTG
ATATACTATCTGCCGTTTGGCCCAATTGTTACAATGATAATGATAGGCTTTATTCTACTC

TGCTCAACGCCCTCGACTGGTTTTATTTTTATAGGGTGTAGCTCTATTATATTACCAGCT
TTGTTATACGCGATGCATTCAAACAAAAAACAATTCGAACGTGTATTTGGAAAAGGGAGA
AAAAAGCCAATGAAGGTAAAAAAAGGAGGCGGGAAAATATCTAGATTTTTTAAAGAAGTA
GACAAA---------------------------
>AMZ172 .
------------------------------TTAAGGAAAATTTCCCTGTGTTCTATTTTC
GTATTGGCATATCTCAGTTCTGCTAAGTGCGCGTACCGAGACGAATCTGACATATTGTAC
AGCGACGACTCCAGATCCGATTTAGCAAGCAACGGGGCACCCACCGACAGCTACGAATCT
TTAACAGCAAGTAGTGAGTCTCTAGCAGAAAGCAACGATGCACCCAGCAACAGCTATGAA
TCTTTTCCAGAAATTAGAGAAAATCTAACCGCAAGTGAGGAATCCCTAACATCATGTGAG
GAATCCCTAACAGGAAGTAATGAATCCCTAACAGGAAGTAATGAATCCCTAACAGGAAGT
AAT---------------------------------------------------------
---------------------------GAATCCCTAACAGGAAGTAATGAATCCCTAACA
GAAAGTAGAGAATCTCTAGAGGCCAGTAGAGAATCGGTAAGAGCAAGTAGAGAGTCTCTA
GCGGCCAGTAGAGAATCCCTGAACGACTTTTGTGGGAGCGAAGAATCAGTAGCATTCGAA
GGAGAGCCAAATGAAAAGACATTCATGGGAGACGTCTTAAGTGGTGGAGAATGTGAGAAT
AGTCTCTCAAGAGAAGATTTATTTCATATAGAAGTAGGATCCGAAGAATCGCTAGATGAT
GCCTCAAAATATAATTTCCAAAAGGATTTATCAACTAGCGATAATAGTTCATTCGAAGAT
GATCAGTCATTGAAAAGGGGACTCAAAAGGAACAGCTCAGTATCTAGTCTGGACAGCGAT
ATGGGAAGTTATAAAAATGGAAGATCTAGAGACAGATTGGATATATATTCGGACCTACCT
AGAAGGCCAATACATGGGGATAATGCACCACAAGAAAAGGAGAAATGTCTGGATTTCAAA
GAACTTCTAGAGAGAGAAAGGAACAACCACCAAAGGAATAGTGTAAATATAAAATTAAAA
AATATATACGGAGAAGAACAATTGTATAATGTGAAGAAAGATAGCTTTATTGTAAAATTG
ATATACTATCTGCCGTTTGGCCCAATTGTTACAATGATAATGATAGGCTTTATTCTACTC
TGCTCAACGCCCTCGACTGGTTTTATTTTTATAGGGTGTAGCTCTATTATATTACCAGCT
TTGTTATACGCGATGCATTCAAACAAAAAACAATTCGAACGTGTATTTGGAAAAGGGAGA
AAAAAGCCAATGAAGGTAAAAAAAGGAGGCGGGAAAATATCTAGATTTTTTAAAGAAGTA
GACAAA---------------------------
>AMZ209 .
------------------------------TTAAGGAAAATTTCCCTGTGTTCTATTTTC
GTATTGGCATATCTCAGTTCTGCTAAGTGCGCGTACCGAGACGAATCTGACATATTGTAC
AGCGACGACTCCAGATCCGATTTAGCAAGCAACGGGGCACCCACCGACAGCTACGAATCT
TTAACAGCAAGTAGTGAGTCTCTAGCAGAAAGCAACGATGCACCCAGCAACAGCTATGAA
TCTTTTCCAGAAATTAGAGAAAATCTAACCGCAAGTGAGGAATCCCTAACATCATGTGAG
GAATCCCTAACAGGAAGTAATGAATCCCTAACAGGAAGTAATGAATCCCTAACAGGAAGT
AAT---------------------------------------------------------
---------------------------GAATCCCTAACAGGAAGTAATGAATCCCTAACA
GAAAGTAGAGAATCTCTAGAGGCCAGTAGAGAATCGCTAAGAGCAAGTAGAGAGTCTCTA
GCGGCCAGTAGAGAATCCCTGAACGACTTTTGTGGGAGCGAAGAATCAGTAGCATTCGAA
GGAGAGCCAAATGAAAAGACATTCATGGGAGACGTCTTAAGTGGTGGAGAATGTGAGAAT
AGTCTCTCAAGAGAAGATTTATTTCATATAGAAGTAGGATCCGAAGAATCGCTAGATGAT
GCCTCAAAATATAATTTCCAAAAGGATTTATCAACTAGCGATAATAGTTCATTCGAAGAT
GATCAGTCATTGAAAAGGGGACTCAAAAGGAACAGCTCAGTATCTAGTCTGGACAGCGAT
ATGGGAAGTTATAAAAATGGAAGATCTAGAGACAGATTGGATATATATTCGGACCTACCT
AGAAGGCCAATACATGGGGATAATGCACCACAAGAAAAGGAGAAATGTCTGGATTTCAAA
GAACTTCTAGAGAGAGAAAGGAACAACCACCAAAGGAATAGTGTAAATATAAAATTAAAA
AATATATACGGAGAAGAACAATTGTATAATGTGAAGAAAGATAGCTTTATTGTAAAATTG
ATATACTATCTGCCGTTTGGCCCAATTGTTACAATGATAATGATAGGCTTTATTCTACTC
TGCTCAACGCCCTCGACTGGTTTTATTTTTATAGGGTGTAGCTCTATTATATTACCAGCT
TTGTTATACGCGATGCATTCAAACAAAAAACAATTCGAACGTGTATTTGGAAAAGGGAGA

AAAAAGCCAATGAAGGTAAAAAAAGGAGGCGGGAAAATATCTAGATTTTTTAAAGAAGTA
GACAAA---------------------------
>AMZ211 .
------------------------------TTAAGGAAAATTTCCCTGTGTTCTATTTTC
GTATTGGCATATCTCAGTTCTGCTAAGTGCGCGTACCGAGACGAATCTGACATATTGTAC
AGCGACGACTCCAGATCCGATTTAGCAAGCAACGGGGCACCCACCGACAGCTACGAATCT
TTAACAGCAAGTAGTGAGTCTCTAGCAGAAAGCAACGATGCACCCAGCAACAGCTATGAA
TCTTTTCCAGAAATTAGAGAAAATCTAACCGCAAGTGAGGAATCCCTAACATCATGTGAG
GAATCCCTAACAGGAAGTAATGAATCCCTAACAGGAAGTAATGAATCCCTAACAGGAAGT
AAT---------------------------------------------------------
---------------------------GAATCCCTAACAGGAAGTAATGAATCCCTAACA
GAAAGTAGAGAATCTCTAGAGGCCAGTAGAGAATCGCTAAGAGCAAGTAGAGAGTCTCTA
GCGGCCAGTAGAGAATCCCTGAACGACTTTTGTGGGAGCGAAGAATCAGTAGCATTCGAA
GGAGAGCCAAATGAAAAGACATTCATGGGAGACGTCTTAAGTGGTGGAGAATGTGAGAAT
AGTCTCTCAAGAGAAGATTTATTTCATATAGAAGTAGGATCCGAAGAATCGCTAGATGAT
GCCTCAAAATATAATTTCCAAAAGGATTTATCAACTAGCGATAATAGTTCATTCGAAGAT
GATCAGTCATTGAAAAGGGGACTCAAAAGGAACAGCTCAGTATCTAGTCTGGACAGCGAT
ATGGGAAGTTATAAAAAATAAAAGTTATAGACACAGATTGGATATATATTCGGACCTACCT
AGAAGGCCAATACATGGGGATAATGCACCACAAGAAAAGGAGAAATGTCTGGATTTCAAA
GAACTTCTAGAGAGAGAAAGGAACAACCACCAAAGGAATAGTGTAAATATAAAATTAAAA
AATATATACGGAGAAGAACAATTGTATAATGTGAAGAAAGATAGCTTTATTGTAAAATTG
ATATACTATCTGCCGTTTGGCCCAATTGTTACAATGATAATGATAGGCTTTATTCTACTC
TGCTCAACGCCCTCGACTGGTTTTATTTTTATAGGGTGTAGCTCTATTATATTACCAGCT
TTGTTATACGCGATGCATTCAAACAAAAAACAATTCGAACGTGTATTTGGAAAAGGGAGA
AAAAAGCCAATGAAGGTAAAAAAAGGAGGCGGGAAAATATCTAGATTTTTTAAAGAAGTA
GACAAA---------------------------
>AMZ215 .
------------------------------TTAAGGAAAATTTCCCTGTGTTCTATTTTC
GTATTGGCATATCTCAGTTCTGCTAAGTGCGCGTACCGAGACGAATCTGACATATTGTAC
AGCGACGACTCCAGATCCGATTTAGCAAGCAACGGGGCACCCACCGACAGCTACGAATCT
TTAACAGCAAGTAGTGAGTCTCTAGCAGAAAGCAACGATGCACCCAGCAACAGCTATGAA
TCTTTTCCAGAAATTAGAGAAAATCTAACCGCAAGTGAGGAATCCCTAACATCATGTGAG
GAATCCCTAACAGGAAGTAATGAATCCCTAACAGGAAGTAATGAATCCCTAACAGGAAGT
AAT---------------------------------------------------------
---------------------------GAATCCCTAACAGGAAGTAATGAATCCCTAACA
GAAAGTAGAGAATCTCTAGAGGCCAGTAGAGAATCGCTAAGAGCAAGTAGAGAGTCTCTA
GCGGCCAGTAGAGAATCCCTGAACGACTTTTGTGGGAGCGAAGAATCAGTAGCATTCGAA
GGAGAGCCAAATGAAAAGACATTCATGGGAGACGTCTTAAGTGGTGGAGAATGTGAGAAT
AGTCTCTCAAGAGAAGATTTATTTCATATAGAAGTAGGATCCGAAGAATCGCTAGATGAT
GCCTCAAAATATAATTTCCAAAAGGATTTATCAACTAGCGATAATAGTTCATTCGAAGAT
GATCAGTCATTGAAAAGGGGACTCAAAAGGAACAGCTCAGTATCTAGTCTGGACAGCGAT
ATGGGAAGTTATAAAAAATGGAAGATATAGACACAGATTGGATATATATTCGGACCTACCT
AGAAGGCCAATACATGGGGATAATGCACCACAAGAAAAGGAGAAATGTCTGGATTTCAAA
GAACTTCTAGAGAGAGAAAGGAACAACCACCAAAGGAATAGTGTAAATATAAAATTAAAA
AATATATACGGAGAAGAACAATTGTATAATGTGAAGAAAGATAGCTTTATTGTAAAATTG
ATATACTATCTGCCGTTTGGCCCAATTGTTACAATGATAATGATAGGCTTTATTCTACTC

TGCTCAACGCCCTCGACTGGTTTTATTTTTATAGGGTGTAGCTCTATTATATTACCAGCT
TTGTTATACGCGATGCATTCAAACAAAAAACAATTCGAACGTGTATTTGGAAAAGGGAGA
AAAAAGCCAATGAAGGTAAAAAAAGGAGGCGGGAAAATATCTAGATTTTTTAAAGAAGTA
GACAAA---------------------------
>AMZ284 .
------------------------------TTAAGGAAAATTTCCCTGTGTTCTATTTTC
GTATTGGCATATCTCAGTTCTGCTAAGTGCGCGTACCGAGACGAATCTGACATATTGTAC
AGCGACGACTCCAGATCCGATTTAGCAAGCAACGGGGCACCCACCGACAGCTACGAATCT
TTAACAGCAAGTAGTGAGTCTCTAGCAGAAAGCAACGATGCACCCAGCAACAGCTATGAA
TCTTTTCCAGAAATTAGAGAAAATCTAACCGCAAGTGAGGAATCCCTAACATCATGTGAG
GAATCCCTAACAGGAAGTAATGAATCCCTAACAGGAAGTAATGAATCCCTAACAGGAAGT
AAT---------------------------------------------------------
---------------------------GAATCCCTAACAGGAAGTAATGAATCCCTAACA
GAAAGTAGAGAATCTCTAGAGGCCAGTAGAGAATCGCTAAGAGCAAGTAGAGAGTCTCTA
GCGGCCAGTAGAGAATCCCTGAACGACTTTTGTGGGAGCGAAGAATCAGTAGCATTCGAA
GGAGAGCCAAATGAAAAGACATTCATGGGAGACGTCTTAAGTGGTGGAGAATGTGAGAAT
AGTCTCTCAAGAGAAGATTTATTTCATATAGAAGTAGGATCCGAAGAATCGCTAGATGAT
GCCTCAAAATATAATTTCCAAAAGGATTTATCAACTAGCGATAATAGTTCATTCGAAGAT
GATCAGTCATTGAAAAGGGGACTCAAAAGGAACAGCTCAGTATCTAGTCTGGACAGCGAT
ATGGGAAGTTATAAAAATAAAAGTTATAGACACAGATTGGATATATATTCGGACCTACCT
AGAAGGCCAATACATGGGGATAATGCACCACAAGAAAAGGAGAAATGTCTGGATTTCAAA
GAACTTCTAGAGAGAGAAAGGAACAACCACCAAAGGAATAGTGTAAATATAAAATTAAAA
AATATATACGGAGAAGAACAATTGTATAATGTGAAGAAAGATAGCTTTATTGTAAAATTG
ATATACTATCTGCCGTTTGGCCCAATTGTTACAATGATAATGATAGGCTTTATTCTACTC
TGCTCAACGCCCTCGACTGGTTTTATTTTTATAGGGTGTAGCTCTATTATATTACCAGCT
TTGTTATACGCGATGCATTCAAACAAAAAACAATTCGAACGTGTATTTGGAAAAGGGAGA
AAAAAGCCAATGAAGGTAAAAAAAGGAGGCGGGAAAATATCTAGATTTTTTAAAGAAGTA
GACAAA---------------------------
>AMZ337 .
------------------------------TTAAGGAAAATTTCCCTGTGTTCTATTTTC
GTATTGGCATATCTCAGTTCTGCTAAGTGCGCGTACCGAGACGAATCTGACATATTGTAC
AGCGACGACTCCAGATCCGATTTAGCAAGCAACGGGGCACCCACCGACAGCTACGAATCT
TTAACAGCAAGTAGTGAGTCTCTAGCAGAAAGCAACGATGCACCCAGCAACAGCTATGAA
TCTTTTCCAGAAATTAGAGAAAATCTAACCGCAAGTGAGGAATCCCTAACATCATGTGAG
GAATCCCTAACAGGAAGTAATGAATCCCTAACAGGAAGTAATGAATCCCTAACAGGAAGT
AAT---------------------------------------------------------
---------------------------GAATCCCTAACAGGAAGTAATGAATCCCTAACA
GAAAGTAGAGAATCTCTAGAGGCCAGTAGAGAATCGCTAAGAGCAAGTAGAGAGTCTCTA
GCGGCCAGTAGAGAATCCCTGAACGACTTTTGTGGGAGCGAAGAATCAGTAGCATTCGAA
GGAGAGCCAAATGAAAAGACATTCATGGGAGACGTCTTAAGTGGTGGAGAATGTGAGAAT
AGTCTCTCAAGAGAAGATTTATTTCATATAGAAGTAGGATCCGAAGAATCGCTAGATGAT
GCCTCAAAATATAATTTCCAAAAGGATTTATCAACTAGCGATAATAGTTCATTCGAAGAT
GATCAGTCATTGAAAAGGGGACTCAAAAGGAACAGCTCAGTATCTAGTCTGGACAGCGAT
ATGGGAAGTTATAAAAATGGAAGATCTAGAGACAGATTGGATATATATTCGGACCTACCT
AGAAGGCCAATACATGGGGATAATGCACCACAAGAAAAGGAGAAATGTCTGGATTTCAAA
GAACTTCTAGAGAGAGAAAGGAACAACCACCAAAGGAATAGTGTAAATATAAAATTAAAA
AATATATACGGAGAAGAACAATTGTATAATGTGAAGAAAGATAGCTTTATTGTAAAATTG
ATATACTATCTGCCGTTTGGCCCAATTGTTACAATGATAATGATAGGCTTTATTCTACTC
TGCTCAACGCCCTCGACTGGTTTTATTTTTATAGGGTGTAGCTCTATTATATTACCAGCT
TTGTTATACGCGATGCATTCAAACAAAAAACAATTCGAACGTGTATTTGGAAAAGGGAGA

AAAAAGCCAATGAAGGTAAAAAAAGGAGGCGGGAAAATATCTAGATTTTTTAAAGAAGTA
GACAAA---------------------------
>AMZ494 .
------------------------------TTAAGGAAAATTTCCCTGTGTTCTATTTTC
GTATTGGCATATCTCAGTTCTGCTAAGTGCGCGTACCGAGACGAATCTGACATATTGTAC
AGCGACGACTCCAGATCCGATTTAGCAAGCAACGGGGCACCCACCGACAGCTACGAATCT
TTAACAGCAAGTAGTGAGTCTCTAGCAGAAAGCAACGATGCACCCAGCAACAGCTATGAA
TCTTTTCCAGAAATTAGAGAAAATCTAACCGCAAGTGAGGAATCCCTAACATCATGTGAG
GAATCCCTAACAGGAAGTAATGAATCCCTAACAGGAAGTAATGAATCCCTAACAGGAAGT
AAT---------------------------------------------------------
------------------------------------------------GAATCCCTAACA
GAAAGTAGAGAATCTCTAGAGGCCAGTAGAGAATCGCTAAGAGCAAGTAGAGAGTCTCTA
GCGGCCAGTAGAGAATCCCTGAACGACTTTTGTGGGAGCGAAGAATCAGTAGCATTCGAA
GGAGAGCCAAATGAAAAGACATTCATGGGAGACGTCTTAAGTGGTGGAGAATGTGAGAAT
AGTCTCTCAAGAGAAGATTTATTTCATATAGAAGTAGGATCCGAAGAATCGCTAGATGAT
GCCTCAAAATATAATTTCCAAAAGGATTTATCAACTAGCGATAATAGTTTATTCGAAGAT
GATCAGTCATTGAAAAGGGGACTCAAAAGGAACAGCTCAGTATCTAGTCTGGACAGCGAT
ATGGGAAGTTATAAAAATGGAAGATCTAGAGACAGATTGGATATATATTCGGACCTACCT
AGAAGGCTAATACATGGGGATAATGCACCACAAGAAAAGGAGAAATGTCTGGATTTCAAA
GAACTTCTAGAGAGAGAAAGGAACAACCACCAAAGGAATAGTGTAAATATAAAATTAAAA
AATATATACGGAGAAGAACAATTGTATAATGTGAAGAAAGATAGCTTTATTGTAAAATTG
ATATACTATCTGCCGTTTGGCCCAATTGTTACAATGATAATGATAGGTTTTTTTCTACTC
TGCTGGACCCCCTCGATTGGTTTTATTTTTATAGGGTGTAGCTCTATTATATTACCAGCT
TTGTTATACGCGATGCATTCAAACAAAAAACAATTCGAACGTGTATTTGGAAAAGGGAGA
AAAAAGCCAATGAAGGTAAAAAAAGGAGGCGGGAAAATATCTAGATTTTTTAAAGAAGTA
GACAAA---------------------------
>AMZ503 .
------------------------------TTAAGGAAAATTTCCCTGTGTTCTATTTTC
GTATTGGCATATCTCAGTTCTGCTAAGTGCGCGTACCGAGACGAATCTGACATATTGTAC
AGCGACGACTCCAGATCCGATTTAGCAAGCAACGGGGCACCCACCGACAGCTACGAATCT
TTAACAGCAAGTAGTGAGTCTCTAGCAGAAAGCAACGATGCACCCAGCAACAGCTATGAA
TCTTTTCCAGAAATTAGAGAAAATCTAACCGCAAGTGAGGAATCCCTAACATCATGTGAG
GAATCCCTAACAGGAAGTAATGAATCCCTAACAGGAAGTAATGAATCCCTAACAGGAAGT
AAT---------------------GAATCCCTAACAGGAAGTAATGAATCCCTAACAGGA
AGTAATGAATCCCTAACAGGAAGTAATGAATCCCTAACAGGAAGTAATGAATCCCTAACA
GAAAGTAGAGAATCTCTAGAGGCCAGTAGAGAATCGCTAAGAGCAAGTAGAGAGTCTCTA
GCGGCCAGTAGAGAATCCCTGAACGACTTTTGTGGGAGCGAAGAATCAGTAGCATTCGAA
GGAGAGCCAAATGAAAAGACATTCATGGGAGACGTCTTAAGTGGTGGAGAATGTGAGAAT
AGTCTCTCAAGAGAAGATTTATTTCATATAGAAGTAGGATCCGAAGAATCGCTAGATGAT
GCCTCAAAATATAATTTCCAAAAGGATTTATCAACTAGCGATAATAGTTTATTCGAAGAT
GATCAGTCATTGAAAAGGGGACTCAAAAGGAACAGCTCAGTATCTAGTCTGGACAGCGAT
ATGGGAAGTTATAAAAATGGAAGATCTAGAGACAGATTGGATATATATTCGGACCTACCT
AGAAGGCTAATACATGGGGATAATGCACCACAAGAAAAGGAGAAATGTCTGGATTTCAAA
GAACTTCTAGAGAGAGAAAGGAACAACCACCAAAGGAATAGTGTAAATATAAAATTAAAA
AATATATACGGAGAAGAACAATTGTATAATGTGAAGAAAGATAGCTTTATTGTAAAATTG
ATATACTATCTGCCGTTTGGCCCAATTGTTACAATGATAATGATAGGTTTTTTTCTACTC

TGCTGGACCCCCTCGATTGGTTTTACTTTTATAGGGTGTAGCTCTATTATATTACCAGCT
TTGTTATACGCGATGCATTCAAACAAAAAACAATTCGAACGTGTATTTGGAAAAGGGAGA
AAAAAGCCAATGAAGGTAAAAAAAGGAGGCGGGAAAATATCTAGATTTTTTAAAGAAGTA
GACAAA---------------------------
>AMZ506 .
------------------------------TTAAGGAAAATTTCCCTGTGTTCTATTTTC
GTATTGGCATATCTCAGTTCTGCTAAGTGCGCGTACCGAGACGAATCTGACATATTGTAC
AGCGACGACTCCAGATCCGATTTAGCAAGCAACGGGGCACCCACCGACAGCTACGAATCT
TTAACAGCAAGTAGTGAGTCTCTAGCAGAAAGCAACGATGCACCCAGCAACAGCTATGAA
TCTTTTCCAGAAATTAGAGAAAATCTAACCGCAAGTGAGGAATCCCTAACATCATGTGAG
GAATCCCTAACAGGAAGTAATGAATCCCTAACAGGAAGTAATGAATCCCTAACAGGAAGT
AAT---------------------------------------------------------
---------------------------GAATCCCTAACAGGAAGTAATGAATCCCTAACA
GAAAGTAGAGAATCTCTAGAGGCCAGTAGAGAATCGCTAAGAGCAAGTAGAGAGTCTCTA
GCGGCCAGTAGAGAATCCCTGAACGACTTTTGTGGGAGCGAAGAATCAGTAGCATTCGAA
GGAGAGCCAAATGAAAAGACATTCATGGGAGACGTCTTAAGTGGTGGAGAATGTGAGAAT
AGTCTCTCAAGAGAAGATTTATTTCATATAGAAGTAGGATCCGAAGAATCGCTAGATGAT
GCCTCAAAATATAATTTCCAAAAGGATTTATCAACTAGCGATAATAGTTCATTCGAAGAT
GATCAGTCATTGAAAAGGGGACTCAAAAGGAACAGCTCAGTATCTAGTCTGGACAGCGAT
ATGGGAAGTTATAAAAATAAAAGTTATAGACACAGATTGGATATATATTCGGACCTACCT
AGAAGGCCAATACATGGGGATAATGCACCACAAGAAAAGGAGAAATGTCTGGATTTCAAA
GAACTTCTAGAGAGAGAAAGGAACAACCACCAAAGGAATAGTGTAAATATAAAATTAAAA
AATATATACGGAGAAGAACAATTGTATAATGTGAAGAAAGATAGCTTTATTGTAAAATTG
ATATACTATCTGCCGTTTGGCCCAATTGTTACAATGATAATGATAGGCTTTATTCTACTC
TGCTCAACGCCCTCGACTGGTTTTATTTTTATAGGGTGTAGCTCTATTATATTACCAGCT
TTGTTATACGCGATGCATTCAAACAAAAAACAATTCGAACGTGTATTTGGAAAAGGGAGA
AAAAAGCCAATGAAGGTAAAAAAAGGAGGCGGGAAAATATCTAGATTTTTTAAAGAAGTA
GACAAA---------------------------
>AMZ517 .
------------------------------TTAAGGAAAATTTCCCTGTGTTCTATTTTC
GTATTGGCATATCTCAGTTCTGCTAAGTGCGCGTACCGAGACGAATCTGACATATTGTAC
AGCGACGACTCCAGATCCGATTTAGCAAGCAACGGGGCACCCACCGACAGCTACGAATCT
TTAACAGCAAGTAGTGAGTCTCTAGCAGAAAGCAACGATGCACCCAGCAACAGCTATGAA
TCTTTTCCAGAAATTAGAGAAAATCTAACCGCAAGTGAGGAATCCCTAACATCATGTGAG
GAATCCCTAACAGGAAGTAATGAATCCCTAACAGGAAGTAATGAATCCCTAACAGGAAGT
AAT------------------------------------------GAATCCCTAACAGGA
AGTAATGAATCCCTAACAGGAAGTAATGAATCCCTAACAGGAAGTAATGAATCCCTAACA
GAAAGTAGAGAATCTCTAGAGGCCAGTAGAGAATCGCTAAGAGCAAGTAGAGAGTCTCTA
GCGGCCAGTAGAGAATCCCTGAACGACTTTTGTGGGAGCGAAGAATCAGTAGCATTCGAA
GGAGAGCCAAATGAAAAGACATTCATGGGAGACGTCTTAAGTGGTGGAGAATGTGAGAAT
AGTCTCTCAAGAGAAGATTTATTTCATATAGAAGTAGGATCCGAAGAATCGCTAGATGAT
GCCTCAAAATATAATTTCCAAAAGGATTTATCAACTAGCGATAATAGTTCATTCGAAGAT
GATCAGTCATTGAAAAGGGGACTCAAAAGGAACAGCTCAGTATCTAGTCTGGACAGCGAT
ATGGGAAGTTATAAAAATAAAAGTTATAGACACAGATTGGATATATATTCGGACCTACCT
AGAAGGCCAATACATGGGGATAATGCACCACAAGAAAAGGAGAAATGTCTGGATTTCAAA
GAACTTCTAGAGAGAGAAAGGAACAACCACCAAAGGAATAGTGTAAATATAAAATTAAAA
AATATATACGGAGAAGAACAATTGTATAATGTGAAGAAAGATAGCTTTATTGTAAAATTG
ATATACTATCTGCCGTTTGGCCCAATTGTTACAATGATAATGATAGGTTTTTTTCTACTC
TGCTGGACCCCCTCGATTGGTTTTACTTTTATAGGGTGTAGCTCTATTATATTACCAGCT
TTGTTATACGCGATGCATTCAAACAAAAAACAATTCGAACGTGTATTTGGAAAAGGGAGA

AAAAAGCCAATGAAGGTAAAAAAAGGAGGCGGGAAAATATCTAGATTTTTTAAAGAAGTA
GACAAA---------------------------
>AMZ534 .
------------------------------TTAAGGAAAATTTCCCTGTGTTCTATTTTC
GTATTGGCATATCTCAGTTCTGCTAAGTGCGCGTACCGAGACGAATCTGACATATTGTAC
AGCGACGACTCCAGATCCGATTTAGCAAGCAACGGGGCACCCACCGACAGCTACGAATCT
TTAACAGCAAGTAGTGAGTCTCTAGCAGAAAGCAACGATGCACCCAGCAACAGCTATGAA
TCTTTTCCAGAAATTAGAGAAAATCTAACCGCAAGTGAGGAATCCCTAACATCATGTGAG
GAATCCCTAACAGGAAGTAATGAATCCCTAACAGGAAGTAATGAATCCCTAACAGGAAGT
AAT---------------------------------------------------------
---------------------------GAATCCCTAACAGGAAGTAATGAATCCCTAACA
GAAAGTAGAGAATCTCTAGAGGCCAGTAGAGAATCGCTAAGAGCAAGTAGAGAGTCTCTA
GCGGCCAGTAGAGAATCCCTGAACGACTTTTGTGGGAGCGAAGAATCAGTAGCATTCGAA
GGAGAGCCAAATGAAAAGACATTCATGGGAGACGTCTTAAGTGGTGGAGAATGTGAGAAT
AGTCTCTCAAGAGAAGATTTATTTCATATAGAAGTAGGATCCGAAGAATCGCTAGATGAT
GCCTCAAAATATAATTTCCAAAAGGATTTATCAACTAGCGATAATAGTTCATTCGAAGAT
GATCAGTCATTGAAAAGGGGACTCAAAAGGAACAGCTCAGTATCTAGTCTGGACAGCGAT
ATGGGAAGTTATAAAAATAAAAGTTATAGACACAGATTGGATATATATTCGGACCTACCT
AGAAGGCCAATACATGGGGATAATGCACCACAAGAAAAGGAGAAATGTCTGGATTTCAAA
GAACTTCTAGAGAGAGAAAGGAACAACCACCAAAGGAATAGTGTAAATATAAAATTAAAA
AATATATACGGAGAAGAACAATTGTATAATGTGAAGAAAGATAGCTTTATTGTAAAATTG
ATATACTATCTGCCGTTTGGCCCAATTGTTACAATGATAATGATAGGCTTTATTCTACTC
TGCTCAACGCCCTCGACTGGTTTTATTTTTATAGGGTGTAGCTCTATTATATTACCAGCT
TTGTTATACGCGATGCATTCAAACAAAAAACAATTCGAACGTGTATTTGGAAAAGGGAGA
AAAAAGCCAATGAAGGTAAAAAAAGGAGGCGGGAAAATATCTAGATTTTTTAAAGAAGTA
GACAAA---------------------------
>AMZ551 .
------------------------------TTAAGGAAAATTTCCCTGTGTTCTATTTTC
GTATTGGCATATCTCAGTTCTGCTAAGTGCGCGTACCGAGACGAATCTGACATATTGTAC
AGCGACGACTCCAGATCCGATTTAGCAAGCAACGGGGCACCCACCGACAGCTACGAATCT
TTAACAGCAAGTAGTGAGTCTCTAGCAGAAAGCAACGATGCACCCAGCAACAGCTATGAA
TCTTTTCCAGAAATTAGAGAAAATCTAACCGCAAGTGAGGAATCCCTAACATCATGTGAG
GAATCCCTAACAGGAAGTAATGAATCCCTAACAGGAAGTAATGAATCCCTAACAGAAAGT
AAT---------------------------------------------------------
------GAATCCCTAACAGGAAGTAATGAATCCCTAACAGGAAGTAATGAATCCCTAACA
GAAAGTAGAGAATCTCTAGAGGCCAGTAGAGAATCGCTAAGAGCAAGTAGAGAGTCTCTA
GCGGCCAGTAGAGAATCCCTGAACGACTTTTGTGGGAGCGAAGAATCAGTAGCATTCGAA
GGAGAGCCAAATGAAAAGACATTCATGGGAGACGTCTTAAGTGGTGGAGAATGTGAGAAT
AGTCTCTCAAGAGAAGATTTATTTCATATAGAAGTAGGATCCGAAGAATCGCTAGATGAT
GCCTCAAAATATAATTTCCAAAAGGATTTATCAACTAGCGATAATAGTTTATTCGAAGAT
GATCAGTCATTGAAAAGGGGACTCAAAAGGAACAGCTCAGTATCTAGTCTGGACAGCGAT
ATGGGAAGTTATAAAAATGGAAGATCTAGAGACAGATTGGATATATATTCGGACCTACCT
AGAAGGCTAATACATGGGGATAATGCACCACAAGAAAAGGAGAAATGTCTGGATTTCAAA
GAACTTCTAGAGAGAGAAAGGAACAACCACCAAAGGAATAGTGTAAATATAAAATTAAAA
AATATATACGGAGAAGAACAATTGTATAATGTGAAGAAAGATAGCTTTATTGTAAAATTG
ATATACTATCTGCCGTTTGGCCCAATTGTTACAATGATAATGATAGGTTTTTTTCTACTC

```
TGCTGGACCCCCTCGATTGGTTTTATTTTTATAGGGTGTAGCTCTATTATATTACCAGCT
TTGTTATACGCGATGCATTCAAACAAAAAACAATTCGAACGTGTATTTGGAAAAGGGAGA
AAAAAGCCAATGAAGGTAAAAAAAGGAGGCGGGAAAATATCTAGATTTTTTAAAGAAGTA
GACAAA---------------------------
>AMZ568 .
------------------------------TTAAGGAAAATTTCCCTGTGTTCTATTTTC
GTATTGGCATATCTCAGTTCTGCTAAGTGCGCGTACCGAGACGAATCTGACATATTGTAC
AGCGACGACTCCAGATCCGATTTAGCAAGCAACGGGGCACCCACCGACAGCTACGAATCT
TTAACAGCAAGTAGTGAGTCTCTAGCAGAAAGCAACGATGCACCCAGCAACAGCTATGAA
TCTTTTCCAGAAATTAGAGAAAATCTAACCGCAAGTGAGGAATCCCTAACATCATGTGAG
GAATCCCTAACAGGAAGTAATGAATCCCTAACAGGAAGTAATGAATCCCTAACAGGAAGT
AAT---------------------------------------------------------
---------------------------GAATCCCTAACAGGAAGTAATGAATCCCTAACA
GAAAGTAGAGAATCTCTAGAGGCCAGTAGAGAATCGCTAAGAGCAAGTAGAGAGTCTCTA
GCGGCCAGTAGAGAATCCCTGAACGACTTTTGTGGGAGCGAAGAATCAGTAGCATTCGAA
GGAGAGCCAAATGAAAAGACATTCATGGGAGACGTCTTAAGTGGTGGAGAATGTGAGAAT
AGTCTCTCAAGAGAAGATTTATTTCATATAGAAGTAGGATCCGAAGAATCGCTAGATGAT
GCCTCAAAATATAATTTCCAAAAGGATTTATCAACTAGCGATAATAGTTCATTCGAAGAT
GATCAGTCATTGAAAAGGGGACTCAAAAGGAACAGCTCAGTATCTAGTCTGGACAGCGAT
ATGGGAAGTTATAAAAATAAAAGTTATAGACACAGATTGGATATATATTCGGACCTACCT
AGAAGGCCAATACATGGGGATAATGCACCACAAGAAAAGGAGAAATGTCTGGATTTCAAA
GAACTTCTAGAGAGAGAAAGGAACAACCACCAAAGGAATAGTGTAAATATAAAATTAAAA
AATATATACGGAGAAGAACAATTGTATAATGTGAAGAAAGATAGCTTTATTGTAAAATTG
ATATACTATCTGCCGTTTGGCCCAATTGTTACAATGATAATGATAGGCTTTATTCTACTC
TGCTCAACGCCCTCGACTGGTTTTATTTTTATAGGGTGTAGCTCTATTATATTACCAGCT
TTGTTATACGCGATGCATTCAAACAAAAAACAATTCGAACGTGTATTTGGAAAAGGGAGA
AAAAAGCCAATGAAGGTAAAAAAAGGAGGCGGGAAAATATCTAGATTTTTTAAAGAAGTA
GACAAA---------------------------
>AMZ569 .
------------------------------TTAAGGAAAATTTCCCTGTGTTCTATTTTC
GTATTGGCATATCTCAGTTCTGCTAAGTGCGCGTACCGAGACGAATCTGACATATTGTAC
AGCGACGACTCCAGATCCGATTTAGCAAGCAACGGGGCACCCACCGACAGCTACGAATCT
TTAACAGCAAGTAGTGAGTCTCTAGCAGAAAGCAACGATGCACCCAGCAACAGCTATGAA
TCTTTTCCAGAAATTAGAGAAAATCTAACCGCAAGTGAGGAATCCCTAACATCATGTGAG
GAATCCCTAACAGGAAGTAATGAATCCCTAACAGGAAGTAATGAATCCCTAACAGGAAGT
AAT---------------------------------------------------------
---------------------------GAATCCCTAACAGGAAGTAATGAATCCCTAACA
GAAAGTAGAGAATCTCTAGAGGCCAGTAGAGAATCGCTAAGAGCAAGTAGAGAGTCTCTA
GCGGCCAGTAGAGAATCCCTGAACGACTTTTGTGGGAGCGAAGAATCAGTAGCATTCGAA
GGAGAGCCAAATGAAAAGACATTCATGGGAGACGTCTTAAGTGGTGGAGAATGTGAGAAT
AGTCTCTCAAGAGAAGATTTATTTCATATAGAAGTAGGATCCGAAGAATCGCTAGATGAT
GCCTCAAAATATAATTTCCAAAAGGATTTATCAACTAGCGATAATAGTTCATTCGAAGAT
GATCAGTCATTGAAAAGGGGACTCAAAAGGAACAGCTCAGTATCTAGTCTGGACAGCGAT
ATGGGAAGTTATAAAAATAAAAGTTATAGACACAGATTGGATATATATTCGGACCTACCT
AGAAGGCCAATACATGGGGATAATGCACCACAAGAAAAGGAGAAATGTCTGGATTTCAAA
GAACTTCTAGAGAGAGAAAGGAACAACCACCAAAGGAATAGTGTAAATATAAAATTAAAA
AATATATACGGAGAAGAACAATTGTATAATGTGAAGAAAGATAGCTTTATTGTAAAATTG
ATATACTATCTGCCGTTTGGCCCAATTGTTACAATGATAATGATAGGCTTTATTCTACTC
TGCTCAACGCCCTCGACTGGTTTTATTTTTATAGGGTGTAGCTCTATTATATTACCAGCT
TTGTTATACGCGATGCATTCAAACAAAAAACAATTCGAACGTGTATTTGGAAAAGGGAGA
```

AAAAAGCCAATGAAGGTAAAAAAAGGAGGCGGGAAAATATCTAGATTTTTTAAAGAAGTA
GACAAA---------------------------
>AMZ576 .
------------------------------TTAAGGAAAATTTCCCTGTGTTCTATTTTC
GTATTGGCATATCTCAGTTCTGCTAAGTGCGCGTACCGAGACGAATCTGACATATTGTAC
AGCGACGACTCCAGATCCGATTTAGCAAGCAACGGGGCACCCACCGACAGCTACGAATCT
TTAACAGCAAGTAGTGAGTCTCTAGCAGAAAGCAACGATGCACCCAGCAACAGCTATGAA
TCTTTTCCAGAAATTAGAGAAAATCTAACCGCAAGTGAGGAATCCCTAACA---------
------------GGAAGTAATGAATCCCTAACAGGAAGTAATGAATCCCTAACAGAAAGT
AAT------------------------------------------GAATCCCTAACAGGA
AGTAATGAATCCCTAACAGGAAGTAATGAATCCCTAACAGGAAGTAATGAATCCCTAACA
GAAAGTAGAGAATCTCTAGAGGCCAGTAGAGAATCGCTAAGAGCAAGTAGAGAGTCTCTA
GCGGCCAGTAGAGAATCCCTGAACGACTTTTGTGGGAGCGAAGAATCAGTAGCATGCGAA
GGAGAGCCAAATGAAAAGACATTCATGGGAGACGTCTTAAGTGGTGGAGAATGTGAGAAT
AGTCTCTCAAGAGAAGATTTATTTCATATAGAAGTAGGATCCGAAGAATCGCTAGATGAT
GCCTCAAAATATAATTTCCAAAAGGATTTATCAACTAGCGATAATAGTTTATTCGAAGAT
GATCAGTCATTGAAAAGGGGACTCAAAAGGAACAGCTCAGTATCTAGTCTGGACAGCGAT
ATGGGAAGTTATAAAAATGGAAGATCTAGAGACAGATTGGATATATATTCGGACCTACCT
AGAAGGCTAATACATGGGGATAATGCACCACAAGAAAAGGAGAAATGTCTGGATTTCAAA
GAACTTCTAGAGAGAGAAAGGAACAACCACCAAAGGAATAGTGTAAATATAAAATTAAAA
AATATATACGGAGAAGAACAATTGTATAATGTGAAGAAAGATAGCTTTATTGTAAAATTG
ATATACTATCTGCCGTTTGGCCCAATTGTTACAATGATAATGATAGGTTTTTTTCTACTC
TGCTGGACCCCCTCGATTGGTTTTACTTTTATAGGGTGTAGCTCTATTATATTACCAGCT
TTGTTATACGCGATGCATTCAAACAAAAAACAATTCGAACGTGTATTTGGAAAAGGGAGA
AAAAAGCCAATGAAGGTAAAAAAAGGAGGCGGGAAAATATCTAGATTTTTTAAAGAAGTA
GACAAA---------------------------
>AMZ596 .
------------------------------TTAAGGAAAATTTCCCTGTGTTCTATTTTC
GTATTGGCATATCTCAGTTCTGCTAAGTGCGCGTACCGAGACGAATCTGACATATTGTAC
AGCGACGACTCCAGATCCGATTTAGCAAGCAACGGGGCACCCACCGACAGCTACGAATCT
TTAACAGCAAGTAGTGAGTCTCTAGCAGAAAGCAACGATGCACCCAGCAACAGCTATGAA
TCTTTTCCAGAAATTAGAGAAAATCTAACCGCAAGTGAGGAATCCCTAACATCATGTGAG
GAATCCCTAACAGGAAGTAATGAATCCCTAACAGGAAGTAATGAATCCCTAACAGGAAGT
AAT---------------------------------------------------------
---------------------------GAATCCCTAACAGGAAGTAATGAATCCCTAACA
GAAAGTAGAGAATCTCTAGAGGCCAGTAGAGAATCGCTAAGAGCAAGTAGAGAGTCTCTA
GCGGCCAGTAGAGAATCCCTGAACGACTTTTGTGGGAGCGAAGAATCAGTAGCATTCGAA
GGAGAGCCAAATGAAAAGACATTCATGGGAGACGTCTTAAGTGGTGGAGAATGTGAGAAT
AGTCTCTCAAGAGAAGATTTATTTCATATAGAAGTAGGATCCGAAGAATCGCTAGATGAT
GCCTCAAAATATAATTTCCAAAAGGATTTATCAACTAGCGATAATAGTTCATTCGAAGAT
GATCAGTCATTGAAAAGGGGACTCAAAAGGAACAGCTCAGTATCTAGTCTGGACAGCGAT
ATGGGAAGTTATAAAAATGGAAGATCTAGAGACAGATTGGATATATATTCGGACCTACCT
AGAAGGCCAATACATGGGGATAATGCACCACAAGAAAAGGAGAAATGTCTGGATTTCAAA
GAACTTCTAGAGAGAGAAAGGAACAACCACCAAAGGAATAGTGTAAATATAAAATTAAAA
AATATATACGGAGAAGAACAATTGTATAATGTGAAGAAAGATAGCTTTATTGTAAAATTG
ATATACTATCTGCCGTTTGGCCCAATTGTTACAATGATAATGATAGGCTTTATTCTACTC

TGCTCAACGCCCTCGACTGGTTTTATTTTTATAGGGTGTAGCTCTATTATATTACCAGCT
TTGTTATACGCGATGCATTCAAACAAAAAACAATTCGAACGTGTATTTGGAAAAGGGAGA
AAAAAGCCAATGAAGGTAAAAAAAGGAGGCGGGAAAATATCTAGATTTTTTAAAGAAGTA
GACAAA---------------------------
>AMZ614 .
------------------------------TTAAGGAAAATTTCCCTGTGTTCTATTTTC
GTATTGGCATATCTCAGTTCTGCTAAGTGCGCGTACCGAGACGAATCTGACATATTGTAC
AGCGACGACTCCAGATCCGATTTAGCAAGCAACGGGGCACCCACCGACAGCTACGAATCT
TTAACAGCAAGTAGTGAGTCTCTAGCAGAAAGCAACGATGCACCCAGCAACAGCTATGAA
TCTTTTCCAGAAATTAGAGAAAATCTAACCGCAAGTGAGGAATCCCTAACATCATGTGAG
GAATCCCTAACAGGAAGTAATGAATCCCTAACAGGAAGTAATGAATCCCTAACAGGAAGT
AAT------------------------------------------GAATCCCTAACAGGA
AGTAATGAATCCCTAACAGGAAGTAATGAATCCCTAACAGGAAGTAATGAATCCCTAACA
GAAAGTAGAGAATCTCTAGAGGCCAGTAGAGAATCGCTAAGAGCAAGTAGAGAGTCTCTA
GCGGCCAGTAGAGAATCCCTGAACGACTTTTGTGGGAGCGAAGAATCAGTAGCATTCGAA
GGAGAGCCAAATGAAAAGACATTCATGGGAGACGTCTTAAGTGGTGGAGAATGTGAGAAT
AGTCTCTCAAGAGAAGATTTATTTCATATAGAAGTAGGATCCGAAGAATCGCTAGATGAT
GCCTCAAAATATAATTTCCAAAAGGATTTATCAACTAGCGATAATAGTTCATTCGAAGAT
GATCAGTCATTGAAAAGGGGACTCAAAAGGAACAGCTCAGTATCTAGTCTGGACAGCGAT
ATGGGAAGTTATAAAAATAAAAGTTATAGACACAGATTGGATATATATTCGGACCTACCT
AGAAGGCCAATACATGGGGATAATGCACCACAAGAAAAGGAGAAATGTCTGGATTTCAAA
GAACTTCTAGAGAGAGAAAGGAACAACCACCAAAGGAATAGTGTAAATATAAAATTAAAA
AATATATACGGAGAAGAACAATTGTATAATGTGAAGAAAGATAGCTTTATTGTAAAATTG
ATATACTATCTGCCGTTTGGCCCAATTGTTACAATGATAATGATAGGTTTTTTTCTACTC
TGCTGGACCCCCTCGATTGGTTTTACTTTTATAGGGTGTAGCTCTATTATATTACCAGCT
TTGTTATACGCGATGCATTCAAACAAAAAACAATTCGAACGTGTATTTGGAAAAGGGAGA
AAAAAGCCAATGAAGGTAAAAAAAGGAGGCGGGAAAATATCTAGATTTTTTAAAGAAGTA
GACAAA---------------------------
>AMZ658 .
------------------------------TTAAGGAAAATTTCCCTGTGTTCTATTTTC
GTATTGGCATATCTCAGTTCTGCTAAGTGCGCGTACCGAGACGAATCTGACATATTGTAC
AGCGACGACTCCAGATCCGATTTAGCAAGCAACGGGGCACCCACCGACAGCTACGAATCT
TTAACAGCAAGTAGTGAGTCTCTAGCAGAAAGCAACGATGCACCCAGCAACAGCTATGAA
TCTTTTCCAGAAATTAGAGAAAATCTAACCGCAAGTGAGGAATCCCTAACATCATGTGAG
GAATCCCTAACAGGAAGTAATGAATCCCTAACAGGAAGTAATGAATCCCTAACAGGAAGT
AAT---------------------------------------------------------
---------------------------GAATCCCTAACAGGAAGTAATGAATCCCTAACA
GAAAGTAGAGAATCTCTAGAGGCCAGTAGAGAATCGCTAAGAGCAAGTAGAGAGTCTCTA
GCGGCCAGTAGAGAATCCCTGAACGACTTTTGTGGGAGCGAAGAATCAGTAGCATTCGAA
GGAGAGCCAAATGAAAAGACATTCATGGGAGACGTCTTAAGTGGTGGAGAATGTGAGAAT
AGTCTCTCAAGAGAAGATTTATTTCATATAGAAGTAGGATCCGAAGAATCGCTAGATGAT
GCCTCAAAATATAATTTCCAAAAGGATTTATCAACTAGCGATAATAGTTCATTCGAAGAT
GATCAGTCATTGAAAAGGGGACTCAAAAGGAACAGCTCAGTATCTAGTCTGGACAGCGAT
ATGGGAAGTTATAAAAATAAAAGTTATAGACACAGATTGGATATATATTCGGACCTACCT
AGAAGGCCAATACATGGGGATAATGCACCACAAGAAAAGGAGAAATGTCTGGATTTCAAA
GAACTTCTAGAGAGAGAAAGGAACAACCACCAAAGGAATAGTGTAAATATAAAATTAAAA
AATATATACGGAGAAGAACAATTGTATAATGTGAAGAAAGATAGCTTTATTGTAAAATTG
ATATACTATCTGCCGTTTGGCCCAATTGTTACAATGATAATGATAGGCTTTATTCTACTC
TGCTCAACGCCCTCGACTGGTTTTATTTTTATAGGGTGTAGCTCTATTATATTACCAGCT
TTGTTATACGCGATGCATTCAAACAAAAAACAATTCGAACGTGTATTTGGAAAAGGGAGA

AAAAAGCCAATGAAGGTAAAAAAAGGAGGCGGGAAAATATCTAGATTTTTTAAAGAAGTA
GACAAA---------------------------
>AMZ660 .
------------------------------TTAAGGAAAATTTCCCTGTGTTCTATTTTC
GTATTGGCATATCTCAGTTCTGCTAAGTGCGCGTACCGAGACGAATCTGACATATTGTAC
AGCGACGACTCCAGATCCGATTTAGCAAGCAACGGGGCACCCACCGACAGCTACGAATCT
TTAACAGCAAGTAGTGAGTCTCTAGCAGAAAGCAACGATGCACCCAGCAACAGCTATGAA
TCTTTTCCAGAAATTAGAGAAAATCTAACCGCAAGTGAGGAATCCCTAACATCATGTGAG
GAATCCCTAACAGGAAGTAATGAATCCCTAACAGGAAGTAATGAATCCCTAACAGGAAGT
AAT---------------------------------------------------------
---------------------------GAATCCCTAACAGGAAGTAATGAATCCCTAACA
GAAAGTAGAGAATCTCTAGAGGCCAGTAGAGAATCGCTAAGAGCAAGTAGAGAGTCTCTA
GCGGCCAGTAGAGAATCCCTGAACGACTTTTGTGGGAGCGAAGAATCAGTAGCATTCGAA
GGAGAGCCAAATGAAAAGACATTCATGGGAGACGTCTTAAGTGGTGGAGAATGTGAGAAT
AGTCTCTCAAGAGAAGATTTATTTCATATAGAAGTAGGATCCGAAGAATCGCTAGATGAT
GCCTCAAAATATAATTTCCAAAAGGATTTATCAACTAGCGATAATAGTTCATTCGAAGAT
GATCAGTCATTGAAAAGGGGACTCAAAAGGAACAGCTCAGTATCTAGTCTGGACAGCGAT
ATGGGAAGTTATAAAAATGGAAGATCTAGAGACAGATTGGATATATATTCGGACCTACCT
AGAAGGCCAATACATGGGGATAATGCACCACAAGAAAAGGAGAAATGTCTGGATTTCAAA
GAACTTCTAGAGAGAGAAAGGAACAACCACCAAAGGAATAGTGTAAATATAAAATTAAAA
AATATATACGGAGAAGAACAATTGTATAATGTGAAGAAAGATAGCTTTATTGTAAAATTG
ATATACTATCTGCCGTTTGGCCCAATTGTTACAATGATAATGATAGGCTTTATTCTACTC
TGCTCAACGCCCTCGACTGGTTTTATTTTTATAGGGTGTAGCTCTATTATATTACCAGCT
TTGTTATACGCGATGCATTCAAACAAAAAACAATTCGAACGTGTATTTGGAAAAGGGAGA
AAAAAGCCAATGAAGGTAAAAAAAGGAGGCGGGAAAATATCTAGATTTTTTAAAGAAGTA
GACAAA---------------------------
>AMZ706 .
------------------------------TTAAGGAAAATTTCCCTGTGTTCTATTTTC
GTATTGGCATATCTCAGTTCTGCTAAGTGCGCGTACCGAGACGAATCTGACATATTGTAC
AGCGACGACTCCAGATCCGATTTAGCAAGCAACGGGGCACCCACCGACAGCTACGAATCT
TTAACAGCAAGTAGTGAGTCTCTAGCAGAAAGCAACGATGCACCCAGCAACAGCTATGAA
TCTTTTCCAGAAATTAGAGAAAATCTAACCGCAAGTGAGGAATCCCTAACATCATGTGAG
GAATCCCTAACAGGAAGTAATGAATCCCTAACAGGAAGTAATGAATCCCTAACAGGAAGT
AAT---------------------------------------------------------
---------------------------GAATCCCTAACAGGAAGTAATGAATCCCTAACA
GAAAGTAGAGAATCTCTAGAGGCCAGTAGAGAATCGCTAAGAGCAAGTAGAGAGTCTCTA
GCGGCCAGTAGAGAATCCCTGAACGACTTTTGTGGGAGCGAAGAATCAGTAGCATTCGAA
GGAGAGCCAAATGAAAAGACATTCATGGGAGACGTCTTAAGTGGTGGAGAATGTGAGAAT
AGTCTCTCAAGAGAAGATTTATTTCATATAGAAGTAGGATCCGAAGAATCGCTAGATGAT
GCCTCAAAATATAATTTCCAAAAGGATTTATCAACTAGCGATAATAGTTCATTCGAAGAT
GATCAGTCATTGAAAAGGGGACTCAAAAGGAACAGCTCAGTATCTAGTCTGGACAGCGAT
ATGGGAAGTTATAAAAATGGAAGATCTAGAGACAGATTGGATATATATTCGGACCTACCT
AGAAGGCCAATACATGGGGATAATGCACCACAAGAAAAGGAGAAATGTCTGGATTTCAAA
GAACTTCTAGAGAGAGAAAGGAACAACCACCAAAGGAATAGTGTAAATATAAAATTAAAA
AATATATACGGAGAAGAACAATTGTATAATGTGAAGAAAGATAGCTTTATTGTAAAATTG
ATATACTATCTGCCGTTTGGCCCAATTGTTACAATGATAATGATAGGCTTTATTCTACTC

```
TGCTCAACGCCCTCGACTGGTTTTATTTTTATAGGGTGTAGCTCTATTATATTACCAGCT
TTGTTATACGCGATGCATTCAAACAAAAAACAATTCGAACGTGTATTTGGAAAAGGGAGA
AAAAAGCCAATGAAGGTAAAAAAAGGAGGCGGGAAAATATCTAGATTTTTTAAAGAAGTA
GACAAA---------------------------
>AMZ732 .
------------------------------TTAAGGAAAATTTCCCTGTGTTCTATTTTC
GTATTGGCATATCTCAGTTCTGCTAAGTGCGCGTACCGAGACGAATCTGACATATTGTAC
AGCGACGACTCCAGATCCGATTTAGCAAGCAACGGGGCACCCACCGACAGCTACGAATCT
TTAACAGCAAGTAGTGAGTCTCTAGCAGAAAGCAACGATGCACCCAGCAACAGCTATGAA
TCTTTTCCAGAAATTAGAGAAAATCTAACCGCAAGTGAGGAATCCCTAACATCATGTGAG
GAATCCCTAACAGGAAGTAATGAATCCCTAACAGGAAGTAATGAATCCCTAACAGGAAGT
AAT---------------------------------------------------------
---------------------------GAATCCCTAACAGGAAGTAATGAATCCCTAACA
GAAAGTAGAGAATCTCTAGAGGCCAGTAGAGAATCGCTAAGAGCAAGTAGAGAGTCTCTA
GCGGCCAGTAGAGAATCCCTGAACGACTTTTGTGGGAGCGAAGAATCAGTAGCATTCGAA
GGAGAGCCAAATGAAAAGACATTCATGGGAGACGTCTTAAGTGGTGGAGAATGTGAGAAT
AGTCTCTCAAGAGAAGATTTATTTCATATAGAAGTAGGATCCGAAGAATCGCTAGATGAT
GCCTCAAAATATAATTTCCAAAAGGATTTATCAACTAGCGATAATAGTTCATTCGAAGAT
GATCAGTCATTGAAAAGGGGACTCAAAAGGAACAGCTCAGTATCTAGTCTGGACAGCGAT
ATGGGAAGTTATAAAAATGGAAGATCTAGAGACAGATTGGATATATATTCGGACCTACCT
AGAAGGCCAATACATGGGGATAATGCACCACAAGAAAAGGAGAAATGTCTGGATTTCAAA
GAACTTCTAGAGAGAGAAAGGAACAACCACCAAAGGAATAGTGTAAATATAAAATTAAAA
AATATATACGGAGAAGAACAATTGTATAATGTGAAGAAAGATAGCTTTATTGTAAAATTG
ATATACTATCTGCCGTTTGGCCCAATTGTTACAATGATAATGATAGGCTTTATTCTACTC
TGCTCAACGCCCTCGACTGGTTTTATTTTTATAGGGTGTAGCTCTATTATATTACCAGCT
TTGTTATACGCGATGCATTCAAACAAAAAACAATTCGAACGTGTATTTGGAAAAGGGAGA
AAAAAGCCAATGAAGGTAAAAAAAGGAGGCGGGAAAATATCTAGATTTTTTAAAGAAGTA
GACAAA---------------------------
>COD3_R .
------------------------------TTAAGGAAAATTTCCCTGTGTTCTATTTTC
GTATTGGCATATCTCAGTTCTGCTAAGTGCGCGTACCGAGACGAATCTGACATATTGTAC
AGCGACGACTCCAGATCCGATTTAGCAAGCAACGGGGCACCCACCGACAGCTACGAATCT
TTAACAGCAAGTAGTGAGTCTCTAGCAGAAAGCAACGATGCACCCAGCAACAGCTATGAA
TCTTTTCCAGAAATTAGAGAAAATCTAACCGCAAGTGAGGAATCCCTAACATCATGTGAG
GAATCCCTAACAGGAAGTAATGAATCCCTAACAGGAAGTAATGAATCCCTAACAGGAAGT
AAT---------------------------------------------------------
---------------------------GAATCCCTAACAGGAAGTAATGAATCCCTAACA
GAAAGTAGAGAATCTCTAGAGGCCAGTAGAGAATCGCTAAGAGCAAGTAGAGAGTCTCTA
GCGGCCAGTAGAGAATCCCTGAACGACTTTTGTGGGAGCGAAGAATCAGTAGCATTCGAA
GGAGAGCCAAATGAAAAGACATTCATGGGAGACGTCTTAAGTGGTGGAGAATGTGAGAAT
AGTCTCTCAAGAGAAGATTTATTTCATATAGAAGTAGGATCCGAAGAATCGCTAGATGAT
GCCTCAAAATATAATTTCCAAAAGGATTTATCAACTAGCGATAATAGTTTATTCGAAGAT
GATCAGTCATTGAAAAGGGGACTCAAAAGGAACAGCTCAGTATCTAGTCTGGACAGCGAT
ATGGGAAGTTATAAAAATGGAAGATCTAGAGACAGATTGGATATATATTCGGACCTACCT
AGAAGGCTAATACATGGGGATAATGCACCACAAGAAAAGGAGAAATGTCTGGATTTCAAA
GAACTTCTAGAGAGAGAAAGGAACAACCACCAAAGGAATAGTGTAAATATAAAATTAAAA
AATATATACGGAGAAGAACAATTGTATAATGTGAAGAAAGATAGCTTTATTGTAAAATTG
ATATACTATCTGCCGTTTGGCCCAATTGTTACAATGATAATGATAGGCTTTATTCTACTC
TGGTCAACGCCCTGGACTGGTTTTATTTTTATAGGGTGTAGCTCTATTATATTACCAGCT
TTGTTATACGCGATGCATTCAAACAAAAAACAATTCGAACGTGTATTTGGAAAAGGGAGA
```

AAAAAGCCAATGAAGGTAAAAAAAGGAGGCGGGAAAATATCTAGATTTTTTAAAGAAGTA
GACAAA---------------------------
>COD5 .
------------------------------TTAAGGAAAATTTCCCTGTGTTCTATTTTC
GTATTGGCATATCTCAGTTCTGCTAAGTGCGCGTACCGAGACGAATCTGACATATTGTAC
AGCGACGACTCCAGATCCGATTTAGCAAGCAACGGGGCACCCACCGACAGCTACGAATCT
TTAACAGCAAGTAGTGAGTCTCTAGCAGAAAGCAACGATGCACCCAGCAACAGCTATGAA
TCTTTTCCAGAAATTAGAGAAAATCTAACCGCAAGTGAGGAATCCCTAACATCATGTGAG
GAATCCCTAACAGGAAGTAATGAATCCCTAACAGGAAGTAATGAATCCCTAACAGGAAGT
AAT---------------------------------------------------------
------------------------------------------------GAATCCCTAACA
GAAAGTAGAGAATCTCTAGAGGCCAGTAGAGAATCGCTAAGAGCAAGTAGAGAGTCTCTA
GCGGCCAGTAGAGAATCCCTGAACGACTTTTGTGGGAGCGAAGAATCAGTAGCATTCGAA
GGAGAGCCAAATGAAAAGACATTCATGGGAGACGTCTTAAGTGGTGGAGAATGTGAGAAT
AGTCTCTCAAGAGAAGATTTATTTCATATAGAAGTAGGATCCGAAGAATCGCTAGATGAT
GCCTCAAAATATAATTTCCAAAAGGATTTATCAACTAGCGATAATAGTTTATTCGAAGAT
GATCAGTCATTGAAAAGGGGACTCAAAAGGAACAGCTCAGTATCTAGTCTGGACAGCGAT
ATGGGAAGTTATAAAAATGGAAGATCTAGAGACAGATTGGATATATATTCGGACCTACCT
AGAAGGCTAATACATGGGGATAATGCACCACAAGAAAAGGAGAAATGTCTGGATTTCAAA
GAACTTCTAGAGAGAGAAAGGAACAACCACCAAAGGAATAGTGTAAATATAAAATTAAAA
AATATATACGGAGAAGAACAATTGTATAATGTGAAGAAAGATAGCTTTATTGTAAAATTG
ATATACTATCTGCCGTTTGGCCCAATTGTTACAATGATAATGATAGGCTTTATTCTACTC
TGGTCAACGCCCTGGACTGGTTTTATTTTTATAGGGTGTAGCTCTATTATATTACCAGCT
TTGTTATACGCGATGCATTCAAACAAAAAACAATTCGAACGTGTATTTGGAAAAGGGAGA
AAAAAGCCAATGAAGGTAAAAAAAGGAGGCGGGAAAATATCTAGATTTTTTAAAGAAGTA
GACAAA---------------------------
>COD8 .
------------------------------TTAAGGAAAATTTCCCTGTGTTCTATTTTC
GTATTGGCATATCTCAGTTCTGCTAAGTGCGCGTACCGAGACGAATCTGACATATTGTAC
AGCGACGACTCCAGATCCGATTTAGCAAGCAACGGGGCACCCACCGACAGCTACGAATCT
TTAACAGCAAGTAGTGAGTCTCTAGCAGAAAGCAACGATGCACCCAGCAACAGCTATGAA
TCTTTTCCAGAAATTAGAGAAAATCTAACCGCAAGTGAGGAATCCCTAACATCATGTGAG
GAATCCCTAACAGGAAGTAACGAATCCCTAACAGGAAGTAATGAATCCCTAACAGGAAGT
AAT---------------------------------------------------------
---------------------------GAATCCCTAACAGGAAGTAATGAATCCCTAACA
GAAAGTAGAGAATCTCTAGAGGCCAGTAGAGAATCGCTAAGAGCAAGTAGAGAGTCTCTA
GCGGCCAGTAGAGAATCCCTGAACGACTTTTGTGGGAGCGAAGAATCAGTAGCATTCGAA
GGAGAGCCAAATGAAAAGACATTCATGGGAGACGTCTTAAGTGGTGGAGAATGTGAGAAT
AGTCTCTCAAGAGAAGATTTATTTCATATAGAAGTAGGATCCGAAGAATCGCTAGATGAT
GCCTCAAAATATAATTTCCAAAAGGATTTATCAACTAGCGATAATAGTTCATTCGAAGAT
GATCAGTCATTGAAAAGGGGACTCAAAAGGAACAGCTCAGTATCTAGTCTGGACAGCGAT
ATGGGAAGTTATAAAAATGGAAGATCTAGAGACAGATTGGATATATATTCGGACCTACCT
AGAAGGCTAATACATGGGGATAATGCACCACAAGAAAAGGAGAAATGTCTGGATTTCAAA
GAACTTCTAGAGAGAGAAAGGAACAACCACCAAAGGAATAGTGTAAATATAAAATTAAAA
AATATATACGGAGAAGAACAATTGTATAATGTGAAGAAAGATAGCTTTATTGTAAAATTG
ATATACTATCTGCCGTTTGGCCCAATTGTTACAATGATAATGATAGGTTTTTTTCTACTC

```
TGCTGGACCCCCTCGATTGGTTTTACTTTTATAGGGTGTAGCTCTATTATATTACCAGCT
TTGTTATACGCGATGCATTCAAACAAAAAACAATTCGAACGTGTATTTGGAAAAGGGAGA
AAAAAGCCAATGAAGGTAAAAAAAGGAGGCGGGAAAATATCTAGATTTTTTAAAGAAGTA
GACAAA---------------------------
>COD9 .
------------------------------TTAAGGAAAATTTCCCTGTGTTCTATTTTC
GTATTGGCATATCTCAGTTCTGCTAAGTGCGCGTACCGAGACGAATCTGACATATTGTAC
AGCGACGACTCCAGATCCGATTTAGCAAGCAACGGGGCACCCACCGACAGCTACGAATCT
TTAACAGCAAGTAGTGAGTCTCTAGCAGAAAGCAACGATGCACCCAGCAACAGCTATGAA
TCTTTTCCAGAAATTAGAGAAAATCTAACCGCAAGTGAGGAATCCCTAACATCATGTGAG
GAATCCCTAACAGGAAGTAATGAATCCCTAACAGGAAGTAATGAATCCCTAACAGGAAGT
AAT---------------------------------------------------------
------------------------------------------------GAATCCCTAACA
GAAAGTAGAGAATCTCTAGAGGCCAGTAGAGAATCGCTAAGAGCAAGTAGAGAGTCTCTA
GCGGCCAGTAGAGAATCCCTGAACGACTTTTGTGGGAGCGAAGAATCAGTAGCATTCGAA
GGAGAGCCAAATGAAAAGACATTCATGGGAGACGTCTTAAGTGGTGGAGAATGTGAGAAT
AGTCTCTCAAGAGAAGATTTATTTCATATAGAAGTAGGATCCGAAGAATCGCTAGATGAT
GCCTCAAAATATAATTTCCAAAAGGATTTATCAACTAGCGATAATAGTTTATTCGAAGAT
GATCAGTCATTGAAAAGGGGACTCAAAAGGAACAGCTCAGTATCTAGTCTGGACAGCGAT
ATGGGAAGTTATAAAAATGGAAGATCTAGAGACAGATTGGATATATATTCGGACCTACCT
AGAAGGCTAATACATGGGGATAATGCACCACAAGAAAAGGAGAAATGTCTGGATTTCAAA
GAACTTCTAGAGAGAGAAAGGAACAACCACCAAAGGAATAGTGTAAATATAAAATTAAAA
AATATATACGGAGAAGAACAATTGTATAATGTGAAGAAAGATAGCTTTATTGTAAAATTG
ATATACTATCTGCCGTTTGGCCCAATTGTTACAATGATAATTATAGGCTTTATTCTACTC
TGGTCAACGCCCTGGACTGGTTTTATTTTTATAGGGTGTAGCTCTATTATATTACCAGCT
TTGTTATACGCGATGCATTCAAACAAAAAACAATTCGAACGTGTATTTGGAAAAGGGAGA
AAAAAGCCAATGAAGGTAAAAAAAGGAGGCGGGAAAATATCTAGATTTTTTAAAGAAGTA
GACAAA---------------------------
>COINS5 .
------------------------------TTAAGGAAAATTTCCCTGTGTTCTATTTTC
GTATTGGCATATCTCAGTTCTGCTAAGTGCGCGTACCGAGACGAATCTGACATATTGTAC
AGCGACGACTCCAGATCCGATTTAGCAAGCAACGGGGCACCCACCGACAGCTACGAATCT
TTAACAGCAAGTAGTGAGTCTCTAGCAGAAAGCAACGATGCACCCAGCAACAGCTATGAA
TCTTTTCCAGAAATTAGAGAAAATCTAACCGCAAGTGAGGAATCCCTAACATCATGTGAG
GAATCCCTAACAGGAAGTAACGAATCCCTAACAGGAAGTAATGAATCCCTAACAGGAAGT
AAT---------------------------------------------------------
---------------------------GAATCCCTAACAGGAAGTAATGAATCCCTAACA
GAAAGTAGAGAATCTCTAGAGGCCAGTAGAGAATCGCTAAGAGCAAGTAGAGAGTCTCTA
GCGGCCAGTAGAGAATCCCTGAACGACTTTTGTGGGAGCGAAGAATCAGTAGCATTCGAA
GGAGAGCCAAATGAAAAGACATTCATGGGAGACGTCTTAAGTGGTGGAGAATGTGAGAAT
AGTCTCTCAAGAGAAGATTTATTTCATATAGAAGTAGGATCCGAAGAATCGCTAGATGAT
GCCTCAAAATATAATTTCCAAAAGGATTTATCAACTAGCGATAATAGTTCATTCGAAGAT
GATCAGTCATTGAAAAGGGGACTCAAAAGGAACAGCTCAGTATCTAGTCTGGACAGCGAT
ATGGGAAGTTATAAAAATGGAAGATCTAGAGACAGATTGGATATATATTCGGACCTACCT
AGAAGGCTAATACATGGGGATAATGCACCACAAGAAAAGGAGAAATGTCTGGATTTCAAA
GAACTTCTAGAGAGAGAAAGGAACAACCACCAAAGGAATAGTGTAAATATAAAATTAAAA
AATATATACGGAGAAGAACAATTGTATAATGTGAAGAAAGATAGCTTTATTGTAAAATTG
ATATACTATCTGCCGTTTGGCCCAATTGTTACAATGATAATGATAGGTTTTTTTCTACTC
TGCTGGACCCCCTCGATTGGTTTTACTTTTATAGGGTGTAGCTCTATTATATTACCAGCT
TTGTTATACGCGATGCATTCAAACAAAAAACAATTCGAACGTGTATTTGGAAAAGGGAGA
```

AAAAAGCCAATGAAGGTAAAAAAAGGAGGCGGGAAAATATCTAGATTTTTTAAAGAAGTA
GACAAA---------------------------
>COINS4 .
------------------------------TTAAGGAAAATTTCCCTGTGTTCTATTTTC
GTATTGGCATATCTCAGTTCTGCTAAGTGCGCGTACCGAGACGAATCTGACATATTGTAC
AGCGACGACTCCAGATCCGATTTAGCAAGCAACGGGGCACCCACCGACAGCTACGAATCT
TTAACAGCAAGTAGTGAGTCTCTAGCAGAAAGCAACGATGCACCCAGCAACAGCTATGAA
TCTTTTCCAGAAATTAGAGAAAATCTAACCGCAAGTGAGGAATCCCTAACATCATGTGAG
GAATCCCTAACAGGAAGTAATGAATCCCTAACAGGAAGTAATGAATCCCTAACAGGAAGT
AAT---------------------------------------------------------
---------------------------GAATCCCTAACAGGAAGTAATGAATCCCTAACA
GAAAGTAGAGAATCTCTAGAGGCCAGTAGAGAATCGCTAAGAGCAAGTAGAGAGTCTCTA
GCGGCCAGTAGAGAATCCCTGAACGACTTTTGTGGGAGCGAAGAATCAGTAGCATTCGAA
GGAGAGCCAAATGAAAAGACATTCATGGGAGACGTCTTAAGTGGTGGAGAATGTGAGAAT
AGTCTCTCAAGAGAAGATTTATTTCATATAGAAGTAGGATCCGAAGAATCGCTAGATGAT
GCCTCAAAATATAATTTCCAAAAGGATTTATCAACTAGCGATAATAGTTTATTCGAAGAT
GATCAGTCATTGAAAAGGGGACTCAAAAGGAACAGCTCAGTATCTAGTCTGGACAGCGAT
ATGGGAAGTTATAAAAATGGAAGATCTAGAGACAGATTGGATATATATTCGGACCTACCT
AGAAGGCTAATACATGGGGATAATGCACCACAAGAAAAGGAGAAATGTCTGGATTTCAAA
GAACTTCTAGAGAGAGAAAGGAACAACCACCAAAGGAATAGTGTAAATATAAAATTAAAA
AATATATACGGAGAAGAACAATTGTATAATGTGAAGAAAGATAGCTTTATTGTAAAATTG
ATATACTATCTGCCGTTTGGCCCAATTGTTACAATGATAATGATAGGCTTTATTCTACTC
TGGTCAACGCCCTGGACTGGTTTTATTTTTATAGGGTGTAGCTCTATTATATTACCAGCT
TTGTTATACGCGATGCATTCAAACAAAAAACAATTCGAACGTGTATTTGGAAAAGGGAGA
AAAAAGCCAATGAAGGTAAAAAAAGGAGGCGGGAAAATATCTAGATTTTTTAAAGAAGTA
GACAAA---------------------------
>COINS14 .
------------------------------TTAAGGAAAATTTCCCTGTGTTCTATTTTC
GTATTGGCATATCTCAGTTCTGCTAAGTGCGCGTACCGAGACGAATCTGACATATTGTAC
AGCGACGACTCCAGATCCGATTTAGCAAGCAACGGGGCACCCACCGACAGCTACGAATCT
TTAACAGCAAGTAGTGAGTCTCTAGCAGAAAGCAACGATGCACCCAGCAACAGCTATGAA
TCTTTTCCAGAAATTAGAGAAAATCTAACCGCAAGTGAGGAATCCCTAACATCATGTGAG
GAATCCCTAACAGGAAGTAATGAATCCCTAACAGGAAGTAATGAATCCCTAACAGGAAGT
AAT---------------------------------------------------------
------------------------------------------------GAATCCCTAACA
GAAAGTAGAGAATCTCTAGAGGCCAGTAGAGAATCGCTAAGAGCAAGTAGAGAGTCTCTA
GCGGCCAGTAGAGAATCCCTGAACGACTTTTGTGGGAGCGAAGAATCAGTAGCATTCGAA
GGAGAGCCAAATGAAAAGACATTCATGGGAGACGTCTTAAGTGGTGGAGAATGTGAGAAT
AGTCTCTCAAGAGAAGATTTATTTCATATAGAAGTAGGATCCGAAGAATCGCTAGATGAT
GCCTCAAAATATAATTTCCAAAAGGATTTATCAACTAGCGATAATAGTTTATTCGAAGAT
GATCAGTCATTGAAAAGGGGACTCAAAAGGAACAGCTCAGTATCTAGTCTGGACAGCGAT
ATGGGAAGTTATAAAAATGGAAGATCTAGAGACAGATTGGATATATATTCGGACCTACCT
AGAAGGCTAATACATGGGGATAATGCACCACAAGAAAAGGAGAAATGTCTGGATTTCAAA
GAACTTCTAGAGAGAGAAAGGAACAACCACCAAAGGAATAGTGTAAATATAAAATTAAAA
AATATATACGGAGAAGAACAATTGTATAATGTGAAGAAAGATAGCTTTATTGTAAAATTG
ATATACTATCTGCCGTTTGGCCCAATTGTTACAATGATAATTATAGGCTTTATTCTACTC

TGGTCAACGCCCTGGACTGGTTTTATTTTTATAGGGTGTAGCTCTATTATATTACCAGCT
TTGTTATACGCGATGCATTCAAACAAAAAACAATTCGAACGTGTATTTGGAAAAGGGAGA
AAAAAGCCAATGAAGGTAAAAAAAGGAGGCGGGAAAATATCTAGATTTTTTAAAGAAGTA
GACAAA---------------------------
>COINS20 .
------------------------------TTAAGGAAAATTTCCCTGTGTTCTATTTTC
GTATTGGCATATCTCAGTTCTGCTAAGTGCGCGTACCGAGACGAATCTGACATATTGTAC
AGCGACGACTCCAGATCCGATTTAGCAAGCAACGGGGCACCCACCGACAGCTACGAATCT
TTAACAGCAAGTAGTGAGTCTCTAGCAGAAAGCAACGATGCACCCAGCAACAGCTATGAA
TCTTTTCCAGAAATTAGAGAAAATCTAACCGCAAGTGAGGAATCCCTAACATCATGTGAG
GAATCCCTAACAGGAAGTAATGAATCCCTAACAGGAAGTAATGAATCCCTAACAGGAAGT
AAT---------------------------------------------------------
---------------------------GAATCCCTAACAGGAAGTAATGAATCCCTAACA
GAAAGTAGAGAATCTCTAGAGGCCAGTAGAGAATCGCTAAGAGCAAGTAGAGAGTCTCTA
GCGGCCAGTAGAGAATCCCTGAACGACTTTTGTGGGAGCGAAGAATCAGTAGCATTCGAA
GGAGAGCCAAATGAAAAGACATTCATGGGAGACGTCTTAAGTGGTGGAGAATGTGAGAAT
AGTCTCTCAAGAGAAGATTTATTTCATATAGAAGTAGGATCCGAAGAATCGCTAGATGAT
GCCTCAAAATATAATTTCCAAAAGGATTTATCAACTAGCGATAATAGTTCATTCGAAGAT
GATCAGTCATTGAAAAGGGGACTCAAAAGGAACAGCTCAGTATCTAGTCTGGACAGCGAT
ATGGGAAGTTATAAAAATAAAAGTTATAGACACAGATTGGATATATATTCGGACCTACCT
AGAAGGCCAATACATGGGGATAATGCACCACAAGAAAAGGAGAAATGTCTGGATTTCAAA
GAACTTCTAGAGAGAGAAAGGAACAACCACCAAAGGAATAGTGTAAATATAAAATTAAAA
AATATATACGGAGAAGAACAATTGTATAATGTGAAGAAAGATAGCTTTATTGTAAAATTG
ATATACTATCTGCCGTTTGGCCCAATTGTTACAATGATAATGATAGGCTTTATTCTACTC
TGCTCAACGCCCTCGACTGGTTTTATTTTTATAGGGTGTAGCTCTATTATATTACCAGCT
TTGTTATACGCGATGCATTCAAACAAAAAACAATTCGAACGTGTATTTGGAAAAGGGAGA
AAAAAGCCAATGAAGGTAAAAAAAGGAGGCGGGAAAATATCTAGATTTTTTAAAGAAGTA
GACAAA---------------------------
>COINS36 .
------------------------------TTAAGGAAAATTTCCCTGTGTTCTATTTTC
GTATTGGCATATCTCAGTTCTGCTAAGTGCGCGTACCGAGACGAATCTGACATATTGTAC
AGCGACGACTCCAGATCCGATTTAGCAAGCAACGGGGCACCCACCGACAGCTACGAATCT
TTAACAGCAAGTAGTGAGTCTCTAGCAGAAAGCAACGATGCACCCAGCAACAGCTATGAA
TCTTTTCCAGAAATTAGAGAAAATCTAACCGCAAGTGAGGAATCCCTAACATCATGTGAG
GAATCCCTAACAGGAAGTAACGAATCCCTAACAGGAAGTAATGAATCCCTAACAGGAAGT
AAT---------------------------------------------------------
---------------------------GAATCCCTAACAGGAAGTAATGAATCCCTAACA
GAAAGTAGAGAATCTCTAGAGGCCAGTAGAGAATCGCTAAGAGCAAGTAGAGAGTCTCTA
GCGGCCAGTAGAGAATCCCTGAACGACTTTTGTGGGAGCGAAGAATCAGTAGCATTCGAA
GGAGAGCCAAATGAAAAGACATTCATGGGAGACGTCTTAAGTGGTGGAGAATGTGAGAAT
AGTCTCTCAAGAGAAGATTTATTTCATATAGAAGTAGGATCCGAAGAATCGCTAGATGAT
GCCTCAAAATATAATTTCCAAAAGGATTTATCAACTAGCGATAATAGTTCATTCGAAGAT
GATCAGTCATTGAAAAGGGGACTCAAAAGGAACAGCTCAGTATCTAGTCTGGACAGCGAT
ATGGGAAGTTATAAAAATGGAAGATCTAGAGACAGATTGGATATATATTCGGACCTACCT
AGAAGGCTAATACATGGGGATAATGCACCACAAGAAAAGGAGAAATGTCTGGATTTCAAA
GAACTTCTAGAGAGAGAAAGGAACAACCACCAAAGGAATAGTGTAAATATAAAATTAAAA
AATATATACGGAGAAGAACAATTGTATAATGTGAAGAAAGATAGCTTTATTGTAAAATTG
ATATACTATCTGCCGTTTGGCCCAATTGTTACAATGATAATGATAGGCTTTATTCTACTC
TGCTCAACGCCCTCGACTGGTTTTATTTTTATAGGGTGTAGCTCTATTATATTACCAGCT
TTGTTATACGCGATGCATTCAAACAAAAAACAATTCGAACGTGTATTTGGAAAAGGGAGA

```
AAAAAGCCAATGAAGGTAAAAAAAGGAGGCGGGAAAATATCTAGATTTTTTAAAGAAGTA
GACAAA---------------------------
>COINS39 .
------------------------------TTAAGGAAAATTTCCCTGTGTTCTATTTTC
GTATTGGCATATCTCAGTTCTGCTAAGTGCGCGTACCGAGACGAATCTGACATATTGTAC
AGCGACGACTCCAGATCCGATTTAGCAAGCAACGGGGCACCCACCGACAGCTACGAATCT
TTAACAGCAAGTAGTGAGTCTCTAGCAGAAAGCAACGATGCACCCAGCAACAGCTATGAA
TCTTTTCCAGAAATTAGAGAAAATCTAACCGCAAGTGAGGAATCCCTAACATCATGTGAG
GAATCCCTAACAGGAAGTAATGAATCCCTAACAGGAAGTAATGAATCCCTAACAGGAAGT
AAT---------------------------------------------------------
------------------------------------------------GAATCCCTAACA
GAAAGTAGAGAATCTCTAGAGGCCAGTAGAGAATCGCTAAGAGCAAGTAGAGAGTCTCTA
GCGGCCAGTAGAGAATCCCTGAACGACTTTTGTGGGAGCGAAGAATCAGTAGCATTCGAA
GGAGAGCCAAATGAAAAGACATTCATGGGAGACGTCTTAAGTGGTGGAGAATGTGAGAAT
AGTCTCTCAAGAGAAGATTTATTTCATATAGAAGTAGGATCCGAAGAATCGCTAGATGAT
GCCTCAAAATATAATTTCCAAAAGGATTTATCAACTAGCGATAATAGTTTATTCGAAGAT
GATCAGTCATTGAAAAGGGGACTCAAAAGGAACAGCTCAGTATCTAGTCTGGACAGCGAT
ATGGGAAGTTATAAAAATGGAAGATCTAGAGACAGATTGGATATATATTCGGACCTACCT
AGAAGGCTAATACATGGGGATAATGCACCACAAGAAAAGGAGAAATGTCTGGATTTCAAA
GAACTTCTAGAGAGAGAAAGGAACAACCACCAAAGGAATAGTGTAAATATAAAATTAAAA
AATATATACGGAGAAGAACAATTGTATAATGTGAAGAAAGATAGCTTTATTGTAAAATTG
ATATACTATCTGCCGTTTGGCCCAATTGTTACAATGATAATTATAGGCTTTATTCTACTC
TGGTCAACGCCCTGGACTGGTTTTATTTTTATAGGGTGTAGCTCTATTATATTACCAGCT
TTGTTATACGCGATGCATTCAAACAAAAAACAATTCGAACGTGTATTTGGAAAAGGGAGA
AAAAAGCCAATGAAGGTAAAAAAAGGAGGCGGGAAAATATCTAGATTTTTTAAAGAAGTA
GACAAA---------------------------
>COINS42 .
------------------------------TTAAGGAAAATTTCCCTGTGTTCTATTTTC
GTATTGGCATATCTCAGTTCTGCTAAGTGCGCGTACCGAGACGAATCTGACATATTGTAC
AGCGACGACTCCAGATCCGATTTAGCAAGCAACGGGGCACCCACCGACAGCTACGAATCT
TTAACAGCAAGTAGTGAGTCTCTAGCAGAAAGCAACGATGCACCCAGCAACAGCTATGAA
TCTTTTCCAGAAATTAGAGAAAATCTAACCGCAAGTGAGGAATCCCTAACATCATGTGAG
GAATCCCTAACAGGAAGTAACGAATCCCTAACAGGAAGTAATGAATCCCTAACAGGAAGT
AAT---------------------------------------------------------
---------------------------GAATCCCTAACAGGAAGTAATGAATCCCTAACA
GAAAGTAGAGAATCTCTAGAGGCCAGTAGAGAATCGCTAAGAGCAAGTAGAGAGTCTCTA
GCGGCCAGTAGAGAATCCCTGAACGACTTTTGTGGGAGCGAAGAATCAGTAGCATTCGAA
GGAGAGCCAAATGAAAAGACATTCATGGGAGACGTCTTAAGTGGTGGAGAATGTGAGAAT
AGTCTCTCAAGAGAAGATTTATTTCATATAGAAGTAGGATCCGAAGAATCGCTAGATGAT
GCCTCAAAATATAATTTCCAAAAGGATTTATCAACTAGCGATAATAGTTCATTCGAAGAT
GATCAGTCATTGAAAAGGGGACTCAAAAGGAACAGCTCAGTATCTAGTCTGGACAGCGAT
ATGGGAAGTTATAAAAATGGAAGATCTAGAGACAGATTGGATATATATTCGGACCTACCT
AGAAGGCTAATACATGGGGATAATGCACCACAAGAAAAGGAGAAATGTCTGGATTTCAAA
GAACTTCTAGAGAGAGAAAGGAACAACCACCAAAGGAATAGTGTAAATATAAAATTAAAA
AATATATACGGAGAAGAACAATTGTATAATGTGAAGAAAGATAGCTTTATTGTAAAATTG
ATATACTATCTGCCGTTTGGCCCAATTGTTACAATGATAATGATAGGTTTTTTTCTACTC
```

TGCTCAACCCCCTCGATTGGTTTTATTTTTATAGGGTGTAGCTCTATTATATTACCAGCT
TTGTTATACGCGATGCATTCAAACAAAAAACAATTCGAACGTGTATTTGGAAAAGGGAGA
AAAAAGCCAATGAAGGTAAAAAAAGGAGGCGGGAAAATATCTAGATTTTTTAAAGAAGTA
GACAAA---------------------------
>COP02914 .
------------------------------TTAAGGAAAATTTCCCTGTGTTCTATTTTC
GTATTGGCATATCTCAGTTCTGCTAAGTGCGCGTACCGAGACGAATCTGACATATTGTAC
AGCGACGACTCCAGATCCGATTTAGCAAGCAACGGGGCACCCACCGACAGCTACGAATCT
TTAACAGCAAGTAGTGAGTCTCTAGCAGAAAGCAACGATGCACCCAGCAACAGCTATGAA
TCTTTTCCAGAAATTAGAGAAAATCTAACCGCAAGTGAGGAATCCCTAACATCATGTGAG
GAATCCCTAACAGGAAGTAATGAATCCCTAACAGGAAGTAATGAATCCCTAACAGGAAGT
AAT---------------------------------------------------------
------------------------------------------------GAATCCCTAACA
GAAAGTAGAGAATCTCTAGAGGCCAGTAGAGAATCGCTAAGAGCAAGTAGAGAGTCTCTA
GCGGCCAGTAGAGAATCCCTGAACGACTTTTGTGGGAGCGAAGAATCAGTAGCATGCGAA
GGAGAGCCAAATGAAAAGACATTCATGGGAGACGTCTTAAGTGGTGGAGAATGTGAGAAT
AGTCTCTCAAGAGAAGATTTATTTCATATAGAAGTAGGATCCGAAGAATCGCTAGATGAT
GCCTCAAAATATAATTTCCAAAAGGATTTATCAACTAGCGATAATAGTTTATTCGAAGAT
GATCAGTCATTGAAAAGGGGACTCAAAAGGAACAGCTCAGTATCTAGTCTGGACAGCGAT
ATGGGAAGTTATAAAAATGGAAGATCTAGAGACAGATTGGATATATATTCGGACCTACCT
AGAAGGCTAATACATGGGGATAATGCACCACAAGAAAAGGAGAAATGTCTGGATTTCAAA
GAACTTCTAGAGAGAGAAAGGAACAACCACCAAAGGAATAGTGTAAATATAAAATTAAAA
AATATATACGGAGAAGAACAATTGTATAATGTGAAGAAAGATAGCTTTATTGTAAAATTG
ATATACTATCTGCCGTTTGGCCCAATTGTTACAATGATAATTATAGGCTTTATTCTACTC
TGGTCAACGACTTGGACTGGTTTTATTTTTATAGGGTGTAGCTCTATTATATTACCAGCT
TTGTTATACGCGATGCATTCAAACAAAAAACAATTCGAACGTGTATTTGGAAAAGGGAGA
AAAAAGCCAATGAAGGTAAAAAAAGGAGGCGGGAAAATATCTAGATTTTTTAAAGAAGTA
GACAAA---------------------------
>COP03114 .
------------------------------TTAAGGAAAATTTCCCTGTGTTCTATTTTC
GTATTGGCATATCTCAGTTCTGCTAAGTGCGCGTACCGAGACGAATCTGACATATTGTAC
AGCGACGACTCCAGATCCGATTTAGCAAGCAACGGGGCACCCACCGACAGCTACGAATCT
TTAACAGCAAGTAGTGAGTCTCTAGCAGAAAGCAACGATGCACCCAGCAACAGCTATGAA
TCTTTTCCAGAAATTAGAGAAAATCTAACCGCAAGTGAGGAATCCCTAACATCATGTGAG
GAATCCCTAACAGGAAGTAATGAATCCCTAACAGGAAGTAATGAATCCCTAACAGGAAGT
AAT---------------------------------------------------------
------------------------------------------------GAATCCCTAACA
GAAAGTAGAGAATCTCTAGAGGCCAGTAGAGAATCGCTAAGAGCAAGTAGAGAGTCTCTA
GCGGCCAGTAGAGAATCCCTGAACGACTTTTGTGGGAGCGAAGAATCAGTAGCATGCGAA
GGAGAGCCAAATGAAAAGACATTCATGGGAGACGTCTTAAGTGGTGGAGAATGTGAGAAT
AGTCTCTCAAGAGAAGATTTATTTCATATAGAAGTAGGATCCGAAGAATCGCTAGATGAT
GCCTCAAAATATAATTTCCAAAAGGATTTATCAACTAGCGATAATAGTTTATTCGAAGAT
GATCAGTCATTGAAAAGGGGACTCAAAAGGAACAGCTCAGTATCTAGTCTGGACAGCGAT
ATGGGAAGTTATAAAAATGGAAGATCTAGAGACAGATTGGATATATATTCGGACCTACCT
AGAAGGCTAATACATGGGGATAATGCACCACAAGAAAAGGAGAAATGTCTGGATTTCAAA
GAACTTCTAGAGAGAGAAAGGAACAACCACCAAAGGAATAGTGTAAATATAAAATTAAAA
AATATATACGGAGAAGAACAATTGTATAATGTGAAGAAAGATAGCTTTATTGTAAAATTG
ATATACTATCTGCCGTTTGGCCCAATTGTTACAATGATAATTATAGGCTTTATTCTACTC
TGGTCAACGACTTGGACTGGTTTTATTTTTATAGGGTGTAGCTCTATTATATTACCAGCT
TTGTTATACGCGATGCATTCAAACAAAAAACAATTCGAACGTGTATTTGGAAAAGGGAGA

AAAAAGCCAATGAAGGTAAAAAAAGGAGGCGGGAAAATATCTAGATTTTTTAAAGAAGTA
GACAAA---------------------------
>COP03214 .
------------------------------TTAAGGAAAATTTCCCTGTGTTCTATTTTC
GTATTGGCATATCTCAGTTCTGCTAAGTGCGCGTACCGAGACGAATCTGACATATTGTAC
AGCGACGACTCCAGATCCGATTTAGCAAGCAACGGGGCACCCACCGACAGCTACGAATCT
TTAACAGCAAGTAGTGAGTCTCTAGCAGAAAGCAACGATGCACCCAGCAACAGCTATGAA
TCTTTTCCAGAAATTAGAGAAAATCTAACCGCAAGTGAGGAATCCCTAACATCATGTGAG
GAATCCCTAACAGGAAGTAATGAATCCCTAACAGGAAGTAATGAATCCCTAACAGGAAGT
AAT---------------------------------------------------------
---------------------------GAATCCCTAACAGGAAGTAATGAATCCCTAACA
GAAAGTAGAGAATCTCTAGAGGCCAGTAGAGAATCGCTAAGAGCAAGTAGAGAGTCTCTA
GCGGCCAGTAGAGAATCCCTGAACGACTTTTGTGGGAGCGAAGAATCAGTAGCATTCGAA
GGAGAGCCAAATGAAAAGACATTCATGGGAGACGTCTTAAGTGGTGGAGAATGTGAGAAT
AGTCTCTCAAGAGAAGATTTATTTCATATAGAAGTAGGATCCGAAGAATCGCTAGATGAT
GCCTCAAAATATAATTTCCAAAAGGATTTATCAACTAGCGATAATAGTTCATTCGAAGAT
GATCAGTCATTGAAAAGGGGACTCAAAAGGAACAGCTCAGTATCTAGTCTGGACAGCGAT
ATGGGAAGTTATAAAAATAAAAGTTATAGACACAGATTGGATATATATTCGGACCTACCT
AGAAGGCCAATACATGGGGATAATGCACCACAAGAAAAGGAGAAATGTCTGGATTTCAAA
GAACTTCTAGAGAGAGAAAGGAACAACCACCAAAGGAATAGTGTAAATATAAAATTAAAA
AATATATACGGAGAAGAACAATTGTATAATGTGAAGAAAGATAGCTTTATTGTAAAATTG
ATATACTATCTGCCGTTTGGCCCAATTGTTACAATGATAATGATAGGCTTTATTCTACTC
TGGTCAACGCCCTGGACTGGTTTTATTTTTATAGGGTGTAGCTCTATTATATTACCAGCT
TTGTTATACGCGATGCATTCAAACAAAAAACAATTCGAACGTGTATTTGGAAAAGGGAGA
AAAAAGCCAATGAAGGTAAAAAAAGGAGGCGGGAAAATATCTAGATTTTTTAAAGAAGTA
GACAAA---------------------------
>COP03514 .
------------------------------TTAAGGAAAATTTCCCTGTGTTCTATTTTC
GTATTGGCATATCTCAGTTCTGCTAAGTGCGCGTACCGAGACGAATCTGACATATTGTAC
AGCGACGACTCCAGATCCGATTTAGCAAGCAACGGGGCACCCACCGACAGCTACGAATCT
TTAACAGCAAGTAGTGAGTCTCTAGCAGAAAGCAACGATGCACCCAGCAACAGCTATGAA
TCTTTTCCAGAAATTAGAGAAAATCTAACCGCAAGTGAGGAATCCCTAACATCATGTGAG
GAATCCCTAACAGGAAGTAATGAATCCCTAACAGGAAGTAATGAATCCCTAACAGGAAGT
AAT---------------------GAATCCCTAACAGGAAGTAATGAATCCCTAACAGGA
AGTAATGAATCCCTAACAGGAAGTAATGAATCCCTAACAGGAAGTAATGAATCCCTAACA
GAAAGTAGAGAATCTCTAGAGGCCAGTAGAGAATCGCTAAGAGCAAGTAGAGAGTCTCTA
GCGGCCAGTAGAGAATCCCTGAACGACTTTTGTGGGAGCGAAGAATCAGTAGCATTCGAA
GGAGAGCCAAATGAAAAGACATTCATGGGAGACGTCTTAAGTGGTGGAGAATGTGAGAAT
AGTCTCTCAAGAGAAGATTTATTTCATATAGAAGTAGGATCCGAAGAATCGCTAGATGAT
GCCTCAAAATATAATTTCCAAAAGGATTTATCAACTAGCGATAATAGTTTATTCGAAGAT
GATCAGTCATTGAAAAGGGGACTCAAAAGGAACAGCTCAGTATCTAGTCTGGACAGCGAT
ATGGGAAGTTATAAAAATGGAAGATCTAGAGACAGATTGGATATATATTCGGACCTACCT
AGAAGGCTAATACATGGGGATAATGCACCACAAGAAAAGGAGAAATGTCTGGATTTCAAA
GAACTTCTAGAGAGAGAAAGGAACAACCACCAAAGGAATAGTGTAAATATAAAATTAAAA
AATATATACGGAGAAGAACAATTGTATAATGTGAAGAAAGATAGCTTTATTGTAAAATTG
ATATACTATCTGCCGTTTGGCCCAATTGTTACAATGATAATGATAGGTTTTTTTCTACTC

```
TGCTGGACCCCCTCGATTGGTTTTACTTTTATAGGGTGTAGCTCTATTATATTACCAGCT
TTGTTATACGCGATGCATTCAAACAAAAAACAATTCGAACGTGTATTTGGAAAAGGGAGA
AAAAAGCCAATGAAGGTAAAAAAAGGAGGCGGGAAAATATCTAGATTTTTTAAAGAAGTA
GACAAA---------------------------
>COP03714 .
------------------------------TTAAGGAAAATTTCCCTGTGTTCTATTTTC
GTATTGGCATATCTCAGTTCTGCTAAGTGCGCGTACCGAGACGAATCTGACATATTGTAC
AGCGACGACTCCAGATCCGATTTAGCAAGCAACGGGGCACCCACCGACAGCTACGAATCT
TTAACAGCAAGTAGTGAGTCTCTAGCAGAAAGCAACGATGCACCCAGCAACAGCTATGAA
TCTTTTCCAGAAATTAGAGAAAATCTAACCGCAAGTGAGGAATCCCTAACA---------
------------GGAAGTAATGAATCCCTAACAGGAAGTAATGAATCCCTAACAGAAAGT
AAT------------------------------------------GAATCCCTAACAGGA
AGTAATGAATCCCTAACAGGAAGTAATGAATCCCTAACAGGAAGTAATGAATCCCTAACA
GAAAGTAGAGAATCTCTAGAGGCCAGTAGAGAATCGCTAAGAGCAAGTAGAGAGTCTCTA
GCGGCCAGTAGAGAATCCCTGAACGACTTTTGTGGGAGCGAAGAATCAGTAGCATTCGAA
GGAGAGCCAAATGAAAAGACATTCATGGGAGACGTCTTAAGTGGTGGAGAATGTGAGAAT
AGTCTCTCAAGAGAAGATTTATTTCATATAGAAGTAGGATCCGAAGAATCGCTAGATGAT
GCCTCAAAATATAATTTCCAAAAGGATTTATCAACTAGCGATAATAGTTTATTCGAAGAT
GATCAGTCATTGAAAAGGGGACTCAAAAGGAACAGCTCAGTATCTAGTCTGGACAGCGAT
ATGGGAAGTTATAAAAATGGAAGATCTAGAGACAGATTGGATATATATTCGGACCTACCT
AGAAGGCTAATACATGGGGATAATGCACCACAAGAAAAGGAGAAATGTCTGGATTTCAAA
GAACTTCTAGAGAGAGAAAGGAACAACCACCAAAGGAATAGTGTAAATATAAAATTAAAA
AATATATACGGAGAAGAACAATTGTATAATGTGAAGAAAGATAGCTTTATTGTAAAATTG
ATATACTATCTGCCGTTTGGCCCAATTGTTACAATGATAATGATAGGTTTTTTTCTACTC
TGCTGGACCCCCTCGATTGGTTTTATTTTTATAGGGTGTAGCTCTATTATATTACCAGCT
TTGTTATACGCGATGCATTCAAACAAAAAACAATTCGAACGTGTATTTGGAAAAGGGAGA
AAAAAGCCAATGAAGGTAAAAAAAGGAGGCGGGAAAATATCTAGATTTTTTAAAGAAGTA
GACAAA---------------------------
>COP04114 .
------------------------------TTAAGGAAAATTTCCCTGTGTTCTATTTTC
GTATTGGCATATCTCAGTTCTGCTAAGTGCGCGTACCGAGACGAATCTGACATATTGTAC
AGCGACGACTCCAGATCCGATTTAGCAAGCAACGGGGCACCCACCGACAGCTACGAATCT
TTAACAGCAAGTAGTGAGTCTCTAGCAGAAAGCAACGATGCACCCAGCAACAGCTATGAA
TCTTTTCCAGAAATTAGAGAAAATCTAACCGCAAGTGAGGAATCCCTAACATCATGTGAG
GAATCCCTAACAGGAAGTAATGAATCCCTAACAGGAAGTAATGAATCCCTAACAGGAAGT
AAT---------------------------------------------------------
------------------------------------------------GAATCCCTAACA
GAAAGTAGAGAATCTCTAGAGGCCAGTAGAGAATCGCTAAGAGCAAGTAGAGAGTCTCTA
GCGGCCAGTAGAGAATCCCTGAACGACTTTTGTGGGAGCGAAGAATCAGTAGCATGCGAA
GGAGAGCCAAATGAAAAGACATTCATGGGAGACGTCTTAAGTGGTGGAGAATGTGAGAAT
AGTCTCTCAAGAGAAGATTTATTTCATATAGAAGTAGGATCCGAAGAATCGCTAGATGAT
GCCTCAAAATATAATTTCCAAAAGGATTTATCAACTAGCGATAATAGTTTATTCGAAGAT
GATCAGTCATTGAAAAGGGGACTCAAAAGGAACAGCTCAGTATCTAGTCTGGACAGCGAT
ATGGGAAGTTATAAAAATGGAAGATCTAGAGACAGATTGGATATATATTCGGACCTACCT
AGAAGGCTAATACATGGGGATAATGCACCACAAGAAAAGGAGAAATGTCTGGATTTCAAA
GAACTTCTAGAGAGAGAAAGGAACAACCACCAAAGGAATAGTGTAAATATAAAATTAAAA
AATATATACGGAGAAGAACAATTGTATAATGTGAAGAAAGATAGCTTTATTGTAAAATTG
ATATACTATCTGCCGTTTGGCCCAATTGTTACAATGATAATTATAGGCTTTATTCTACTC
TGGTCAACGACTTGGACTGGTTTTATTTTTATAGGGTGTAGCTCTATTATATTACCAGCT
TTGTTATACGCGATGCATTCAAACAAAAAACAATTCGAACGTGTATTTGGAAAAGGGAGA
```

AAAAAGCCAATGAAGGTAAAAAAAGGAGGCGGGAAAATATCTAGATTTTTTAAAGAAGTA
GACAAA---------------------------
>COP04414 .
------------------------------TTAAGGAAAATTTCCCTGTGTTCTATTTTC
GTATTGGCATATCTCAGTTCTGCTAAGTGCGCGTACCGAGACGAATCTGACATATTGTAC
AGCGACGACTCCAGATCCGATTTAGCAAGCAACGGGGCACCCACCGACAGCTACGAATCT
TTAACAGCAAGTAGTGAGTCTCTAGCAGAAAGCAACGATGCACCCAGCAACAGCTATGAA
TCTTTTCCAGAAATTAGAGAAAATCTAACCGCAAGTGAGGAATCCCTAACATCATGTGAG
GAATCCCTAACAGGAAGTAATGAATCCCTAACAGGAAGTAATGAATCCCTAACAGGAAGT
AAT---------------------------------------------------------
------------------------------------------------GAATCCCTAACA
GAAAGTAGAGAATCTCTAGAGGCCAGTAGAGAATCGCTAAGAGCAAGTAGAGAGTCTCTA
GCGGCCAGTAGAGAATCCCTGAACGACTTTTGTGGGAGCGAAGAATCAGTAGCATGCGAA
GGAGAGCCAAATGAAAAGACATTCATGGGAGACGTCTTAAGTGGTGGAGAATGTGAGAAT
AGTCTCTCAAGAGAAGATTTATTTCATATAGAAGTAGGATCCGAAGAATCGCTAGATGAT
GCCTCAAAATATAATTTCCAAAAGGATTTATCAACTAGCGATAATAGTTTATTCGAAGAT
GATCAGTCATTGAAAAGGGGACTCAAAAGGAACAGCTCAGTATCTAGTCTGGACAGCGAT
ATGGGAAGTTATAAAAATGGAAGATCTAGAGACAGATTGGATATATATTCGGACCTACCT
AGAAGGCTAATACATGGGGATAATGCACCACAAGAAAAGGAGAAATGTCTGGATTTCAAA
GAACTTCTAGAGAGAGAAAGGAACAACCACCAAAGGAATAGTGTAAATATAAAATTAAAA
AATATATACGGAGAAGAACAATTGTATAATGTGAAGAAAGATAGCTTTATTGTAAAATTG
ATATACTATCTGCCGTTTGGCCCAATTGTTACAATGATAATTATAGGCTTTATTCTACTC
TGGTCAACGACTTGGACTGGTTTTATTTTTATAGGGTGTAGCTCTATTATATTACCAGCT
TTGTTATACGCGATGCATTCAAACAAAAAACAATTCGAACGTGTATTTGGAAAAGGGAGA
AAAAAGCCAATGAAGGTAAAAAAAGGAGGCGGGAAAATATCTAGATTTTTTAAAGAAGTA
GACAAA---------------------------
>COP04514 .
------------------------------TTAAGGAAAATTTCCCTGTGTTCTATTTTC
GTATTGGCATATCTCAGTTCTGCTAAGTGCGCGTACCGAGACGAATCTGACATATTGTAC
AGCGACGACTCCAGATCCGATTTAGCAAGCAACGGGGCACCCACCGACAGCTACGAATCT
TTAACAGCAAGTAGTGAGTCTCTAGCAGAAAGCAACGATGCACCCAGCAACAGCTATGAA
TCTTTTCCAGAAATTAGAGAAAATCTAACCGCAAGTGAGGAATCCCTAACATCATGTGAG
GAATCCCTAACAGGAAGTAATGAATCCCTAACAGGAAGTAATGAATCCCTAACAGGAAGT
AAT---------------------------------------------------------
------------------------------------------------GAATCCCTAACA
GAAAGTAGAGAATCTCTAGAGGCCAGTAGAGAATCGCTAAGAGCAAGTAGAGAGTCTCTA
GCGGCCAGTAGAGAATCCCTGAACGACTTTTGTGGGAGCGAAGAATCAGTAGCATGCGAA
GGAGAGCCAAATGAAAAGACATTCATGGGAGACGTCTTAAGTGGTGGAGAATGTGAGAAT
AGTCTCTCAAGAGAAGATTTATTTCATATAGAAGTAGGATCCGAAGAATCGCTAGATGAT
GCCTCAAAATATAATTTCCAAAAGGATTTATCAACTAGCGATAATAGTTTATTCGAAGAT
GATCAGTCATTGAAAAGGGGACTCAAAAGGAACAGCTCAGTATCTAGTCTGGACAGCGAT
ATGGGAAGTTATAAAAATGGAAGATCTAGAGACAGATTGGATATATATTCGGACCTACCT
AGAAGGCTAATACATGGGGATAATGCACCACAAGAAAAGGAGAAATGTCTGGATTTCAAA
GAACTTCTAGAGAGAGAAAGGAACAACCACCAAAGGAATAGTGTAAATATAAAATTAAAA
AATATATACGGAGAAGAACAATTGTATAATGTGAAGAAAGATAGCTTTATTGTAAAATTG
ATATACTATCTGCCGTTTGGCCCAATTGTTACAATGATAATTATAGGCTTTATTCTACTC

TGGTCAACGACTTGGACTGGTTTTATTTTTATAGGGTGTAGCTCTATTATATTACCAGCT
TTGTTATACGCGATGCATTCAAACAAAAAACAATTCGAACGTGTATTTGGAAAAGGGAGA
AAAAAGCCAATGAAGGTAAAAAAAGGAGGCGGGAAAATATCTAGATTTTTTAAAGAAGTA
GACAAA---------------------------
>COP04614 .
------------------------------TTAAGGAAAATTTCCCTGTGTTCTATTTTC
GTATTGGCATATCTCAGTTCTGCTAAGTGCGCGTACCGAGACGAATCTGACATATTGTAC
AGCGACGACTCCAGATCCGATTTAGCAAGCAACGGGGCACCCACCGACAGCTACGAATCT
TTAACAGCAAGTAGTGAGTCTCTAGCAGAAAGCAACGATGCACCCAGCAACAGCTATGAA
TCTTTTCCAGAAATTAGAGAAAATCTAACCGCAAGTGAGGAATCCCTAACATCATGTGAG
GAATCCCTAACAGGAAGTAATGAATCCCTAACAGGAAGTAATGAATCCCTAACAGGAAGT
AAT---------------------------------------------------------
------------------------------------------------GAATCCCTAACA
GAAAGTAGAGAATCTCTAGAGGCCAGTAGAGAATCGCTAAGAGCAAGTAGAGAGTCTCTA
GCGGCCAGTAGAGAATCCCTGAACGACTTTTGTGGGAGCGAAGAATCAGTAGCATGCGAA
GGAGAGCCAAATGAAAAGACATTCATGGGAGACGTCTTAAGTGGTGGAGAATGTGAGAAT
AGTCTCTCAAGAGAAGATTTATTTCATATAGAAGTAGGATCCGAAGAATCGCTAGATGAT
GCCTCAAAATATAATTTCCAAAAGGATTTATCAACTAGCGATAATAGTTTATTCGAAGAT
GATCAGTCATTGAAAAGGGGACTCAAAAGGAACAGCTCAGTATCTAGTCTGGACAGCGAT
ATGGGAAGTTATAAAAATGGAAGATCTAGAGACAGATTGGATATATATTCGGACCTACCT
AGAAGGCTAATACATGGGGATAATGCACCACAAGAAAAGGAGAAATGTCTGGATTTCAAA
GAACTTCTAGAGAGAGAAAGGAACAACCACCAAAGGAATAGTGTAAATATAAAATTAAAA
AATATATACGGAGAAGAACAATTGTATAATGTGAAGAAAGATAGCTTTATTGTAAAATTG
ATATACTATCTGCCGTTTGGCCCAATTGTTACAATGATAATTATAGGCTTTATTCTACTC
TGGTCAACGACTTGGACTGGTTTTATTTTTATAGGGTGTAGCTCTATTATATTACCAGCT
TTGTTATACGCGATGCATTCAAACAAAAAACAATTCGAACGTGTATTTGGAAAAGGGAGA
AAAAAGCCAATGAAGGTAAAAAAAGGAGGCGGGAAAATATCTAGATTTTTTAAAGAAGTA
GACAAA---------------------------
>COP04814 .
------------------------------TTAAGGAAAATTTCCCTGTGTTCTATTTTC
GTATTGGCATATCTCAGTTCTGCTAAGTGCGCGTACCGAGACGAATCTGACATATTGTAC
AGCGACGACTCCAGATCCGATTTAGCAAGCAACGGGGCACCCACCGACAGCTACGAATCT
TTAACAGCAAGTAGTGAGTCTCTAGCAGAAAGCAACGATGCACCCAGCAACAGCTATGAA
TCTTTTCCAGAAATTAGAGAAAATCTAACCGCAAGTGAGGAATCCCTAACATCATGTGAG
GAATCCCTAACAGGAAGTAATGAATCCCTAACAGGAAGTAATGAATCCCTAACAGGAAGT
AAT---------------------------------------------------------
------------------------------------------------GAATCCCTAACA
GAAAGTAGAGAATCTCTAGAGGCCAGTAGAGAATCGCTAAGAGCAAGTAGAGAGTCTCTA
GCGGCCAGTAGAGAATCCCTGAACGACTTTTGTGGGAGCGAAGAATCAGTAGCATGCGAA
GGAGAGCCAAATGAAAAGACATTCATGGGAGACGTCTTAAGTGGTGGAGAATGTGAGAAT
AGTCTCTCAAGAGAAGATTTATTTCATATAGAAGTAGGATCCGAAGAATCGCTAGATGAT
GCCTCAAAATATAATTTCCAAAAGGATTTATCAACTAGCGATAATAGTTTATTCGAAGAT
GATCAGTCATTGAAAAGGGGACTCAAAAGGAACAGCTCAGTATCTAGTCTGGACAGCGAT
ATGGGAAGTTATAAAAATGGAAGATCTAGAGACAGATTGGATATATATTCGGACCTACCT
AGAAGGCTAATACATGGGGATAATGCACCACAAGAAAAGGAGAAATGTCTGGATTTCAAA
GAACTTCTAGAGAGAGAAAGGAACAACCACCAAAGGAATAGTGTAAATATAAAATTAAAA
AATATATACGGAGAAGAACAATTGTATAATGTGAAGAAAGATAGCTTTATTGTAAAATTG
ATATACTATCTGCCGTTTGGCCCAATTGTTACAATGATAATTATAGGCTTTATTCTACTC
TGGTCAACGACTTGGACTGGTTTTATTTTTATAGGGTGTAGCTCTATTATATTACCAGCT
TTGTTATACGCGATGCATTCAAACAAAAAACAATTCGAACGTGTATTTGGAAAAGGGAGA

AAAAAGCCAATGAAGGTAAAAAAAGGAGGCGGGAAAATATCTAGATTTTTTAAAGAAGTA
GACAAA---------------------------
>COP04914 .
------------------------------TTAAGGAAAATTTCCCTGTGTTCTATTTTC
GTATTGGCATATCTCAGTTCTGCTAAGTGCGCGTACCGAGACGAATCTGACATATTGTAC
AGCGACGACTCCAGATCCGATTTAGCAAGCAACGGGGCACCCACCGACAGCTACGAATCT
TTAACAGCAAGTAGTGAGTCTCTAGCAGAAAGCAACGATGCACCCAGCAACAGCTATGAA
TCTTTTCCAGAAATTAGAGAAAATCTAACCGCAAGTGAGGAATCCCTAACATCATGTGAG
GAATCCCTAACAGGAAGTAACGAATCCCTAACAGGAAGTAATGAATCCCTAACAGGAAGT
AAT---------------------------------------------------------
---------------------------GAATCCCTAACAGGAAGTAATGAATCCCTAACA
GAAAGTAGAGAATCTCTAGAGGCCAGTAGAGAATCGCTAAGAGCAAGTAGAGAGTCTCTA
GCGGCCAGTAGAGAATCCCTGAACGACTTTTGTGGGAGCGAAGAATCAGTAGCATTCGAA
GGAGAGCCAAATGAAAAGACATTCATGGGAGACGTCTTAAGTGGTGGAGAATGTGAGAAT
AGTCTCTCAAGAGAAGATTTATTTCATATAGAAGTAGGATCCGAAGAATCGCTAGATGAT
GCCTCAAAATATAATTTCCAAAAGGATTTATCAACTAGCGATAATAGTTCATTCGAAGAT
GATCAGTCATTGAAAAGGGGACTCAAAAGGAACAGCTCAGTATCTAGTCTGGACAGCGAT
ATGGGAAGTTATAAAAATGGAAGATCTAGAGACAGATTGGATATATATTCGGACCTACCT
AGAAGGCTAATACATGGGGATAATGCACCACAAGAAAAGGAGAAATGTCTGGATTTCAAA
GAACTTCTAGAGAGAGAAAGGAACAACCACCAAAGGAATAGTGTAAATATAAAATTAAAA
AATATATACGGAGAAGAACAATTGTATAATGTGAAGAAAGATAGCTTTATTGTAAAATTG
ATATACTATCTGCCGTTTGGCCCAATTGTTACAATGATAATGATAGGTTTTTTTCTACTC
TGCTGGACCCCCTCGATTGGTTTTACTTTTATAGGGTGTAGCTCTATTATATTACCAGCT
TTGTTATACGCGATGCATTCAAACAAAAAACAATTCGAACGTGTATTTGGAAAAGGGAGA
AAAAAGCCAATGAAGGTAAAAAAAGGAGGCGGGAAAATATCTAGATTTTTTAAAGAAGTA
GACAAA---------------------------
>COP05014 .
------------------------------TTAAGGAAAATTTCCCTGTGTTCTATTTTC
GTATTGGCATATCTCAGTTCTGCTAAGTGCGCGTACCGAGACGAATCTGACATATTGTAC
AGCGACGACTCCAGATCCGATTTAGCAAGCAACGGGGCACCCACCGACAGCTACGAATCT
TTAACAGCAAGTAGTGAGTCTCTAGCAGAAAGCAACGATGCACCCAGCAACAGCTATGAA
TCTTTTCCAGAAATTAGAGAAAATCTAACCGCAAGTGAGGAATCCCTAACATCATGTGAG
GAATCCCTAACAGGAAGTAATGAATCCCTAACAGGAAGTAATGAATCCCTAACAGGAAGT
AAT---------------------------------------------------------
---------------------------GAATCCCTAACAGGAAGTAATGAATCCCTAACA
GAAAGTAGAGAATCTCTAGAGGCCAGTAGAGAATCGCTAAGAGCAAGTAGAGAGTCTCTA
GCGGCCAGTAGAGAATCCCTGAACGACTTTTGTGGGAGCGAAGAATCAGTAGCATTCGAA
GGAGAGCCAAATGAAAAGACATTCATGGGAGACGTCTTAAGTGGTGGAGAATGTGAGAAT
AGTCTCTCAAGAGAAGATTTATTTCATATAGAAGTAGGATCCGAAGAATCGCTAGATGAT
GCCTCAAAATATAATTTCCAAAAGGATTTATCAACTAGCGATAATAGTTTATTCGAAGAT
GATCAGTCATTGAAAAGGGGACTCAAAAGGAACAGCTCAGTATCTAGTCTGGACAGCGAT
ATGGGAAGTTATAAAAATGGAAGATCTAGAGACAGATTGGATATATATTCGGACCTACCT
AGAAGGCTAATACATGGGGATAATGCACCACAAGAAAAGGAGAAATGTCTGGATTTCAAA
GAACTTCTAGAGAGAGAAAGGAACAACCACCAAAGGAATAGTGTAAATATAAAATTAAAA
AATATATACGGAGAAGAACAATTGTATAATGTGAAGAAAGATAGCTTTATTGTAAAATTG
ATATACTATCTGCCGTTTGGCCCAATTGTTACAATGATAATGATAGGCTTTATTCTACTC

TGGTCAACGCCCTGGACTGGTTTTATTTTTATAGGGTGTAGCTCTATTATATTACCAGCT
TTGTTATACGCGATGCATTCAAACAAAAAACAATTCGAACGTGTATTTGGAAAAGGGAGA
AAAAAGCCAATGAAGGTAAAAAAAGGAGGCGGGAAAATATCTAGATTTTTTAAAGAAGTA
GACAAA---------------------------
>COP05214 .
------------------------------TTAAGGAAAATTTCCCTGTGTTCTATTTTC
GTATTGGCATATCTCAGTTCTGCTAAGTGCGCGTACCGAGACGAATCTGACATATTGTAC
AGCGACGACTCCAGATCCGATTTAGCAAGCAACGGGGCACCCACCGACAGCTACGAATCT
TTAACAGCAAGTAGTGAGTCTCTAGCAGAAAGCAACGATGCACCCAGCAACAGCTATGAA
TCTTTTCCAGAAATTAGAGAAAATCTAACCGCAAGTGAGGAATCCCTAACATCATGTGAG
GAATCCCTAACAGGAAGTAATGAATCCCTAACAGGAAGTAATGAATCCCTAACAGGAAGT
AAT---------------------------------------------------------
------------------------------------------------GAATCCCTAACA
GAAAGTAGAGAATCTCTAGAGGCCAGTAGAGAATCGCTAAGAGCAAGTAGAGAGTCTCTA
GCGGCCAGTAGAGAATCCCTGAACGACTTTTGTGGGAGCGAAGAATCAGTAGCATTCGAA
GGAGAGCCAAATGAAAAGACATTCATGGGAGACGTCTTAAGTGGTGGAGAATGTGAGAAT
AGTCTCTCAAGAGAAGATTTATTTCATATAGAAGTAGGATCCGAAGAATCGCTAGATGAT
GCCTCAAAATATAATTTCCAAAAGGATTTATCAACTAGCGATAATAGTTTATTCGAAGAT
GATCAGTCATTGAAAAGGGGACTCAAAAGGAACAGCTCAGTATCTAGTCTGGACAGCGAT
ATGGGAAGTTATAAAAATGGAAGATCTAGAGACAGATTGGATATATATTCGGACCTACCT
AGAAGGCTAATACATGGGGATAATGCACCACAAGAAAAGGAGAAATGTCTGGATTTCAAA
GAACTTCTAGAGAGAGAAAGGAACAACCACCAAAGGAATAGTGTAAATATAAAATTAAAA
AATATATACGGAGAAGAACAATTGTATAATGTGAAGAAAGATAGCTTTATTGTAAAATTG
ATATACTATCTGCCGTTTGGCCCAATTGTTACAATGATAATTATAGGCTTTATTCTACTC
TGGTCAACGCCCTGGACTGGTTTTATTTTTATAGGGTGTAGCTCTATTATATTACCAGCT
TTGTTATACGCGATGCATTCAAACAAAAAACAATTCGAACGTGTATTTGGAAAAGGGAGA
AAAAAGCCAATGAAGGTAAAAAAAGGAGGCGGGAAAATATCTAGATTTTTTAAAGAAGTA
GACAAA---------------------------
>COP05314 .
------------------------------TTAAGGAAAATTTCCCTGTGTTCTATTTTC
GTATTGGCATATCTCAGTTCTGCTAAGTGCGCGTACCGAGACGAATCTGACATATTGTAC
AGCGACGACTCCAGATCCGATTTAGCAAGCAACGGGGCACCCACCGACAGCTACGAATCT
TTAACAGCAAGTAGTGAGTCTCTAGCAGAAAGCAACGATGCACCCAGCAACAGCTATGAA
TCTTTTCCAGAAATTAGAGAAAATCTAACCGCAAGTGAGGAATCCCTAACA---------
------------GGAAGTAATGAATCCCTAACAGGAAGTAATGAATCCCTAACAGAAAGT
AAT------------------------------------------GAATCCCTAACAGGA
AGTAATGAATCCCTAACAGGAAGTAATGAATCCCTAACAGGAAGTAATGAATCCCTAACA
GAAAGTAGAGAATCTCTAGAGGCCAGTAGAGAATCGCTAAGAGCAAGTAGAGAGTCTCTA
GCGGCCAGTAGAGAATCCCTGAACGACTTTTGTGGGAGCGAAGAATCAGTAGCATTCGAA
GGAGAGCCAAATGAAAAGACATTCATGGGAGACGTCTTAAGTGGTGGAGAATGTGAGAAT
AGTCTCTCAAGAGAAGATTTATTTCATATAGAAGTAGGATCCGAAGAATCGCTAGATGAT
GCCTCAAAATATAATTTCCAAAAGGATTTATCAACTAGCGATAATAGTTTATTCGAAGAT
GATCAGTCATTGAAAAGGGGACTCAAAAGGAACAGCTCAGTATCTAGTCTGGACAGCGAT
ATGGGAAGTTATAAAAATGGAAGATCTAGAGACAGATTGGATATATATTCGGACCTACCT
AGAAGGCTAATACATGGGGATAATGCACCACAAGAAAAGGAGAAATGTCTGGATTTCAAA
GAACTTCTAGAGAGAGAAAGGAACAACCACCAAAGGAATAGTGTAAATATAAAATTAAAA
AATATATACGGAGAAGAACAATTGTATAATGTGAAGAAAGATAGCTTTATTGTAAAATTG
ATATACTATCTGCCGTTTGGCCCAATTGTTACAATGATAATGATAGGTTTTTTTCTACTC
TGCTGGACCCCCTCGATTGGTTTTATTTTTATAGGGTGTAGCTCTATTATATTACCAGCT
TTGTTATACGCGATGCATTCAAACAAAAAACAATTCGAACGTGTATTTGGAAAAGGGAGA

AAAAAGCCAATGAAGGTAAAAAAAGGAGGCGGGAAAATATCTAGATTTTTTAAAGAAGTA
GACAAA---------------------------
>COP05514 .
------------------------------TTAAGGAAAATTTCCCTGTGTTCTATTTTC
GTATTGGCATATCTCAGTTCTGCTAAGTGCGCGTACCGAGACGAATCTGACATATTGTAC
AGCGACGACTCCAGATCCGATTTAGCAAGCAACGGGGCACCCACCGACAGCTACGAATCT
TTAACAGCAAGTAGTGAGTCTCTAGCAGAAAGCAACGATGCACCCAGCAACAGCTATGAA
TCTTTTCCAGAAATTAGAGAAAATCTAACCGCAAGTGAGGAATCCCTAACATCATGTGAG
GAATCCCTAACAGGAAGTAATGAATCCCTAACAGGAAGTAATGAATCCCTAACAGGAAGT
AAT---------------------------------------------------------
------------------------------------------------GAATCCCTAACA
GAAAGTAGAGAATCTCTAGAGGCCAGTAGAGAATCGCTAAGAGCAAGTAGAGAGTCTCTA
GCGGCCAGTAGAGAATCCCTGAACGACTTTTGTGGGAGCGAAGAATCAGTAGCATGCGAA
GGAGAGCCAAATGAAAAGACATTCATGGGAGACGTCTTAAGTGGTGGAGAATGTGAGAAT
AGTCTCTCAAGAGAAGATTTATTTCATATAGAAGTAGGATCCGAAGAATCGCTAGATGAT
GCCTCAAAATATAATTTCCAAAAGGATTTATCAACTAGCGATAATAGTTTATTCGAAGAT
GATCAGTCATTGAAAAGGGGACTCAAAAGGAACAGCTCAGTATCTAGTCTGGACAGCGAT
ATGGGAAGTTATAAAAATGGAAGATCTAGAGACAGATTGGATATATATTCGGACCTACCT
AGAAGGCTAATACATGGGGATAATGCACCACAAGAAAAGGAGAAATGTCTGGATTTCAAA
GAACTTCTAGAGAGAGAAAGGAACAACCACCAAAGGAATAGTGTAAATATAAAATTAAAA
AATATATACGGAGAAGAACAATTGTATAATGTGAAGAAAGATAGCTTTATTGTAAAATTG
ATATACTATCTGCCGTTTGGCCCAATTGTTACAATGATAATTATAGGCTTTATTCTACTC
TGGTCAACGACTTGGACTGGTTTTATTTTTATAGGGTGTAGCTCTATTATATTACCAGCT
TTGTTATACGCGATGCATTCAAACAAAAAACAATTCGAACGTGTATTTGGAAAAGGGAGA
AAAAAGCCAATGAAGGTAAAAAAAGGAGGCGGGAAAATATCTAGATTTTTTAAAGAAGTA
GACAAA---------------------------
>COT008 .
------------------------------TTAAGGAAAATTTCCCTGTGTTCTATTTTC
GTATTGGCATATCTCAGTTCTGCTAAGTGCGCGTACCGAGACGAATCTGACATATTGTAC
AGCGACGACTCCAGATCCGATTTAGCAAGCAACGGGGCACCCACCGACAGCTACGAATCT
TTAACAGCAAGTAGTGAGTCTCTAGCAGAAAGCAACGATGCACCCAGCAACAGCTATGAA
TCTTTTCCAGAAATTAGAGAAAATCTAACCGCAAGTGAGGAATCCCTAACATCATGTGAG
GAATCCCTAACAGGAAGTAATGAATCCCTAACAGGAAGTAATGAATCCCTAACAGGAAGT
AAT---------------------------------------------------------
---------------------------GAATCCCTAACAGGAAGTAATGAATCCCTAACA
GAAAGTAGAGAATCTCTAGAGGCCAGTAGAGAATCGCTAAGAGCAAGTAGAGAGTCTCTA
GCGGCCAGTAGAGAATCCCTGAACGACTTTTGTGGGAGCGAAGAATCAGTAGCATTCGAA
GGAGAGCCAAATGAAAAGACATTCATGGGAGACGTCTTAAGTGGTGGAGAATGTGAGAAT
AGTCTCTCAAGAGAAGATTTATTTCATATAGAAGTAGGATCCGAAGAATCGCTAGATGAT
GCCTCAAAATATAATTTCCAAAAGGATTTATCAACTAGCGATAATAGTTCATTCGAAGAT
GATCAGTCATTGAAAAGGGGACTCAAAAGGAACAGCTCAGTATCTAGTCTGGACAGCGAT
ATGGGAAGTTATAAAAATAAAAGTTATAGACACAGATTGGATATATATTCGGACCTACCT
AGAAGGCCAATACATGGGGATAATGCACCACAAGAAAAGGAGAAATGTCTGGATTTCAAA
GAACTTCTAGAGAGAGAAAGGAACAACCACCAAAGGAATAGTGTAAATATAAAATTAAAA
AATATATACGGAGAAGAACAATTGTATAATGTGAAGAAAGATAGCTTTATTGTAAAATTG
ATATACTATCTGCCGTTTGGCCCAATTGTTACAATGATAATGATAGGCTTTATTCTACTT

TGGTCAACGCCCTGGACTGGTTTTATTTTTATAGGGTGTAGCTCTATTATATTACCAGCT
TTGTTATACGCGATGCATTCAAACAAAAAACAATTCGAACGTGTATTTGGAAAAGGGAGA
AAAAAGCCAATGAAGGTAAAAAAAGGAGGCGGGAAAATATCTAGATTTTTTAAAGAAGTA
GACAAA---------------------------
>COT017 .
------------------------------TTAAGGAAAATTTCCCTGTGTTCTATTTTC
GTATTGGCATATCTCAGTTCTGCTAAGTGCGCGTACCGAGACGAATCTGACATATTGTAC
AGCGACGACTCCAGATCCGATTTAGCAAGCAACGGGGCACCCACCGACAGCTACGAATCT
TTAACAGCAAGTAGTGAGTCTCTAGCAGAAAGCAACGATGCACCCAGCAACAGCTATGAA
TCTTTTCCAGAAATTAGAGAAAATCTAACCGCAAGTGAGGAATCCCTAACATCATGTGAG
GAATCCCTAACAGGAAGTAATGAATCCCTAACAGGAAGTAATGAATCCCTAACAGGAAGT
AAT---------------------GAATCCCTAACAGGAAGTAATGAATCCCTAACAGGA
AGTAATGAATCCCTAACAGGAAGTAATGAATCCCTAACAGGAAGTAATGAATCCCTAACA
GAAAGTAGAGAATCTCTAGAGGCCAGTAGAGAATCGCTAAGAGCAAGTAGAGAGTCTCTA
GCGGCCAGTAGAGAATCCCTGAACGACTTTTGTGGGAGCGAAGAATCAGTAGCATTCGAA
GGAGAGCCAAATGAAAAGACATTCATGGGAGACGTCTTAAGTGGTGGAGAATGTGAGAAT
AGTCTCTCAAGAGAAGATTTATTTCATATAGAAGTAGGATCCGAAGAATCGCTAGATGAT
GCCTCAAAATATAATTTCCAAAAGGATTTATCAACTAGCGATAATAGTTTATTCGAAGAT
GATCAGTCATTGAAAAGGGGACTCAAAAGGAACAGCTCAGTATCTAGTCTGGACAGCGAT
ATGGGAAGTTATAAAAATGGAAGATCTAGAGACAGATTGGATATATATTCGGACCTACCT
AGAAGGCTAATACATGGGGATAATGCACCACAAGAAAAGGAGAAATGTCTGGATTTCAAA
GAACTTCTAGAGAGAGAAAGGAACAACCACCAAAGGAATAGTGTAAATATAAAATTAAAA
AATATATACGGAGAAGAACAATTGTATAATGTGAAGAAAGATAGCTTTATTGTAAAATTG
ATATACTATCTGCCGTTTGGCCCAATTGTTACAATGATAATTATAGGCTTTATTCTACTC
TGGTCAACGCCCTGGACTGGTTTTATTTTTATAGGGTGTAGCTCTATTATATTACCAGCT
TTGTTATACGCGATGCATTCAAACAAAAAACAATTCGAACGTGTATTTGGAAAAGGGAGA
AAAAAGCCAATGAAGGTAAAAAAAGGAGGCGGGAAAATATCTAGATTTTTTAAAGAAGTA
GACAAA---------------------------
>COT019 .
------------------------------TTAAGGAAAATTTCCCTGTGTTCTATTTTC
GTATTGGCATATCTCAGTTCTGCTAAGTGCGCGTACCGAGACGAATCTGACATATTGTAC
AGCGACGACTCCAGATCCGATTTAGCAAGCAACGGGGCACCCACCGACAGCTACGAATCT
TTAACAGCAAGTAGTGAGTCTCTAGCAGAAAGCAACGATGCACCCAGCAACAGCTATGAA
TCTTTTCCAGAAATTAGAGAAAATCTAACCGCAAGTGAGGAATCCCTAACATCATGTGAG
GAATCCCTAACAGGAAGTAATGAATCCCTAACAGGAAGTAATGAATCCCTAACAGGAAGT
AAT---------------------------------------------------------
------GAATCCCTAACAGGAAGTAATGAATCCCTAACAGGAAGTAATGAATCCCTAACA
GAAAGTAGAGAATCTCTAGAGGCCAGTAGAGAATCGCTAAGAGCAAGTAGAGAGTCTCTA
GCGGCCAGTAGAGAATCCCTGAACGACTTTTGTGGGAGCGAAGAATCAGTAGCATTCGAA
GGAGAGCCAAATGAAAAGACATTCATGGGAGACGTCTTAAGTGGTGGAGAATGTGAGAAT
AGTCTCTCAAGAGAAGATTTATTTCATATAGAAGTAGGATCCGAAGAATCGCTAGATGAT
GCCTCAAAATATAATTTCCAAAAGGATTTATCAACTAGCGATAATAGTTCATTCGAAGAT
GATCAGTCATTGAAAAGGGGACTCAAAAGGAACAGCTCAGTATCTAGTCTGGACAGCGAT
ATGGGAAGTTATAAAAATAAAAGTTATAGACACAGATTGGATATATATTCGGACCTACCT
AGAAGGCCAATACATGGGGATAATGCACCACAAGAAAAGGAGAAATGTCTGGATTTCAAA
GAACTTCTAGAGAGAGAAAGGAACAACCACCAAAGGAATAGTGTAAATATAAAATTAAAA
AATATATACGGAGAAGAACAATTGTATAATGTGAAGAAAGATAGCTTTATTGTAAAATTG
ATATACTATCTGCCGTTTGGCCCAATTGTTACAATGATAATGATAGGCTTTATTCTACTC
TGCTCAACGCCCTCGACTGGTTTTATTTTTATAGGGTGTAGCTCTATTATATTACCAGCT
TTGTTATACGCGATGCATTCAAACAAAAAACAATTCGAACGTGTATTTGGAAAAGGGAGA

AAAAAGCCAATGAAGGTAAAAAAAGGAGGCGGGAAAATATCTAGATTTTTTAAAGAAGTA
GACAAA---------------------------
>COT020 .
------------------------------TTAAGGAAAATTTCCCTGTGTTCTATTTTC
GTATTGGCATATCTCAGTTCTGCTAAGTGCGCGTACCGAGACGAATCTGACATATTGTAC
AGCGACGACTCCAGATCCGATTTAGCAAGCAACGGGGCACCCACCGACAGCTACGAATCT
TTAACAGCAAGTAGTGAGTCTCTAGCAGAAAGCAACGATGCACCCAGCAACAGCTATGAA
TCTTTTCCAGAAATTAGAGAAAATCTAACCGCAAGTGAGGAATCCCTAACA---------
------------GGAAGTAATGAATCCCTAACAGGAAGTAATGAATCCCTAACAGAAAGT
AAT------------------------------------------GAATCCCTAACAGGA
AGTAATGAATCCCTAACAGGAAGTAATGAATCCCTAACAGGAAGTAATGAATCCCTAACA
GAAAGTAGAGAATCTCTAGAGGCCAGTAGAGAATCGCTAAGAGCAAGTAGAGAGTCTCTA
GCGGCCAGTAGAGAATCCCTGAACGACTTTTGTGGGAGCGAAGAATCAGTAGCATTCGAA
GGAGAGCCAAATGAAAAGACATTCATGGGAGACGTCTTAAGTGGTGGAGAATGTGAGAAT
AGTCTCTCAAGAGAAGATTTATTTCATATAGAAGTAGGATCCGAAGAATCGCTAGATGAT
GCCTCAAAATATAATTTCCAAAAGGATTTATCAACTAGCGATAATAGTTTATTCGAAGAT
GATCAGTCATTGAAAAGGGGACTCAAAAGGAACAGCTCAGTATCTAGTCTGGACAGCGAT
ATGGGAAGTTATAAAAATGGAAGATCTAGAGACAGATTGGATATATATTCGGACCTACCT
AGAAGGCTAATACATGGGGATAATGCACCACAAGAAAAGGAGAAATGTCTGGATTTCAAA
GAACTTCTAGAGAGAGAAAGGAACAACCACCAAAGGAATAGTGTAAATATAAAATTAAAA
AATATATACGGAGAAGAACAATTGTATAATGTGAAGAAAGATAGCTTTATTGTAAAATTG
ATATACTATCTGCCGTTTGGCCCAATTGTTACAATGATAATGATAGGTTTTTTTCTACTC
TGCTGGACCCCCTCGATTGGTTTTATTTTTATAGGGTGTAGCTCTATTATATTACCAGCT
TTGTTATACGCGATGCATTCAAACAAAAAACAATTCGAACGTGTATTTGGAAAAGGGAGA
AAAAAGCCAATGAAGGTAAAAAAAGGAGGCGGGAAAATATCTAGATTTTTTAAAGAAGTA
GACAAA---------------------------
>COT021_R .
------------------------------TTAAGGAAAATTTCCCTGTGTTCTATTTTC
GTATTGGCATATCTCAGTTCTGCTAAGTGCGCGTACCGAGACGAATCTGACATATTGTAC
AGCGACGACTCCAGATCCGATTTAGCAAGCAACGGGGCACCCACCGACAGCTACGAATCT
TTAACAGCAAGTAGTGAGTCTCTAGCAGAAAGCAACGATGCACCCAGCAACAGCTATGAA
TCTTTTCCAGAAATTAGAGAAAATCTAACCGCAAGTGAGGAATCCCTAACA---------
------------GGAAGTAATGAATCCCTAACAGGAAGTAATGAATCCCTAACAGAAAGT
AAT------------------------------------------GAATCCCTAACAGGA
AGTAATGAATCCCTAACAGGAAGTAATGAATCCCTAACAGGAAGTAATGAATCCCTAACA
GAAAGTAGAGAATCTCTAGAGGCCAGTAGAGAATCGCTAAGAGCAAGTAGAGAGTCTCTA
GCGGCCAGTAGAGAATCCCTGAACGACTTTTGTGGGAGCGAAGAATCAGTAGCATTCGAA
GGAGAGCCAAATGAAAAGACATTCATGGGAGACGTCTTAAGTGGTGGAGAATGTGAGAAT
AGTCTCTCAAGAGAAGATTTATTTCATATAGAAGTAGGATCCGAAGAATCGCTAGATGAT
GCCTCAAAATATAATTTCCAAAAGGATTTATCAACTAGCGATAATAGTTCATTCGAAGAT
GATCAGTCATTGAAAAGGGGACTCAAAAGGAACAGCTCAGTATCTAGTCTGGACAGCGAT
ATGGGAAGTTATAAAAATGGAAGATCTAGAGACAGATTGGATATATATTCGGACCTACCT
AGAAGGCTAATACATGGGGATAATGCACCACAAGAAAAGGAGAAATGTCTGGATTTCAAA
GAACTTCTAGAGAGAGAAAGGAACAACCACCAAAGGAATAGTGTAAATATAAAATTAAAA
AATATATACGGAGAAGAACAATTGTATAATGTGAAGAAAGATAGCTTTATTGTAAAATTG
ATATACTATCTGCCGTTTGGCCCAATTGTTACAATGATAATTATAGGCTTTATTCTACTC

TGGTCAACGGGCTGGACTGGTTTTATTTTTATAGGGTGTAGCTCTATTATATTACCAGCT
TTGTTATACGCGATGTATTCAAACAAAAAACAATTCGAACGTGTATTTGGAAAAGGGAGA
AAAAAGCCAATGAAGGTAAAAAAAGGAGGCGGGAAAATATCTAGATTTTTTAAAGAAGTA
GACAAA---------------------------
>COM004 .
------------------------------TTAAGGAAAATTTCCCTGTGTTCTATTTTC
GTATTGGCATATCTCAGTTCTGCTAAGTGCGCGTACCGAGACGAATCTGACATATTGTAC
AGCGACGACTCCAGATCCGATTTAGCAAGCAACGGGGCACCCACCGACAGCTACGAATCT
TTAACAGCAAGTAGTGAGTCTCTAGCAGAAAGCAACGATGCACCCAGCAACAGCTATGAA
TCTTTTCCAGAAATTAGAGAAAATCTAACCGCAAGTGAGGAATCCCTAACATCATGTGAG
GAATCCCTAACAGGAAGTAATGAATCCCTAACAGGAAGTAATGAATCCCTAACAGGAAGT
AAT---------------------------------------------------------
------------------------------------------------GAATCCCTAACA
GAAAGTAGAGAATCTCTAGAGGCCAGTAGAGAATCGCTAAGAGCAAGTAGAGAGTCTCTA
GCGGCCAGTAGAGAATCCCTGAACGACTTTTGTGGGAGCGAAGAATCAGTAGCATGCGAA
GGAGAGCCAAATGAAAAGACATTCATGGGAGACGTCTTAAGTGGTGGAGAATGTGAGAAT
AGTCTCTCAAGAGAAGATTTATTTCATATAGAAGTAGGATCCGAAGAATCGCTAGATGAT
GCCTCAAAATATAATTTCCAAAAGGATTTATCAACTAGCGATAATAGTTCATTCGAAGAT
GATCAGTCATTGAAAAGGGGACTCAAAAGGAACAGCTCAGTATCTAGTCTGGACAGCGAT
ATGGGAAGTTATAAAAATAAAAGTTATAGACACAGATTGGATATATATTCGGACCTACCT
AGAAGGCCAATACATGGGGATAATGCACCACAAGAAAAGGAGAAATGTCTGGATTTCAAA
GAACTTCTAGAGAGAGAAAGGAACAACCACCAAAGGAATAGTGTAAATATAAAATTAAAA
AATATATACGGAGAAGAACAATTGTATAATGTGAAGAAAGATAGCTTTATTGTAAAATTG
ATATACTATCTGCCGTTTGGCCCAATTGTTACAATGATAATGATAGGTTTTTTTCTACTC
TGCTGGACCCCCTCGATTGGTTTTACTTTTATAGGGTGTAGCTCTATTATATTACCAGCT
TTGTTATACGCGATGCATTCAAACAAAAAACAATTCGAACGTGTATTTGGAAAAGGGAGA
AAAAAGCCAATGAAGGTAAAAAAAGGAGGCGGGAAAATATCTAGATTTTTTAAAGAAGTA
GACAAA---------------------------
>COM010 .
------------------------------TTAAGGAAAATTTCCCTGTGTTCTATTTTC
GTATTGGCATATCTCAGTTCTGCTAAGTGCGCGTACCGAGACGAATCTGACATATTGTAC
AGCGACGACTCCAGATCCGATTTAGCAAGCAACGGGGCACCCACCGACAGCTACGAATCT
TTAACAGCAAGTAGTGAGTCTCTAGCAGAAAGCAACGATGCACCCAGCAACAGCTATGAA
TCTTTTCCAGAAATTAGAGAAAATCTAACCGCAAGTGAGGAATCCCTAACATCATGTGAG
GAATCCCTAACAGGAAGTAATGAATCCCTAACAGGAAGTAATGAATCCCTAACAGGAAGT
AAT---------------------------------------------------------
------------------------------------------------GAATCCCTAACA
GAAAGTAGAGAATCTCTAGAGGCCAGTAGAGAATCGCTAAGAGCAAGTAGAGAGTCTCTA
GCGGCCAGTAGAGAATCCCTGAACGACTTTTGTGGGAGCGAAGAATCAGTAGCATGCGAA
GGAGAGCCAAATGAAAAGACATTCATGGGAGACGTCTTAAGTGGTGGAGAATGTGAGAAT
AGTCTCTCAAGAGAAGATTTATTTCATATAGAAGTAGGATCCGAAGAATCGCTAGATGAT
GCCTCAAAATATAATTTCCAAAAGGATTTATCAACTAGCGATAATAGTTCATTCGAAGAT
GATCAGTCATTGAAAAGGGGACTCAAAAGGAACAGCTCAGTATCTAGTCTGGACAGCGAT
ATGGGAAGTTATAAAAATAAAAGTTATAGACACAGATTGGATATATATTCGGACCTACCT
AGAAGGCCAATACATGGGGATAATGCACCACAAGAAAAGGAGAAATGTCTGGATTTCAAA
GAACTTCTAGAGAGAGAAAGGAACAACCACCAAAGGAATAGTGTAAATATAAAATTAAAA
AATATATACGGAGAAGAACAATTGTATAATGTGAAGAAAGATAGCTTTATTGTAAAATTG
ATATACTATCTGCCGTTTGGCCCAATTGTTACAATGATAATGATAGGTTTTTTTCTACTC
TGCTGGACCCCCTCGATTGGTTTTACTTTTATAGGGTGTAGCTCTATTATATTACCAGCT
TTGTTATACGCGATGCATTCAAACAAAAAACAATTCGAACGTGTATTTGGAAAAGGGAGA

```
AAAAAGCCAATGAAGGTAAAAAAAGGAGGCGGGAAAATATCTAGATTTTTTAAAGAAGTA
GACAAA---------------------------
>COM011_R .
------------------------------TTAAGGAAAATTTCCCTGTGTTCTATTTTC
GTATTGGCATATCTCAGTTCTGCTAAGTGCGCGTACCGAGACGAATCTGACATATTGTAC
AGCGACGACTCCAGATCCGATTTAGCAAGCAACGGGGCACCCACCGACAGCTACGAATCT
TTAACAGCAAGTAGTGAGTCTCTAGCAGAAAGCAACGATGCACCCAGCAACAGCTATGAA
TCTTTTCCAGAAATTAGAGAAAATCTAACCGCAAGTGAGGAATCCCTAACATCATGTGAG
GAATCCCTAACAGGAAGTAATGAATCCCTAACAGGAAGTAATGAATCCCTAACAGGAAGT
AAT---------------------------------------------------------
---------------------------GAATCCCTAACAGGAAGTAATGAATCCCTAACA
GAAAGTAGAGAATCTCTAGAGGCCAGTAGAGAATCGCTAAGAGCAAGTAGAGAGTCTCTA
GCGGCCAGTAGAGAATCCCTGAACGACTTTTGTGGGAGCGAAGAATCAGTAGCATTCGAA
GGAGAGCCAAATGAAAAGACATTCATGGGAGACGTCTTAAGTGGTGGAGAATGTGAGAAT
AGTCTCTCAAGAGAAGATTTATTTCATATAGAAGTAGGATCCGAAGAATCGCTAGATGAT
GCCTCAAAATATAATTTCCAAAAGGATTTATCAACTAGCGATAATAGTTTATTCGAAGAT
GATCAGTCATTGAAAAGGGGACTCAAAAGGAACAGCTCAGTATCTAGTCTGGACAGCGAT
ATGGGAAGTTATAAAAATAAAAGATCTAGAGACAGATTGGATATATATTCGGACCTACCT
AGAAGGCTAATACATGGGGATAATGCACCACAAGAAAAGGAGAAATGTCTGGATTTCAAA
GAACTTCTAGAGAGAGAAAGGAACAACCACCAAAGGAATAGTGTAAATATAAAATTAAAA
AATATATACGGAGAAGAACAATTGTATAATGTGAAGAAAGATAGCTTTATTGTAAAATTG
ATATACTATCTGCCGTTTGGCCCAATTGTTACAATGATAATGATAGGCTTTATTCTACTC
TGGTCAACGCCCTGGATTGGTTTTATTTTTATAGGGTGTAGCTCTATTATATTACCAGCT
TTGTTATACGCGATGCATTCAAACAAAAAACAATTCGAACGTGTATTTGGAAAAGGGAGA
AAAAAGCCAATGAAGGTAAAAAAAGGAGGCGGGAAAATATCTAGATTTTTTAAAGAAGTA
GACAAA---------------------------
>COM013 .
------------------------------TTAAGGAAAATTTCCCTGTGTTCTATTTTC
GTATTGGCATATCTCAGTTCTGCTAAGTGCGCGTACCGAGACGAATCTGACATATTGTAC
AGCGACGACTCCAGATCCGATTTAGCAAGCAACGGGGCACCCACCGACAGCTACGAATCT
TTAACAGCAAGTAGTGAGTCTCTAGCAGAAAGCAACGATGCACCCAGCAACAGCTATGAA
TCTTTTCCAGAAATTAGAGAAAATCTAACCGCAAGTGAGGAATCCCTAACATCATGTGAG
GAATCCCTAACAGGAAGTAATGAATCCCTAACAGGAAGTAATGAATCCCTAACAGGAAGT
AAT---------------------------------------------------------
---------------------------GAATCCCTAACAGGAAGTAATGAATCCCTAACA
GAAAGTAGAGAATCTCTAGAGGCCAGTAGAGAATCGCTAAGAGCAAGTAGAGAGTCTCTA
GCGGCCAGTAGAGAATCCCTGAACGACTTTTGTGGGAGCGAAGAATCAGTAGCATGCGAA
GGAGAGCCAAATGAAAAGACATTCATGGGAGACGTCTTAAGTGGTGGAGAATGTGAGAAT
AGTCTCTCAAGAGAAGATTTATTTCATATAGAAGTAGGATCCGAAGAATCGCTAGATGAT
GCCTCAAAATATAATTTCCAAAAGGATTTATCAACTAGCGATAATAGTTCATTCGAAGAT
GATCAGTCATTGAAAAGGGGACTCAAAAGGAACAGCTCAGTATCTAGTCTGGACAGCGAT
ATGGGAAGTTATAAAAATAAAAGTTATAGACACAGATTGGATATATATTCGGACCTACCT
AGAAGGCCAATACATGGGGATAATGCACCACAAGAAAAGGAGAAATGTCTGGATTTCAAA
GAACTTCTAGAGAGAGAAAGGAACAACCACCAAAGGAATAGTGTAAATATAAAATTAAAA
AATATATACGGAGAAGAACAATTGTATAATGTGAAGAAAGATAGCTTTATTGTAAAATTG
ATATACTATCTGCCGTTTGGCCCAATTGTTACAATGATAATGATAGGCTTTATTCTACTC
```

TGGTCAACGCCCTCGATTGGTTTTATTTTTATAGGGTGTAGCTCTATTATATTACCAGCT
TTGTTATACGCGATGCATTCAAACAAAAAACAATTCGAACGTGTATTTGGAAAAGGGAGA
AAAAAGCCAATGAAGGTAAAAAAAGGAGGCGGGAAAATATCTAGATTTTTTAAAGAAGTA
GACAAA---------------------------
>COM014_R .
------------------------------TTAAGGAAAATTTCCCTGTGTTCTATTTTC
GTATTGGCATATCTCAGTTCTGCTAAGTGCGCGTACCGAGACGAATCTGACATATTGTAC
AGCGACGACTCCAGATCCGATTTAGCAAGCAACGGGGCACCCACCGACAGCTACGAATCT
TTAACAGCAAGTAGTGAGTCTCTAGCAGAAAGCAACGATGCACCCAGCAACAGCTATGAA
TCTTTTCCAGAAATTAGAGAAAATCTAACCGCAAGTGAGGAATCCCTAACATCATGTGAG
GAATCCCTAACAGGAAGTAATGAATCCCTAACAGGAAGTAATGAATCCCTAACAGGAAGT
AAT---------------------------------------------------------
------------------------------------------------GAATCCCTAACA
GAAAGTAGAGAATCTCTAGAGGCCAGTAGAGAATCGCTAAGAGCAAGTAGAGAGTCTCTA
GCGGCCAGTAGAGAATCCCTGAACGACTTTTGTGGGAGCGAAGAATCAGTAGCATGCGAA
GGAGAGCCAAATGAAAAGACATTCATGGGAGACGTCTTAAGTGGTGGAGAATGTGAGAAT
AGTCTCTCAAGAGAAGATTTATTTCATATAGAAGTAGGATCCGAAGAATCGCTAGATGAT
GCCTCAAAATATAATTTCCAAAAGGATTTATCAACTAGCGATAATAGTTTATTCGAAGAT
GATCAGTCATTGAAAAGGGGACTCAAAAGGAACAGCTCAGTATCTAGTCTGGACAGCGAT
ATGGGAAGTTATAAAAATGGAAGATCTAGAGACAGATTGGATATATATTCGGACCTACCT
AGAAGGCTAATACATGGGGATAATGCACCACAAGAAAAGGAGAAATGTCTGGATTTCAAA
GAACTTCTAGAGAGAGAAAGGAACAACCACCAAAGGAATAGTGTAAATATAAAATTAAAA
AATATATACGGAGAAGAACAATTGTATAATGTGAAGAAAGATAGCTTTATTGTAAAATTG
ATATACTATCTGCCGTTTGGCCCAATTGTTACAATGATAATTATAGGCTTTATTCTACTC
TGGTCAACGCCCTGGACTGGTTTTATTTTTATAGGGTGTAGCTCTATTATATTACCAGCT
TTGTTATACGCGATGCATTCAAACAAAAAACAATTCGAACGTGTATTTGGAAAAGGGAGA
AAAAAGCCAATGAAGGTAAAAAAAGGAGGCGGGAAAATATCTAGATTTTTTAAAGAAGTA
GACAAA---------------------------
>COM015 .
------------------------------TTAAGGAAAATTTCCCTGTGTTCTATTTTC
GTATTGGCATATCTCAGTTCTGCTAAGTGCGCGTACCGAGACGAATCTGACATATTGTAC
AGCGACGACTCCAGATCCGATTTAGCAAGCAACGGGGCACCCACCGACAGCTACGAATCT
TTAACAGCAAGTAGTGAGTCTCTAGCAGAAAGCAACGATGCACCCAGCAACAGCTATGAA
TCTTTTCCAGAAATTAGAGAAAATCTAACCGCAAGTGAGGAATCCCTAACATCATGTGAG
GAATCCCTAACAGGAAGTAATGAATCCCTAACAGGAAGTAATGAATCCCTAACAGGAAGT
AAT------------------------------------------GAATCCCTAACAGGA
AGTAATGAATCCCTAACAGGAAGTAATGAATCCCTAACAGGAAGTAATGAATCCCTAACA
GAAAGTAGAGAATCTCTAGAGGCCAGTAGAGAATCGCTAAGAGCAAGTAGAGAGTCTCTA
GCGGCCAGTAGAGAATCCCTGAACGACTTTTGTGGGAGCGAAGAATCAGTAGCATTCGAA
GGAGAGCCAAATGAAAAGACATTCATGGGAGACGTCTTAAGTGGTGGAGAATGTGAGAAT
AGTCTCTCAAGAGAAGATTTATTTCATATAGAAGTAGGATCCGAAGAATCGCTAGATGAT
GCCTCAAAATATAATTTCCAAAAGGATTTATCAACTAGCGATAATAGTTCATTCGAAGAT
GATCAGTCATTGAAAAGGGGACTCAAAAGGAACAGCTCAGTATCTAGTCTGGACAGCGAT
ATGGGAAGTTATAAAAATAAAAGTTATAGACACAGATTGGATATATATTCGGACCTACCT
AGAAGGCCAATACATGGGGATAATGCACCACAAGAAAAGGAGAAATGTCTGGATTTCAAA
GAACTTCTAGAGAGAGAAAGGAACAACCACCAAAGGAATAGTGTAAATATAAAATTAAAA
AATATATACGGAGAAGAACAATTGTATAATGTGAAGAAAGATAGCTTTATTGTAAAATTG
ATATACTATCTGCCGTTTGGCCCAATTGTTACAATGATAATTATAGGCTTTATTCTACTC
TGGTCAACGCCCTGGACTGGTTTTATTTTTATAGGGTGTAGCTCTATTATATTACCAGCT
TTGTTATACGCGATGCATTCAAACAAAAAACAATTCGAACGTGTATTTGGAAAAGGGAGA

AAAAAGCCAATGAAGGTAAAAAAAGGAGGCGGGAAAATATCTAGATTTTTTAAAGAAGTA
GACAAA---------------------------
>COM016 .
------------------------------TTAAGGAAAATTTCCCTGTGTTCTATTTTC
GTATTGGCATATCTCAGTTCTGCTAAGTGCGCGTACCGAGACGAATCTGACATATTGTAC
AGCGACGACTCCAGATCCGATTTAGCAAGCAACGGGGCACCCACCGACAGCTACGAATCT
TTAACAGCAAGTAGTGAGTCTCTAGCAGAAAGCAACGATGCACCCAGCAACAGCTATGAA
TCTTTTCCAGAAATTAGAGAAAATCTAACCGCAAGTGAGGAATCCCTAACATCATGTGAG
GAATCCCTAACAGGAAGTAATGAATCCCTAACAGGAAGTAATGAATCCCTAACAGGAAGT
AAT---------------------------------------------------------
------------------------------------------------GAATCCCTAACA
GAAAGTAGAGAATCTCTAGAGGCCAGTAGAGAATCGCTAAGAGCAAGTAGAGAGTCTCTA
GCGGCCAGTAGAGAATCCCTGAACGACTTTTGTGGGAGCGAAGAATCAGTAGCATTCGAA
GGAGAGCCAAATGAAAAGACATTCATGGGAGACGTCTTAAGTGGTGGAGAATGTGAGAAT
AGTCTCTCAAGAGAAGATTTATTTCATATAGAAGTAGGATCCGAAGAATCGCTAGATGAT
GCCTCAAAATATAATTTCCAAAAGGATTTATCAACTAGCGATAATAGTTTATTCGAAGAT
GATCAGTCATTGAAAAGGGGACTCAAAAGGAACAGCTCAGTATCTAGTCTGGACAGCGAT
ATGGGAAGTTATAAAAATGGAAGATCTAGAGACAGATTGGATATATATTCGGACCTACCT
AGAAGGCTAATACATGGGGATAATGCACCACAAGAAAAGGAGAAATGTCTGGATTTCAAA
GAACTTCTAGAGAGAGAAAGGAACAACCACCAAAGGAATAGTGTAAATATAAAATTAAAA
AATATATACGGAGAAGAACAATTGTATAATGTGAAGAAAGATAGCTTTATTGTAAAATTG
ATATACTATCTGCCGTTTGGCCCAATTGTTACAATGATAATTATAGGCTTTATTCTACTC
TGGTCAACGCCCTGGACTGGTTTTATTTTTATAGGGTGTAGCTCTATTATATTACCAGCT
TTGTTATACGCGATGCATTCAAACAAAAAACAATTCGAACGTGTATTTGGAAAAGGGAGA
AAAAAGCCAATGAAGGTAAAAAAAGGAGGCGGGAAAATATCTAGATTTTTTAAAGAAGTA
GACAAA---------------------------
>COM017 .
------------------------------TTAAGGAAAATTTCCCTGTGTTCTATTTTC
GTATTGGCATATCTCAGTTCTGCTAAGTGCGCGTACCGAGACGAATCTGACATATTGTAC
AGCGACGACTCCAGATCCGATTTAGCAAGCAACGGGGCACCCACCGACAGCTACGAATCT
TTAACAGCAAGTAGTGAGTCTCTAGCAGAAAGCAACGATGCACCCAGCAACAGCTATGAA
TCTTTTCCAGAAATTAGAGAAAATCTAACCGCAAGTGAGGAATCCCTAACATCATGTGAG
GAATCCCTAACAGGAAGTAATGAATCCCTAACAGGAAGTAATGAATCCCTAACAGGAAGT
AAT---------------------------------------------------------
------------------------------------------------GAATCCCTAACA
GAAAGTAGAGAATCTCTAGAGGCCAGTAGAGAATCGCTAAGAGCAAGTAGAGAGTCTCTA
GCGGCCAGTAGAGAATCCCTGAACGACTTTTGTGGGAGCGAAGAATCAGTAGCATGCGAA
GGAGAGCCAAATGAAAAGACATTCATGGGAGACGTCTTAAGTGGTGGAGAATGTGAGAAT
AGTCTCTCAAGAGAAGATTTATTTCATATAGAAGTAGGATCCGAAGAATCGCTAGATGAT
GCCTCAAAATATAATTTCCAAAAGGATTTATCAACTAGCGATAATAGTTCATTCGAAGAT
GATCAGTCATTGAAAAGGGGACTCAAAAGGAACAGCTCAGTATCTAGTCTGGACAGCGAT
ATGGGAAGTTATAAAAATAAAAGTTATAGACACAGATTGGATATATATTCGGACCTACCT
AGAAGGCCAATACATGGGGATAATGCACCACAAGAAAAGGAGAAATGTCTGGATTTCAAA
GAACTTCTAGAGAGAGAAAGGAACAACCACCAAAGGAATAGTGTAAATATAAAATTAAAA
AATATATACGGAGAAGAACAATTGTATAATGTGAAGAAAGATAGCTTTATTGTAAAATTG
ATATACTATCTGCCGTTTGGCCCAATTGTTACAATGATAATGATAGGTTTTTTTCTACTC

TGCTGGACCCCCTCGATTGGTTTTACTTTTATAGGGTGTAGCTCTATTATATTACCAGCT
TTGTTATACGCGATGCATTCAAACAAAAAACAATTCGAACGTGTATTTGGAAAAGGGAGA
AAAAAGCCAATGAAGGTAAAAAAAGGAGGCGGGAAAATATCTAGATTTTTTAAAGAAGTA
GACAAA---------------------------
>BO210 .
------------------------------TTAAGGAAAATTTCCCTGTGTTCTATTTTC
GTATTGGCATATCTCAGTTCTGCTAAGTGCGCGTACCGAGACGAATCTGACATATTGTAC
AGCGACGACTCCAGATCCGATTTAGCAAGCAACGGGGCACCCACCGACAGCTACGAATCT
TTAACAGCAAGTAGTGAGTCTCTAGCAGAAAGCAACGATGCACCCAGCAACAGCTATGAA
TCTTTTCCAGAAATTAGAGAAAATCTAACCGCAAGTGAGGAATCCCTAACATCATGTGAG
GAATCCCTAACAGGAAGTAATGAATCCCTAACAGGAAGTAATGAATCCCTAACAGGAAGT
AAT---------------------------------------------------------
---------------------------GAATCCCTAACAGGAAGTAATGAATCCCTAACA
GAAAGTAGAGAATCTCTAGAGGCCAGTAGAGAATCGCTAAGAGCAAGTAGAGAGTCTCTA
GCGGCCAGTAGAGAATCCCTGAACGACTTTTGTGGGAGCGAAGAATCAGTAGCATGCGAA
GGAGAGCCAAATGAAAAGACATTCATGGGAGACGTCTTAAGTGGTGGAGAATGTGAGAAT
AGTCTCTCAAGAGAAGATTTATTTCATATAGAAGTAGGATCCGAAGAATCGCTAGATGAT
GCCTCAAAATATAATTTCCAAAAGGATTTATCAACTAGCGATAATAGTTCATTCGAAGAT
GATCAGTCATTGAAAAGGGGACTCAAAAGGAACAGCTCAGTATCTAGTCTGGACAGCGAT
ATGGGAAGTTATAAAAATGGAAGATCTAGAGACAGATTGGATATATATTCGGACCTACCT
AGAAGGCTAATACATGGGGATAATGCACCACAAGAAAAGGAGAAATGTCTGGATTTCAAA
GAACTTCTAGAGAGAGAAAGGAACAACCACCAAAGGAATAGTGTAAATATAAAATTAAAA
AATATATACGGAGAAGAACAATTGTATAATGTGAAGAAAGATAGCTTTATTGTAAAATTG
ATATACTATCTGCCGTTTGGCCCAATTGTTACAATGATAATGATAGGTTTTTTTCTACTC
TGCTGGACCCCCTCGATTGGTTTTACTTTTATAGGGTGTAGCTCTATTATATTACCAGCT
TTGTTATACGCGATGTATTCAAACAAAAAACAATTCGAACGTGTATTTGGAAAAGGGAGA
AAAAAGCCAATGAAGGTAAAAAAAGGAGGCGGGAAAATATCTAGATTTTTTAAGAAAGTA
GACAAA---------------------------
>BO217_R .
------------------------------TTAAGGAAAATTTCCCTGTGTTCTATTTTC
GTATTGGCATATCTCAGTTCTGCTAAGTGCGCGTACCGAGACGAATCTGACATATTGTAC
AGCGACGACTCCAGATCCGATTTAGCAAGCAACGGGGCACCCACCGACAGCTACGAATCT
TTAACAGCAAGTAGTGAGTCTCTAGCAGAAAGCAACGATGCACCCAGCAACAGCTATGAA
TCTTTTCCAGAAATTAGAGAAAATCTAACCGCAAGTGAGGAATCCCTAACATCATGTGAG
GAATCCCTAACAGGAAGTAATGAATCCCTAACAGGAAGTAATGAATCCCTAACAGGAAGT
AAT---------------------------------------------------------
---------------------------GAATCCCTAACAGGAAGTAATGAATCCCTAACA
GAAAGTAGAGAATCTCTAGAGGCCAGTAGAGAATCGCTAAGAGCAAGTAGAGAGTCTCTA
GCGGCCAGTAGAGAATCCCTGAACGACTTTTGTGGGAGCGAAGAATCAGTAGCATTCGAA
GGAGAGCCAAATGAAAAGACATTCATGGGAGACGTCTTAAGTGGTGGAGAATGTGAGAAT
AGTCTCTCAAGAGAAGATTTATTTCATATAGAAGTAGGATCCGAAGAATCGCTAGATGAT
GCCTCAAAATATAATTTCCAAAAGGATTTATCAACTAGCGATAATAGTTCATTCGAAGAT
GATCAGTCATTGAAAAGGGGACTCAAAAGGAACAGCTCAGTATCTAGTCTGGACAGCGAT
ATGGGAAGTTATAAAAATAAAAGTTATAGACACAGATTGGATATATATTCGGACCTACCT
AGAAGGCCAATACATGGGGATAATGCACCACAAGAAAAGGAGAAATGTCTGGATTTCAAA
GAACTTCTAGAGAGAGAAAGGAACAACCACCAAAGGAATAGTGTAAATATAAAATTAAAA
AATATATACGGAGAAGAACAATTGTATAATGTGAAGAAAGATAGCTTTATTGTAAAATTG
ATATACTATCTGCCGTTTGGCCCAATTGTTACAATGATAATGATAGGCTTTATTCTACTC
TGCTCAACGCCCTCGACTGGTTTTATTTTTATAGGGTGTAGCTCTATTATATTACCAGCT
TTGTTATACGCGATGCATTCAAACAAAAAACAATTCGAACGTGTATTTGGAAAAGGGAGA

AAAAAGCCAATGAAGGTAAAAAAAGGAGGCGGGAAAATATCTAGATTTTTTAAAGAAGTA
GACAAA---------------------------
>BO219 .
------------------------------TTAAGGAAAATTTCCCTGTGTTCTATTTTC
GTATTGGCATATCTCAGTTCTGCTAAGTGCGCGTACCGAGACGAATCTGACATATTGTAC
AGCGACGACTCCAGATCCGATTTAGCAAGCAACGGGGCACCCACCGACAGCTACGAATCT
TTAACAGCAAGTAGTGAGTCTCTAGCAGAAAGCAACGATGCACCCAGCAACAGCTATGAA
TCTTTTCCAGAAATTAGAGAAAATCTAACCGCAAGTGAGGAATCCCTAACATCATGTGAG
GAATCCCTAACAGGAAGTAATGAATCCCTAACAGGAAGTAATGAATCCCTAACAGGAAGT
AAT------------------------------------------GAATCCCTAACAGGA
AGTAATGAATCCCTAACAGGAAGTAATGAATCCCTAACAGGAAGTAATGAATCCCTAACA
GAAAGTAGAGAATCTCTAGAGGCCAGTAGAGAATCGCTAAGAGCAAGTAGAGAGTCTCTA
GCGGCCAGTAGAGAATCCCTGAACGACTTTTGTGGGAGCGAAGAATCAGTAGCATGCGAA
GGAGAGCCAAATGAAAAGACATTCATGGGAGACGTCTTAAGTGGTGGAGAATGTGAGAAT
AGTCTCTCAAGAGAAGATTTATTTCATATAGAAGTAGGATCCGAAGAATCGCTAGATGAT
GCCTCAAAATATAATTTCCAAAAGGATTTATCAACTAGCGATAATAGTTCATTCGAAGAT
GATCAGTCATTGAAAAGGGGACTCAAAAGGAACAGCTCAGTATCTAGTCTGGACAGCGAT
ATGGGAAGTTATAAAAATAAAAGTTATAGACACAGATTGGATATATATTCGGACCTACCT
AGAAGGCCAATACATGGGGATAATGCACCACAAGAAAAGGAGAAATGTCTGGATTTCAAA
GAACTTCTAGAGAGAGAAAGGAACAACCACCAAAGGAATAGTGTAAATATAAAATTAAAA
AATATATACGGAGAAGAACAATTGTATAATGTGAAGAAAGATAGCTTTATTGTAAAATTG
ATATACTATCTGCCGTTTGGCCCAATTGTTACAATGATAATTATAGGCTTTATTCTACTC
TGGTCAACGCCCTGGACTGGTTTTATTTTTATAGGGTGTAGCTCTATTATATTACCAGCT
TTGATATACGCGATGCATTCAAACAAAAAACAATTCGAACGTGTATTTGGAAAAGGGAGA
AAAAAGCCAATGAAGGTAAAAAAAGGAGGCGGGAAAATATCTAGATTTTTTAAAGAAGTA
GACAAA---------------------------
>BO234 .
------------------------------TTAAGGAAAATTTCCCTGTGTTCTATTTTC
GTATTGGCATATCTCAGTTCTGCTAAGTGCGCGTACCGAGACGAATCTGACATATTGTAC
AGCGACGACTCCAGATCCGATTTAGCAAGCAACGGGGCACCCACCGACAGCTACGAATCT
TTAACAGCAAGTAGTGAGTCTCTAGCAGAAAGCAACGATGCACCCAGCAACAGCTATGAA
TCTTTTCCAGAAATTAGAGAAAATCTAACCGCAAGTGAGGAATCCCTAACATCATGTGAG
GAATCCCTAACAGGAAGTAATGAATCCCTAACAGGAAGTAATGAATCCCTAACAGGAAGT
AAT---------------------------------------------------------
---------------------------GAATCCCTAACAGGAAGTAATGAATCCCTAACA
GAAAGTAGAGAATCTCTAGAGGCCAGTAGAGAATCGCTAAGAGCAAGTAGAGAGTCTCTA
GCGGCCAGTAGAGAATCCCTGAACGACTTTTGTGGGAGCGAAGAATCAGTAGCATTCGAA
GGAGAGCCAAATGAAAAGACATTCATGGGAGACGTCTTAAGTGGTGGAGAATGTGAGAAT
AGTCTCTCAAGAGAAGATTTATTTCATATAGAAGTAGGATCCGAAGAATCGCTAGATGAT
GCCTCAAAATATAATTTCCAAAAGGATTTATCAACTAGCGATAATAGTTCATTCGAAGAT
GATCAGTCATTGAAAAGGGGACTCAAAAGGAACAGCTCAGTATCTAGTCTGGACAGCGAT
ATGGGAAGTTATAAAAATAAAAGTTATAGACACAGATTGGATATATATTCGGACCTACCT
AGAAGGCCAATACATGGGGATAATGCACCACAAGAAAAGGAGAAATGTCTGGATTTCAAA
GAACTTCTAGAGAGAGAAAGGAACAACCACCAAAGGAATAGTGTAAATATAAAATTAAAA
AATATATACGGAGAAGAACAATTGTATAATGTGAAGAAAGATAGCTTTATTGTAAAATTG
ATATACTATCTGCCGTTTGGCCCAATTGTTACAATGATAATGATAGGCTTTATTCTACTC

TGCTCAACGCCCTCGACTGGTTTTATTTTTATAGGGTGTAGCTCTATTATATTACCAGCT
TTGTTATACGCGATGCATTCAAACAAAAAACAATTCGAACGTGTATTTGGAAAAGGGAGA
AAAAAGCCAATGAAGGTAAAAAAAGGAGGCGGGAAAATATCTAGATTTTTTAAAGAAGTA
GACAAA---------------------------
>BO251_R .
------------------------------TTAAGGAAAATTTCCCTGTGTTCTATTTTC
GTATTGGCATATCTCAGTTCTGCTAAGTGCGCGTACCGAGACGAATCTGACATATTGTAC
AGCGACGACTCCAGATCCGATTTAGCAAGCAACGGGGCACCCACCGACAGCTACGAATCT
TTAACAGCAAGTAGTGAGTCTCTAGCAGAAAGCAACGATGCACCCAGCAACAGCTATGAA
TCTTTTCCAGAAATTAGAGAAAATCTAACCGCAAGTGAGGAATCCCTAACATCATGTGAG
GAATCCCTAACAGGAAGTAATGAATCCCTAACAGGAAGTAATGAATCCCTAACAGGAAGT
AAT------------------------------------------GAATCCCTAACAGGA
AGTAATGAATCCCTAACAGGAAGTAATGAATCCCTAACAGGAAGTAATGAATCCCTAACA
GAAAGTAGAGAATCTCTAGAGGCCAGTAGAGAATCGCTAAGAGCAAGTAGAGAGTCTCTA
GCGGCCAGTAGAGAATCCCTGAACGACTTTTGTGGGAGCGAAGAATCAGTAGCATGCGAA
GGAGAGCCAAATGAAAAGACATTCATGGGAGACGTCTTAAGTGGTGGAGAATGTGAGAAT
AGTCTCTCAAGAGAAGATTTATTTCATATAGAAGTAGGATCCGAAGAATCGCTAGATGAT
GCCTCAAAATATAATTTCCAAAAGGATTTATCAACTAGCGATAATAGTTCATTCGAAGAT
GATCAGTCATTGAAAAGGGGACTCAAAAGGAACAGCTCAGTATCTAGTCTGGACAGCGAT
ATGGGAAGTTATAAAAATAAAAGTTATAGACACAGATTGGATATATATTCGGACCTACCT
AGAAGGCCAATACATGGGGATAATGCACCACAAGAAAAGGAGAAATGTCTGGATTTCAAA
GAACTTCTAGAGAGAGAAAGGAACAACCACCAAAGGAATAGTGTAAATATAAAATTAAAA
AATATATACGGAGAAGAACAATTGTATAATGTGAAGAAAGATAGCTTTATTGTAAAATTG
ATATACTATCTGCCGTTTGGCCCAATTGTTACAATGATAATTATAGGCTTTATTCTACTC
TGGTCAACGCCCTGGACTGGTTTTATTTTTATAGGGTGTAGCTCTATTATATTACCAGCT
TTGATATACGCGATGCATTCAAACAAAAAACAATTCGAACGTGTATTTGGAAAAGGGAGA
AAAAAGCCAATGAAGGTAAAAAAAGGAGGCGGGAAAATATCTAGATTTTTTAAAGAAGTA
GACAAA---------------------------
>BO257 .
------------------------------TTAAGGAAAATTTCCCTGTGTTCTATTTTC
GTATTGGCATATCTCAGTTCTGCTAAGTGCGCGTACCGAGACGAATCTGACATATTGTAC
AGCGACGACTCCAGATCCGATTTAGCAAGCAACGGGGCACCCACCGACAGCTACGAATCT
TTAACAGCAAGTAGTGAGTCTCTAGCAGAAAGCAACGATGCACCCAGCAACAGCTATGAA
TCTTTTCCAGAAATTAGAGAAAATCTAACCGCAAGTGAGGAATCCCTAACATCATGTGAG
GAATCCCTAACAGGAAGTAATGAATCCCTAACAGGAAGTAATGAATCCCTAACAGGAAGT
AAT---------------------------------------------------------
------------------------------------------------GAATCCCTAACA
GAAAGTAGAGAATCTCTAGAGGCCAGTAGAGAATCGCTAAGAGCAAGTAGAGAGTCTCTA
GCGGCCAGTAGAGAATCCCTGAACGACTTTTGTGGGAGCGAAGAATCAGTAGCATTCGAA
GGAGAGCCAAATGAAAAGACATTCATGGGAGACGTCTTAAGTGGTGGAGAATGTGAGAAT
AGTCTCTCAAGAGAAGATTTATTTCATATAGAAGTAGGATCCGAAGAATCGCTAGATGAT
GCCTCAAAATATAATTTCCAAAAGGATTTATCAACTAGCGATAATAGTTCATTCGAAGAT
GATCAGTCATTGAAAAGGGGACTCAAAAGGAACAGCTCAGAATCTAGTCTGGACAGCGAT
ATGGGAAGTTATAAAAATAAAAGTTATAGACACAGATTGGATATATATTCGGACCTACCT
AGAAGGCCAATACATGGGGATAATGCACCACAAGAAAAGGAGAAATGTCTGGATTTCAAA
GAACTTCTAGAGAGAGAAAGGAACAACCACCAAAGGAATAGTGTAAATATAAAATTAAAA
AATATATACGGAGAAGAACAATTGTATAATGTGAAGAAAGATAGCTTTATTGTAAAATTG
ATATACTATCTGCCGTTTGGCCCAATTGTTACAATGATAATGATAGGCTTTATTCTACTC
TGCTGGACCCCCTCGATTGGTTTTACTTTTATAGGGTGTAGCTCTATTATATTACCAGCT
TTGTTATACGCGATGTATTCAAACAAAAAACAATTCGAACGTGTATTTGGAAAAGGGAGA

AAAAAGCCAATGAAGGTAAAAAAAGGAGGCGGGAAAATATCTAGATTTTTTAAGAAAGTA
GACAAA---------------------------
>BO269 .
------------------------------TTAAGGAAAATTTCCCTGTGTTCTATTTTC
GTATTGGCATATCTCAGTTCTGCTAAGTGCGCGTACCGAGACGAATCTGACATATTGTAC
AGCGACGACTCCAGATCCGATTTAGCAAGCAACGGGGCACCCACCGACAGCTACGAATCT
TTAACAGCAAGTAGTGAGTCTCTAGCAGAAAGCAACGATGCACCCAGCAACAGCTATGAA
TCTTTTCCAGAAATTAGAGAAAATCTAACCGCAAGTGAGGAATCCCTAACATCATGTGAG
GAATCCCTAACAGGAAGTAATGAATCCCTAACAGGAAGTAATGAATCCCTAACAGGAAGT
AAT---------------------------------------------------------
------------------------------------------------GAATCCCTAACA
GAAAGTAGAGAATCTCTAGAGGCCAGTAGAGAATCGCTAAGAGCAAGTAGAGAGTCTCTA
GCGGCCAGTAGAGAATCCCTGAACGACTTTTGTGGGAGCGAAGAATCAGTAGCATTCGAA
GGAGAGCCAAATGAAAAGACATTCATGGGAGACGTCTTAAGTGGTGGAGAATGTGAGAAT
AGTCTCTCAAGAGAAGATTTATTTCATATAGAAGTAGGATCCGAAGAATCGCTAGATGAT
GCCTCAAAATATAATTTCCAAAAGGATTTATCAACTAGCGATAATAGTTCATTCGAAGAT
GATCAGTCATTGAAAAGGGGACTCAAAAGGAACAGCTCAGTATCTAGTCTGGACAGCGAT
ATGGGAAGTTATAAAAATAAAAGTTATAGACACAGATTGGATATATATTCGGACCTACCT
AGAAGGCCAATACATGGGGATAATGCACCACAAGAAAAGGAGAAATGTCTGGATTTCAAA
GAACTTCTAGAGAGAGAAAGGAACAACCACCAAAGGAATAGTGTAAATATAAAATTAAAA
AATATATACGGAGAAGAACAATTGTATAATGTGAAGAAAGATAGCTTTATTGTAAAATTG
ATATACTATCTGCCGTTTGGCCCAATTGTTACAATGATAATTATAGGCTTTATTCTACTC
TGGTCAACGCCCTGGACTGGTTTTATTTTTATAGGGTGTAGCTCTATTATATTACCAGCT
TTGATATACGCGATGCATTCAAACAAAAAACAATTCGAACGTGTATTTGGAAAAGGGAGA
AAAAAGCCAATGAAGGTAAAAAAAGGAGGCGGGAAAATATCTAGATTTTTTAAGAAAGTA
GACAAA---------------------------
>BO288 .
------------------------------TTAAGGAAAATTTCCCTGTGTTCTATTTTC
GTATTGGCATATCTCAGTTCTGCTAAGTGCGCGTACCGAGACGAATCTGACATATTGTAC
AGCGACGACTCCAGATCCGATTTAGCAAGCAACGGGGCACCCACCGACAGCTACGAATCT
TTAACAGCAAGTAGTGAGTCTCTAGCAGAAAGCAACGATGCACCCAGCAACAGCTATGAA
TCTTTTCCAGAAATTAGAGAAAATCTAACCGCAAGTGAGGAATCCCTAACATCATGTGAG
GAATCCCTAACAGGAAGTAATGAATCCCTAACAGGAAGTAATGAATCCCTAACAGGAAGT
AAT---------------------------------------------------------
------------------------------------------------GAATCCCTAACA
GAAAGTAGAGAATCTCTAGAGGCCAGTAGAGAATCGCTAAGAGCAAGTAGAGAGTCTCTA
GCGGCCAGTAGAGAATCCCTGAACGACTTTTGTGGGAGCGAAGAATCAGTAGCATTCGAA
GGAGAGCCAAATGAAAAGACATTCATGGGAGACGTCTTAAGTGGTGGAGAATGTGAGAAT
AGTCTCTCAAGAGAAGATTTATTTCATATAGAAGTAGGATCCGAAGAATCGCTAGATGAT
GCCTCAAAATATAATTTCCAAAAGGATTTATCAACTAGCGATAATAGTTCATTCGAAGAT
GATCAGTCATTGAAAAGGGGACTCAAAAGGAACAGCTCAGAATCTAGTCTGGACAGCGAT
ATGGGAAGTTATAAAAATAAAAGTTATAGACACAGATTGGATATATATTCGGACCTACCT
AGAAGGCCAATACATGGGGATAATGCACCACAAGAAAAGGAGAAATGTCTGGATTTCAAA
GAACTTCTAGAGAGAGAAAGGAACAACCACCAAAGGAATAGTGTAAATATAAAATTAAAA
AATATATACGGAGAAGAACAATTGTATAATGTGAAGAAAGATAGCTTTATTGTAAAATTG
ATATACTATCTGCCGTTTGGCCCAATTGTTACAATGATAATGATAGGCTTTATTCTACTC

TGCTGGACCCCCTCGATTGGTTTTACTTTTATAGGGTGTAGCTCTATTATATTACCAGCT
TTGTTATACGCGATGTATTCAAACAAAAAACAATTCGAACGTGTATTTGGAAAAGGGAGA
AAAAAGCCAATGAAGGTAAAAAAAGGAGGCGGGAAAATATCTAGATTTTTTAAAGAAGTA
GACAAA---------------------------
>BO292 .
------------------------------TTAAGGAAAATTTCCCTGTGTTCTATTTTC
GTATTGGCATATCTCAGTTCTGCTAAGTGCGCGTACCGAGACGAATCTGACATATTGTAC
AGCGACGACTCCAGATCCGATTTAGCAAGCAACGGGGCACCCACCGACAGCTACGAATCT
TTAACAGCAAGTAGTGAGTCTCTAGCAGAAAGCAACGATGCACCCAGCAACAGCTATGAA
TCTTTTCCAGAAATTAGAGAAAATCTAACCGCAAGTGAGGAATCCCTAACATCATGTGAG
GAATCCCTAACAGGAAGTAATGAATCCCTAACAGGAAGTAATGAATCCCTAACAGGAAGT
AAT---------------------------------------------------------
---------------------------GAATCCCTAACAGGAAGTAATGAATCCCTAACA
GAAAGTAGAGAATCTCTAGAGGCCAGTAGAGAATCGCTAAGAGCAAGTAGAGAGTCTCTA
GCGGCCAGTAGAGAATCCCTGAACGACTTTTGTGGGAGCGAAGAATCAGTAGCATTCGAA
GGAGAGCCAAATGAAAAGACATTCATGGGAGACGTCTTAAGTGGTGGAGAATGTGAGAAT
AGTCTCTCAAGAGAAGATTTATTTCATATAGAAGTAGGATCCGAAGAATCGCTAGATGAT
GCCTCAAAATATAATTTCCAAAAGGATTTATCAACTAGCGATAATAGTTTATTCGAAGAT
GATCAGTCATTGAAAAGGGGACTCAAAAGGAACAGCTCAGTATCTAGTCTGGACAGCGAT
ATGGGAAGTTATAAAAATGGAAGATCTAGAGACAGATTGGATATATATTCGGACCTACCT
AGAAGGCTAATACATGGGGATAATGCACCACAAGAAAAGGAGAAATGTCTGGATTTCAAA
GAACTTCTAGAGAGAGAAAGGAACAACCACCAAAGGAATAGTGTAAATATAAAATTAAAA
AATATATACGGAGAAGAACAATTGTATAATGTGAAGAAAGATAGCTTTATTGTAAAATTG
ATATACTATCTGCCGTTTGGCCCAATTGTTACAATGATAATGATAGGTTTTATTCTACTC
TGCTCAACGCCCTCGACTGGTTTTATTTTTATAGGGTGTAGCTCTATTATATTACCAGCT
TTGTTATACGCGATGCATTCAAACAAAAAACAATTCGAACGTGTATTTGGAAAAGGGAGA
AAAAAGCCAATGAAGGTAAAAAAAGGAGGCGGGAAAATATCTAGATTTTTTAAAGAAGTA
GACAAA---------------------------
>BO693 .
------------------------------TTAAGGAAAATTTCCCTGTGTTCTATTTTC
GTATTGGCATATCTCAGTTCTGCTAAGTGCGCGTACCGAGACGAATCTGACATATTGTAC
AGCGACGACTCCAGATCCGATTTAGCAAGCAACGGGGCACCCACCGACAGCTACGAATCT
TTAACAGCAAGTAGTGAGTCTCTAGCAGAAAGCAACGATGCACCCAGCAACAGCTATGAA
TCTTTTCCAGAAATTAGAGAAAATCTAACCGCAAGTGAGGAATCCCTAACATCATGTGAG
GAATCCCTAACAGGAAGTAAT---------------------------------------
------------------------------------------------------------
------------------------------------------------GAATCCCTAACA
GAAAGTAGAGAATCTCTAGAGGCCAGTAGAGAATCGCTAAGAGCAAGTAGAGAGTCTCTA
GCGGCCAGTAGAGAATCCCTGAACGACTTTTGTGGGAGCGAAGAATCAGTAGCATTCGAA
GGAGAGCCAAATGAAAAGACATTCATGGGAGACGTCTTAAGTGGTGGAGAATGTGAGAAT
AGTCTCTCAAGAGAAGATTTATTTCATATAGAAGTAGGATCCGAAGAATCGCTAGATGAT
GCCTCAAAATATAATTTCCAAAAGGATTTATCAACTAGCGATAATAGTTTATTCGAAGAT
GATCAGTCATTGAAAAGGGGACTCAAAAGGAACAGCTCAGTATCTAGTCTGGACAGCGAT
ATGGGAAGTTATAAAAATAAAAGTTATAGACACAGATTGGATATATATTCGGACCTACCT
AGAAGGCTAATACATGGGGATAATGCACCACAAGAAAAGGAGAAATGTCTGGATTTCAAA
GAACTTCTAGAGAGAGAAAGGAACAACCACCAAAGGAATAGTGTAAATATAAAATTAAAA
AATATATACGGAGAAGAACAATTGTATAATGTGAAGAAAGATAGCTTTATTGTAAAATTG
ATATACTATCTGCCGTTTGGCCCAATTGTTACAATGATAATGATAGGCTTTATTCTACTC
TGCTCAACGCCCTCGATTGGTTTTATTTTTATAGGGTGTAGCTCTATTATATTACCAGCT
TTGTTATACGCGATGCATTCAAACAAAAAACAATTCGAACGTGTATTTGGAAAAGGGAGA

AAAAAGCCAATGAAGGTAAAAAAAGGAGGCGGGAAAATATCTAGATTTTTTAAAGAAGTA
GACAAA---------------------------
>BO861 .
------------------------------TTAAGGAAAATTTCCCTGTGTTCTATTTTC
GTATTGGCATATCTCAGTTCTGCTAAGTGCGCGTACCGAGACGAATCTGACATATTGTAC
AGCGACGACTCCAGATCCGATTTAGCAAGCAACGGGGCACCCACCGACAGCTACGAATCT
TTAACAGCAAGTAGTGAGTCTCTAGCAGAAAGCAACGATGCACCCAGCAACAGCTATGAA
TCTTTTCCAGAAATTAGAGAAAATCTAACCGCAAGTGAGGAATCCCTAACATCATGTGAG
GAATCCCTAACAGGAAGTAATGAATCCCTAACAGGAAGTAATGAATCCCTAACAGGAAGT
AAT------------------------------------------GAATCCCTAACAGGA
AGTAATGAATCCCTAACAGGAAGTAATGAATCCCTAACAGGAAGTAATGAATCCCTAACA
GAAAGTAGAGAATCTCTAGAGGCCAGTAGAGAATCGCTAAGAGCAAGTAGAGAGTCTCTA
GCGGCCAGTAGAGAATCCCTGAACGACTTTTGTGGGAGCGAAGAATCAGTAGCATGCGAA
GGAGAGCCAAATGAAAAGACATTCATGGGAGACGTCTTAAGTGGTGGAGAATGTGAGAAT
AGTCTCTCAAGAGAAGATTTATTTCATATAGAAGTAGGATCCGAAGAATCGCTAGATGAT
GCCTCAAAATATAATTTCCAAAAGGATTTATCAACTAGCGATAATAGTTCATTCGAAGAT
GATCAGTCATTGAAAAGGGGACTCAAAAGGAACAGCTCAGTATCTAGTCTGGACAGCGAT
ATGGGAAGTTATAAAAATAAAAGTTATAGACACAGATTGGATATATATTCGGACCTACCT
AGAAGGCCAATACATGGGGATAATGCACCACAAGAAAAGGAGAAATGTCTGGATTTCAAA
GAACTTCTAGAGAGAGAAAGGAACAACCACCAAAGGAATAGTGTAAATATAAAATTAAAA
AATATATACGGAGAAGAACAATTGTATAATGTGAAGAAAGATAGCTTTATTGTAAAATTG
ATATACTATCTGCCGTTTGGCCCAATTGTTACAATGATAATTATAGGCTTTATTCTACTC
TGGTCAACGCCCTGGACTGGTTTTATTTTTATAGGGTGTAGCTCTATTATATTACCAGCT
TTGATATACGCGATGCATTCAAACAAAAAACAATTCGAACGTGTATTTGGAAAAGGGAGA
AAAAAGCCAATGAAGGTAAAAAAAGGAGGCGGGAAAATATCTAGATTTTTTAAAGAAGTA
GACAAA---------------------------
>BO864 .
------------------------------TTAAGGAAAATTTCCCTGTGTTCTATTTTC
GTATTGGCATATCTCAGTTCTGCTAAGTGCGCGTACCGAGACGAATCTGACATATTGTAC
AGCGACGACTCCAGATCCGATTTAGCAAGCAACGGGGCACCCACCGACAGCTACGAATCT
TTAACAGCAAGTAGTGAGTCTCTAGCAGAAAGCAACGATGCACCCAGCAACAGCTATGAA
TCTTTTCCAGAAATTAGAGAAAATCTAACCGCAAGTGAGGAATCCCTAACATCATGTGAG
GAATCCCTAACAGGAAGTAATGAATCCCTAACAGGAAGTAATGAATCCCTAACAGGAAGT
AAT---------------------------------------------------------
---------------------------GAATCCCTAACAGGAAGTAATGAATCCCTAACA
GAAAGTAGAGAATCTCTAGAGGCCAGTAGAGAATCGCTAAGAGCAAGTAGAGAGTCTCTA
GCGGCCAGTAGAGAATCCCTGAACGACTTTTGTGGGAGCGAAGAATCAGTAGCATTCGAA
GGAGAGCCAAATGAAAAGACATTCATGGGAGACGTCTTAAGTGGTGGAGAATGTGAGAAT
AGTCTCTCAAGAGAAGATTTATTTCATATAGAAGTAGGATCCGAAGAATCGCTAGATGAT
GCCTCAAAATATAATTTCCAAAAGGATTTATCAACTAGCGATAATAGTTCATTCGAAGAT
GATCAGTCATTGAAAAGGGGACTCAAAAGGAACAGCTCAGTATCTAGTCTGGACAGCGAT
ATGGGAAGTTATAAAAATGGAAGATCTAGAGACAGATTGGATATATATTCGGACCTACCT
AGAAGGCTAATACATGGGGATAATGCACCACAAGAAAAGGAGAAATGTCTGGATTTCAAA
GAACTTCTAGAGAGAGAAAGGAACAACCACCAAAGGAATAGTGTAAATATAAAATTAAAA
AATATATACGGAGAAGAACAATTGTATAATGTGAAGAAAGATAGCTTTATTGTAAAATTG
ATATACTATCTGCCGTTTGGCCCAATTGTTACAATGATAATGATAGGCTTTATTCTACTC

TGGTCAACGCCCTGGACTGGTTTTATTTTTATAGGGTGTAGCTCTATTATATTACCAGCT
TTGTTATACGCGATGCATTCAAACAAAAAACAATTCGAACGTGTATTTGGAAAAGGGAGA
AAAAAGCCAATGAAGGTAAAAAAAGGAGGCGGGAAAATATCTAGATTTTTTAAAGAAGTA
GACAAA---------------------------
>BO908 .
------------------------------TTAAGGAAAATTTCCCTGTGTTCTATTTTC
GTATTGGCATATCTCAGTTCTGCTAAGTGCGCGTACCGAGACGAATCTGACATATTGTAC
AGCGACGACTCCAGATCCGATTTAGCAAGCAACGGGGCACCCACCGACAGCTACGAATCT
TTAACAGCAAGTAGTGAGTCTCTAGCAGAAAGCAACGATGCACCCAGCAACAGCTATGAA
TCTTTTCCAGAAATTAGAGAAAATCTAACCGCAAGTGAGGAATCCCTAACATCATGTGAG
GAATCCCTAACAGGAAGTAATGAATCCCTAACAGGAAGTAATGAATCCCTAACAGGAAGT
AAT---------------------------------------------------------
------------------------------------------------GAATCCCTAACA
GAAAGTAGAGAATCTCTAGAGGCCAGTAGAGAATCGCTAAGAGCAAGTAGAGAGTCTCTA
GCGGCCAGTAGAGAATCCCTGAACGACTTTTGTGGGAGCGAAGAATCAGTAGCATTCGAA
GGAGAGCCAAATGAAAAGACATTCATGGGAGACGTCTTAAGTGGTGGAGAATGTGAGAAT
AGTCTCTCAAGAGAAGATTTATTTCATATAGAAGTAGGATCCGAAGAATCGCTAGATGAT
GCCTCAAAATATAATTTCCAAAAGGATTTATCAACTAGCGATAATAGTTCATTCGAAGAT
GATCAGTCATTGAAAAGGGGACTCAAAAGGAACAGCTCAGAATCTAGTCTGGACAGCGAT
ATGGGAAGTTATAAAAATAAAAGTTATAGACACAGATTGGATATATATTCGGACCTACCT
AGAAGGCCAATACATGGGGATAATGCACCACAAGAAAAGGAGAAATGTCTGGATTTCAAA
GAACTTCTAGAGAGAGAAAGGAACAACCACCAAAGGAATAGTGTAAATATAAAATTAAAA
AATATATACGGAGAAGAACAATTGTATAATGTGAAGAAAGATAGCTTTATTGTAAAATTG
ATATACTATCTGCCGTTTGGCCCAATTGTTACAATGATAATGATAGGCTTTATTCTACTC
TGCTGGACCCCCTCGATTGGTTTTACTTTTATAGGGTGTAGCTCTATTATATTACCAGCT
TTGTTATACGCGATGTATTCAAACAAAAAACAATTCGAACGTGTATTTGGAAAAGGGAGA
AAAAAGCCAATGAAGGTAAAAAAAGGAGGCGGGAAAATATCTAGATTTTTTAAAGAAGTA
GACAAA---------------------------
>BO908_R .
------------------------------TTAAGGAAAATTTCCCTGTGTTCTATTTTC
GTATTGGCATATCTCAGTTCTGCTAAGTGCGCGTACCGAGACGAATCTGACATATTGTAC
AGCGACGACTCCAGATCCGATTTAGCAAGCAACGGGGCACCCACCGACAGCTACGAATCT
TTAACAGCAAGTAGTGAGTCTCTAGCAGAAAGCAACGATGCACCCAGCAACAGCTATGAA
TCTTTTCCAGAAATTAGAGAAAATCTAACCGCAAGTGAGGAATCCCTAACATCATGTGAG
GAATCCCTAACAGGAAGTAATGAATCCCTAACAGGAAGTAATGAATCCCTAACAGGAAGT
AAT---------------------------------------------------------
------------------------------------------------GAATCCCTAACA
GAAAGTAGAGAATCTCTAGAGGCCAGTAGAGAATCGCTAAGAGCAAGTAGAGAGTCTCTA
GCGGCCAGTAGAGAATCCCTGAACGACTTTTGTGGGAGCGAAGAATCAGTAGCATTCGAA
GGAGAGCCAAATGAAAAGACATTCATGGGAGACGTCTTAAGTGGTGGAGAATGTGAGAAT
AGTCTCTCAAGAGAAGATTTATTTCATATAGAAGTAGGATCCGAAGAATCGCTAGATGAT
GCCTCAAAATATAATTTCCAAAAGGATTTATCAACTAGCGATAATAGTTCATTCGAAGAT
GATCAGTCATTGAAAAGGGGACTCAAAAGGAACAGCTCAGAATCTAGTCTGGACAGCGAT
ATGGGAAGTTATAAAAATAAAAGTTATAGACACAGATTGGATATATATTCGGACCTACCT
AGAAGGCCAATACATGGGGATAATGCACCACAAGAAAAGGAGAAATGTCTGGATTTCAAA
GAACTTCTAGAGAGAGAAAGGAACAACCACCAAAGGAATAGTGTAAATATAAAATTAAAA
AATATATACGGAGAAGAACAATTGTATAATGTGAAGAAAGATAGCTTTATTGTAAAATTG
ATATACTATCTGCCGTTTGGCCCAATTGTTACAATGATAATGATAGGCTTTATTCTACTC
TGCTGGACCCCCTCGATTGGTTTTACTTTTATAGGGTGTAGCTCTATTATATTACCAGCT
TTGTTATACGCGATGTATTCAAACAAAAAACAATTCGAACGTGTATTTGGAAAAGGGAGA

AAAAAGCCAATGAAGGTAAAAAAAGGAGGCGGGAAAATATCTAGATTTTTTAAAGAAGTA
GACAAA---------------------------
>BO919 .
------------------------------TTAAGGAAAATTTCCCTGTGTTCTATTTTC
GTATTGGCATATCTCAGTTCTGCTAAGTGCGCGTACCGAGACGAATCTGACATATTGTAC
AGCGACGACTCCAGATCCGATTTAGCAAGCAACGGGGCACCCACCGACAGCTACGAATCT
TTAACAGCAAGTAGTGAGTCTCTAGCAGAAAGCAACGATGCACCCAGCAACAGCTATGAA
TCTTTTCCAGAAATTAGAGAAAATCTAACCGCAAGTGAGGAATCCCTAACATCATGTGAG
GAATCCCTAACAGGAAGTAATGAATCCCTAACAGGAAGTAATGAATCCCTAACAGGAAGT
AAT---------------------------------------------------------
---------------------------GAATCCCTAACAGGAAGTAATGAATCCCTAACA
GAAAGTAGAGAATCTCTAGAGGCCAGTAGAGAATCGCTAAGAGCAAGTAGAGAGTCTCTA
GCGGCCAGTAGAGAATCCCTGAACGACTTTTGTGGGAGCGAAGAATCAGTAGCATGCGAA
GGAGAGCCAAATGAAAAGACATTCATGGGAGACGTCTTAAGTGGTGGAGAATGTGAGAAT
AGTCTCTCAAGAGAAGATTTATTTCATATAGAAGTAGGATCCGAAGAATCGCTAGATGAT
GCCTCAAAATATAATTTCCAAAAGGATTTATCAACTAGCGATAATAGTTCATTCGAAGAT
GATCAGTCATTGAAAAGGGGACTCAAAAGGAACAGCTCAGTATCTAGTCTGGACAGCGAT
ATGGGAAGTTATAAAAATGGAAGATCTAGAGACAGATTGGATATATATTCGGACCTACCT
AGAAGGCTAATACATGGGGATAATGCACCACAAGAAAAGGAGAAATGTCTGGATTTCAAA
GAACTTCTAGAGAGAGAAAGGAACAACCACCAAAGGAATAGTGTAAATATAAAATTAAAA
AATATATACGGAGAAGAACAATTGTATAATGTGAAGAAAGATAGCTTTATTGTAAAATTG
ATATACTATCTGCCGTTTGGCCCAATTGTTACAATGATAATGATAGGCTTTATTCTACTC
TGGTCAACGGGCTGGACTGGTTTTATTTTTATAGGGTGTAGCTCTATTATATTACCAGCT
TTGTTATACGCGATGCATTCAAACAAAAAACAATTCGAACGTGTATTTGGAAAAGGGAGA
AAAAAGCCAATGAAGGTAAAAAAAGGAGGCGGGAAAATATCTAGATTTTTTAAAGAAGTA
GACAAA---------------------------
>BO920_R .
------------------------------TTAAGGAAAATTTCCCTGTGTTCTATTTTC
GTATTGGCATATCTCAGTTCTGCTAAGTGCGCGTACCGAGACGAATCTGACATATTGTAC
AGCGACGACTCCAGATCCGATTTAGCAAGCAACGGGGCACCCACCGACAGCTACGAATCT
TTAACAGCAAGTAGTGAGTCTCTAGCAGAAAGCAACGATGCACCCAGCAACAGCTATGAA
TCTTTTCCAGAAATTAGAGAAAATCTAACCGCAAGTGAGGAATCCCTAACATCATGTGAG
GAATCCCTAACAGGAAGTAATGAATCCCTAACAGGAAGTAATGAATCCCTAACAGGAAGT
AAT---------------------------------------------------------
---------------------------GAATCCCTAACAGGAAGTAATGAATCCCTAACA
GAAAGTAGAGAATCTCTAGAGGCCAGTAGAGAATCGCTAAGAGCAAGTAGAGAGTCTCTA
GCGGCCAGTAGAGAATCCCTGAACGACTTTTGTGGGAGCGAAGAATCAGTAGCATGCGAA
GGAGAGCCAAATGAAAAGACATTCATGGGAGACGTCTTAAGTGGTGGAGAATGTGAGAAT
AGTCTCTCAAGAGAAGATTTATTTCATATAGAAGTAGGATCCGAAGAATCGCTAGATGAT
GCCTCAAAATATAATTTCCAAAAGGATTTATCAACTAGCGATAATAGTTCATTCGAAGAT
GATCAGTCATTGAAAAGGGGACTCAAAAGGAACAGCTCAGTATCTAGTCTGGACAGCGAT
ATGGGAAGTTATAAAAATGGAAGATCTAGAGACAGATTGGATATATATTCGGACCTACCT
AGAAGGCTAATACATGGGGATAATGCACCACAAGAAAAGGAGAAATGTCTGGATTTCAAA
GAACTTCTAGAGAGAGAAAGGAACAACCACCAAAGGAATAGTGTAAATATAAAATTAAAA
AATATATACGGAGAAGAACAATTGTATAATGTGAAGAAAGATAGCTTTATTGTAAAATTG
ATATACTATCTGCCGTTTGGCCCAATTGTTACAATGATAATGATAGGTTTTTTTCTACTC

TGCTGGACCCCCTCGATTGGTTTTACTTTTATAGGGTGTAGCTCTATTATATTACCAGCT
TTGTTATACGCGATGTATTCAAACAAAAAACAATTCGAACGTGTATTTGGAAAAGGGAGA
AAAAAGCCAATGAAGGTAAAAAAAGGAGGCGGGAAAATATCTAGATTTTTTAAAGAAGTA
GACAAA---------------------------
>BO921 .
------------------------------TTAAGGAAAATTTCCCTGTGTTCTATTTTC
GTATTGGCATATCTCAGTTCTGCTAAGTGCGCGTACCGAGACGAATCTGACATATTGTAC
AGCGACGACTCCAGATCCGATTTAGCAAGCAACGGGGCACCCACCGACAGCTACGAATCT
TTAACAGCAAGTAGTGAGTCTCTAGCAGAAAGCAACGATGCACCCAGCAACAGCTATGAA
TCTTTTCCAGAAATTAGAGAAAATCTAACCGCAAGTGAGGAATCCCTAACATCATGTGAG
GAATCCCTAACAGGAAGTAATGAATCCCTAACAGGAAGTAATGAATCCCTAACAGGAAGT
AAT---------------------------------------------------------
------------------------------------------------GAATCCCTAACA
GAAAGTAGAGAATCTCTAGAGGCCAGTAGAGAATCGCTAAGAGCAAGTAGAGAGTCTCTA
GCGGCCAGTAGAGAATCCCTGAACGACTTTTGTGGGAGCGAAGAATCAGTAGCATTCGAA
GGAGAGCCAAATGAAAAGACATTCATGGGAGACGTCTTAAGTGGTGGAGAATGTGAGAAT
AGTCTCTCAAGAGAAGATTTATTTCATATAGAAGTAGGATCCGAAGAATCGCTAGATGAT
GCCTCAAAATATAATTTCCAAAAGGATTTATCAACTAGCGATAATAGTTCATTCGAAGAT
GATCAGTCATTGAAAAGGGGACTCAAAAGGAACAGCTCAGAATCTAGTCTGGACAGCGAT
ATGGGAAGTTATAAAAATAAAAGTTATAGACACAGATTGGATATATATTCGGACCTACCT
AGAAGGCCAATACATGGGGATAATGCACCACAAGAAAAGGAGAAATGTCTGGATTTCAAA
GAACTTCTAGAGAGAGAAAGGAACAACCACCAAAGGAATAGTGTAAATATAAAATTAAAA
AATATATACGGAGAAGAACAATTGTATAATGTGAAGAAAGATAGCTTTATTGTAAAATTG
ATATACTATCTGCCGTTTGGCCCAATTGTTACAATGATAATGATAGGCTTTATTCTACTC
TGCTGGACCCCCTCGATTGGTTTTACTTTTATAGGGTGTAGCTCTATTATATTACCAGCT
TTGTTATACGCGATGTATTCAAACAAAAAACAATTCGAACGTGTATTTGGAAAAGGGAGA
AAAAAGCCAATGAAGGTAAAAAAAGGAGGCGGGAAAATATCTAGATTTTTTAAAGAAGTA
GACAAA---------------------------
>BO923 .
------------------------------TTAAGGAAAATTTCCCTGTGTTCTATTTTC
GTATTGGCATATCTCAGTTCTGCTAAGTGCGCGTACCGAGACGAATCTGACATATTGTAC
AGCGACGACTCCAGATCCGATTTAGCAAGCAACGGGGCACCCACCGACAGCTACGAATCT
TTAACAGCAAGTAGTGAGTCTCTAGCAGAAAGCAACGATGCACCCAGCAACAGCTATGAA
TCTTTTCCAGAAATTAGAGAAAATCTAACCGCAAGTGAGGAATCCCTAACATCATGTGAG
GAATCCCTAACAGGAAGTAATGAATCCCTAACAGGAAGTAATGAATCCCTAACAGGAAGT
AAT---------------------------------------------------------
---------------------------GAATCCCTAACAGGAAGTAATGAATCCCTAACA
GAAAGTAGAGAATCTCTAGAGGCCAGTAGAGAATCGCTAAGAGCAAGTAGAGAGTCTCTA
GCGGCCAGTAGAGAATCCCTGAACGACTTTTGTGGGAGCGAAGAATCAGTAGCATGCGAA
GGAGAGCCAAATGAAAAGACATTCATGGGAGACGTCTTAAGTGGTGGAGAATGTGAGAAT
AGTCTCTCAAGAGAAGATTTATTTCATATAGAAGTAGGATCCGAAGAATCGCTAGATGAT
GCCTCAAAATATAATTTCCAAAAGGATTTATCAACTAGCGATAATAGTTCATTCGAAGAT
GATCAGTCATTGAAAAGGGGACTCAAAAGGAACAGCTCAGTATCTAGTCTGGACAGCGAT
ATGGGAAGTTATAAAAATGGAAGATATAGAGACAGATTGGATATATATTCGGACCTACCT
AGAAGGCCAATACATGGGGATAATGCACCACAAGAAAAGGAGAAATGTCTGGATTTCAAA
GAACTTCTAGAGAGAGAAAGGAACAACCACCAAAGGAATAGTGTAAATATAAAATTAAAA
AATATATACGGAGAAGAACAATTGTATAATGTGAAGAAAGATAGCTTTATTGTAAAATTG
ATATACTATCTGCCGTTTGGCCCAATTGTTACAATGATAATGATAGGTTTTATTCTACTC
TGCTCGACCCCCTCGATTGGTTTTACTTTTATAGGGTGTAGCTCTATTATATTACCAGCT
TTGTTATACGCGATGTATTCAAACAAAAAACAATTCGAACGTGTATTTGGAAAAGGGAGA

AAAAAGCCAATGAAGGTAAAAAAAGGAGGCGGGAAAATATCTAGATTTTTTAAAGAAGTA
GACAAA---------------------------
>BO931 .
------------------------------TTAAGGAAAATTTCCCTGTGTTCTATTTTC
GTATTGGCATATCTCAGTTCTGCTAAGTGCGCGTACCGAGACGAATCTGACATATTGTAC
AGCGACGACTCCAGATCCGATTTAGCAAGCAACGGGGCACCCACCGACAGCTACGAATCT
TTAACAGCAAGTAGTGAGTCTCTAGCAGAAAGCAACGATGCACCCAGCAACAGCTATGAA
TCTTTTCCAGAAATTAGAGAAAATCTAACCGCAAGTGAGGAATCCCTAACATCATGTGAG
GAATCCCTAACAGGAAGTAAT---------------------------------------
------------------------------------------------------------
-------------------------------------------------GAATCCCTAACA
GAAAGTAGAGAATCTCTAGAGGCCAGTAGAGAATCGCTAAGAGCAAGTAGAGAGTCTCTA
GCGGCCAGTAGAGAATCCCTGAACGACTTTTGTGGGAGCGAAGAATCAGTAGCATTCGAA
GGAGAGCCAAATGAAAAGACATTCATGGGAGACGTCTTAAGTGGTGGAGAATGTGAGAAT
AGTCTCTCAAGAGAAGATTTATTTCATATAGAAGTAGGATCCGAAGAATCGCTAGATGAT
GCCTCAAAATATAATTTCCAAAAGGATTTATCAACTAGCGATAATAGTTTATTCGAAGAT
GATCAGTCATTGAAAAGGGGACTCAAAAGGAACAGCTCAGTATCTAGTCTGGACAGCGAT
ATGGGAAGTTATAAAAATAAAAGTTATAGACACAGATTGGATATATATTCGGACCTACCT
AGAAGGCTAATACATGGGGATAATGCACCACAAGAAAAGGAGAAATGTCTGGATTTCAAA
GAACTTCTAGAGAGAGAAAGGAACAACCACCAAAGGAATAGTGTAAATATAAAATTAAAA
AATATATACGGAGAAGAACAATTGTATAATGTGAAGAAAGATAGCTTTATTGTAAAATTG
ATATACTATCTGCCGTTTGGCCCAATTGTTACAATGATAATGATAGGCTTTATTCTACTC
TGCTCAACGCCCTCGATTGGTTTTATTTTTATAGGGTGTAGCTCTATTATATTACCAGCT
TTGTTATACGCGATGCATTCAAACAAAAAACAATTCGAACGTGTATTTGGAAAAGGGAGA
AAAAAGCCAATGAAGGTAAAAAAAGGAGGCGGGAAAATATCTAGATTTTTTAAAGAAGTA
GACAAA---------------------------
>BO938 .
------------------------------TTAAGGAAAATTTCCCTGTGTTCTATTTTC
GTATTGGCATATCTCAGTTCTGCTAAGTGCGCGTACCGAGACGAATCTGACATATTGTAC
AGCGACGACTCCAGATCCGATTTAGCAAGCAACGGGGCACCCACCGACAGCTACGAATCT
TTAACAGCAAGTAGTGAGTCTCTAGCAGAAAGCAACGATGCACCCAGCAACAGCTATGAA
TCTTTTCCAGAAATTAGAGAAAATCTAACCGCAAGTGAGGAATCCCTAACATCATGTGAG
GAATCCCTAACAGGAAGTAATGAATCCCTAACAGGAAGTAATGAATCCCTAACAGGAAGT
AAT---------------------------------------------------------
-------------------------------------------------GAATCCCTAACA
GAAAGTAGAGAATCTCTAGAGGCCAGTAGAGAATCGCTAAGAGCAAGTAGAGAGTCTCTA
GCGGCCAGTAGAGAATCCCTGAACGACTTTTGTGGGAGCGAAGAATCAGTAGCATTCGAA
GGAGAGCCAAATGAAAAGACATTCATGGGAGACGTCTTAAGTGGTGGAGAATGTGAGAAT
AGTCTCTCAAGAGAAGATTTATTTCATATAGAAGTAGGATCCGAAGAATCGCTAGATGAT
GCCTCAAAATATAATTTCCAAAAGGATTTATCAACTAGCGATAATAGTTCATTCGAAGAT
GATCAGTCATTGAAAAGGGGACTCAAAAGGAACAGCTCAGAATCTAGTCTGGACAGCGAT
ATGGGAAGTTATAAAAATAAAAGTTATAGACACAGATTGGATATATATTCGGACCTACCT
AGAAGGCCAATACATGGGGATAATGCACCACAAGAAAAGGAGAAATGTCTGGATTTCAAA
GAACTTCTAGAGAGAGAAAGGAACAACCACCAAAGGAATAGTGTAAATATAAAATTAAAA
AATATATACGGAGAAGAACAATTGTATAATGTGAAGAAAGATAGCTTTATTGTAAAATTG
ATATACTATCTGCCGTTTGGCCCAATTGTTACAATGATAATGATAGGCTTTATTCTACTC

TGCTGGACCCCCTCGATTGGTTTTACTTTTATAGGGTGTAGCTCTATTATATTACCAGCT
TTGTTATACGCGATGCATTCAAACAAAAAACAATTCGAACGTGTATTTGGAAAAGGGAGA
AAAAAGCCAATGAAGGTAAAAAAAGGAGGCGGGAAAATATCTAGATTTTTTAAAGAAGTA
GACAAA---------------------------
>BO939 .
------------------------------TTAAGGAAAATTTCCCTGTGTTCTATTTTC
GTATTGGCATATCTCAGTTCTGCTAAGTGCGCGTACCGAGACGAATCTGACATATTGTAC
AGCGACGACTCCAGATCCGATTTAGCAAGCAACGGGGCACCCACCGACAGCTACGAATCT
TTAACAGCAAGTAGTGAGTCTCTAGCAGAAAGCAACGATGCACCCAGCAACAGCTATGAA
TCTTTTCCAGAAATTAGAGAAAATCTAACCGCAAGTGAGGAATCCCTAACATCATGTGAG
GAATCCCTAACAGGAAGTAATGAATCCCTAACAGGAAGTAATGAATCCCTAACAGGAAGT
AAT---------------------------------------------------------
---------------------------GAATCCCTAACAGGAAGTAATGAATCCCTAACA
GAAAGTAGAGAATCTCTAGAGGCCAGTAGAGAATCGCTAAGAGCAAGTAGAGAGTCTCTA
GCGGCCAGTAGAGAATCCCTGAACGACTTTTGTGGGAGCGAAGAATCAGTAGCATGCGAA
GGAGAGCCAAATGAAAAGACATTCATGGGAGACGTCTTAAGTGGTGGAGAATGTGAGAAT
AGTCTCTCAAGAGAAGATTTATTTCATATAGAAGTAGGATCCGAAGAATCGCTAGATGAT
GCCTCAAAATATAATTTCCAAAAGGATTTATCAACTAGCGATAATAGTTCATTCGAAGAT
GATCAGTCATTGAAAAGGGGACTCAAAAGGAACAGCTCAGTATCTAGTCTGGACAGCGAT
ATGGGAAGTTATAAAAATGGAAGATCTAGAGACAGATTGGATATATATTCGGACCTACCT
AGAAGGCTAATACATGGGGATAATGCACCACAAGAAAAGGAGAAATGTCTGGATTTCAAA
GAACTTCTAGAGAGAGAAAGGAACAACCACCAAAGGAATAGTGTAAATATAAAATTAAAA
AATATATACGGAGAAGAACAATTGTATAATGTGAAGAAAGATAGCTTTATTGTAAAATTG
ATATACTATCTGCCGTTTGGCCCAATTGTTACAATGATAATGATAGGCTTTATTCTACTC
TGCTCAACGCCCTCGACTGGTTTTATTTTTATAGGGTGTAGCTCTATTATATTACCAGCT
TTGTTATACGCGATGCATTCAAACAAAAAACAATTCGAACGTGTATTTGGAAAAGGGAGA
AAAAAGCCAATGAAGGTAAAAAAAGGAGGCGGGAAAATATCTAGATTTTTTAAAGAAGTA
GACAAA---------------------------
>BO943 .
------------------------------TTAAGGAAAATTTCCCTGTGTTCTATTTTC
GTATTGGCATATCTCAGTTCTGCTAAGTGCGCGTACCGAGACGAATCTGACATATTGTAC
AGCGACGACTCCAGATCCGATTTAGCAAGCAACGGGGCACCCACCGACAGCTACGAATCT
TTAACAGCAAGTAGTGAGTCTCTAGCAGAAAGCAACGATGCACCCAGCAACAGCTATGAA
TCTTTTCCAGAAATTAGAGAAAATCTAACCGCAAGTGAGGAATCCCTAACATCATGTGAG
GAATCCCTAACAGGAAGTAATGAATCCCTAACAGGAAGTAATGAATCCCTAACAGGAAGT
AAT---------------------------------------------------------
------------------------------------------------GAATCCCTAACA
GAAAGTAGAGAATCTCTAGAGGCCAGTAGAGAATCGCTAAGAGCAAGTAGAGAGTCTCTA
GCGGCCAGTAGAGAATCCCTGAACGACTTTTGTGGGAGCGAAGAATCAGTAGCATTCGAA
GGAGAGCCAAATGAAAAGACATTCATGGGAGACGTCTTAAGTGGTGGAGAATGTGAGAAT
AGTCTCTCAAGAGAAGATTTATTTCATATAGAAGTAGGATCCGAAGAATCGCTAGATGAT
GCCTCAAAATATAATTTCCAAAAGGATTTATCAACTAGCGATAATAGTTCATTCGAAGAT
GATCAGTCATTGAAAAGGGGACTCAAAAGGAACAGCTCAGAATCTAGTCTGGACAGCGAT
ATGGGAAGTTATAAAAATAAAAGTTATAGACACAGATTGGATATATATTCGGACCTACCT
AGAAGGCCAATACATGGGGATAATGCACCACAAGAAAAGGAGAAATGTCTGGATTTCAAA
GAACTTCTAGAGAGAGAAAGGAACAACCACCAAAGGAATAGTGTAAATATAAAATTAAAA
AATATATACGGAGAAGAACAATTGTATAATGTGAAGAAAGATAGCTTTATTGTAAAATTG
ATATACTATCTGCCGTTTGGCCCAATTGTTACAATGATAATGATAGGCTTTATTCTACTC
TGCTGGACCCCCTCGATTGGTTTTACTTTTATAGGGTGTAGCTCTATTATATTACCAGCT
TTGTTATACGCGATGTATTCAAACAAAAAACAATTCGAACGTGTATTTGGAAAAGGGAGA

AAAAAGCCAATGAAGGTAAAAAAAGGAGGCGGGAAAATATCTAGATTTTTTAAGAAAGTA
GACAAA---------------------------
>BO948 .
------------------------------TTAAGGAAAATTTCCCTGTGTTCTATTTTC
GTATTGGCATATCTCAGTTCTGCTAAGTGCGCGTACCGAGACGAATCTGACATATTGTAC
AGCGACGACTCCAGATCCGATTTAGCAAGCAACGGGGCACCCACCGACAGCTACGAATCT
TTAACAGCAAGTAGTGAGTCTCTAGCAGAAAGCAACGATGCACCCAGCAACAGCTATGAA
TCTTTTCCAGAAATTAGAGAAAATCTAACCGCAAGTGAGGAATCCCTAACATCATGTGAG
GAATCCCTAACAGGAAGTAAT---------------------------------------
------------------------------------------------------------
------------------------------------------------GAATCCCTAACA
GAAAGTAGAGAATCTCTAGAGGCCAGTAGAGAATCGCTAAGAGCAAGTAGAGAGTCTCTA
GCGGCCAGTAGAGAATCCCTGAACGACTTTTGTGGGAGCGAAGAATCAGTAGCATTCGAA
GGAGAGCCAAATGAAAAGACATTCATGGGAGACGTCTTAAGTGGTGGAGAATGTGAGAAT
AGTCTCTCAAGAGAAGATTTATTTCATATAGAAGTAGGATCCGAAGAATCGCTAGATGAT
GCCTCAAAATATAATTTCCAAAAGGATTTATCAACTAGCGATAATAGTTCATTCGAAGAT
GATCAGTCATTGAAAAGGGGACTCAAAAGGAACAGCTCAGTATCTAGTCTGGACAGCGAT
ATGGGAAGTTATAAAAATAAAAGTTATAGACACAGATTGGATATATATTCGGACCTACCT
AGAAGGCTAATACATGGGGATAATGCACCACAAGAAAAGGAGAAATGTCTGGATTTCAAA
GAACTTCTAGAGAGAGAAAGGAACAACCACCAAAGGAATAGTGTAAATATAAAATTAAAA
AATATATACGGAGAAGAACAATTGTATAATGTGAAGAAAGATAGCTTTATTGTAAAATTG
ATATACTATCTGCCGTTTGGCCCAATTGTTACAATGATAATGATAGGCTTTATTCTACTC
TGCTCAACGCCCTCGATTGGTTTTATTTTTATAGGGTGTAGCTCTATTATATTACCAGCT
TTGTTATACGCGATGCATTCAAACAAAAAACAATTCGAACGTGTATTTGGAAAAGGGAGA
AAAAAGCCAATGAAGGTAAAAAAAGGAGGCGGGAAAATATCTAGATTTTTTAAAGAAGTA
GACAAA---------------------------
>BO950 .
------------------------------TTAAGGAAAATTTCCCTGTGTTCTATTTTC
GTATTGGCATATCTCAGTTCTGCTAAGTGCGCGTACCGAGACGAATCTGACATATTGTAC
AGCGACGACTCCAGATCCGATTTAGCAAGCAACGGGGCACCCACCGACAGCTACGAATCT
TTAACAGCAAGTAGTGAGTCTCTAGCAGAAAGCAACGATGCACCCAGCAACAGCTATGAA
TCTTTTCCAGAAATTAGAGAAAATCTAACCGCAAGTGAGGAATCCCTAACATCATGTGAG
GAATCCCTAACAGGAAGTAATGAATCCCTAACAGGAAGTAATGAATCCCTAACAGGAAGT
AAT---------------------------------------------------------
---------------------------GAATCCCTAACAGGAAGTAATGAATCCCTAACA
GAAAGTAGAGAATCTCTAGAGGCCAGTAGAGAATCGCTAAGAGCAAGTAGAGAGTCTCTA
GCGGCCAGTAGAGAATCCCTGAACGACTTTTGTGGGAGCGAAGAATCAGTAGCATTCGAA
GGAGAGCCAAATGAAAAGACATTCATGGGAGACGTCTTAAGTGGTGGAGAATGTGAGAAT
AGTCTCTCAAGAGAAGATTTATTTCATATAGAAGTAGGATCCGAAGAATCGCTAGATGAT
GCCTCAAAATATAATTTCCAAAAGGATTTATCAACTAGCGATAATAGTTCATTCGAAGAT
GATCAGTCATTGAAAAGGGGACTCAAAAGGAACAGCTCAGTATCTAGTCTGGACAGCGAT
ATGGGAAGTTATAAAAATAAAAGTTATAGACACAGATTGGATATATATTCGGACCTACCT
AGAAGGCCAATACATGGGGATAATGCACCACAAGAAAAGGAGAAATGTCTGGATTTCAAA
GAACTTCTAGAGAGAGAAAGGAACAACCACCAAAGGAATAGTGTAAATATAAAATTAAAA
AATATATACGGAGAAGAACAATTGTATAATGTGAAGAAAGATAGCTTTATTGTAAAATTG
ATATACTATCTGCCGTTTGGCCCAATTGTTACAATGATAATGATAGGCTTTATTCTACTC

TGGTCAACGGGCTGGACTGGTTTTATTTTTATAGGGTGTAGCTCTATTATATTACCAGCT
TTGTTATACGCGATGTATTCAAACAAAAAACAATTCGAACGTGTATTTGGAAAAGGGAGA
AAAAAGCCAATGAAGGTAAAAAAAGGAGGCGGGAAAATATCTAGATTTTTTAAAGAAGTA
GACAAA---------------------------
>BO951 .
------------------------------TTAAGGAAAATTTCCCTGTGTTCTATTTTC
GTATTGGCATATCTCAGTTCTGCTAAGTGCGCGTACCGAGACGAATCTGACATATTGTAC
AGCGACGACTCCAGATCCGATTTAGCAAGCAACGGGGCACCCACCGACAGCTACGAATCT
TTAACAGCAAGTAGTGAGTCTCTAGCAGAAAGCAACGATGCACCCAGCAACAGCTATGAA
TCTTTTCCAGAAATTAGAGAAAATCTAACCGCAAGTGAGGAATCCCTAACATCATGTGAG
GAATCCCTAACAGGAAGTAATGAATCCCTAACAGGAAGTAATGAATCCCTAACAGGAAGT
AAT------------------------------------------GAATCCCTAACAGGA
AGTAATGAATCCCTAACAGGAAGTAATGAATCCCTAACAGGAAGTAATGAATCCCTAACA
GAAAGTAGAGAATCTCTAGAGGCCAGTAGAGAATCGCTAAGAGCAAGTAGAGAGTCTCTA
GCGGCCAGTAGAGAATCCCTGAACGACTTTTGTGGGAGCGAAGAATCAGTAGCATTCGAA
GGAGAGCCAAATGAAAAGACATTCATGGGAGACGTCTTAAGTGGTGGAGAATGTGAGAAT
AGTCTCTCAAGAGAAGATTTATTTCATATAGAAGTAGGATCCGAAGAATCGCTAGATGAT
GCCTCAAAATATAATTTCCAAAAGGATTTATCAACTAGCGATAATAGTTCATTCGAAGAT
GATCAGTCATTGAAAAGGGGACTCAAAAGGAACAGCTCAGTATCTAGTCTGGACAGCGAT
ATGGGAAGTTATAAAAATGGAAGATCTAGAGACAGATTGGATATATATTCGGACCTACCT
AGAAGGCTAATACATGGGGATAATGCACCACAAGAAAAGGAGAAATGTCTGGATTTCAAA
GAACTTCTAGAGAGAGAAAGGAACAACCACCAAAGGAATAGTGTAAATATAAAATTAAAA
AATATATACGGAGAAGAACAATTGTATAATGTGAAGAAAGATAGCTTTATTGTAAAATTG
ATATACTATCTGCCGTTTGGCCCAATTGTTACAATGATAATGATAGGTTTTATTCTACTC
TGGTCAACGCCCTGGACTGGTTTTATTTTTATAGGGTGTAGCTCTATTATATTACCAGCT
TTGTTATACGCGATGCATTCAAACAAAAAACAATTCGAACGTGTATTTGGAAAAGGGAGA
AAAAAGCCAATGAAGGTAAAAAAAGGAGGCGGGAAAATATCTAGATTTTTTAAAGAAGTA
GACAAA---------------------------
>BO952 .
------------------------------TTAAGGAAAATTTCCCTGTGTTCTATTTTC
GTATTGGCATATCTCAGTTCTGCTAAGTGCGCGTACCGAGACGAATCTGACATATTGTAC
AGCGACGACTCCAGATCCGATTTAGCAAGCAACGGGGCACCCACCGACAGCTACGAATCT
TTAACAGCAAGTAGTGAGTCTCTAGCAGAAAGCAACGATGCACCCAGCAACAGCTATGAA
TCTTTTCCAGAAATTAGAGAAAATCTAACCGCAAGTGAGGAATCCCTAACATCATGTGAG
GAATCCCTAACAGGAAGTAATGAATCCCTAACAGGAAGTAATGAATCCCTAACAGGAAGT
AAT---------------------------------------------------------
------------------------------------------------GAATCCCTAACA
GAAAGTAGAGAATCTCTAGAGGCCAGTAGAGAATCGCTAAGAGCAAGTAGAGAGTCTCTA
GCGGCCAGTAGAGAATCCCTGAACGACTTTTGTGGGAGCGAAGAATCAGTAGCATTCGAA
GGAGAGCCAAATGAAAAGACATTCATGGGAGACGTCTTAAGTGGTGGAGAATGTGAGAAT
AGTCTCTCAAGAGAAGATTTATTTCATATAGAAGTAGGATCCGAAGAATCGCTAGATGAT
GCCTCAAAATATAATTTCCAAAAGGATTTATCAACTAGCGATAATAGTTCATTCGAAGAT
GATCAGTCATTGAAAAGGGGACTCAAAAGGAACAGCTCAGAATCTAGTCTGGACAGCGAT
ATGGGAAGTTATAAAAATAAAAGTTATAGACACAGATTGGATATATATTCGGACCTACCT
AGAAGGCCAATACATGGGGATAATGCACCACAAGAAAAGGAGAAATGTCTGGATTTCAAA
GAACTTCTAGAGAGAGAAAGGAACAACCACCAAAGGAATAGTGTAAATATAAAATTAAAA
AATATATACGGAGAAGAACAATTGTATAATGTGAAGAAAGATAGCTTTATTGTAAAATTG
ATATACTATCTGCCGTTTGGCCCAATTGTTACAATGATAATGATAGGCTTTATTCTACTC
TGCTGGACCCCCTCGATTGGTTTTACTTTTATAGGGTGTAGCTCTATTATATTACCAGCT
TTGTTATACGCGATGTATTCAAACAAAAAACAATTCGAACGTGTATTTGGAAAAGGGAGA

AAAAAGCCAATGAAGGTAAAAAAAGGAGGCGGGAAAATATCTAGATTTTTTAAGAAAGTA
GACAAA---------------------------
>BO2021_R .
------------------------------TTAAGGAAAATTTCCCTGTGTTCTATTTTC
GTATTGGCATATCTCAGTTCTGCTAAGTGCGCGTACCGAGACGAATCTGACATATTGTAC
AGCGACGACTCCAGATCCGATTTAGCAAGCAACGGGGCACCCACCGACAGCTACGAATCT
TTAACAGCAAGTAGTGAGTCTCTAGCAGAAAGCAACGATGCACCCAGCAACAGCTATGAA
TCTTTTCCAGAAATTAGAGAAAATCTAACCGCAAGTGAGGAATCCCTAACATCATGTGAG
GAATCCCTAACAGGAAGTAATGAATCCCTAACAGGAAGTAATGAATCCCTAACAGGAAGT
AAT---------------------------------------------------------
------------------------------------------------GAATCCCTAACA
GAAAGTAGAGAATCTCTAGAGGCCAGTAGAGAATCGCTAAGAGCAAGTAGAGAGTCTCTA
GCGGCCAGTAGAGAATCCCTGAACGACTTTTGTGGGAGCGAAGAATCAGTAGCATTCGAA
GGAGAGCCAAATGAAAAGACATTCATGGGAGACGTCTTAAGTGGTGGAGAATGTGAGAAT
AGTCTCTCAAGAGAAGATTTATTTCATATAGAAGTAGGATCCGAAGAATCGCTAGATGAT
GCCTCAAAATATAATTTCCAAAAGGATTTATCAACTAGCGATAATAGTTCATTCGAAGAT
GATCAGTCATTGAAAAGGGGACTCAAAAGGAACAGCTCAGAATCTAGTCTGGACAGCGAT
ATGGGAAGTTATAAAAATAAAAGTTATAGACACAGATTGGATATATATTCGGACCTACCT
AGAAGGCCAATACATGGGGATAATGCACCACAAGAAAAGGAGAAATGTCTGGATTTCAAA
GAACTTCTAGAGAGAGAAAGGAACAACCACCAAAGGAATAGTGTAAATATAAAATTAAAA
AATATATACGGAGAAGAACAATTGTATAATGTGAAGAAAGATAGCTTTATTGTAAAATTG
ATATACTATCTGCCGTTTGGCCCAATTGTTACAATGATAATGATAGGCTTTATTCTACTC
TGCTGGACCCCCTCGATTGGTTTTACTTTTATAGGGTGTAGCTCTATTATATTACCAGCT
TTGTTATACGCGATGTATTCAAACAAAAAACAATTCGAACGTGTATTTGGAAAAGGGAGA
AAAAAGCCAATGAAGGTAAAAAAAGGAGGCGGGAAAATATCTAGATTTTTTAAAGAAGTA
GACAAA---------------------------
>BO2111 .
------------------------------TTAAGGAAAATTTCCCTGTGTTCTATTTTC
GTATTGGCATATCTCAGTTCTGCTAAGTGCGCGTACCGAGACGAATCTGACATATTGTAC
AGCGACGACTCCAGATCCGATTTAGCAAGCAACGGGGCACCCACCGACAGCTACGAATCT
TTAACAGCAAGTAGTGAGTCTCTAGCAGAAAGCAACGATGCACCCAGCAACAGCTATGAA
TCTTTTCCAGAAATTAGAGAAAATCTAACCGCAAGTGAGGAATCCCTAACATCATGTGAG
GAATCCCTAACAGGAAGTAATGAATCCCTAACAGGAAGTAATGAATCCCTAACAGGAAGT
AAT---------------------------------------------------------
------------------------------------------------GAATCCCTAACA
GAAAGTAGAGAATCTCTAGAGGCCAGTAGAGAATCGCTAAGAGCAAGTAGAGAGTCTCTA
GCGGCCAGTAGAGAATCCCTGAACGACTTTTGTGGGAGCGAAGAATCAGTAGCATTCGAA
GGAGAGCCAAATGAAAAGACATTCATGGGAGACGTCTTAAGTGGTGGAGAATGTGAGAAT
AGTCTCTCAAGAGAAGATTTATTTCATATAGAAGTAGGATCCGAAGAATCGCTAGATGAT
GCCTCAAAATATAATTTCCAAAAGGATTTATCAACTAGCGATAATAGTTCATTCGAAGAT
GATCAGTCATTGAAAAGGGGACTCAAAAGGAACAGCTCAGAATCTAGTCTGGACAGCGAT
ATGGGAAGTTATAAAAATAAAAGTTATAGACACAGATTGGATATATATTCGGACCTACCT
AGAAGGCCAATACATGGGGATAATGCACCACAAGAAAAGGAGAAATGTCTGGATTTCAAA
GAACTTCTAGAGAGAGAAAGGAACAACCACCAAAGGAATAGTGTAAATATAAAATTAAAA
AATATATACGGAGAAGAACAATTGTATAATGTGAAGAAAGATAGCTTTATTGTAAAATTG
ATATACTATCTGCCGTTTGGCCCAATTGTTACAATGATAATGATAGGCTTTATTCTACTC

TGCTGGACCCCCTCGATTGGTTTTACTTTTATAGGGTGTAGCTCTATTATATTACCAGCT
TTGTTATACGCGATGTATTCAAACAAAAAACAATTCGAACGTGTATTTGGAAAAGGGAGA
AAAAAGCCAATGAAGGTAAAAAAAGGAGGCGGGAAAATATCTAGATTTTTTAAAGAAGTA
GACAAA---------------------------
>BO2821 .
------------------------------TTAAGGAAAATTTCCCTGTGTTCTATTTTC
GTATTGGCATATCTCAGTTCTGCTAAGTGCGCGTACCGAGACGAATCTGACATATTGTAC
AGCGACGACTCCAGATCCGATTTAGCAAGCAACGGGGCACCCACCGACAGCTACGAATCT
TTAACAGCAAGTAGTGAGTCTCTAGCAGAAAGCAACGATGCACCCAGCAACAGCTATGAA
TCTTTTCCAGAAATTAGAGAAAATCTAACCGCAAGTGAGGAATCCCTAACATCATGTGAG
GAATCCCTAACAGGAAGTAATGAATCCCTAACAGGAAGTAATGAATCCCTAACAGGAAGT
AAT---------------------------------------------------------
---------------------------GAATCCCTAACAGGAAGTAATGAATCCCTAACA
GAAAGTAGAGAATCTCTAGAGGCCAGTAGAGAATCGCTAAGAGCAAGTAGAGAGTCTCTA
GCGGCCAGTAGAGAATCCCTGAACGACTTTTGTGGGAGCGAAGAATCAGTAGCATGCGAA
GGAGAGCCAAATGAAAAGACATTCATGGGAGACGTCTTAAGTGGTGGAGAATGTGAGAAT
AGTCTCTCAAGAGAAGATTTATTTCATATAGAAGTAGGATCCGAAGAATCGCTAGATGAT
GCCTCAAAATATAATTTCCAAAAGGATTTATCAACTAGCGATAATAGTTCATTCGAAGAT
GATCAGTCATTGAAAAGGGGACTCAAAAGGAACAGCTCAGTATCTAGTCTGGACAGCGAT
ATGGGAAGTTATAAAAATGGAAGATCTAGAGACAGATTGGATATATATTCGGACCTACCT
AGAAGGCTAATACATGGGGATAATGCACCACAAGAAAAGGAGAAATGTCTGGATTTCAAA
GAACTTCTAGAGAGAGAAAGGAACAACCACCAAAGGAATAGTGTAAATATAAAATTAAAA
AATATATACGGAGAAGAACAATTGTATAATGTGAAGAAAGATAGCTTTATTGTAAAATTG
ATATACTATCTGCCGTTTGGCCCAATTGTTACAATGATAATGATAGGCTTTATTCTACTC
TGCTCAACGCCCTCGACTGGTTTTATTTTTATAGGGTGTAGCTCTATTATATTACCAGCT
TTGTTATACGCGATGCATTCAAACAAAAAACAATTCGAACGTGTATTTGGAAAAGGGAGA
AAAAAGCCAATGAAGGTAAAAAAAGGAGGCGGGAAAATATCTAGATTTTTTAAAGAAGTA
GACAAA---------------------------
>VC673 .
------------------------------TTAAGGAAAATTTCCCTGTGTTCTATTTTC
GTATTGGCATATCTCAGTTCTGCTAAGTGCGCGTACCGAGACGAATCTGACATATTGTAC
AGCGACGACTCCAGATCCGATTTAGCAAGCAACGGGGCACCCACCGACAGCTACGAATCT
TTAACAGCAAGTAGTGAGTCTCTAGCAGAAAGCAACGATGCACCCAGCAACAGCTATGAA
TCTTTTCCAGAAATTAGAGAAAATCTAACCGCAAGTGAGGAATCCCTAACATCATGTGAG
GAATCCCTAACAGGAAGTAATGAATCCCTAACAGGAAGTAATGAATCCCTAACAGGAAGT
AAT---------------------------------------------------------
---------------------------GAATCCCTAACAGGAAGTAATGAATCCCTAACA
GAAAGTAGAGAATCTCTAGAGGCCAGTAGAGAATCGCTAAGAGCAAGTAGAGAGTCTCTA
GCGGCCAGTAGAGAATCCCTGAACGACTTTTGTGGGAGCGAAGAATCAGTAGCATGCGAA
GGAGAGCCAAATGAAAAGACATTCATGGGAGACGTCTTAAGTGGTGGAGAATGTGAGAAT
AGTCTCTCAAGAGAAGATTTATTTCATATAGAAGTAGGATCCGAAGAATCGCTAGATGAT
GCCTCAAAATATAATTTCCAAAAGGATTTATCAACTAGCGATAATAGTTCATTCGAAGAT
GATCAGTCATTGAAAAGGGGACTCAAAAGGAACAGCTCAGTATCTAGTCTGGACAGCGAT
ATGGGAAGTTATAAAAATGGAAGATCTAGAGACAGATTGGATATATATTCGGACCTACCT
AGAAGGCTAATACATGGGGATAATGCACCACAAGAAAAGGAGAAATGTCTGGATTTCAAA
GAACTTCTAGAGAGAGAAAGGAACAACCACCAAAGGAATAGTGTAAATATAAAATTAAAA
AATATATACGGAGAAGAACAATTGTATAATGTGAAGAAAGATAGCTTTATTGTAAAATTG
ATATACTATCTGCCGTTTGGCCCAATTGTTACAATGATAATGATAGGCTTTATTCTACTC
TGCTGGACCCCCTCGATTGGTTTTACTTTTATAGGGTGTAGCTCTATTATATTACCAGCT
TTGTTATACGCGATGTATTCAAACAAAAAACAATTCGAACGTGTATTTGGAAAAGGGAGA

AAAAAGCCAATGAAGGTAAAAAAAGGAGGCGGGAAAATATCTAGATTTTTTAAAGAAGTA
GACAAA---------------------------
>VC683 .
------------------------------TTAAGGAAAATTTCCCTGTGTTCTATTTTC
GTATTGGCATATCTCAGTTCTGCTAAGTGCGCGTACCGAGACGAATCTGACATATTGTAC
AGCGACGACTCCAGATCCGATTTAGCAAGCAACGGGGCACCCACCGACAGCTACGAATCT
TTAACAGCAAGTAGTGAGTCTCTAGCAGAAAGCAACGATGCACCCAGCAACAGCTATGAA
TCTTTTCCAGAAATTAGAGAAAATCTAACCGCAAGTGAGGAATCCCTAACATCATGTGAG
GAATCCCTAACAGGAAGTAACGAATCCCTAACAGGAAGTAATGAATCCCTAACAGGAAGT
AAT---------------------------------------------------------
---------------------------GAATCCCTAACAGGAAGTAATGAATCCCTAACA
GAAAGTAGAGAATCTCTAGAGGCCAGTAGAGAATCGCTAAGAGCAAGTAGAGAGTCTCTA
GCGGCCAGTAGAGAATCCCTGAACGACTTTTGTGGGAGCGAAGAATCAGTAGCATTCGAA
GGAGAGCCAAATGAAAAGACATTCATGGGAGACGTCTTAAGTGGTGGAGAATGTGAGAAT
AGTCTCTCAAGAGAAGATTTATTTCATATAGAAGTAGGATCCGAAGAATCGCTAGATGAT
GCCTCAAAATATAATTTCCAAAAGGATTTATCAACTAGCGATAATAGTTTATTCGAAGAT
GATCAGTCATTGAAAAGGGGACTCAAAAGGAACAGCTCAGTATCTAGTCTGGACAGCGAT
ATGGGAAGTTATAAAAATGGAAGATCTAGAGACAGATTGGATATATATTCGGACCTACCT
AGAAGGCTAATACATGGGGATAATGCACCACAAGAAAAGGAGAAATGTCTGGATTTCAAA
GAACTTCTAGAGAGAGAAAGGAACAACCACCAAAGGAATAGTGTAAATATAAAATTAAAA
AATATATACGGAGAAGAACAATTGTATAATGTGAAGAAAGATAGCTTTATTGTAAAATTG
ATATACTATCTGCCGTTTGGCCCAATTGTTACAATGATAATGATAGGTTTTATTCTACTC
TGGTCAACGCCCTGGACTGGTTTTATTTTTATAGGGTGTAGCTCTATTATATTACCAGCT
TTGTTATACGCGATGCATTCAAACAAAAAACAATTCGAACGTGTATTTGGAAAAGGGAGA
AAAAAGCCAATGAAGGTAAAAAAAGGAGGCGGGAAAATATCTAGATTTTTTAAAGAAGTA
GACAAA---------------------------
>VC794 .
------------------------------TTAAGGAAAATTTCCCTGTGTTCTATTTTC
GTATTGGCATATCTCAGTTCTGCTAAGTGCGCGTACCGAGACGAATCTGACATATTGTAC
AGCGACGACTCCAGATCCGATTTAGCAAGCAACGGGGCACCCACCGACAGCTACGAATCT
TTAACAGCAAGTAGTGAGTCTCTAGCAGAAAGCAACGATGCACCCAGCAACAGCTATGAA
TCTTTTCCAGAAATTAGAGAAAATCTAACCGCAAGTGAGGAATCCCTAACATCATGTGAG
GAATCCCTAACAGGAAGTAATGAATCCCTAACAGGAAGTAATGAATCCCTAACAGGAAGT
AAT---------------------------------------------------------
---------------------------GAATCCCTAACAGGAAGTAATGAATCCCTAACA
GAAAGTAGAGAATCTCTAGAGGCCAGTAGAGAATCGCTAAGAGCAAGTAGAGAGTCTCTA
GCGGCCAGTAGAGAATCCCTGAACGACTTTTGTGGGAGCGAAGAATCAGTAGCATGCGAA
GGAGAGCCAAATGAAAAGACATTCATGGGAGACGTCTTAAGTGGTGGAGAATGTGAGAAT
AGTCTCTCAAGAGAAGATTTATTTCATATAGAAGTAGGATCCGAAGAATCGCTAGATGAT
GCCTCAAAATATAATTTCCAAAAGGATTTATCAACTAGCGATAATAGTTCATTCGAAGAT
GATCAGTCATTGAAAAGGGGACTCAAAAGGAACAGCTCAGTATCTAGTCTGGACAGCGAT
ATGGGAAGTTATAAAAATGGAAGATCTAGAGACAGATTGGATATATATTCGGACCTACCT
AGAAGGCTAATACATGGGGATAATGCACCACAAGAAAAGGAGAAATGTCTGGATTTCAAA
GAACTTCTAGAGAGAGAAAGGAACAACCACCAAAGGAATAGTGTAAATATAAAATTAAAA
AATATATACGGAGAAGAACAATTGTATAATGTGAAGAAAGATAGCTTTATTGTAAAATTG
ATATACTATCTGCCGTTTGGCCCAATTGTTACAATGATAATGATAGGCTTTATTCTACTC

TGGTCAACGGGCTGGACTGGTTTTATTTTTATAGGGTGTAGCTCTATTATATTACCAGCT
TTGTTATACGCGATGCATTCAAACAAAAAACAATTCGAACGTGTATTTGGAAAAGGGAGA
AAAAAGCCAATGAAGGTAAAAAAAGGAGGCGGGAAAATATCTAGATTTTTTAAAGAAGTA
GACAAA---------------------------
>VC803 .
------------------------------TTAAGGAAAATTTCCCTGTGTTCTATTTTC
GTATTGGCATATCTCAGTTCTGCTAAGTGCGCGTACCGAGACGAATCTGACATATTGTAC
AGCGACGACTCCAGATCCGATTTAGCAAGCAACGGGGCACCCACCGACAGCTACGAATCT
TTAACAGCAAGTAGTGAGTCTCTAGCAGAAAGCAACGATGCACCCAGCAACAGCTATGAA
TCTTTTCCAGAAATTAGAGAAAATCTAACCGCAAGTGAGGAATCCCTAACATCATGTGAG
GAATCCCTAACAGGAAGTAATGAATCCCTAACAGGAAGTAATGAATCCCTAACAGGAAGT
AAT------------------------------------------GAATCCCTAACAGGA
AGTAATGAATCCCTAACAGGAAGTAATGAATCCCTAACAGGAAGTAATGAATCCCTAACA
GAAAGTAGAGAATCTCTAGAGGCCAGTAGAGAATCGCTAAGAGCAAGTAGAGAGTCTCTA
GCGGCCAGTAGAGAATCCCTGAACGACTTTTGTGGGAGCGAAGAATCAGTAGCATTCGAA
GGAGAGCCAAATGAAAAGACATTCATGGGAGACGTCTTAAGTGGTGGAGAATGTGAGAAT
AGTCTCTCAAGAGAAGATTTATTTCATATAGAAGTAGGATCCGAAGAATCGCTAGATGAT
GCCTCAAAATATAATTTCCAAAAGGATTTATCAACTAGCGATAATAGTTTATTCGAAGAT
GATCAGTCATTGAAAAGGGGACTCAAAAGGAACAGCTCAGTATCTAGTCTGGACAGCGAT
ATGGGAAGTTATAAAAATGGAAGATCTAGAGACAGATTGGATATATATTCGGACCTACCT
AGAAGGCTAATACATGGGGATAATGCACCACAAGAAAAGGAGAAATGTCTGGATTTCAAA
GAACTTCTAGAGAGAGAAAGGAACAACCACCAAAGGAATAGTGTAAATATAAAATTAAAA
AATATATACGGAGAAGAACAATTGTATAATGTGAAGAAAGATAGCTTTATTGTAAAATTG
ATATACTATCTGCCGTTTGGCCCAATTGTTACAATGATAATGATAGGTTTTATTCTACTC
TGGTCAACGCCCTGGACTGGTTTTATTTTTATAGGGTGTAGCTCTATTATATTACCAGCT
TTGTTATACGCGATGCATTCAAACAAAAAACAATTCGAACGTGTATTTGGAAAAGGGAGA
AAAAAGCCAATGAAGGTAAAAAAAGGAGGCGGGAAAATATCTAGATTTTTTAAAGAAGTA
GACAAA---------------------------
>VC862 .
------------------------------TTAAGGAAAATTTCCCTGTGTTCTATTTTC
GTATTGGCATATCTCAGTTCTGCTAAGTGCGCGTACCGAGACGAATCTGACATATTGTAC
AGCGACGACTCCAGATCCGATTTAGCAAGCAACGGGGCACCCACCGACAGCTACGAATCT
TTAACAGCAAGTAGTGAGTCTCTAGCAGAAAGCAACGATGCACCCAGCAACAGCTATGAA
TCTTTTCCAGAAATTAGAGAAAATCTAACCGCAAGTGAGGAATCCCTAACATCATGTGAG
GAATCCCTAACAGGAAGTAATGAATCCCTAACAGGAAGTAATGAATCCCTAACAGGAAGT
AAT---------------------------------------------------------
---------------------------GAATCCCTAACAGGAAGTAATGAATCCCTAACA
GAAAGTAGAGAATCTCTAGAGGCCAGTAGAGAATCGCTAAGAGCAAGTAGAGAGTCTCTA
GCGGCCAGTAGAGAATCCCTGAACGACTTTTGTGGGAGCGAAGAATCAGTAGCATTCGAA
GGAGAGCCAAATGAAAAGACATTCATGGGAGACGTCTTAAGTGGTGGAGAATGTGAGAAT
AGTCTCTCAAGAGAAGATTTATTTCATATAGAAGTAGGATCCGAAGAATCGCTAGATGAT
GCCTCAAAATATAATTTCCAAAAGGATTTATCAACTAGCGATAATAGTTCATTCGAAGAT
GATCAGTCATTGAAAAGGGGACTCAAAAGGAACAGCTCAGTATCTAGTCTGGACAGCGAT
ATGGGAAGTTATAAAAATAAAAGTTATAGACACAGATTGGATATATATTCGGACCTACCT
AGAAGGCCAATACATGGGGATAATGCACCACAAGAAAAGGAGAAATGTCTGGATTTCAAA
GAACTTCTAGAGAGAGAAAGGAACAACCACCAAAGGAATAGTGTAAATATAAAATTAAAA
AATATATACGGAGAAGAACAATTGTATAATGTGAAGAAAGATAGCTTTATTGTAAAATTG
ATATACTATCTGCCGTTTGGCCCAATTGTTACAATGATAATGATAGGCTTTATTCTACTC
TGCTCAACGCCCTCGACTGGTTTTATTTTTATAGGGTGTAGCTCTATTATATTACCAGCT
TTGTTATACGCGATGCATTCAAACAAAAAACAATTCGAACGTGTATTTGGAAAAGGGAGA

AAAAAGCCAATGAAGGTAAAAAAAGGAGGCGGGAAAATATCTAGATTTTTTAAAGAAGTA
GACAAA---------------------------
>VC894 .
------------------------------TTAAGGAAAATTTCCCTGTGTTCTATTTTC
GTATTGGCATATCTCAGTTCTGCTAAGTGCGCGTACCGAGACGAATCTGACATATTGTAC
AGCGACGACTCCAGATCCGATTTAGCAAGCAACGGGGCACCCACCGACAGCTACGAATCT
TTAACAGCAAGTAGTGAGTCTCTAGCAGAAAGCAACGATGCACCCAGCAACAGCTATGAA
TCTTTTCCAGAAATTAGAGAAAATCTAACCGCAAGTGAGGAATCCCTAACATCATGTGAG
GAATCCCTAACAGGAAGTAATGAATCCCTAACAGGAAGTAATGAATCCCTAACAGGAAGT
AAT---------------------------------------------------------
---------------------------GAATCCCTAACAGGAAGTAATGAATCCCTAACA
GAAAGTAGAGAATCTCTAGAGGCCAGTAGAGAATCGCTAAGAGCAAGTAGAGAGTCTCTA
GCGGCCAGTAGAGAATCCCTGAACGACTTTTGTGGGAGCGAAGAATCAGTAGCATGCGAA
GGAGAGCCAAATGAAAAGACATTCATGGGAGACGTCTTAAGTGGTGGAGAATGTGAGAAT
AGTCTCTCAAGAGAAGATTTATTTCATATAGAAGTAGGATCCGAAGAATCGCTAGATGAT
GCCTCAAAATATAATTTCCAAAAGGATTTATCAACTAGCGATAATAGTTCATTCGAAGAT
GATCAGTCATTGAAAAGGGGACTCAAAAGGAACAGCTCAGTATCTAGTCTGGACAGCGAT
ATGGGAAGTTATAAAAATGAAAGTTATAGAGACAGATTGGATATATATTCGGACCTACCT
AGAAGGCCAATACATGGGGATAATGCACCACAAGAAAAGGAGAAATGTCTGGATTTCAAA
GAACTTCTAGAGAGAGAAAGGAACAACCACCAAAGGAATAGTGTAAATATAAAATTAAAA
AATATATACGGAGAAGAACAATTGTATAATGTGAAGAAAGATAGCTTTATTGTAAAATTG
ATATACTATCTGCCGTTTGGCCCAATTGTTACAATGATAATTATAGGCTTTATTCTACTT
TGGTCAACGCCCTGGACTGGTTTTATTTTTATAGGGTGTAGCTTTATTATATTACCAGCT
TTGTTATACGCGATGCATTCAAACAAAAAACAATTCGAACGTGTATTTGGAAAAGGGAGA
AAAAAGCCAATGAAGGTAAAAAAAGGAGGCGGGAAAATATCTAGATTTTTTAAAGAAGTA
GACAAA---------------------------
>VC898 .
------------------------------TTAAGGAAAATTTCCCTGTGTTCTATTTTC
GTATTGGCATATCTCAGTTCTGCTAAGTGCGCGTACCGAGACGAATCTGACATATTGTAC
AGCGACGACTCCAGATCCGATTTAGCAAGCAACGGGGCACCCACCGACAGCTACGAATCT
TTAACAGCAAGTAGTGAGTCTCTAGCAGAAAGCAACGATGCACCCAGCAACAGCTATGAA
TCTTTTCCAGAAATTAGAGAAAATCTAACCGCAAGTGAGGAATCCCTAACATCATGTGAG
GAATCCCTAACAGGAAGTAATGAATCCCTAACAGGAAGTAATGAATCCCTAACAGGAAGT
AAT---------------------------------------------------------
-------------------------------------------------GAATCCCTAACA
GAAAGTAGAGAATCTCTAGAGGCCAGTAGAGAATCGCTAAGAGCAAGTAGAGAGTCTCTA
GCGGCCAGTAGAGAATCCCTGAACGACTTTTGTGGGAGCGAAGAATCAGTAGCATTCGAA
GGAGAGCCAAATGAAAAGACATTCATGGGAGACGTCTTAAGTGGTGGAGAATGTGAGAAT
AGTCTCTCAAGAGAAGATTTATTTCATATAGAAGTAGGATCCGAAGAATCGCTAGATGAT
GCCTCAAAATATAATTTCCAAAAGGATTTATCAACTAGCGATAATAGTTCATTCGAAGAT
GATCAGTCATTGAAAAGGGGACTCAAAAGGAACAGCTCAGAATCTAGTCTGGACAGCGAT
ATGGGAAGTTATAAAAATAAAAGTTATAGACACAGATTGGATATATATTCGGACCTACCT
AGAAGGCCAATACATGGGGATAATGCACCACAAGAAAAGGAGAAATGTCTGGATTTCAAA
GAACTTCTAGAGAGAGAAAGGAACAACCACCAAAGGAATAGTGTAAATATAAAATTAAAA
AATATATACGGAGAAGAACAATTGTATAATGTGAAGAAAGATAGCTTTATTGTAAAATTG
ATATACTATCTGCCGTTTGGCCCAATTGTTACAATGATAATGATAGGCTTTATTCTACTC

TGCTGGACCCCCTCGATTGGTTTTACTTTTATAGGGTGTAGCTCTATTATATTACCAGCT
TTGTTATACGCGATGTATTCAAACAAAAAACAATTCGAACGTGTATTTGGAAAAGGGAGA
AAAAAGCCAATGAAGGTAAAAAAAGGAGGCGGGAAAATATCTAGATTTTTTAAAGAAGTA
GACAAA---------------------------
>VC900 .
------------------------------TTAAGGAAAATTTCCCTGTGTTCTATTTTC
GTATTGGCATATCTCAGTTCTGCTAAGTGCGCGTACCGAGACGAATCTGACATATTGTAC
AGCGACGACTCCAGATCCGATTTAGCAAGCAACGGGGCACCCACCGACAGCTACGAATCT
TTAACAGCAAGTAGTGAGTCTCTAGCAGAAAGCAACGATGCACCCAGCAACAGCTATGAA
TCTTTTCCAGAAATTAGAGAAAATCTAACCGCAAGTGAGGAATCCCTAACATCATGTGAG
GAATCCCTAACAGGAAGTAATGAATCCCTAACAGGAAGTAATGAATCCCTAACAGGAAGT
AAT---------------------------------------------------------
---------------------------GAATCCCTAACAGGAAGTAATGAATCCCTAACA
GAAAGTAGAGAATCTCTAGAGGCCAGTAGAGAATCGCTAAGAGCAAGTAGAGAGTCTCTA
GCGGCCAGTAGAGAATCCCTGAACGACTTTTGTGGGAGCGAAGAATCAGTAGCATGCGAA
GGAGAGCCAAATGAAAAGACATTCATGGGAGACGTCTTAAGTGGTGGAGAATGTGAGAAT
AGTCTCTCAAGAGAAGATTTATTTCATATAGAAGTAGGATCCGAAGAATCGCTAGATGAT
GCCTCAAAATATAATTTCCAAAAGGATTTATCAACTAGCGATAATAGTTCATTCGAAGAT
GATCAGTCATTGAAAAGGGGACTCAAAAGGAACAGCTCAGTATCTAGTCTGGACAGCGAT
ATGGGAAGTTATAAAAATGGAAGATCTAGAGACAGATTGGATATATATTCGGACCTACCT
AGAAGGCTAATACATGGGGATAATGCACCACAAGAAAAGGAGAAATGTCTGGATTTCAAA
GAACTTCTAGAGAGAGAAAGGAACAACCACCAAAGGAATAGTGTAAATATAAAATTAAAA
AATATATACGGAGAAGAACAATTGTATAATGTGAAGAAAGATAGCTTTATTGTAAAATTG
ATATACTATCTGCCGTTTGGCCCAATTGTTACAATGATAATGATAGGCTTTATTCTACTC
TGCTGGACCCCCTCGATTGGTTTTACTTTTATAGGGTGTAGCTCTATTATATTACCAGCT
TTGTTATACGCGATGTATTCAAACAAAAAACAATTCGAACGTGTATTTGGAAAAGGGAGA
AAAAAGCCAATGAAGGTAAAAAAAGGAGGCGGGAAAATATCTAGATTTTTTAAAGAAGTA
GACAAA---------------------------
>VC916 .
------------------------------TTAAGGAAAATTTCCCTGTGTTCTATTTTC
GTATTGGCATATCTCAGTTCTGCTAAGTGCGCGTACCGAGACGAATCTGACATATTGTAC
AGCGACGACTCCAGATCCGATTTAGCAAGCAACGGGGCACCCACCGACAGCTACGAATCT
TTAACAGCAAGTAGTGAGTCTCTAGCAGAAAGCAACGATGCACCCAGCAACAGCTATGAA
TCTTTTCCAGAAATTAGAGAAAATCTAACCGCAAGTGAGGAATCCCTAACATCATGTGAG
GAATCCCTAACAGGAAGTAATGAATCCCTAACAGGAAGTAATGAATCCCTAACAGAAAGT
AATGAATCCCTAACAGGAAGTAATGAATCCCTAACAGGAAGTAATGAATCCCTAACAGGA
AGTAATGAATCCCTAACAGGAAGTAATGAATCCCTAACAGGAAGTAATGAATCCCTAACA
GAAAGTAGAGAATCTCTAGAGGCCAGTAGAGAATCGCTAAGAGCAAGTAGAGAGTCTCTA
GCGGCCAGTAGAGAATCCCTGAACGACTTTTGTGGGAGCGAAGAATCAGTAGCATTCGAA
GGAGAGCCAAATGAAAAGACATTCATGGGAGACGTCTTAAGTGGTGGAGAATGTGAGAAT
AGTCTCTCAAGAGAAGATTTATTTCATATAGAAGTAGGATCCGAAGAATCGCTAGATGAT
GCCTCAAAATATAATTTCCAAAAGGATTTATCAACTAGCGATAATAGTTTATTCGAAGAT
GATCAGTCATTGAAAAGGGGACTCAAAAGGAACAGCTCAGTATCTAGTCTGGACAGCGAT
ATGGGAAGTTATAAAAATGGAAGATCTAGAGACAGATTGGATATATATTCGGACCTACCT
AGAAGGCTAATACATGGGGATAATGCACCACAAGAAAAGGAGAAATGTCTGGATTTCAAA
GAACTTCTAGAGAGAGAAAGGAACAACCACCAAAGGAATAGTGTAAATATAAAATTAAAA
AATATATACGGAGAAGAACAATTGTATAATGTGAAGAAAGATAGCTTTATTGTAAAATTG
ATATACTATCTGCCGTTTGGCCCAATTGTTACAATGATAATGATAGGTTTTTTTCTACTC
TGCTGGACCCCCTCGATTGGTTTTATTTTTATAGGGTGTAGCTCTATTATATTACCAGCT
TTGTTATACGCGATGCATTCAAACAAAAAACAATTCGAACGTGTATTTGGAAAAGGGAGA

AAAAAGCCAATGAAGGTAAAAAAAGGAGGCGGGAAAATATCTAGATTTTTTAAAGAAGTA
GACAAA--------------------------
>VC917_R .
------------------------------TTAAGGAAAATTTCCCTGTGTTCTATTTTC
GTATTGGCATATCTCAGTTCTGCTAAGTGCGCGTACCGAGACGAATCTGACATATTGTAC
AGCGACGACTCCAGATCCGATTTAGCAAGCAACGGGGCACCCACCGACAGCTACGAATCT
TTAACAGCAAGTAGTGAGTCTCTAGCAGAAAGCAACGATGCACCCAGCAACAGCTATGAA
TCTTTTCCAGAAATTAGAGAAAATCTAACCGCAAGTGAGGAATCCCTAACATCATGTGAG
GAATCCCTAACAGGAAGTAATGAATCCCTAACAGGAAGTAATGAATCCCTAACAGGAAGT
AAT---------------------------------------------------------
------------------------------------------------GAATCCCTAACA
GAAAGTAGAGAATCTCTAGAGGCCAGTAGAGAATCGCTAAGAGCAAGTAGAGAGTCTCTA
GCGGCCAGTAGAGAATCCCTGAACGACTTTTGTGGGAGCGAAGAATCAGTAGCATTCGAA
GGAGAGCCAAATGAAAAGACATTCATGGGAGACGTCTTAAGTGGTGGAGAATGTGAGAAT
AGTCTCTCAAGAGAAGATTTATTTCATATAGAAGTAGGATCCGAAGAATCGCTAGATGAT
GCCTCAAAATATAATTTCCAAAAGGATTTATCAACTAGCGATAATAGTTCATTCGAAGAT
GATCAGTCATTGAAAAGGGGACTCAAAAGGAACAGCTCAGTATCTAGTCTGGACAGCGAT
ATGGGAAGTTATAAAAATAGAAGATATAGACACAGATTGGATATATATTCGGACCTACCT
AGAAGGCCAATACATGGGGATAATGCACCACAAGAAAAGGAGAAATGTCTGGATTTCAAA
GAACTTCTAGAGAGAGAAAGGAACAACCACCAAAGGAATAGTGTAAATATAAAATTAAAA
AATATATACGGAGAAGAACAATTGTATAATGTGAAGAAAGATAGCTTTATTGTAAAATTG
ATATACTATCTGCCGTTTGGCCCAATTGTTACAATGATAATGATAGGCTTTATTCTACTC
TGGTCAACGCCCTGGACTGGTTTTATTTTTATAGGGTGTAGCTCTATTATATTACCAGCT
TTGTTATACGCGATGCATTCAAACAAAAAACAATTCGAACGTGTATTTGGAAAAGGGAGA
AAAAAGCCAATGAAGGTAAAAAAAGGAGGCGGGAAAATATCTAGATTTTTTAAAGAAGTA
GACAAA--------------------------
>VC928 .
------------------------------TTAAGGAAAATTTCCCTGTGTTCTATTTTC
GTATTGGCATATCTCAGTTCTGCTAAGTGCGCGTACCGAGACGAATCTGACATATTGTAC
AGCGACGACTCCAGATCCGATTTAGCAAGCAACGGGGCACCCACCGACAGCTACGAATCT
TTAACAGCAAGTAGTGAGTCTCTAGCAGAAAGCAACGATGCACCCAGCAACAGCTATGAA
TCTTTTCCAGAAATTAGAGAAAATCTAACCGCAAGTGAGGAATCCCTAACATCATGTGAG
GAATCCCTAACAGGAAGTAATGAATCCCTAACAGGAAGTAATGAATCCCTAACAGGAAGT
AAT---------------------------------------------------------
------------------------------------------------GAATCCCTAACA
GAAAGTAGAGAATCTCTAGAGGCCAGTAGAGAATCGCTAAGAGCAAGTAGAGAGTCTCTA
GCGGCCAGTAGAGAATCCCTGAACGACTTTTGTGGGAGCGAAGAATCAGTAGCATTCGAA
GGAGAGCCAAATGAAAAGACATTCATGGGAGACGTCTTAAGTGGTGGAGAATGTGAGAAT
AGTCTCTCAAGAGAAGATTTATTTCATATAGAAGTAGGATCCGAAGAATCGCTAGATGAT
GCCTCAAAATATAATTTCCAAAAGGATTTATCAACTAGCGATAATAGTTCATTCGAAGAT
GATCAGTCATTGAAAAGGGGACTCAAAAGGAACAGCTCAGAATCTAGTCTGGACAGCGAT
ATGGGAAGTTATAAAAATAAAAGTTATAGACACAGATTGGATATATATTCGGACCTACCT
AGAAGGCCAATACATGGGGATAATGCACCACAAGAAAAGGAGAAATGTCTGGATTTCAAA
GAACTTCTAGAGAGAGAAAGGAACAACCACCAAAGGAATAGTGTAAATATAAAATTAAAA
AATATATACGGAGAAGAACAATTGTATAATGTGAAGAAAGATAGCTTTATTGTAAAATTG
ATATACTATCTGCCGTTTGGCCCAATTGTTACAATGATAATGATAGGCTTTATTCTACTC

TGCTGGACCCCCTCGATTGGTTTTACTTTTATAGGGTGTAGCTCTATTATATTACCAGCT
TTGTTATACGCGATGTATTCAAACAAAAAACAATTCGAACGTGTATTTGGAAAAGGGAGA
AAAAAGCCAATGAAGGTAAAAAAAGGAGGCGGGAAAATATCTAGATTTTTTAAAGAAGTA
GACAAA---------------------------
>VC933 .
------------------------------TTAAGGAAAATTTCCCTGTGTTCTATTTTC
GTATTGGCATATCTCAGTTCTGCTAAGTGCGCGTACCGAGACGAATCTGACATATTGTAC
AGCGACGACTCCAGATCCGATTTAGCAAGCAACGGGGCACCCACCGACAGCTACGAATCT
TTAACAGCAAGTAGTGAGTCTCTAGCAGAAAGCAACGATGCACCCAGCAACAGCTATGAA
TCTTTTCCAGAAATTAGAGAAAATCTAACCGCAAGTGAGGAATCCCTAACATCATGTGAG
GAATCCCTAACAGGAAGTAATGAATCCCTAACAGGAAGTAATGAATCCCTAACAGGAAGT
AAT---------------------------------------------------------
---------------------------GAATCCCTAACAGGAAGTAATGAATCCCTAACA
GAAAGTAGAGAATCTCTAGAGGCCAGTAGAGAATCGCTAAGAGCAAGTAGAGAGTCTCTA
GCGGCCAGTAGAGAATCCCTGAACGACTTTTGTGGGAGCGAAGAATCAGTAGCATGCGAA
GGAGAGCCAAATGAAAAGACATTCATGGGAGACGTCTTAAGTGGTGGAGAATGTGAGAAT
AGTCTCTCAAGAGAAGATTTATTTCATATAGAAGTAGGATCCGAAGAATCGCTAGATGAT
GCCTCAAAATATAATTTCCAAAAGGATTTATCAACTAGCGATAATAGTTCATTCGAAGAT
GATCAGTCATTGAAAAGGGGACTCAAAAGGAACAGCTCAGTATCTAGTCTGGACAGCGAT
ATGGGAAGTTATAAAAATGGAAGATCTAGAGACAGATTGGATATATATTCGGACCTACCT
AGAAGGCTAATACATGGGGATAATGCACCACAAGAAAAGGAGAAATGTCTGGATTTCAAA
GAACTTCTAGAGAGAGAAAGGAACAACCACCAAAGGAATAGTGTAAATATAAAATTAAAA
AATATATACGGAGAAGAACAATTGTATAATGTGAAGAAAGATAGCTTTATTGTAAAATTG
ATATACTATCTGCCGTTTGGCCCAATTGTTACAATGATAATGATAGGTTTTTTTCTACTC
TGCTGGACCCCCTCGATTGGTTTTACTTTTATAGGGTGTAGCTCTATTATATTACCAGCT
TTGTTATACGCGATGTATTCAAACAAAAAACAATTCGAACGTGTATTTGGAAAAGGGAGA
AAAAAGCCAATGAAGGTAAAAAAAGGAGGCGGGAAAATATCTAGATTTTTTAAAGAAGTA
GACAAA---------------------------
>VC944 .
------------------------------TTAAGGAAAATTTCCCTGTGTTCTATTTTC
GTATTGGCATATCTCAGTTCTGCTAAGTGCGCGTACCGAGACGAATCTGACATATTGTAC
AGCGACGACTCCAGATCCGATTTAGCAAGCAACGGGGCACCCACCGACAGCTACGAATCT
TTAACAGCAAGTAGTGAGTCTCTAGCAGAAAGCAACGATGCACCCAGCAACAGCTATGAA
TCTTTTCCAGAAATTAGAGAAAATCTAACCGCAAGTGAGGAATCCCTAACATCATGTGAG
GAATCCCTAACAGGAAGTAATGAATCCCTAACAGGAAGTAATGAATCCCTAACAGGAAGT
AAT---------------------------------------------------------
------------------------------------------------GAATCCCTAACA
GAAAGTAGAGAATCTCTAGAGGCCAGTAGAGAATCGCTAAGAGCAAGTAGAGAGTCTCTA
GCGGCCAGTAGAGAATCCCTGAACGACTTTTGTGGGAGCGAAGAATCAGTAGCATTCGAA
GGAGAGCCAAATGAAAAGACATTCATGGGAGACGTCTTAAGTGGTGGAGAATGTGAGAAT
AGTCTCTCAAGAGAAGATTTATTTCATATAGAAGTAGGATCCGAAGAATCGCTAGATGAT
GCCTCAAAATATAATTTCCAAAAGGATTTATCAACTAGCGATAATAGTTCATTCGAAGAT
GATCAGTCATTGAAAAGGGGACTCAAAAGGAACAGCTCAGTATCTAGTCTGGACAGCGAT
ATGGGAAGTTATAAAAATAAAAGTTATAGACACAGATTGGATATATATTCGGACCTACCT
AGAAGGCCAATACATGGGGATAATGCACCACAAGAAAAGGAGAAATGTCTGGATTTCAAA
GAACTTCTAGAGAGAGAAAGGAACAACCACCAAAGGAATAGTGTAAATATAAAATTAAAA
AATATATACGGAGAAGAACAATTGTATAATGTGAAGAAAGATAGCTTTATTGTAAAATTG
ATATACTATCTGCCGTTTGGCCCAATTGTTACAATGATAATTATAGGCTTTATTCTACTC
TGGTCAACGCCCTGGACTGGTTTTATTTTTATAGGGTGTAGCTCTATTATATTACCAGCT
TTGTTATACGCGATGCATTCAAACAAAAAACAATTCGAACGTGTATTTGGAAAAGGGAGA

AAAAAGCCAATGAAGGTAAAAAAAGGAGGCGGGAAAATATCTAGATTTTTTAAAGAAGTA
GACAAA---------------------------
>VC953 .
------------------------------TTAAGGAAAATTTCCCTGTGTTCTATTTTC
GTATTGGCATATCTCAGTTCTGCTAAGTGCGCGTACCGAGACGAATCTGACATATTGTAC
AGCGACGACTCCAGATCCGATTTAGCAAGCAACGGGGCACCCACCGACAGCTACGAATCT
TTAACAGCAAGTAGTGAGTCTCTAGCAGAAAGCAACGATGCACCCAGCAACAGCTATGAA
TCTTTTCCAGAAATTAGAGAAAATCTAACCGCAAGTGAGGAATCCCTAACATCATGTGAG
GAATCCCTAACAGGAAGTAAT---------------------------------------
------------------------------------------------------------
------------------------------------------------GAATCCCTAACA
GAAAGTAGAGAATCTCTAGAGGCCAGTAGAGAATCGCTAAGAGCAAGTAGAGAGTCTCTA
GCGGCCAGTAGAGAATCCCTGAACGACTTTTGTGGGAGCGAAGAATCAGTAGCATTCGAA
GGAGAGCCAAATGAAAAGACATTCATGGGAGACGTCTTAAGTGGTGGAGAATGTGAGAAT
AGTCTCTCAAGAGAAGATTTATTTCATATAGAAGTAGGATCCGAAGAATCGCTAGATGAT
GCCTCAAAATATAATTTCCAAAAGGATTTATCAACTAGCGATAATAGTTTATTCGAAGAT
GATCAGTCATTGAAAAGGGGACTCAAAAGGAACAGCTCAGTATCTAGTCTGGACAGCGAT
ATGGGAAGTTATAAAAATAAAAGTTATAGACACAGATTGGATATATATTCGGACCTACCT
AGAAGGCTAATACATGGGGATAATGCACCACAAGAAAAGGAGAAATGTCTGGATTTCAAA
GAACTTCTAGAGAGAGAAAGGAACAACCACCAAAGGAATAGTGTAAATATAAAATTAAAA
AATATATACGGAGAAGAACAATTGTATAATGTGAAGAAAGATAGCTTTATTGTAAAATTG
ATATACTATCTGCCGTTTGGCCCAATTGTTACAATGATAATGATAGGCTTTATTCTACTC
TGCTCAACGCCCTCGATTGGTTTTATTTTTATAGGGTGTAGCTCTATTATATTACCAGCT
TTGTTATACGCGATGCATTCAAACAAAAAACAATTCGAACGTGTATTTGGAAAAGGGAGA
AAAAAGCCAATGAAGGTAAAAAAAGGAGGCGGGAAAATATCTAGATTTTTTAAAGAAGTA
GACAAA---------------------------
>VC954 .
------------------------------TTAAGGAAAATTTCCCTGTGTTCTATTTTC
GTATTGGCATATCTCAGTTCTGCTAAGTGCGCGTACCGAGACGAATCTGACATATTGTAC
AGCGACGACTCCAGATCCGATTTAGCAAGCAACGGGGCACCCACCGACAGCTACGAATCT
TTAACAGCAAGTAGTGAGTCTCTAGCAGAAAGCAACGATGCACCCAGCAACAGCTATGAA
TCTTTTCCAGAAATTAGAGAAAATCTAACCGCAAGTGAGGAATCCCTAACATCATGTGAG
GAATCCCTAACAGGAAGTAACGAATCCCTAACAGGAAGTAATGAATCCCTAACAGGAAGT
AAT---------------------------------------------------------
---------------------------GAATCCCTAACAGGAAGTAATGAATCCCTAACA
GAAAGTAGAGAATCTCTAGAGGCCAGTAGAGAATCGCTAAGAGCAAGTAGAGAGTCTCTA
GCGGCCAGTAGAGAATCCCTGAACGACTTTTGTGGGAGCGAAGAATCAGTAGCATTCGAA
GGAGAGCCAAATGAAAAGACATTCATGGGAGACGTCTTAAGTGGTGGAGAATGTGAGAAT
AGTCTCTCAAGAGAAGATTTATTTCATATAGAAGTAGGATCCGAAGAATCGCTAGATGAT
GCCTCAAAATATAATTTCCAAAAGGATTTATCAACTAGCGATAATAGTTTATTCGAAGAT
GATCAGTCATTGAAAAGGGGACTCAAAAGGAACAGCTCAGTATCTAGTCTGGACAGCGAT
ATGGGAAGTTATAAAAATGGAAGATCTAGAGACAGATTGGATATATATTCGGACCTACCT
AGAAGGCTAATACATGGGGATAATGCACCACAAGAAAAGGAGAAATGTCTGGATTTCAAA
GAACTTCTAGAGAGAGAAAGGAACAACCACCAAAGGAATAGTGTAAATATAAAATTAAAA
AATATATACGGAGAAGAACAATTGTATAATGTGAAGAAAGATAGCTTTATTGTAAAATTG
ATATACTATCTGCCGTTTGGCCCAATTGTTACAATGATAATGATAGGTTTTATTCTACTC

TGGTCAACGCCCTGGACTGGTTTTATTTTTATAGGGTGTAGCTCTATTATATTACCAGCT
TTGTTATACGCGATGCATTCAAACAAAAAACAATTCGAACGTGTATTTGGAAAAGGGAGA
AAAAAGCCAATGAAGGTAAAAAAAGGAGGCGGGAAAATATCTAGATTTTTTAAAGAAGTA
GACAAA---------------------------
>VC974 .
------------------------------TTAAGGAAAATTTCCCTGTGTTCTATTTTC
GTATTGGCATATCTCAGTTCTGCTAAGTGCGCGTACCGAGACGAATCTGACATATTGTAC
AGCGACGACTCCAGATCCGATTTAGCAAGCAACGGGGCACCCACCGACAGCTACGAATCT
TTAACAGCAAGTAGTGAGTCTCTAGCAGAAAGCAACGATGCACCCAGCAACAGCTATGAA
TCTTTTCCAGAAATTAGAGAAAATCTAACCGCAAGTGAGGAATCCCTAACATCATGTGAG
GAATCCCTAACAGGAAGTAATGAATCCCTAACAGGAAGTAATGAATCCCTAACAGGAAGT
AAT------------------------------------------GAATCCCTAACAGGA
AGTAATGAATCCCTAACAGGAAGTAATGAATCCCTAACAGGAAGTAATGAATCCCTAACA
GAAAGTAGAGAATCTCTAGAGGCCAGTAGAGAATCGCTAAGAGCAAGTAGAGAGTCTCTA
GCGGCCAGTAGAGAATCCCTGAACGACTTTTGTGGGAGCGAAGAATCAGTAGCATTCGAA
GGAGAGCCAAATGAAAAGACATTCATGGGAGACGTCTTAAGTGGTGGAGAATGTGAGAAT
AGTCTCTCAAGAGAAGATTTATTTCATATAGAAGTAGGATCCGAAGAATCGCTAGATGAT
GCCTCAAAATATAATTTCCAAAAGGATTTATCAACTAGCGATAATAGTTTATTCGAAGAT
GATCAGTCATTGAAAAGGGGACTCAAAAGGAACAGCTCAGTATCTAGTCTGGACAGCGAT
ATGGGAAGTTATAAAAATGGAAGATCTAGAGACAGATTGGATATATATTCGGACCTACCT
AGAAGGCTAATACATGGGGATAATGCACCACAAGAAAAGGAGAAATGTCTGGATTTCAAA
GAACTTCTAGAGAGAGAAAGGAACAACCACCAAAGGAATAGTGTAAATATAAAATTAAAA
AATATATACGGAGAAGAACAATTGTATAATGTGAAGAAAGATAGCTTTATTGTAAAATTG
ATATACTATCTGCCGTTTGGCCCAATTGTTACAATGATAATGATAGGTTTTATTCTACTC
TGGTCAACGCCCTGGACTGGTTTTATTTTTATAGGGTGTAGCTCTATTATATTACCAGCT
TTGTTATACGCGATGCATTCAAACAAAAAACAATTCGAACGTGTATTTGGAAAAGGGAGA
AAAAAGCCAATGAAGGTAAAAAAAGGAGGCGGGAAAATATCTAGATTTTTTAAAGAAGTA
GACAAA---------------------------
>VC976 .
------------------------------TTAAGGAAAATTTCCCTGTGTTCTATTTTC
GTATTGGCATATCTCAGTTCTGCTAAGTGCGCGTACCGAGACGAATCTGACATATTGTAC
AGCGACGACTCCAGATCCGATTTAGCAAGCAACGGGGCACCCACCGACAGCTACGAATCT
TTAACAGCAAGTAGTGAGTCTCTAGCAGAAAGCAACGATGCACCCAGCAACAGCTATGAA
TCTTTTCCAGAAATTAGAGAAAATCTAACCGCAAGTGAGGAATCCCTAACATCATGTGAG
GAATCCCTAACAGGAAGTAATGAATCCCTAACAGGAAGTAATGAATCCCTAACAGGAAGT
AAT---------------------------------------------------------
------------------------------------------------GAATCCCTAACA
GAAAGTAGAGAATCTCTAGAGGCCAGTAGAGAATCGCTAAGAGCAAGTAGAGAGTCTCTA
GCGGCCAGTAGAGAATCCCTGAACGACTTTTGTGGGAGCGAAGAATCAGTAGCATTCGAA
GGAGAGCCAAATGAAAAGACATTCATGGGAGACGTCTTAAGTGGTGGAGAATGTGAGAAT
AGTCTCTCAAGAGAAGATTTATTTCATATAGAAGTAGGATCCGAAGAATCGCTAGATGAT
GCCTCAAAATATAATTTCCAAAAGGATTTATCAACTAGCGATAATAGTTCATTCGAAGAT
GATCAGTCATTGAAAAGGGGACTCAAAAGGAACAGCTCAGAATCTAGTCTGGACAGCGAT
ATGGGAAGTTATAAAAATAAAAGTTATAGACACAGATTGGATATATATTCGGACCTACCT
AGAAGGCCAATACATGGGGATAATGCACCACAAGAAAAGGAGAAATGTCTGGATTTCAAA
GAACTTCTAGAGAGAGAAAGGAACAACCACCAAAGGAATAGTGTAAATATAAAATTAAAA
AATATATACGGAGAAGAACAATTGTATAATGTGAAGAAAGATAGCTTTATTGTAAAATTG
ATATACTATCTGCCGTTTGGCCCAATTGTTACAATGATAATGATAGGCTTTATTCTACTC
TGCTGGACCCCCTCGATTGGTTTTACTTTTATAGGGTGTAGCTCTATTATATTACCAGCT
TTGTTATACGCGATGTATTCAAACAAAAAACAATTCGAACGTGTATTTGGAAAAGGGAGA

AAAAAGCCAATGAAGGTAAAAAAAGGAGGCGGGAAAATATCTAGATTTTTTAAAGAAGTA
GACAAA---------------------------
>VC985 .
------------------------------TTAAGGAAAATTTCCCTGTGTTCTATTTTC
GTATTGGCATATCTCAGTTCTGCTAAGTGCGCGTACCGAGACGAATCTGACATATTGTAC
AGCGACGACTCCAGATCCGATTTAGCAAGCAACGGGGCACCCACCGACAGCTACGAATCT
TTAACAGCAAGTAGTGAGTCTCTAGCAGAAAGCAACGATGCACCCAGCAACAGCTATGAA
TCTTTTCCAGAAATTAGAGAAAATCTAACCGCAAGTGAGGAATCCCTAACATCATGTGAG
GAATCCCTAACAGGAAGTAATGAATCCCTAACAGGAAGTAATGAATCCCTAACAGGAAGT
AAT---------------------------------------------------------
------------------------------------------------GAATCCCTAACA
GAAAGTAGAGAATCTCTAGAGGCCAGTAGAGAATCGCTAAGAGCAAGTAGAGAGTCTCTA
GCGGCCAGTAGAGAATCCCTGAACGACTTTTGTGGGAGCGAAGAATCAGTAGCATTCGAA
GGAGAGCCAAATGAAAAGACATTCATGGGAGACGTCTTAAGTGGTGGAGAATGTGAGAAT
AGTCTCTCAAGAGAAGATTTATTTCATATAGAAGTAGGATCCGAAGAATCGCTAGATGAT
GCCTCAAAATATAATTTCCAAAAGGATTTATCAACTAGCGATAATAGTTCATTCGAAGAT
GATCAGTCATTGAAAAGGGGACTCAAAAGGAACAGCTCAGTATCTAGTCTGGACAGCGAT
ATGGGAAGTTATAAAAATAAAAGTTATAGACACAGATTGGATATATATTCGGACCTACCT
AGAAGGCCAATACATGGGGATAATGCACCACAAGAAAAGGAGAAATGTCTGGATTTCAAA
GAACTTCTAGAGAGAGAAAGGAACAACCACCAAAGGAATAGTGTAAATATAAAATTAAAA
AATATATACGGAGAAGAACAATTGTATAATGTGAAGAAAGATAGCTTTATTGTAAAATTG
ATATACTATCTGCCGTTTGGCCCAATTGTTACAATGATAATTATAGGCTTTATTCTACTC
TGGTCAACGCCCTGGACTGGTTTTATTTTTATAGGGTGTAGCTCTATTATATTACCAGCT
TTGATATACGCGATGCATTCAAACAAAAAACAATTCGAACGTGTATTTGGAAAAGGGAGA
AAAAAGCCAATGAAGGTAAAAAAAGGAGGCGGGAAAATATCTAGATTTTTTAAAGAAGTA
GACAAA---------------------------
>VC987 .
------------------------------TTAAGGAAAATTTCCCTGTGTTCTATTTTC
GTATTGGCATATCTCAGTTCTGCTAAGTGCGCGTACCGAGACGAATCTGACATATTGTAC
AGCGACGACTCCAGATCCGATTTAGCAAGCAACGGGGCACCCACCGACAGCTACGAATCT
TTAACAGCAAGTAGTGAGTCTCTAGCAGAAAGCAACGATGCACCCAGCAACAGCTATGAA
TCTTTTCCAGAAATTAGAGAAAATCTAACCGCAAGTGAGGAATCCCTAACATCATGTGAG
GAATCCCTAACAGGAAGTAATGAATCCCTAACAGGAAGTAATGAATCCCTAACAGGAAGT
AAT---------------------------------------------------------
------------------------------------------------GAATCCCTAACA
GAAAGTAGAGAATCTCTAGAGGCCAGTAGAGAATCGCTAAGAGCAAGTAGAGAGTCTCTA
GCGGCCAGTAGAGAATCCCTGAACGACTTTTGTGGGAGCGAAGAATCAGTAGCATTCGAA
GGAGAGCCAAATGAAAAGACATTCATGGGAGACGTCTTAAGTGGTGGAGAATGTGAGAAT
AGTCTCTCAAGAGAAGATTTATTTCATATAGAAGTAGGATCCGAAGAATCGCTAGATGAT
GCCTCAAAATATAATTTCCAAAAGGATTTATCAACTAGCGATAATAGTTCATTCGAAGAT
GATCAGTCATTGAAAAGGGGACTCAAAAGGAACAGCTCAGAATCTAGTCTGGACAGCGAT
ATGGGAAGTTATAAAAATAAAAGTTATAGACACAGATTGGATATATATTCGGACCTACCT
AGAAGGCCAATACATGGGGATAATGCACCACAAGAAAAGGAGAAATGTCTGGATTTCAAA
GAACTTCTAGAGAGAGAAAGGAACAACCACCAAAGGAATAGTGTAAATATAAAATTAAAA
AATATATACGGAGAAGAACAATTGTATAATGTGAAGAAAGATAGCTTTATTGTAAAATTG
ATATACTATCTGCCGTTTGGCCCAATTGTTACAATGATAATGATAGGCTTTATTCTACTC

TGCTGGACCCCCTCGATTGGTTTTACTTTTATAGGGTGTAGCTCTATTATATTACCAGCT
TTGTTATACGCGATGTATTCAAACAAAAAACAATTCGAACGTGTATTTGGAAAAGGGAGA
AAAAAGCCAATGAAGGTAAAAAAAGGAGGCGGGAAAATATCTAGATTTTTTAAAGAAGTA
GACAAA---------------------------
>BR910 .
------------------------------TTAAGGAAAATTTCCCTGTGTTCTATTTTC
GTATTGGCATATCTCAGTTCTGCTAAGTGCGCGTACCGAGACGAATCTGACATATTGTAC
AGCGACGACTCCAGATCCGATTTAGCAAGCAACGGGGCACCCACCGACAGCTACGAATCT
TTAACAGCAAGTAGTGAGTCTCTAGCAGAAAGCAACGATGCACCCAGCAACAGCTATGAA
TCTTTTCCAGAAATTAGAGAAAATCTAACCGCAAGTGAGGAATCCCTAACATCATGTGAG
GAATCCCTAACAGGAAGTAATGAATCCCTAACAGGAAGTAATGAATCCCTAACAGGAAGT
AAT---------------------------------------------------------
------------------------------------------------GAATCCCTAACA
GAAAGTAGAGAATCTCTAGAGGCCAGTAGAGAATCGCTAAGAGCAAGTAGAGAGTCTCTA
GCGGCCAGTAGAGAATCCCTGAACGACTTTTGTGGGAGCGAAGAATCAGTAGCATTCGAA
GGAGAGCCAAATGAAAAGACATTCATGGGAGACGTCTTAAGTGGTGGAGAATGTGAGAAT
AGTCTCTCAAGAGAAGATTTATTTCATATAGAAGTAGGATCCGAAGAATCGCTAGATGAT
GCCTCAAAATATAATTTCCAAAAGGATTTATCAACTAGCGATAATAGTTCATTCGAAGAT
GATCAGTCATTGAAAAGGGGACTCAAAAGGAACAGCTCAGAATCTAGTCTGGACAGCGAT
ATGGGAAGTTATAAAAATAAAAGTTATAGACACAGATTGGATATATATTCGGACCTACCT
AGAAGGCCAATACATGGGGATAATGCACCACAAGAAAAGGAGAAATGTCTGGATTTCAAA
GAACTTCTAGAGAGAGAAAGGAACAACCACCAAAGGAATAGTGTAAATATAAAATTAAAA
AATATATACGGAGAAGAACAATTGTATAATGTGAAGAAAGATAGCTTTATTGTAAAATTG
ATATACTATCTGCCGTTTGGCCCAATTGTTACAATGATAATGATAGGCTTTATTCTACTC
TGCTGGACCCCCTCGATTGGTTTTACTTTTATAGGGTGTAGCTCTATTATATTACCAGCT
TTGTTATACGCGATGTATTCAAACAAAAAACAATTCGAACGTGTATTTGGAAAAGGGAGA
AAAAAGCCAATGAAGGTAAAAAAAGGAGGCGGGAAAATATCTAGATTTTTTAAAGAAGTA
GACAAA---------------------------
>BR960 .
------------------------------TTAAGGAAAATTTCCCTGTGTTCTATTTTC
GTATTGGCATATCTCAGTTCTGCTAAGTGCGCGTACCGAGACGAATCTGACATATTGTAC
AGCGACGACTCCAGATCCGATTTAGCAAGCAACGGGGCACCCACCGACAGCTACGAATCT
TTAACAGCAAGTAGTGAGTCTCTAGCAGAAAGCAACGATGCACCCAGCAACAGCTATGAA
TCTTTTCCAGAAATTAGAGAAAATCTAACCGCAAGTGAGGAATCCCTAACATCATGTGAG
GAATCCCTAACAGGAAGTAATGAATCCCTAACAGGAAGTAATGAATCCCTAACAGGAAGT
AAT---------------------------------------------------------
---------------------------GAATCCCTAACAGGAAGTAATGAATCCCTAACA
GAAAGTAGAGAATCTCTAGAGGCCAGTAGAGAATCGCTAAGAGCAAGTAGAGAGTCTCTA
GCGGCCAGTAGAGAATCCCTGAACGACTTTTGTGGGAGCGAAGAATCAGTAGCATGCGAA
GGAGAGCCAAATGAAAAGACATTCATGGGAGACGTCTTAAGTGGTGGAGAATGTGAGAAT
AGTCTCTCAAGAGAAGATTTATTTCATATAGAAGTAGGATCCGAAGAATCGCTAGATGAT
GCCTCAAAATATAATTTCCAAAAGGATTTATCAACTAGCGATAATAGTTCATTCGAAGAT
GATCAGTCATTGAAAAGGGGACTCAAAAGGAACAGCTCAGTATCTAGTCTGGACAGCGAT
ATGGGAAGTTATAAAAATGGAAGATCTAGAGACAGATTGGATATATATTCGGACCTACCT
AGAAGGCTAATACATGGGGATAATGCACCACAAGAAAAGGAGAAATGTCTGGATTTCAAA
GAACTTCTAGAGAGAGAAAGGAACAACCACCAAAGGAATAGTGTAAATATAAAATTAAAA
AATATATACGGAGAAGAACAATTGTATAATGTGAAGAAAGATAGCTTTATTGTAAAATTG
ATATACTATCTGCCGTTTGGCCCAATTGTTACAATGATAATGATAGGTTTTTTTCTACTC
TGCTGGACCCCCTCGATTGGTTTTACTTTTATAGGGTGTAGCTCTATTATATTACCAGCT
TTGTTATACGCGATGTATTCAAACAAAAAACAATTCGAACGTGTATTTGGAAAAGGGAGA

AAAAAGCCAATGAAGGTAAAAAAAGGAGGCGGGAAAATATCTAGATTTTTTAAAGAAGTA
GACAAA---------------------------
>Pcynomolgy_1_BAEJ010001511 .
ATGAAAGCAATAATGAATGGTTCCCTTTGTTTAAGGAAAATTTTCCTGTGTTCTATTTTC
GTATTGGTATATCTCAGTTCTGCTAAGTGTGCGTACCGAGACGGATCTGACATATTGTAC
AGCGAAGACTCCACATATGATTTAGCAATCAACGAAGCATCCACCAACAGCTATGAATCT
TTAGCAGCAAGTATTGAATCTCTAACAGCAAGTAGTGAG---------------------
---------------------TCTCTAGCAGCAGGTAGTGAATCTCTAGCAGCAAGTAGT
------------------------------------------------------------
------------------------------------------------------------
------GAATCTCTAACAGGAAGTAGTGAATCTCTAACAGCAAGTAGTGAATCTCTAACA
GCAAGCAGTGAGTCTCTAGCAGCAGGTAGTGAATCTCTAACAGAAAGTAGTGAATCTATA
GCATCAATTAATGAATCCCTGAATGACTTTTATGGCAGCGAAGAATTGATATCATGTGAA
GGAGAGCCAAATAAAAAGAGATTCATAGGAGATGTCTTAAGGGATGGAATATCTGAGGAT
GATCTATTAAGAGAAGATTTATTTCATGTACAAGAAGGATCCGAAGAAATGCTAAATGAC
GTCTTAAAAAATCATTCCCAAAAAGATTTATCTACTAGCGAAAATAGTTTATTCGAAGAT
GATCAGTCATTGAAAAGTGGCTTCAGAAAAAAACTGCTCAGAAACTAGTCTGGACAGCTAT
ATGGGAAGTTTTAAAAATGGAAGATCTAGACACGGATTGGATATAGATTCGGATCCACAT
AGAAGGCCTACGCATGGAGGTAATGAACCACAAGGAGAGGAGAAATCTGTGAACTTCAAA
GAACATCTAGGGAGACAAAGG---AACCAACAAAGGAATAGTGTAAATATAAAATTAAAA
AATTTTTACGGAGAAGAAAAAAATGTATAATTCGAAGAAAAATAGATTTATTATAAAATTT
ATGTACTATCTGCCGTTTGTCCCAATTGTTGCAGTGATAATAATTTTAATGATTCTTCTA
CTCACGACCCCCAAGATTGGTTTGACTTTTTTGTTATGTAGCCCTGCTGTTATAGCAGCT
TTTATATACGTGATGTATTCAAACAGAAAACAATTCAAACGTACATTTGGAACAAGGAGA
------------------------------------------------------------
---------------------------------
>Pcynomolgy_2_BAEJ010008741 .
ATGAAAGCAATAATGAATGGTTCCCTTTGTTTAAGGAAAATTTTCCTGTGTTCTATTTTC
GTATTGGTATATCTCAGTTCTGCTAAGTGTGCGTACCGAGACGGATCTGACATATTGTAC
AGCGAAGACTCCACATATGATTTAGCAATCAACGAAGCATCCACCAACAGCTATGAATCT
TTAGCAGCAAGTATTGAATCTCTAACAGCAAGTAGTGAG---------------------
---------------------TCTCTAGCAGCAGGTAGTGAATCTCTAGCAGCAAGTAGT
------------------------------------------------------------
------------------------------------------------------------
------GAATCTCTAACAGGAAGTAGTGAATCTCTAACAGCAAGTAGTGAATCTCTAACA
GCAAGCAGTGAGTCTCTAGCAGCAGGTAGTGAATCTCTAACAGAAAGTAGTGAATCTATA
GCATCAATTAATGAATCCCTGAATGACTTTTATGGCAGCGAAGAATTGATATCATGTGAA
GGAGAGCCAAATAAAAAGAGATTCATAGGAGATGTCTTAAGGGATGGAATATCTGAGGAT
GATCTATTAAGAGAAGATTTATTTCATGTACAAGAAGGATCCGAAGAAATGCTAAATGAC
GTTTTAAAAAATCATTCCCAAAAAGATTTATCTACTAGCGAAAATAGTTTATTCGAAGAT
GATCAGTCATTGAAAAGTGGCTTCAGAAAAAAACTGCTCAGAAACTAGTCTGGACAGCTAT
ATGGGAAGTTTTAAAAATGGAAGATCTAGACACGGATTGGATATAGATTCGGATCCACAT
AGAAGGCCTACGCATGGAGGTAATGAACCACAAGGAGAGGAGAAATCTGTGAACTTCAAA
GAACATCTAGGGAGACAAAGG---AACCAACAAAGGAATAGTGTAAATATAAAATTAAAA
AATTTTTACGGAGAAGAAAAAAATGTATAATTCGAAGAAAAATAGATTTATTATAAAATTT
ATGTACTATCTGCCGTTTGTCCCAATTGTTGCAGTGATAATAATTTTAATGATTCTTCTA

```
CTCACGACCCCCAAGATTGGTTTGACTTTTTTGTTATGTAGCCCTGCTGTTATAGCAGCT
TTTATATACGTGATGTATTCAAACAGAAAACAATTCAAACGTACATTTGGAACAAGGAGA
------------------------------------------------------------
---------------------------------
>Pinui .
ATGAAAGGAATAGTTATTGGTTTCATTTGTTTAAGGAAAGTTTTCCTGTGTTCCATTTTC
CTATTGGCATATCTGAGTTCGACTAAGTGCGCTTACCGAGAAGGATCTTACATATGGTAC
AACGAAAACTCCACATCTGATTTAGCAATCAACGAGGCATCCACGAGCAGCTATGAATCT
TTAGCATCGAGTAGTGAATCTTTATCAGCACGTAGC------------------------
------------------------------------------------------------
------------------------------------------------------------
------------------------------------------------------------
---------------------------GAATCTCTATCAGCACGTAATGGATCTCTATCA
GCAAGTAGTGAATCCCTAGCAGCAGGTAGTGAATCTCTAGAAGCAAGTAGTGAATCTCTA
GAACCAAGTAATGAATCCGAGGATGACCTTTATGACAGCGAAGAATCCGTAGCATGTCAA
GGAGAGCTAAATAACAAGACCCCCATGTGGGATGGCTTAAGGGATGGAATATCTGAAGAT
GATGTGTCAGGAGAAGATTTGTCTCATGTAGAAGAGGGATTCGAAGAAATGCTAAATGCT
GCCTTAAAAAATAATTTCCAAAAAGATTTATCCACTAGCGAAAATAGTTTATACGGAGAT
GATCAGTTATCGAAAATCGGCTTCAGTAATAATTGCTCAGAAACTAATCTGGACAGCGAC
ATGGGAGATTTTAAAAGTGGAAGGTCTACACACGGATTCGATATAGATATGGACACACCT
AAGAGGTCTACGGATAGAGATCATGCACCCCAAGGAGAGGAGGAATGGATGAATGTCAAA
GCAGATCTACAGAGACAAAGG---AATCACCAAAGGAATAGTGTAAATATAAAATTAAAG
AATTTTTACGGAGAAGAAAAAATATATAATGTGAAGAGAGATAGCTTTATGGTAAAAGTG
ATAAACTATCTGCCGTTTGCCCCAATTGTTACATTGATAATTATAGTCCTTGCTCTATTT
TGCTTCAAATGCCTTTATTGTTGTCTTTTGGTG---TGTAGCTTTGCTATGATAGCAGCT
TGGAAATACCGGAAACGTTTAAACAGAAAACAATTCGAACGTGTATTTGGAAAAAAGAGA
AGAAAGCTAAATAGAGCAGAAAAAAGAGGTGGGAAAATATCTAGATTTCTTAAGGAGGGA
CACAGAGTTTTGTTTGACTATTTGGATCTCTTT
>Pfragile .
ATGAAAGCAATAATAACTGGTTCCCTTTGTTTAATGAAATTTTCTTTGTGTTATATTCTA
TTATTGGCATATCACAGTTCGGCTAAGTTAACGTACCGAGACGTATCTAACATATTGTAC
AGCAGGGACTCCACATCTGATTTAGGAATACAGGAGGCATACACCAACAGCTATGAATCT
TTAGCAGCAAGTAGTGAGTCTTTAGCAGCAAGTAGT------------------------
------------------------------------------------------------
------------------------------------------------------------
------------------------------------------------------------
---------------------------GAGTCTCTATCAGGAAGTAAT------------
------------------------------------------------------------
------------TTATCCCTGAATGACTTATATGGGAGCGCAGAATCGTTAGCATGTGAA
GGAGAGCCACATGACAAGACATTCTTTGGGGATGTCCTGAGCGGTGGAATATCTGCGGAT
GATATATCAAAAGAAGATTTATTTCGTGTAGAACAAGGATACGAAGAAATTCTAAATGAT
GTCTTAAAAAATAAATTCCAAAAAAAAATATCTATTAGCGGAAATAGTTTATTCCAGGAT
GATCAGTCATGGAGAGGGGACTTCAGAAGGAACTACTCAGAAACTAGTCTGGACAGAGAC
ATGGAAAGTTTTAAAAATGAAAAATCTAGAGAGGGATTGAATATAGATTCGGAAGAACCT
AGAAGATCTACGCATGGAGTTAATGCTCCGCAGGGAGAGGAGAAATATGTGAACTTGCAA
GAACATCTGGAGGGACAAAGG---AACCACCAAAGGAATAACGTAAATATAAAATTAAGC
AGAAATTACGGACAAGCAGATATGTATAATGTGAGGAAAAATAGCTTTTTTACAAAATCA
ATATACTATTTGTCGTTTGGCCCAACTGTTACAGTGTTACTTATAATCCTTATTCAACTC
---TCTAATCCCTGGATTGGTTGGCCACTTTTGGTGTGTAGCACTGTTCTGCTATCAGCT
TTGACATACGCGATGCGTTCAAATAAAACACAATGCGAACGTATATTTCGAAAATGGAGA
```

AAAAAGCTGAATAAAGTAAAAAAAGGAGGTTGGACAATACGTAGATTTCTTAACAAGGTA
GACAAATTCTTGTTTGACGTTTTGGATGGCTTT

**B**. Alignment of the *rbsa* found in monkey-malaria parasites.

```
Sal-I-A_AAKM01000020      ATGAAAGGAA TAATGAATGG TTCCCTTTAT TTAAGGAAAA TTTCCCTGTG TTCTATTTTC GTATTGGCAT ATCTCAGTTC TGCTAAGGTA AGATAAAGAC  [ 100]
PV0-A_FLZR010000201       .......... .......... ........G. .......... .......... .......... .......... .......... .......... ..........  [ 100]
India_A_AFBK010012711     .......... .......... .......... .......... .......... .......... .......... .......... .......... ..........  [ 100]
NKorea-A_AFNJ010003131    .......... .......... ........G. .......... .......... .......... .......... .......... .......... ..........  [ 100]
Mau-A_AFNI010001631       .......... .......... ........G. .......... .......... .......... .......... .......... .......... ..........  [ 100]
Sal-I-B_AAKM01000020      ...G..---- --...G.A.. --------GC C...A..T.. ---------- ---------- ---------- -----G..A. .ATCC.CA.. .A....----  [ 100]
PV0-B_FLZR010000201       ...G..---- --...G.A.. --------GC C...A..T.. ---------- ---------- ---------- -----G..A. .ATCC.CA.. .A....----  [ 100]
India-B_AFBK010012681     ...G..---- --...G.A.. --------GC C...A..T.. ---------- ---------- ---------- -----G..A. .ATCC.CA.. .A....----  [ 100]
NKorea-B_AFNJ010003161    ...G..---- --...G.A.. --------GC C...A..T.. ---------- ---------- ---------- -----G..A. .ATCC.CA.. .A....----  [ 100]
Mau-B_AFNI010001651       ...G..---- --...G.A.. --------GC C...A..T.. ---------- ---------- ---------- -----G..A. .ATCC.CA.. .A....----  [ 100]
Pcyn-A_BAEJ010001511      .......C.. .......... ........G. .......... ...T...... .......... .......T.. .......... .......... ......G.T.  [ 100]
Pcyn-B_BAEJ010008741      .......C.. .......... ........G. .......... ...T...... .......... .......T.. .......... .......... ......G.T.  [ 100]
Pinu-A_AMYR010003101      .......... ..G.T.T... ..T.A...G. .........G ...T...... ...C...... C......... ....G..... GA........ ......GCTG  [ 100]
Pfra-A_JOOM010015021      .......C.. ....A.C... ........G. ....T....T ....TT.... ..A....C.A T......... ...A...... G......... ......G.T.  [ 100]

Sal-I-A_AAKM01000020      GCGTTGAAGA GCGCTGTCTG TCGCCCTTGC GCAATGCGTT GCTCGTCTGA GCGTGCACGG GCAGATCAGT AGGCAGACAA ATAGACAGCC TATAAAATGA  [ 200]
PV0-A_FLZR010000201       .......... .......... .......... .......... .......... .......... .......... .......... .....T.... ..........  [ 200]
India_A_AFBK010012711     .......... .......... .......... .......... .......... .......... .......... .......... .......... ..........  [ 200]
NKorea-A_AFNJ010003131    .......... .......... .......... .......... .......... .......... .......... .......... .......... ..........  [ 200]
Mau-A_AFNI010001631       .......... .......... .......... .......... .......... .......... .......... .......... .......... ..........  [ 200]
Sal-I-B_AAKM01000020      ---------- ---------- ---------- ---------- ---------- ---------- ---------- ---------- ------.C.. G.CG...CA.  [ 200]
PV0-B_FLZR010000201       ---------- ---------- ---------- ---------- ---------- ---------- ---------- ---------- ------.C.. G.CG...CA.  [ 200]
India-B_AFBK010012681     ---------- ---------- ---------- ---------- ---------- ---------- ---------- ---------- ------.C.. G.CG...CA.  [ 200]
NKorea-B_AFNJ010003161    ---------- ---------- ---------- ---------- ---------- ---------- ---------- ---------- ------.C.. G.CG...CA.  [ 200]
Mau-B_AFNI010001651       ---------- ---------- ---------- ---------- ---------- ---------- ---------- ---------- ------.C.. G.CG...CA.  [ 200]
Pcyn-A_BAEJ010001511      .......... .....AG... ...T...CA. .......... A.G.T.T... .T........ ......GG.. ..A.G..... .A..G..... ........A.  [ 200]
Pcyn-B_BAEJ010008741      .......... .....AG... ...T...CA. .......... A.G.T.T... .T........ ......GG.. ..A.G..... .A..G..... ........A.  [ 200]
Pinu-A_AMYR010003101      TG.....G.. ..C...GT.. .GAT....A. .A....T.C. A.G.T..... ..C..A.T.. .A.T..G.A. ..AA.A.A.. ....G...T. ........A.  [ 200]
Pfra-A_JOOM010015021      A.A.....C. A.....G... ...T.A..A. .......A.. A.GTT..... .....A.T.. ......GG.. .CA.....G. ...C.T.T.. .G......A.  [ 200]

Sal-I-A_AAKM01000020      ATAAACTTGT ACATAATATG TACGCGCGTG GGAGATATGC TACCCCGCTT ACGCCGTTTA AGCCATTTAC GCCTTATCAT CCC----TTT CCCCTTCCAT  [ 300]
PV0-A_FLZR010000201       .......... .......... .......... .......... .......... .......... .......... .......... ...----... ..........  [ 300]
India_A_AFBK010012711     .......... .......... .......... .......... .......... .......... .......... .......... ...----... ..........  [ 300]
NKorea-A_AFNJ010003131    .......... .......... .......... .......... .......... .......... .......... .......... ...----... ..........  [ 300]
Mau-A_AFNI010001631       .......... .......... .......... .......... .......... .......... .......... .......... ...----... ..........  [ 300]
Sal-I-B_AAKM01000020      .C....C..C -..C.----- -------.CC A.C...GA.T .......T.. .TATA.C... CC..T....T ...G.T..C. A..----... ....C..TG.  [ 300]
PV0-B_FLZR010000201       .C....C..C -..C.----- -------.CC A.C...GA.T .......T.. .TATA.C... CC..T....T ...G.T..C. A..----... ....C..TG.  [ 300]
India-B_AFBK010012681     .C....C..C -..C.----- -------.CC A.C...GA.T .......T.. .TATA.C... CC..T....T ...G.T..C. A..----... ....C..TG.  [ 300]
NKorea-B_AFNJ010003161    .C....C..C -..C.----- -------.CC A.C...GA.T .......T.. .TATA.C... CC..T....T ...G.T..C. A..----... ....C..TG.  [ 300]
Mau-B_AFNI010001651       .C....C..C -..C.----- -------.CC A.C...GA.T .......T.. .TATA.C... CC..T....T ...G.T..C. A..----... ....C..TG.  [ 300]
Pcyn-A_BAEJ010001511      .......... .......... ....T.TT.. .......CAT .......T.. ..T.A.---- ---------- ----....C. ...----... ..T.C..TG.  [ 300]
Pcyn-B_BAEJ010008741      .......... .......... ....T.TT.. .......CAT .......T.. ..T.A.---- ---------- ----....C. ...----... ..T.C..TG.  [ 300]
Pinu-A_AMYR010003101      ...G...... .......... CT.CT..... ..CT...CAT ...G...TC. G..TAAC--- ---------- -----...T. ..T----... ..T.C..TG.  [ 300]
Pfra-A_JOOM010015021      .....G.... ...A.....A ....T..... ..C..C..AT .......T.. ..-------- ---------- ..TA....C. ...CCTT... ..T.G.GTG.  [ 300]

Sal-I-A_AAKM01000020      TAAGTGCGCG TACCGAGACG AATCTGACAT ATTGTACAGC GACGACTCCA GATCCGATTT AGCAAGCAAC GGGGCACCCA CCGACAGCTA CGAATCTTTA  [ 400]
PV0-A_FLZR010000201       .C........ .......... .......... .......... .......... .......... .......... .......... .......... ..........  [ 400]
```

```
India_A_AFBK010012711      .......... .......... .......... .......... .......... .......... .......... .......... .......... ..........  [ 400]
NKorea-A_AFNJ010003131     .C........ .......... .......... .......... .......... .......... .......... .......... .......... ..........  [ 400]
Mau-A_AFNI010001631        .C........ .......... .......... .......... .......... .......... .......... .......... .......... ..........  [ 400]
Sal-I-B_AAKM01000020       .C...A...C .T..T..... G......... ..G....... .......... C...T..... .......... .AC...T... ..A....... T.........  [ 400]
PV0-B_FLZR010000201        .C...A...C .T..T..... G......... ..G....... .......... C...T..... .......... .AC...T... ..A....... T.........  [ 400]
India-B_AFBK010012681      .C.A.A...C .T..T..... G......... ..G....... .......... C...T..... .......... .AC...T... ..A....... T.........  [ 400]
NKorea-B_AFNJ010003161     .C...A...C .T..T..... G......... ..G....... .......... C...T..... .......... .AC...T... ..A....... T.........  [ 400]
Mau-B_AFNI010001651        .C...A...C .T..T..... G......... ..G....... .......... C...T..... .......... .AC...T... ..A....... T.........  [ 400]
Pcyn-A_BAEJ010001511       .C....T... .......... G......... .......... ..A....... C..AT..... .....T.... .AA...T... ..A....... T.........  [ 400]
Pcyn-B_BAEJ010008741       .C....T... .......... G......... .......... ..A....... C..AT..... .....T.... .AA...T... ..A....... T.........  [ 400]
Pinu-A_AMYR010003101       .T.......T ........A. G...T..... ..G.....A. ..AA...... C...T..... .....T.... .A....T... .GAG...... T.........  [ 400]
Pfra-A_JOOM010015021       .C...TAA.. .......... T...A..... .......... AGG....... C...T..... ..G..TAC.G .A....TA.. ..A....... T.........  [ 400]

Sal-I-A_AAKM01000020       ACAGCAAGTA GTGAGTCTCT AGCAGAAAGC AACGATGCAC CCAGCAACAG CTATGAATCT TTTCCAGAAA TTAGAGAAAA TCTAACCGCA AGTGAGGAAT  [ 500]
PV0-A_FLZR010000201        .......... .......... .......... .......... .......... .......... .......... .......... .......... ..........  [ 500]
India_A_AFBK010012711      .......... .......... .......... .......... .......... .......... .......... .......... .......... ..........  [ 500]
NKorea-A_AFNJ010003131     .......... .......... .......... .......... .......... .......... .......... .......... .......... ..........  [ 500]
Mau-A_AFNI010001631        .......... .......... .......... .......... .......... .......... .......... .......... .......... ..........  [ 500]
Sal-I-B_AAKM01000020       GA....C... .......... ..G..C...T .G.......T .A.C...... ...------- ---------- ---------- ---------- ----------  [ 500]
PV0-B_FLZR010000201        GA....C... .......... ..G..C...T .G.......T .A.C...... ...------- ---------- ---------- ---------- ----------  [ 500]
India-B_AFBK010012681      GA....C... .......... ..G..C...T .G.......T .A.C...... ...------- ---------- ---------- ---------- ----------  [ 500]
NKorea-B_AFNJ010003161     GA....C... .......... ..G..C...T .G.......T .A.C...... ...------- ---------- ---------- ---------- ----------  [ 500]
Mau-B_AFNI010001651        GA....C... .......... ..G..C...T .G.......T .A.C...... ...------- ---------- ---------- ---------- ----------  [ 500]
Pcyn-A_BAEJ010001511       G......... T...A..... .A...C...T .GT..-.TCT .T....G... G..------- ---------- ---------- ---------- ----------  [ 500]
Pcyn-B_BAEJ010008741       G......... T...A..... .A...C...T .GT..-.TCT .T....G... G..------- ---------- ---------- ---------- ----------  [ 500]
Pinu-A_AMYR010003101       G..T.G.... ....A...T. .T...C.C.T .G...----- ---------- ---------- ---------- ---------- ---------- ----------  [ 500]
Pfra-A_JOOM010015021       G......... ........T. ...------- ---------- ---------- ---------- ---------- ---------- ---------- ----------  [ 500]

Sal-I-A_AAKM01000020       CCCTAACATC ATGTGAGGAA TCCCTAACAG GAAGTAATGA ATCCCTAACA GGAAGTAATG AATCCCTAAC AGGAAGTAAT GAATCCCTAA CAGGAAGTAA  [ 600]
PV0-A_FLZR010000201        .......... .......--- ---------- ---------- ---------- ---------. .......... .......... .......... ..........  [ 600]
India_A_AFBK010012711      .......... .......--- ---------- ---------- ---------- ---------. .......... .......... .......... ..........  [ 600]
NKorea-A_AFNJ010003131     .......... .......--- ---------- --------.. .......... .......... .......... .......... .......... ..........  [ 600]
Mau-A_AFNI010001631        .......... .......--- ---------- --------.. .......... .......... .......... .......... .......... ..........  [ 600]
Sal-I-B_AAKM01000020       ---------- ---------- ---------- ---------- ---------- ---------- ---------- ---------. ......T..G AT.A.T..GG  [ 600]
PV0-B_FLZR010000201        ---------- ---------- ---------- ---------- ---------- ---------- ---------- ---------. ......T..G AT.A.T..GG  [ 600]
India-B_AFBK010012681      ---------- ---------- ---------- ---------- ---------- ---------- ---------- ---------. ......T..G AT.A.T..GG  [ 600]
NKorea-B_AFNJ010003161     ---------- ---------- ---------- ---------- ---------- ---------- ---------- ---------. ......T..G AT.A.T..GG  [ 600]
Mau-B_AFNI010001651        ---------- ---------- ---------- ---------- ---------- ---------- ---------- ---------. ......T..G AT.A.T..GG  [ 600]
Pcyn-A_BAEJ010001511       ---------- ---------- ---------- ---------- ---------- -------G.. ....T...G. ..C.....G. .....T.... .........G  [ 600]
Pcyn-B_BAEJ010008741       ---------- ---------- ---------- ---------- ---------- -------G.. ....T...G. ..C.....G. .....T.... .........G  [ 600]
Pinu-A_AMYR010003101       ---------- ---------- ---------- ---------- ---------- ---------- ---------- ---------- --...T...T ...C.C....  [ 600]
Pfra-A_JOOM010015021       ---------- ---------- ---------- ---------- ---------- ---------- ---------- ---------- ---------- ----------  [ 600]

Sal-I-A_AAKM01000020       TGAATCCCTA ACAGGAAGTA ATGAATCCCT AACAGAAAGT AGAGAATCTC TAGAGGCCAG TAGAGAATCG CTAAGAGCAA GTAGAGAGTC TCTAGCGGCC  [ 700]
PV0-A_FLZR010000201        .......... .......... .......... .......... .......... .......... .......... .......... .......... ..........  [ 700]
India_A_AFBK010012711      .......... .......... .......... .......... .......... .......... .......... .......... .......... ..........  [ 700]
NKorea-A_AFNJ010003131     .......... .......... .......... .......... .......... .......... .......... .......... .......... ..........  [ 700]
Mau-A_AFNI010001631        .......... .......... .......... .......... .......... .......... .......... .......... .......... ..........  [ 700]
Sal-I-B_AAKM01000020       ...G..G..C GG..C...C. GC..TG.ATC C..GA.C..C T.T......T ..TCA..AT. ...T..G..C ..CG...... .......A.. ....A.A.AA  [ 700]
PV0-B_FLZR010000201        ...G..G..C GG..C...C. GC..TG.ATC C..GA.C..C T.T......T ..TCA..AT. ...T..G..C ..CG...... .......A.. ....A.A.AA  [ 700]
India-B_AFBK010012681      ...G..G..C GG..C...C. GC..TG.ATC C..GA.C..C T.T......T ..TCA..AT. ...T..G..C ..CG...... .......A.. ....A.A.AA  [ 700]
NKorea-B_AFNJ010003161     ...G..G..C GG..C...C. GC..TG.ATC C..GA.C..C T.T......T ..TCA..AT. ...T..G..C ..CG...... .......A.. ....A.A.AA  [ 700]
```

```
Mau-B_AFNI010001651        ...G..G..C GG..C...C. GC..TG.ATC C..GA.C..C T.T......T ..TCA..AT. ...T..G..C ..CG...... .......A.. ....A.A.AA  [ 700]
Pcyn-A_BAEJ010001511       ......T... ....C..... G......T.. .....C...C ..T..G.... ...CA..AG. ...T.....T ....C..A.. ....T..A.. .A....AT.A  [ 700]
Pcyn-B_BAEJ010008741       ......T... ....C..... G......T.. .....C...C ..T..G.... ...CA..AG. ...T.....T ....C..A.. ....T..A.. .A....AT.A  [ 700]
Pinu-A_AMYR010003101       ..G...T... T...C..... ---------- ---------- -.T.....C. ...CA..AG. ...T.....T ...GA..... ....T..A.. .....AAC.A  [ 700]
Pfra-A_JOOM010015021       ---------- ---------- ---------- ---------- ---------- ---------- ---------- -----..... ....T..... ....T.A.GA  [ 700]
Sal-I-A_AAKM01000020       AGTAGAGAAT CCCTGAACGA CTTTTGTGGG AGCGAAGAAT CAGTAGCATT CGAAGGAGAG CCAAATGAAA AGACATTCAT GGGAGACGTC TTAAGTGGTG  [ 800]
PV0-A_FLZR010000201        .......... .......... .......... .......... .......... .......... .......... .......... .......... ..........  [ 800]
India_A_AFBK010012711      .......... .......... .......... .......... .......... .......... .......... .......... .......... ..........  [ 800]
NKorea-A_AFNJ010003131     .......... .......... .......... .......... .........G .......... .......... .......... .......... ..........  [ 800]
Mau-A_AFNI010001631        .......... .......... .......... .......... .........G .......... .......... .......... .......... ..........  [ 800]
Sal-I-B_AAKM01000020       ....AT.... ..T....... G....A...C G.A....... .GA......G T....AT... ......A... ...A...... ...G..T.AA .....GA...  [ 800]
PV0-B_FLZR010000201        ....AT.... ..T....... G....A...C G.A....... .GA......G T....AT... ......A... .......... ...G..T.AA .....GA...  [ 800]
India-B_AFBK010012681      ....AT.... ..T....... G....A.A.C G.A....... .GA......G T....AT... ......A... .......... ...G..T.AA .....GA...  [ 800]
NKorea-B_AFNJ010003161     ....AT.... ..T....... G....A...C G.A....... .GA......G T....AT... ......A... .......... ...G..T.AA .....GA...  [ 800]
Mau-B_AFNI010001651        ....AT.... ..T....... G....A...C G.A....... .GA......G T....AT... T.....A... .......... ...G..T.AA .....GA...  [ 800]
Pcyn-A_BAEJ010001511       .T..AT.... .......T.. .....A...C .......... TGA..T...G T......... ......A... ...G...... A.....T... .....G.A..  [ 800]
Pcyn-B_BAEJ010008741       .T..AT.... .......T.. .....A...C .......... TGA..T...G T......... ......A... ...G...... A.....T... .....G.A..  [ 800]
Pinu-A_AMYR010003101       ....AT.... ..GA.G.T.. .C...A..AC .......... .C.......G TC........ .T....A.C. ....CCC... .T.G..T.G. .....G.A..  [ 800]
Pfra-A_JOOM010015021       ....ATTT.. .......T.. ...A.A.... ....C..... .GT......G T......... ...C....C. ........T. T..G..T... C.G..C....  [ 800]

Sal-I-A_AAKM01000020       GAGAATGTGA GAATAGTCTC TCAAGAGAAG ATTTATTTCA TATAGAAGTA GGATCCGAAG AATCGCTAGA TGATGCCTCA AAATATAATT TCCAAAAGGA  [ 900]
PV0-A_FLZR010000201        .......... .......... .......... .......... .......... .......... .......... .......... .......... ..........  [ 900]
India_A_AFBK010012711      .......... .......... .......... .......... .......... .......... .......... .......... .......... ..........  [ 900]
NKorea-A_AFNJ010003131     .......... .......... .......... .......... .......... .......... .......... .......... .......... ..........  [ 900]
Mau-A_AFNI010001631        .......... .......... .......... .......... .......... .......... .......... .......... .......... ..........  [ 900]
Sal-I-B_AAKM01000020       A.AT..C... .G...A...A C......... .......... .G......A. ....A..... ..AT....A. ....T...T. .......... .....T.A..  [ 900]
PV0-B_FLZR010000201        A.AT..C... .G...A...A C......... .......... .G......A. ....A..... ..AT....A. ....T...T. .......... .....T.A..  [ 900]
India-B_AFBK010012681      A.AT..C... .G...A...A C......... .......... .G......A. ....A..... ..AT....A. ....T...T. .......... .....T.A..  [ 900]
NKorea-B_AFNJ010003161     A.AT..C... .G...A...A C......... .......... .G......A. ....A..... ..AT....A. ....T...T. .......... .....T.A..  [ 900]
Mau-B_AFNI010001651        A.AT..C... .G...A...A C......... .......... .G......A. ....A..... ..AT....A. ....T...T. .......... .....T.A..  [ 900]
Pcyn-A_BAEJ010001511       ..AT..C... .G..GA...A .T........ .......... .G..C...A. .......... ..AT....A. ...C.T..T. ...A..C... C......A..  [ 900]
Pcyn-B_BAEJ010008741       ..AT..C... .G..GA...A .T........ .......... .G..C...A. .......... ..AT....A. ...C.TT.T. ...A..C... C......A..  [ 900]
Pinu-A_AMYR010003101       ..AT..C... AG..GA.G.G ...G...... ....G.C... .G......AG ....T..... ..AT....A. ..C.....T. ...A...... .......A..  [ 900]
Pfra-A_JOOM010015021       ..AT..C..C .G..GA.A.A ....A..... .........G .G.....CA. ....A..... ..ATT...A. .....T..T. ...A....A. .......AA.  [ 900]

Sal-I-A_AAKM01000020       TTTATCAACT AGCGATAATA GTTCATTCGA AGATGATCAG TCATTGAAAA GGGGACTCAA AAGGAACAGC TCAGTATCTA GTCTGGACAG CGATATGGGA  [1000]
PV0-A_FLZR010000201        .......... .......... .......... .......... .......... .......... .......... .......... .......... ..........  [1000]
India_A_AFBK010012711      .......... .......... .......... .......... .......... .......... .......... .......... .......... ..........  [1000]
NKorea-A_AFNJ010003131     .......... .......... .......... .......... .......... .......... .......... .......... .......... ..........  [1000]
Mau-A_AFNI010001631        .......... .......... ...T...... .......... .......... .......... .......... .......... .......... ..........  [1000]
Sal-I-B_AAKM01000020       ......T... ...A.A.... ...T...... .......... .......... .......... .......... ....A..... ....A..... .C.C......  [1000]
PV0-B_FLZR010000201        ......T... ...A.A.... ...T...... .......... .......... .......... .......... ....A..A.. ....A..... .C.C......  [1000]
India-B_AFBK010012681      ......T... ...A.A.... ...T...... .......... .......... .......... .......... ....A..... ....A..... .C.C......  [1000]
NKorea-B_AFNJ010003161     ......T... ...A.A.... ...T...... .......... .......... .......... .......... ....A..... ....A..... .C.C......  [1000]
Mau-B_AFNI010001651        ......T... ...A.A.... ...T...... .......... .......... .......... .....G.... ....A..... ....A..... .C.C......  [1000]
Pcyn-A_BAEJ010001511       ......T... .....A.... ...T...... .......... .......... .T..CT...G ..AA...T.. ....A.A... .......... .T........  [1000]
Pcyn-B_BAEJ010008741       ......T... .....A.... ...T...... .......... .......... .T..CT...G ..AA...T.. ....A.A... .......... .T........  [1000]
Pinu-A_AMYR010003101       ......C... .....A.... ...T..A..G .......... .T..C..... TC..CT...G T.AT..TT.. ....A.A... A......... ...C......  [1000]
Pfra-A_JOOM010015021       AA....T.T. ....GA.... ...T....C. G......... ....G..G.G ...ACT...G .......TA. ....A.A... .......... A..C....A.  [1000]

Sal-I-A_AAKM01000020       AGTTATAAAA ATAAAAGTTA TAGACACAGA TTGGATATAT ATTCGGACCT ACCTAGAAGG CCAATACATG GGGATAATGC ACCACAAGAA AAGGAGAAAT  [1100]
PV0-A_FLZR010000201        .......... .......... .......... .......... .......... .......... .......... .......... .......... ..........  [1100]
India_A_AFBK010012711      .......... .......... .......... .......... .......... .......... .......... .......... .......... ..........  [1100]
NKorea-A_AFNJ010003131     .......... ..GG...A.C ....G..... .......... .......... .......... .T........ .......... .......... ..........  [1100]
Mau-A_AFNI010001631        .......... ..GG...A.C ....G..... .......... .......... .......... .T........ .......... .......... ..........  [1100]
Sal-I-B_AAKM01000020       .......... .......... .......... .........G .......A.C T........A T.T....... ...G...... ........G. G..A......  [1100]
```

```
PV0-B_FLZR010000201        .......... .......... .......... .........G .......A.C T........A T.T....... ...G...... ........G. G..A......  [1100]
India-B_AFBK010012681      .......... .......... .......... .........G .......A.C T........A T.T....... ...G...... ........G. G..A......  [1100]
NKorea-B_AFNJ010003161     .......... .......... .......... .........G .......A.C T........A T.T....... ...G...... ........G. G..A......  [1100]
Mau-B_AFNI010001651        .......... .......... .......... .........G .......A.C T........A T.T....... ...G...... ........G. G..A......  [1100]
Pcyn-A_BAEJ010001511       ....T..... ..GG...A.C .......G.. .........G .......T.C ..A....... ..T.CG.... .A.G.....A ........G. G.........  [1100]
Pcyn-B_BAEJ010008741       ....T..... ..GG...A.C .......G.. .........G .......T.C ..A....... ..T.CG.... .A.G.....A ........G. G.........  [1100]
Pinu-A_AMYR010003101       GA..T..... G.GG...G.C ..C....G.. ..C......G ..AT....AC .....AG... T.T.CGG..A .A...C.... ...C....G. G.....G...  [1100]
Pfra-A_JOOM010015021       ....T..... ..G...AA.C ....G.GG.. ...A.....G .......AGA .........A T.T.CG.... .A.T...... T..G..G.G. G.........  [1100]
Sal-I-A_AAKM01000020       GTCTGGATTT CAAAGAACTT CTAGAGAGAG AAAGGAACAA CCACCAAAGG AATAGTGTAA ATATAAAATT AAAAAATATA TACGGAGAAG AACAATTGTA  [1200]
PV0-A_FLZR010000201        .......... .......... .......... .......... .......... .......... .......... .......... .......... ..........  [1200]
India_A_AFBK010012711      .......... .......... .......... .......... .......... .......... .......... .......... .......... ..........  [1200]
NKorea-A_AFNJ010003131     .......... .......... .......... .......... .......... .......... .......... .......... .......... ..........  [1200]
Mau-A_AFNI010001631        .......... .......... .......... .......... .......... .......... .......... .......... .......... ..........  [1200]
Sal-I-B_AAKM01000020       A.G....C.. ..G....... ......-..C .....---.. .......... .......... ..G....... .........T .......... .....A....  [1200]
PV0-B_FLZR010000201        A.G....C.. ..G....... ......-..C .....---.. .......... .......... ..G....... .........T .......... .....A....  [1200]
India-B_AFBK010012681      A.G....C.. ..G....... ......-..C .....---.. .......... .......... ..G....... .........T .......... .....A....  [1200]
NKorea-B_AFNJ010003161     A.G....C.. ..G....... ......-..C .....---.. .......... .......... ..G....... .........T .......... .....A....  [1200]
Mau-B_AFNI010001651        A.G....C.. ..G....... ......-..C .....---.. .......... .......... ..G....... .........T .......... .....A....  [1200]
Pcyn-A_BAEJ010001511       C.G..A.C.. ........A. ....G....C .....---.. ...A...... .......... .......... .......T.T .......... ..A..A....  [1200]
Pcyn-B_BAEJ010008741       C.G..A.C.. ........A. ....G....C .....---.. ...A...... .......... .......... .......T.T .......... ..A..A....  [1200]
Pinu-A_AMYR010003101       .GA..A..G. .....C.GA. ...C.....C .....---.. T......... .......... .......... ...G...T.T .......... ..A..A.A..  [1200]
Pfra-A_JOOM010015021       A.G..A.C.. GC......A. ..G...G..C .....---.. .......... ....AC.... .......... ..GC.GA.AT ......C... C.G.TA....  [1200]

Sal-I-A_AAKM01000020       TAATGTGAAG AAAGATAGCT TTATTGTAAA ATTGATATAC TATTTGCCGT TTGGCCCAAT TGTTACAATG ATAATGA--- TAGGCTTTAT TCTACTCTGG  [1300]
PV0-A_FLZR010000201        .......... .......... .......... .......... ...C...... .......... .......... .....T.--- .......... .........C  [1300]
India_A_AFBK010012711      .......... .......... .......... .......... ...C...... .......... .......... .......--- .......... .........C  [1300]
NKorea-A_AFNJ010003131     .......... .......... .......... .......... ...C...... .......... .......... .....T.--- .......... ..........  [1300]
Mau-A_AFNI010001631        .......... .......... .......... .......... ...C...... .......... .......... .......--- .......... ..........  [1300]
Sal-I-B_AAKM01000020       ...A...... .G.A...... .......... .......... .......... ..TT....G. .......... .......--- ....T...T. .........C  [1300]
PV0-B_FLZR010000201        ...A...... .G.A...... .......... .......... .......... ..TT....G. .......... .......--- ....T...T. .........C  [1300]
India-B_AFBK010012681      ...A...... .G.A...... .......... .......... .......... ..TT....G. .......... .......--- ....T...T. .........C  [1300]
NKorea-B_AFNJ010003161     ...A...... .G.A...... .......... .......... .......... ..TT....G. .......... .......--- ....T...T. .........C  [1300]
Mau-B_AFNI010001651        ...A...... .G.A...... .......... .......... ...C...... .......... .......... .......--- ....T...T. .........C  [1300]
Pcyn-A_BAEJ010001511       ....TC.... ...A....A. .....A.... ...T..G... ...C...... ...T...... ....G..G.. .....A.TTT ..ATGA..C. .......---  [1300]
Pcyn-B_BAEJ010008741       ....TC.... ...A....A. .....A.... ...T..G... ...C...... ...T...... ....G..G.. .....A.TTT ..ATGA..C. .......---  [1300]
Pinu-A_AMYR010003101       .......... .G........ ....G..... .G.....A.. ...C...... ...C...... .......T.. .....T.--- ...T.C..GC ....T.T..C  [1300]
Pfra-A_JOOM010015021       ........G. ...A...... ..T..AC... ..CA...... ......T... .........C .......G.. T..C.T.--- ..AT.C.... ..A....---  [1300]

Sal-I-A_AAKM01000020       TCAACGCCCT GGACTGGTTT TAT-----TT TTATAGGGTG TAGCTCTATT ATATTACCAG CTTTGTTATA CGCGATGCAT TCAAACAAAA AACAATTCGA  [1400]
PV0-A_FLZR010000201        .......... C......... ...-----.. .......... .......... .......... .......... .......... .......... ..........  [1400]
India_A_AFBK010012711      .......... C......... ...-----.. .......... .......... .......... .......... .......... .......... ..........  [1400]
NKorea-A_AFNJ010003131     ......GG.. .......... ...-----.. .......... .......... .......... .......... .......T.. .......... ..........  [1400]
Mau-A_AFNI010001631        ......G.T. .......... ...-----.. .......... .......... .......... .......... .......... .......... ..........  [1400]
Sal-I-B_AAKM01000020       .GG..C.... C..T...... G..-----.. ..TGG...C. ..A....... G.GA...... .....A.G.. .......... .......... ..........  [1400]
PV0-B_FLZR010000201        .GG..C.... C..T...... G..-----.. ..TGG...C. ..A....... G.GA...... .....A.G.. .......... .......... ..........  [1400]
India-B_AFBK010012681      .GG..C.... C..T...... G..-----.. ..TGG...C. ..A....... G.GA...... .....A.... .......... .......... ..-.......  [1400]
NKorea-B_AFNJ010003161     .GG..C.... C..T...... G..-----.. ..TGG...C. ..A....... G.GA...... .....A.G.. .......... .......... ..........  [1400]
Mau-B_AFNI010001651        .GG..C.... C..T...... ..C-----.. .......... .......... .......... .......... .......... .......... ..........  [1400]
Pcyn-A_BAEJ010001511       A.G..C...A A..T...... G.C-----.. ..T.GTTA.. ....C..GC. G.TA..G... ....TA.... ..T....T.. .......G.. .........A.  [1400]
Pcyn-B_BAEJ010008741       A.G..C...A A..T...... G.C-----.. ..T.GTTA.. ....C..GC. G.TA..G... ....TA.... ..T....T.. .......G.. .........A.  [1400]
Pinu-A_AMYR010003101       .--------. CA.A..CC.. ...TGTTG.C ..T.G.T... .....T.GC. ..GA..G... ...G.AA... .CG..AA.G. .T.....G.. ..........  [1400]
Pfra-A_JOOM010015021       ..T.AT.... ...T.....G GCC-----AC ..T.G.T... ....A..G.. C.GC..T... .....AC... ........G. .....T.... C.....G...  [1400]
```

```
Sal-I-A_AAKM01000020      ACGTGTATTT GGAAAAGGGA GAAAAAAGCC AATGAAGGTA AAAAAAGGAG GCGGGAAAAT ATCTAGATTT TTTAAAGAAG TAGACAAATT CTTGTTTGAC  [1500]
PV0-A_FLZR010000201       .......... .......... .......... .......... .......... .......... .......... .......... .......... ..........  [1500]
India_A_AFBK010012711     .......... .......... .......... .......... .......... .......... .......... .......... .......... ..........  [1500]
NKorea-A_AFNJ010003131    .......... .......... .......... .......... .......... .......... .......... .......... .......... ..........  [1500]
Mau-A_AFNI010001631       .......... .......... ...------- ---------- ---------- ---------- ---------- ---------- ---------- ----------  [1500]
Sal-I-B_AAKM01000020      .......... ......A... ..C....A.A ..A...A... .........A ....A.T... .......... ........G. ........C. ..........  [1500]
PV0-B_FLZR010000201       .......... ......A... ..C....A.A ..A...A... .........A ....A.T... .......... ........G. ........C. ..........  [1500]
India-B_AFBK010012681     .G.G...... .......... .......... .......... .......... .......... .......... .......... .......... ..........  [1500]
NKorea-B_AFNJ010003161    .......... ......A... ..C....A.A ..A...A... .........A ....A.T... .......... ........G. ........C. ..........  [1500]
Mau-B_AFNI010001651       .......... .......... .......... .......... .......... .......... .......... .......... .......... ..........  [1500]
Pcyn-A_BAEJ010001511      ....AC.... ....C.A... ..T....... .TAT..AT.. .....G.... .......... .......... ......A.GT ........A. ..........  [1500]
Pcyn-B_BAEJ010008741      ....AC.... ....C.A... ..T....... .TAT..AAA. ...GGG.... .......... .......... ......A.GT ........A. ..........  [1500]
Pinu-A_AMYR010003101      .......... ......AA.. ...G.....T ..AT.GA.C. G.....A... .T........ .......... C....G..G. G.C...G.G. T.........  [1500]
Pfra-A_JOOM010015021      ....A..... C.....T... .........T G.AT..A... .......... .TT...C... .CG....... C....CA.G. .......... ..........  [1500]

Sal-I-A_AAKM01000020      GATTTGGATG GTTATTAA [1518]
PV0-A_FLZR010000201       .......... ........ [1518]
India_A_AFBK010012711     .......... ........ [1518]
NKorea-A_AFNJ010003131    .......... ........ [1518]
Mau-A_AFNI010001631       ---------- -------- [1518]
Sal-I-B_AAKM01000020      .......... ........ [1518]
PV0-B_FLZR010000201       .......... ........ [1518]
India-B_AFBK010012681     .......... ........ [1518]
NKorea-B_AFNJ010003161    .......... ........ [1518]
Mau-B_AFNI010001651       .......... ........ [1518]
Pcyn-A_BAEJ010001511      .......... .C.T.... [1518]
Pcyn-B_BAEJ010008741      .......... .C.T..T. [1518]
Pinu-A_AMYR010003101      T........C TC.T.... [1518]
Pfra-A_JOOM010015021      .T........ .C.T.... [1518]
```

**C**. Alignment of the 4,000 bp *P. vivax* duplicated fragment

```
pvrbsa      ATGAAAGGAA TAATGAATGG TTCCCTTTAT TTAAGGAAAA TTTCCCTGTG TTCTATTTTC GTATTGGCAT ATCTCAGTTC ---TGCTAAG GTAAGATAAA  [ 100]
pvrbsap     CAC...A.C. A.C..C.A.A ------A... .CC...TGC. .G...A---- ---------- -----.A..A ...G-.A... AAAA...... AA..A.A..T  [ 100]

pvrbsa      GACGCGTTGA AGAGCGCTGT CTGTCGCCCT TGCG-----C AATGCGTTGC TCGTCTGAGC GTGCAC---- ---------- ------GGGC ----AGATCA  [ 200]
pvrbsap     A.AAGA.AA. ..TA....A. G.A.AT..AG .AG.AAGAC. .T.T.AA.AT A.TC...TAT A..T..CACC CCCAATGGAA ATGGAA.... CTAA....A.  [ 200]

pvrbsa      G----TAGGC AGACAAATAG ACAGCCTATA AAATGAATAA ACTTGTACAT AATATGTACG CGCGTGGGAG ATATGCTACC CCGCTTACGC CGTTTAAGCC  [ 300]
pvrbsap     .GTAC..TC. .C.T...-.T .A.C..G.CG ...CA..C.. ..C..C-..C .--------- ---.CCA.C. ..GA.T.... ...T...TAT A.C...CC..  [ 300]

pvrbsa      ATTTACGCCT TATCATCCCT TTCCCCTTCC ATTAAGTGCG CGTACCGAGA CGAATCTGAC ATATTGTACA GCGACGACTC CAGATCCGAT TTAGCAAGCA  [ 400]
pvrbsap     T....T...G .T..C.A... ......C..T G..C...A.. .C.T..T... ..G....... ....G..... .......... ..C...T... ..........  [ 400]

pvrbsa      ACGGGGCACC CACCGACAGC TACGAATCTT TAACAGCAAG TAGTGAGTCT CTAGCAGAAA GCAACGATGC ACCCAGCAAC AGCTATGAAT CTTTTCCAGA  [ 500]
pvrbsap     ...AC...T. ....A..... ..T....... ..GA....C. .......... ....G..C.. .T.G...... .T.A.C.... .......... .C..------  [ 500]

pvrbsa      AATTAGAGAA AATCTAACCG CAAGTGAGGA ATCCCTAACA TCATGTGAGG AATCCCT--- AACAGGAAGT AATGAATCCC TAACAGGAAG TAATGAATCC  [ 600]
pvrbsap     ---------- ---------- -.GA...--- ---------- --.....GT. .G..G..CGG .G..A.C..C G...C....- --..GAAC.. CTG......T  [ 600]

pvrbsa      CTAACAGGAA GTAATGAATC CCTAACAGGA AGTAATGAAT CCCTAACAGG AAGTAATGAA TCCCTAACAG AAAGTAGAGA ATCTCTAGAG GCCAGTAGAG  [ 700]
pvrbsap     T..T...C.T ...G...G.. ...CGG..C. ....GA.... .T.......A .......--- ---------- ---------- ---------- ----------  [ 700]

pvrbsa      AATCGCTAAG AGCAAGTAGA GAGTCTCTAG CGGCCAGTAG AGAATCCCTG AACGACTTTT GTGGGAGCGA AGAATCAGTA GCATTCGAAG GAGAGCCAAA  [ 800]
pvrbsap     ---------- ---------- ---------- ---------- -......T.. .....G.... A...CG.A.. ......GA.. ....GT.... AT........  [ 800]

pvrbsa      TGAAAAGACA TTCATGGGAG ACGTCTTAAG TGGTGGAGAA TGTGAGAATA GTCTCTCAAG AGAAGATTTA TTTCATATAG AAGTAGGATC CGAAGAATCG  [ 900]
pvrbsap     .A......A. ........G. .T.AA..... GA...A.AT. .C....G... A...AC.... .......... ......G... ...A.....A .......AT.  [ 900]

pvrbsa      CTAGATGATG CCTCAAAATA TAATTTCCAA AAGGATTTAT CAACTAGCGA TAATAGTTCA TTCGAAGATG ATCAGTCATT GAAAAGGGGA CTCAAAAGGA  [1000]
pvrbsap     ...A.....T ...T...... .......... T.A....... .T......A. A.......T. .......... .......... .......... ..........  [1000]

pvrbsa      ACAGCTCAGT ATCTAGTCTG GACAGCGATA TGGGAAGTTA TAAAAATAAA AGTTATAGAC ACAGATTGGA TATATATTCG GACCTACCTA GAAGGCCAAT  [1100]
pvrbsap     .........A .........A ......C.C. .......... .......... .......... .......... ....G..... ..A.CT.... ....AT.T..  [1100]

pvrbsa      ACATGGGGAT AATGCACCAC AAGAAAAGGA GAAATGTCTG GATTTCAAAG AACTTCTAGA GAGAGAAAGG AACAACCACC AAAGGAATAG TGTAAATATA  [1200]
pvrbsap     ........G. .......... ...G.G..A. .....A.G.. ..C....G.. .......... .-..C..... ---....... .......... .......G..  [1200]

pvrbsa      AAATTAAAAA ATATATACGG AGAAGAACAA TTGTATAATG TGAAGAAAGA TAGCTTTATT GTAAAATTGA TATACTATTT GCCGTTTGGC CCAATTGTTA  [1300]
pvrbsap     .......... ....T..... .......... A.......A. ......G.A. .......... .......... .......... .......TT. ...G......  [1300]

pvrbsa      CAATGATAAT GATAGGCTTT ATTCTACTCT GGTCAACGCC CTGGACTGGT TTTATTTTTA TAGGGTGTAG CTCTATTATA TTACCAGCTT TGTTATACGC  [1400]
pvrbsap     .......... ......T... T......... .C.GG..C.. ..C..T.... ..G......T GG...C...A .......G.G A......... ..A.G.....  [1400]

pvrbsa      GATGCATTCA AACAAAAAAC AATTCGAACG TGTATTTGGA AAAGGGAGAA AAAAGCCAAT GAAGGTAAAA AAAGGAGGCG GGAAAATATC TAGATTTTTT  [1500]
pvrbsap     .......... .......... .......... .......... ...A.....C ....A.A..A ...A...... ......A... .A.T...... ..........  [1500]
```

```
pvrbsa     AAAGAAGTAG ACAAATTCTT GTTTGACGAT TTGGATGGTT ATTAATTGAT AAACCGAGTT GGGATCATTT CTTAATGTTA TTCTTATGAA TCATTAAAAT  [1600]
pvrbsap    .....G.... .....C.... .......... .......... .......... .......... .......... .......... .......... ..........  [1600]

pvrbsa     TGTAATTTAG GCGCTTGCTA TCAGTTTGGG AATCTTCTGT TCAGAAGTAA AAGCATGTGA GC-AGTTATA ACCTTTTTTC TGGTTTGCAA ACATGTGTAT  [1700]
pvrbsap    .......... ..A....... GA.A...... G..T..T... ....G...T. ..AAGA.... ..A.A.A... .G........ .C........ .A....TG..  [1700]

pvrbsa     ACACGATAAA TTTGTAT--- ATATGTTCAT ATTTTTGTAG GCAATTCTGA AATAGACTAA TGCCACTAGG ATAGGTTCTA TTTCAATGCA GCATCGCCGT  [1800]
pvrbsap    ...T...... ......CATG ..G......C .......C.. ......T.TT ...G...... .......... ..G...C... ......C..T ......T.C.  [1800]

pvrbsa     CTTTTTTCTA ATAA-----T TTTTTTTTTT TTCTTTTTTT TTATATGTCT TCTGAGGTTT ACCTTTATGG TAGAATTACA CCCTCGCCTC TTTCATTTCA  [1900]
pvrbsap    .......... ..T.GCTTT. .......... ..T.C.G... .......... .......... .......... .......... .......... ..........  [1900]

pvrbsa     CTGCTACTCC ATTTCTCCAT GTTTCTGTAA GAATGATACG CACCTTTTGT GTGCTTCCTA AATTGTAAAT TCTTAATGAA ATACTTCGAT GCACCACTGT  [2000]
pvrbsap    .......... .......... .......... .......... .......... .......... .......... .......... .......... ..........  [2000]

pvrbsa     CTATATTATT AATTTTGTTT TAGAAAAAAT TAAACTTATT TTATCATTTT ATTGAAAATT TTTAACAACA ATGAGATGGC TATGAAACGC TTCATGCGCA  [2100]
pvrbsap    .......... .......... .......... .......... .......... .......... .......... .......... .......... ..........  [2100]

pvrbsa     TGTTTCTCTT TTGAGAAATC TTCGAAGGCG CTTGCTTAGG GTAATTAAAA AGAACGAATC ACCATTTAGA GAATTAAAAG GTGTCCACTT GCTAGCTGTA  [2200]
pvrbsap    .......... .......... ..-------- ---------- ---------- ---------- ---------- ---------- ---------- ----------  [2200]

pvrbsa     TTTTTAATTA TCCAACTGCG TGGCTACCCT TTTTTACACT ATTGCGAAGG ATGGGGG-GA ACTAAGCGTC TAAAACAGTG TTTCATTGTT TGGCAAAAAG  [2300]
pvrbsap    ---------- ---------- ---------- ---------- ----T..... .......AA. .A......G. .....G.... .......... ...A...G.A  [2300]

pvrbsa     --GTGTGTTA TTTTTTAGGG AAACACAAAA TTGCTATCTG GAAAGGCATA TTTAAATGAA TGCAAATAAT AACTAAATTA ACAAATGATT TGGTTTGTTT  [2400]
pvrbsap    GT........ .....C.... .......... .......... ........C. .......... .......... .......... .......--- ....G.....  [2400]

pvrbsa     TTCTCTTCAT TTTGCTCTTC TTATTTGATA TCATAAAAAG GGGGAATTAT TCAAATGTGT AGTGATGCCA TTCTTTCTTT GTGACTA--- ----------  [2500]
pvrbsap    ......G... .........A C.C...AT.. G....T.... .........C .......... .......... .......... .......TTT TTTTTTTTGG  [2500]

pvrbsa     ---------- ---------- ---------- ---------- --TTTTTTTT TGGAAAGGTT AACGTGCGGC ATGGTAATCC AAGTGCCACT ACAGTTAGCG  [2600]
pvrbsap    AAAGTTTGCA CATGCCGGCA TGGCATCCCT GTTAAGTTTT AT........ .......... .......... ........TT .......... TT........  [2600]

pvrbsa     CTAATAGAGT GTTTTTTTTT TTTCACAAGC AGCTGCTTCA CCTTTGCAGG AACGTTAATA TTTGAACCAA GTATGTGGTA GGCTTGCCTC TATTTAGACG  [2700]
pvrbsap    .......... ........C. ...------- ---------- ---------- ---------- ---------- ---------- ---------- ----------  [2700]

pvrbsa     GAACAGCCAT TATGAGAGCT AAAAAAATAG TTTAATTTGT TCCACAGGGA CATGCCAAAC AATTAGAATA GATTTTTTTT TTTTATTAGC AATTTCGTAG  [2800]
pvrbsap    ---------- ---------- ---------- ---------- ---------- ---------- ---------- ---------- -----..... .........A  [2800]

pvrbsa     GTCGCACTTT GATTTGTTTG CCCTAAGAGC CCCCCTGGGG AATCCCAGCG AGCGAGCCTG TGCAGTGTGC TAGCTGAAAA AAAATAAT-- -----AATAC  [2900]
pvrbsap    .......... .......... .......... T..TGC.... ........A. ...A.C..CA ....A..... ..A.C..... .T..A...AA AATAA....A  [2900]

pvrbsa     TAATAGTATT AATAATAGTA ATAAAATTTA GCGAAATTTG TTATTTGAAA AGATC----G TGTTCCAAGG TATAGCAATT TTCGTTCCCC TTTTTCCAGA  [3000]
pvrbsap    C....A..A. ......T... ........C. .....G..G. C......... .T.G.GAGG. .....A...A .......... ........AA ..........  [3000]

pvrbsa     TCTATGACGC GCAATATTTT AAGATACGAA ATTTTTTACG TAATCAATTG TTTCATGAAC A--------- ---------- ---------- ----------  [3100]
pvrbsap    .T........ .......... G.A...T... .A.C...... -......... ...T...... .AAGTGCGAG TTGGTCTTTT AATGTTTTCC TCTTGCAGGA  [3100]

pvrbsa     ---------- ---------- ---------- ---------- ---------- ---------- ---------- ---------- ---------- ----------  [3200]
```

```
pvrbsap    TGCTATATTG ACTGTATGCT CACAACATTG CAATGTCAAG TTGGCAGATA AGAGGAAATT CAAATTGGAA ACCTGCACAT ACATCGAGGA CGCCTATCTT  [3200]

pvrbsa     ---------- ---------- ---------- ---------- ---------- ---------- ---------- ---------- ---------- ----------  [3300]
pvrbsap    CCCCGAGTTT GTTTACATGC CAGACGAACA TTTACATTTT GAGGAGAGTG CGCATTAGCA TGTATGTCAC ATGTATGTAC ATGTATCTAT GGAACAGAAA  [3300]

pvrbsa     ---------- ---------- ---------- ---------- ---------- ---------- ---------- ---------- ---------- ----------  [3400]
pvrbsap    GGGGAACGCT CCAAAGGGTG AACCAGTAGG AGCGCAGCCG CGCAATACCT CTACAACAGT AGTTCCTATG TTATGACTTA AACGGTTTTA TTAAATAGTG  [3400]

pvrbsa     ---------- ---------- ---------- ---------- ---------- ---------- ---------- ---------- ---------- ----------  [3500]
pvrbsap    CCTTACGGAA GGTGGAATGA CAAAAGAAAA AGCTTCCTTT GGCAGTGTTC TTGAAAATGA AAAAAGATGA ACCGCGCTTT TATAACGATG TTATGGTATT  [3500]

pvrbsa     ---------- ---------- ---------- ---------- ---------- ---------- ---------- AAGTACCCGT TAGGTGCAAA TGAAGTTTTT  [3600]
pvrbsap    AGGAAATGAT TAGATCTGTT CAGACTTTTC CTCCCCCCCC CCCTCACCAC CACCTTTTCT ATTCATGTAC ........A. C...C..... G....C....  [3600]

pvrbsa     TCCTTCGGCC AAAGCAGGCG CAACATGCAT GCATATATCC TTCTTTTTTT TTTTTGGAAA AAAAAGCGAA ATGTACATGG CACAATTTTT GTTGTGCAAA  [3700]
pvrbsap    .T........ ....GG..T. ....G..A.. A....-..G. A.A.CC.... .......... .......... .......... .......... ..........  [3700]

pvrbsa     ATGCAGTGGA ACTTATTGTA GGGGAAAGTT TAAATATGTC ATTTGGGGGG ATTAATTGCG AGCCGTTTGA CCATTTAAGT TTGAAAAATT TTAGGCGGTT  [3800]
pvrbsap    ...G..A... .......... ....G..A.A ........C. .......... .....C...A ........A. ........T. ........A. ..........  [3800]

pvrbsa     TTACCCGCGA TGTGAATGTT --TCTCCCCT TGAGTTTTCT TTCTTTAATG TTGAGGGAAT TATTTACGAG GGGGAATTGA TTTTTGCAAA CTTAGAAGCA  [3900]
pvrbsap    ...T....A. A......... CC........ GA.AG..... ......T... ....A..G.A .........A ....TG.... ........T. ..C.......  [3900]

pvrbsa     TCATGCGTGT ATGCATATAT AT-GTATAAC TATGTA-GTA CCTAAATTGT CATGGTGTAC CGTGCCGCGC CGCGGGGGGA GTGGCGGCTG GGACAACA--  [4000]
pvrbsap    C.....T... .......... ..G......G ......G... .........- ---------. .....T.TA. .A..C..CC. ........A. A.......AT  [4000]

pvrbsa     CTTATGCGTT TAAAAAAATTA TTTATCGGAG CACCATAAAT TTTCCCTTTG TTCAGGGTCA TTCTGGTGCA GATAACCAAA CTGAAAATTA ATACGGTGTA  [4100]
pvrbsap    T......... .......... .......... .......... .......... .......... .......... .......... .......... ..........  [4100]

pvrbsa     CATATATGAA ACATAAAAGT GTCTTCTCTT TGTAAAAGCT GAGAGTAATT TTTTTCGCTC TACCAGGAGA TTACGATACA GGCAAGAGTT AAGTTAATAC  [4200]
pvrbsap    .......... .......... .......... .......... .......... .......... .......... .......... .......... ..........  [4200]

pvrbsa     TTACGTTGAT GTCAACATCG GTGGCAATAT TAGTAAGATG AACGAAAGCT TGCTCTTTGC TTCGTAATTA GAAAGATAAA AAAAAAATTG CGCTATTAAT  [4300]
pvrbsap    .......... .......... .......... .......... .......... .......... .......... .......... .......... ..........  [4300]

pvrbsa     AAATATTCTA AAGGAGGGGA TATACTTAAT GAATTAACTA ATGCATGCAG AATTATCCCT GTTCCTCATT TTGCATTTTG CGAAGAACCT TTTGGTTCCA  [4400]
pvrbsap    .......... .......... .......... .......... .......... .......... .......... ..A....... .......... ..........  [4400]

pvrbsa     GCAGACGGGG GGAAAAAAAA CAAACCCTTT GAGAATAAAT TGTTTTCATT TTAATTAACG CCTTTGCTTT ATTGAAGTTT ATAAATGTAC ATAAAAATAT  [4500]
pvrbsap    .......... .......... .......... .......... .......... .......... .......... .......... .......... ..........  [4500]

pvrbsa     GTAAATATTT ATTCATTTAG AAAACAAAAT TGGCGATTTA TTTTTCAAAA ATTAAATTGC CCTGTTACAA ATAAGGAGAA GAAAAAACGC ATTAATTATT  [4600]
pvrbsap    .......... .......... .......... .......... .......... .......... .......... .......... .......... ..........  [4600]

pvrbsa     CTCTAAATTT ACAAATTTTA AACTCGTATA AGCGCGTACC GATTTAATTT GCTGGGAAAT TAAAAAAAAA AAA [4673]
pvrbsap    ...AGC.... .AC....... .......T.. .......... .......... .......... .......... ... [4673]
```

**D**. Alignment of the 10,000 bp *P. cynomolgi* duplicated fragment

```
Pcrbsa      AAGGGTGTAT TTTTTGTTTA GCGAAAAAGG AGAAGTGATC AAAAATAAAG TAAAATTATT ATATAAATTG TATAAAAGTG ATATAATATT AAACTTTAAA  [  100]
Pcrbsap     TT.AAG.C.A CCACCA...G .G.....T.T -----.TTC. ....T..GCA .TT....C.. -.....--.. ATG..CC.C. .AGG.GCTG. G.TTA.GCG.  [  100]

Pcrbsa      TAAAAAAATT ATTTTAACAG GAAGGAAAAA AATGATGCCT CGGGGTATAA TGGCAAATTT GTTAGAAATA TTGTATACCC -TCCAAACAT ATTATGCTGC  [  200]
Pcrbsap     CTTTTC.T.C G....GTG.A A.TT.....G .TGAG.A.AA .T..--.AGG .CA..CT.G. .CGT.GTC.T ..TCC.GT.. A.TTTGG.T. CC.T.T..AT  [  200]

Pcrbsa      GATCTCATGG GTCCAGGCTA TATTC-ATGT ACATAATTAC ATAATTTACT TATTGATTAA ATAAATTATT AGTGTGTTAA GTGAATTTGT AATATTCTCT  [  300]
Pcrbsap     T.A.A.T.TT .CT..T.TA. CG..TT.... GG.GCGAGC. .CTC.....C G.A.A...TT CCT....T.- -..CG..ATG C.C..AG.C. ..GT....A.  [  300]

Pcrbsa      TAATCATTGA CGGTTACAGT TTATATGCTT CCGTAATTTA ATTTTTATTG CAAAAATAAT GCATATATTT ATCTTTTTAA ATATAGAAAA GCCCATAAAA  [  400]
Pcrbsap     .TT.TGA.A. TTT..TA..- --.....--- ---...G..C T.CAGGGG.. T.T.T..... ---C..CC.G T..C....GT TCC.CTTCTG TTTTG.C...  [  400]

Pcrbsa      ATATAATTTC GAAGTTCTTT TTTAAATAAC ATTTTCGGGG AAAAGCCAAC TTTTTTTTTG T-TATGGGCC TTCTGGTATA TTTTATTTTG TTATTTTGAC  [  500]
Pcrbsap     G.T--...CA .GTA..G.A. A..T..ATCT G.....A..A ..TTATTG.T .....C.GCT .G...AA.TT .ATGTT...T .A.A....C. ....A..TGT  [  500]

Pcrbsa      AC--GCCTTG AGGAGAGTAC TCTTTCATGC GCAA-AAGGA ATAGTACAAT ATAGAGATAG AGGATGGTGT GTTCGGGCCT TTTTTATTTT TTTCCCTAAG  [  600]
Pcrbsap     .GTAATA..T .TCG..AC.. CT.C.GC.AT ATTTT.CT.G G..C.GTGG. ..TATTT..C .TA.------ .C.TAAAA.G .A...CA.G. GG..A....A  [  600]

Pcrbsa      CTTAACTTTT TTGAAAACTC GATTACTGGA AAAAATTTAC ATTTGTGGGA GTTGAAAATT TTCCATTTCG CAGCCAATAA AAGTATATAC GGAATTTAAT  [  700]
Pcrbsap     G...CAG... ---------- -GC.G..A.C C........A C.C.A.TAA. TC.T..---- .CTA.....T T------... T.T.T.CA.. ATTG..--..  [  700]

Pcrbsa      TTTTATAGAT CTCAAAAAGG GGGACAGAAA CAAACAAAAT AGATTTTATC CCTGTCAT-T TTCTAGTAAA ATGTGCACGT AAAATTGCGC GCTTCTTTAC  [  800]
Pcrbsap     AA.AG..ATC A.T.TTCG.. TT.C..ATGT T.T..GTGT. TA.....TC. .T.A.AT.A. ..T..T.T.C ..A..TG.C. ..G.C.T.TA TG.GT...C.  [  800]

Pcrbsa      ATTAGCATTA GCAGCTGTAG GTAACTATAC GAACTGAACG CATACTTTAA TCACTAATTG GAGGCGTGGG AAAATACATT TTCTATGCTC TTTCGTTCTC  [  900]
Pcrbsap     ...TTTC... T.TTT.TG.T T.GC.ACA.. T.TT.TG.AA A..CT...CC .TTT..---- ----.ACC.T TT.T.TGGCA .CT...T..T GGGG.GC..T  [  900]

Pcrbsa      AAATTTTGCA AAAGAATAAA AAAAAAGGCC ACCCAAAGGA GGAAGTATAA CATGTAAAGG AAGCATGTAA GAATATGCAA GGAGTTCATA TGTAAGCGGT  [ 1000]
Pcrbsap     T.TC....G. ....T..GGC .....TA... T..TT.TC-- --.TT.CA.G G...C.G..A ..-T.AAA.G .T....-T.T .ATA.C..GG ..AT.A-.AA  [ 1000]

Pcrbsa      GATAGGCTGT TAGTATGTCT TCTTAATCAC GCATAAGTAA ACAAAACGTC GTTATATCCT TCTATTGAGG ATAAACAAAA TGATTCATTT ATCCATGCGA  [ 1100]
Pcrbsap     A...AT.A.. GTT.T.T.T. .T.....-.T A.......TC .A.G.GA.GA A..---.TT. ..ATG..G.. G--------- ------.... ...A------  [ 1100]

Pcrbsa      TTTATTTGCA TTATCGCCCA CATTTTTTTA AAATGCAGAA TGTATACTTC CCTGGATGAC TGTTTGTTGT ATTCTGTACA TTATTTTATT GCACATGTTC  [ 1200]
Pcrbsap     --C...CAT. A..AT..TG. A-----..A. .....-.A.. .A.T.GAG-- -..AA...GG .ACA.A.GT. .AGTGAA.A. A.CAA..T.. ..G...AAAA  [ 1200]

Pcrbsa      CTAACAACTC CGAACACGTT CAACCTTTTT ATGAATTTTA GTTTACCCAT GAAAAGTAAA AAGTTTTGCA TTGTTACAAA TGGGTAGGTT GTGCAGCCCT  [ 1300]
Pcrbsap     A...TCTA.T TAG.GGAA.G A..TT.AAA. T..G.C...- -..CT.AT.. .CTT.ACTGT G..G.G.A.. .----.T..T ..C.CT--C. ....TATT..  [ 1300]

Pcrbsa      TTGAGCACTT GTGAGTATAA ATTTTTTTTT CTTAAATTTC AGGTTTGTCA CACTTTTACC GCGTTTGGGA CAGCGCGGCA CTGTTTTATT TAAAAGCGCT  [ 1400]
Pcrbsap     ..-------. CAA...GC.T ....AAG.-- ---....G.T TT...CT..C TT.G.CAGAT ...A..T.A. T.---T.TT. AC....---- -------..A  [ 1400]

Pcrbsa      TTATTAACTT TGGGTTCATA CATTTTTTTC ACCTTCCTAT GGTGAAGAAA AGTCTCCATG ATCTTATTAC CATTTTGACA TATTATCTCA ACTGCAAATA  [ 1500]
Pcrbsap     .CGA.T.... .-------.. TT.......T .AT..TT.T. CC.AGC.T.. .C.-..A.AA G.TGCGC.TG ...----..G A..CGG.AT. .A.A..T...  [ 1500]
```

```
Pcrbsa      TGAACGATAA AAATTATATT CTATGGGGCA CAAAATGCCA ACATAACACA TGTATAGGAG GAAAAGTGCG GTGATAGATG ACACATGCTT G-GTTGCGCC  [ 1600]
Pcrbsap     ..T..CC.TG C.TG.GC.CA T.T.T..--- ..GG..AGTC ...C------ ..C.CGA..A .C.C.T.T.T ..T.C.C..T .ACTGC...C AA...TTT..  [ 1600]

Pcrbsa      AAATAGTGAC ATTAAAAGGG GGGATGGGAC AGCATATTGA GACGAGGGAG AGGTATAGTA CTCCTCTTTT ATGCCTTAAA AGGGGAATGC TCATTTTTAG  [ 1700]
Pcrbsap     CC..TTATG. ..A.C..AAT ----..AA.T .A.CC...-. C.TAT.T..T .---..T..G .CG..T.... T.C.G.---- -.A...G..A GTT.....--  [ 1700]

Pcrbsa      ATCAATTTTG ACCAGCTATA TTATATGTAC AAGTTGTTAA GAAACAATTT CTGTATATGA GTGAGTAGCA CAGCTGACAT GTTTTCCCAG TTTTGCCAAA  [ 1800]
Pcrbsap     -----..C.. CG..A..-.. C.G.T.A.TA ..AAGA.... T.GGG..G.C GC.C---CA. ....AAC.T. -----....C .C...ATTCA .C..ATA.C.  [ 1800]

Pcrbsa      AAAAAAAACA AAAACAAAAA TAACTACTCC CTCTTTAATC ACCCCTGAGG GGTTTGTCCA TACGCTTCTT CATTTTGTGA GTGTACACTA TTTTATGTAC  [ 1900]
Pcrbsap     ..TGC...TG GG.GA..... .G.T.GA--- ------.G.G .AGT..T.TA .A...AAGA. G.A..CC.CC G.C..GA.-- ----.TC.A. G....A..-.  [ 1900]

Pcrbsa      AATTTGAGAG AGAGGCCAAT TA-TGATGTT ATTTTTTTTC TAGGTTCAGC GCGCGCTACG CTGTAGACAC ATTTTTATGA AGCAGACAAG CGCGAACTGC  [ 2000]
Pcrbsap     .GAAG...G. .TCA...... CGGCC.C... GCGC..CCG. ..T...GC.. TTC.T...T. T..------. GC..CCGCC. T..T.-.GCT TC..CCA..A  [ 2000]

Pcrbsa      TCAGATGTCT GGTTAGCACA TTATGTAAAT GTTTTGCTTG TTGGGGTGAA GAAAAAAGGA GGTCAGCTTT TTTATCAAGA CAACGAGGGT AATATATACA  [ 2100]
Pcrbsap     .GC.T.AC.- AC..T..TT. .CC.ACG... AA---.TACA CCCCC...T. ACTTCTGA.C C.CTT.T.AC CCC-....T. T..TAT.TT. T---...TT.  [ 2100]

Pcrbsa      TACCTTGTTT CAAGAGAGTT TGGCTCTCTA AGAGTAAGAG GA-TTGGCGC ATAGAAGGAG CAACTTAATA GAACATGCAA GAGCAACAAC TT-AACTGCA  [ 2200]
Pcrbsap     .G.TCCT... ..GAGTGA.C AAAG-----. ...C.GGT.C ..C..C.TAA .ATAC.T... .G.T....-G .......T.T A.ATGT.C.. .CC..A..A.  [ 2200]

Pcrbsa      TTGCAATGTT CAAATAAAGG GAAATAACAA TCCTGTTTTT ACGTTTCATT TGGACCCACC ATTTA-GACA TAACCCCTCC CCCCACTAAA GCTGCGCACT  [ 2300]
Pcrbsap     .G..C..TG. A..GAT.TAC ATGG.G.AG. ..A.A..A.. ..TG.GA..A AAA.A--G.. .....T..A. AT.TA.A.GA TGTA.TG... AT.AAAG.G.  [ 2300]

Pcrbsa      TGAAAAAGAG GCTCCATTTA TGTTTTATAT TTCTCAACGT GTGATATAAT GAGATGGCGT TCTAAGTAAA AATCTGTTCC TAACCCCTTC GTTCATACTT  [ 2400]
Pcrbsap     G...C..TGA ----...AG. -A...G..GA C---..T.C. A.C-.G...A ..TTCCT.T. CGG.TTC.TG T.G.CA.... G.GGAGTC.G ..A-GCGT..  [ 2400]

Pcrbsa      GGAA---AAA AAAAAAAAAA AAAAAGCGCA ATATGATACG AACAAAGGTT AGCAATTCTT TCATAAATAG GGAAGTCGTG C--ACCAGGG GAGTCTTAAC  [ 2500]
Pcrbsap     AC..TTTT.. C..GT.T... .T..TT.A.. .C.AC...TA TT.C..TAAC GC.....--. GA.G.GGC.A AA..T..A.. .GA.TT.CA. A...A.ATGT  [ 2500]

Pcrbsa      AATTTAAGAA CATAGTAGAG CTAAAATTAA GCGGCTCTCT AACCCAGTGT GTGCAATGTC TAGGGTTAAT TATTTTCAGT AGAACTGAAC CCTATTTTTC  [ 2600]
Pcrbsap     .TACC...GG A.A..C---. .G..GT.A.. .A..AGAAAA ..AAATA.AG CCATG.AA.A .GCTA.A..A G.AGAA..AG .A..AAA... ---...C..A  [ 2600]

Pcrbsa      GTGGAGGTGA TATTAAACTT AAACGTTGAT ACATTAGGGG AGGAGAAGAG TGCAATTTTT TGGTCTTCAA AAAATTATCG ACACATTAAG GATGCCAATT  [ 2700]
Pcrbsap     ACA.CTC.TG .GAC..---- ------.... GA....AATA .CA....C.A .AA.-----. .A.----... .....GT..C .GTTG..CCA A...--.GCA  [ 2700]

Pcrbsa      ATACAATGTA CATCCCCTTT TGAATGCCCT ACAAATGGTT ATGTCGAGTG CGCTAATTGT TATGTGCAGG CTGAGGAGCG ATAAGGCAGC ATATGTATAT  [ 2800]
Pcrbsap     G.GTG..C.T T...A---.A AA..GATTT. G.GT.G.TC. T.AA.C.AAT TAAGC.GGAA C..T.TTTAC .AA.AA..TA .A.GTT.G.. ........--  [ 2800]

Pcrbsa      TCTGCCAGAG TGCATGTTTT ACCACTACAT ACACGTGTCT TTTTTTTTTC GTAACGATGA TACTTCTTAC ATTGGTGTGG TGAAAAC-AG TGAGAATGTA  [ 2900]
Pcrbsap     C..A..C.GA ..A..----- ..G.T..T.C .T...G---- -------..G .A..GA..AT G.TG...CTT ..GACCTAAA ....T.TG.C CT...G.C..  [ 2900]

Pcrbsa      GCGAAGGGAA C-ATAAAAAA AGTTTCCCCC TTTATAATGA CTCATCGCGA CATTTTCTAC ATACAAAAGG GTATCCCTTC TACAGCAAGT GGAGAAGCTA  [ 3000]
Pcrbsap     A...TTTTGT .G....T..T GAAAGT.AGA A...C..CA. G.TG-...A. GGG.ACA..T .A.G....A. -.GGA.AACA GGA..A...A CA.A.G.AG.  [ 3000]

Pcrbsa      ACCCTCCACC CCCAACCCCT CCCATGTTGC TTCCACTCGA GCGATCGATC CCGTTCGTTG CTGCTTTTAA AA-AATTCGC GTTTTCCCAG TTTTCAAAAA  [ 3100]
```

```
Pcrbsap    .AA.GG..AT ----.TTTTA ATA...A..A G.TTT..... AGT.A.AG.. TTAGCAA..T A.CTG..C.. G.T...ATTA ..GCAAATGA .G.G.C....  [ 3100]

Pcrbsa     AGGGAGAAGT CTCACTTATA TAAACTCGAC ATTTTATGGT GGGAGGGAAT CCTATGTGAG AACTCTTTAC AGCACATTTA CCGCGCGCCA GCGAACTTAA  [ 3200]
Pcrbsap    GA.A.A...A G.A..A.--- ---------- -....G.TAA .AA.AAT... AA-..A.... GTG.AC.C.- .A.G..G.A. AT-------. AT.C..A...  [ 3200]

Pcrbsa     GGAGGATAAA CGCACAAAAA GCACACACTT ATTTTGTGTG CACCAAAATG ATGCATCTCC GAACGTGCCT CAAGAGGTGC CTGGGTACTC TTTTTTTTTC  [ 3300]
Pcrbsap    AA.T..---- .T.C.....G .TGAGT.AGC ...CCA---- ----....GA ....-----. A..TT..A.G -...CA.CA. AAAACA.--- -------.C.  [ 3300]

Pcrbsa     TCACTTCTAA AAATGCACAA GCGGGACGAA TGTAAAAGAA GACCCTCTCT CGGCGGCGCT TATAAATGAT AATTATATAT AGCTCCACTT TCTCCCCCCA  [ 3400]
Pcrbsap    A.....TG.- ---------- -...AT..T. ..CGT.TA.- --TTT.GC.. ATAT..A--- ..A...C... .GA.-C.GG. G....T...C C---------  [ 3400]

Pcrbsa     CAAAATGCTA AAAATCGATG TACGCAAACG ATCCTTATGC ATTTGAGCTG GAAGCAACAA GGCGCAACAA CGTCCTTTA- -GGAGGAAAT TAAAATGCGA  [ 3500]
Pcrbsap    ---.G..--- -G..G..G.. C...-G...T ..---...A ....AGA.-- ....A..T.. AAAA.T.... TT.T.GCCGT CAT...TG.G CGC...TT-.  [ 3500]

Pcrbsa     ACAACGTTCC TCTTTATGAA AGAGATATAG CAGCAACGTT AACTCCATAA TAACCCGGAT AGAACACTTA TTGCGACAAA TAAAAAAATG GAAAATCGAA  [ 3600]
Pcrbsap    G...-...TG CT...GCT.. GA.A...AGC A.ATGTG..C G.AC...-.T .GGG--..GG G.TT....GC ...-..T..- CGC.CCCG.. TTTGT.G.TG  [ 3600]

Pcrbsa     AATGGGCAGA TTAAGACTTA AAAAGGGGAG GCGGTAAATT ACAAAGTTCC CACTCCTGGT AATGCATACA ATACACACAA TATGTGTGTT GCGGAGGAAG  [ 3700]
Pcrbsap    T...T.T.T. CGTGC..A.. TGC.AA.A.. -----..G.- ---.G..G.G .C.CG..T.. .CA.TGGG.T .GGTTTT.CC .T.T.TCT.. ..AC.AA..T  [ 3700]

Pcrbsa     AGTGGTCATT TTTTAAAATC GTTTCGAAAG AGATATATGA ATTGTCTCAT CTCTCACAGC GCACGCAGGG CAAAAA-AGG CATAAACG-C CATATTACGT  [ 3800]
Pcrbsap    G.AACA..A. ...ATG...T ..G-.A...A .T...GGGA. .AGA.A.A.C AAA.T.A.AA AA.AAA.AAA T.GG..G.TA ..G...AAAT T.C.AC.A.C  [ 3800]

Pcrbsa     AGAATTTAAG AAAACGAAAA AGCAATCCTC TACCAGAGGA TAAATTACAT GCTTCTTTAT GCACATACAT AGCGTGAAGC GCAG-CCAAA TACAGTGAAG  [ 3900]
Pcrbsap    .T.CA..C.C .GC.T.C..C .AA..CA..T CTTA.AC.AT .GTGG.G-G. .G..GGGA.. AA.TC.G.GA ..G.A...AA A..AAT.GG. GTTGCCA.CA  [ 3900]

Pcrbsa     CGAAAGGATT CACAAAA--A CAAAAACAAA AACAAAAACC GAATTGAAGC TAGCCATATA ATACAGCTCA CATGTGCATA TCCCCTCTCT GCCACCAAGC  [ 4000]
Pcrbsap    AA......GG ...TCCTTC. TG.G..T..T GTTGC...TT TTG...CG.. CCAT--.T.. ..GTG.TGTG A.AA.TT-.G CTAAAAT.T. ....A..GAT  [ 4000]

Pcrbsa     CCTGGCAACT TCTACGCCTA GCTCCTTTTT CTTTTTATCG CCAACCGGTT TGAT----TT ACTCGACCAG GGTTAGAGAT CCTTACACAA GTTTATTCCA  [ 4100]
Pcrbsap    .GC.-T...- ---.G.GT.G ...T.CA..C G.GA..T..A TA..GGTA.A .A..ATTG.. G..AATAT.T ATGG.A.... TG...AT.G. A.AG.C.A..  [ 4100]

Pcrbsa     TTAATATGAC AATATCTGTC ATGCGTTTTT TTACATAGAC AGAGAAAAAG AAGGGTATAC AATGTGGA-A CAACGCATGG ACGCACAGTC ATAGGCGGTT  [ 4200]
Pcrbsap    ..TCCG.T-- --...T.TA. GAT.A..... ..G..----- ------...A T...A...-. .T.T..T.CG ...TA.G.TT CTAT.T.A.T C.TTA..T.C  [ 4200]

Pcrbsa     TTACTTTACT ACAATTTGGG GGTGGGGGTA ACTGAAATGA ACCCTCCCTT CCCCAAGAAT GACATAACGA CGTAAAGATA TTTACGGTTT GAGTAAGTAC  [ 4300]
Pcrbsap    A..T...CG. -..TC...C. A..TT---CT T..AT.T... TTTACGT.A. .TT.T.TTT. .TTCATCAA. TTG.C.T..T ...T...AGA A.A..CA..G  [ 4300]

Pcrbsa     CCCACACGAT GATGAAGGGG AAGAGCGAGT ACGATACGAC ATGAACATGC AACATATG-C AGCTGCTCAT TGGTACTATC TCCCTATGTG ACATAAGCTG  [ 4400]
Pcrbsap    .T-..TTTT. ...TC.TCT. TCAT.T..T. TTTTCTTACA T..GG...AT ..TT...TT. .AA.A.CT-- ---..T..AT ..TT.G..GA ..GGCGTAAA  [ 4400]

Pcrbsa     TACAATTTGT GAACACGCGT GGCAGTAATT CACTGTGACA TGAAAGTCAC ACACATTATG TGAACATGCG CTGCGTTTCT TTCCATTCCA CGTTACGCAC  [ 4500]
Pcrbsap    G.GG.AC..- ...TTTTTA. AT.CT..TA. A.--..A.T. -.T..T.T.G .T-T..C..C ......AATT T.A.T...G. CAAAG.CTAG .ACC.TTT--  [ 4500]

Pcrbsa     CGGATGGGAA GAAGAGGGTT ATTGCCTGCC CCATTTATGT CTACCAACTG TGACCAACAA AAAGGGGTGT TCAGAATGAA GGACGATTTG TATAAATACA  [ 4600]
Pcrbsap    ..ACG..... .GTA.CAA.. T.CT.T...A AT.GG..C.. T.TT--.TCA CATTT..T.- -------..C .G.AC....- ---T.T...T ...GG.ATT.  [ 4600]

Pcrbsa     CATTTCGTGA AAT-ACGCAA TGGATTGCAT ATTAATAGTT TATGGATTTT ATTTACTCGG AAAAGTTTTC ACCTTTTATT AGCAATGTAG ATCTCTGTGC  [ 4700]
Pcrbsap    TC....A.T. ...T.TTTTC AAA.AC.TC. T.C.CCTT.. ..AA.G...C G.AA.T.TCT -----....T TTTA...G.. ....T.A--- --------..  [ 4700]
```

```
Pcrbsa     TTTCTTAGTT CTCTTGGCAC GTCACATATC GGAAACGCTT ACTTTTTACA CAATAGAATG AACATCTGGT GAGGGGAAAG CGGAGGGGGG GAAATCCCCT  [ 4800]
Pcrbsap    ...T...... .C-------- -..G.---.T ..G.CGC... C..C.GG.GC A..G...GCT TC...T.T.. .C.C.CT.TT TTTTA.---- -----TT.A.  [ 4800]

Pcrbsa     TCTTCTATAA CATCAGAAGG GTAAGTAAAT AATGCAACAT AAACTTAGTT AAGCGCAGCA AATTGAGCAC ATGCTTCGAA TTATACATTT TAAATGTAGC  [ 4900]
Pcrbsap    .T..T.G..- ...AT..C.C ..---...GC TC.CTC.G.. ..T.AC.--. .C.T..TT.G ..------.A ..TG.AAA.. AA...T.C.. ....GA.GAG  [ 4900]

Pcrbsa     AATGCTGATG TACATACATT TTATATACGC GCGTATGTAT GGTTAATAGA AATACCTTCG AAT-GTATTA AAAGGGGGAA TAAAAAAATT GCGCTGATGC  [ 5000]
Pcrbsap    ..A.AC...- ------TG.G .CG.C..... A.T.G....G TA.C.-..AC TT...GAAG. CG.T.C.CA. ..TC.TC.TT C.C....TC. AT.TCA..TA  [ 5000]

Pcrbsa     ATGTACAATA TGCTTGAGCG AATATATGTA AAGCCTCAGT TAAGTAGCAC ACTTTTTTTT TTTTTTTTTA CGTATTTACA TGAGTAATAA AATAATATGA  [ 5100]
Pcrbsap    ..TC....AT .ATCC----- --.T.T.AC. .-----.... .TC.A.AA-- -----...CC .....G.A.. TA.-...C.. .....GC... TT.G..--..  [ 5100]


Pcrbsa     CGCCGCTCTC GTTTTTTTAG CATGTGACGA GAAACAGGCA CAGCCACGGT GTGTTATATG TACATCGCAC AAAATGGTAC AAGGGGATAG AAACGGCATT  [ 5200]
Pcrbsap    GTT.TA.A.A A....C.G.T AGATCA.ATT TTTG..AAT. GG.AA.---. A.A.A..G.. .GT.GTATCT .G.TAA..TG G..TCA..GT C..-ACTG..  [ 5200]

Pcrbsa     TATTTATTTA TCTATAAGTT GGCTCGTTAA GTTAGCGCAC CAAATAGGGG TTAAATTGGC GCCTCAAATT TCGCTTAACT TATTCATAGC ATTGCCCTTA  [ 5300]
Pcrbsap    .G..A..A.- CAA.C.GAA. ..A.A...TT T..T.TA.GT .TTG...A.- ------..TG TT.CTGT... ....C----- -...TTCGAT G..TT..--.  [ 5300]

Pcrbsa     CGGCTAACAT TGCGCTTAAC TTAAGACTCG TTCGATTCGC AAATTTTAGC AAATTTTTGT ACATACAAAC TTAGAAAACA ATTTATAAAA TAATTAAGAG  [ 5400]
Pcrbsap    ..TTGG.... .ATT...TTT .C..T.AAAA G.--G..AAT .T.....TAT ..TA.C..A. .TTG.TGGCG .CCTT...A. G...T--... .G...GTA.A  [ 5400]

Pcrbsa     GTAAAGAAAA TTTT-----A CATTAAAAGG AGTTAACCAA TTCGCAATA- ACCCCCTCTC CCTGTTTTTT TTTTTTTTTT TTATATAGTA TGAGAAAAAA  [ 5500]
Pcrbsap    ..TCT.G... ....TTCCT. T.CA.TGC.. G..AC..AT. CGTAT.G..T .TTATG.TGT .AAA...... ..CAA..... CG....TC.. .C.T.CTGTT  [ 5500]

Pcrbsa     ATAGAAGAAA CAAAAAAATA ATTATTTTTT AAAAATAGAG GTACCGTAGT ACTGCTATAT TATACTATTT AAAAATATCT ACAGCAAAGT AACACACAAA  [ 5600]
Pcrbsap    T.GCTCCGCT .CC.TT.G-. .CA....... TT..GC.--- -..TTA..-. .T.-T...G. C..TT.GG.. ...CG.GGG. TGCTTGTTT. GG.T------  [ 5600]

Pcrbsa     AAAAAAAAAA AAAAAAACAC ATGGGAGGGA GGGAAGGGTC GCTAGGAGTA TAATAAATAA CTTATATGGA TTTGGTATAG CAGTATAAAT ATATATTACC  [ 5700]
Pcrbsap    ---------- ------G.T. G..T....TG ...CCT.T.. A..G..G.A. ACT.CTTC.T .C.T.GC..- -.C...---. ..T.T.GCG. -.GCT.CG.T  [ 5700]

Pcrbsa     TACTACTACC CTATGCATGC ACACTTAAGC AAGGATTTAC GGATTTAATA TTGCTAAAAA ATGGTGAATT TTTTTTTTTT AAATCGCAAA AAAATTTGGC  [ 5800]
Pcrbsap    GTGAG..T.G .G...T-..G GAGT..TCCA ..CTT.C.GA .CT...CGA. ..TTCCG.G. T.TTC...C. ..CC------ ---------- -G.....TT.  [ 5800]

Pcrbsa     AAACCTTTTC TTAGGTGCGC AAAATTGCTC AGCCGCTACG TAGTATTAGT TTAACAATTT AATGTGCTGT TTGGCATATC CCACGCAAGT AAATAAATGA  [ 5900]
Pcrbsap    ....--.... CG..A.TTT. G..C..T.CG .A.TTT.--- ---------- CA...T..CC G.GA.----. ..C.A.CT.T ..GAA.T.AA ..T.TTT.TG  [ 5900]

Pcrbsa     ATGAATAATT TCAATATATT ATACCTAATC CTTATCTTCA CCCTTTCTCT GATCAAATAG CAAAGTTTTA AATAAAATAA AACCTAGCAC AGCGAATAAA  [ 6000]
Pcrbsap    --....TT.C C...AT.G.C G.G.T.GC.- ----...... A.G.CCT.G. C....C.G.. A..CTG...C TT..TT.-GT TC.T.GTTGT T.T...A.T.  [ 6000]

Pcrbsa     AACGAAAAAA AAACAAACTA ATAAAATAAA AAAATCGTTA AAAATTACAT ATTTATTTAT GTACAATTTT --ATTATCAC TATAAAAAAG TTCAGAAGGA  [ 6100]
Pcrbsap    ..T.CG..GG ..CTCC.--- -------.G. ....C...G. C.CGCGG-.. G..C.GA..C A-.G....GC GA...T..GT A.AT.....A .A....G.T-  [ 6100]

Pcrbsa     AAGAAAAAAA AAACTAAAAT CATCACAAAA AGTTTGCTTT CTTCAAAGGG GGTTTTTACC CGTTTAACTA AGAAAAAAAA TTATTTTTTA AATCCCTTCG  [ 6200]
Pcrbsap    --.T.C.GGT C.CTC..G.G T.A.G---G. TC...T.CC. T.....TTTC .A.A...TTA ATG..T.T.. .ATCG..T.- -.G.G..G.. .G..ATCCAT  [ 6200]
```

```
Pcrbsa      CTTTTTTTTT TTTTAATGGA TATTATGTAT ACATTATATT ATAATATATT ATTATACTAT ACTATCATAT TATATATTTA CATAATGGTA CATATATCAC  [ 6300]
Pcrbsap     .G....G.A. G..CC.---G .G.A....GA .G.----.GC .CCGAC.G.. T.GGGGGC.C TT.TGA.GTA .T.G.T..GG ......A.CT T...------  [ 6300]

Pcrbsa      AGTGCGTACA AGTACAGTAC ATTTTTTTAT TTATGCATTA TTAACGCTTT GTTTTTTTTC TAGAGATATC GTTTAGAATT TTTTTTTTAA TTATTTTTGT  [ 6400]
Pcrbsap     .T.A.T.GTT .A..A.T..- -...AGC.T. .CT.CA.AGG .G.GA.AC.. AC.A.AG..- --...G.CC. ------...A .G....G.TT CCC....C.C  [ 6400]

Pcrbsa      TTAAGCAAAC CCGTTTTATT AAACAAACAT ATTCATTCTT CTCATTTAAA ATTTTAACAA TTTTTTTTTT TAACATTTCT CCTTTACCAT TATTTTACCC  [ 6500]
Pcrbsap     C.GCC.---- .TCC..CGC. .T.TGGGTTA G..A.A.GC. TGGG..GG.T ....-.T... ...CG.GG.. C----..C.A .....GGGGG AG..AC..A.  [ 6500]

Pcrbsa      GCCCTCTTCA CCACAGGCTT TTTTACGCAA CACAATTGTT ATCTAATACG TTAATGCTAT TATTATCTAT TAAATAAATG ATAAGAAAAA AAAAATATAT  [ 6600]
Pcrbsap     .TAT.GA.G. ..C.----.. GGG.GT...T .C.C..CA.C ..-------- --......CA GCA...TG.. C..CGG.... ..C..C.C.. TGC------.  [ 6600]

Pcrbsa      AAAAAAACAG AAGAAAAAAA TAAAATAAAA AAAAAACTAG CATTGAAATA ACAAAGTTAT ATATAAACTA AACCCAACGC AATCCATCCC CCTAAGCGCT  [ 6700]
Pcrbsap     C.GC.TTTT. .TC.GCGCG. .----.GCC. CC.TC.TCTT .C.CT.TT.T G---..C.-C ..G.CGCT.. .C..T..AAA GG.ATTCATT AAACGTT..G  [ 6700]

Pcrbsa      TTTTTAATAG CCCTCCAATA AGGCGAAACA TAATAAAACT AAATCTTTTA TTTACTACCT AGTTTATTAA CTCATTTTTA AAAATGAAAT TCACCAGAGT  [ 6800]
Pcrbsap     ..G....AGA GAGAGGGG.. G.C.AGCGTG GTG.GC..A. TT.G.AAG.. C...TACGT. ..GA....TT T.A.AA..A. ..CG..T.TC .TGTA.ATA.  [ 6800]

Pcrbsa      ATGTTCAGTT TTCGCTTTCC TCCTTGCTGT TAAGTTGTTA GCCCCAGAAT GCCACTGTGA TCATATAA-A TAACAAGCCC CACGTACACA ACAATCACAG  [ 6900]
Pcrbsap     CCT.CTGAAA AC.AGAACTG .GG.A.T.CC .T.A..--.. .TTT..ACC. CTTGT...TT .TT..C..C. .C....TATT GGA..GTGAT .TTT.T.ATA  [ 6900]

Pcrbsa      CCATGGTAGT GGCAATTGCG GAGGCCACAG TGGCAACTCA TCTGCTAATT CAGGAAATTC AGGTTGCT-C CAATGCGGTA TCAGGAAACT CTTCAAATTC  [ 7000]
Pcrbsap     TG..CAA..A .CATG..TT. CGT.TTT.-- --.T..A.T. .TA...TG.. .--.TC..CG GAT..ATCA. ..TCAT.... .A.TTG..AA T..GG.T.AA  [ 7000]

Pcrbsa      TGTAAACACC CCGGGCTCTG GCGCTTCTGC ATCATCAGCT AAATTATCAG GCCCAGGAAC CCCCCCACCA GCTCCAACCC CTTCTCCAGC AACACCCTCA  [ 7100]
Pcrbsap     ..G.GG.--- ----.T.A.A TTTTAA.... G..G.A.C.C ..T..T.TGT CTT.TTA..G .T..AG.A.T TG.TAT.TTT A.G..TT--- -----TT.TT  [ 7100]

Pcrbsa      GCTAAAGATG ACGCGGGAAA AGCAATTGAC CAAATTGACG ACCAAATTGA GAAGAAAAAG AAAAATAAAA AATTATGCAT AATATCATCA GCAGCCACAG  [ 7200]
Pcrbsap     C.AC...T.T TTT.TAT.T. .TTGT.CATT A...A..T.A .TAT..C.TT .CT.TGTGTA T..GTC..C. --.C.GAAC. GGA.....TC .A.TTTT.--  [ 7200]

Pcrbsa      CTTTGGCTCT CTTACTAGGA GGAGCACTAG GATTCGGAAT TTACAAAAAC AGAAAGGCAC CAAAGGTAAA CGGAGATAAT ACAAATGGAA CCCCCGCCAC  [ 7300]
Pcrbsap     T.G.....T. TC.T...AA. A.GTT..AT. CT..T----. ..GA.GTTCT .T.GGA...A TGTTT..TTT TATTT-.GT. C.C....CC. T.TTGATA..  [ 7300]

Pcrbsa      TGAAGGTTCT GGCCCTGTCG TAGAAGGAAA TGCCGAAACC CCTGCTCCAG CCGATACTCC CGCCGTCCCA GAGACCCCAC AAGAATCATA ATTAAACAAT  [ 7400]
Pcrbsap     CC.---..T. --..T.A--- -------... .TTTA..--- ----.C.T.A T.C...T--- ----A.TT.. A.A.A.TTTT TGTCC.TT.C T..G.G---.  [ 7400]

Pcrbsa      AACGGCACCT TGTACATATA AGTGCAACTG CGTATTATGC ACATGTGTAC ATGTTTATTC ATCCGTGTGT GAATAAATAA ATACGCATTT ATGCATTATG  [ 7500]
Pcrbsap     TG.TT..GG. .A...T.T.- --.TA..T.A .T.T....AA .-..-----. ..T..C..C. T..TT.T... C..A.TTG.. ..---..AAG G..A.--...  [ 7500]

Pcrbsa      TGTGCCGACA CCGTGCGTAT GCTCCGCACG TACATGTGCG CAACTTGAA- CATGTATGAA CCACTTCATA TACTGTTGTT GCGCTGAGGT ATTTATTTTA  [ 7600]
Pcrbsap     .T.AAA...G T...A.T.G. TGAAGT..TC .GA..CAT.. TC...CA.GG TGGAGT.C.. TTTA..TGCG .C.......G CT.A..GTAC ..CCT.....  [ 7600]

Pcrbsa      GAAGCACAGA CAACCATGAG AAGNNNAAAG AACTTTTTAT TGATTTCTTA GTAAAGGCGT GCACAATTTT ---ACGTCCT TTCCGCAAAT CATAAAGACC  [ 7700]
Pcrbsap     TT.TTG-... A....TCC.C T.ACGA.TT. C...GG..T. AA..A.TC.T .C.G----.. CT...T.... GAA.TAGG.. C.T.TTCC.. ...GTTT...  [ 7700]

Pcrbsa      TGTACTGTTT TTTCATTCAA CTACACGGAT TAAAAGTATT CTTCTGTGCA CCTTGTGCGG AGCAAAGATC GTTGTGTTAT TCGCGGTGGG GACGCTTTTC  [ 7800]
Pcrbsap     .T.GGA.C.. ...-G.A.TT T.TTTTTTT. .----...C. T.AT.T.TT. ....T.T--. .ATTG..... C..----... .G.AAAA.TT C.T.G...GG  [ 7800]

Pcrbsa      GCTTTGTGTA AAAATGTCCA AGTTTTAACT GCTCTGATTA GAAGATGTAG GTATCTATGT TTATACCCTT TTGCTTCATT TCTGTTCGTT AGAAAGTCTA  [ 7900]
Pcrbsap     ..C..T.C.T TG.G.T...T .C..A.G-.C ..G-.AT.C. TCCCT.T.G. .GG.G.G.-- ---C.GTGG. .C..GGG... GA.A.-...A G.GG.A.AA.  [ 7900]
```

```
Pcrbsa      CGCCGATGTT AACTGTGTGC GGCGAAGGAA AATGGCTGTA AAAGTAAACC TGTCAATTGG AAATTACCTG TGACGCAGGG TTCTCCCTTT ATGCCTACCA  [ 8000]
Pcrbsap     ..G..TGCGC .CA.A.A... A-.AT..T.T T...TAG... T--.C.T.TG ..GTT...TA C..C..T.-. .A.T.TCC-- ..GCAAA... ..A.T..AA.  [ 8000]

Pcrbsa      TTTGGGAAAA TAGAGTGTGT TGTTGGCGAA AAACGCAGTA GGTGGAGAGT TCTCCAGGGG TCATTTCTTT CATACAACAC GTCGTAACTA CTGCTCTGTT  [ 8100]
Pcrbsap     ...T.CGCTT ACA....ATG .T.AC.T.GT G..TAATTCG A..TCTTGCG C..ATCAAAA .TGCGAACG. G..G..GTTT TCAT.TG... TCCGAT.T..  [ 8100]

Pcrbsa      ACACGGAGCA ACAACAGAAA TATTTTCATG TCCAAATGCG AAATGCATTT TTGAGAAGGG GAAAAATCAT AATGTCTTCT CTATTTCTTT TTTTTTAAAA  [ 8200]
Pcrbsap     G.G.A-..TG TTC.TTT..G ...CA.T.A- ---.....G. GG.AC...AA A..T...C-- -...T..TT. C.A...GCA. G.GGA.T... ......----  [ 8200]

Pcrbsa      ACTCCACAAG TACCTTTCTG TTTTTCACGT TCCCCTGTTT GGTATAAATA TTTGCCCCAA AAAGGACGCA CAATTTGTGC TTGCAAAATA TGGATCCTTA  [ 8300]
Pcrbsap     -.GT...T.T ..TT..A.G- ----.TG.A. ..ATT.T..C AT..C...CG ...------. ...AATT... G---.C.C.A .GAT.....T .CTG.TTGCG  [ 8300]

Pcrbsa      TTTTTTTAAA TGTATTTTGT CACACCATAA GGGGGCGAAG AATTGTCTGC TGAGCAGAAT GTGTTTTTTT CTGATTTGCA CATGTGTGTA TATACACTTT  [ 8400]
Pcrbsap     CA..---..C .CCC...AA. T.TTTTT..T AC.TTTA..A .T..TCA.A- ---------- A.T....CA. ACAT...T.C .....T.TG. A..GG.TAA.  [ 8400]

Pcrbsa      TGGTGTAGTG AAATTATTGT AATTTTTTTT TTTTTCCGC- ---TTTGCGT ATTGCATTAT CT-CAAAAGG GTGTTCATGA GAAAAGTGTG TACAACAAAT  [ 8500]
Pcrbsap     .T.A.G.A-. G...CCG.A. GGGCA.A... .GACG..A.A AAA....AAC GC.AT...T. T.A..C..TT ......CC-- ------.C.T GGA.GA....  [ 8500]

Pcrbsa      TCGCATACGA GCAGGGGGCA AACAAACTAT TCTGCGTGAA AAAAAGTAAA AAAGTAAATT AACTGAGCTG TTTTTTATTT GCGAAATATT TTTTTACGCC  [ 8600]
Pcrbsap     .T.TTAGGA. TT.AAAT... ..G.G----- -----..... .....AA... .C.A.TT-.. G..AT.TAA. ....GCCACG .AA...C.AG CGAAA.A..A  [ 8600]

Pcrbsa      GCGTTCTTTT TGGCAATCAT TTAGGTTACC CTGTGACAAA GTTTGTGTGT GTGCTGGCTG ATTCATTTTT CTGCTAATAT TTATAATTGT TCGAGCTTCA  [ 8700]
Pcrbsap     AA...GC... .TCAT..... .A.AA.G--- TGA.A.A... ...AA..AA- A..T..-.A. G.--G..ACA TAAG...A.. AA.AC..C.G G.AG--.AT.  [ 8700]

Pcrbsa      TCATTTGGCA AATCAAAAAG GAGATTTTTG AGGGGGGATC AAAAATTGTA GTAGAATGAG GAGGTATTTT TCTTCGTTTC ATTTTTGTTA ATGTTTTAAA  [ 8800]
Pcrbsap     .TTGCA..TG ..G.CCCC.A A...GC..-- .T..A.T..A .G.TG...GT .C...T.TTA T.AAA.A... C...TAAA.T ..GCA.---- -------...  [ 8800]

Pcrbsa      AAAGTGTGCG TAAAAATGTG AACAGAATTG GAGGAGTTGC AGATAATGCG TCCTTACTTG CGGTTGGAAA TATCAACTGT TCGGTGGACG CTTTGATTTA  [ 8900]
Pcrbsap     .TG.CAAA.T CGT..C.T.T TGG.AC.C.. CCTATA.... GT.GG..A.A ..AAGG.A.. .AT---A... .T........ GTA..T.--- ----------  [ 8900]

Pcrbsa      GCACTCTCCA TTTTGTGCAC TTTGCCATCC ACCGCGCTTT GGAGGTAGTA CTCGGCTTCC ATCTGCTATG TGTTACTTTA ACTGGGTGAA TGAATTCCTC  [ 9000]
Pcrbsap     -----.A... .A..A.A--- -...GA.AGG .TA..CT.A. .A.AC.TC.T T.TT..A.G. G..CCAA..A ..C.G...GG CAC.AA.A.G AA.TA.GTAT  [ 9000]

Pcrbsa      CCAGCGTTAT GTTGTATCAA ATTGGACTAC TAAAGTTGCG CCTAAAAGTG TCCTCCTCTT TTCGCGCTTC TGCATACAGC AACATGTTTT AAACGAAAAA  [ 9100]
Pcrbsap     TGC.AA..T. T.C.GTATG. GCA.A.T..G CG..A..T.C ------G..T ...C.TAAA. ..T..A.--. AAAT..TG.. .C...T---- ---..C....  [ 9100]

Pcrbsa      AGGACAAATG GTTTTCCAGG TTCATTAGAA AGTCATCGAT A-ACTTTACA GTCATGTAAA GTGCACGATG GAACGCAAAA ATTTCAGGAG TGTATGAATG  [ 9200]
Pcrbsap     ---------- .....G..T. CG.T...AG. G.A-...TT. .C.T...GA. AGA..A.... ...TG.---- ---------- -C..TGT..A .AA.A....T  [ 9200]

Pcrbsa      CTCTATTTCC GAGTGAAAAA AAGGACAAAC CATTCAAATG CGCTTCGCGC GGAATGCGCA CGAGAGTTAA ATGTTAATTG GGCGAATATG GGGGATGTGC  [ 9300]
Pcrbsap     T..CTC..-- ---------. ..AC.TC.C. ..C------- --...T.GTT .A...TGAT- ---------- ---.C.T... ....TG..AC ..---....G  [ 9300]

Pcrbsa      TATCACGCAG GAAACGGAAA ATGATCCA-A AATGAGCAGA AAGGCAGACA AATAAGTAAA AAGGCGGACA AATGAGAAAA AAGGCAGACA AATGAGCAAA  [ 9400]
Pcrbsap     -.GG.AAA.A ....A..GGC ..AT.A..T. ....CA..AC .CATT.TTTG CCAT.AC.TT GTA...--T. .T..TTGC.T TTTTTCC... TTC.T.TTTT  [ 9400]
```

```
Pcrbsa     AAACGATGCG GTATAAAAAT CACAAACGAA GTCACTTCTG GACAGCAGAC GAACTAGGAG TGAGCGCCGC GTCACAGCGG GGAGGCACCC TTAAGGGCCA  [ 9500]
Pcrbsap    .TTTT.C..A .......... TG....G.TG .A-------. T.T.AA.T.G .CT...AT.T A.GA.T.TTA CG.....T.. AA...TT.A. GCCTACTT.T  [ 9500]

Pcrbsa     CCGAGTTTTG ATAAAATTTG AATTATCCAG AAGAGAGCGA TTTCTCACAA AAGTGAATTT CGAATTAGGA AG-ATGGCAA ATATAGTACA GCGGTAGAAA  [ 9600]
Pcrbsap    A.CC..G.AA GCGC.G..GC ..A.GA...T .T.C-.TTT. ..ATGT.... .TAC..TAGC TA.T.A.TC. C.T..CT.C. CAT.TA..T. ..A.G.-.TG  [ 9600]

Pcrbsa     TGAATTGCGC CACCA-GGGG GCTGCTCATT TGGTAAGGAA GGAGCTAACC ATGTTTGGAT AGCGTGGTTT TTTTCTCACC ATTGGGTTTC ATTTAATTAG  [ 9700]
Pcrbsap    ..C.CCTA.G A.TG.T.CA. ATG.T...C. .T..GG.A.T AT.--...GA ..A.CC.T.A TT..AAA..A .------.T. .AC.C..A.G ..G..T..G.  [ 9700]

Pcrbsa     ACCGTTTTGT GGGGGGTTCT GCTTTTTGCA GAGCTATTAT GCACATTCCT G--GCGGTTA GTACTTTTTC AACTGATTGC GCAAAATAGT CATCCACATT  [ 9800]
Pcrbsap    ----...CA. TAT.--..T. ....A..TAT .G.TCCA... .A...CAT.. .CT..A.C.. T.G...A..T T-T..G.CCT TTG...G... A---------  [ 9800]

Pcrbsa     TTTTTCTTTT CCGATGTGAA GTTCTGCCAC CTATTTATTT TATCCGCCAT ATATTTCTTA AATTCATCAT TAGAATTAGA TTCCTTCTTT AGAAAGT-TG  [ 9900]
Pcrbsap    -----TGG.. GAA.G.A.GG .G.TCAT.GA A..ACG--.. .G....ATTA .CTA.G.... ..AAA.G-.. ...TTAC.TT ..A..GAAGC .....A.A..  [ 9900]

Pcrbsa     GCAAAGTCGT CTAAATCGTT TGCATGCATT TTCATCAAAT T-GCTCGTCA GGATGTCACT ACACTGCCAC CAGTATTTGG ATCTATATTC CTCTCTTAAA  [10000]
Pcrbsap    AG..TAC.TC T.T..AAAAG .T...A..A. .AAG.G.C.. .T..GAAG.. ..GAAA-.TA .A..CTAT.A A..A.AA.AA .AG....CAT TCG.AAA...  [10000]

Pcrbsa     TTTTTTTCTT TTGCAAATGA CAGAGTAAAA ACCCACAGCT CATCAACCAT AATTTGAAAT TTTCTTCTTA GATAATCGTG TATATCGAAG TACAATTGGA  [10100]
Pcrbsap    ---------- --AA.....G TTTCACTGT. .GTTG.GAA. TGG....--- -...G....C A..T.ATA.. TTC.--.... .GC.CT.G.A ..GG...A..  [10100]

Pcrbsa     -TCATCTCTT CTTGGGTCAT ACATGGTCCT AATGCATCAA TGGTGTCCCA TATTTCTGTT TTCAGATCTT TCTTCAACGG GGTAGAATTT GCTGGAATGT  [10200]
Pcrbsap    A.T..TGAC. A..AAA.A.. .AT.--.T.. .---...A.. ....AATG-. .....A...A A--....T.. ..C.TT-T.T AA.GC.G.A. --...C.CA.  [10200]

Pcrbsa     TTTTTGGTTC CTTATCTTTT GTTTTTTGTT TGACTTTTGT CGGGGTCTCT TCTTCTGCGC -TGCTATCCT TTGTGAAGCA TCTTT----T TAAGTTTACG  [10300]
Pcrbsap    .A..C.T..A T.A..AGGGG .CAA---..A AA..ACG... ATACT.TG.A CAAAA..GAA A.ATG.G.A. .C..T...GG .T...ATTA. ...TA..TTC  [10300]

Pcrbsa     ATGAACGCGC TCAAATTTCG TCTACTTGCG TGGCTTGGTT GTGATTTTTC GGTTTGGTTG CTTCTGTTAG TTTGGGCGTT CTATTGGGGG AGGGGTATGC  [10400]
Pcrbsap    TAA.GA..AT AT...C.GTA CACCA.CT.A ..TA..AT.. A.--.....A C.AA.TA.CT T.GT...A.. C.GCACAAAA ...CCATTT. T.T..G..AA  [10400]

Pcrbsa     GATTTGTAAA GTGGCGGTAA AGTTGAACAG AGTGGTGAAA AAGGAGAAAA TGTTGTTGTA AAGATGGT-T AGTGCTATCG CACGGAGTGA CAAGCGTAGA  [10500]
Pcrbsap    A..CCT.... A...A.T.TC CCC.A..A.A .A.AAA.TCC ..AT.T.TT. .-..A..T.. T.T..TT.A. .AC.AA.A.. ...TA.T.-. T.G.TA..T.  [10500]

Pcrbsa     TAGAATGAAC AGCCAGTGTG CACTAATTGG CAGGAACAAA AGTACATATA CGCATCTACG AGTGCGTACT TACCAGAAGG AGAAGAAAAA GAAGGACTGA  [10600]
Pcrbsap    ACCCC.AGGT GA.A.C.T.- T..GG...TC T.TTT.A... ..A..-C... A.T.AT.CTC ....T.A--. .G.TT...AT G.T.C.GGC. TTTACCT.TC  [10600]

Pcrbsa     AAGAAAGATC TTCAAACGGT TGATACAGGC AAAATGTTGG AATTCTGTTG CCCCTTTTTT TTCCCTATAT TGATGTGAAC ATGTGCATAA CCTCCTGGTG  [10700]
Pcrbsap    .T.T.T.C.G A.A...---- --...G.AAT ...-.A..A. ....T.---- ---..G.CA. ..TA.A..-. .AG.T.TC.T G.A.AG.... AGGAA.AAAT  [10700]

Pcrbsa     GCATTCATCT TACTGCACTT TCTTCTAACG AGTTAGCCGC GCTGTAGCAA TTTGGTGTGC AGTGAGCAGC ATATATATAG GGACCACTGC TATTTCTTTT  [10800]
Pcrbsap    AA......AA ATTAAT.T.. .GGATA.TGC G.A.GAT.AA ATAT.TT... AG....AAAT .A-A..A.A. T..A..GA.. ATGTA.AAAT A.A..A....  [10800]

Pcrbsa     -TAAGCGTCT CAGAGACGTT TAACTGAGTC AGTGGACATG CGTGCAGATT ATCACTTTAA CATTAGATCT GTGTAGA-AC ACGTGGTGTG GCCACCTGGG  [10900]
Pcrbsap    A..TATA.A. TGT..-TA.A ...T.T---- -..TAC.CCT .AC.G...C- .C..T...T. T..GTCC.T. T.T..C.TGT GA.CAA...A AA...AAT..  [10900]

Pcrbsa     CATAAATCAA TACTTTTGCC TTTACTAAAG CATAAAAACG GGGTGTACCG CATGAAGAAA ATTAAAAACA GAGGTGACTG GTGGCAAAAA GAAAAAAAAA  [11000]
Pcrbsap    TT.GGT...- ..AG.A..AA A.A.A..T.T T.ATG...T. CCTCTACT.. T...T.-.T. ....T.TTTG T.T.------ --......T. -----...T.  [11000]

Pcrbsa     ATAGATTCAC TTCTCATCAT AGAGAAGCAC CATGTAACGT TAAAAAGATT CAAATTA--C GGTCGCTTTG GTGTAACGAA AAAACGATAA AAAGAAAAAT  [11100]
Pcrbsap    T..TG.GTT. ...CG.GA.. -.T.C.CTG. ..-A.GGTA. ...TC.TT.A T.GCA..TTT A....GACCA T.T.TT.T.T GGTG.TT..C C.TCCTT.CG  [11100]
```

```
Pcrbsa     TTAAATACAA ATTTGCAAAA GGAGAATGAT GAAATTATGC ACACTTTTTT TTTTTTTATT ATATATAGTA TGACGAATGA GTTAATTTTT TCAGGCAGGT  [11200]
Pcrbsap    ...TGC.GTG .AA.ATT... ----...A.. .C...A.AAT G..A.G...- ---------- --.A...A.. .A.AA..A.G A..C...GC- -..A.G..-.  [11200]

Pcrbsa     GTAGCCAGAA GGGGTAATGC AAAATGGAGA TGGTTTTACA CCTAAATTGG GGGATCTCCA AAAACTAGGT ATACCCCTAT ATTTAGGGTA TTATTTAAGT  [11300]
Pcrbsap    .GG.GGG.GG .....G.C.G GGC.C----. ....C.A.A. .G.T..--AA ATAGC.AT.. G..GA..AAG .A.A.T..TC T..GT..T.T .C..C...T.  [11300]

Pcrbsa     GAAAAATAAA AATGGATCTC CCCCCTGACC CAGTTTTTTC ACTTAAAAAT GGGGAATTCA AATTTATGAT ACACACGTGA GTCAGATCAC TCTAAAGGGC  [11400]
Pcrbsap    TT.G..A.TG .T..A..TA- ---------- ----.A.A.A .TC.G....A AAAA..---. ...C.G.A.. .A.----... CCAG..ATGG G.C.T.T.CA  [11400]

Pcrbsa     TTACATCCTA CAAAT-GTGT GAAGTATAAA AAATTAAGAA AAAAAAATTA CTTTTCCTTG ATAATTCGCT TTATAATTCT AGCTTTTAAA ATTTATTCTC  [11500]
Pcrbsap    A.G.GG.GA. A....T.GAG A..AA..T.. ..GGC..A.. .G..G..A.. GGG.C----A .A..AGG.-- ----..G..A .TT.GAA... ..G.T.AAGT  [11500]

Pcrbsa     TTTATAAAGA AAATTGCGAG TTCGCTTTTG CGCGACTCAA CTGCGCGCTG TGCAATTGGT GAGTTGTAAT TATATATAAA TCCCGTTCTA TAAAAATTGC  [11600]
Pcrbsap    ...TC...TT ......-A.T ...TT..... ----.T.TTT T.TTTTTT.A AAA....A.A AGT..T.TT. .TA....--- .GTAN..T.T ..TGCG.ATA  [11600]

Pcrbsa     GGCAAAGGGG CAGTTATTTT TTTCTCAACC TTGCCACCTC TTACTATCAG AAAAAGTGAT GGAGCAACGT GAAGAATAAA TCTGCGCTTA GGTTAGGTGC  [11700]
Pcrbsap    A......ATT A..AAT.... .G.AG..GTT AA..A.---- --.T..-..A .TG....T.. AA.ATTT.T. A.TTTT.G-- .T..TT.G.. TT......A.  [11700]

Pcrbsa     CGACTGGGGA GGCGGCTACG TTTGCGCCTA TGACTTTGAG TGTTTGTAGT GATCTCGGTA GTGCGTCACC AGCGAAAACA ACTTTCTCTT ACTTCCTTCT  [11800]
Pcrbsap    ATTT.AAATG CAAAAT.GTT ...AGATAGT .TTGC..TC. CC...T.GT. TT.T.TATA. T.ATA.TTTT .-T...GTNN NG...A.TA. T...A.AAA.  [11800]

Pcrbsa     CGTTCTTATG CGTCCAAAAC GTTTTTAAAA TGAAACAATA ATGCTAGT-- TTTTTTGGGG GGGGCGAATT ATAACACATC TGGTGAAATG TCACCAATTT  [11900]
Pcrbsap    TAG.AG-G.. .AATA.TTC. TA.CA....G .T.T.T..A. .CATA.C.AA A.GA.CA..A AAATAAC..A G...A..... A.C..TT.AA .TGTT...A.  [11900]

Pcrbsa     GGGTGATATT TGATCCAATC AAGGTAAGCT TCATTTTTTC AGATTTTCTC ACTTTC-TCC TTTTACTAAT ACTGCTAATT TGTGGGAAGC AGCCCCCCGC  [12000]
Pcrbsap    AT-.AT.... C.TAATG.AT ..--...TTA AT...AA.GA .AGAA..TGA ..AA..A.TT A.A..GCG.A .GGAT..GAA GAC.AA...A .TTTG..AAG  [12000]

Pcrbsa     CGTGATTTCT TTGTGAACAC ACATGTGTAC ACCCCTGAGC GACAAAAAAA AAAAATACCC ATCAGATCAG ATTTGT-TTC GCCTGAGCGA ATAAGCGAAA  [12100]
Pcrbsap    T.CA.A.... G.T.---... ..T.TCC.TT .TTG.CATAT A.AT.GT..C CTGGT.GAT. T.GCC.GT.. TCA.A.GC.T .T..C.AA.. T.....C.TG  [12100]

Pcrbsa     TGGAAAGGAT TATG-AAATG ATTTCTCAGC AAATACGGCA TAAAAACCCG AGTGGGAAAT AAAATAAATT CAGCTGTAAG AAAAGTTAAC CAAAAGTATT  [12200]
Pcrbsap    CAAGTGAA.G ...ATGC..A TA..A.AT.T .G.A..T..G A.CGGCT.AT TAAAAC.GT. .C...CT.C. .GA..-.G.C .TTTT..CTA T..GGA..AC  [12200]

Pcrbsa     CACGAAACAG TGAAAAAAAA AAAAAAAAAA AAAGGGCCAA AATGTGCATA TAAATACACC TCAATTTTCA GTACACTTTT GCGAATAAAG CT-TTGTGTA  [12300]
Pcrbsap    T...G..A.. CTGT.GCT.. T.CTTGCTTT .GCACT.TTG .T.AATTTCT .G.G.GTGTA CT--.G..A. .C---..... AA...A.... T.A..AAC.T  [12300]

Pcrbsa     ACGGTTGGGG ATTGCCCAGT CGTTACACTT TTAGCAGATC AGTGTGGCCA TAATCTATAA TCACATTTCA GGTTTCGTAA GAAAAGGGGG ACAAAATGAA  [12400]
Pcrbsap    .A..--AATT ..AA.AA..A A..A....G. --.ATG.... C..CCATTTT ..G.G.G..T CA.----..G A.....TG.C CT.TCA.CTT TTG.TG.T.G  [12400]

Pcrbsa     GAGAAAGGTT TAACAAATGA GGGGATCCCA CATGTAAATG AAAATATTTT CCTTTGACCA ACATACTGGA AGGGGTCTGT TAAAATGTGC AACGATTTTT  [12500]
Pcrbsap    .GT.TT..CC .....T---- --..C.ATG. .GG....CG. GG...----- ---.A..GTT CG..T.C... GA...AGCC. G.G..ATA.. T..C.CA.C.  [12500]

Pcrbsa     AACTCTGCAT TGGTTCACTC ATCCGTACGT GTTATCCTTT TAGATGTCCA GTTTGTTTCG TCCTTATTTT TTTGCGGAAC TCTGAGAGGA GCGCATGTTT  [12600]
Pcrbsap    ..----.G.A G.---..G-. .GG..CGTAA A...C..AA. .CT.AAGAAG AGAG..AGT. A.-------- ---AA.A..T AACA.T.CA. .GC..A--.C  [12600]
```

```
Pcrbsa     TCCCCGCGTG GTAAAAACAG ATGGGATTTG CTAACCCGCT GAGAAAATTT GCGAATTTGT TTTTGTTTTA GTGCCTTTAT GTTCCCATGC CTTTCTTCAC  [12700]
Pcrbsap    .GG.TTT..A A.TGG..T-. ......A..T AA....TT.C C.A..----- ---------- C.CAA..GG. .G..AAG.C. .G.G...GCA GCCG.GGT.A  [12700]

Pcrbsa     GTTTCGCCTC ATCATCGTCA TCCCCCTGTG GGGAGGGAGT GAAAGGTTTC CAAATTGGTA TCTCCTCCAA GGGATACACG AATAAAAGAA AAAAAACGTA  [12800]
Pcrbsap    T.CCA..TC. .AT.G...-. .ATTAAAA.T .TT--.C... T...ACGC.. GT.G...AA- ----T.T... A.A..CG.TA TT.T..GT.. ------..CT  [12800]

Pcrbsa     TACATATATA TATGCATAAT AACTGCACAG AAGAGTTAAT ACATAATGAG TGGGCGTAGC GTTAATTGGG TGTGAATACC GTGTCCCTTC CACAGGGA-G  [12900]
Pcrbsap    .TT.------ ---..T.... C----....T ..CT.A..C. ..G..TC..C .TT.T.CG-- --C.T..T.C .A.T.----T ....T.T..T A.TTAAA.T.  [12900]

Pcrbsa     ACTCTTTAAA TTGAGTCACC ATTTGGTTGG CTGCCACAGT TGGTACATTT TAGTCACATC CGATTA--TG ATGTATACAA GTAAAAATAA TAAAACCTCA  [13000]
Pcrbsap    .T.....TT. --AG...--- -...CT..T. ..T.GG..T. ..A-.G..C. .-..T..T.T GAG.A.AT.A GA..G.T... AGC...CAG. ..T.G.A.TG  [13000]

Pcrbsa     T-CTTTTTTT TTTTTTTGCA TT--ATGATG GTGCTATTCG AGTGAGCATT TTTGCTACTT CGATTTAATC ACATGTGTGT GACGTCCGTG GCAAATAAAA  [13100]
Pcrbsap    CG.G...GAA .AC.ACA... .GGA..A.CA AAAT.GAA.A ...C..A... ..-.T.CT.. T--.C.T..T TTGGC.TA.. T...ATTAAT AGG.G..GCT  [13100]

Pcrbsa     ATGTGTGCGC TAAACATGCG AAGGGAAAAG GGGCATCCCT ACAACACGTG CCAGCGATGT AGCTGCGGTG CAATCGTAAT GGTGTCCCTC GCGTAATGCC  [13200]
Pcrbsap    -..G.G..AT .TGTAT.CA. .T.TC.G.G. T.AA..T.T. .G.TTTTC.. GAGA.A.-AC .A.....AAA GC..TTGCC. AAAA.A.T.. CAT....CAA  [13200]

Pcrbsa     AAAGAAATGA TTCCCAATAT AACTGACGTC TACGTCTATA CAGGTGTAAC CATAAAGATA ACTCGCATCA CATTTTGCGA ACAAACACAG ATGGCCCCTT  [13300]
Pcrbsap    G..CG..A.T .-----.AGG G.G...A.A. G.T-CAG... .C.TC....T .T...CC... .ACTATGC.G -.C.AG..TT TGG.TG.A.. ..-----T..  [13300]

Pcrbsa     CACAGAAAAC AAGCCAATTT TTATGTTACA CTTATTCATA TAAAATGCAT TTGCAATTAA AGAAGTTCTA AAAAATAGTG ATGCATGTCC GCTTTGTGGA  [13400]
Pcrbsap    -----....T ...AG.T... C.C.-.CGG. G...A..TCT ..G.T...T. CC----..C. GTGCC..A.G .G....--CA .A.TC.T.GG .T.C..G..C  [13400]

Pcrbsa     GACGCTCTTA CGTGATATCT TCGATAACAA CCTACCGTTT AGCAAATAGA TAC--ACTTG CGTAACTCGT GTGTCTTTTT TTTTTTTATA TTGCCATGGG  [13500]
Pcrbsap    ..-.TAT.CG ..CA.G---- -...G..AGT TAA.AGAA.. GA.GG.AG.G C..CAC.AG. ...GGAG.T. .C.G...AA. ..GAC.C.A- ----..C...  [13500]

Pcrbsa     TTAGTTAACA CAGCTTAAGT GTCAATAAAA ATGGCATGCA TACAAACATT GTGATGAATT TACTGAAATG CCGATTAGCA AAAAAAAATT -TTTTAAATG  [13600]
Pcrbsap    AA.AC.C..- T..T.....- ----.C..G. G.A.G..TG. C.G.TTA..A .CTC.----. .CT...TT.C TT.GA.G.TG .TGC.TGGCC G....T.G.T  [13600]

Pcrbsa     AAAAAAAATG ATTTTCCCTT TTGAGGTCCT TTTTGTGAGA GGATTAATAT ATCCTGCGTT CCTAATTGGT TTATCCATTG CTGTTTTGTT TTTATTTACT  [13700]
Pcrbsap    CGTG..T... ....GT.TGG ..A.TTC.GA .AAC.AAC.. .ATC.----. .A.....--. AA.T.GC..C AA..A.GA.A TAT.C..A.G .GGGA..GAA  [13700]

Pcrbsa     TGCTATGTGT GAATAAATGC CGCACGGGGT TTACCCTCCA GTAGAGGATC CATTTC--AT ATTTAGTTGT TCCGCTTACT TTTTTTTTTG CATTCCACGT  [13800]
Pcrbsap    .A.GG.TGA. TTGCTT..TT T.A.GAAAA. A.TGGGATGC ...A..TG.. .C....CCT. T.C..C..AA .T......TC A.AC.G...C TT..T.G...  [13800]

Pcrbsa     TCTCAGGATC TACAAATGCG TGTACTTACC ATTTTTACGT CCCTAAATGT TTCTCATTTT GAAATTTTGT TTAAGGGGAT GCTACTCATA AATGGTCCTC  [13900]
Pcrbsap    A---..A..G ..TTTGCTT. AT.GTAA.G. T.C..AGA.G AA.G.TG... G...A.CACA AGG.A---.. ......CA.C AAC.GGTC.G TGAT.....T  [13900]

Pcrbsa     ---TGCATTT TCCTGTGTGC ATGATTCTAG TGATGCAGAG CAAAAAGCAA TGCTCACTCT ACTCATGCCC TCAAGAACAG CATTTTCTTC ATTATTTCTT  [14000]
Pcrbsap    AGA..A.C.A GG...CAC.. G..C.A.-.C ....ATGT.- T..CG..TT. CTAAA.T.AC GA.TC...TT G..GATT.GT AC.....C.. -------.AC  [14000]

Pcrbsa     CCCCTAGTAC GAGTC-CCTT TATTGCCATT GTCATTGTCA TCATCCTTTT CCCCTTCAAC TTCCGTAGTC ATTTTAAAAT AATTCCCATA AAGTGAGGAA  [14100]
Pcrbsap    TGAAA...GT AG..AAT... ...CAAT.CA --T..C..G. .GGGGA.AGA TTAT.G...T .AT--..A.. T.GAACG.GG ...G..T.GT ...C-.T..T  [14100]

Pcrbsa     TTATACGA-- GATATCTCCA GATCCATTAC CACAATAAGT AAAGTCAAGC TCTCCTACAT CCGCTCCGGT AAAATTAGGA TTTTGAAAAC TATATGAATT  [14200]
Pcrbsap    .C..CA..TT .TGC.GA.T. CG...C.GC. .TTTG..CAC .CC.C.CGT. G.......CG ATTGAAA.A. .TG..G.ATT G....G.C.A G.AGAA.GGG  [14200]

Pcrbsa     TTTCACTTTT CTAAGTTTTT TTTTCCTTTT CTTATTTTTT TTTAAATTAC AGCTATGGGA ATTTTTCCTT CGGGCGCCTC CCTCGGTGGC TCCTTTCTCA  [14300]
Pcrbsap    GA.T.TA.C. TCTTT...C. GGAAAAACCG TAA..CC.A. C..---...A ..GA.G.A.. .G.CG.AACA A..---TT.. .G.A....AA -...GCGGA.  [14300]
```

```
Pcrbsa     AGGTCATTA- TCTATCTCAT TTACATCTTC GTTAAAGTGT TCATCCTCTT CG-GGGGTAC CGCACAAAAA GTCACACATT TTATCGTACC ATGGT-----  [14400]
Pcrbsap    G.A......A .GG.A.GA.. AG.GGCGAAT .GC.TGT.T. ..G.GGGGG. T.C.ACAA.T A.TGTG...G T.TGTTTTA. ..G.T.A.A. G.C.AAAGAC  [14400]

Pcrbsa     -CCTAGTGGG GTGCGCTATC ACAGAATAAA AAGTGAAATA TTTATGTAAT G--CTCTTTA CTTATTGGAA GTGCAATTCG GTTTTATATA CGATAAAAAA  [14500]
Pcrbsap    ATT.C.AT.A A..GAA...A CTTTTT..CC .TT.TGTTCG C..CAACT.. .AAT.A.G.G A.C...C.C. AAATG...TT C..G.G..GT T.G..GT..T  [14500]

Pcrbsa     AGAAAAAAAA TGTAACGATG TGTGAGTATA TCACCCAAGT GGC-ATTTAA CTTACGTCGT ATAAACACGC TGCTAGGGGT ACTACGATTA AGAGTGAAAG  [14600]
Pcrbsap    ..C..TG... ..A..T-... .-.A.T.T.T .TGGTT.TA. AT.T.A.... A..GACAT.. T.T.TTT.AT C....TTAC. ..C.ATTA.. CA..-A...T  [14600]

Pcrbsa     CAAGGGAAGA AAGAAAATAA AAGTGTTTAG GATTAAAAAT A-ATTCTTCT GAGCTTCC-T CCAGGGAGAA CATTTCCCGT AGTGATGCTA TTTGTTCCAT  [14700]
Pcrbsap    .CTCAAC.AT TTT...C..T C.A.T...T. C..GC..... .TG.AT..G. ..AT..G.A. GTTTTA..TG TG...TGT-. .TCAT..T.G C.A...--.A  [14700]

Pcrbsa     TATGAATAAT GGGAGCACTC TCCGTGT-TA ACAGCTGATA AAGGCTGCAG GGTTGAGGAT TCATTTCCGA GGAAGGAAGA ATTCACAGTG CCTACGGCAT  [14800]
Pcrbsap    ..GT..G... AAA....AAT GTGA.AAA.T ..GAGGA... T.TTT.CTTA TAG.A.TTTC ..TCCATT.. .T..TTCTTG ...A.TTAGC A.AGTCTT.A  [14800]

Pcrbsa     CGGTGATAGC CTCAGTAAC- GCCGCTAAGA ACACTCTGTA CTATTGTGCT GGTAACCGTA GTTGATATTT GGTTGGTGTT CCCTTCGAGT GAAAAGGAAT  [14900]
Pcrbsap    ..A..GAT.T ..TG..TC.T A.A..G.T.. .GG.CGCAGC GA.A..C.A. ACGC.AT.A. AA.TGC.G.G AC.GT.AA.C AT..GAAC.C TG..T.T..A  [14900]

Pcrbsa     CAATAGTCGT GGTTAAGGGT GTTGAAGGGG AAGTTAAAGT TGTTACATGT GTATCATTGT CATCGAACAA TTCACTTTGA AAGCTCGTTC CATTATTCAT  [15000]
Pcrbsap    .T.C.CCAA. TC..-----C T......AAT ..--.GGTAC .CC.T...AA AA...TAGAA .GCA...A.G ----.CA.A. ..ATA..A.. .T...--...  [15000]

Pcrbsa     TGTGCTGGGT GTGTTAATGT TTTTATAAAA ATGAATCGGA ATATCTGGCA GGTGCGACTT TCAGCCA-CC CCCCCCTCAA AAAAAAACTA TTTCGTGA-A  [15100]
Pcrbsap    A..AGA..AA .A.AAGG..A ...GGA.G.T ...CTGG.AT ..G.ACTTTT A....ATG.C .T..TA.ATT T..AAA.TC. C.GTGTGT.C .G.A...TT.  [15100]

Pcrbsa     ATGTAAAAAA GTGATGAAGT TATTGTATCG TGTTGTGTTC CAATTTTTTG GTACCATTTA AAGAAGAACG TTTAAAAAAA AAATGTGTAA ATTGTGTAAC  [15200]
Pcrbsap    CC........ ......C--. .GCG.A..TA CA.CNNNAAA G..C.....A T.G--....- ---------- C...GT...G GCG..CAC.. T..---..CG  [15200]

Pcrbsa     TCTGTTTAGT GAGATGTTGC TCATTTTGAA ACGTTTAGAA TACGTTGCAT TGTGGTGAAA AAAAGAATCA TGACAATGTA ACGTGCAGGT ATTTTTTCAA  [15300]
Pcrbsap    ..CT..CC.C A.ATCA.AAA --GACC..T. CT....T--- .T.A..CA.C .ACACG..TT .....T..-- ------.C.T CT.....C-- -C..G.G.GG  [15300]

Pcrbsa     AGTGGAATAT CTTCTTCTTC ATCTTTCTTT TTTTTTTTTT TTTTTTTTGC CACAGTTGTT CATTTAAAAA ATGTGTAAAG TGGTAGGTAG TCCACCTTTT  [15400]
Pcrbsap    ..CAA.G-.. .G.TG.G..A T..GCGG.GG GGACGC.... CGC...G..T A.A.A.-..C ..AG.TTT.. C..CTCTG.T .A.A..A.GT AGGTATC.A.  [15400]

Pcrbsa     GAAGAAAAAC AAATTAAGCA ACAATCAATG TGGTCTTAAA TTTAAAAAAT GGGAATATGT ACAATGTGAG CGGCGTGGGA CGGTCGGAAT TAAAAAAGTA  [15500]
Pcrbsap    .TTT.T.CC. ---..TT..T T..T.---.C ..T..G.T.G ---...GTC. AC.CCG.... TA.C....T. .....AA... AAA.G.---C .GT.......  [15500]

Pcrbsa     AGCTGCGGTT CTTTGGAAAG -GGGTGT-AC GAAATGGTCT CCTTCTTTGC A---CATAAA AAAAAAGGGG GTTTTCACTT TATTAATTTG TGCGTTACTG  [15600]
Pcrbsap    .A.--.T..C AA.......T TACC...G.. .C.GG.T... ..C.T.A... CTAC...TTG GG....TA.A ..G.GTTG.. GGCG..AAAC GCA..AGG..  [15600]

Pcrbsa     TTGTATTTCT GCTGGTTTGT TCCTTTTGGG CTTCTTACAT GTTCGTTTTG GCCGTATGTT TGCACGGCAT ACTTTTTTTT TTTTTTTCGA CCACATCTTC  [15700]
Pcrbsap    GA.AG..CTC CAG..G.CA. .T....CATA .A----...C ..-...AACT A.T.CTCTG. .A.....AGC .ACAACAGAA A.A.....AT GTC..AA.G.  [15700]

Pcrbsa     CAAATGTGCA AAGTATCTGT CTCTCATCTG GGACTTTTTC ACTGTTTGCC ATTTTAACAA GGAAACGTAA ATGCTGTATT TTTTAAATTA ACAGCACACA  [15800]
Pcrbsap    G.....CATT TTTG.GAA.G GGAAA.ATCA TA.TG.C... T..A...CTT T....TTA.. AACTC.AC.. G.A.CT.TC. G...TTCACG TTCC.CTGTT  [15800]
```

```
Pcrbsa     TATTGTACTT CTTTACTTTG AGCAATGTGC CCTTCTTTTT TTTAATTGCA ATCTTTTTGC CAACTGGCGT GTCGCAAGAA TAGATAAAAT CTACAGTTTC  [15900]
Pcrbsap    .GG.A..AA. A...G.CCCA .AA.GGAC.. A.AA----.. .G.GC..... .AA.A.GGAT .--..TATT. T.-------- ----.T.... G..---...T  [15900]

Pcrbsa     GATATGATCA ATATGGGGAG GCGAAATTGT GTGCATTGAA GTTGGCTATA AAATTTGCGT GCATTTAACG CGCAAGAAGA TCAAACAGAT AATCCATTTG  [16000]
Pcrbsap    .TC.----.. CC..AA..G. .....GAAT. ..CTGC...G --------C. G...G..TT. TTT.C.G.TT T...CATGTG .---GT.T.. .---..C..T  [16000]

Pcrbsa     TGCCAAAAAG AGGCGAAAGT GGAACCTCCT GCAAGCATGC GTGACGTGTC GTAATGGTTT TAGCGGGTGA CCCCCAATTC GAGGTAGCAT ACAAAATAGG  [16100]
Pcrbsap    ..GTGT.GT. .AATT.TT.. AATTTT.TT. TTTTT.-C.. T.TG...A.T .C.T.ATC.C A.AA.....T T.ATG.GAAA AGT..-.T.C .AC....TC.  [16100]

Pcrbsa     GAGTATGAA- AATGGGCACA TGCGGTTAGA TGCAT-ATGA TAATTATGTG TTTGAATTTG CCCTTCTTTT TTTTTTTTTT TTTTTTTCTT TCCTTTTTGC  [16200]
Pcrbsap    C.-..C..GC .GG.....A. CAAAC.ATTC ...G.G.AA. A..G..AAAA AG.A....AA .TGAG..G.. ....A...GC GAAA.A.T.. .TTACGCC..  [16200]

Pcrbsa     TTATATCTTA CTCGTTAAA- ATGTTACCAG GGGGGAGGGG GGCAAATTAA TCGTGCGTCC ATTTAACTCT CGTTGAACTT ATCCGCATTT TTAAGTGTCT  [16300]
Pcrbsap    G.TCT.T..G GCAA.C.TTT .G......CT .T.AC.AA.T --TTGTG.GT GT.CTG.CTG ...C.TT.T. .TGCT..--. ..TTAT.A.. G.TC.A.CT.  [16300]

Pcrbsa     CGGAAAATGG CACAATAGGC AAAAAGATCA TGTTAATTGA TATGGGCATT CAAATAACAC GAAAAAGCGG CACACTCC-- TAGGCCAACC ATGGAACTTG  [16400]
Pcrbsap    .ATC.TT... ..A.TC.AAA .GG.GAT.TT ..AGGGGG.. .-CAAAA... GT.G..GA.T ..GG.G.TAT TTTT..T.GT .TCATTTTTG T.AATGT..T  [16400]

Pcrbsa     AAAGAATTAT ---CAAAAAT TGGAACTTTA TAAAAAGCGT TTAAAAGGGG GAATATACCC GAGTGCACAA CAGGAAGTGT GCTCGAAAAT GCGCTGAGCA  [16500]
Pcrbsap    ...A..G.G. GCGT...... GT....AGA. .TGG.G.A.. .GC.G.T--- -...GCGT.. TTACTTG.GG TT....A.A. ---.A.CTG. T..G..GA.G  [16500]

Pcrbsa     CATGGCCGAT CAGATTTGCA ACGTTCACAC ATTATCATAA TGTTGAAGAG AAAGGAGGAA ACTTCGCCCA TATTCCCCAT CTTCAACATT TTTTAGCATC  [16600]
Pcrbsap    .T.T.ATTTA GCAC.C.C.. TTT.GTG... T..GC...C- CACC.CGCTT TGGA.GTAGT ...CG..TTC C..CTG.T.. G.GTT..T.. AAC.G.GTGA  [16600]

Pcrbsa     ATGCTTAGCT CAGCACAACA TATTTGTTAA AAAAAAAAAA TTGAAATAAA TGTTTATTCA TTATATTTAA CTCTTTTAAA ATATAACCAC -TATCGACAT  [16700]
Pcrbsap    ...AA.TC.. .CCAG.GTT. .G..GTA.C. ..TTGG.CT. C.A..G.TG- C.CC..---. AAG.G.CCTC .......CGC GCT.CTG..T AC.G.A....  [16700]

Pcrbsa     GTCAATTTGA GCACATTGAA AATGAGTCAT ATCTGCTAAA CCGCCAAATC TGAATATTTT TTAAAAAGGG GTGCCTCTGT GTATAAATTA AAATACCCCC  [16800]
Pcrbsap    ..---...A. A.-----... ..AAG.A..A ..-------- -----GGT.T .CC.GG..CA ...G....-- ------TCA. CG....C..T .C.GT.ATGT  [16800]

Pcrbsa     AAATGAGAAG ACTAAAAGGA GATGGATCAG CATAATATGG TCATTTTTTT TAAATTGTCA AACGTTGCCA CAACTGCTTA GGAAGCCGTT CCATGTTCAG  [16900]
Pcrbsap    ...GTGC.C. .TGG..C.C. A.AATT.... G.GTG....A ATGC.C.A.. .C-------- ---------- .G.G..AAA. AA.G.A.AAA ....--...A  [16900]

Pcrbsa     ATGCTTCTTC GAATATGAAA ATGTGTACGT GTTTTTTTTA TTGACACATG TGCGAAGTTA AAAAATATTT TCCCCATTTT GTCTTTTTCT CCCTTTAGAG  [17000]
Pcrbsap    ....G-.... .CGCG---G. ...C.C...A .AG..AAA.G ..A.T---.. G.....TA.G GGGG..G.GC .----..CAC .CAGGAAA.G ------GA.A  [17000]

Pcrbsa     TTGTTTTGCA AAAGAATAGG GTAAAAGGCA GAAAAAATGA TCGTATGAAT GTCTTTATGC TAGAGTGCAT ACCTTTGTCA GCCGCTGATC AAAATGGGAA  [17100]
Pcrbsap    A..A.CCA-. ..T..GC..A AAGGC..A.. -..T..G.A. AAAGGC.G.C AAA.GAGAAA A.AG.CAG.C .AA.GA.CA. AAAA.GATG. GGT..AAA..  [17100]

Pcrbsa     CAAATGAGTG TGTCACCTTT TAGTACATAT TTTAAAAAAA AAAAAAGGAA GGTCCAAAAA AGGACCGACT TACGTATGTA ACATCTAATG GTTTACTTGG  [17200]
Pcrbsap    TC.CAA.CGA A.....T.C. --.G...GCA GACG..CT.G G.GTG..CGC C.CGTC.C.G C..GGA.G.A CC.T..A.GG C..C.G.G.- -...GA.---  [17200]

Pcrbsa     ACAGAAAAAT TTAAGGAGAT ATACTAAACG AAACAATTTA TAAAATTTGC TGTTCGTCCA TTTTGGCACA CGTTTCGC-T AATTGAGGAA TTAAAATGAT  [17300]
Pcrbsap    -----..... ..---..AT. ..C.AG..GA G.G.G....C .C.C.AAA.T GAA.T-..G. A..A..A.G. T.GCAAATA. .G.AC..CGG .AG......A  [17300]

Pcrbsa     TAATGCTAAT GTGGGCACTG CGTAATGAGC ACATCTGGAT GTAATAAAAT TATCTATCTA TCTATCTAGC TATCTATTTT TTTTTTTTTA GCCAAAGAAG  [17400]
Pcrbsap    .TGC..C.CC AG...-G... .T-------- -...T...TA AGG.AGG.GC ..A.C..--G .T.GGA.... -----G.GG. ......C.C. C.----ATT.  [17400]

Pcrbsa     GGTCCTATTT TATAAGACAA CCATTTCGAA TCCGTTCAAC GCTTCAAATC CGATTTGCTC GCATAAGTGT TAAGGTTGTT AATGCTTTCC GAGTTAGGTA  [17500]
Pcrbsap    ...TTC.... A..T....CG TTT.G.G.GG G--....TG. TT..TGC.GA GCTA..ATG. A...-----. CCT..CG... .G.A....TT C.AC.GAT.G  [17500]
```

```
Pcrbsa      CACAAAGTTG AAGTGGGGAA AGGAGAGGGG AGGGTCATCA TTATGATCCC CCCAAAACTT AGTAATAAAG TTTATATTGA AAAATTCAAG ACATTATTTT  [17600]
Pcrbsap     .G....A.-- ---------- ---------- --A......C AC..TT.TTT .T-------- ---------- ...CCGA..T G..G...--- -------.GC  [17600]

Pcrbsa      AGTTTTTTTT TTTTTTTCAC TCATATGGTT TGAACATTCA AGGAATATTT CGAGCTGACG ACTCGACTTA GTAGAACTTG TGTTTTGACC GCGAAAGTGT  [17700]
Pcrbsap     CACC.A...A ....A.C.G. -.....AT.. CTT.A..... -----.CA.. A..AT.AGAT T.CTTCT... .-.A.GT.G. CAAAG.CGT. TAA.TC..T.  [17700]

Pcrbsa      GTATGTAAAG TTATGCACTT AAGGAGTAAA AAG--AATTC TCCCCCACCC TTTGTTGCAT CTACTTGCAA AGATGAGCTA ATTTTTATTC TTTTTAAAAA  [17800]
Pcrbsap     .C...C.TTT .C..CA.A.. GCTCGTC.GG .T.TC.C.A. A.TG.....A G.AT...G.. ...TA.T.C- ---.CTCT.. .A....T... ....GC...T  [17800]

Pcrbsa      AATATAATAA TAAAAAGTAG GCTATCCATT TTTATTTAAC CCTAATGCTC TTTTCTTTTT GCACCATATT TGGAATGGCG CAAAGGTAAA AAAAAAAAAT  [17900]
Pcrbsap     G.C.G.G... A..CCCAC.. CTC...A.CC A.A....G.- ---...TT.. ..C.TAGA.A ATCGTG...A .C...--.TA ...TT.G.TC .TCTCTTCT.  [17900]

Pcrbsa      GCGGGATTCA TATTGTAAGT G-ACAGTTGG TTCACTCGTT ACCAAACAAC AGAACAATTT GCACAAACAG ATTGTCAAAG GATTGACAAG GAAATTGAAT  [18000]
Pcrbsap     .G.TC..A.. .GG.CCT.A. .C.TCAA... .GTC.CATA. TT.TGTTTT. ...T.--... CTT...CGG. G.------.. A...TG.TG. A.TG..TTT.  [18000]

Pcrbsa      AGTTGCGTTT ATCCTAAGTG GTATATTCTT TGCATTAAAG CATTTCCCCC TTTTAACCAA ATGGTGAGCC ATTTTTTTCC TGCATTCAAA AATAGAAATG  [18100]
Pcrbsap     G...C.--.. ...T.---.T ..T.T..G.. ..AC..TTGT .GGGGT.T.T .C..--.TGC GCT.CT.T.. T..G.GAAG. AT.T..TT.. GT.TACG...  [18100]

Pcrbsa      TATTTTCATT TGCTCTTTAG TGTACTTAAA TGTGACTAGC AAAATGTCAC TAGTACCCAT ACACGTGGTG GAGTTTAGTC AATTGTTTTT TTTATTATAT  [18200]
Pcrbsap     A.CGCG.TCA AA---...C. .C.....G-- C...G..T.- -----..TGT G.T.TTT.G- --GTT....T .CT.C.GT.A GT...GGCG. .C....GGGG  [18200]

Pcrbsa      TAAATATTTA CTTAGCCTTT TCCTCACAAT GAATTAAGGA AACATAATTA GAAAGGTTGA AAAAAGTGTG TAGTAGTTTC TTCCTGCCAA ATGCTCGCAC  [18300]
Pcrbsap     G.GGGG.A.G .G-.TTTG.A AAG.GG.GG. A..G.TGAAC .GAG.GG.G. A.....A-.. ...TGT...T GTAA..A.GG ..AG...T.- ----......  [18300]

Pcrbsa      GTTTTAAAAT CACTAGCACG AAAGGGAGAC AACGGATGGT -TAGAAAAAA GAACGGTCGT TCTACAGTAT TGAACCAAAG GGCTGTCCCC ATTTTTGTAA  [18400]
Pcrbsap     .-----G.G. G..A...GTA G.TA.A.TG- ...A.CCA.. G.GC.CT..T TGG.A.--.A A.A.A....- -----..T.T ACGCA..TA. GAG.GC...C  [18400]

Pcrbsa      TTTTTAAAAG TGCCCGAATA TTACGTATCA TGAGTTCGCC TACCAGTTTA TAAGCGCAAC TGTGCACATC CATGTGGGCG GACGTACTAT ATATAACATA  [18500]
Pcrbsap     ..ACC.G... GAGAA...A. AG.A.G.CTG AA..------ -.AAGA.C.T C..A..---- -------G.T G..ACA...A A.------.. G.-.GGA..T  [18500]

Pcrbsa      ATGTGGGGAT CCTTTATAAC CTCTTTAAAC ATAATAAAAG ATATACCCCC CATTTATCGT TTTCTTCAGA GGGGTTTCTT TCATTGGTTT ATTGGTGTGA  [18600]
Pcrbsap     C...T.CCCC TT...T.TC. ..A.A.TG.T G.G.AC.TGT GC...A..T. .TGG.GG.A. .CATC.T.CT .CAC...... CT.AC.AG.. .--.CC.C.C  [18600]

Pcrbsa      AGAAGAAAAG TATCATATAA TAGGCA-CAT TAATTTAGTG GGTCCTCCCC TAATGTGCAA AATTTTATAG TTAACATTAA AAGGGGGAAG GGGGCGAATG  [18700]
Pcrbsap     T.T..C..TT .GGTG.GC.G .GA...G... AT..A...G. ACCA..G.TA .TTCT.TTT. .GCG.CTC.. A-G..G..T. .CT.A.TC.. T..A.--...  [18700]

Pcrbsa      GATATAGGCC ACTGAACTCA AATGATTAAA AGAGTTGCAC ATATATCTTT CCACATTGCT CATTAGTAAC AACTA--GTT CTTAAAAAAA TGTTTAAATG  [18800]
Pcrbsap     CG.GC..ATT .TCACTT.A. C..T.G-.TC T.T..A.A.. .CG.GGTG.G G.CACC..GG ...A.A.C.A T...TTT.CC T...CT...G CA.AA...C.  [18800]

Pcrbsa      AATAATCCCC ATTTGGCTTT ATCAGATTTA GCTACGTATA AACGAAGTTA TGGTTGTCCA TGGTGAATTA TATTTTTTTT AATGAGTATG CCGATTAGAG  [18900]
Pcrbsap     GGGTG.A..G CA.GAAGAAA ..T.A.AAC. .AGGT.AC.G ---.TG.CA. AA.AAAAAA. AAA.A.GA.T C.C..C.CA. C..AGAG.A. .--.CC.TGT  [18900]

Pcrbsa      GAAGATAAAA AC-TGCTAAA TGGGGTAACG CAGAGCGAAA AAATAAAAGC ATAAATTTTC GTTACGGCAG CGTAATGTTA AAGTTCTAAT TTACTGA--A  [19000]
Pcrbsap     A.C.T..... .GA.T.A..T .AC...CG.T TT.-.T.T.. CG.A....CG .....A---- ---.GAAA.A TT...ATAC. ..T..GC..A AGGAGA.TG.  [19000]
```

```
Pcrbsa     TCAAACTTAA AGTTTTTTTT TTTTTTTTAA CATATTCAAT TTAATGCTAC CTATTTAATT TCAGGTTAGC AGTTTTTTTT TTTTGGAAAA ATACTGAGCA  [19100]
Pcrbsap    .G...T.ATG CACAC..... .........T T....AT.G. A.G.C.--.A TG.G...... .TTT-CAG.. ..G.G.AGCC AGAA..GGT. ..G.AA.ATG  [19100]

Pcrbsa     TATATAATAT GGCTCGCCCC AATGTTATAA AAGCGCCTAC GGTTTTCTTA ACAAATATAA T--ATATATG TCTTCAGTTC AGTTTATATA TATTTTTTTT  [19200]
Pcrbsap    G.G..GG.-- --T.TA.A.. T.AA..GGGG G.T.T..A.A AAC.AGG.AT ..CCC....T .TAGGG...T AT..A...GA .AAA..A.A. .GGA.C.CCC  [19200]

Pcrbsa     TTGCACATTA GTTTTGGTTG CCTTGCGTGC ATAATATATC ACATTTTTAT ATCAATTTTT TTTAACATTT GCTCGCATCT GTTGTCTAAT TAATATTTGT  [19300]
Pcrbsap    CCCTGACCC. .....--..C A...AAAAAT GGGGA..-.. .A....A.GA TA..CACG.G AG.C.G..CA CTCTAA.GGG C..ACA.CC. AC.A..G...  [19300]

Pcrbsa     CACGCTATAA TATAAAAGGA AAATGGTTGC AGAATTAAAT GCGTAAAGCA GATGTTTCAT TTTGGTAACG GTTATTATTA AAAAAGAGAG TGAAAAGATA  [19400]
Pcrbsap    G.A.-..... A.A.TT..A. ...CTT..C. TTG..A.TTC ..T.T.TA-- -..TC.AGC. ...AAA.TTT A..C.CT... T...GA.A.T ..CG.GTTCG  [19400]

Pcrbsa     ATTTTTTAAA GCTTATTTTG ACAACCAGGA AGCATAAAAA AACTACGTCA TTATTTTGTA CAACCACTGT TCAGCAAAAC ATAAAAAAAA AGCATTGAAA  [19500]
Pcrbsap    C....GCGCG A..------- -....TGC.C GCTG.GC..T TGG.GA..TG .A...A.A.. T..ATC.C.. ..TAT....A T.GCGGC... G.----.GC.  [19500]

Pcrbsa     GAAAATGGGC GCTGATCATG GCCATCA-TA ACTATATCTT GGAAC--TGT TGCGAATTAA AAAAAAAATA ATGTATTAAA GGAATGGACG TATTATGAAT  [19600]
Pcrbsap    .TT.T.TTTT T..C.A.C.T ....C.TC.T .....CAGAA AA.GTGA..G A..A.CG.G. .G..T....C -..CGC.T.G .TT.G.TG.C G.C.GG.G.G  [19600]

Pcrbsa     GCGATT-TAT TTATTTTTTT TTTTTATAAA GATGTTGTAA AAATTGTTAA TCTGTTTTCA CGAATAATAT TTGTTTAAAA AAATGAACGC GCACTAA---  [19700]
Pcrbsap    ...GC.ACG. ..GCGCC.A. GAC..TG.GT .T.TG.AGTG .TC.C.G..G .GC..CAC.. GCG.A..C.A C.T.C.CTT. CTTCCTT.T. .TT..T.TGC  [19700]

Pcrbsa     ----GAAGTG TTCTTT--TT AAAAAAAAAA CCCCCGTCG- --CGATACAG ATTCGCGTCC GCATACCTGC TTATATGATA A-GGTATGCG TAACCTAAAA  [19800]
Pcrbsap    GTCCA..AC. ..T..AAAA. G...C..T.. TG.TA..TTT TTT.GGGGG. GCGAATTATA A..C.T...G .G.A...TC. CCAA.T..G. .G.TA.TTG.  [19800]

Pcrbsa     TTAAA---GA TTATTCTTTT TCTTTCCTGA AGTTATTA-- ---TGCTTTT AAGAGTATTT TTATTTTA-- AAAAATA--- -CC------- ATATATTTAA  [19900]
Pcrbsap    .CC..TCAAG G..AG...CA .T...T.A.. TT..C.C.CT TTC.C..... .CT.A..C.G C..A...GTG GG..GC.GCC C..CGCCGTG ..T.C...GT  [19900]

Pcrbsa     AAAAACAAAA AAGT-TACTC GTATGCATAA TAAATACATA TTTATAATTA TATTATACGT TGCACTTTTN AAAAAAGAAT AGAGCTCAGA TAATATTTGT  [20000]
Pcrbsap    G..C...C.T GT..AC..C. C.GA..GAC. A...A.A.A. A.ACCC..C. G..C.G.TT. GTTT.GCC.G .GCG..T..G C..AA.--.G A..GGA..A.  [20000]

Pcrbsa     AGTGTCATAA TTTTTCTGAT ATTCCATGTC CTTTCATATG ATTACTTAGA TTACGTATAT TTTTATATTC ATTTTAAGAA TTAAAAAGTT GCAAATCTGT  [20100]
Pcrbsap    GAAA.G..TT C.CAG.AA.. .CGG...AAA AACC.GAG.. GGA.A.A.A. .A.-A.TC.G C.G..AGAAA .G..A.CC.. --..GT.T.C A.G..A.A..  [20100]

Pcrbsa     TTTNNNTCAA ATATTAAGTG CTGTGCTTGA GATATTCTAC TACAGAAAAA AAAGACCATT TATTTTTTTA TTTCA----G ACGTGGTAAT TGAATGAAAT  [20200]
Pcrbsap    AAN....... .......... .......C.. .......... .......... .......... .......... .....TTTT. .......... ..........  [20200]

Pcrbsa     ACGAGATGAA AGCAATAATG AATGGTTCCC TTTGTTTAAG GAAAATTTTC CTGTGTTCTA TTTTCGTATT GGTATATCTC AGTTCTGCTA AGGTAAGATA  [20300]
Pcrbsap    .......... .......... .......... .......... .......... .......... .......... .......... .......... ..........  [20300]

Pcrbsa     AGGTCGCGTT GAAGAGCGCT AGCTGTCGTC CTCACGCAAT GCGTTACGCT TTTGAGTGTG CACGGGCAGA TGGGTAGACG GACAAAAAGG CAGCCTATAA  [20400]
Pcrbsap    .......... .......... .......... .......... .......... .......... .......... .......... .......... ..........  [20400]

Pcrbsa     AATAAATAAA CTTGTACATA ATATGTACGT GTTTGGGAGA TACATTACCC CGTTTACTCA GTATCCTCCC TTTCCTCCTC TGTTCAGTGT GCGTACCGAG  [20500]
Pcrbsap    .......... .......... .......... .......... .......... .......... .......... .......... .......... ..........  [20500]

Pcrbsa     ACGGATCTGA CATATTGTAC AGCGAAGACT CCACATATGA TTTAGCAATC AACGAAGCAT CCACCAACAG CTATGAATCT TTAGCAGCAA GTATTGAATC  [20600]
Pcrbsap    .......... .......... .......... .......... .......... .......... .......... .......... .......... ..........  [20600]

Pcrbsa     TCTAACAGCA AGTAGTGAGT CTCTAGCAGC AGGTAGTGAA TCTCTAGCAG CAAGTAGTGA ATCTCTAACA GGAAGTAGTG AATCTCTAAC AGCAAGTAGT  [20700]
Pcrbsap    .......... .......... .......... .......... .......... .......... .......... .......... .......... ..........  [20700]
```

```
Pcrbsa     GAATCTCTAA CAGCAAGCAG TGAGTCTCTA GCAGCAGGTA GTGAATCTCT AACAGAAAGT AGTGAATCTA TAGCATCAAT TAATGAATCC CTGAATGACT  [20800]
Pcrbsap    .......... .......... .......... .......... .......... .......... .......... .......... .......... ..........  [20800]

Pcrbsa     TTTATGGCAG CGAAGAATTG ATATCATGTG AAGGAGAGCC AAATAAAAAG AGATTCATAG GAGATGTCTT AAGGGATGGA ATATCTGAGG ATGATCTATT  [20900]
Pcrbsap    .......... .......... .......... .......... .......... .......... .......... .......... .......... ..........  [20900]

Pcrbsa     AAGAGAAGAT TTATTTCATG TACAAGAAGG ATCCGAAGAA ATGCTAAATG ACGTCTTAAA AAATCATTCC CAAAAAGATT TATCTACTAG CGAAAATAGT  [21000]
Pcrbsap    .......... .......... .......... .......... .......... ....T..... .......... .......... .......... ..........  [21000]

Pcrbsa     TTATTCGAAG ATGATCAGTC ATTGAAAAGT GGCTTCAGAA AAAACTGCTC AGAAACTAGT CTGGACAGCT ATATGGGAAG TTTTAAAAAT GGAAGATCTA  [21100]
Pcrbsap    .......... .......... .......... .......... .......... .......... .......... .......... .......... ..........  [21100]

Pcrbsa     GACACGGATT GGATATAGAT TCGGATCCAC ATAGAAGGCC TACGCATGGA GGTAATGAAC CACAAGGAGA GGAGAAATCT GTGAACTTCA AAGAACATCT  [21200]
Pcrbsap    .......... .......... .......... .......... .......... .......... .......... .......... .......... ..........  [21200]

Pcrbsa     AGGGAGACAA AGGAACCAAC AAAGGAATAG TGTAAATATA AAATTAAAAA ATTTTTACGG AGAAGAAAAA ATGTATAATT CGAAGAAAAA TAGATTTATT  [21300]
Pcrbsap    .......... .......... .......... .......... .......... .......... .......... .......... .......... ..........  [21300]

Pcrbsa     ATAAAATTTA TGTACTATCT GCCGTTTGTC CCAATTGTTG CAGTGATAAT AATTTTAATG ATTCTTCTAC TCACGACCCC CAAGATTGGT TTGACTTTTT  [21400]
Pcrbsap    .......... .......... .......... .......... .......... .......... .......... .......... .......... ..........  [21400]

Pcrbsa     TGTTATGTAG CCCTGCTGTT ATAGCAGCTT TTATATACGT GATGTATTCA AACAGAAAAC AATTCAAACG TACATTTGGA ACAAGGAGAT AAAAGCCATA  [21500]
Pcrbsap    .......... .......... .......... .......... .......... .......... .......... .......... .......... ..........  [21500]

Pcrbsa     TAAATTAAAA AAGGGAGGCG GGAAAATATC TAGATTTTTT AAAAAGTTAG ACAAAATCTT GTTTGACGAT TTGGATGGCT TTTAATTGGT AAACGGAGTT  [21600]
Pcrbsap    ....AA.... GG........ .......... .......... .......... .......... .......... .......... ...T...T.. ......G...  [21600]

Pcrbsa     GGGTTCATTT CTCAATGCTA TTCTTATGAA TCATTAAAAT TATAATTTAT GCACTTGCTA TCAGTTTGGG AATCATCTAT TCCGAAGTTA AAAAGTATTA  [21700]
Pcrbsap    .......... T......... .......... .......... .......... .......... .......... .......... .......... ..........  [21700]

Pcrbsa     GCATAATAAG CTTTTTTAGT TTGCAAAAAT GTGGATACAT GATAAATTTG TATATGATAT GTTCACATTT TTGTAGGCAT TTTTGAAATA GACTAATGTC  [21800]
Pcrbsap    .......... .......... .......... .......... .......... .......... .......... .......... .......... ..........  [21800]

Pcrbsa     ACTAGGATAG GTCCTATTTC AATGCTGCAT CGTCTTCTTT TTTCTAATTA GTTTTTTCGT TTTCTTTTTT TACATGTCTT CTGAGATTGA CCTTTATGTT  [21900]
Pcrbsap    .......... .......... .......... .......... .......... .......... .......... .......... .......... ..........  [21900]

Pcrbsa     AGAATTATCC TTTCTTCTCT TTCATTTCAC CGCTATTCCA TTTCTCCATA TTTCTGTAAG AATGATACAC GTTTTGTGTG CTTACTAAAT TGTAAAGTCT  [22000]
Pcrbsap    .......... .......... .......... .......... .......... .......... .......... .......... .......... ..........  [22000]

Pcrbsa     TAATAAAATA CTTCAACGCA CCACTGTCTG CATTATTAAT TTTGTTTTTG AAAAAATTAA CATATTGTTA TCCATTTTAT TGAAGAATTT TCACAACTAT  [22100]
Pcrbsap    .......... .......... .......... .......... .......... .......... .......... .......... .......... ..........  [22100]

Pcrbsa     GAGATGGTTA TGAAACGCTT CATACGGATG ATTCTCTTTT AAGACATCTG CGAAGGCGCT TGCTCAGGTT AATTAAAAAG AACGAATCCC CGTTTAGAGC  [22200]
Pcrbsap    .......... .......... .......... .......... .......... .......... .......... .......... .......... ..........  [22200]
```

```
Pcrbsa     ATTAAAAGGT GTTCACATGC TAGCTGTATT TTTTATTATC CAACTGCATG ATTACCTTTT TTGTACACTA TTGCGAAGGA TGGGGGAAAA TATGCAGCTA  [22300]
Pcrbsap    .......... .......... .......... .......... .......... .......... .......... .......... .......... ..........  [22300]

Pcrbsa     AATATTGTTT CATTGTTTGG CAAAGAGAGT GCTATTTTTC AGGGGAACAC AAAATTGTTT TCTGGAAAGG CATATTTAAA TGAATGCAAT AATAATTGAG  [22400]
Pcrbsap    .......... .......... .......... .......... .......... .......... .......... .......... .......... .........A  [22400]

Pcrbsa     TTAACAAATG ATGTGGNGTG TTTTTCTTTG CATTGCGTTC TTCCGATTTG TTATCATAAA AAGGGGAAAT TATTCAAATG TGTAGTGAAG TGATGCCATT  [22500]
Pcrbsap    .......... ......T... .......... .......... .......... .......... .......... .......... .......... ..........  [22500]

Pcrbsa     CTTTTTTTGT GACTATTTTT TTGGGGAGGT TTACACATAC GGCATAACAT CACTGTTAAG TTTGATTTTT TCGGAAAGGT TAACGTGCGG CATGGTAATC  [22600]
Pcrbsap    .......... .......... .......... .......... .......... .......... .......... .......... .......... ..........  [22600]

Pcrbsa     CAAGTGCCAC TACAGTTAGA GCTAATGGAG TGTTTTTTTT TTTTTTTTTA CACAAGCAGC TTCTTCACCT TTGCAGGAAC ATTAATATGT GAACCAAATT  [22700]
Pcrbsap    .......... .......... .......... .......... .......... .......... .......... .......... .......... ..........  [22700]

Pcrbsa     TGTTGTAGGC TTGCCTCTAT TTAGACAGAA CAGCCATTAT GAAGACTAAA AAAATAGTTA ATTCGTTCGA CAGGGACATA CCAAACAATT AGGATAGAGT  [22800]
Pcrbsap    .......... .......... .......... .......... .......... .......... .......... .......... .......... ..........  [22800]

Pcrbsa     TTTATTTTTT TTTTAGCAAT TTCGTAGGTC GCACTTTGAT TTGTTTGCCC GAAGAACAGT CACGGGGAAT CCCATCGAGC GAGCCTATGC AATGTGCTAA  [22900]
Pcrbsap    ...--..... .......... .......... .......... .......... .......... .......... .......... .......... ..........  [22900]

Pcrbsa     CTGAAAAAAA AAAAAAAAAT TAATAAAATA AAATAA-AGT AA-AATAATA TTAATAAAAT TCAGAAATTT TTTTTGTTTG AAATGAACGA GGGTGTTCCA  [23000]
Pcrbsap    .......... .......... ........A. ......T... ..T....... .......... .......... .......... .......... ..........  [23000]

Pcrbsa     AGGTATAGCA ATTTTCATCC CTATTTTTTC AAAATTATGA CGCGCAATAT TTTAAGATAC GAAATTTTTT ACGTAATCAA TTGCTTCATG AACAAATTAC  [23100]
Pcrbsap    .......... .......... .......... .......... .......... .......... .......... .......... .......... ..........  [23100]

Pcrbsa     GAGTTGGTCT TTTAATTTTT TCCTCTCGCA GGCTGCTATA TTGCCTGTAT GATCACAAAA TTGCAATGTT AAATTGGCAG AGAAGAGGAA GTTCAAATTA  [23200]
Pcrbsap    .......... .......... .......... .......... .......... .......... .......... .......... .......... ..........  [23200]

Pcrbsa     TAAACCTGCG CATACAACGA GGATGCCTGT CTTCTCTGAG TTTTTTACAT GCCAGATCAA CATTTACACT TTTGAGGAGT AGTGAGCATT ATCATGTATG  [23300]
Pcrbsap    .......... .......... .......... .......... .......... .......... .......... .......... .......... ..........  [23300]

Pcrbsa     TACACATGTA TGTACGTGTA TATGTGGAAC ATAAAAGGGA ACGGTCCAAA AGGTGAAGTA GTAGGAGCGC AGCCGAGCTA TACCTTTAGA ACAATAGTTC  [23400]
Pcrbsap    .......... .......... .......... .......... .......... .......... .......... .......... .......... ..........  [23400]

Pcrbsa     CTATGCTATG ACTTAAACGG TTTTATTAAA TAATGCCTCA CTGAAGGTGG AAAGACAAAA AAAAAA-TTT CCTTTGTCAT TGTTCTTGAA AATGTGAAAA  [23500]
Pcrbsap    .......... .......... .......... .......... .......... .......... ......A... .......... .......... ..........  [23500]

Pcrbsa     GATGAACCGC GCTTATATAA CGATGATGAT ATGGTATTAA GAACTGATCA GATTTGTTCA TACTACTTCC TACACACACA TACCTTTTCG ATTCATGCAC  [23600]
Pcrbsap    .......... .......... .......... .......... .......... .......... .......... .......... .......... ..........  [23600]

Pcrbsa     AGTACCCATT AGGCGCAAAT GAAGTTTTTT TTCATCGGTC AAAGCAGGCG CAATATGCGT ACATATTTCC TTTTTTTTTT TTTTGAAAAA AAAGGGAAAT  [23700]
Pcrbsap    .......... .......... .......... .......... .......... .......... ........-- -......... .......... ..........  [23700]

Pcrbsa     GTGCATGGCA CAATTTTTGT TGTGTAAACT GGGGAGGAAC TTATTGTAGA GGGAAATTTA AATATGCCAT TTGGGGGGAT TAATTGCGAG CTATTTGACC  [23800]
Pcrbsap    .......... .......... .......... .......... .......... .......... .......... .......... .......... ..........  [23800]

Pcrbsa     TTTTAAGTTT GAAAATGTCC AGGCGTTTTT ATCCGCGATG TGAATGTCCC CCGCCTCAGG AGTTTTATTT CTTTAATGTT GAGGGAAATA TTTACGAGGG  [23900]
Pcrbsap    .......... .......... .......... .......... .......... .......... .......... .......... .......... ..........  [23900]
```

```
Pcrbsa     GGAATTCATT TTTGCATACT TAGAAGCAGC ATGCTTATAT ACATATATAT GTATAAGTAT AAGTATGTAG GTACCTAAAT TGTCGTGCCC TGCTGCGCCG  [24000]
Pcrbsap    .......... .......... .......... .......... .......... .......... .......... .......... .......... ..........  [24000]

Pcrbsa     CGCGAGGCAA TGGCGGCAGG GACAACAACT ATGCGTTTAA AAAATTATTT GTCGGAGCAT TATAAATTCT CCGTTTGTTT AGTGTCATTT CCATGCAGAT  [24100]
Pcrbsap    .......... .......... .......... .......... .......... .......... .......... .......... .......... ..........  [24100]

Pcrbsa     GACCCAACTG AAAGTTAATA CGGTGTATAT ATATGTAATA TATAAGTATC TTTTCTTTGT AAAAGCTGAC AGCAATTTTT TTCGCTTTAC CAGGAGATCA  [24200]
Pcrbsap    .......... .......... .......... .......... .......... .......... .......... .......... .......... ..........  [24200]

Pcrbsa     TGATCTAGGC AAGAGTTGAG TTAATACTTA CGTTGATATC AACATCGATG GCAATATTAG TAAGATGAAC TAGCGCCAGC TCTCTGTTTC GTAATTAGAA  [24300]
Pcrbsap    .......... .......... .......... .......... .......... .......... .......... .......... .......... ..........  [24300]

Pcrbsa     ATCTAAAAAA AAAAAAT-GA ACAAGAGAGA ACGTCTCAAA AGAAAGGAAT AATGAGGAAG AAAATGATGC AAGGATAAAT AAACAATCGT AAAAATATCC  [24400]
Pcrbsap    .......... ......AA.. .......... .......... .......... .......... .......... .......... .......... ..........  [24400]

Pcrbsa     CTCAAAAAAA TCCTTAAACA ACAAAATGGA GATAAACTTC TATACACTCT TCAAAAGGTG ACAATTGGGC TTTAATAGTC TTTATACAAA CAAATGGAAA  [24500]
Pcrbsap    .......... .......... .......... .......... .......... .......... .......... .......... .......... ..........  [24500]

Pcrbsa     AAAAAAAAAT TGCGGTATTA CATAAATATT CTAAAGGAGG GAATATACTA AATGAATTTA ATGCATGTAG CAATCCCCCG GTCCCTCTTT TTACATTTTG  [24600]
Pcrbsap    .......... .......... .......... .......... .......... .......... .......... .......... .......... ..........  [24600]

Pcrbsa     CGAAGAGCCC CTTTTGGTTA CAGCAGAAGG GGGGGAAAAA AAACAAATAC TTTGAGAATA AATTGTTTTC ATTTTAATTA ACGCCTTTGC TTGAGTGAAG  [24700]
Pcrbsap    .......... .......... .......... .......... ..-....... .......... .......... .......... .......... ..........  [24700]

Pcrbsa     TTTATAAATG TATATAAATA TGTAAATATT TATTCACTTA GAAAACAAAA TTGGCGATTC ATTTTTCTAA AATTAAATTG CCCTGTTACA AATAGGGAGA  [24800]
Pcrbsap    .......... .......... .......... .......... .......... .......... .......... .......... .......... ..........  [24800]

Pcrbsa     AGATAAAACA CATTATTTAT TCTCCGGATT TACCAATTTT AAACTCGTTT AAGCGCGTAT CGATTTAAAT TACGGGGGGG AAAAAAAAA- TTAAGTTATG  [24900]
Pcrbsap    .......... .......... .......... .......... .......... .......... .......... .......... .........A ..........  [24900]

Pcrbsa     CAAATGTATA GCCTATCCCT TTTTACGTAC GGTACTAATG TGCTTTTTTT GCATGACAAA CTTGAAGTGT TTTCTTCTCT TCTCCTTTTT TTCGTAATTT  [25000]
Pcrbsap    .......... .......... .......... .......... .......... .......... .......... ...T...... .......... ..........  [25000]

Pcrbsa     TTATGAATTC CCAAATTTAA TGTAAAAATT TGAAATTTAA TTTTATAACA ATTATATCTA TATCGACATG AGCAATAGAA TTATTTTTAG AATACTTCTA  [25100]
Pcrbsap    .......... .......... .......... .......... .......... .......... .......... .......... .......... ..........  [25100]

Pcrbsa     CGCAATAAAG GATGGCCTAT TGTTTGTAAA ACTGCATGAG GCGCTACCTT TTTTTTTTTT TTTTTCAAAA TAAATAGGGT ACAGATATAA TACATTTTTT  [25200]
Pcrbsap    .......... .......... .......... ........-- -------T.. .......... ....C..... .......... .......... ..........  [25200]

Pcrbsa     AAAGTTTTTT TTAATATATT TGGTTAATAT TTATTAAAAA AATTTATATC ACTAGGAATA TCAAATTTTA TGAAAATTAA GCTTGCTTAA CTAAAGCAAA  [25300]
Pcrbsap    .......... .......... .......... .......... .......... .......... .......... .......... .......... ..........  [25300]

Pcrbsa     ATAATACATT TTATTTGGCA TATAATTAGG ATTAGGTAAA TATTTTTACT TATAAAAAGC TTTCATAATA TAGATACCTT AGCTCAAGTT TTTGTTTTCA  [25400]
Pcrbsap    .......... .......... .......... .......... .......... .......... .......... .......... .......... ..........  [25400]
```

```
Pcrbsa     AAATTTTTAT CATTCTGTTT TTTATTTCCA AAACAAAATT TCTGTCGAAA CTTTTTTGTT ATTCTTGCCT TATTTCACTT GCTACTGCCT TGCCAGTTAT  [25500]
Pcrbsap    .......... .......... .......... .......... .......... .......... .......... .......... .......... ..........  [25500]

Pcrbsa     ACTAAAAAAA AAAAAATAAA ATAAAATAAA ATAAAAATAA AATAAACAAA AATAAAAATA AAATAAAACA AAAAAAAAAA ATAAGATAAA ATAACAGAAA  [25600]
Pcrbsap    .......... .......... .......... .......... .......... .......-.. .......-.. .......... .......... ..........  [25600]

Pcrbsa     TAACCTCATT CTTATAAATA TTATTAATCT TAAAAACAAA ATGGTTGCAA AGAACGCTTT TTCATGCGAA GTTGCGGAAT TCCAAAAGAA GGAAAAATCA  [25700]
Pcrbsap    .......... .......... .......... .......... .......... .......... .......... .......... .......... ..........  [25700]

Pcrbsa     AACGTCCACA AGTACAGTGG AAAGGCCCTT GCATTAACCC TCCTGTTAGT GCTCATAGGA TGCAGCGCCA ACGTGGTACG TGTATTCTCC CCTGATTTTA  [25800]
Pcrbsap    .......... .......... .......... .......... .......... .......... .......... .......... .......... ..........  [25800]

Pcrbsa     AGAACATATC AAAGCATTCT TCCAAATTAT ACGATTTATT ATTTATTAAA TGGGTATATT ACTTTAAATG TTTTTTTTTT TTTCTATGTA AAAAATGATT  [25900]
Pcrbsap    .......... .......... .......... .......... .......... .......... .........T .......... .......... ..........  [25900]

Pcrbsa     CACCCCTCTC CTTTTTTATA TGTCCCTTTT TTAGCCTACC TTTGAGAATG CAAAAAACTG CGGAAATGGT GTAGAAAAAA AATTCAGTGG AAAAAACATG  [26000]
Pcrbsap    .......... .......... .......... .......... .......... .......... .......... .......... .......... ..........  [26000]

Pcrbsa     AGAATTTTAT TTGTAAA-CA CGGAAATGTT ATGTCATTTG ATAGTGAAGA AAGCTTAGAG GATTTAGACA CGCGATCTAC TACTGTGGGG GGTAACGATG  [26100]
Pcrbsap    .......... .......T.. .......... .......... .......... .......... .......... .......... .......... ..........  [26100]

Pcrbsa     ACACTTTTTC TTATTCCAGT AATGAATCCT TGAACCCATC TGAGAGAGAA AATTTATTAA AAGAAGATGA TGATGATTAT TACTATTATG ATTTTTCAGA  [26200]
Pcrbsap    .......... .......... .......... .......... .......... .......... .......... .......... .......... ..........  [26200]

Pcrbsa     ATTTTTGTCC AAAGAGAAAT TTAACGAGAA TAACTTTATA ACTTTGAATA ACAAATTCAA GGGGAAAATG AAAAAGCCAA TATCTATCCT TTGCGCTGTG  [26300]
Pcrbsap    .......... .......... .......... .......... .......... .......... .......... .......... .......... ..........  [26300]

Pcrbsa     ACATCTGCGA TAGTACTGTA CTATTGCCCA CAAGTGTTGA TTCCTATTAT GATACTGTTA ATCATTTTTG CAATAGGATT GGGTATACAT TTAAAATTCC  [26400]
Pcrbsap    .......... .......... .......... .......... .......... .......... .......... .......... .......... ..........  [26400]

Pcrbsa     TTAACAAACA TAAAAAAAGA GAAAATGATT TCTAGGAAAT AAGAAAGAAA AATTATATAT CCATTTGCTT TCTCATGAGG AGAGATATTA GTGGGGCATA  [26500]
Pcrbsap    .......... .......... .......... .......... .......... .......... .......... .......... .......... ..........  [26500]

Pcrbsa     AAGGGGAAAA AAAAAAAAAA AAACTGCGAA GGGATTATGT GCCGAAATGT TGAAAGGAAA AAGTGCCACT TGGTTATACT GTTATAAGAG TCTTAAATGA  [26600]
Pcrbsap    .......... .......... ..-....... .......... .......... .......... .......... .......... .......... ..........  [26600]

Pcrbsa     TAATATAAAA ATGTTTGCTC TCCATATGCA TACAAAATGT GCTTCAAAAA ATTTGTCAAG AATTTATTTT TACAATTTAA TTTGGGATTT TTTGTTTTAA  [26700]
Pcrbsap    .......... .......... .......... .......... .......... .......... .......... .......... .......... ..........  [26700]

Pcrbsa     CCCATTAGCA GAAGAAAGTA GTACTCCCAA AAAAAAAAAA AAGAAAAAAT GTTTTTTTTT CACGCTTTGT TATTTTGTCG AGTTTTAAGT TTAGACGTCT  [26800]
Pcrbsap    .......... .......... .......... .......... .......... .......... .......... .......... .......... ..........  [26800]

Pcrbsa     GAAGCGAGAA ATGGATTGCC ACCCGCTGTG TATATTTTAA TGTTTATTTT TGCTCCGAAA AAATGAAGCT CTGCACATGT TATATTTATA ATATGTACAT  [26900]
Pcrbsap    .......... .......... .......... .......... .......... .......... .......... .......... .......... ..........  [26900]

Pcrbsa     TTGTCTAATG CGTAATGTTT AAGCTTAGAA AAGGGGGAAC ACTTATATGC CTTCATTTAT TCTCTTTTGT TTGAATCCGT TAATTTGCTT TTATTTGTTT  [27000]
Pcrbsap    .......... .......... .......... .......... .......... .......... .......... .......... .......... ..........  [27000]

Pcrbsa     TNTGAAAATA ATGCATGTAA TTAATCAACT CGTAATATAT ATTCGCTTTG AACTTTTTAA TTACGATTTC GCAATATGGA TATTTAAAAA AA-GCCTCAC  [27100]
Pcrbsap    .T........ .......... .......... .......... .......... .......... .......... .......... .......... ..A.......  [27100]
```

```
Pcrbsa      TATGTGATGA GAACATGATG CATCTTTTTA ATCGATCAAG TTTTGACTTG AAACAAATAA TGCATCCACT GAGCCGAATG CAGTGTTGGT CGAAGTATTT  [27200]
Pcrbsap     .......... .......... .......... .......... .......... .......... .......... .......... .......... ..........  [27200]

Pcrbsa      TCTAAAAAAG AGATATATGC CATACGAATT ACCTTTCTAA TTTGTTATGT TTAAAAAAAA AAAAAGTTGA AACTTGAACT GAGCAGAGAC AAATGCACCC  [27300]
Pcrbsap     .......... .......... .......... .......... .......... .......... .......... .......... .......... ..........  [27300]

Pcrbsa      TTGAAATTGT TTGTATGGTT GTACATATTT ACATGAATGT TCTGATCATT CTTTAAATAA TAATAATAGT TTAATCTTGC TAATGGTCCA TTTTGTTTGG  [27400]
Pcrbsap     .......... .......... .......... .......... .......... .......... .......... .......... .......... ..........  [27400]

Pcrbsa      ATGTATATAC CTTTATATCT ATCTCAAAAA AGTTGAATAT TTTAAATATG AAATAACTTA AGGTGACCTC AGTTGTATGA ATGATAATAT CTTTTTGTTA  [27500]
Pcrbsap     .......... .......... .......... .......... .......... .......... .......... .......... .......... ..........  [27500]

Pcrbsa      AATGGTTTTG ATTTGTCTTC TGAATTACGG TCACTCATTT TAAGATGGTA CGATAATTGG TTAACCATTT GATCGACACA CAACATGGTT TAGTTATGAA  [27600]
Pcrbsap     .......... .......... .......... .......... .......... .......... .......... .......... .......... ..........  [27600]

Pcrbsa      ATTCATGGGC AATGTGGAGC TATCTTTTTT TAGGGGTTAT TTTTAGCTCA TACCTATGGA ATCAAAAAAT TTTATCATTT TCGTTGCATG AGTGAACCTA  [27700]
Pcrbsap     .......... .......... .......... .......... .......... .......... .......... .......... .......... ..........  [27700]

Pcrbsa      ATAGAAAGTA AACTGTCAAT ATTTAATTTT GCATAATGTC AGTGAGTGCT ATTTTTTCAT TGATACCACA CGGTGCAAAT GAAATTAATG AAGCATTACA  [27800]
Pcrbsap     .......... .......... .......... .......... .......... .........- ---------. .......... .......... ..........  [27800]

Pcrbsa      GATGTGACAG GATGAAACGT TAACCTCAAG AATACGATGT TCTAATAAAA TAAATGGTAA GGATTTAATT ACTGTACTCT TGCAAAGGGT TGTGGAATTA  [27900]
Pcrbsap     .......... .G........ .......... .......... .......... .......... .......... .......... .......... ..........  [27900]

Pcrbsa      CCAATATAAT TGGTGTAGCC AACTCAGTAG ATATACAATC AGTTAACTAT ATGTATAAAT CGCAAAAATG AATGAATTTC TCTACTTACA TGTTATGATA  [28000]
Pcrbsap     .......... .......... .......... .......... .......... .......... .......... .......... .......... ..........  [28000]

Pcrbsa      GCATATTTTT TGGGCGAAAA AAGGGAAATT TTTATCAGCT AATTAAAAGC GAACAAACAG CGGTTATATC ATAAAAGCAC TTGCACTGTG TCAAGCGCAC  [28100]
Pcrbsap     .......... .......... .......... .......... .......... .......... .......... .......... .......... ..........  [28100]

Pcrbsa      AAAATTTATA GTCACACAAG TACAGGAATA TATTAGGGGA AATTTATTAC ATATTTTATG TTATAATCAT GTAAAAATTA GCACAAAAAA AGATATGTTA  [28200]
Pcrbsap     .......... .......... .......... .......... .......... .......... .......... .......... .......... ..........  [28200]

Pcrbsa      TGATTTAAAG ATACAATGAA GTGTAAATTC CTAATATTAA AGGAATTACG AATGATAGCA CTAGAGCAGG GGGGCTGGTT ATCATTTTTT TTTTTTT--A  [28300]
Pcrbsap     .......... .......... .......... .......... .......... .......... .......... .......... ...G...... .......TT.  [28300]

Pcrbsa      ATTCTTTTAT ATGTAAAATT GGAAGAATGG AGACACCTGG ACAGTTTGTG CAAAATGAAA TACAGCATAA TAGTCATTAC ATTGATGTAA TTTATATCTC  [28400]
Pcrbsap     .......... .......... .........- .......... T......... ..G..A.... .G........ .......... .......... .........T  [28400]

Pcrbsa      CTTTGTGATT ATAACCAGTG GGAATTATTT CTGCATAAAA ATATTTGAAT AATTATTTTT ATTGAAAAAA AGAATGTCAC TGTATAAATA AGTAAAGCCA  [28500]
Pcrbsap     .......... .......T.. .......... .......... .......... .......... .......... -......... .......... ....G.....  [28500]

Pcrbsa      ATTTTTATGT TATCCATAAA ATTTACCTAA AAGAAGCAAG AAAAATAACT GTGGGACAAC CCGAAGTGGT CACAAATAAG AACCAAACTA CAAAGGTAAA  [28600]
Pcrbsap     .......... .......... .......... .......... .......... A.T....--- -------... .......... .......... ..........  [28600]
```

```
Pcrbsa     CAAATTATTA TGAAGTACTT TTTTT----- ---GAAAACC AAAACTTATG CTATTCTGGA ATGAAAATTC TATCAGTATG AACGTTTTAG TTTACGGGGA  [28700]
Pcrbsap    .......... .......... .....TATTT TAA.....TA ...T...... .......... ......G... .......... .......... ........-.  [28700]

Pcrbsa     AAAAATAATT ACTATAAAAT GATACTGGAA TGATGTGAAA AGAACAAAAA TTAACAATAC ATATGAACAT ATACATCTGC TTACGAAATA ACATTCAAGG  [28800]
Pcrbsap    .......... .G........ .......... .......T.. .......... .......... .......... .......... .......... .......T..  [28800]

Pcrbsa     GGAAACAATG CAGTCCATCT ATGGAACAAA TGCAGACTTA ACGATTAAAA AGAGAGGACA ATATTACAAG GGGTGGGCGC TATTGGAAAA ATTGACATTT  [28900]
Pcrbsap    T........T ........G. ...C.....T .A........ .......... .A.A...... ........G. .......... .......... ..........  [28900]

Pcrbsa     AAAAGACAAA TAAAAAGGTA AAATGTAAGA ATTCATCTGT TTCTTTCGCG TACAATTGTT CTATTTTGGC ATACATTGTT CAATATATTG TGGTAATTCA  [29000]
Pcrbsap    .......... .......... .......... .......... .......... .......... .......... .......... .......... ..........  [29000]

Pcrbsa     AACAATTTAT TTTTTAAGGG GGGTAAATCA ACGCTTATGT AACTTTTGTT CTCAGTGAAT CACCAGTTCT TTTAGTTATT TTAATCATTC GCAGGATGTG  [29100]
Pcrbsap    .......... .......... .......... .......... .......... .......... .......... .......... .......... ..........  [29100]

Pcrbsa     CTGGTGCAGA TGGCTTTTTT GTTGCGGCTT ATTTCGCTGC GACCTGTTTT TTCGGTGCAG CTGGTTTTTT CGGTGCAGCT GGTTTTTTTG GTGCAGCTGG  [29200]
Pcrbsap    .......... .......... .......... .......... .......... .......... .......... .......... .......... ..........  [29200]

Pcrbsa     TTTTTTTGCT TTGGATGTTT TTTTGGAAGG TTTTTTT--- -CGGGTTTAA GAATTTCCAT TGAGTACAAT AAAGGCTTTA TGTATTTATC ATAATCTGAG  [29300]
Pcrbsap    .......... .......... .......... .......TTT T......... .......... .......... .......... .......... ..........  [29300]

Pcrbsa     CTTCGAGTAT GTTCTTCTAA TTTTTTGAGG GATTCAGTTA ATTTGTCCAC ATCATTTTGG AATGCCTTGC ATTCGGGAAA TTTTCTTGGT TTGGTAATTC  [29400]
Pcrbsap    .......... .......... .......... .......... .......... .......... .......... .......... .......... ..........  [29400]

Pcrbsa     CATTTGGGGA GTTGTTTTTT TACAAATATT ACTATACTAA ATGGGGTAAT TTAAAAAATT TAATAAATTT GTATATATGC TTTTATACTA TAATAAATGG  [29500]
Pcrbsap    .......... ....G.C... .TTT--...C ...G..T... .....-.... ....G....A ...AC..... .......... .......... ..........  [29500]

Pcrbsa     TGAATGAAAA TAAAAAAAAA TATATGTATG AATATATATA TGGGAAAAAT ATATTAGACA TAGGAGCAAT GACTAACTAA TTGATCAACC ACACTTCCAG  [29600]
Pcrbsap    .......... .......... .......... .......... .......... .......... .......... .......... .......... ..........  [29600]

Pcrbsa     AACATGAGAC GAATAAGGAG GAAAAAATAA ATAAGGATGC GTATAAAGCT TTCATTTTTG ATAGTATATG ATTATTTTTT ATTTTTAATT TTAAATTGGG  [29700]
Pcrbsap    .......... .......... .......... .......... ......TT.. .......... .......... .........- ------.... ..........  [29700]

Pcrbsa     GTTAGCTCTA TTTTTTTTCG ATTTAGTAAT AAACTATTAT ATTGCTTTGC CTTAGTATTG CGA [29763]
Pcrbsap    .......... .......... .......... .......... .......... .......... ... [29763]
```

**E**. Alignment of the *P. cynomolgi*, *P. inui*, *P. fragile* and *pvrbsa* CDS haplotypes found worldwide.

```
H_1           ATG AAA GGA ATA ATG AAT GGT TCC CTT TAT TTA AGG AAA ATT TCC CTG TGT TCT ATT TTC GTA TTG GCA TAT CTC AGT TCT GCT AAG TGC GCG TAC CGA  [  99]
H_2           ... ... ... ... ... ... ... ... ... .G. ... ... ... ... ... ... ... ... ... ... ... ... ... ... ... ... ... ... ... ... ... ... ...  [  99]
H_3           ... ... ... ... ... ... ... ... ... .G. ... ... ... ... ... ... ... ... ... ... ... ... ... ... ... ... ... ... ... ... ... ... ...  [  99]
H_4           ... ... ... ... ... ... ... ... ... .G. ... ... ... ... ... ... ... ... ... ... ... ... ... ... ... ... ... ... ... ... ... ... ...  [  99]
H_5           ... ... ... ... ... ... ... ... ... .G. ... ... ... ... ... ... ... ... ... ... ... ... ... ... ... ... ... ... ... ... ... ... ...  [  99]
H_6           ... ... ... ... ... ... ... ... ... .G. ... ... ... ... ... ... ... ... ... ... ... ... ... ... ... ... ... ... ... ... ... ... ...  [  99]
H_7           ... ... ... ... ... ... ... ... ... .G. ... ... ... ... ... ... ... ... ... ... ... ... ... ... ... ... ... ... ... ... ... ... ...  [  99]
H_8           ... ... ... ... ... ... ... ... ... .G. ... ... ... ... ... ... ... ... ... ... ... ... ... ... ... ... ... ... ... ... ... ... ...  [  99]
H_9           ... ... ... ... ... ... ... ... ... ... ... ... ... ... ... ... ... ... ... ... ... ... ... ... ... ... ... ... ... ... ... ... ...  [  99]
H_10          ... ... ... ... ... ... ... ... ... .G. ... ... ... ... ... ... ... ... ... ... ... ... ... ... ... ... ... ... ... ... ... ... ...  [  99]
H_11          ... ... ... ... ... ... ... ... ... .G. ... ... ... ... ... ... ... ... ... ... ... ... ... ... ... ... ... ... ... ... ... ... ...  [  99]
H_12          ... ... ... ... ... ... ... ... ... .G. ... ... ... ... ... ... ... ... ... ... ... ... ... ... ... ... ... ... ... ... ... ... ...  [  99]
H_13          ... ... ... ... ... ... ... ... ... .G. ... ... ... ... ... ... ... ... ... ... ... ... ... ... ... ... ... ... ... ... ... ... ...  [  99]
H_14          ... ... ... ... ... ... ... ... ... .G. ... ... ... ... ... ... ... ... ... ... ... ... ... ... ... ... ... ... ... ... ... ... ...  [  99]
H_15          ... ... ... ... ... ... ... ... ... ... ... ... ... ... ... ... ... ... ... ... ... ... ... ... ... ... ... ... ... ... ... ... ...  [  99]
H_16          ... ... ... ... ... ... ... ... ... .G. ... ... ... ... ... ... ... ... ... ... ... ... ... ... ... ... ... ... ... ... ... ... ...  [  99]
H_17          ... ... ... ... ... ... ... ... ... .G. ... ... ... ... ... ... ... ... ... ... ... ... ... ... ... ... ... ... ... ... ... ... ...  [  99]
H_18          ... ... ... ... ... ... ... ... ... .G. ... ... ... ... ... ... ... ... ... ... ... ... ... ... ... ... ... ... ... ... ... ... ...  [  99]
H_19          ... ... ... ... ... ... ... ... ... .G. ... ... ... ... ... ... ... ... ... ... ... ... ... ... ... ... ... ... ... ... ... ... ...  [  99]
H_20          ... ... ... ... ... ... ... ... ... .G. ... ... ... ... ... ... ... ... ... ... ... ... ... ... ... ... ... ... ... ... ... ... ...  [  99]
H_21          ... ... ... ... ... ... ... ... ... .G. ... ... ... ... ... ... ... ... ... ... ... ... ... ... ... ... ... ... ... ... ... ... ...  [  99]
H_22          ... ... ... ... ... ... ... ... ... ... ... ... ... ... ... ... ... ... ... ... ... ... ... ... ... ... ... ... ... ... ... ... ...  [  99]
H_23          ... ... ... ... ... ... ... ... ... .G. ... ... ... ... ... ... ... ... ... ... ... ... ... ... ... ... ... ... ... ... ... ... ...  [  99]
H_24          ... ... ... ... ... ... ... ... ... .G. ... ... ... ... ... ... ... ... ... ... ... ... ... ... ... ... ... ... ... ... ... ... ...  [  99]
H_25          ... ... ... ... ... ... ... ... ... .G. ... ... ... ... ... ... ... ... ... ... ... ... ... ... ... ... ... ... ... ... ... ... ...  [  99]
H_26          ... ... ... ... ... ... ... ... ... .G. ... ... ... ... ... ... ... ... ... ... ... ... ... ... ... ... ... ... ... ... ... ... ...  [  99]
H_27          ... ... ... ... ... ... ... ... ... .G. ... ... ... ... ... ... ... ... ... ... ... ... ... ... ... ... ... ... ... ... ... ... ...  [  99]
H_28          ... ... ... ... ... ... ... ... ... ... ... ... ... ... ... ... ... ... ... ... ... ... ... ... ... ... ... ... ... ... ... ... ...  [  99]
H_29          ... ... ... ... ... ... ... ... ... .G. ... ... ... ... ... ... ... ... ... ... ... ... ... ... ... ... ... ... ... ... ... ... ...  [  99]
H_30          ... ... ... ... ... ... ... ... ... .G. ... ... ... ... ... ... ... ... ... ... ... ... ... ... ... ... ... ... ... ... ... ... ...  [  99]
H_31          ... ... ... ... ... ... ... ... ... ... ... ... ... ... ... ... ... ... ... ... ... ... ... ... ... ... ... ... ... ... ... ... ...  [  99]
H_32          --- --- --- --- --- --- --- --- --- --- ... ... ... ... ... ... ... ... ... ... ... ... ... ... ... ... ... ... ... ... ... ... ...  [  99]
H_33          --- --- --- --- --- --- --- --- --- --- ... ... ... ... ... ... ... ... ... ... ... ... ... ... ... ... ... ... ... ... ... ... ...  [  99]
H_34          --- --- --- --- --- --- --- --- --- --- ... ... ... ... ... ... ... ... ... ... ... ... ... ... ... ... ... ... ... ... ... ... ...  [  99]
H_35          --- --- --- --- --- --- --- --- --- --- ... ... ... ... ... ... ... ... ... ... ... ... ... ... ... ... ... ... ... ... ... ... ...  [  99]
H_36          --- --- --- --- --- --- --- --- --- --- ... ... ... ... ... ... ... ... ... ... ... ... ... ... ... ... ... ... ... ... ... ... ...  [  99]
H_37          --- --- --- --- --- --- --- --- --- --- ... ... ... ... ... ... ... ... ... ... ... ... ... ... ... ... ... ... ... ... ... ... ...  [  99]
H_38          --- --- --- --- --- --- --- --- --- --- ... ... ... ... ... ... ... ... ... ... ... ... ... ... ... ... ... ... ... ... ... ... ...  [  99]
H_39          --- --- --- --- --- --- --- --- --- --- ... ... ... ... ... ... ... ... ... ... ... ... ... ... ... ... ... ... ... ... ... ... ...  [  99]
H_40          --- --- --- --- --- --- --- --- --- --- ... ... ... ... ... ... ... ... ... ... ... ... ... ... ... ... ... ... ... ... ... ... ...  [  99]
H_41          --- --- --- --- --- --- --- --- --- --- ... ... ... ... ... ... ... ... ... ... ... ... ... ... ... ... ... ... ... ... ... ... ...  [  99]
H_42          --- --- --- --- --- --- --- --- --- --- ... ... ... ... ... ... ... ... ... ... ... ... ... ... ... ... ... ... ... ... ... ... ...  [  99]
H_43          --- --- --- --- --- --- --- --- --- --- ... ... ... ... ... ... ... ... ... ... ... ... ... ... ... ... ... ... ... ... ... ... ...  [  99]
H_44          --- --- --- --- --- --- --- --- --- --- ... ... ... ... ... ... ... ... ... ... ... ... ... ... ... ... ... ... ... ... ... ... ...  [  99]
H_45          --- --- --- --- --- --- --- --- --- --- ... ... ... ... ... ... ... ... ... ... ... ... ... ... ... ... ... ... ... ... ... ... ...  [  99]
H_46          --- --- --- --- --- --- --- --- --- --- ... ... ... ... ... ... ... ... ... ... ... ... ... ... ... ... ... ... ... ... ... ... ...  [  99]
H_47          --- --- --- --- --- --- --- --- --- --- ... ... ... ... ... ... ... ... ... ... ... ... ... ... ... ... ... ... ... ... ... ... ...  [  99]
H_48          --- --- --- --- --- --- --- --- --- --- ... ... ... ... ... ... ... ... ... ... ... ... ... ... ... ... ... ... ... ... ... ... ...  [  99]
H_49          --- --- --- --- --- --- --- --- --- --- ... ... ... ... ... ... ... ... ... ... ... ... ... ... ... ... ... ... ... ... ... ... ...  [  99]
H_50          --- --- --- --- --- --- --- --- --- --- ... ... ... ... ... ... ... ... ... ... ... ... ... ... ... ... ... ... ... ... ... ... ...  [  99]
H_51          --- --- --- --- --- --- --- --- --- --- ... ... ... ... ... ... ... ... ... ... ... ... ... ... ... ... ... ... ... ... ... ... ...  [  99]
H_52          --- --- --- --- --- --- --- --- --- --- ... ... ... ... ... ... ... ... ... ... ... ... ... ... ... ... ... ... ... ... ... ... ...  [  99]
```

```
H_53        --- --- --- --- --- --- --- --- --- --- ... ... ... ... ... ... ... ... ... ... ... ... ... ... ... ... ... ... ... ... ... ... ...  [  99]
H_54        --- --- --- --- --- --- --- --- --- --- ... ... ... ... ... ... ... ... ... ... ... ... ... ... ... ... ... ... ... ... ... ... ...  [  99]
H_55        --- --- --- --- --- --- --- --- --- --- ... ... ... ... ... ... ... ... ... ... ... ... ... ... ... ... ... ... ... ... ... ... ...  [  99]
H_56        --- --- --- --- --- --- --- --- --- --- ... ... ... ... ... ... ... ... ... ... ... ... ... ... ... ... ... ... ... ... ... ... ...  [  99]
H_57        --- --- --- --- --- --- --- --- --- --- ... ... ... ... ... ... ... ... ... ... ... ... ... ... ... ... ... ... ... ... ... ... ...  [  99]
H_58        --- --- --- --- --- --- --- --- --- --- ... ... ... ... ... ... ... ... ... ... ... ... ... ... ... ... ... ... ... ... ... ... ...  [  99]
H_59        --- --- --- --- --- --- --- --- --- --- ... ... ... ... ... ... ... ... ... ... ... ... ... ... ... ... ... ... ... ... ... ... ...  [  99]
H_60        --- --- --- --- --- --- --- --- --- --- ... ... ... ... ... ... ... ... ... ... ... ... ... ... ... ... ... ... ... ... ... ... ...  [  99]
H_61        --- --- --- --- --- --- --- --- --- --- ... ... ... ... ... ... ... ... ... ... ... ... ... ... ... ... ... ... ... ... ... ... ...  [  99]
H_62        --- --- --- --- --- --- --- --- --- --- ... ... ... ... ... ... ... ... ... ... ... ... ... ... ... ... ... ... ... ... ... ... ...  [  99]
H_63        --- --- --- --- --- --- --- --- --- --- ... ... ... ... ... ... ... ... ... ... ... ... ... ... ... ... ... ... ... ... ... ... ...  [  99]
H_64        --- --- --- --- --- --- --- --- --- --- ... ... ... ... ... ... ... ... ... ... ... ... ... ... ... ... ... ... ... ... ... ... ...  [  99]
H_65        --- --- --- --- --- --- --- --- --- --- ... ... ... ... ... ... ... ... ... ... ... ... ... ... ... ... ... ... ... ... ... ... ...  [  99]
H_66        --- --- --- --- --- --- --- --- --- --- ... ... ... ... ... ... ... ... ... ... ... ... ... ... ... ... ... ... ... ... ... ... ...  [  99]
H_67        --- --- --- --- --- --- --- --- --- --- ... ... ... ... ... ... ... ... ... ... ... ... ... ... ... ... ... ... ... ... ... ... ...  [  99]
H_68        --- --- --- --- --- --- --- --- --- --- ... ... ... ... ... ... ... ... ... ... ... ... ... ... ... ... ... ... ... ... ... ... ...  [  99]
H_69        --- --- --- --- --- --- --- --- --- --- ... ... ... ... ... ... ... ... ... ... ... ... ... ... ... ... ... ... ... ... ... ... ...  [  99]
H_70        --- --- --- --- --- --- --- --- --- --- ... ... ... ... ... ... ... ... ... ... ... ... ... ... ... ... ... ... ... ... ... ... ...  [  99]
Pcyn_rbsa   ... ... .C. ... ... ... ... ... ... .G. ... ... ... ... .T. ... ... ... ... ... ... ... .T. ... ... ... ... ... ... ..T ... ... ...  [  99]
Pcyn_rbsap  ... ... .C. ... ... ... ... ... ... .G. ... ... ... ... .T. ... ... ... ... ... ... ... .T. ... ... ... ... ... ... ..T ... ... ...  [  99]
Pinu_rbsa   ... ... ... ... G.T .T. ... .T. A.. .G. ... ... ... G.. .T. ... ... ..C ... ... C.. ... ... ... ..G ... ..G A.. ... ... ..T ... ...  [  99]
Pfra_rbsa   ... ... .C. ... ..A .C. ... ... ... .G. ... .T. ... T.. ..T T.. ... .A. ... C.A T.. ... ... ... .A. ... ..G ... ... .TA A.. ... ...  [  99]

H_1         GAC GAA TCT GAC ATA TTG TAC AGC GAC GAC TCC AGA TCC GAT TTA GCA AGC AAC GGG GCA CCC ACC GAC AGC TAC GAA TCT TTA ACA GCA AGT AGT GAG  [ 198]
H_2         ... ... ... ... ... ... ... ... ... ... ... ... ... ... ... ... ... ... ... ... ... ... ... ... ... ... ... ... ... ... ... ... ...  [ 198]
H_3         ... ... ... ... ... ... ... ... ... ... ... ... ... ... ... ... ... ... ... ... ... ... ... ... ... ... ... ... ... ... ... ... ...  [ 198]
H_4         ... ... ... ... ... ... ... ... ... ... ... ... ... ... ... ... ... ... ... ... ... ... ... ... ... ... ... ... ... ... ... ... ...  [ 198]
H_5         ... ... ... ... ... ... ... ... ... ... ... ... ... ... ... ... ... ... ... ... ... ... ... ... ... ... ... ... ... ... ... ... ...  [ 198]
H_6         ... ... ... ... ... ... ... ... ... ... ... ... ... ... ... ... ... ... ... ... ... ... ... ... ... ... ... ... ... ... ... ... ...  [ 198]
H_7         ... ... ... ... ... ... ... ... ... ... ... ... ... ... ... ... ... ... ... ... ... ... ... ... ... ... ... ... ... ... ... ... ...  [ 198]
H_8         ... ... ... ... ... ... ... ... ... ... ... ... ... ... ... ... ... ... ... ... ... ... ... ... ... ... ... ... ... ... ... ... ...  [ 198]
H_9         ... ... ... ... ... ... ... ... ... ... ... ... ... ... ... ... ... ... ... ... ... ... ... ... ... ... ... ... ... ... ... ... ...  [ 198]
H_10        ... ... ... ... ... ... ... ... ... ... ... ... ... ... ... ... ... ... ... ... ... ... ... ... ... ... ... ... ... ... ... ... ...  [ 198]
H_11        ... ... ... ... ... ... ... ... ... ... ... ... ... ... ... ... ... ... ... ... ... ... ... ... ... ... ... ... ... ... ... ... ...  [ 198]
H_12        ... ... ... ... ... ... ... ... ... ... ... ... ... ... ... ... ... ... ... ... ... ... ... ... ... ... ... ... ... ... ... ... ...  [ 198]
H_13        ... ... ... ... ... ... ... ... ... ... ... ... ... ... ... ... ... ... ... ... ... ... ... ... ... ... ... ... ... ... ... ... ...  [ 198]
H_14        ... ... ... ... ... ... ... ... ... ... ... ... ... ... ... ... ... ... ... ... ... ... ... ... ... ... ... ... ... ... ... ... ...  [ 198]
H_15        ... ... ... ... ... ... ... ... ... ... ... ... ... ... ... ... ... ... ... ... ... ... ... ... ... ... ... ... ... ... ... ... ...  [ 198]
H_16        ... ... ... ... ... ... ... ... ... ... ... ... ... ... ... ... ... ... ... ... ... ... ... ... ... ... ... ... ... ... ... ... ...  [ 198]
H_17        ... ... ... ... ... ... ... ... ... ... ... ... ... ... ... ... ... ... ... ... ... ... ... ... ... ... ... ... ... ... ... ... ...  [ 198]
H_18        ... ... ... ... ... ... ... ... ... ... ... ... ... ... ... ... ... ... ... ... ... ... ... ... ... ... ... ... ... ... ... ... ...  [ 198]
H_19        ... ... ... ... ... ... ... ... ... ... ... ... ... ... ... ... ... ... ... ... ... ... ... ... ... ... ... ... ... ... ... ... ...  [ 198]
H_20        ... ... ... ... ... ... ... ... ... ... ... ... ... ... ... ... ... ... ... ... ... ... ... ... ... ... ... ... ... ... ... ... ...  [ 198]
H_21        ... ... ... ... ... ... ... ... ... ... ... ... ... ... ... ... ... ... ... ... ... ... ... ... ... ... ... ... ... ... ... ... ...  [ 198]
H_22        ... ... ... ... ... ... ... ... ... ... ... ... ... ... ... ... ... ... ... ... ... ... ... ... ... ... ... ... ... ... ... ... ...  [ 198]
H_23        ... ... ... ... ... ... ... ... ... ... ... ... ... ... ... ... ... ... ... ... ... ... ... ... ... ... ... ... ... ... ... ... ...  [ 198]
H_24        ... ... ... ... ... ... ... ... ... ... ... ... ... ... ... ... ... ... ... ... ... ... ... ... ... ... ... ... ... ... ... ... ...  [ 198]
H_25        ... ... ... ... ... ... ... ... ... ... ... ... ... ... ... ... ... ... ... ... ... ... ... ... ... ... ... ... ... ... ... ... ...  [ 198]
H_26        ... ... ... ... ... ... ... ... ... ... ... ... ... ... ... ... ... ... ... ... ... ... ... ... ... ... ... ... ... ... ... ... ...  [ 198]
H_27        ... ... ... ... ... ... ... ... ... ... ... ... ... ... ... ... ... ... ... ... ... ... ... ... ... ... ... ... ... ... ... ... ...  [ 198]
H_28        ... ... ... ... ... ... ... ... ... ... ... ... ... ... ... ... ... ... ... ... ... ... ... ... ... ... ... ... ... ... ... ... ...  [ 198]
H_29        ... ... ... ... ... ... ... ... ... ... ... ... ... ... ... ... ... ... ... ... ... ... ... ... ... ... ... ... ... ... ... ... ...  [ 198]
H_30        ... ... ... ... ... ... ... ... ... ... ... ... ... ... ... ... ... ... ... ... ... ... ... ... ... ... ... ... ... ... ... ... ...  [ 198]
H_31        ... ... ... ... ... ... ... ... ... ... ... ... ... ... ... ... ... ... ... ... ... ... ... ... ... ... ... ... ... ... ... ... ...  [ 198]
H_32        ... ... ... ... ... ... ... ... ... ... ... ... ... ... ... ... ... ... ... ... ... ... ... ... ... ... ... ... ... ... ... ... ...  [ 198]
H_33        ... ... ... ... ... ... ... ... ... ... ... ... ... ... ... ... ... ... ... ... ... ... ... ... ... ... ... ... ... ... ... ... ...  [ 198]
H_34        ... ... ... ... ... ... ... ... ... ... ... ... ... ... ... ... ... ... ... ... ... ... ... ... ... ... ... ... ... ... ... ... ...  [ 198]
H_35        ... ... ... ... ... ... ... ... ... ... ... ... ... ... ... ... ... ... ... ... ... ... ... ... ... ... ... ... ... ... ... ... ...  [ 198]
H_36        ... ... ... ... ... ... ... ... ... ... ... ... ... ... ... ... ... ... ... ... ... ... ... ... ... ... ... ... ... ... ... ... ...  [ 198]
H_37        ... ... ... ... ... ... ... ... ... ... ... ... ... ... ... ... ... ... ... ... ... ... ... ... ... ... ... ... ... ... ... ... ...  [ 198]
```

```
H_38         ... ... ... ... ... ... ... ... ... ... ... ... ... ... ... ... ... ... ... ... ... ... ... ... ... ... ... ... ... ... ... ... ...  [ 198]
H_39         ... ... ... ... ... ... ... ... ... ... ... ... ... ... ... ... ... ... ... ... ... ... ... ... ... ... ... ... ... ... ... ... ...  [ 198]
H_40         ... ... ... ... ... ... ... ... ... ... ... C.. ... ... ... T.. ... ... ... ... ... C.. ... ... ... ... ... ... ... ... ... ... ...  [ 198]
H_41         ... ... ... ... ... ... ... ... ... ... ... ... ... ... ... ... ... ... ... ... ... ... ... ... ... ... ... ... ... ... ... ... ...  [ 198]
H_42         ... ... ... ... ... ... ... ... ... ... ... ... ... ... ... ... ... ... ... ... ... ... ... ... ... ... ... ... ... ... ... ... ...  [ 198]
H_43         ... ... ... ... ... ... ... ... ... ... ... ... ... ... ... ... ... ... ... ... ... ... ... ... ... ... ... ... ... ... ... ... ...  [ 198]
H_44         ... ... ... ... ... ... ... ... ... ... ... ... ... ... ... ... ... ... ... ... ... ... ... ... ... ... ... ... ... ... ... ... ...  [ 198]
H_45         ... ... ... ... ... ... ... ... ... ... ... ... ... ... ... ... ... ... ... ... ... ... ... ... ... ... ... ... ... ... ... ... ...  [ 198]
H_46         ... ... ... ... ... ... ... ... ... ... ... ... ... ... ... ... ... ... ... ... ... ... ... ... ... ... ... ... ... ... ... ... ...  [ 198]
H_47         ... ... ... ... ... ... ... ... ... ... ... ... ... ... ... ... ... ... ... ... ... ... ... ... ... ... ... ... ... ... ... ... ...  [ 198]
H_48         ... ... ... ... ... ... ... ... ... ... ... ... ... ... ... ... ... ... ... ... ... ... ... ... ... ... ... ... ... ... ... ... ...  [ 198]
H_49         ... ... ... ... ... ... ... ... ... ... ... ... ... ... ... ... ... ... ... ... ... ... ... ... ... ... ... ... ... ... ... ... ...  [ 198]
H_50         ... ... ... ... ... ... ... ... ... ... ... ... ... ... ... ... ... ... ... ... ... ... ... ... ... ... ... ... ... ... ... ... ...  [ 198]
H_51         ... ... ... ... ... ... ... ... ... ... ... ... ... ... ... ... ... ... ... ... ... ... ... ... ... ... ... ... ... ... ... ... ...  [ 198]
H_52         ... ... ... ... ... ... ... ... ... ... ... ... ... ... ... ... ... ... ... ... ... ... ... ... ... ... ... ... ... ... ... ... ...  [ 198]
H_53         ... ... ... ... ... ... ... ... ... ... ... ... ... ... ... ... ... ... ... ... ... ... ... ... ... ... ... ... ... ... ... ... ...  [ 198]
H_54         ... ... ... ... ... ... ... ... ... ... ... ... ... ... ... ... ... ... ... ... ... ... ... ... ... ... ... ... ... ... ... ... ...  [ 198]
H_55         ... ... ... ... ... ... ... ... ... ... ... ... ... ... ... ... ... ... ... ... ... ... ... ... ... ... ... ... ... ... ... ... ...  [ 198]
H_56         ... ... ... ... ... ... ... ... ... ... ... ... ... ... ... ... ... ... ... ... ... ... ... ... ... ... ... ... ... ... ... ... ...  [ 198]
H_57         ... ... ... ... ... ... ... ... ... ... ... ... ... ... ... ... ... ... ... ... ... ... ... ... ... ... ... ... ... ... ... ... ...  [ 198]
H_58         ... ... ... ... ... ... ... ... ... ... ... ... ... ... ... ... ... ... ... ... ... ... ... ... ... ... ... ... ... ... ... ... ...  [ 198]
H_59         ... ... ... ... ... ... ... ... ... ... ... ... ... ... ... ... ... ... ... ... ... ... ... ... ... ... ... ... ... ... ... ... ...  [ 198]
H_60         ... ... ... ... ... ... ... ... ... ... ... ... ... ... ... ... ... ... ... ... ... ... ... ... ... ... ... ... ... ... ... ... ...  [ 198]
H_61         ... ... ... ... ... ... ... ... ... ... ... ... ... ... ... ... ... ... ... ... ... ... ... ... ... ... ... ... ... ... ... ... ...  [ 198]
H_62         ... ... ... ... ... ... ... ... ... ... ... ... ... ... ... ... ... ... ... ... ... ... ... ... ... ... ... ... ... ... ... ... ...  [ 198]
H_63         ... ... ... ... ... ... ... ... ... ... ... ... ... ... ... ... ... ... ... ... ... ... ... ... ... ... ... ... ... ... ... ... ...  [ 198]
H_64         ... ... ... ... ... ... ... ... ... ... ... ... ... ... ... ... ... ... ... ... ... ... ... ... ... ... ... ... ... ... ... ... ...  [ 198]
H_65         ... ... ... ... ... ... ... ... ... ... ... ... ... ... ... ... ... ... ... ... ... ... ... ... ... ... ... ... ... ... ... ... ...  [ 198]
H_66         ... ... ... ... ... ... ... ... ... ... ... ... ... ... ... ... ... ... ... ... ... ... ... ... ... ... ... ... ... ... ... ... ...  [ 198]
H_67         ... ... ... ... ... ... ... ... ... ... ... ... ... ... ... ... ... ... ... ... ... ... ... ... ... ... ... ... ... ... ... ... ...  [ 198]
H_68         ... ... ... ... ... ... ... ... ... ... ... ... ... ... ... ... ... ... ... ... ... ... ... ... ... ... ... ... ... ... ... ... ...  [ 198]
H_69         ... ... ... ... ... ... ... ... ... ... ... ... ... ... ... ... ... ... ... ... ... ... ... ... ... ... ... ... ... ... ... ... ...  [ 198]
H_70         ... ... ... ... ... ... ... ... ... ... ... ... ... ... ... ... ... ... ... ... ... ... ... ... ... ... ... ... ... ... ... ... ...  [ 198]
Pcyn_rbsa    ... .G. ... ... ... ... ... ... ..A ... ... .C. .AT ... ... ... .T. ... .AA ... T.. ... A.. ... ..T ... ... ... G.. ... ... .T. ..A  [ 198]
Pcyn_rbsap   ... .G. ... ... ... ... ... ... ..A ... ... .C. .AT ... ... ... .T. ... .AA ... T.. ... A.. ... ..T ... ... ... G.. ... ... .T. ..A  [ 198]
Pinu_rbsa    ..A .G. ... T.. ... .G. ... .A. ..A A.. ... .C. ..T ... ... ... .T. ... .A. ... T.. ..G AG. ... ..T ... ... ... G.. T.G ... ... ..A  [ 198]
Pfra_rbsa    ... .T. ... A.. ... ... ... ... AGG ... ... .C. ..T ... ... .G. .TA C.G .A. ... TA. ... A.. ... ..T ... ... ... G.. ... ... ... ...  [ 198]

H_1          TCT CTA GCA GAA AGC AAC GAT GCA CCC AGC AAC AGC TAT GAA TCT TTT CCA GAA ATT AGA GAA AAT CTA ACC GCA AGT GAG GAA TCC CTA ACA TCA TGT  [ 297]
H_2          ... ... ... ... ... ... ... ... ... ... ... ... ... ... ... ... ... ... ... ... ... ... ... ... ... ... ... ... ... ... ... ... ...  [ 297]
H_3          ... ... ... ... ... ... ... ... ... ... ... ... ... ... ... ... ... ... ... ... ... ... ... ... ... ... ... ... ... ... ... ... ...  [ 297]
H_4          ... ... ... ... ... ... ... ... ... ... ... ... ... ... ... ... ... ... ... ... ... ... ... ... ... ... ... ... ... ... ... ... ...  [ 297]
H_5          ... ... ... ... ... ... ... ... ... ... ... ... ... ... ... ... ... ... ... ... ... ... ... ... ... ... ... ... ... ... ... --- ---  [ 297]
H_6          ... ... ... ... ... ... ... ... ... ... ... ... ... ... ... ... ... ... ... ... ... ... ... ... ... ... ... ... ... ... ... ... ...  [ 297]
H_7          ... ... ... ... ... ... ... ... ... ... ... ... ... ... ... ... ... ... ... ... ... ... ... ... ... ... ... ... ... ... ... ... ...  [ 297]
H_8          ... ... ... ... ... ... ... ... ... ... ... ... ... ... ... ... ... ... ... ... ... ... ... ... ... ... ... ... ... ... ... ... ...  [ 297]
H_9          ... ... ... ... ... ... ... ... ... ... ... ... ... ... ... ... ... ... ... ... ... ... ... ... ... ... ... ... ... ... ... ... ...  [ 297]
H_10         ... ... ... ... ... ... ... ... ... ... ... ... ... ... ... ... ... ... ... ... ... ... ... ... ... ... ... ... ... ... ... ... ...  [ 297]
H_11         ... ... ... ... ... ... ... ... ... ... ... ... ... ... ... ... ... ... ... ... ... ... ... ... ... ... ... ... ... ... ... ... ...  [ 297]
H_12         ... ... ... ... ... ... ... ... ... ... ... ... ... ... ... ... ... ... ... ... ... ... ... ... ... ... ... ... ... ... ... ... ...  [ 297]
H_13         ... ... ... ... ... ... ... ... ... ... ... ... ... ... ... ... ... ... ... ... ... ... ... ... ... ... ... ... ... ... ... ... ...  [ 297]
H_14         ... ... ... ... ... ... ... ... ... ... ... ... ... ... ... ... ... ... ... ... ... ... ... ... ... ... ... ... ... ... ... ... ...  [ 297]
H_15         ... ... ... ... ... ... ... ... ... ... ... ... ... ... ... ... ... ... ... ... ... ... ... ... ... ... ... ... ... ... ... ... ...  [ 297]
H_16         ... ... ... ... ... ... ... ... ... ... ... ... ... ... ... ... ... ... ... ... ... ... ... ... ... ... ... ... ... ... ... ... ...  [ 297]
H_17         ... ... ... ... ... ... ... ... ... ... ... ... ... ... ... ... ... ... ... ... ... ... ... ... ... ... ... ... ... ... ... ... ...  [ 297]
H_18         ... ... ... ... ... ... ... ... ... ... ... ... ... ... ... ... ... ... ... ... ... ... ... ... ... ... ... ... ... ... ... ... ...  [ 297]
```

```
H_19          ... ... ... ... ... ... ... ... ... ... ... ... ... ... ... ... ... ... ... ... ... ... ... ... ... ... ... ... ... ... ... ... ...  [ 297]
H_20          ... ... ... ... ... ... ... ... ... ... ... ... ... ... ... ... ... ... ... ... ... ... ... ... ... ... ... ... ... ... ... ... ...  [ 297]
H_21          ... ... ... ... ... ... ... ... ... ... ... ... ... ... ... ... ... ... ... ... ... ... ... ... ... ... ... ... ... ... ... ... ...  [ 297]
H_22          ... ... ... ... ... ... ... ... ... ... ... ... ... ... ... ... ... ... ... ... ... ... ... ... ... ... ... ... ... ... ... ... ...  [ 297]
H_23          ... ... ... ... ... ... ... ... ... ... ... ... ... ... ... ... ... ... ... ... ... ... ... ... ... ... ... ... ... ... ... ... ...  [ 297]
H_24          ... ... ... ... ... ... ... ... ... ... ... ... ... ... ... ... ... ... ... ... ... ... ... ... ... ... ... ... ... ... ... ... ...  [ 297]
H_25          ... ... ... ... ... ... ... ... ... ... ... ... ... ... ... ... ... ... ... ... ... ... ... ... ... ... ... ... ... ... ... ... ...  [ 297]
H_26          ... ... ... ... ... ... ... ... ... ... ... ... ... ... ... ... ... ... ... ... ... ... ... ... ... ... ... ... ... ... ... ... ...  [ 297]
H_27          ... ... ... ... ... ... ... ... ... ... ... ... ... ... ... ... ... ... ... ... ... ... ... ... ... ... ... ... ... ... ... ... ...  [ 297]
H_28          ... ... ... ... ... ... ... ... ... ... ... ... ... ... ... ... ... ... ... ... ... ... ... ... ... ... ... ... ... ... ... ... ...  [ 297]
H_29          ... ... ... ... ... ... ... ... ... ... ... ... ... ... ... ... ... ... ... ... ... ... ... ... ... ... ... ... ... ... ... ... ...  [ 297]
H_30          ... ... ... ... ... ... ... ... ... ... ... ... ... ... ... ... ... ... ... ... ... ... ... ... ... ... ... ... ... ... ... ... ...  [ 297]
H_31          ... ... ... ... ... ... ... ... ... ... ... ... ... ... ... ... ... ... ... ... ... ... ... ... ... ... ... ... ... ... ... ... ...  [ 297]
H_32          ... ... ... ... ... ... ... ... ... ... ... ... ... ... ... ... ... ... ... ... ... ... ... ... ... ... ... ... ... ... ... --- ---  [ 297]
H_33          ... ... ... ... ... ... ... ... ... ... ... ... ... ... ... ... ... ... ... ... ... ... ... ... ... ... ... ... ... ... ... ... ...  [ 297]
H_34          ... ... ... ... ... ... ... ... ... ... ... ... ... ... ... ... ... ... ... ... ... ... ... ... ... ... ... ... ... ... ... ... ...  [ 297]
H_35          ... ... ... ... ... ... ... ... ... ... ... ... ... ... ... ... ... ... ... ... ... ... ... ... ... ... ... ... ... ... ... ... ...  [ 297]
H_36          ... ... ... ... ... ... ... ... ... ... ... ... ... ... ... ... ... ... ... ... ... ... ... ... ... ... ... ... ... ... ... ... ...  [ 297]
H_37          ... ... ... ... ... ... ... ... ... ... ... ... ... ... ... ... ... ... ... ... ... ... ... ... ... ... ... ... ... ... ... ... ...  [ 297]
H_38          ... ... ... ... ... ... ... ... ... ... ... ... ... ... ... ... ... ... ... ... ... ... ... ... ... ... ... ... ... ... ... --- ---  [ 297]
H_39          ... ... ... ... ... ... ... ... ... ... ... ... ... ... ... ... ... ... ... ... ... ... ... ... ... ... ... ... ... ... ... ... ...  [ 297]
H_40          ... ... ... ... ... ... ... ... ... ... ... ... ... ... ... ... ... ... ... ... ... ... ... ... ... ... ... ... ... ... ... ... ...  [ 297]
H_41          ... ... ... ... ... ... ... ... ... ... ... ... ... ... ... ... ... ... ... ... ... ... ... ... ... ... ... ... ... ... ... ... ...  [ 297]
H_42          ... ... ... ... ... ... ... ... ... ... ... ... ... ... ... ... ... ... ... ... ... ... ... ... ... ... ... ... ... ... ... ... ...  [ 297]
H_43          ... ... ... ... ... ... ... ... ... ... ... ... ... ... ... ... ... ... ... ... ... ... ... ... ... ... ... ... ... ... ... ... ...  [ 297]
H_44          ... ... ... ... ... ... ... ... ... ... ... ... ... ... ... ... ... ... ... ... ... ... ... ... ... ... ... ... ... ... ... ... ...  [ 297]
H_45          ... ... ... ... ... ... ... ... ... ... ... ... ... ... ... ... ... ... ... ... ... ... ... ... ... ... ... ... ... ... ... ... ...  [ 297]
H_46          ... ... ... ... ... ... ... ... ... ... ... ... ... ... ... ... ... ... ... ... ... ... ... ... ... ... ... ... ... ... ... ... ...  [ 297]
H_47          ... ... ... ... ... ... ... ... ... ... ... ... ... ... ... ... ... ... ... ... ... ... ... ... ... ... ... ... ... ... ... --- ---  [ 297]
H_48          ... ... ... ... ... ... ... ... ... ... ... ... ... ... ... ... ... ... ... ... ... ... ... ... ... ... ... ... ... ... ... ... ...  [ 297]
H_49          ... ... ... ... ... ... ... ... ... ... ... ... ... ... ... ... ... ... ... ... ... ... ... ... ... ... ... ... ... ... ... ... ...  [ 297]
H_50          ... ... ... ... ... ... ... ... ... ... ... ... ... ... ... ... ... ... ... ... ... ... ... ... ... ... ... ... ... ... ... ... ...  [ 297]
H_51          ... ... ... ... ... ... ... ... ... ... ... ... ... ... ... ... ... ... ... ... ... ... ... ... ... ... ... ... ... ... ... ... ...  [ 297]
H_52          ... ... ... ... ... ... ... ... ... ... ... ... ... ... ... ... ... ... ... ... ... ... ... ... ... ... ... ... ... ... ... ... ...  [ 297]
H_53          ... ... ... ... ... ... ... ... ... ... ... ... ... ... ... ... ... ... ... ... ... ... ... ... ... ... ... ... ... ... ... ... ...  [ 297]
H_54          ... ... ... ... ... ... ... ... ... ... ... ... ... ... ... ... ... ... ... ... ... ... ... ... ... ... ... ... ... ... ... ... ...  [ 297]
H_55          ... ... ... ... ... ... ... ... ... ... ... ... ... ... ... ... ... ... ... ... ... ... ... ... ... ... ... ... ... ... ... ... ...  [ 297]
H_56          ... ... ... ... ... ... ... ... ... ... ... ... ... ... ... ... ... ... ... ... ... ... ... ... ... ... ... ... ... ... ... ... ...  [ 297]
H_57          ... ... ... ... ... ... ... ... ... ... ... ... ... ... ... ... ... ... ... ... ... ... ... ... ... ... ... ... ... ... ... ... ...  [ 297]
H_58          ... ... ... ... ... ... ... ... ... ... ... ... ... ... ... ... ... ... ... ... ... ... ... ... ... ... ... ... ... ... ... ... ...  [ 297]
H_59          ... ... ... ... ... ... ... ... ... ... ... ... ... ... ... ... ... ... ... ... ... ... ... ... ... ... ... ... ... ... ... ... ...  [ 297]
H_60          ... ... ... ... ... ... ... ... ... ... ... ... ... ... ... ... ... ... ... ... ... ... ... ... ... ... ... ... ... ... ... ... ...  [ 297]
H_61          ... ... ... ... ... ... ... ... ... ... ... ... ... ... ... ... ... ... ... ... ... ... ... ... ... ... ... ... ... ... ... ... ...  [ 297]
H_62          ... ... ... ... ... ... ... ... ... ... ... ... ... ... ... ... ... ... ... ... ... ... ... ... ... ... ... ... ... ... ... ... ...  [ 297]
H_63          ... ... ... ... ... ... ... ... ... ... ... ... ... ... ... ... ... ... ... ... ... ... ... ... ... ... ... ... ... ... ... ... ...  [ 297]
H_64          ... ... ... ... ... ... ... ... ... ... ... ... ... ... ... ... ... ... ... ... ... ... ... ... ... ... ... ... ... ... ... ... ...  [ 297]
H_65          ... ... ... ... ... ... ... ... ... ... ... ... ... ... ... ... ... ... ... ... ... ... ... ... ... ... ... ... ... ... ... ... ...  [ 297]
H_66          ... ... ... ... ... ... ... ... ... ... ... ... ... ... ... ... ... ... ... ... ... ... ... ... ... ... ... ... ... ... ... ... ...  [ 297]
H_67          ... ... ... ... ... ... ... ... ... ... ... ... ... ... ... ... ... ... ... ... ... ... ... ... ... ... ... ... ... ... ... ... ...  [ 297]
H_68          ... ... ... ... ... ... ... ... ... ... ... ... ... ... ... ... ... ... ... ... ... ... ... ... ... ... ... ... ... ... ... ... ...  [ 297]
H_69          ... ... ... ... ... ... ... ... ... ... ... ... ... ... ... ... ... ... ... ... ... ... ... ... ... ... ... ... ... ... ... ... ...  [ 297]
H_70          ... ... ... ... ... ... ... ... ... ... ... ... ... ... ... ... ... ... ... ... ... ... ... ... ... ... ... ... ... ... ... ... ...  [ 297]
Pcyn_rbsa     ... ... A.. .C. ..T .GT ..G --- --- --- --- --- --- --- --- --- --- --- --- --- --- TC. ... G.A ... G.. AGT ... ..T ... G.. G.. A..  [ 297]
Pcyn_rbsap    ... ... A.. .C. ..T .GT ..G --- --- --- --- --- --- --- --- --- --- --- --- --- --- TC. ... G.A ... G.. AGT ... ..T ... G.. G.. A..  [ 297]
Pinu_rbsa     ... T.. T.. .C. C.T .G. --- --- --- --- --- --- --- --- --- --- --- --- --- --- --- --- --- --- --- --- --- --- --- --- --- --- ---  [ 297]
Pfra_rbsa     ... T.. ... .C. ..T .GT --- --- --- --- --- --- --- --- --- --- --- --- --- --- --- --- --- --- --- --- --- --- --- --- --- --- ---  [ 297]

H_1           GAG GAA TCC CTA ACA GGA AGT AAT GAA TCC CTA ACA GGA AGT AAT GAA TCC CTA ACA GGA AGT AAT --- --- --- --- --- --- --- --- --- --- ---  [ 396]
H_2           ... ... ... ... ... ... ... ... ... ... ... ... ... ... ... ... ... ... ... ... ... ... --- --- --- --- --- --- --- --- --- --- ---  [ 396]
H_3           ... ... ... ... ... ... ... ... ... ... ... ... ... ... ... ... ... ... ... ... ... ... --- --- --- --- --- --- --- --- --- --- ---  [ 396]
```

```
H_4      ... ... ... ... ... ... ... ... ... ... ... ... ... ... ... ... ... ... ... ... ... ... --- --- --- --- --- --- --- --- --- --- --- [ 396]
H_5      --- --- --- --- --- ... ... ... ... ... ... ... ... ... ... ... ... ... ... ... ... ... --- --- --- --- --- --- --- --- --- --- --- [ 396]
H_6      ... ... ... ... ... ... ... ... ... ... ... ... ... ... ... ... ... ... ... ... ... ... --- --- --- --- --- --- --- --- --- --- --- [ 396]
H_7      ... ... ... ... ... ... ... ... ... ... ... ... ... ... ... ... ... ... ... ... ... ... --- --- --- --- --- --- --- --- --- --- --- [ 396]
H_8      ... ... ... ... ... ... ... ... ... ... ... ... ... ... ... ... ... ... ... ... ... ... --- --- --- --- --- --- --- --- --- --- --- [ 396]
H_9      ... ... ... ... ... ... ... ... ... ... ... ... ... ... ... ... ... ... ... ... ... ... --- --- --- --- --- --- --- --- --- --- --- [ 396]
H_10     ... ... ... ... ... ... ... ... ... ... ... ... ... ... ... ... ... ... ... .A. ... ... --- --- --- --- --- --- --- --- --- --- --- [ 396]
H_11     ... ... ... ... ... ... ... ... ... ... ... ... ... ... ... ... ... ... ... ... ... ... --- --- --- --- --- --- --- --- --- --- --- [ 396]
H_12     ... ... ... ... ... ... ... ... ... ... ... ... ... ... ... ... ... ... ... ... ... ... --- --- --- --- --- --- --- --- --- --- --- [ 396]
H_13     ... ... ... ... ... ... ... ... ... ... ... ... ... ... ... ... ... ... ... ... ... ... --- --- --- --- --- --- --- --- --- --- --- [ 396]
H_14     ... ... ... ... ... ... ... ... ... ... ... ... ... ... ... ... ... ... ... ... ... ... --- --- --- --- --- --- --- --- --- --- --- [ 396]
H_15     ... ... ... ... ... ... ... ... ... ... ... ... ... ... ... ... ... ... ... ... ... ... --- --- --- --- --- --- --- --- --- --- --- [ 396]
H_16     ... ... ... ... ... ... ... ... ... ... ... ... --- --- --- --- --- --- --- --- --- --- --- --- --- --- --- --- --- --- --- --- --- [ 396]
H_17     ... ... ... ... ... ... ... ... ... ... ... ... ... ... ... ... ... ... ... ... ... ... --- --- --- --- --- --- --- --- --- --- --- [ 396]
H_18     ... ... ... ... ... ... ... ... ... ... ... ... ... ... ... ... ... ... ... ... ... ... --- --- --- --- --- --- --- --- --- --- --- [ 396]
H_19     ... ... ... ... ... ... ... ... ... ... ... ... ... ... ... ... ... ... ... ... ... ... --- --- --- --- --- --- --- --- --- --- --- [ 396]
H_20     ... ... ... ... ... ... ... ... ... ... ... ... ... ... ... ... ... ... ... ... ... ... --- --- --- --- --- --- --- --- --- --- --- [ 396]
H_21     ... ... ... ... ... ... ... ... ... ... ... ... ... ... ... ... ... ... ... ... ... ... --- --- --- --- --- --- --- --- --- --- --- [ 396]
H_22     ... ... .T. ... ... ... ... ... ... ... ... ... ... ... ... ... ... ... ... ... ... ... --- --- --- --- --- --- --- --- --- --- --- [ 396]
H_23     ... ... ... ... ... ... ... ... ... ... ... ... ... ... ... ... ... ... ... ... ... ... --- --- --- --- --- --- --- --- --- --- --- [ 396]
H_24     ... ... ... ... ... ... ... ... ... ... ... ... ... ... ... ... ... ... ... ... ... ... --- --- --- --- --- --- --- --- --- --- --- [ 396]
H_25     ... ... ... ... ... ... ... ... ... ... ... ... ... ... ... ... ... ... ... ... ... ... --- --- --- --- --- --- --- --- --- --- --- [ 396]
H_26     ... ... ... ... ... ... ... ... ... ... ... ... ... ... ... ... ... ... ... ... ... ... --- --- --- --- --- --- --- --- --- --- --- [ 396]
H_27     ... ... ... ... ... ... ... ... ... ... ... ... ... ... ... ... ... ... ... ... ... ... --- --- --- --- --- --- --- --- --- --- --- [ 396]
H_28     ... ... ... ... ... ... ... ... ... ... ... ... ... ... ... ... ... ... ... ... ... ... --- --- --- --- --- --- --- --- --- --- --- [ 396]
H_29     ... ... ... ... ... ... ... ... ... ... ... ... ... ... ... ... ... ... ... ... ... ... --- --- --- --- --- --- --- --- --- --- --- [ 396]
H_30     ... ... ... ... ... ... ... ... ... ... ... ... ... ... ... ... ... ... ... ... ... ... --- --- --- --- --- --- --- --- --- --- --- [ 396]
H_31     ... ... ... ... ... ... ... ..C ... ... ... ... ... ... ... ... ... ... ... ... ... ... --- --- --- --- --- --- --- --- --- --- --- [ 396]
H_32     --- --- --- --- --- ... ... ... ... ... ... ... ... ... ... ... ... ... ... .A. ... ... --- --- --- --- --- --- --- --- --- --- --- [ 396]
H_33     ... ... ... ... ... ... ... ... ... ... ... ... ... ... ... ... ... ... ... ... ... ... --- --- --- --- --- --- --- --- --- --- --- [ 396]
H_34     ... ... ... ... ... ... ... ... ... ... ... ... ... ... ... ... ... ... ... ... ... ... --- --- --- --- --- --- --- --- --- --- --- [ 396]
H_35     ... ... ... ... ... ... ... ... ... ... ... ... ... ... ... ... ... ... ... ... ... ... --- --- --- --- --- --- --- --- --- --- --- [ 396]
H_36     ... ... ... ... ... ... ... ..C ... ... ... ... ... ... ... ... ... ... ... ... ... ... --- --- --- --- --- --- --- --- --- --- --- [ 396]
H_37     ... ... ... ... ... ... ... ... ... ... ... ... ... ... ... ... ... ... ... ... ... ... --- --- --- --- --- --- --- --- --- --- --- [ 396]
H_38     --- --- --- --- --- ... ... ... ... ... ... ... ... ... ... ... ... ... ... .A. ... ... --- --- --- --- --- --- --- --- --- --- --- [ 396]
H_39     ... ... ... ... ... ... ... ... --- --- --- --- --- --- --- --- --- --- --- --- --- --- --- --- --- --- --- --- --- --- --- --- --- [ 396]
H_40     ... ... ... ... ... ... ... ... ... ... ... ... ... ... ... ... ... ... ... ... ... ... --- --- --- --- --- --- --- --- --- --- --- [ 396]
H_41     ... ... ... ... ... ... ... ... ... ... ... ... ... ... ... ... ... ... ... ... ... ... --- --- --- --- --- --- --- --- --- --- --- [ 396]
H_42     ... ... ... ... ... ... ... ... ... ... ... ... ... ... ... ... ... ... ... ... ... ... --- --- --- --- --- --- --- --- --- --- --- [ 396]
H_43     ... ... ... ... ... ... ... ... ... ... ... ... ... ... ... ... ... ... ... ... ... ... --- --- --- --- --- --- --- --- --- --- --- [ 396]
H_44     ... ... ... ... ... ... ... ... ... ... ... ... ... ... ... ... ... ... ... ... ... ... --- --- --- --- --- --- --- --- --- --- --- [ 396]
H_45     ... ... ... ... ... ... ... ..C ... ... ... ... ... ... ... ... ... ... ... ... ... ... --- --- --- --- --- --- --- --- --- --- --- [ 396]
H_46     ... ... ... ... ... ... ... ... ... ... ... ... ... ... ... ... ... ... ... ... ... ... --- --- --- --- --- --- --- --- --- --- --- [ 396]
H_47     --- --- --- --- --- ... ... ... ... ... ... ... ... ... ... ... ... ... ... .A. ... ... --- --- --- --- --- --- --- --- --- --- --- [ 396]
H_48     ... ... ... ... ... ... ... ... ... ... ... ... ... ... ... ... ... ... ... ... ... ... --- --- --- --- --- --- --- --- --- --- --- [ 396]
H_49     ... ... ... ... ... ... ... ... ... ... ... ... ... ... ... ... ... ... ... ... ... ... --- --- --- --- --- --- --- --- --- --- --- [ 396]
H_50     ... ... ... ... ... ... ... ... ... ... ... ... ... ... ... ... ... ... ... ... ... ... --- --- --- --- --- --- --- --- --- --- --- [ 396]
H_51     ... ... ... ... ... ... ... ... ... ... ... ... ... ... ... ... ... ... ... ... ... ... --- --- --- --- --- --- --- --- --- --- --- [ 396]
H_52     ... ... ... ... ... ... ... ... ... ... ... ... ... ... ... ... ... ... ... ... ... ... --- --- --- --- --- --- --- --- --- --- --- [ 396]
H_53     ... ... ... ... ... ... ... ... ... ... ... ... ... ... ... ... ... ... ... ... ... ... --- --- --- --- --- --- --- --- --- --- --- [ 396]
H_54     ... ... ... ... ... ... ... ... ... ... ... ... ... ... ... ... ... ... ... ... ... ... --- --- --- --- --- --- --- --- --- --- --- [ 396]
H_55     ... ... ... ... ... ... ... ... ... ... ... ... ... ... ... ... ... ... ... ... ... ... --- --- --- --- --- --- --- --- --- --- --- [ 396]
H_56     ... ... ... ... ... ... ... ... ... ... ... ... ... ... ... ... ... ... ... ... ... ... --- --- --- --- --- --- --- --- --- --- --- [ 396]
H_57     ... ... ... ... ... ... ... ... ... ... ... ... ... ... ... ... ... ... ... ... ... ... --- --- --- --- --- --- --- --- --- --- --- [ 396]
H_58     ... ... ... ... ... ... ... ... --- --- --- --- --- --- --- --- --- --- --- --- --- --- --- --- --- --- --- --- --- --- --- --- --- [ 396]
H_59     ... ... ... ... ... ... ... ... ... ... ... ... ... ... ... ... ... ... ... ... ... ... --- --- --- --- --- --- --- --- --- --- --- [ 396]
```

```
H_60         ... ... ... ... ... ... ... ... ... ... ... ... ... ... ... ... ... ... ... ... ... ... --- --- --- --- --- --- --- --- --- --- ---  [ 396]
H_61         ... ... ... ... ... ... ... ... ... ... ... ... ... ... ... ... ... ... ... ... ... ... --- --- --- --- --- --- --- --- --- --- ---  [ 396]
H_62         ... ... ... ... ... ... ... ... ... ... ... ... ... ... ... ... ... ... ... ... ... ... --- --- --- --- --- --- --- --- --- --- ---  [ 396]
H_63         ... ... ... ... ... ... ... ... ... ... ... ... ... ... ... ... ... ... ... ... ... ... --- --- --- --- --- --- --- --- --- --- ---  [ 396]
H_64         ... ... ... ... ... ... ... ... --- --- --- --- --- --- --- --- --- --- --- --- --- --- --- --- --- --- --- --- --- --- --- --- ---  [ 396]
H_65         ... ... ... ... ... ... ... ... ... ... ... ... ... ... ... ... ... ... ... ... ... ... --- --- --- --- --- --- --- --- --- --- ---  [ 396]
H_66         ... ... ... ... ... ... ... ... ... ... ... ... ... ... ... ... ... ... ... ... ... ... --- --- --- --- --- --- --- --- --- --- ---  [ 396]
H_67         ... ... ... ... ... ... ... ..C ... ... ... ... ... ... ... ... ... ... ... ... ... ... --- --- --- --- --- --- --- --- --- --- ---  [ 396]
H_68         ... ... ... ... ... ... ... ... ... ... ... ... ... ... ... ... ... ... ... ... ... ... --- --- --- --- --- --- --- --- --- --- ---  [ 396]
H_69         ... ... ... ... ... ... ... ... ... ... ... ... ... ... ... ... ... ... ... ... ... ... --- --- --- --- --- --- --- --- --- --- ---  [ 396]
H_70         ... ... ... ... ... ... ... ... ... ... ... ... ... ... ... ... ... ... ... ... ... ... --- --- --- --- --- --- --- --- --- --- ---  [ 396]
Pcyn_rbsa    AGT --- --- --- --- --- --- --- --- --- --- --- --- --- --- --- --- --- --- --- --- --- --- --- --- --- --- --- --- --- --- --- ---  [ 396]
Pcyn_rbsap   AGT --- --- --- --- --- --- --- --- --- --- --- --- --- --- --- --- --- --- --- --- --- --- --- --- --- --- --- --- --- --- --- ---  [ 396]
Pinu_rbsa    --- --- --- --- --- --- --- --- --- --- --- --- --- --- --- --- --- --- --- --- --- --- --- --- --- --- --- --- --- --- --- --- ---  [ 396]
Pfra_rbsa    --- --- --- --- --- --- --- --- --- --- --- --- --- --- --- --- --- --- --- --- --- --- --- --- --- --- --- --- --- --- --- --- ---  [ 396]

H_1          --- --- --- --- --- --- --- --- --- --- GAA TCC CTA ACA GGA AGT AAT GAA TCC CTA ACA GGA AGT AAT GAA TCC CTA ACA GAA AGT AGA GAA TCT  [ 495]
H_2          --- --- --- --- --- --- --- --- --- --- ... ... ... ... ... ... ... ... ... ... ... ... ... ... ... ... ... ... ... ... ... ... ...  [ 495]
H_3          --- --- --- --- --- --- --- --- --- --- ... ... ... ... ... ... ... ... ... ... ... ... ... ... ... ... ... ... ... ... ... ... ...  [ 495]
H_4          --- --- --- --- --- --- --- --- --- --- ... ... ... ... ... ... ... ... ... ... ... ... ... ... ... ... ... ... ... ... ... ... ...  [ 495]
H_5          --- --- --- GAA TCC CTA ACA GGA AGT AAT ... ... ... ... ... ... ... ... ... ... ... ... ... ... ... ... ... ... ... ... ... ... ...  [ 495]
H_6          --- --- --- --- --- --- --- --- --- --- ... ... ... ... ... ... ... ... ... ... ... ... ... ... ... ... ... ... ... ... ... ... ...  [ 495]
H_7          --- --- --- --- --- --- --- --- --- --- ... ... ... ... ... ... ... ... ... ... ... ... ... ... ... ... ... ... ... ... ... ... ...  [ 495]
H_8          --- --- --- --- --- --- --- --- --- --- ... ... ... ... ... ... ... ... ... ... ... ... ... ... ... ... ... ... ... ... ... ... ...  [ 495]
H_9          --- --- --- --- --- --- --- --- --- --- ... ... ... ... ... ... ... ... ... ... ... ... ... ... ... ... ... ... ... ... ... ... ...  [ 495]
H_10         --- --- --- --- --- --- --- --- --- --- ... ... ... ... ... ... ... ... ... ... ... ... ... ... ... ... ... ... ... ... ... ... ...  [ 495]
H_11         --- --- --- --- --- --- --- --- --- --- ... ... ... ... ... ... ... ... ... ... ... ... ... ... ... ... ... ... ... ... ... ... ...  [ 495]
H_12         --- --- --- --- --- --- --- --- --- --- ... ... ... ... ... ... ... ... ... ... ... .A. ... .GA ... ... ... ... ... ... ... ... ...  [ 495]
H_13         --- --- --- --- --- --- --- --- --- --- ... ... ... ... ... ... ... ... ... ... ... ... ... ... ... ... ... ... ... ... ... ... ...  [ 495]
H_14         --- --- --- --- --- --- --- --- --- --- ... ... ... ... ... ... ... ... ... ... ... ... ... ... ... ... ... ... ... ... ... ... ...  [ 495]
H_15         --- --- --- --- --- --- --- --- --- --- ... ... ... ... ... ... ... ... ... ... ... ... ... ... ... ... ... ... ... ... ... ... ...  [ 495]
H_16         GAA AGT AGA GAA TCC CTA ACA GGA AGT AAT ... ... ... ... .A. ... .GA ... ... ... ... .A. ... .GA ... ... ... ... ... ... ... ... ...  [ 495]
H_17         --- --- --- --- --- --- --- --- --- --- ... ... ... ... ... ... ... ... ... ... ... ... ... ... ... ... ... ... ... ... ... ... ...  [ 495]
H_18         --- --- --- --- --- --- --- --- --- --- ... ... ... ... ... ... ... ... ... ... ... ... ... ... ... ... ... ... ... ... ... ... ...  [ 495]
H_19         --- --- --- --- --- --- --- --- --- --- ... ... ... ... ... ... ... ... ... ... ... ... ... ... ... ... ... ... ... ... ... ... ...  [ 495]
H_20         --- --- --- --- --- --- --- --- --- --- ... ... ... ... ... ... ... ... ... ... ... ... ... ... ... ... ... ... ... ... ... ... ...  [ 495]
H_21         --- --- --- --- --- --- --- --- --- --- ... ... ... ... ... ... ... ... ... ... ... ... ... ... ... ... ... ... ... ... ... ... ...  [ 495]
H_22         --- --- --- --- --- --- --- --- --- --- ... ... ... ... ... ... ... ... ... ... ... ... ... ... ... ... ... ... ... ... ... ... ...  [ 495]
H_23         --- --- --- --- --- --- --- --- --- --- ... ... ... ... ... ... ... ... ... ... ... ... ... ... ... ... ... ... ... ... ... ... ...  [ 495]
H_24         --- --- --- --- --- --- --- --- --- --- ... ... ... ... ... ... ... ... ... ... ... ... ... ... ... ... ... ... ... ... ... ... ...  [ 495]
H_25         --- --- --- --- --- --- --- --- --- --- ... ... ... ... ... ... ... ... ... ... ... ... ... ... ... ... ... ... ... ... ... ... ...  [ 495]
H_26         --- --- --- --- --- --- --- --- --- --- ... ... ... ... ... ... ... ... ... ... ... ... ... ... ... ... ... ... ... ... ... ... ...  [ 495]
H_27         --- --- --- --- --- --- --- --- --- --- ... ... ... ... ... ... ... ... ... ... ... ... ... ... ... ... ... ... ... ... ... ... ...  [ 495]
H_28         --- --- --- --- --- --- --- --- --- --- ... ... ... ... ... ... ... ... ... ... ... ... ... ... ... ... ... ... ... ... ... ... ...  [ 495]
H_29         --- --- --- --- --- --- --- --- --- --- ... ... ... ... ... ... ... ... ... ... ... ... ... ... ... ... ... ... ... ... ... ... ...  [ 495]
H_30         --- --- --- --- --- --- --- --- --- --- ... ... ... ... ... ... ... ... ... ... ... ... ... ... ... ... ... ... ... ... ... ... ...  [ 495]
H_31         --- --- --- --- --- --- --- --- --- --- ... ... ... ... ... ... ... ... ... ... ... ... ... ... ... ... ... ... ... ... ... ... ...  [ 495]
H_32         --- --- --- GAA TCC CTA ACA GGA AGT AAT ... ... ... ... ... ... ... ... ... ... ... ... ... ... ... ... ... ... ... ... ... ... ...  [ 495]
H_33         --- --- --- --- --- --- --- --- --- --- --- --- --- --- --- --- --- --- --- --- --- --- --- --- ... ... ... ... ... ... ... ... ...  [ 495]
H_34         --- --- --- --- --- --- --- --- --- --- ... ... ... ... ... ... ... ... ... ... ... ... ... ... ... ... ... ... ... ... ... ... ...  [ 495]
H_35         --- --- --- --- --- --- --- --- --- --- --- --- --- --- --- --- --- ... ... ... ... ... ... ... ... ... ... ... ... ... ... ... ...  [ 495]
H_36         --- --- --- --- --- --- --- --- --- --- --- --- --- --- --- --- --- ... ... ... ... ... ... ... ... ... ... ... ... ... ... ... ...  [ 495]
H_37         --- --- --- --- --- --- --- --- --- --- --- --- --- --- --- --- --- ... ... ... ... ... ... ... ... ... ... ... ... ... ... ... ...  [ 495]
H_38         --- --- --- GAA TCC CTA ACA GGA AGT AAT ... ... ... ... ... ... ... ... ... ... ... ... ... ... ... ... ... ... ... ... ... ... ...  [ 495]
H_39         --- --- --- --- --- --- --- --- --- --- --- --- --- --- --- --- --- --- --- --- --- --- --- --- ... ... ... ... ... ... ... ... ...  [ 495]
H_40         --- --- --- --- --- --- --- --- --- --- --- --- --- --- --- --- --- ... ... ... ... ... ... ... ... ... ... ... ... ... ... ... ...  [ 495]
H_41         --- --- --- GAA TCC CTA ACA GGA AGT AAT ... ... ... ... ... ... ... ... ... ... ... ... ... ... ... ... ... ... ... ... ... ... ...  [ 495]
H_42         --- --- --- --- --- --- --- --- --- --- --- --- --- --- --- --- --- --- --- --- --- --- --- --- ... ... ... ... ... ... ... ... ...  [ 495]
H_43         --- --- --- --- --- --- --- --- --- --- --- --- --- --- --- --- --- ... ... ... ... ... ... ... ... ... ... ... ... ... ... ... ...  [ 495]
H_44         --- --- --- --- --- --- --- --- --- --- --- --- --- --- --- --- --- ... ... ... ... ... ... ... ... ... ... ... ... ... ... ... ...  [ 495]
```

```
H_45          --- --- --- --- --- --- --- --- --- --- --- --- --- --- --- --- --- ... ... ... ... ... ... ... ... ... ... ... ... ... ... ... ...  [ 495]
H_46          --- --- --- --- --- --- --- --- --- --- --- --- --- --- --- --- --- --- --- --- --- --- --- --- ... ... ... ... ... ... ... ... ...  [ 495]
H_47          --- --- --- GAA TCC CTA ACA GGA AGT AAT ... ... ... ... ... ... ... ... ... ... ... ... ... ... ... ... ... ... ... ... ... ... ...  [ 495]
H_48          --- --- --- --- --- --- --- --- --- --- --- --- --- --- --- --- --- --- --- --- --- --- --- --- ... ... ... ... ... ... ... ... ...  [ 495]
H_49          --- --- --- --- --- --- --- --- --- --- --- --- --- --- --- --- --- ... ... ... ... ... ... ... ... ... ... ... ... ... ... ... ...  [ 495]
H_50          --- --- --- --- --- --- --- --- --- --- --- --- --- --- --- --- --- ... ... ... ... ... ... ... ... ... ... ... ... ... ... ... ...  [ 495]
H_51          --- --- --- --- --- --- --- --- --- --- --- --- --- --- --- --- --- --- --- --- --- --- --- --- ... ... ... ... ... ... ... ... ...  [ 495]
H_52          --- --- --- --- --- --- --- --- --- --- --- --- --- --- --- --- --- ... ... ... ... ... ... ... ... ... ... ... ... ... ... ... ...  [ 495]
H_53          --- --- --- GAA TCC CTA ACA GGA AGT AAT ... ... ... ... ... ... ... ... ... ... ... ... ... ... ... ... ... ... ... ... ... ... ...  [ 495]
H_54          --- --- --- --- --- --- --- --- --- --- --- --- --- --- --- --- --- --- --- --- --- --- --- --- ... ... ... ... ... ... ... ... ...  [ 495]
H_55          --- --- --- --- --- --- --- --- --- --- --- --- --- --- --- --- --- --- --- --- --- --- --- --- ... ... ... ... ... ... ... ... ...  [ 495]
H_56          --- --- --- --- --- --- --- --- --- --- --- --- --- --- --- --- --- --- --- --- --- --- --- --- ... ... ... ... ... ... ... ... ...  [ 495]
H_57          --- --- --- --- --- --- --- --- --- --- --- --- --- --- --- --- --- ... ... ... ... ... ... ... ... ... ... ... ... ... ... ... ...  [ 495]
H_58          --- --- --- --- --- --- --- --- --- --- --- --- --- --- --- --- --- --- --- --- --- --- --- --- ... ... ... ... ... ... ... ... ...  [ 495]
H_59          --- --- --- --- --- --- --- --- --- --- --- --- --- --- --- --- --- ... ... ... ... ... ... ... ... ... ... ... ... ... ... ... ...  [ 495]
H_60          --- --- --- --- --- --- --- --- --- --- --- --- --- --- --- --- --- ... ... ... ... ... ... ... ... ... ... ... ... ... ... ... ...  [ 495]
H_61          --- --- --- --- --- --- --- --- --- --- --- --- --- --- --- --- --- ... ... ... ... ... ... ... ... ... ... ... ... ... ... ... ...  [ 495]
H_62          --- --- --- --- --- --- --- --- --- --- --- --- --- --- --- --- --- ... ... ... ... ... ... ... ... ... ... ... ... ... ... ... ...  [ 495]
H_63          --- --- --- --- --- --- --- --- --- --- --- --- --- --- --- --- --- --- --- --- --- --- --- --- ... ... ... ... ... ... ... ... ...  [ 495]
H_64          --- --- --- --- --- --- --- --- --- --- --- --- --- --- --- --- --- --- --- --- --- --- --- --- ... ... ... ... ... ... ... ... ...  [ 495]
H_65          --- --- --- GAA TCC CTA ACA GGA AGT AAT ... ... ... ... ... ... ... ... ... ... ... ... ... ... ... ... ... ... ... ... ... ... ...  [ 495]
H_66          --- --- --- --- --- --- --- --- --- --- --- --- --- --- --- --- --- ... ... ... ... ... ... ... ... ... ... ... ... ... ... ... ...  [ 495]
H_67          --- --- --- --- --- --- --- --- --- --- --- --- --- --- --- --- --- ... ... ... ... ... ... ... ... ... ... ... ... ... ... ... ...  [ 495]
H_68          --- --- --- GAA TCC CTA ACA GGA AGT AAT ... ... ... ... ... ... ... ... ... ... ... ... ... ... ... ... ... ... ... ... ... ... ...  [ 495]
H_69          --- --- --- --- --- --- --- --- --- --- --- --- --- --- --- --- --- ... ... ... ... ... ... ... ... ... ... ... ... ... ... ... ...  [ 495]
H_70          --- --- --- --- --- --- --- --- --- --- --- --- --- --- --- --- --- --- --- --- --- --- --- --- ... ... ... ... ... ... ... ... ...  [ 495]
Pcyn_rbsa     --- --- --- --- --- --- --- --- --- --- ... ..T ... ... ... ... .G. ... ..T ... ... .C. ... .G. ... ..T ... ... .C. ..C ..T ..G ...  [ 495]
Pcyn_rbsap    --- --- --- --- --- --- --- --- --- --- ... ..T ... ... ... ... .G. ... ..T ... ... .C. ... .G. ... ..T ... ... .C. ..C ..T ..G ...  [ 495]
Pinu_rbsa     --- --- --- --- --- --- --- --- --- --- --- --- --- --- --- --- --- ... ..T ... T.. .C. C.. ... .G. ..T ... T.. .C. ... ..T ... ..C  [ 495]
Pfra_rbsa     --- --- --- --- --- --- --- --- --- --- --- --- --- --- --- --- --- ..G ..T ... T.. ... ... ... --- --- --- --- --- --- --- --- ---  [ 495]

H_1           CTA GAG GCC AGT AGA GAA TCG CTA AGA GCA AGT AGA GAG TCT CTA GCG GCC AGT AGA GAA TCC CTG AAC GAC TTT TGT GGG AGC GAA GAA TCA GTA GCA  [ 594]
H_2           ... ... ... ... ... ... ... ... ... ... ... ... ... ... ... ... ... ... ... ... ... ... ... ... ... ... ... ... ... ... ... ... ...  [ 594]
H_3           ... ... ... ... ... ... ... ... ... ... ... ... ... ... ... ... ... ... ... ... ... ... ... ... ... ... ... ... ... ... ... ... ...  [ 594]
H_4           ... ... ... ... ... ... ... ... ... ... ... ... ... ... ... ... ... ... ... ... ... ... ... ... ... ... ... ... ... ... ... ... ...  [ 594]
H_5           ... ... ... ... ... ... ... ... ... ... ... ... ... ... ... ... ... ... ... ... ... ... ... ... ... ... ... ... ... ... ... ... ...  [ 594]
H_6           ... ... ... ... ... ... ... ... ... ... ... ... ... ... ... ... ... ... ... ... ... ... ... ... ... ... ... ... ... ... ... ... ...  [ 594]
H_7           ... ... ... ... ... ... ... ... ... ... ... ... ... ... ... ... ... ... ... ... ... ... ... ... ... ... ... ... ... ... ... ... ...  [ 594]
H_8           ... ... ... ... ... ... ... ... ... ... ... ... ... ... ... ... ... ... ... ... ... ... ... ... ... ... ... ... ... ... ... ... ...  [ 594]
H_9           ... ... ... ... ... ... ... ... ... ... ... ... ... ... ... ... ... ... ... ... ... ... ... ... ... ... ... ... ... ... ... ... ...  [ 594]
H_10          ... ... ... ... ... ... ... ... ... ... ... ... ... ... ... ... ... ... ... ... ... ... ... ... ... ... ... ... ... ... ... ... ...  [ 594]
H_11          ... ... ... ... ... ... ... ... ... ... ... ... ... ... ... ... ... ... ... ... ... ... ... ... ... ... ... ... ... ... ... ... ...  [ 594]
H_12          ... ... ... ... ... ... ... ... ... ... ... ... ... ... ... ... ... ... ... ... ... ... ... ... ... ... ... ... ... ... ... ... ...  [ 594]
H_13          ... ... ... ... ... ... ... ... ... ... ... ... ... ... ... ... ... ... ... ... ... ... ... ... ... ... ... ... ... ... ... ... ...  [ 594]
H_14          ... ... ... ... ... ... ... ... ... ... ... ... ... ... ... ... ... ... ... ... ... ... ... ... ... ... ... ... ... ... ... ... ...  [ 594]
H_15          ... ... ... ... ... ... ... ... ... ... ... ... ... ... ... ... ... ... ... ... ... ... ... ... ... ... ... ... ... ... ... ... ...  [ 594]
H_16          ... ... ... ... ... ... ... ... ... ... ... ... ... ... ... ... ... ... ... ... ... ... ... ... ... ... ... ... ... ... ... ... ...  [ 594]
H_17          ... ... ... ... ... ... ... ... ... ... ... ... ... ... ... ... ... ... ... ... ... ... ... ... ... ... ... ... ... ... ... ... ...  [ 594]
H_18          ... ... ... ... ... ... ... ... ... ... ... ... ... ... ... ... ... ... ... ... ... ... ... ... ... ... ... ... ... ... ... ... ...  [ 594]
H_19          ... ... ... ... ... ... ... ... ... ... ... ... ... ... ... ... ... ... ... ... ... ... ... ... ... ... ... ... ... ... ... ... ...  [ 594]
H_20          ... ... ... ... ... ... ... ... ... ... ... ... ... ... ... ... ... ... ... ... ... ... ... ... ... ... ... ... ... ... ... ... ...  [ 594]
H_21          ... ... ... ... ... ... ... ... ... ... ... ... ... ... ... ... ... ... ... ... ... ... ... ... ... ... ... ... ... ... ... ... ...  [ 594]
H_22          ... ... ... ... ... ... ... ... ... ... ... ... ... ... ... ... ... ... ... ... ... ... ... ... ... ... ... ... ... ... ... ... ...  [ 594]
H_23          ... ... ... ... ... ... ... ... ... ... ... ... ... ... ... ... ... ... ... ... ... ... ... ... ... ... ... ... ... ... ... ... ...  [ 594]
H_24          ... ... ... ... ... ... ... ... ... ... ... ... ... ... ... ... ... ... ... ... ... ... ... ... ... ... ... ... ... ... ... ... ...  [ 594]
H_25          ... ... ... ... ... ... ... ... ... ... ... ... ... ... ... ... ... ... ... ... ... ... ... ... ... ... ... ... ... ... ... ... ...  [ 594]
```

```
H_26         ... ... ... ... ... ... ... ... ... ... ... ... ... ... ... ... ... ... ... ... ... ... ... ... ... ... ... ... ... ... ... ... ...  [ 594]
H_27         ... ... ... ... ... ... ... ... ... ... ... ... ... ... ... ... ... ... ... ... ... ... ... ... ... ... ... ... ... ... ... ... ...  [ 594]
H_28         ... ... ... ... ... ... ... ... ... ... ... ... ... ... ... ... ... ... ... ... ... ... ... ... ... ... ... ... ... ... ... ... ...  [ 594]
H_29         ... ... ... ... ... ... ... ... ... ... ... ... ... ... ... ... ... ... ... ... ... ... ... ... ... ... ... ... ... ... ... ... ...  [ 594]
H_30         ... ... ... ... ... ... ... ... ... ... ... ... ... ... ... ... ... ... ... ... ... ... ... ... ... ... ... ... ... ... ... ... ...  [ 594]
H_31         ... ... ... ... ... ... ... ... ... ... ... ... ... ... ... ... ... ... ... ... ... ... ... ... ... ... ... ... ... ... ... ... ...  [ 594]
H_32         ... ... ... ... ... ... ... ... ... ... ... ... ... ... ... ... ... ... ... ... ... ... ... ... ... ... ... ... ... ... ... ... ...  [ 594]
H_33         ... ... ... ... ... ... ... ... ... ... ... ... ... ... ... ... ... ... ... ... ... ... ... ... ... ... ... ... ... ... ... ... ...  [ 594]
H_34         ... ... ... ... ... ... ... ... ... ... ... ... ... ... ... ... ... ... ... ... ... ... ... ... ... ... ... ... ... ... ... ... ...  [ 594]
H_35         ... ... ... ... ... ... ... ... ... ... ... ... ... ... ... ... ... ... ... ... ... ... ... ... ... ... ... ... ... ... ... ... ...  [ 594]
H_36         ... ... ... ... ... ... ... ... ... ... ... ... ... ... ... ... ... ... ... ... ... ... ... ... ... ... ... ... ... ... ... ... ...  [ 594]
H_37         ... ... ... ... ... ... ... ... ... ... ... ... ... ... ... ... ... ... ... ... ... ... ... ... ... ... ... ... ... ... ... ... ...  [ 594]
H_38         ... ... ... ... ... ... ... ... ... ... ... ... ... ... ... ... ... ... ... ... ... ... ... ... ... ... ... ... ... ... ... ... ...  [ 594]
H_39         ... ... ... ... ... ... ... ... ... ... ... ... ... ... ... ... ... ... ... ... ... ... ... ... ... ... ... ... ... ... ... ... ...  [ 594]
H_40         ... ... ... ... ... ... ... ... ... ... ... ... ... ... ... ... ... ... ... ... ... ... ... ... ... ... ... ... ... ... ... ... ...  [ 594]
H_41         ... ... ... ... ... ... ... ... ... ... ... ... ... ... ... ... ... ... ... ... ... ... ... ... ... ... ... ... ... ... ... ... ...  [ 594]
H_42         ... ... ... ... ... ... ... ... ... ... ... ... ... ... ... ... ... ... ... ... ... ... ... ... ... ... ... ... ... ... ... ... ...  [ 594]
H_43         ... ... ... ... ... ... ... G.. ... ... ... ... ... ... ... ... ... ... ... ... ... ... ... ... ... ... ... ... ... ... ... ... ...  [ 594]
H_44         ... ... ... ... ... ... ... ... ... ... ... ... ... ... ... ... ... ... ... ... ... ... ... ... ... ... ... ... ... ... ... ... ...  [ 594]
H_45         ... ... ... ... ... ... ... ... ... ... ... ... ... ... ... ... ... ... ... ... ... ... ... ... ... ... ... ... ... ... ... ... ...  [ 594]
H_46         ... ... ... ... ... ... ... ... ... ... ... ... ... ... ... ... ... ... ... ... ... ... ... ... ... ... ... ... ... ... ... ... ...  [ 594]
H_47         ... ... ... ... ... ... ... ... ... ... ... ... ... ... ... ... ... ... ... ... ... ... ... ... ... ... ... ... ... ... ... ... ...  [ 594]
H_48         ... ... ... ... ... ... ... ... ... ... ... ... ... ... ... ... ... ... ... ... ... ... ... ... ... ... ... ... ... ... ... ... ...  [ 594]
H_49         ... ... ... ... ... ... ... ... ... ... ... ... ... ... ... ... ... ... ... ... ... ... ... ... ... ... ... ... ... ... ... ... ...  [ 594]
H_50         ... ... ... ... ... ... ... ... ... ... ... ... ... ... ... ... ... ... ... ... ... ... ... ... ... ... ... ... ... ... ... ... ...  [ 594]
H_51         ... ... ... ... ... ... ... ... ... ... ... ... ... ... ... ... ... ... ... ... ... ... ... ... ... ... ... ... ... ... ... ... ...  [ 594]
H_52         ... ... ... ... ... ... ... ... ... ... ... ... ... ... ... ... ... ... ... ... ... ... ... ... ... ... ... ... ... ... ... ... ...  [ 594]
H_53         ... ... ... ... ... ... ... ... ... ... ... ... ... ... ... ... ... ... ... ... ... ... ... ... ... ... ... ... ... ... ... ... ...  [ 594]
H_54         ... ... ... ... ... ... ... ... ... ... ... ... ... ... ... ... ... ... ... ... ... ... ... ... ... ... ... ... ... ... ... ... ...  [ 594]
H_55         ... ... ... ... ... ... ... ... ... ... ... ... ... ... ... ... ... ... ... ... ... ... ... ... ... ... ... ... ... ... ... ... ...  [ 594]
H_56         ... ... ... ... ... ... ... ... ... ... ... ... ... ... ... ... ... ... ... ... ... ... ... ... ... ... ... ... ... ... ... ... ...  [ 594]
H_57         ... ... ... ... ... ... ... ... ... ... ... ... ... ... ... ... ... ... ... ... ... ... ... ... ... ... ... ... ... ... ... ... ...  [ 594]
H_58         ... ... ... ... ... ... ... ... ... ... ... ... ... ... ... ... ... ... ... ... ... ... ... ... ... ... ... ... ... ... ... ... ...  [ 594]
H_59         ... ... ... ... ... ... ... ... ... ... ... ... ... ... ... ... ... ... ... ... ... ... ... ... ... ... ... ... ... ... ... ... ...  [ 594]
H_60         ... ... ... ... ... ... ... ... ... ... ... ... ... ... ... ... ... ... ... ... ... ... ... ... ... ... ... ... ... ... ... ... ...  [ 594]
H_61         ... ... ... ... ... ... ... ... ... ... ... ... ... ... ... ... ... ... ... ... ... ... ... ... ... ... ... ... ... ... ... ... ...  [ 594]
H_62         ... ... ... ... ... ... ... ... ... ... ... ... ... ... ... ... ... ... ... ... ... ... ... ... ... ... ... ... ... ... ... ... ...  [ 594]
H_63         ... ... ... ... ... ... ... ... ... ... ... ... ... ... ... ... ... ... ... ... ... ... ... ... ... ... ... ... ... ... ... ... ...  [ 594]
H_64         ... ... ... ... ... ... ... ... ... ... ... ... ... ... ... ... ... ... ... ... ... ... ... ... ... ... ... ... ... ... ... ... ...  [ 594]
H_65         ... ... ... ... ... ... ... ... ... ... ... ... ... ... ... ... ... ... ... ... ... ... ... ... ... ... ... ... ... ... ... ... ...  [ 594]
H_66         ... ... ... ... ... ... ... ... ... ... ... ... ... ... ... ... ... ... ... ... ... ... ... ... ... ... ... ... ... ... ... ... ...  [ 594]
H_67         ... ... ... ... ... ... ... ... ... ... ... ... ... ... ... ... ... ... ... ... ... ... ... ... ... ... ... ... ... ... ... ... ...  [ 594]
H_68         ... ... ... ... ... ... ... ... ... ... ... ... ... ... ... ... ... ... ... ... ... ... ... ... ... ... ... ... ... ... ... ... ...  [ 594]
H_69         ... ... ... ... ... ... ... ... ... ... ... ... ... ... ... ... ... ... ... ... ... ... ... ... ... ... ... ... ... ... ... ... ...  [ 594]
H_70         ... ... ... ... ... ... ... ... ... ... ... ... ... ... ... ... ... ... ... ... ... ... ... ... ... ... ... ... ... ... ... ... ...  [ 594]
Pcyn_rbsa    ... .CA ..A G.. ..T ... ..T ... .C. .A. ... ..T ..A ... A.. ..A T.A .T. .AT ... ... ... ..T ... ... .A. ..C ... ... ... .TG A.. T..  [ 594]
Pcyn_rbsap   ... .CA ..A G.. ..T ... ..T ... .C. .A. ... ..T ..A ... A.. ..A T.A .T. .AT ... ... ... ..T ... ... .A. ..C ... ... ... .TG A.. T..  [ 594]
Pinu_rbsa    ... .CA ..A G.. ..T ... ..T ... GA. ... ... ..T ..A ... ... .AA C.A ... .AT ... ... GA. G.T ... C.. .A. .AC ... ... ... ..C ... ...  [ 594]
Pfra_rbsa    --- --- --- --- --- --- --- --- --- --- --- --- --- --- --- --- --- --- --- TT. ... ... ..T ... ..A .A. ... ... .C. ... ..G T.. ...  [ 594]

H_1          TTC GAA GGA GAG CCA AAT GAA AAG ACA TTC ATG GGA GAC GTC TTA AGT GGT GGA GAA TGT GAG AAT AGT CTC TCA AGA GAA GAT TTA TTT CAT ATA GAA  [ 693]
H_2          ... ... ... ... ... ... ... ... ... ... ... ... ... ... ... ... ... ... ... ... ... ... ... ... ... ... ... ... ... ... ... ... ...  [ 693]
H_3          .G. ... ... ... ... ... ... ... ... ... ... ... ... ... ... ... ... ... ... ... ... ... ... ... ... ... ... ... ... ... ... ... ...  [ 693]
H_4          ... ... ... ... ... ... ... ... ... ... ... ... ... ... ... ... ... ... ... ... ... ... ... ... ... ... ... ... ... ... ... ... ...  [ 693]
H_5          .G. ... ... ... ... ... ... ... ... ... ... ... ... ... ... ... ... ... ... ... ... ... ... ... ... ... ... ... ... ... ... ... ...  [ 693]
H_6          ... ... ... ... ... ... ... ... ... ... ... ... ... ... ... ... ... ... ... ... ... ... ... ... ... ... ... ... ... ... ... ... ...  [ 693]
H_7          .G. ... ... ... ... ... ... ... ... ... ... ... ... ... ... ... ... ... ... ... ... ... ... ... ... ... ... ... ... ... ... ... ...  [ 693]
H_8          ... ... ... ... ... ... ... ... ... ... ... ... ... ... ... ... ... ... ... ... ... ... ... ... ... ... ... ... ... ... ... ... ...  [ 693]
H_9          ... ... ... ... ... ... ... ... ... ... ... ... ... ... ... ... ... ... ... ... ... ... ... ... ... ... ... ... ... ... ... ... ...  [ 693]
H_10         ... ... ... ... ... ... ... ... ... ... ... ... ... ... ... ... ... ... ... ... ... ... ... ... ... ... ... ... ... ... ... ... ...  [ 693]
```

```
H_11   ... ... ... ... ... ... ... ... ... ... ... ... ... ... ... ... ... ... ... ... ... ... ... ... ... ... ... ... ... ... ... ... ...  [ 693]
H_12   ... ... ... ... ... ... ... ... ... ... ... ... ... ... ... ... ... ... ... ... ... ... ... ... ... ... ... ... ... ... ... ... ...  [ 693]
H_13   ... ... ... ... ... ... ... ... ... ... ... ... ... ... ... ... ... ... ... ... ... ... ... ... ... ... ... ... ... ... ... ... ...  [ 693]
H_14   ... ... ... ... ... ... ... ... ... ... ... ... ... ... ... ... ... ... ... ... ... ... ... ... ... ... ... ... ... ... ... ... ...  [ 693]
H_15   .G. ... ... ... ... ... ... ... ... ... ... ... ... ... ... ... ... ... ... ... ... ... ... ... ... ... ... ... ... ... ... ... ...  [ 693]
H_16   .G. ... ... ... ... ... ... ... ... ... ... ... ... ... ... ... ... ... ... ... ... ... ... ... ... ... ... ... ... ... ... ... ...  [ 693]
H_17   ... ... ... ... ... ... ... ... ... ... ... ... ... ... ... ... ... ... ... ... ... ... ... ... ... ... ... ... ... ... ... ... ...  [ 693]
H_18   ... ... ... ... ... ... ... ... ... ... ... ... ... ... ... ... ... ... ... ... ... ... ... ... ... ... ... ... ... ... ... ... ...  [ 693]
H_19   .G. ... ... ... ... ... ... ... ... ... ... ... ... ... ... ... ... ... ... ... ... ... ... ... ... ... ... ... ... ... ... ... ...  [ 693]
H_20   .G. ... ... ... ... ... ... ... ... ... ... ... ... ... ... ... ... ... ... ... ... ... ... ... ... ... ... ... ... ... ... ... ...  [ 693]
H_21   .G. ... ... ... ... ... ... ... ... ... ... ... ... ... ... ... ... ... ... ... ... ... ... ... ... ... ... ... ... ... ... ... ...  [ 693]
H_22   .G. ... ... ... ... ... ... ... ... ... ... ... ... ... ... ... ... ... ... ... ... ... ... ... ... ... ... ... ... ... ... ... ...  [ 693]
H_23   ... ... ... ... ... ... ... ... ... ... ... ... ... ... ... ... ... ... ... ... ... ... ... ... ... ... ... ... ... ... ... ... ...  [ 693]
H_24   ... ... ... ... ... ... ... ... ... ... ... ... ... ... ... ... ... ... ... ... ... ... ... ... ... ... ... ... ... ... ... ... ...  [ 693]
H_25   ... ... ... ... ... ... ... ... ... ... ... ... ... ... ... ... ... ... ... ... ... ... ... ... ... ... ... ... ... ... ... ... ...  [ 693]
H_26   ... ... ... ... ... ... ... ... ... ... ... ... ... ... ... ... ... ... ... ... ... ... ... ... ... ... ... ... ... ... ... ... ...  [ 693]
H_27   ... ... ... ... ... ... ... ... ... ... ... ... ... ... ... ... ... ... ... ... ... ... ... ... ... ... ... ... ... ... ... ... ...  [ 693]
H_28   ... ... ... ... ... ... ... ... ... ... ... ... ... ... ... ... ... ... ... ... ... ... ... ... ... ... ... ... ... ... ... ... ...  [ 693]
H_29   ... ... ... ... ... ... ... ... ... ... ... ... ... ... ... ... ... ... ... ... ... ... ... ... ... ... ... ... ... ... ... ... ...  [ 693]
H_30   ... ... ... ... ... ... ... ... ... ... ... ... ... ... ... ... ... ... ... ... ... ... ... ... ... ... ... ... ... ... ... ... ...  [ 693]
H_31   ... ... ... ... ... ... ... ... ... ... ... ... ... ... ... ... ... ... ... ... ... ... ... ... ... ... ... ... ... ... ... ... ...  [ 693]
H_32   ... ... ... ... ... ... ... ... ... ... ... ... ... ... ... ... ... ... ... ... ... ... ... ... ... ... ... ... ... ... ... ... ...  [ 693]
H_33   ... ... ... ... ... ... ... ... ... ... ... ... ... ... ... ... ... ... ... ... ... ... ... ... ... ... ... ... ... ... ... ... ...  [ 693]
H_34   ... ... ... ... ... ... ... ... ... ... ... ... ... ... ... ... ... ... ... ... ... ... ... ... ... ... ... ... ... ... ... ... ...  [ 693]
H_35   ... ... ... ... ... ... ... ... ... ... ... ... ... ... ... ... ... ... ... ... ... ... ... ... ... ... ... ... ... ... ... ... ...  [ 693]
H_36   ... ... ... ... ... ... ... ... ... ... ... ... ... ... ... ... ... ... ... ... ... ... ... ... ... ... ... ... ... ... ... ... ...  [ 693]
H_37   ... ... ... ... ... ... ... ... ... ... ... ... ... ... ... ... ... ... ... ... ... ... ... ... ... ... ... ... ... ... ... ... ...  [ 693]
H_38   .G. ... ... ... ... ... ... ... ... ... ... ... ... ... ... ... ... ... ... ... ... ... ... ... ... ... ... ... ... ... ... ... ...  [ 693]
H_39   ... ... ... ... ... ... ... ... ... ... ... ... ... ... ... ... ... ... ... ... ... ... ... ... ... ... ... ... ... ... ... ... ...  [ 693]
H_40   ... ... ... ... ... ... ... ... ... ... ... ... ... ... ... ... ... ... ... ... ... ... ... ... ... ... ... ... ... ... ... ... ...  [ 693]
H_41   ... ... ... ... ... ... ... ... ... ... ... ... ... ... ... ... ... ... ... ... ... ... ... ... ... ... ... ... ... ... ... ... ...  [ 693]
H_42   ... ... ... ... ... ... ... ... ... ... ... ... ... ... ... ... ... ... ... ... ... ... ... ... ... ... ... ... ... ... ... ... ...  [ 693]
H_43   ... ... ... ... ... ... ... ... ... ... ... ... ... ... ... ... ... ... ... ... ... ... ... ... ... ... ... ... ... ... ... ... ...  [ 693]
H_44   ... ... ... ... ... ... ... ... ... ... ... ... ... ... ... ... ... ... ... ... ... ... ... ... ... ... ... ... ... ... ... ... ...  [ 693]
H_45   ... ... ... ... ... ... ... ... ... ... ... ... ... ... ... ... ... ... ... ... ... ... ... ... ... ... ... ... ... ... ... ... ...  [ 693]
H_46   .G. ... ... ... ... ... ... ... ... ... ... ... ... ... ... ... ... ... ... ... ... ... ... ... ... ... ... ... ... ... ... ... ...  [ 693]
H_47   ... ... ... ... ... ... ... ... ... ... ... ... ... ... ... ... ... ... ... ... ... ... ... ... ... ... ... ... ... ... ... ... ...  [ 693]
H_48   .G. ... ... ... ... ... ... ... ... ... ... ... ... ... ... ... ... ... ... ... ... ... ... ... ... ... ... ... ... ... ... ... ...  [ 693]
H_49   ... ... ... ... ... ... ... ... ... ... ... ... ... ... ... ... ... ... ... ... ... ... ... ... ... ... ... ... ... ... ... ... ...  [ 693]
H_50   .G. ... ... ... ... ... ... ... ... ... ... ... ... ... ... ... ... ... ... ... ... ... ... ... ... ... ... ... ... ... ... ... ...  [ 693]
H_51   .G. ... ... ... ... ... ... ... ... ... ... ... ... ... ... ... ... ... ... ... ... ... ... ... ... ... ... ... ... ... ... ... ...  [ 693]
H_52   .G. ... ... ... ... ... ... ... ... ... ... ... ... ... ... ... ... ... ... ... ... ... ... ... ... ... ... ... ... ... ... ... ...  [ 693]
H_53   .G. ... ... ... ... ... ... ... ... ... ... ... ... ... ... ... ... ... ... ... ... ... ... ... ... ... ... ... ... ... ... ... ...  [ 693]
H_54   ... ... ... ... ... ... ... ... ... ... ... ... ... ... ... ... ... ... ... ... ... ... ... ... ... ... ... ... ... ... ... ... ...  [ 693]
H_55   ... ... ... ... ... ... ... ... ... ... ... ... ... ... ... ... ... ... ... ... ... ... ... ... ... ... ... ... ... ... ... ... ...  [ 693]
H_56   ... ... ... ... ... ... ... ... ... ... ... ... ... ... ... ... ... ... ... ... ... ... ... ... ... ... ... ... ... ... ... ... ...  [ 693]
H_57   ... ... ... ... ... ... ... ... ... ... ... ... ... ... ... ... ... ... ... ... ... ... ... ... ... ... ... ... ... ... ... ... ...  [ 693]
H_58   ... ... ... ... ... ... ... ... ... ... ... ... ... ... ... ... ... ... ... ... ... ... ... ... ... ... ... ... ... ... ... ... ...  [ 693]
H_59   ... ... ... ... ... ... ... ... ... ... ... ... ... ... ... ... ... ... ... ... ... ... ... ... ... ... ... ... ... ... ... ... ...  [ 693]
H_60   .G. ... ... ... ... ... ... ... ... ... ... ... ... ... ... ... ... ... ... ... ... ... ... ... ... ... ... ... ... ... ... ... ...  [ 693]
H_61   .G. ... ... ... ... ... ... ... ... ... ... ... ... ... ... ... ... ... ... ... ... ... ... ... ... ... ... ... ... ... ... ... ...  [ 693]
H_62   .G. ... ... ... ... ... ... ... ... ... ... ... ... ... ... ... ... ... ... ... ... ... ... ... ... ... ... ... ... ... ... ... ...  [ 693]
H_63   ... ... ... ... ... ... ... ... ... ... ... ... ... ... ... ... ... ... ... ... ... ... ... ... ... ... ... ... ... ... ... ... ...  [ 693]
H_64   ... ... ... ... ... ... ... ... ... ... ... ... ... ... ... ... ... ... ... ... ... ... ... ... ... ... ... ... ... ... ... ... ...  [ 693]
H_65   ... ... ... ... ... ... ... ... ... ... ... ... ... ... ... ... ... ... ... ... ... ... ... ... ... ... ... ... ... ... ... ... ...  [ 693]
H_66   .G. ... ... ... ... ... ... ... ... ... ... ... ... ... ... ... ... ... ... ... ... ... ... ... ... ... ... ... ... ... ... ... ...  [ 693]
```

```
H_67         ... ... ... ... ... ... ... ... ... ... ... ... ... ... ... ... ... ... ... ... ... ... ... ... ... ... ... ... ... ... ... ... ...  [ 693]
H_68         ... ... ... ... ... ... ... ... ... ... ... ... ... ... ... ... ... ... ... ... ... ... ... ... ... ... ... ... ... ... ... ... ...  [ 693]
H_69         .G. ... ... ... ... ... ... ... ... ... ... ... ... ... ... ... ... ... ... ... ... ... ... ... ... ... ... ... ... ... ... ... ...  [ 693]
H_70         ... ... ... ... ... ... ... ... ... ... ... ... ... ... ... ... ... ... ... ... ... ... ... ... ... ... ... ... ... ... ... ... ...  [ 693]
Pcyn_rbsa    .GT ... ... ... ... ... A.. ... .G. ... ..A ... ..T ... ... ..G .A. ... AT. .C. ... G.. GA. ..A .T. ... ... ... ... ... ... G.. C..  [ 693]
Pcyn_rbsap   .GT ... ... ... ... ... A.. ... .G. ... ..A ... ..T ... ... ..G .A. ... AT. .C. ... G.. GA. ..A .T. ... ... ... ... ... ... G.. C..  [ 693]
Pinu_rbsa    .GT C.. ... ... .T. ... A.C ... ..C CC. ... T.G ..T .G. ... ..G .A. ... AT. .C. ..A G.. GA. G.G ... G.. ... ... ..G .C. ... G.. ...  [ 693]
Pfra_rbsa    .GT ... ... ... ... C.. ..C ... ... ... T.T ..G ..T ... C.G ..C ... ... AT. .C. .C. G.. GA. A.A ... .A. ... ... ... ... .G. G.. ...  [ 693]

H_1          GTA GGA TCC GAA GAA TCG CTA GAT GAT GCC TCA AAA TAT AAT TTC CAA AAG GAT TTA TCA ACT AGC GAT AAT AGT TCA TTC GAA GAT GAT CAG TCA TTG  [ 792]
H_2          ... ... ... ... ... ... ... ... ... ... ... ... ... ... ... ... ... ... ... ... ... ... ... ... ... ... ... ... ... ... ... ... ...  [ 792]
H_3          ... ... ... ... ... ... ... ... ... ... ... ... ... ... ... ... ... ... ... ... ... ... ... ... ... ... ... ... ... ... ... ... ...  [ 792]
H_4          ... ... ... ... ... ... ... ... ... ... ... ... ... ... ... ... ... ... ... ... ... ... ... ... ... .T. ... ... ... ... ... ... ...  [ 792]
H_5          ... ... ... ... ... ... ... ... ... ... ... ... ... ... ... ... ... ... ... ... ... ... ... ... ... .T. ... ... ... ... ... ... ...  [ 792]
H_6          ... ... ... ... ... ... ... ... ... ... ... ... ... ... ... ... ... ... ... ... ... ... ... ... ... ... ... ... ... ... ... ... ...  [ 792]
H_7          ... ... ... ... ... ... ... ... ... ... ... ... ... ... ... ... ... ... ... ... ... ... ... ... ... ... ... ... ... ... ... ... ...  [ 792]
H_8          ... ... ... ... ... ... ... ... ... ... ... ... ... ... ... ... ... ... ... ... ... ... ... ... ... ... ... ... ... ... ... ... ...  [ 792]
H_9          ... ... ... ... ... ... ... ... ... ... ... ... ... ... ... ... ... ... ... ... ... ... ... ... ... ... ... ... ... ... ... ... ...  [ 792]
H_10         ... ... ... ... ... ... ... ... ... ... ... ... ... ... ... ... ... ... ... ... ... ... ... ... ... .T. ... ... ... ... ... ... ...  [ 792]
H_11         ... ... ... ... ... ... ... ... ... ... ... ... ... ... ... ... ... ... ... ... ... ... ... ... ... ... ... ... ... ... ... ... ...  [ 792]
H_12         ... ... ... ... ... ... ... ... ... ... ... ... ... ... ... ... ... ... ... ... ... ... ... ... ... ... ... ... ... ... ... ... ...  [ 792]
H_13         ... ... ... ... ... ... ... ... ... ... ... ... ... ... ... ... ... ... ... ... ... ... ... ... ... ... ... ... ... ... ... ... ...  [ 792]
H_14         ... ... ... ... ... ... ... ... ... ... ... ... ... ... ... ... ... ... ... ... ... ... ... ... ... ... ... ... ... ... ... ... ...  [ 792]
H_15         ... ... ... ... ... ... ... ... ... ... ... ... ... ... ... ... ... ... ... ... ... ... ... ... ... ... ... ... ... ... ... ... ...  [ 792]
H_16         ... ... ... ... ... ... ... ... ... ... ... ... ... ... ... ... ... ... ... ... ... ... ... ... ... ... ... ... ... ... ... ... ...  [ 792]
H_17         ... ... ... ... ... ... ... ... ... ... ... ... ... ... ... ... ... ... ... ... ... ... ... ... ... ... ... ... ... ... ... ... ...  [ 792]
H_18         ... ... ... ... ... ... ... ... ... ... ... ... ... ... ... ... ... ... ... ... ... ... ... ... ... ... ... ... ... ... ... ... ...  [ 792]
H_19         ... ... ... ... ... ... ... ... ... ... ... ... ... ... ... ... ... ... ... ... ... ... ... ... ... .T. ... ... ... ... ... ... ...  [ 792]
H_20         ... ... ... ... ... ... ... ... ... ... ... ... ... ... ... ... ... ... ... ... ... ... ... ... ... ... ... ... ... ... ... ... ...  [ 792]
H_21         ... ... ... ... ... ... ... ... ... ... ... ... ... ... ... ... ... ... ... ... ... ... ... ... ... ... ... ... ... ... ... ... ...  [ 792]
H_22         ... ... ... ... ... ... ... ... ... ... ... ... ... ... ... ... ... ... ... ... ... ... ... ... ... ... ... ... ... ... ... ... ...  [ 792]
H_23         ... ... ... ... ... ... ... ... ... ... ... ... ... ... ... ... ... ... ... ... ... ... ... ... ... ... ... ... ... ... ... ... ...  [ 792]
H_24         ... ... ... ... ... ... ... ... ... ... ... ... ... ... ... ... ... ... ... ... ... ... ... ... ... ... ... ... ... ... ... ... ...  [ 792]
H_25         ... ... ... ... ... ... ... ... ... ... ... ... ... ... ... ... .G. ... ... ... ... ... ... ... ... ... ... ... ... ... ... ... ...  [ 792]
H_26         ... ... ... ... ... ... ... ... ... ... ... ... ... ... ... ... ... ... ... ... ... ... ... ... ... ... ... ... ... ... ... ... ...  [ 792]
H_27         ... ... ... ... ... ... ... ... ... ... ... ... ... ... ... ... ... ... ... ... ... ... ... ... ... ... ... ... ... ... ... ... ...  [ 792]
H_28         ... ... ... ... ... ... ... ... ... ... ... ... ... ... ... ... ... ... ... ... ... ... ... ... ... ... ... ... ... ... ... ... ...  [ 792]
H_29         ... ... ... ... ... ... ... ... ... ... ... ... ... ... ... ... ... ... ... ... ... ... ... ... ... .T. ... ... ... ... ... ... ...  [ 792]
H_30         ... ... ... ... ... ... ... ... ... ... ... ... ... ... ... ... ... ... ... ... ... ... ... ... ... .T. ... ... ... ... ... ... ...  [ 792]
H_31         ... ... ... ... ... ... ... ... ... ... ... ... ... ... ... ... ... ... ... ... ... ... ... ... ... ... ... ... ... ... ... ... ...  [ 792]
H_32         ... ... ... ... ... ... ... ... ... ... ... ... ... ... ... ... ... ... ... ... ... ... ... ... ... ... ... ... ... ... ... ... ...  [ 792]
H_33         ... ... ... ... ... ... ... ... ... ... ... ... ... ... ... ... ... ... ... ... ... ... ... ... ... ... ... ... ... ... ... ... ...  [ 792]
H_34         ... ... ... ... ... ... ... ... ... ... ... ... ... ... ... ... ... ... ... ... ... ... ... ... ... .T. ... ... ... ... ... ... ...  [ 792]
H_35         ... ... ... ... ... ... ... ... ... ... ... ... ... ... ... ... ... ... ... ... ... ... ... ... ... ... ... ... ... ... ... ... ...  [ 792]
H_36         ... ... ... ... ... ... ... ... ... ... ... ... ... ... ... ... ... ... ... ... ... ... ... ... ... ... ... ... ... ... ... ... ...  [ 792]
H_37         ... ... ... ... ... ... ... ... ... ... ... ... ... ... ... ... ... ... ... ... ... ... ... ... ... ... ... ... ... ... ... ... ...  [ 792]
H_38         ... ... ... ... ... ... ... ... ... ... ... ... ... ... ... ... ... ... ... ... ... ... ... ... ... .T. ... ... ... ... ... ... ...  [ 792]
H_39         ... ... ... ... ... ... ... ... ... ... ... ... ... ... ... ... ... ... ... ... ... ... ... ... ... .T. ... ... ... ... ... ... ...  [ 792]
H_40         ... ... ... ... ... ... ... ... ... ... ... ... ... ... ... ... ... ... ... ... ... ... ... ... ... ... ... ... ... ... ... ... ...  [ 792]
H_41         ... ... ... ... ... ... ... ... ... ... ... ... ... ... ... ... ... ... ... ... ... ... ... ... ... ... ... ... ... ... ... ... ...  [ 792]
H_42         ... ... ... ... ... ... ... ... ... ... ... ... ... ... ... ... ... ... ... ... ... ... ... ... ... ... ... ... ... ... ... ... ...  [ 792]
H_43         ... ... ... ... ... ... ... ... ... ... ... ... ... ... ... ... ... ... ... ... ... ... ... ... ... ... ... ... ... ... ... ... ...  [ 792]
H_44         ... ... ... ... ... ... ... ... ... ... ... ... ... ... ... ... ... ... ... ... ... ... ... ... ... ... ... ... ... ... ... ... ...  [ 792]
H_45         ... ... ... ... ... ... ... ... ... ... ... ... ... ... ... ... ... ... ... ... ... ... ... ... ... ... ... ... ... ... ... ... ...  [ 792]
H_46         ... ... ... ... ... ... ... ... ... ... ... ... ... ... ... ... ... ... ... ... ... ... ... ... ... .T. ... ... ... ... ... ... ...  [ 792]
H_47         ... ... ... ... ... ... ... ... ... ... ... ... ... ... ... ... ... ... ... ... ... ... ... ... ... ... ... ... ... ... ... ... ...  [ 792]
H_48         ... ... ... ... ... ... ... ... ... ... ... ... ... ... ... ... ... ... ... ... ... ... ... ... ... ... ... ... ... ... ... ... ...  [ 792]
H_49         ... ... ... ... ... ... ... ... ... ... ... ... ... ... ... ... ... ... ... ... ... ... ... ... ... .T. ... ... ... ... ... ... ...  [ 792]
H_50         ... ... ... ... ... ... ... ... ... ... ... ... ... ... ... ... ... ... ... ... ... ... ... ... ... ... ... ... ... ... ... ... ...  [ 792]
H_51         ... ... ... ... ... ... ... ... ... ... ... ... ... ... ... ... ... ... ... ... ... ... ... ... ... .T. ... ... ... ... ... ... ...  [ 792]
```

```
H_52         ... ... ... ... ... ... ... ... ... ... ... ... ... ... ... ... ... ... ... ... ... ... ... ... ... ... ... ... ... ... ... ... ...  [ 792]
H_53         ... ... ... ... ... ... ... ... ... ... ... ... ... ... ... ... ... ... ... ... ... ... ... ... ... ... ... ... ... ... ... ... ...  [ 792]
H_54         ... ... ... ... ... ... ... ... ... ... ... ... ... ... ... ... ... ... ... ... ... ... ... ... ... ... ... ... ... ... ... ... ...  [ 792]
H_55         ... ... ... ... ... ... ... ... ... ... ... ... ... ... ... ... ... ... ... ... ... ... ... ... ... ... ... ... ... ... ... ... ...  [ 792]
H_56         ... ... ... ... ... ... ... ... ... ... ... ... ... ... ... ... ... ... ... ... ... ... ... ... ... ... ... ... ... ... ... ... ...  [ 792]
H_57         ... ... ... ... ... ... ... ... ... ... ... ... ... ... ... ... ... ... ... ... ... ... ... ... ... .T. ... ... ... ... ... ... ...  [ 792]
H_58         ... ... ... ... ... ... ... ... ... ... ... ... ... ... ... ... ... ... ... ... ... ... ... ... ... .T. ... ... ... ... ... ... ...  [ 792]
H_59         ... ... ... ... ... ... ... ... ... ... ... ... ... ... ... ... ... ... ... ... ... ... ... ... ... ... ... ... ... ... ... ... ...  [ 792]
H_60         ... ... ... ... ... ... ... ... ... ... ... ... ... ... ... ... ... ... ... ... ... ... ... ... ... ... ... ... ... ... ... ... ...  [ 792]
H_61         ... ... ... ... ... ... ... ... ... ... ... ... ... ... ... ... ... ... ... ... ... ... ... ... ... ... ... ... ... ... ... ... ...  [ 792]
H_62         ... ... ... ... ... ... ... ... ... ... ... ... ... ... ... ... ... ... ... ... ... ... ... ... ... ... ... ... ... ... ... ... ...  [ 792]
H_63         ... ... ... ... ... ... ... ... ... ... ... ... ... ... ... ... ... ... ... ... ... ... ... ... ... ... ... ... ... ... ... ... ...  [ 792]
H_64         ... ... ... ... ... ... ... ... ... ... ... ... ... ... ... ... ... ... ... ... ... ... ... ... ... ... ... ... ... ... ... ... ...  [ 792]
H_65         ... ... ... ... ... ... ... ... ... ... ... ... ... ... ... ... ... ... ... ... ... ... ... ... ... ... ... ... ... ... ... ... ...  [ 792]
H_66         ... ... ... ... ... ... ... ... ... ... ... ... ... ... ... ... ... ... ... ... ... ... ... ... ... ... ... ... ... ... ... ... ...  [ 792]
H_67         ... ... ... ... ... ... ... ... ... ... ... ... ... ... ... ... ... ... ... ... ... ... ... ... ... .T. ... ... ... ... ... ... ...  [ 792]
H_68         ... ... ... ... ... ... ... ... ... ... ... ... ... ... ... ... ... ... ... ... ... ... ... ... ... .T. ... ... ... ... ... ... ...  [ 792]
H_69         ... ... ... ... ... ... ... ... ... ... ... ... ... ... ... ... ... ... ... ... ... ... ... ... ... ... ... ... ... ... ... ... ...  [ 792]
H_70         ... ... ... ... ... ... ... ... ... ... ... ... ... ... ... ... ... ... ... ... ... ... ... ... ... ... ... ... ... ... ... ... ...  [ 792]
Pcyn_rbsa    .A. ... ... ... ... AT. ... A.. ..C .T. .T. ... A.. C.. .C. ... ..A ... ... ..T ... ... ..A ... ... .T. ... ... ... ... ... ... ...  [ 792]
Pcyn_rbsap   .A. ... ... ... ... AT. ... A.. ..C .TT .T. ... A.. C.. .C. ... ..A ... ... ..T ... ... ..A ... ... .T. ... ... ... ... ... ... ...  [ 792]
Pinu_rbsa    .AG ... .T. ... ... AT. ... A.. .C. ... .T. ... A.. ... ... ... ..A ... ... ..C ... ... ..A ... ... .T. .A. .G. ... ... ... .T. .C.  [ 792]
Pfra_rbsa    CA. ... .A. ... ... ATT ... A.. ... .T. .T. ... A.. ..A ... ... ..A A.A A.. ..T .T. ... .GA ... ... .T. ... C.G ... ... ... ... .G.  [ 792]

H_1          AAA AGG GGA CTC AAA AGG AAC AGC TCA GTA TCT AGT CTG GAC AGC GAT ATG GGA AGT TAT AAA AAT AAA AGT TAT AGA CAC AGA TTG GAT ATA TAT TCG  [ 891]
H_2          ... ... ... ... ... ... ... ... ... ... ... ... ... ... ... ... ... ... ... ... ... ... ... ... ... ... ... ... ... ... ... ... ...  [ 891]
H_3          ... ... ... ... ... ... ... ... ... ... ... ... ... ... ... ... ... ... ... ... ... ... GG. ..A .C. ... G.. ... ... ... ... ... ...  [ 891]
H_4          ... ... ... ... ... ... ... ... ... ... ... ... ... ... ... ... ... ... ... ... ... ... GG. ..A .C. ... G.. ... ... ... ... ... ...  [ 891]
H_5          ... ... ... ... ... ... ... ... ... ... ... ... ... ... ... ... ... ... ... ... ... ... GG. ..A .C. ... G.. ... ... ... ... ... ...  [ 891]
H_6          ... ... ... ... ... ... ... ... ... ... ... ... ... ... ... ... ... ... ... ... ... ... ... ... ... ... ... ... ... ... ... ... ...  [ 891]
H_7          ... ... ... ... ... ... ... ... ... ... ... ... ... ... ... ... ... ... ... ... ... ... GG. ..A .C. ... G.. ... ... ... ... ... ...  [ 891]
H_8          ... ... ... ... ... ... ... ... ... ... ... ... ... ... ... ... ... ... ... ... ... ... GG. ..A .C. ... G.. ... ... ... ... ... ...  [ 891]
H_9          ... ... ... ... ... ... ... ... ... ... ... ... ... ... ... ... ... ... ... ... ... ... ... ... ... ... ... ... ... ... ... ... ...  [ 891]
H_10         ... ... ... ... ... ... ... ... ... ... ... ... ... ... ... ... ... ... ... ... ... ... GG. ..A .C. ... G.. ... ... ... ... ... ...  [ 891]
H_11         ... ... ... ... ... ... ... ... ... ... ... ... ... ... ... ... ... ... ... ... ... ... ... ... ... ... ... ... ... ... ... ... ...  [ 891]
H_12         ... ... ... ... ... ... ... ... ... ... ... ... ... ... ... ... ... ... ... ... ... ... ... ... ... ... ... ... ... ... ... ... ...  [ 891]
H_13         ... ... ... ... ... ... ... ... ... ... ... ... ... ... ... ... ... ... ... ... ... ... ... ... ... ... ... ... ... ... ... ... ...  [ 891]
H_14         ... ... ... ... ... ... ... ... ... ... ... ... ... ... ... ... ... ... ... ... ... ... ... ... ... ... ... ... ... ... ... ... ...  [ 891]
H_15         ... ... ... ... ... ... ... ... ... ... ... ... ... ... ... ... ... ... ... ... ... ... GG. ..A .C. ... G.. ... ... ... ... ... ...  [ 891]
H_16         ... ... ... ... ... ... ... ... ... ... ... ... ... ... ... ... ... ... ... ... ... ... ... ... ... ... ... ... ... ... ... ... ...  [ 891]
H_17         ... ... ... ... ... ... ... ... ... ... ... ... ... ... ... ... ... ... ... ... ... ... ... ... ... ... ... ... ... ... ... ... ...  [ 891]
H_18         ... ... ... ... ... ... ... ... ... ... ... ... ... ... ... ... ... ... ... ... ... ... ... ... ... ... ... ... ... ... ... ... ...  [ 891]
H_19         ... ... ... ... ... ... ... ... ... ... ... ... ... ... ... ... ... ... ... ... ... ... ... ... ... ... ... ... ... ... ... ... ...  [ 891]
H_20         ... ... ... ... ... ... ... ... ... ... ... ... ... ... ... ... ... ... ... ... ... ... ... ... ... ... ... ... ... ... ... ... ...  [ 891]
H_21         ... ... ... ... ... ... ... ... ... ... ... ... ... ... ... ... ... ... ... ... ... ... ... ... ... ... ... ... ... ... ... ... ...  [ 891]
H_22         ... ... ... ... ... ... ... ... ... ... ... ... ... ... ... ... ... ... ... ... ... ... ... ... ... ... ... ... ... ... ... ... ...  [ 891]
H_23         ... ... ... ... ... ... ... ... ... ... ... ... ... ... ... ... ... ... ... ... ... ... ... ... ... ... ... ... ... ... ... ... ...  [ 891]
H_24         ... ... ... ... ... ... ... ... ... ... ... ... ... ... ... ... ... ... ... ... ... ... ... ... ... ... ... ... ... ... ... ... ...  [ 891]
H_25         ... ... ... ... ... ... ... ... ... ... ... ... ... ... ... ... ... ... ... ... ... ... ... ... ... ... ... ... ... ... ... ... ...  [ 891]
H_26         ... ... ... ... ... ... ... ... ... ... ... ... ... ... ... ... ... ... ... ... ... ... ... ... ... ... ... ... ... ... ... ... ...  [ 891]
H_27         ... ... ... ... ... ... ... ... ... ... ... ... ... ... ... ... ... ... ... ... ... ... ... ... ... ... ... ... ... ... ... ... ...  [ 891]
H_28         ... ... ... ... ... ... ... ... ... ... ... ... ... ... ... ... ... ... ... ... ... ... ... ... ... ... ... ... ... ... ... ... ...  [ 891]
H_29         ... ... ... ... ... ... ... ... ... ... ... ... ... ... ... ... ... ... ... ... ... ... GG. ..A .C. ... G.. ... ... ... ... ... ...  [ 891]
H_30         ... ... ... ... ... ... ... ... ... ... ... ... ... ... ... ... ... ... ... ... ... ... GG. ..A .C. ... G.. ... ... ... ... ... ...  [ 891]
H_31         ... ... ... ... ... ... ... ... ... ... ... ... ... ... ... ... ... ... ... ... ... ... GG. ..A .C. ... G.. ... ... ... ... ... ...  [ 891]
H_32         ... ... ... ... ... ... ... ... ... ... ... ... ... ... ... ... ... ... ... ... ... ... GG. ..A .C. ... G.. ... ... .T. ... ... ...  [ 891]
```

```
H_33        ... ... ... ... ... ... ... ... ... ... ... ... ... ... ... ... ... ... ... ... ... ... ... ... ... ... ... ... ... ... ... ... ...  [ 891]
H_34        ... ... ... ... ... ... ... ... ... ... ... ... ... ... ... ... ... ... ... ... ... ... ... ... ... ... ... ... ... ... ... ... ...  [ 891]
H_35        ... ... ... ... ... ... ... ... ... ... ... ... ... ... ... ... ... ... ... ... ... ... GG. ..A .C. ... G.. ... ... ... ... ... ...  [ 891]
H_36        ... ... ... ... ... ... ... ... ... ... ... ... ... ... ... ... ... ... ... ... ... ... GG. ..A .C. ... G.. ... ... ... ... ... ...  [ 891]
H_37        ... ... ... ... ... ... ... ... ... ... ... ... ... ... ... ... ... ... ... ... ... ... ... ... ... ... ... ... ... ... ... ... ...  [ 891]
H_38        ... ... ... ... ... ... ... ... ... ... ... ... ... ... ... ... ... ... ... ... ... ... GG. ..A .C. ... G.. ... ... ... ... ... ...  [ 891]
H_39        ... ... ... ... ... ... ... ... ... ... ... ... ... ... ... ... ... ... ... ... ... ... GG. ..A .C. ... G.. ... ... ... ... ... ...  [ 891]
H_40        ... ... ... ... ... ... ... ... ... ... ... ... ... ... ... ... ... ... ... ... ... ... GG. ..A ... ... G.. ... ... ... ... ... ...  [ 891]
H_41        ... ... ... ... ... ... ... ... ... ... ... ... ... ... ... ... ... ... ... ... ... ... ... ... ... ... ... ... ... ... ... ... ...  [ 891]
H_42        ... ... ... ... ... ... ... ... ... ... ... ... ... ... ... ... ... ... ... ... ... ... ... ... ... ... ... ... ... ... ... ... ...  [ 891]
H_43        ... ... ... ... ... ... ... ... ... ... ... ... ... ... ... ... ... ... ... ... ... ... GG. ..A .C. ... G.. ... ... ... ... ... ...  [ 891]
H_44        ... ... ... ... ... ... ... ... ... ... ... ... ... ... ... ... ... ... ... ... ... ... GG. ..A ... ... ... ... ... ... ... ... ...  [ 891]
H_45        ... ... ... ... ... ... ... ... ... ... ... ... ... ... ... ... ... ... ... ... ... ... GG. ..A .C. ... G.. ... ... ... ... ... ...  [ 891]
H_46        ... ... ... ... ... ... ... ... ... ... ... ... ... ... ... ... ... ... ... ... ... ... GG. ..A .C. ... G.. ... ... ... ... ... ...  [ 891]
H_47        ... ... ... ... ... ... ... ... ... ... ... ... ... ... ... ... ... ... ... ... ... ... GG. ..A .C. ... G.. ... ... ... ... ... ...  [ 891]
H_48        ... ... ... ... ... ... ... ... ... ... ... ... ... ... ... ... ... ... ... ... ... ... ... ... ... ... ... ... ... ... ... ... ...  [ 891]
H_49        ... ... ... ... ... ... ... ... ... ... ... ... ... ... ... ... ... ... ... ... ... ... ... ..A .C. ... G.. ... ... ... ... ... ...  [ 891]
H_50        ... ... ... ... ... ... ... ... ... ... ... ... ... ... ... ... ... ... ... ... ... ... ... ... ... ... ... ... ... ... ... ... ...  [ 891]
H_51        ... ... ... ... ... ... ... ... ... ... ... ... ... ... ... ... ... ... ... ... ... ... GG. ..A .C. ... G.. ... ... ... ... ... ...  [ 891]
H_52        ... ... ... ... ... ... ... ... ... ... ... ... ... ... ... ... ... ... ... ... ... ... GG. ..A .C. ... G.. ... ... ... ... ... ...  [ 891]
H_53        ... ... ... ... ... ... ... ... ... ... ... ... ... ... ... ... ... ... ... ... ... ... ... ... ... ... ... ... ... ... ... ... ...  [ 891]
H_54        ... ... ... ... ... ... ... ... ... .A. ... ... ... ... ... ... ... ... ... ... ... ... ... ... ... ... ... ... ... ... ... ... ...  [ 891]
H_55        ... ... ... ... ... ... ... ... ... ... ... ... ... ... ... ... ... ... ... ... ... ... ... ... ... ... ... ... ... ... ... ... ...  [ 891]
H_56        ... ... ... ... ... ... ... ... ... .A. ... ... ... ... ... ... ... ... ... ... ... ... ... ... ... ... ... ... ... ... ... ... ...  [ 891]
H_57        ... ... ... ... ... ... ... ... ... ... ... ... ... ... ... ... ... ... ... ... ... ... GG. ..A .C. ... G.. ... ... ... ... ... ...  [ 891]
H_58        ... ... ... ... ... ... ... ... ... ... ... ... ... ... ... ... ... ... ... ... ... ... ... ... ... ... ... ... ... ... ... ... ...  [ 891]
H_59        ... ... ... ... ... ... ... ... ... ... ... ... ... ... ... ... ... ... ... ... ... ... GG. ..A .C. ... G.. ... ... ... ... ... ...  [ 891]
H_60        ... ... ... ... ... ... ... ... ... ... ... ... ... ... ... ... ... ... ... ... ... ... GG. ..A .C. ... G.. ... ... ... ... ... ...  [ 891]
H_61        ... ... ... ... ... ... ... ... ... ... ... ... ... ... ... ... ... ... ... ... ... ... GG. ..A .C. ... G.. ... ... ... ... ... ...  [ 891]
H_62        ... ... ... ... ... ... ... ... ... ... ... ... ... ... ... ... ... ... ... ... ... ... GG. ..A ... ... G.. ... ... ... ... ... ...  [ 891]
H_63        ... ... ... ... ... ... ... ... ... .A. ... ... ... ... ... ... ... ... ... ... ... ... ... ... ... ... ... ... ... ... ... ... ...  [ 891]
H_64        ... ... ... ... ... ... ... ... ... ... ... ... ... ... ... ... ... ... ... ... ... ... ... ... ... ... ... ... ... ... ... ... ...  [ 891]
H_65        ... ... ... ... ... ... ... ... ... ... ... ... ... ... ... ... ... ... ... ... ... ... GG. ..A .C. ... G.. ... ... ... ... ... ...  [ 891]
H_66        ... ... ... ... ... ... ... ... ... ... ... ... ... ... ... ... ... ... ... ... ... ... GG. ..A .C. ... G.. ... ... ... ... ... ...  [ 891]
H_67        ... ... ... ... ... ... ... ... ... ... ... ... ... ... ... ... ... ... ... ... ... ... GG. ..A .C. ... G.. ... ... ... ... ... ...  [ 891]
H_68        ... ... ... ... ... ... ... ... ... ... ... ... ... ... ... ... ... ... ... ... ... ... GG. ..A .C. ... G.. ... ... ... ... ... ...  [ 891]
H_69        ... ... ... ... ... ... ... ... ... ... ... ... ... ... ... ... ... ... ... ... ... ... G.. ... ... ... G.. ... ... ... ... ... ...  [ 891]
H_70        ... ... ... ... ... ... ... ... ... ... ... ... ... ... ... ... ... ... ... ... ... ... .G. ..A ... ... ... ... ... ... ... ... ...  [ 891]
Pcyn_rbsa   ... ..T ..C T.. .G. .AA ... T.. ... .A. A.. ... ... ... ... T.. ... ... ... .T. ... ... GG. ..A .C. ... ... G.. ... ... ... G.. ...  [ 891]
Pcyn_rbsap  ... ..T ..C T.. .G. .AA ... T.. ... .A. A.. ... ... ... ... T.. ... ... ... .T. ... ... GG. ..A .C. ... ... G.. ... ... ... G.. ...  [ 891]
Pinu_rbsa   ... .TC ..C T.. .GT .AT ..T T.. ... .A. A.. .A. ... ... ... ..C ... ... GA. .T. ... .G. GG. ..G .C. .C. ... G.. ..C ... ... G.. AT.  [ 891]
Pfra_rbsa   .G. G.. .AC T.. .G. ... ... TA. ... .A. A.. ... ... ... ..A ..C ... .A. ... .T. ... ... G.. .AA .C. ... G.G G.. ... A.. ... G.. ...  [ 891]

H_1         GAC CTA CCT AGA AGG CCA ATA CAT GGG GAT AAT GCA CCA CAA GAA AAG GAG AAA TGT CTG GAT TTC AAA GAA CTT CTA GAG AGA GAA AGG AAC AAC CAC  [ 990]
H_2         ... ... ... ... ... ... ... ... ... ... ... ... ... ... ... ... ... ... ... ... ... ... ... ... ... ... ... ... ... ... ... ... ...  [ 990]
H_3         ... ... ... ... ... .T. ... ... ... ... ... ... ... ... ... ... ... ... ... ... ... ... ... ... ... ... ... ... ... ... ... ... ...  [ 990]
H_4         ... ... ... ... ... .T. ... ... ... ... ... ... ... ... ... ... ... ... ... ... ... ... ... ... ... ... ... ... ... ... ... ... ...  [ 990]
H_5         ... ... ... ... ... ... ... ... ... ... ... ... ... ... ... ... ... ... ... ... ... ... ... ... ... ... ... ... ... ... ... ... ...  [ 990]
H_6         ... ... ... ... ... ... ... ... ... ... ... ... ... ... ... ... ... ... ... ... ... ... ... ... ... ... ... ... ... ... ... ... ...  [ 990]
H_7         ... ... ... ... ... .T. ... ... ... ... ... ... ... ... ... ... ... ... ... ... ... ... ... ... ... ... ... ... ... ... ... ... ...  [ 990]
H_8         ... ... ... ... ... ... ... ... ... ... ... ... ... ... ... ... ... ... ... ... ... ... ... ... ... ... ... ... ... ... ... ... ...  [ 990]
H_9         ... ... ... ... ... ... ... ... ... ... ... ... ... ... ... ... ... ... ... ... ... ... ... ... ... ... ... ... ... ... ... ... ...  [ 990]
H_10        ... ... ... ... ... .T. ... ... ... ... ... ... ... ... ... ... ... ... ... ... ... ... ... ... ... ... ... ... ... ... ... ... ...  [ 990]
H_11        ... ... ... ... ... ... ... ... ... ... ... ... ... ... ... ... ... ... ... ... ... ... ... ... ... ... ... ... ... ... ... ... ...  [ 990]
H_12        ... ... ... ... ... .T. ... ... ... ... ... ... ... ... ... ... ... ... ... ... ... ... ... ... ... ... ... ... ... ... ... ... ...  [ 990]
H_13        ... ... ... ... ... ... ... ... ... ... ... ... ... ... ... ... ... ... ... ... ... ... ... ... ... ... ... ... ... ... ... ... ...  [ 990]
H_14        ... ... ... ... ... ... ... ... ... ... ... ... ... ... ... ... ... ... ... ... ... ... ... ... ... ... ... ... ... ... ... ... ...  [ 990]
H_15        ... ... ... ... ... .T. ... ... ... ... ... ... ... ... ... ... ... ... ... ... ... ... ... ... ... ... ... ... ... ... ... ... ...  [ 990]
H_16        ... ... ... ... ... ... ... ... ... ... ... ... ... ... ... ... ... ... ... ... ... ... ... ... ... ... ... ... ... ... ... ... ...  [ 990]
H_17        ... ... ... ... ... ... ... ... ... ... ... ... ... ... ... ... ... ... ... ... ... ... ... ... ... ... ... ... ... ... ... ... ...  [ 990]
```

```
H_18         ... ... ... ... ... ... ... ... ... ... ... ... ... ... ... ... ... ... ... ... ... ... ... ... ... ... ... ... ... ... ... ... ...  [ 990]
H_19         ... ... ... ... ... ... ... ... ... ... ... ... ... ... ... ... ... ... ... ... ... ... ... ... ... ... ... ... ... ... ... ... ...  [ 990]
H_20         ... ... ... ... ... ... ... ... ... ... ... ... ... ... ... ... ... ... ... ... ... ... ... ... ... ... ... ... ... ... ... ... ...  [ 990]
H_21         ... ... ... ... ... ... ... ... ... ... ... ... ... ... ... ... ... ... ... ... ... ... ... ... ... ... ... ... ... ... ... ... ...  [ 990]
H_22         ... ... ... ... ... ... ... ... ... ... ... ... ... ... ... ... ... ... ... ... ... ... ... ... ... ... ... ... ... ... ... ... ...  [ 990]
H_23         ... ... ... ... ... .T. ... ... ... ... ... ... ... ... ... ... ... ... ... ... ... ... ... ... ... ... ... ... ... ... ... ... ...  [ 990]
H_24         ... ... ... ... ... ... ... ... ... ... ... ... ... ... ... ... ... ... ... ... ... ... ... ... ... ... ... ... ... ... ... ... ...  [ 990]
H_25         ... ... ... ... ... ... ... ... ... ... ... ... ... ... ... ... ... ... ... ... ... ... ... ... ... ... ... ... ... ... ... ... ...  [ 990]
H_26         ... ... ... ... ... ... ... ... ... ... ... ... ... ... ... ... ... ... ... ... ... ... ... ... ... ... ... ... ... ... ... ... ...  [ 990]
H_27         ... ... ... ... ... ... ... ... ... ... ... ... ... ... ... ... ... ... ... ... ... ... ... ... ... ... ... ... ... ... ... ... ...  [ 990]
H_28         ... ... ... ... ... ... ... ... ... ... ... ... ... ... ... ... ... ... ... ... ... ... ... ... ... ... ... ... ... ... ... ... ...  [ 990]
H_29         ... ... ... ... ... .T. ... ... ... ... ... ... ... ... ... ... ... ... ... ... ... ... ... ... ... ... ... ... ... ... ... ... ...  [ 990]
H_30         ... ... ... ... ... .T. ... ... ... ... ... ... ... ... ... ... ... ... ... ... ... ... ... ... ... ... ... ... ... ... ... ... ...  [ 990]
H_31         ... ... ... ... ... .T. ... ... ... ... ... ... ... ... ... ... ... ... ... ... ... ... ... ... ... ... ... ... ... ... ... ... ...  [ 990]
H_32         ... ... ... ... ... ... ... ... ... ... ... ... ... ... ... ... ... ... ... ... ... ... ... ... ... ... ... ... ... ... ... ... ...  [ 990]
H_33         ... ... ... ... ... ... ... ... ... ... ... ... ... ... ... ... ... ... ... ... ... ... ... ... ... ... ... ... ... ... ... ... ...  [ 990]
H_34         ... ... ... ... ... .T. ... ... ... ... ... ... ... ... ... ... ... ... ... ... ... ... ... ... ... ... ... ... ... ... ... ... ...  [ 990]
H_35         ... ... ... ... ... .T. ... ... ... ... ... ... ... ... ... ... ... ... ... ... ... ... ... ... ... ... ... ... ... ... ... ... ...  [ 990]
H_36         ... ... ... ... ... .T. ... ... ... ... ... ... ... ... ... ... ... ... ... ... ... ... ... ... ... ... ... ... ... ... ... ... ...  [ 990]
H_37         ... ... ... ... ... ... ... ... ... ... ... ... ... ... ... ... ... ... ... ... ... ... ... ... ... ... ... ... ... ... ... ... ...  [ 990]
H_38         ... ... ... ... ... .T. ... ... ... ... ... ... ... ... ... ... ... ... ... ... ... ... ... ... ... ... ... ... ... ... ... ... ...  [ 990]
H_39         ... ... ... ... ... .T. ... ... ... ... ... ... ... ... ... ... ... ... ... ... ... ... ... ... ... ... ... ... ... ... ... ... ...  [ 990]
H_40         ... ... ... ... ... ... ... ... ... ... ... ... ... ... ... ... ... ... ... ... ... ... ... ... ... ... ... ... ... ... ... ... ...  [ 990]
H_41         ... ... ... ... ... ... ... ... ... ... ... ... ... ... ... ... ... ... ... ... ... ... ... ... ... ... ... ... ... ... ... ... ...  [ 990]
H_42         ... ... ... ... ... ... ... ... ... ... ... ... ... ... ... ... ... ... ... ... ... ... ... ... ... ... ... ... ... ... ... ... ...  [ 990]
H_43         ... ... ... ... ... ... ... ... ... ... ... ... ... ... ... ... ... ... ... ... ... ... ... ... ... ... ... ... ... ... ... ... ...  [ 990]
H_44         ... ... ... ... ... ... ... ... ... ... ... ... ... ... ... ... ... ... ... ... ... ... ... ... ... ... ... ... ... ... ... ... ...  [ 990]
H_45         ... ... ... ... ... .T. ... ... ... ... ... ... ... ... ... ... ... ... ... ... ... ... ... ... ... ... ... ... ... ... ... ... ...  [ 990]
H_46         ... ... ... ... ... .T. ... ... ... ... ... ... ... ... ... ... ... ... ... ... ... ... ... ... ... ... ... ... ... ... ... ... ...  [ 990]
H_47         ... ... ... ... ... .T. ... ... ... ... ... ... ... ... ... ... ... ... ... ... ... ... ... ... ... ... ... ... ... ... ... ... ...  [ 990]
H_48         ... ... ... ... ... ... ... ... ... ... ... ... ... ... ... ... ... ... ... ... ... ... ... ... ... ... ... ... ... ... ... ... ...  [ 990]
H_49         ... ... ... ... ... .T. ... ... ... ... ... ... ... ... ... ... ... ... ... ... ... ... ... ... ... ... ... ... ... ... ... ... ...  [ 990]
H_50         ... ... ... ... ... ... ... ... ... ... ... ... ... ... ... ... ... ... ... ... ... ... ... ... ... ... ... ... ... ... ... ... ...  [ 990]
H_51         ... ... ... ... ... .T. ... ... ... ... ... ... ... ... ... ... ... ... ... ... ... ... ... ... ... ... ... ... ... ... ... ... ...  [ 990]
H_52         ... ... ... ... ... .T. ... ... ... ... ... ... ... ... ... ... ... ... ... ... ... ... ... ... ... ... ... ... ... ... ... ... ...  [ 990]
H_53         ... ... ... ... ... ... ... ... ... ... ... ... ... ... ... ... ... ... ... ... ... ... ... ... ... ... ... ... ... ... ... ... ...  [ 990]
H_54         ... ... ... ... ... ... ... ... ... ... ... ... ... ... ... ... ... ... ... ... ... ... ... ... ... ... ... ... ... ... ... ... ...  [ 990]
H_55         ... ... ... ... ... ... ... ... ... ... ... ... ... ... ... ... ... ... ... ... ... ... ... ... ... ... ... ... ... ... ... ... ...  [ 990]
H_56         ... ... ... ... ... ... ... ... ... ... ... ... ... ... ... ... ... ... ... ... ... ... ... ... ... ... ... ... ... ... ... ... ...  [ 990]
H_57         ... ... ... ... ... .T. ... ... ... ... ... ... ... ... ... ... ... ... ... ... ... ... ... ... ... ... ... ... ... ... ... ... ...  [ 990]
H_58         ... ... ... ... ... .T. ... ... ... ... ... ... ... ... ... ... ... ... ... ... ... ... ... ... ... ... ... ... ... ... ... ... ...  [ 990]
H_59         ... ... ... ... ... .T. ... ... ... ... ... ... ... ... ... ... ... ... ... ... ... ... ... ... ... ... ... ... ... ... ... ... ...  [ 990]
H_60         ... ... ... ... ... .T. ... ... ... ... ... ... ... ... ... ... ... ... ... ... ... ... ... ... ... ... ... ... ... ... ... ... ...  [ 990]
H_61         ... ... ... ... ... .T. ... ... ... ... ... ... ... ... ... ... ... ... ... ... ... ... ... ... ... ... ... ... ... ... ... ... ...  [ 990]
H_62         ... ... ... ... ... ... ... ... ... ... ... ... ... ... ... ... ... ... ... ... ... ... ... ... ... ... ... ... ... ... ... ... ...  [ 990]
H_63         ... ... ... ... ... ... ... ... ... ... ... ... ... ... ... ... ... ... ... ... ... ... ... ... ... ... ... ... ... ... ... ... ...  [ 990]
H_64         ... ... ... ... ... .T. ... ... ... ... ... ... ... ... ... ... ... ... ... ... ... ... ... ... ... ... ... ... ... ... ... ... ...  [ 990]
H_65         ... ... ... ... ... .T. ... ... ... ... ... ... ... ... ... ... ... ... ... ... ... ... ... ... ... ... ... ... ... ... ... ... ...  [ 990]
H_66         ... ... ... ... ... .T. ... ... ... ... ... ... ... ... ... ... ... ... ... ... ... ... ... ... ... ... ... ... ... ... ... ... ...  [ 990]
H_67         ... ... ... ... ... .T. ... ... ... ... ... ... ... ... ... ... ... ... ... ... ... ... ... ... ... ... ... ... ... ... ... ... ...  [ 990]
H_68         ... ... ... ... ... .T. ... ... ... ... ... ... ... ... ... ... ... ... ... ... ... ... ... ... ... ... ... ... ... ... ... ... ...  [ 990]
H_69         ... ... ... ... ... ... ... ... ... ... ... ... ... ... ... ... ... ... ... ... ... ... ... ... ... ... ... ... ... ... ... ... ...  [ 990]
H_70         ... ... ... ... ... ... ... ... ... ... ... ... ... ... ... ... ... ... ... ... ... ... ... ... ... ... ... ... ... ... ... ... ...  [ 990]
Pcyn_rbsa    ..T .C. .A. ... ... ..T .CG ... ..A .G. ... .A. ... ... .G. G.. ... ... .C. G.. A.C ... ... ... .A. ... .G. ... C.. ... --- ... ..A  [ 990]
Pcyn_rbsap   ..T .C. .A. ... ... ..T .CG ... ..A .G. ... .A. ... ... .G. G.. ... ... .C. G.. A.C ... ... ... .A. ... .G. ... C.. ... --- ... ..A  [ 990]
Pinu_rbsa    ... AC. ... .AG ... T.T .CG G.. A.A ... C.. ... ..C ... .G. G.. ... G.. ..G A.. A.. G.. ... .C. GA. ... C.. ... C.. ... --- ..T ...  [ 990]
```

```
Pfra_rbsa     ..A GA. ... ... ..A T.T .CG ... ..A .T. ... ..T ..G ..G .G. G.. ... ... .A. G.. A.C ..G C.. ... .A. ..G ... G.. C.. ... --- ... ...  [ 990]

H_1           CAA AGG AAT AGT GTA AAT ATA AAA TTA AAA AAT ATA TAC GGA GAA GAA CAA TTG TAT AAT GTG AAG AAA GAT AGC TTT ATT GTA AAA TTG ATA TAC TAT  [1089]
H_2           ... ... ... ... ... ... ... ... ... ... ... ... ... ... ... ... ... ... ... ... ... ... ... ... ... ... ... ... ... ... ... ... ...  [1089]
H_3           ... ... ... ... ... ... ... ... ... ... ... ... ... ... ... ... ... ... ... ... ... ... ... ... ... ... ... ... ... ... ... ... ...  [1089]
H_4           ... ... ... ... ... ... ... ... ... ... ... ... ... ... ... ... ... ... ... ... ... ... ... ... ... ... ... ... ... ... ... ... ...  [1089]
H_5           ... ... ... ... ... ... ... ... ... ... ... ... ... ... ... ... ... ... ... ... ... ... ... ... ... ... ... ... ... ... ... ... ...  [1089]
H_6           ... ... ... ... ... ... ... ... ... ... ... ... ... ... ... ... ... ... ... ... ... ... ... ... ... ... ... ... ... ... ... ... ...  [1089]
H_7           ... ... ... ... ... ... ... ... ... ... ... ... ... ... ... ... ... ... ... ... ... ... ... ... ... ... ... ... ... ... ... ... ...  [1089]
H_8           ... ... ... ... ... ... ... ... ... ... ... ... ... ... ... ... ... ... ... ... ... ... ... ... ... ... ... ... ... ... ... ... ...  [1089]
H_9           ... ... ... ... ... ... ... ... ... ... ... ... ... ... ... ... ... ... ... ... ... ... ... ... ... ... ... ... ... ... ... ... ...  [1089]
H_10          ... ... ... ... ... ... ... ... ... ... ... ... ... ... ... ... ... ... ... ... ... ... ... ... ... ... ... ... ... ... ... ... ...  [1089]
H_11          ... ... ... ... ... ... ... ... ... ... ... ... ... ... ... ... ... ... ... ... ... ... ... ... ... ... ... ... ... ... ... ... ...  [1089]
H_12          ... ... ... ... ... ... ... ... ... ... ... ... ... ... ... ... ... ... ... ... ... ... ... ... ... ... ... ... ... ... ... ... ...  [1089]
H_13          ... ... ... ... ... ... ... ... ... ... ... ... ... ... ... ... ... ... ... ... ... ... ... ... ... ... ... ... ... ... ... ... ...  [1089]
H_14          ... ... ... ... ... ... ... ... ... ... ... ... ... ... ... ... ... ... ... ... ... ... ... ... ... ... ... ... ... ... ... ... ...  [1089]
H_15          ... ... ... ... ... ... ... ... ... ... ... ... ... ... ... ... ... ... ... ... ... ... ... ... ... ... ... ... ... ... ... ... ...  [1089]
H_16          ... ... ... ... ... ... ... ... ... ... ... ... ... ... ... ... ... ... ... ... ... ... ... ... ... ... ... ... ... ... ... ... ...  [1089]
H_17          ... ... ... ... ... ... ... ... ... ... ... ... ... ... ... ... ... ... ... ... ... ... ... ... ... ... ... ... ... ... ... ... ...  [1089]
H_18          ... ... ... ... ... ... ... ... ... ... ... ... ... ... ... ... ... ... ... ... ... ... ... ... ... ... ... ... ... ... ... ... ...  [1089]
H_19          ... ... ... ... ... ... ... ... ... ... ... ... ... ... ... ... ... ... ... ... ... ... ... ... ... ... ... ... ... ... ... ... ...  [1089]
H_20          ... ... ... ... ... ... ... ... ... ... ... ... ... ... ... ... ... ... ... ... ... ... ... ... ... ... ... ... ... ... ... ... ...  [1089]
H_21          ... ... ... ... ... ... ... ... ... ... ... ... ... ... ... ... ... ... ... ... ... ... ... ... ... ... ... ... ... ... ... ... ...  [1089]
H_22          ... ... ... ... ... ... ... ... ... ... ... ... ... ... ... ... ... ... ... ... ... ... ... ... ... ... ... ... ... ... ... ... ...  [1089]
H_23          ... ... ... ... ... ... ... ... ... ... ... ... ... ... ... ... ... ... ... ... ... ... ... ... ... ... ... ... ... ... ... ... ...  [1089]
H_24          ... ... ... ... ... ... ... ... ... ... ... ... ... ... ... ... ... ... ... ... ... ... ... ... ... ... ... ... ... ... ... ... ...  [1089]
H_25          ... ... ... ... ... ... ... ... ... ... ... ... ... ... ... ... ... ... ... ... ... ... ... ... ... ... ... ... ... ... ... ... ...  [1089]
H_26          ... ... ... ... ... ... ... ... ... ... ... ... ... ... ... ... ... ... ... ... ... ... ... ... ... ... ... ... ... ... ... ... ...  [1089]
H_27          ... ... ... ... ... ... ... ... ... ... ... ... ... ... ... ... ... ... ... ... ... ... ... ... ... ... ... ... ... ... ... ... ...  [1089]
H_28          ... ... ... ... ... ... ... ... ... ... ... ... ... ... ... ... ... ... ... ... ... ... ... ... ... ... ... ... ... ... ... ... ...  [1089]
H_29          ... ... ... ... ... ... ... ... ... ... ... ... ... ... ... ... ... ... ... ... ... ... ... ... ... ... ... ... ... ... ... ... ...  [1089]
H_30          ... ... ... ... ... ... ... ... ... ... ... ... ... ... ... ... ... ... ... ... ... ... ... ... ... ... ... ... ... ... ... ... ...  [1089]
H_31          ... ... ... ... ... ... ... ... ... ... ... ... ... ... ... ... ... ... ... ... ... ... ... ... ... ... ... ... ... ... ... ... ...  [1089]
H_32          ... ... ... ... ... ... ... ... ... ... ... ... ... ... ... ... ... ... ... ... ... ... ... ... ... ... ... ... ... ... ... ... ...  [1089]
H_33          ... ... ... ... ... ... G.. ... ... ... ... ..T ... ... ... ... ... A.. ... ..A ... ... .G. A.. ... ... ... ... ... ... ... ... ...  [1089]
H_34          ... ... ... ... ... ... ... ... ... ... ... ... ... ... ... ... ... ... ... ... ... ... ... ... ... ... ... ... ... ... ... ... ...  [1089]
H_35          ... ... ... ... ... ... ... ... ... ... ... ... ... ... ... ... ... ... ... ... ... ... ... ... ... ... ... ... ... ... ... ... ...  [1089]
H_36          ... ... ... ... ... ... ... ... ... ... ... ... ... ... ... ... ... ... ... ... ... ... ... ... ... ... ... ... ... ... ... ... ...  [1089]
H_37          ... ... ... ... ... ... ... ... ... ... ... ... ... ... ... ... ... ... ... ... ... ... ... ... ... ... ... ... ... ... ... ... ...  [1089]
H_38          ... ... ... ... ... ... ... ... ... ... ... ... ... ... ... ... ... ... ... ... ... ... ... ... ... ... ... ... ... ... ... ... ...  [1089]
H_39          ... ... ... ... ... ... ... ... ... ... ... ... ... ... ... ... ... ... ... ... ... ... ... ... ... ... ... ... ... ... ... ... ...  [1089]
H_40          ... ... ... ... ... ... ... ... ... ... ... ... ... ... ... ... ... ... ... ... ... ... ... ... ... ... ... ... ... ... ... ... ...  [1089]
H_41          ... ... ... ... ... ... ... ... ... ... ... ... ... ... ... ... ... ... ... ... ... ... ... ... ... ... ... ... ... ... ... ... ...  [1089]
H_42          ... ... ... ... ... ... ... ... ... ... ... ... ... ... ... ... ... ... ... ... ... ... ... ... ... ... ... ... ... ... ... ... ...  [1089]
H_43          ... ... ... ... ... ... ... ... ... ... ... ... ... ... ... ... ... ... ... ... ... ... ... ... ... ... ... ... ... ... ... ... ...  [1089]
H_44          ... ... ... ... ... ... ... ... ... ... ... ... ... ... ... ... ... ... ... ... ... ... ... ... ... ... ... ... ... ... ... ... ...  [1089]
H_45          ... ... ... ... ... ... ... ... ... ... ... ... ... ... ... ... ... ... ... ... ... ... ... ... ... ... ... ... ... ... ... ... ...  [1089]
H_46          ... ... ... ... ... ... ... ... ... ... ... ... ... ... ... ... ... ... ... ... ... ... ... ... ... ... ... ... ... ... ... ... ...  [1089]
H_47          ... ... ... ... ... ... ... ... ... ... ... ... ... ... ... ... ... ... ... ... ... ... ... ... ... ... ... ... ... ... ... ... ...  [1089]
H_48          ... ... ... ... ... ... ... ... ... ... ... ... ... ... ... ... ... ... ... ... ... ... ... ... ... ... ... ... ... ... ... ... ...  [1089]
H_49          ... ... ... ... ... ... ... ... ... ... ... ... ... ... ... ... ... ... ... ... ... ... ... ... ... ... ... ... ... ... ... ... ...  [1089]
H_50          ... ... ... ... ... ... ... ... ... ... ... ... ... ... ... ... ... ... ... ... ... ... ... ... ... ... ... ... ... ... ... ... ...  [1089]
H_51          ... ... ... ... ... ... ... ... ... ... ... ... ... ... ... ... ... ... ... ... ... ... ... ... ... ... ... ... ... ... ... ... ...  [1089]
H_52          ... ... ... ... ... ... ... ... ... ... ... ... ... ... ... ... ... ... ... ... ... ... ... ... ... ... ... ... ... ... ... ... ...  [1089]
H_53          ... ... ... ... ... ... ... ... ... ... ... ... ... ... ... ... ... ... ... ... ... ... ... ... ... ... ... ... ... ... ... ... ...  [1089]
H_54          ... ... ... ... ... ... ... ... ... ... ... ... ... ... ... ... ... ... ... ... ... ... ... ... ... ... ... ... ... ... ... ... ...  [1089]
H_55          ... ... ... ... ... ... ... ... ... ... ... ... ... ... ... ... ... ... ... ... ... ... ... ... ... ... ... ... ... ... ... ... ...  [1089]
H_56          ... ... ... ... ... ... ... ... ... ... ... ... ... ... ... ... ... ... ... ... ... ... ... ... ... ... ... ... ... ... ... ... ...  [1089]
H_57          ... ... ... ... ... ... ... ... ... ... ... ... ... ... ... ... ... ... ... ... ... ... ... ... ... ... ... ... ... ... ... ... ...  [1089]
H_58          ... ... ... ... ... ... ... ... ... ... ... ... ... ... ... ... ... ... ... ... ... ... ... ... ... ... ... ... ... ... ... ... ...  [1089]
```

```
H_59             ... ... ... ... ... ... ... ... ... ... ... ... ... ... ... ... ... ... ... ... ... ... ... ... ... ... ... ... ... ... ... ... ...  [1089]
H_60             ... ... ... ... ... ... ... ... ... ... ... ... ... ... ... ... ... ... ... ... ... ... ... ... ... ... ... ... ... ... ... ... ...  [1089]
H_61             ... ... ... ... ... ... ... ... ... ... ... ... ... ... ... ... ... ... ... ... ... ... ... ... ... ... ... ... ... ... ... ... ...  [1089]
H_62             ... ... ... ... ... ... ... ... ... ... ... ... ... ... ... ... ... ... ... ... ... ... ... ... ... ... ... ... ... ... ... ... ...  [1089]
H_63             ... ... ... ... ... ... ... ... ... ... ... ... ... ... ... ... ... ... ... ... ... ... ... ... ... ... ... ... ... ... ... ... ...  [1089]
H_64             ... ... ... ... ... ... ... ... ... ... ... ... ... ... ... ... ... ... ... ... ... ... ... ... ... ... ... ... ... ... ... ... ...  [1089]
H_65             ... ... ... ... ... ... ... ... ... ... ... ... ... ... ... ... ... ... ... ... ... ... ... ... ... ... ... ... ... ... ... ... ...  [1089]
H_66             ... ... ... ... ... ... ... ... ... ... ... ... ... ... ... ... ... ... ... ... ... ... ... ... ... ... ... ... ... ... ... ... ...  [1089]
H_67             ... ... ... ... ... ... ... ... ... ... ... ... ... ... ... ... ... ... ... ... ... ... ... ... ... ... ... ... ... ... ... ... ...  [1089]
H_68             ... ... ... ... ... ... ... ... ... ... ... ... ... ... ... ... ... ... ... ... ... ... ... ... ... ... ... ... ... ... ... ... ...  [1089]
H_69             ... ... ... ... ... ... ... ... ... ... ... ... ... ... ... ... ... ... ... ... ... ... ... ... ... ... ... ... ... ... ... ... ...  [1089]
H_70             ... ... ... ... ... ... ... ... ... ... ... ... ... ... ... ... ... ... ... ... ... ... ... ... ... ... ... ... ... ... ... ... ...  [1089]
Pcyn_rbsa        ... ... ... ... ... ... ... ... ... ... ... T.T ... ... ... ... A.. A.. ... ... TC. ... ... A.. ..A ... ... A.. ... ..T ..G ... ...  [1089]
Pcyn_rbsap       ... ... ... ... ... ... ... ... ... ... ... T.T ... ... ... ... A.. A.. ... ... TC. ... ... A.. ..A ... ... A.. ... ..T ..G ... ...  [1089]
Pinu_rbsa        ... ... ... ... ... ... ... ... ... ..G ... T.T ... ... ... ... A.. A.A ... ... ... ... .G. ... ... ... ..G ... ... G.. ... A.. ...  [1089]
Pfra_rbsa        ... ... ... .AC ... ... ... ... ... .GC .GA .AT ... ... C.. .C. G.T A.. ... ... ... .G. ... A.. ... ... T.. AC. ... .CA ... ... ...  [1089]

H_1              TTG CCG TTT GGC CCA ATT GTT ACA ATG ATA ATG ATA GGC TTT ATT CTA CTC TGG TCA ACG CCC TGG ACT GGT TTT ATT TTT ATA GGG TGT AGC TCT ATT  [1188]
H_2              C.. ... ... ... ... ... ... ... ... ... ... ... ... ... ... ... ... ..C ... ... ... .C. ... ... ... ... ... ... ... ... ... ... ...  [1188]
H_3              C.. ... ... ... ... ... ... ... ... ... ..T ... ... ... ... ... ... ... ... ... GG. ... ... ... ... ... ... ... ... ... ... ... ...  [1188]
H_4              C.. ... ... ... ... ... ... ... ... ... ... ... ..T ... T.. ... ... ..C .GG ..C ... .C. .T. ... ... .C. ... ... ... ... ... ... ...  [1188]
H_5              C.. ... ... ... ... ... ... ... ... ... ..T ... ... ... ... ... ... ... ... ... A.T ... ... ... ... ... ... ... ... ... ... ... ...  [1188]
H_6              C.. ... ... ... ... ... ... ... ... ... ... ... ... ... ... ... ... ... ... ... GG. ... ... ... ... ... ... ... ... ... ... ... ...  [1188]
H_7              C.. ... ... ... ... ... ... ... ... ... ... ... ... ... ... ... ... ..C ... ... ... .C. ... ... ... ... ... ... ... ... ... ... ...  [1188]
H_8              C.. ... ... ... ... ... ... ... ... ... ... ... ... ... ... ... ... ..C ... ... ... .C. ... ... ... ... ... ... ... ... ... ... ...  [1188]
H_9              C.. ... ... ... ... ... ... ... ... ... ..T ... ... ... ... ... ... ... ... ... A.T ... ... ... ... ... ... ... ... ... ... ... ...  [1188]
H_10             C.. ... ... ... ... ... ... ... ... ... ... ... ..T ... T.. ... ... ..C .GG ..C ... .C. .T. ... ... ... ... ... ... ... ... ... ...  [1188]
H_11             C.. ... ... ... ... ... ... ... ... ... ... ... ... ... ... ... ... ... ... ... ... ... ... ... ... ... ... ... ... ... ... ... ...  [1188]
H_12             C.. ... ... ... ... ... ... ... ... ... ... ... ... ... ... ... ... ... ... ... G.. ... ... ... ... ... ... ... ... ... ... ... ...  [1188]
H_13             C.. ... ... ... ... ... ... ... ... ... ... ... ..T ... T.. ... ... ..C .GG ..C ... .C. .T. ... ... .C. ... ... ... ... ... ... ...  [1188]
H_14             ... ... ... ... ... ... ... ... ... ... ..T ... ... ... ... ... ... ... ... ... GG. ... ... ... ... ... ... ... ... ... ... ... ...  [1188]
H_15             C.. ... ... ... ... ... ... ... ... ... ... ... ... ... ... ... ... ... ... ... G.T ... ... ... ... ... ... ... ... ... ... ... ...  [1188]
H_16             C.. ... ... ... ... ... ... ... ... ... ... ... ... ... ... ... ... ..C ... ... ... .C. ... ... ... ... ... ... ... ... ... ... ...  [1188]
H_17             C.. ... ... ... ... ... ... ... ... ... ... ... ..T ... T.. ... ... ..C .GG ..C ... .C. .T. ... ... .C. ... ... ... ... ... ... ...  [1188]
H_18             C.. ... ... ... ... ... ... ... ... ... ... ... ... ... ... ... ... ... ... ... ... .C. .T. ... ... ... ... ... ... ... ... ... ...  [1188]
H_19             C.. ... ... ... ... ... ... ... ... ... ... ... ... ... ... ... ... ..C ... ... ... .C. ... ... ... ... ... ... ... ... ... ... ...  [1188]
H_20             C.. ... ... ... ... ... ... ... ... ... ... ... ..T ... T.. ... ... ..C .GG ..C ... .C. .T. ... ... .C. ... ... ... ... ... ... ...  [1188]
H_21             C.. ... ... ... ... ... ... ... ... ... ... ... ... ... ... ... ... ..C .GG ..C ... .C. .T. ... ... ... ... ... ... ... ... ... ...  [1188]
H_22             C.. ... ... ... ... ... ... ... ... ... ... ... ... ... ... ... ..T ... ... ... ... ... ... ... ... ... ... ... ... ... ... ... ...  [1188]
H_23             ... ... ... ... ... ... ... ... ... ... ... ... ... ... ... ... ... ... ... ... ... ... ... ... ... ... ... ... ... ... ... ... ...  [1188]
H_24             C.. ... ... ... ... ... ... ... ... ... ... ... ... ... ... ... ... ... ... ... ... ... ... ... ... ... ... ... ... ... ... ... ...  [1188]
H_25             C.. ... ... ... ... ... ... ... ... ... ... ... ... ... ... ... ... ... ... ... ... ... ... ... ... ... ... ... ... ... ... ... ...  [1188]
H_26             C.. ... ... ... ... ... ... ... ... ... ... ... ... ... ... ... ... ... ... ... ... ... ... ... ... ... ... ... ... ... ... ... ...  [1188]
H_27             C.. ... ... ... ... ... ... ... ... ... ..T ... ... ... ... ... ... ..C ... ... ... .C. ... ... ... ... ... ... ... ... ... ... ...  [1188]
H_28             C.. ... ... ... ... ... ... ... ... ... ... ... ..T ... ... ... ... ... ... ... ... ... ... ... ... ... ... ... ... ... ... ... ...  [1188]
H_29             C.. ... ... ... ... ... ... ... ... ... ... ... ... ... ... ... ... ... ... ... ... ... ... ... ... ... ... ... ... ... ... ... ...  [1188]
H_30             C.. ... ... ... ... ... ... ... ... ... ..T ... ... ... ... ... ... ... ... ... ... ... ... ... ... ... ... ... ... ... ... ... ...  [1188]
H_31             C.. ... ... ... ... ... ... ... ... ... ... ... ... ... ... ... ... ..C ... ... ... .C. ... ... ... ... ... ... ... ... ... ... ...  [1188]
H_32             C.. ... ... ... ... ... ... ... ... ... ... ... ..T ... ... ... ... ... ... ... ... ... ... ... ... ... ... ... ... ... ... ... ...  [1188]
H_33             C.. ... ... ... ... ... ... ... ... ... ... ... ... ... ... ... ... ..C ... ... ... .C. ... ... ... ... ... ... ... ... ... ... ...  [1188]
H_34             C.. ... ... ... ... ... ... ... ... ... ... ... ... ... ... ... ... ..C ... ... ... .C. ... ... ... ... ... ... ... ... ... ... ...  [1188]
H_35             C.. ... ... ... ... ... ... ... ... ... ... ... ... ... ... ... ... ..C ... ... ... .C. ... ... ... ... ... ... ... ... ... ... ...  [1188]
H_36             C.. ... ... ... ... ... ... ... ... ... ... ... ..T ... T.. ... ... ..C .GG ..C ... .C. .T. ... ... .C. ... ... ... ... ... ... ...  [1188]
H_37             C.. ... ... ... ... ... ... ... ... ... ... ... ... ... ... ... ..T ... ... ... ... ... ... ... ... ... ... ... ... ... ... ... ...  [1188]
H_38             C.. ... ... ... ... ... ... ... ... ... ... ... ..T ... T.. ... ... ..C .GG ..C ... .C. .T. ... ... .C. ... ... ... ... ... ... ...  [1188]
H_39             C.. ... ... ... ... ... ... ... ... ... ..T ... ... ... ... ... ... ... ... ... A.T ... ... ... ... ... ... ... ... ... ... ... ...  [1188]
```

```
H_40         C.. ... ... ... ... ... ... ... ... ... ... ... ... ... ... ... ... ..C ... ... ... .C. ... ... ... ... ... ... ... ... ... ... ...  [1188]
H_41         C.. ... ... ... ... ... ... ... ... ... ..T ... ... ... ... ... ... ... ... ... ... ... ... ... ... ... ... ... ... ... ... ... ...  [1188]
H_42         C.. ... ... ... ... ... ... ... ... ... ..T ... ... ... ... ... ... ... ... ... ... ... ... ... ... ... ... ... ... ... ... ... ...  [1188]
H_43         C.. ... ... ... ... ... ... ... ... ... ... ... ... ... ... ... ... ..C ... ... ... .C. ... ... ... ... ... ... ... ... ... ... ...  [1188]
H_44         C.. ... ... ... ... ... ... ... ... ... ... ... ... ... ... ... ... ..C ... ... ... .C. ... ... ... ... ... ... ... ... ... ... ...  [1188]
H_45         C.. ... ... ... ... ... ... ... ... ... ... ... ..T ... T.. ... ... ..C ... ..C ... .C. .T. ... ... ... ... ... ... ... ... ... ...  [1188]
H_46         C.. ... ... ... ... ... ... ... ... ... ..T ... ... ... ... ... ... ... ... ... A.T ... ... ... ... ... ... ... ... ... ... ... ...  [1188]
H_47         C.. ... ... ... ... ... ... ... ... ... ..T ... ... ... ... ... ... ... ... ... GG. ... ... ... ... ... ... ... ... ... ... ... ...  [1188]
H_48         C.. ... ... ... ... ... ... ... ... ... ... ... ..T ... T.. ... ... ..C .GG ..C ... .C. .T. ... ... .C. ... ... ... ... ... ... ...  [1188]
H_49         C.. ... ... ... ... ... ... ... ... ... ... ... ... ... ... ... ... ... ... ... ... ... .T. ... ... ... ... ... ... ... ... ... ...  [1188]
H_50         C.. ... ... ... ... ... ... ... ... ... ... ... ... ... ... ... ... ... ... ... ... .C. .T. ... ... ... ... ... ... ... ... ... ...  [1188]
H_51         C.. ... ... ... ... ... ... ... ... ... ..T ... ... ... ... ... ... ... ... ... ... ... ... ... ... ... ... ... ... ... ... ... ...  [1188]
H_52         C.. ... ... ... ... ... ... ... ... ... ... ... ..T ... T.. ... ... ..C .GG ..C ... .C. .T. ... ... .C. ... ... ... ... ... ... ...  [1188]
H_53         C.. ... ... ... ... ... ... ... ... ... ..T ... ... ... ... ... ... ... ... ... ... ... ... ... ... ... ... ... ... ... ... ... ...  [1188]
H_54         C.. ... ... ... ... ... ... ... ... ... ... ... ... ... ... ... ... ..C .GG ..C ... .C. .T. ... ... .C. ... ... ... ... ... ... ...  [1188]
H_55         C.. ... ... ... ... ... ... ... ... ... ..T ... ... ... ... ... ... ... ... ... ... ... ... ... ... ... ... ... ... ... ... ... ...  [1188]
H_56         C.. ... ... ... ... ... ... ... ... ... ... ... ... ... ... ... ... ..C .GG ..C ... .C. .T. ... ... .C. ... ... ... ... ... ... ...  [1188]
H_57         C.. ... ... ... ... ... ... ... ... ... ... ... ..T ... ... ... ... ..C ... ... ... .C. ... ... ... ... ... ... ... ... ... ... ...  [1188]
H_58         C.. ... ... ... ... ... ... ... ... ... ... ... ... ... ... ... ... ..C ... ... ... .C. .T. ... ... ... ... ... ... ... ... ... ...  [1188]
H_59         C.. ... ... ... ... ... ... ... ... ... ... ... ... ... ... ... ... ... ... ... ... ... ... ... ... ... ... ... ... ... ... ... ...  [1188]
H_60         C.. ... ... ... ... ... ... ... ... ... ... ... ... ... ... ... ... ... ... ... GG. ... ... ... ... ... ... ... ... ... ... ... ...  [1188]
H_61         C.. ... ... ... ... ... ... ... ... ... ... ... ..T ... T.. ... ... ..C .GG ..C ... .C. .T. ... ... .C. ... ... ... ... ... ... ...  [1188]
H_62         C.. ... ... ... ... ... ... ... ... ... ... ... ..T ... ... ... ... ..C ..G ..C ... .C. .T. ... ... .C. ... ... ... ... ... ... ...  [1188]
H_63         C.. ... ... ... ... ... ... ... ... ... ... ... ... ... ... ... ... ..C .GG ..C ... .C. .T. ... ... .C. ... ... ... ... ... ... ...  [1188]
H_64         C.. ... ... ... ... ... ... ... ... ... ... ... ... ... ... ... ... ..C ... ... ... .C. .T. ... ... ... ... ... ... ... ... ... ...  [1188]
H_65         C.. ... ... ... ... ... ... ... ... ... ... ... ..T ... ... ... ... ... ... ... ... ... ... ... ... ... ... ... ... ... ... ... ...  [1188]
H_66         C.. ... ... ... ... ... ... ... ... ... ... ... ... ... ... ... ... ..C .GG ..C ... .C. .T. ... ... .C. ... ... ... ... ... ... ...  [1188]
H_67         C.. ... ... ... ... ... ... ... ... ... ... ... ..T ... ... ... ... ... ... ... ... ... ... ... ... ... ... ... ... ... ... ... ...  [1188]
H_68         C.. ... ... ... ... ... ... ... ... ... ... ... ..T ... ... ... ... ... ... ... ... ... ... ... ... ... ... ... ... ... ... ... ...  [1188]
H_69         C.. ... ... ... ... ... ... ... ... ... ..T ... ... ... ... ... ..T ... ... ... ... ... ... ... ... ... ... ... ... ... ... .T. ...  [1188]
H_70         C.. ... ... ... ... ... ... ... ... ... ... ... ... ... ... ... ... ... ... ... ... ... ... ... ... ... ... ... ... ... ... ... ...  [1188]
Pcyn_rbsa    C.. ... ... .T. ... ... ... G.. G.. ... ..A ..T TTA A.G ... ..T ..A CTC A.G ..C ... AA. .T. ... ..G .C. ... T.G TTA ... ... C.. GC.  [1188]
Pcyn_rbsap   C.. ... ... .T. ... ... ... G.. G.. ... ..A ..T TTA A.G ... ..T ..A CTC A.G ..C ... AA. .T. ... ..G .C. ... T.G TTA ... ... C.. GC.  [1188]
Pinu_rbsa    C.. ... ... .C. ... ... ... ... T.. ... ..T ... .T. C.. GC. ... T.T ..C .TC .AA TG. CTT TA. T.. .G. C.. ..G G.G --- ... ... .T. GC.  [1188]
Pfra_rbsa    ... T.. ... ... ... .C. ... ... G.. T.. C.T ... AT. C.. ... .A. ... --- ..T .AT ... ... .T. ... .GG CCA C.. T.G .T. ... ... A.. G..  [1188]

H_1          ATA TTA CCA GCT TTG TTA TAC GCG ATG CAT TCA AAC AAA AAA CAA TTC GAA CGT GTA TTT GGA AAA GGG AGA AAA AAG CCA ATG AAG GTA AAA AAA GGA  [1287]
H_2          ... ... ... ... ... ... ... ... ... ... ... ... ... ... ... ... ... ... ... ... ... ... ... ... ... ... ... ... ... ... ... ... ...  [1287]
H_3          ... ... ... ... ... ... ... ... ... T.. ... ... ... ... ... ... ... ... ... ... ... ... ... ... ... ... ... ... ... ... ... ... ...  [1287]
H_4          ... ... ... ... ... ... ... ... ... ... ... ... ... ... ... ... ... ... ... ... ... ... ... ... ... ... ... ... ... ... ... ... ...  [1287]
H_5          ... ... ... ... ... ... ... ... ... ... ... ... ... ... ... ... ... ... ... ... ... ... ... ... ... ... ... ... ... ... ... ... ...  [1287]
H_6          ... ... ... ... ... ... ... ... ... T.. ... ... ... ... ... ... ... ... ... ... ... ... ... ... ... ... ... ... ... ... ... ... ...  [1287]
H_7          ... ... ... ... ... ... ... ... ... ... ... ... ... ... ... ... ... ... ... ... ... ... ... ... ... ... ... ... ... ... ... ... ...  [1287]
H_8          ... ... ... ... ... ... ... ... ... ... ... ... ... ... ... ... ... ... ... ... ... ... ... ... ... ... ... ... ... ... ... ... ...  [1287]
H_9          ... ... ... ... ... ... ... ... ... ... ... ... ... ... ... ... ... ... ... ... ... ... ... ... ... ... ... ... ... ... ... ... ...  [1287]
H_10         ... ... ... ... ... ... ... ... ... ... ... ... ... ... ... ... ... ... ... ... ... ... ... ... ... ... ... ... ... ... ... ... ...  [1287]
H_11         ... ... ... ... ... ... ... ... ... ... ... ... ... ... ... ... ... ... ... ... ... ... ... ... ... ... ... ... ... ... ... ... ...  [1287]
H_12         ... ... ... ... ... ... ... ... ... ... ... ... ... ... ... ... ... ... ... ... ... ... ... ... ... ... ... ... ... ... ... ... ...  [1287]
H_13         ... ... ... ... ... ... ... ... ... T.. ... ... ... ... ... ... ... ... ... ... ... ... ... ... ... ... ... ... ... ... ... ... ...  [1287]
H_14         ... ... ... ... ... ... ... ... ... T.. ... ... ... ... ... ... ... ... ... ... ... ... ... ... ... ... ... ... ... ... ... ... ...  [1287]
H_15         ... ... ... ... ... ... ... ... ... ... ... ... ... ... ... ... ... ... ... ... ... ... ... ... ... ... ... ... ... ... ... ... ...  [1287]
H_16         ... ... ... ... ... ... ... ... ... ... ... ... ... ... ... ... ... ... ... ... ... ... ... ... ... ... ... ... ... ... ... ... ...  [1287]
H_17         ... ... ... ... ... ... ... ... ... ... ... ... ... ... ... ... ... ... ... ... ... ... ... ... ... ... ... ... ... ... ... ... ...  [1287]
H_18         ... ... ... ... ... ... ... ... ... ... ... ... ... ... ... ... ... ... ... ... ... ... ... ... ... ... ... ... ... ... ... ... ...  [1287]
H_19         ... ... ... ... ... ... ... ... ... T.. ... ... ... ... ... ... ... ... ... ... ... ... ... ... ... ... ... ... ... ... ... ... ...  [1287]
H_20         ... ... ... ... ... ... ... ... ... T.. ... ... ... ... ... ... ... ... ... ... ... ... ... ... ... ... ... ... ... ... ... ... ...  [1287]
H_21         ... ... ... ... ... A.. ... ... ... ... ... ... ... ... ... ... ... ... ... ... ... ... ... ... ... ... ... ... ... ... ... ... ...  [1287]
H_22         ... ... ... ... ... ... ... ... ... ... ... ... ... ... ... ... ... ... ... ... ... ... ... ... ... ... ... ... ... ... ... ... ...  [1287]
H_23         ... ... ... ... ... ... ... ... ... ... ... ... ... ... ... ... ... ... ... ... ... ... ... ... ... ... ... ... ... ... ... ... ...  [1287]
H_24         ... ... ... ... ... ... ... ... ... ... ... ... ... ... ... ... ... ... ... ... ... ... ... G.. ... ... ... ... ... ... ... ... ...  [1287]
```

```
H_25        ... ... ... ... ... ... ... ... ... ... ... ... ... ... ... ... ... ... ... ... ... ... ... ... ... ... ... ... ... ... ... ... ... ...  [1287]
H_26        G.. ... ... ... ... ... ... ... ... ... ... ... ... ... ... ... ... ... ... ... ... ... ... G.. ... ... ... ... ... ... ... ... ... ...  [1287]
H_27        ... ... ... ... ... ... ... ... ... ... ... ... ... ... ... ... ... ... ... ... ... ... ... ... ... ... ... ... ... ... ... ... ... ...  [1287]
H_28        ... ... ... ... ... ... ... ... ... ... ... ... ... ... ... ... ... ... ... ... ... ... ... ... ... ... ... ... ... ... ... ... ... ...  [1287]
H_29        ... ... ... ... ... ... ... ... ... ... ... ... ... ... ... ... ... ... ... ... ... ... ... ... ... ... ... ... ... ... ... ... ... ...  [1287]
H_30        ... ... ... ... ... ... ... ... ... ... ... ... ... ... ... ... ... ... ... ... ... ... ... ... ... ... ... ... ... ... ... ... ... ...  [1287]
H_31        ... ... ... ... ... ... ... ... ... ... ... ... ... ... ... ... ... ... ... ... ... ... ... ... ... ... ... ... ... ... ... ... ... ...  [1287]
H_32        ... ... ... ... ... ... ... ... ... ... ... ... ... ... ... ... ... ... ... ... ... ... ... ... ... ... ... ... ... ... ... ... ... ...  [1287]
H_33        ... ... ... ... ... ... ... ... ... ... ... ... ... ... ... ... ... ... ... ... ... ... ... ... ... ... ... ... ... ... ... ... ... ...  [1287]
H_34        ... ... ... ... ... ... ... ... ... ... ... ... ... ... ... ... ... ... ... ... ... ... ... ... ... ... ... ... ... ... ... ... ... ...  [1287]
H_35        ... ... ... ... ... ... ... ... ... ... ... ... ... ... ... ... ... ... ... ... ... ... ... ... ... ... ... ... ... ... ... ... ... ...  [1287]
H_36        ... ... ... ... ... ... ... ... ... ... ... ... ... ... ... ... ... ... ... ... ... ... ... ... ... ... ... ... ... ... ... ... ... ...  [1287]
H_37        ... ... ... ... ... ... ... ... ... ... ... ... ... ... ... ... ... ... ... ... ... ... ... ... ... ... ... ... ... ... ... ... ... ...  [1287]
H_38        ... ... ... ... ... ... ... ... ... ... ... ... ... ... ... ... ... ... ... ... ... ... ... ... ... ... ... ... ... ... ... ... ... ...  [1287]
H_39        ... ... ... ... ... ... ... ... ... ... ... ... ... ... ... ... ... ... ... ... ... ... ... ... ... ... ... ... ... ... ... ... ... ...  [1287]
H_40        ... ... ... ... ... ... ... ... ... ... ... ... ... ... ... ... ... ... ... ... ... ... ... ... ... ... ... ... ... ... ... ... ... ...  [1287]
H_41        ... ... ... ... ... ... ... ... ... ... ... ... ... ... ... ... ... ... ... ... ... ... ... ... ... ... ... ... ... ... ... ... ... ...  [1287]
H_42        ... ... ... ... ... A.. ... ... ... ... ... ... ... ... ... ... ... ... ... ... ... ... ... ... ... ... ... ... ... ... ... ... ... ...  [1287]
H_43        ... ... ... ... ... ... ... ... ... ... ... ... ... ... ... ... ... ... ... ... ... ... ... ... ... ... ... ... ... ... ... ... ... ...  [1287]
H_44        ... ... ... ... ... ... ... ... ... ... ... ... ... ... ... ... ... ... ... ... ... ... ... ... ... ... ... ... ... ... ... ... ... ...  [1287]
H_45        ... ... ... ... ... ... ... ... ... ... ... ... ... ... ... ... ... ... ... ... ... ... ... ... ... ... ... ... ... ... ... ... ... ...  [1287]
H_46        ... ... ... ... ... ... ... ... ... ... ... ... ... ... ... ... ... ... ... ... ... ... ... ... ... ... ... ... ... ... ... ... ... ...  [1287]
H_47        ... ... ... ... ... ... ... ... ... T.. ... ... ... ... ... ... ... ... ... ... ... ... ... ... ... ... ... ... ... ... ... ... ... ...  [1287]
H_48        ... ... ... ... ... ... ... ... ... ... ... ... ... ... ... ... ... ... ... ... ... ... ... ... ... ... ... ... ... ... ... ... ... ...  [1287]
H_49        ... ... ... ... ... ... ... ... ... ... ... ... ... ... ... ... ... ... ... ... ... ... ... ... ... ... ... ... ... ... ... ... ... ...  [1287]
H_50        ... ... ... ... ... ... ... ... ... ... ... ... ... ... ... ... ... ... ... ... ... ... ... ... ... ... ... ... ... ... ... ... ... ...  [1287]
H_51        ... ... ... ... ... ... ... ... ... ... ... ... ... ... ... ... ... ... ... ... ... ... ... ... ... ... ... ... ... ... ... ... ... ...  [1287]
H_52        ... ... ... ... ... ... ... ... ... T.. ... ... ... ... ... ... ... ... ... ... ... ... ... ... ... ... ... ... ... ... ... ... ... ...  [1287]
H_53        ... ... ... ... ... A.. ... ... ... ... ... ... ... ... ... ... ... ... ... ... ... ... ... ... ... ... ... ... ... ... ... ... ... ...  [1287]
H_54        ... ... ... ... ... ... ... ... ... T.. ... ... ... ... ... ... ... ... ... ... ... ... ... ... ... ... ... ... ... ... ... ... ... ...  [1287]
H_55        ... ... ... ... ... A.. ... ... ... ... ... ... ... ... ... ... ... ... ... ... ... ... ... ... ... ... ... ... ... ... ... ... ... ...  [1287]
H_56        ... ... ... ... ... ... ... ... ... T.. ... ... ... ... ... ... ... ... ... ... ... ... ... ... ... ... ... ... ... ... ... ... ... ...  [1287]
H_57        ... ... ... ... ... ... ... ... ... ... ... ... ... ... ... ... ... ... ... ... ... ... ... ... ... ... ... ... ... ... ... ... ... ...  [1287]
H_58        ... ... ... ... ... ... ... ... ... ... ... ... ... ... ... ... ... ... ... ... ... ... ... ... ... ... ... ... ... ... ... ... ... ...  [1287]
H_59        ... ... ... ... ... ... ... ... ... ... ... ... ... ... ... ... ... ... ... ... ... ... ... ... ... ... ... ... ... ... ... ... ... ...  [1287]
H_60        ... ... ... ... ... ... ... ... ... ... ... ... ... ... ... ... ... ... ... ... ... ... ... ... ... ... ... ... ... ... ... ... ... ...  [1287]
H_61        ... ... ... ... ... ... ... ... ... T.. ... ... ... ... ... ... ... ... ... ... ... ... ... ... ... ... ... ... ... ... ... ... ... ...  [1287]
H_62        ... ... ... ... ... ... ... ... ... T.. ... ... ... ... ... ... ... ... ... ... ... ... ... ... ... ... ... ... ... ... ... ... ... ...  [1287]
H_63        ... ... ... ... ... ... ... ... ... ... ... ... ... ... ... ... ... ... ... ... ... ... ... ... ... ... ... ... ... ... ... ... ... ...  [1287]
H_64        ... ... ... ... ... ... ... ... ... ... ... ... ... ... ... ... ... ... ... ... ... ... ... ... ... ... ... ... ... ... ... ... ... ...  [1287]
H_65        ... ... ... ... ... ... ... ... ... ... ... ... ... ... ... ... ... ... ... ... ... ... ... ... ... ... ... ... ... ... ... ... ... ...  [1287]
H_66        ... ... ... ... ... ... ... ... ... T.. ... ... ... ... ... ... ... ... ... ... ... ... ... ... ... ... ... ... ... ... ... ... ... ...  [1287]
H_67        ... ... ... ... ... ... ... ... ... ... ... ... ... ... ... ... ... ... ... ... ... ... ... ... ... ... ... ... ... ... ... ... ... ...  [1287]
H_68        ... ... ... ... ... ... ... ... ... ... ... ... ... ... ... ... ... ... ... ... ... ... ... ... ... ... ... ... ... ... ... ... ... ...  [1287]
H_69        ... ... ... ... ... ... ... ... ... ... ... ... ... ... ... ... ... ... ... ... ... ... ... ... ... ... ... ... ... ... ... ... ... ...  [1287]
H_70        ... ... ... ... ... ... ... ... ... ... ... ... ... ... ... ... ... ... ... ... ... ... ... ... ... ... ... ... ... ... ... ... ... ...  [1287]
Pcyn_rbsa   G.T A.. G.. ... ..T A.. ... .T. ... T.. ... ... .G. ... ... ... A.. ... AC. ... ... .C. A.. ... --- --- --- --- --- --- --- --- --- ---  [1287]
Pcyn_rbsap  G.T A.. G.. ... ..T A.. ... .T. ... T.. ... ... .G. ... ... ... A.. ... AC. ... ... .C. A.. ... --- --- --- --- --- --- --- --- --- ---  [1287]
Pinu_rbsa   ..G A.. G.. ... .G. AA. ... CG. .AA .G. .T. ... .G. ... ... ... ... ... ... ... ... ... AA. ... .G. ... .T. .AT .GA .C. G.. ... A.. ...  [1287]
Pfra_rbsa   C.G C.. T.. ... ... AC. ... ... ... .G. ... ..T ... .C. ... .G. ... ... A.. ... C.. ... T.. ... ... ... .TG .AT ..A ... ... ... ... ...  [1287]

H_1         GGC GGG AAA ATA TCT AGA TTT TTT AAA GAA GTA GAC AAA TTC TTG TTT GAC GAT TTG GAT GGT TAT  [1353]
H_2         ... ... ... ... ... ... ... ... ... ... ... ... ... ... ... ... ... ... ... ... ... ...  [1353]
H_3         ... ... ... ... ... ... ... ... ... ... ... ... ... ... ... ... ... ... ... ... ... ...  [1353]
H_4         ... ... ... ... ... ... ... ... ... ... ... ... ... ... ... ... ... ... ... ... ... ...  [1353]
H_5         ... ... ... ... ... ... ... ... ... ... ... ... ... ... ... ... ... ... ... ... ... ...  [1353]
```

```
H_6          ... ... ... ... ... ... ... ... ... ... ... ... ... ... ... ... ... ... ... ... ... ...  [1353]
H_7          ... ... ... ... ... ... ... ... ... ... ... ... ... ... ... ... ... ... ... ... ... ...  [1353]
H_8          ... ... ... ... ... ... ... ... ... ... ... ... ... ... ... ... ... ... ... ... ... ...  [1353]
H_9          ... ... ... ... ... ... ... ... ... ... ... ... ... ... ... ... ... ... ... ... ... ...  [1353]
H_10         ... ... ... ... ... ... ... ... ... ... ... ... ... ... ... ... ... ... ... ... ... ...  [1353]
H_11         ... ... ... ... ... ... ... ... ... ... ... ... ... ... ... ... ... ... ... ... ... ...  [1353]
H_12         ... ... ... ... ... ... ... ... ... ... ... ... ... ... ... ... ... ... ... ... ... ...  [1353]
H_13         ... ... ... ... ... ... ... ... ... ... ... ... ... ... ... ... ... ... ... ... ... ...  [1353]
H_14         ... ... ... ... ... ... ... ... ... ... ... ... ... ... ... ... ... ... ... ... ... ...  [1353]
H_15         ... ... ... ... ... ... ... ... ... ... ... ... ... ... ... ... ... ... ... ... ... ...  [1353]
H_16         ... ... ... ... ... ... ... ... ... ... ... ... ... ... ... ... ... ... ... ... ... ...  [1353]
H_17         ... ... ... ... ... ... ... ... ... ... ... ... ... ... ... ... ... ... ... ... ... ...  [1353]
H_18         ... ... ... ... ... ... ... ... ... ... ... ... ... ... ... ... ... ... ... ... ... ...  [1353]
H_19         ... ... ... ... ... ... ... ... ... ... ... ... ... ... ... ... ... ... ... ... ... ...  [1353]
H_20         ... ... ... ... ... ... ... ... ... ... ... ... ... ... ... ... ... ... ... ... ... ...  [1353]
H_21         ... ... ... ... ... ... ... ... ... ... ... ... ... ... ... ... ... ... ... ... ... ...  [1353]
H_22         ... ... ... ... ... ... ... ... ... ... ... ... ... ... ... ... ... ... ... ... ... ...  [1353]
H_23         ... ... ... ... ... ... ... ... ... ... ... ... ... ... ... ... ... ... ... ... ... ...  [1353]
H_24         ... ... ... ... ... ... ... ... ... ... ... ... ... ... ... ... ... ... ... ... ... ...  [1353]
H_25         ... ... ... ... ... ... ... ... ... ... ... ... ... ... ... ... ... ... ... ... ... ...  [1353]
H_26         ... ... ... ... ... ... ... ... ... ... ... ... ... ... ... ... ... ... ... ... ... ...  [1353]
H_27         ... ... ... ... ... ... ... ... ... ... ... ... ... ... ... ... ... ... ... ... ... ...  [1353]
H_28         ... ... ... ... ... ... ... ... ... ... ... ... ... ... ... ... ... ... ... ... ... ...  [1353]
H_29         ... ... ... ... ... ... ... ... ... ... ... ... ... ... ... ... ... ... ... ... ... ...  [1353]
H_30         ... ... ... ... ... ... ... ... ... ... ... ... ... ... ... ... ... ... ... ... ... ...  [1353]
H_31         ... ... ... ... ... ... ... ... ... ... ... ... ... ... ... ... ... ... ... ... ... ...  [1353]
H_32         ... ... ... ... ... ... ... ... ... ... ... ... ... --- --- --- --- --- --- --- --- ---  [1353]
H_33         ... ... ... ... ... ... ... ... ... ... ... ... ... --- --- --- --- --- --- --- --- ---  [1353]
H_34         ... ... ... ... ... ... ... ... ... ... ... ... ... --- --- --- --- --- --- --- --- ---  [1353]
H_35         ... ... ... ... ... ... ... ... ... ... ... ... ... --- --- --- --- --- --- --- --- ---  [1353]
H_36         ... ... ... ... ... ... ... ... ... ... ... ... ... --- --- --- --- --- --- --- --- ---  [1353]
H_37         ... ... ... ... ... ... ... ... ... ... ... ... ... --- --- --- --- --- --- --- --- ---  [1353]
H_38         ... ... ... ... ... ... ... ... ... ... ... ... ... --- --- --- --- --- --- --- --- ---  [1353]
H_39         ... ... ... ... ... ... ... ... ... ... ... ... ... --- --- --- --- --- --- --- --- ---  [1353]
H_40         ... ... ... ... ... ... ... ... ... ... ... ... ... --- --- --- --- --- --- --- --- ---  [1353]
H_41         ... ... ... ... ... ... ... ... ... ... ... ... ... --- --- --- --- --- --- --- --- ---  [1353]
H_42         ... ... ... ... ... ... ... ... ... ... ... ... ... --- --- --- --- --- --- --- --- ---  [1353]
H_43         ... ... ... ... ... ... ... ... ... ... ... ... ... --- --- --- --- --- --- --- --- ---  [1353]
H_44         ... ... ... ... ... ... ... ... ... ... ... ... ... --- --- --- --- --- --- --- --- ---  [1353]
H_45         ... ... ... ... ... ... ... ... ... ... ... ... ... --- --- --- --- --- --- --- --- ---  [1353]
H_46         ... ... ... ... ... ... ... ... ... ... ... ... ... --- --- --- --- --- --- --- --- ---  [1353]
H_47         ... ... ... ... ... ... ... ... ... ... ... ... ... --- --- --- --- --- --- --- --- ---  [1353]
H_48         ... ... ... ... ... ... ... ... ... ... ... ... ... --- --- --- --- --- --- --- --- ---  [1353]
H_49         ... ... ... ... ... ... ... ... ... ... ... ... ... --- --- --- --- --- --- --- --- ---  [1353]
H_50         ... ... ... ... ... ... ... ... ... ... ... ... ... --- --- --- --- --- --- --- --- ---  [1353]
H_51         ... ... ... ... ... ... ... ... ... ... ... ... ... --- --- --- --- --- --- --- --- ---  [1353]
H_52         ... ... ... ... ... ... ... ... ..G A.. ... ... ... --- --- --- --- --- --- --- --- ---  [1353]
H_53         ... ... ... ... ... ... ... ... ... ... ... ... ... --- --- --- --- --- --- --- --- ---  [1353]
H_54         ... ... ... ... ... ... ... ... ..G A.. ... ... ... --- --- --- --- --- --- --- --- ---  [1353]
H_55         ... ... ... ... ... ... ... ... ..G A.. ... ... ... --- --- --- --- --- --- --- --- ---  [1353]
H_56         ... ... ... ... ... ... ... ... ... ... ... ... ... --- --- --- --- --- --- --- --- ---  [1353]
H_57         ... ... ... ... ... ... ... ... ... ... ... ... ... --- --- --- --- --- --- --- --- ---  [1353]
H_58         ... ... ... ... ... ... ... ... ... ... ... ... ... --- --- --- --- --- --- --- --- ---  [1353]
H_59         ... ... ... ... ... ... ... ... ... ... ... ... ... --- --- --- --- --- --- --- --- ---  [1353]
H_60         ... ... ... ... ... ... ... ... ... ... ... ... ... --- --- --- --- --- --- --- --- ---  [1353]
H_61         ... ... ... ... ... ... ... ... ... ... ... ... ... --- --- --- --- --- --- --- --- ---  [1353]
H_62         ... ... ... ... ... ... ... ... ... ... ... ... ... --- --- --- --- --- --- --- --- ---  [1353]
H_63         ... ... ... ... ... ... ... ... ... ... ... ... ... --- --- --- --- --- --- --- --- ---  [1353]
H_64         ... ... ... ... ... ... ... ... ... ... ... ... ... --- --- --- --- --- --- --- --- ---  [1353]
H_65         ... ... ... ... ... ... ... ... ... ... ... ... ... --- --- --- --- --- --- --- --- ---  [1353]
```

```
H_66          ... ... ... ... ... ... ... ... ... ... ... ... ... --- --- --- --- --- --- --- --- ---  [1353]
H_67          ... ... ... ... ... ... ... ... ... ... ... ... ... --- --- --- --- --- --- --- --- ---  [1353]
H_68          ... ... ... ... ... ... ... ... ... ... ... ... ... --- --- --- --- --- --- --- --- ---  [1353]
H_69          ... ... ... ... ... ... ... ... ... ... ... ... ... --- --- --- --- --- --- --- --- ---  [1353]
H_70          ... ... ... ... ... ... ... ... ... ... ... ... ... --- --- --- --- --- --- --- --- ---  [1353]
Pcyn_rbsa     --- --- --- --- --- --- --- --- --- --- --- --- --- --- --- --- --- --- --- --- --- ---  [1353]
Pcyn_rbsap    --- --- --- --- --- --- --- --- --- --- --- --- --- --- --- --- --- --- --- --- --- ---  [1353]
Pinu_rbsa     ..T ... ... ... ... ... ... C.. ..G ..G .G. C.. .G. G.T ... ... ... T.. ... ... CTC .T.  [1353]
Pfra_rbsa     ..T T.. .C. ... CG. ... ... C.. ..C A.G ... ... ... ... ... ... ... .T. ... ... ..C .T.  [1353]
```

***rbsa*** **and paralogue sequences and alignment**s **A**. Aligned and manually edited sequences in Fasta format. **B**. Alignment of the *rbsa* found in monkey-malaria parasites. **C**. Alignment of the 4,000 bp *P*. *vivax* duplicated fragment. **D**. Alignment of *P*. *cynomolgi* duplicated fragment. **E**. Alignment of the *pvrbsa* haplotypes found worldwide. Star and stop codons are displayed in red within C and D alignments. The putative PRK15370 domain is shown blue whilst peptides that inhibit the PvRBSA reticulocyte binding ability are displayed in green within the E alignment.
