# Supplementary material for: On the Evolution and Function of Plasmodium vivax Reticulocyte Binding Surface Antigen (pvrbsa)
Source: Front Genet. 2018 Sep 10;9:372. doi: 10.3389/fgene.2018.00372 (PMC6139305; doi:10.3389/fgene.2018.00372)
Supplement: Supplementary file 5 [file Data_Sheet_2.pdf]

Supplementary Data Sheet 2. On the evolution and function of *Plasmodium vivax* reticulocyte binding surface antigen (*pvrbsa*)

Paola Andrea Camargo-Ayala, Diego Garzón-Ospina, Darwin Andrés Moreno-Pérez, Laura Alejandra Ricaurte-Contreras, Oscar Noya, Manuel A. Patarroyo*

*** Correspondence:** mapatarr.fidic@gmail.com

Recombination tests

	n	Population	Zns	ZZ	RM
**Full length gene**	135	**Colombia isolates**	0.1013	**0.3268 (< 0.01)**	8
	41	**Amazonas**	0.1737	**0.3346 (< 0.01)**	2
	37	**Chocó**	0.1525	**0.2875 (< 0.01)**	6
	39	**Córdoba**	0.203	**0.3190 (< 0.01)**	5
	18	**Meta**	**0.2778 (< 0.05)**	0.2047	3
	50	**Venezuela**	**0.1512 (< 0.05)**	**0.2627 (< 0.01)**	10
	29	**Bolívar**	0.1820	**0.2624 (< 0.05)**	7
	19	**Coastal area**	0.1795	**0.2427 (< 0.05)**	6

Recombination breakpoints were identified by GARD method at the nucleotides 618 and 1058 (p = 0.0004, nucleotide number base in the Sal-I sequence).

Lineal regression plots for population and subpopulations

Worldwide

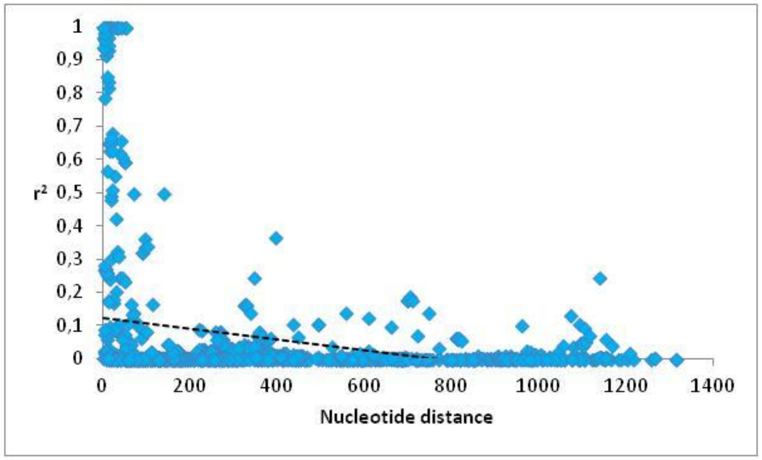

Colombia

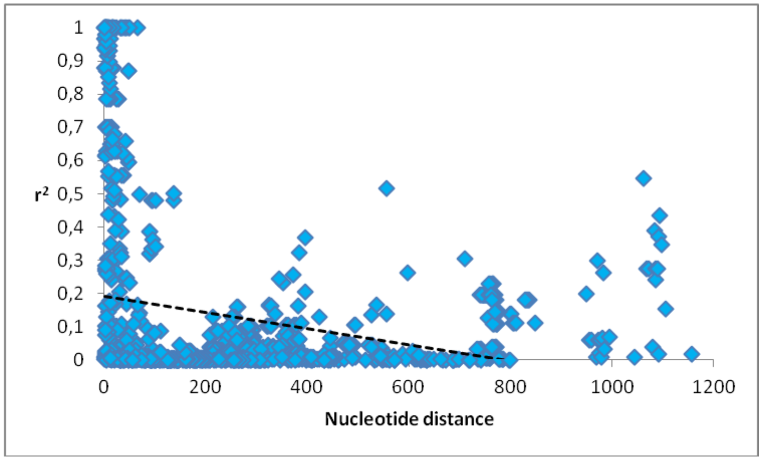

Venezuela

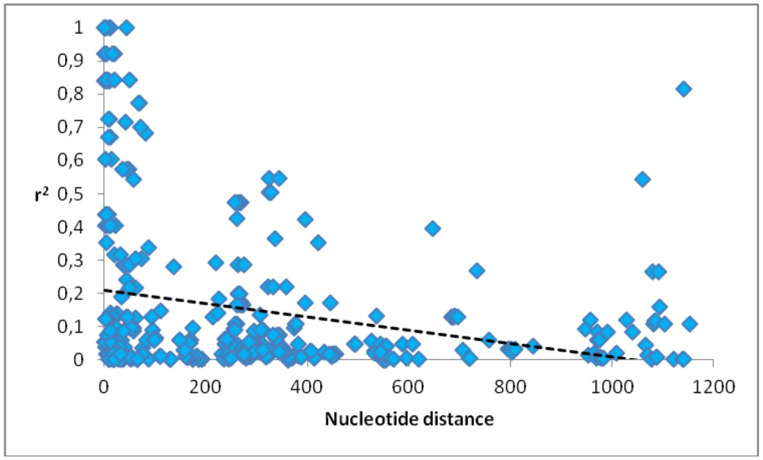

Amazonas - Colombia

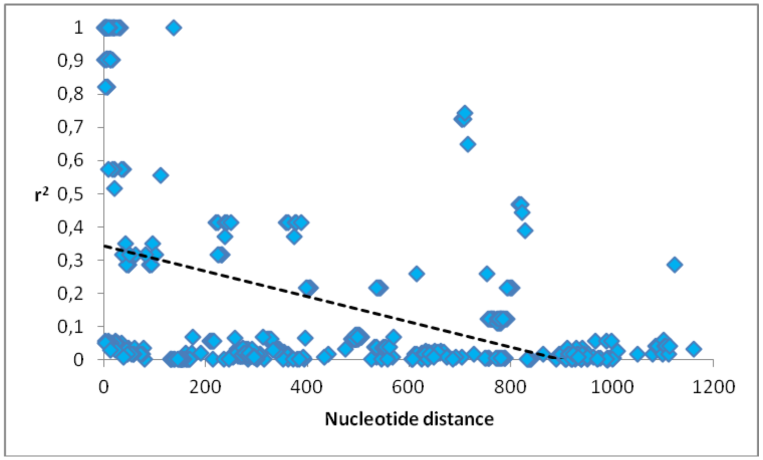

Chocó – Colombia

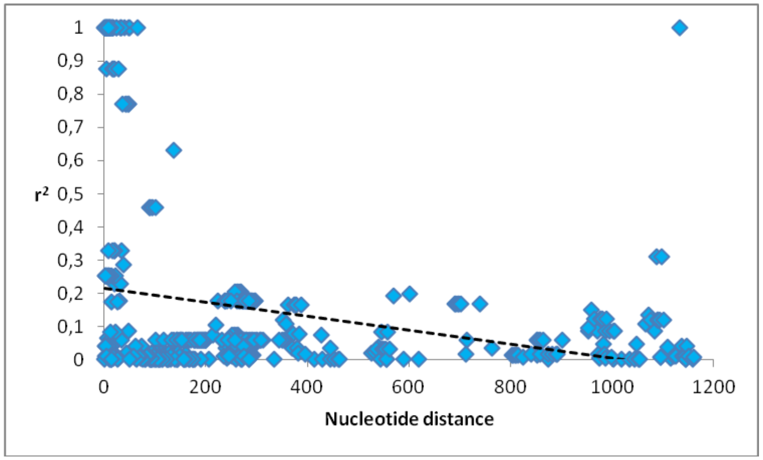

Córdoba –Colombia

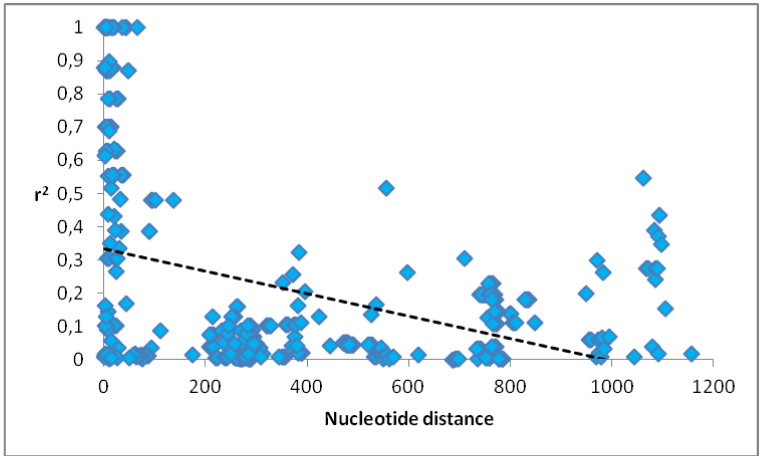

Bolívar - Venezuela

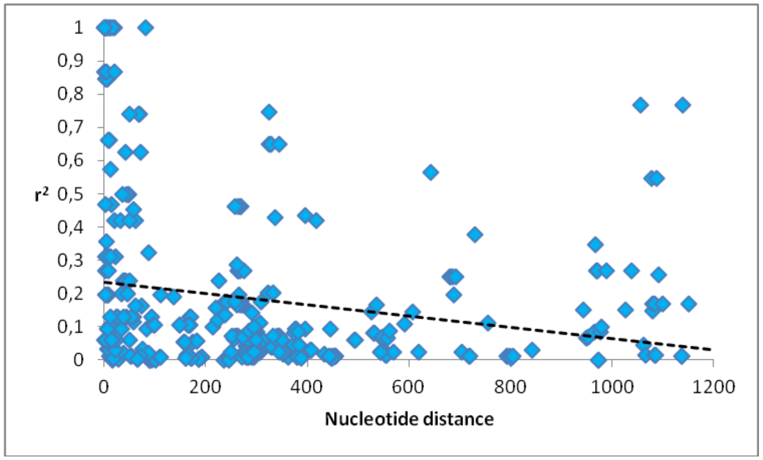

Venezuela's coastal area

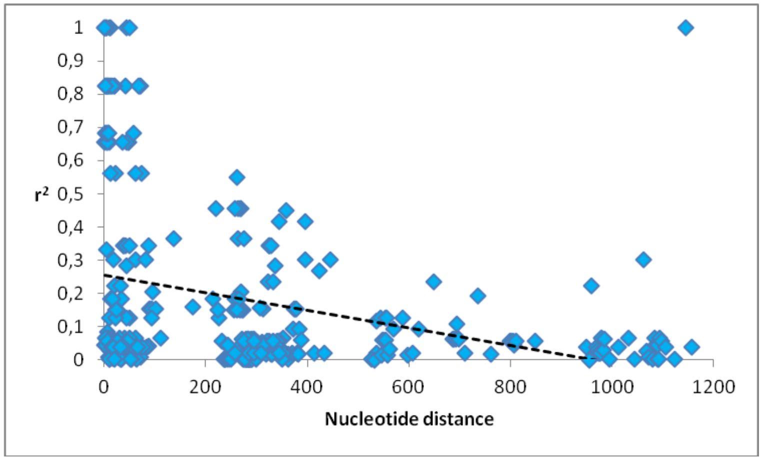

Recombination tests for the *Plasmodium vivax rbsa* gene in Colombian and Venezuelan subpopulations. The regression lines between LD and nucleotide distance are shown. LD declined as nucleotide distance increased, suggesting intragenic recombination.
