# Supplementary material for: On the Evolution and Function of Plasmodium vivax Reticulocyte Binding Surface Antigen (pvrbsa)
Source: Front Genet. 2018 Sep 10;9:372. doi: 10.3389/fgene.2018.00372 (PMC6139305; doi:10.3389/fgene.2018.00372)
Supplement: Supplementary file 6 [file Data_Sheet_3.pdf]

Supplementary Data Sheet 3. On the evolution and function of *Plasmodium vivax* reticulocyte binding surface antigen (*pvrbsa*)

Paola Andrea Camargo-Ayala, Diego Garzón-Ospina, Darwin Andrés Moreno-Pérez, Laura Alejandra Ricaurte-Contreras, Oscar Noya, Manuel A. Patarroyo*

*** Correspondence:** mapatarr.fidic@gmail.com

AMOVA analysis and inter-population F_{ST} values for non-synonymous and synonymous datasets.

$F_{ST}^{Non-syn}$		
**Source of variation**	**% of variation**	**P- value**
**Between populations (F_{CT})**	2.85	0.061
**Amongst subpopulations within populations (F_{SC})**	3.93	0.000
**Amongst subpopulations (F_{ST})**	93.21	0.000

Fst

	**Meta**	**Chocó**	**Amazonas**	**Córdoba**	**Bolívar**	**Coastal area**
**Meta**		0.32715	0.00391	0.01367	0.00293	0.03125
**Chocó**	0.00460		0.00684	0.01074	0.00098	0.01855
**Amazonas**	0.07397*	0.03688*		0.00000	0.00000	0.00000
**Córdoba**	0.04013*	0.02756*	0.08040*		0.00000	0.00000
**Bolívar**	0.05668*	0.05116*	0.08336*	0.06487*		0.14746
**Coastal area**	0.04170*	0.03657*	0.08821*	0.06487*	0.01369	

F_{ST}^{Syn}		
**Source of variation**	**% of variation**	**P- value**
**Between populations (F_{CT})**	6.70	0.064
**Amongst subpopulations within populations (F_{SC})**	2.59	0.019
**Amongst subpopulations (F_{ST})**	90.71	0.000

Fst

	**Meta**	**Chocó**	**Amazonas**	**Córdoba**	**Bolívar**	**Coastal area**
**Meta**		0.09863	0.01953	0.01172	0.02832	0.00781
**Chocó**	0.04338		0.15332	0.20898	0.00977	0.00488
**Amazonas**	0.08114*	0.01569		0.09082	0.00098	0.00098
**Córdoba**	0.10102*	0.01372	0.02589		0.00098	0.00000
**Bolívar**	0.06661*	0.06218*	0.07859*	0.07804*		0.5595
**Coastal area**	0.12931*	0.09241*	0.11377*	0.10167*	-0.00907	

F_{ST} was calculated for parasite subpopulations in Colombian and Venezuela taking just the non-synonymous (or synonymous) mutations into account. Values close to 0 indicated low genetic differentiation whilst values close to 1 indicated high genetic differentiation. Values below the diagonal were the F_{ST} value and those above the diagonal represent the respective *p* values. *: Statistically significant values. $F_{ST}^{Non-syn}$: F_{ST} computed by using just the non-synonymous mutations. F_{ST}^{Syn}: F_{ST} computed by using just the synonymous mutations.
